# Supplementary material for: Chiral Isochalcogenourea‐Catalysed Enantioselective (4+2) Cycloadditions of Allenoates
Source: Angew Chem Weinheim Bergstr Ger. 2023 Dec 11;136(2):e202315345. doi: 10.1002/ange.202315345 (PMC10976662; doi:10.1002/ange.202315345)
Supplement: Supplementary file 1 — Supporting Information [file ANGE-136-0-s001.pdf]

Angewandte Chemie
Eine Zeitschrift der Gesellschaft Deutscher Chemiker

Supporting Information

Chiral Isochalcogenourea-Catalysed Enantioselective (4+2) Cycloadditions of Allenoates

L. S. Vogl, P. Mayer, R. Robiette, M. Waser**

1. General information 2
2. Starting materials and catalysts 3
2.1. Catalysts 3
2.2. Syntheses of benzylidene indanediones 2 4
2.2.1. Synthesis of 1H-indene-1,3(2H)-diones 4
2.2.2. General procedure F for the synthesis of Benzylidene indanediones (2a-h) 8
2.2.3. General procedure G for the synthesis of Benzylidene indanediones (2i-t) 11
2.3. Syntheses of Allenoates (1) 14
2.3.1. General procedure A for the synthesis of phosphonium salts (21) 14
2.3.2. General procedure B for the synthesis of Wittig reagents (22) 15
2.3.3. General procedure C for the synthesis of allenoates (1) 16
3. (4+2)-Cycloaddition reactions 18
3.1. General procedure D for the syntheses of products 3 18
3.2. Analytical details of products 3 18
3.3. Robustness Screen 33
4. Further Transformations 34
4.1. Hydrolysis of tert. butyl (S)-6-oxo-5-phenyl-5,6-dihydro-4H-indeno [1,2-b] oxepine-2-carboxylate 34
4.2. Hydrogenations of ethyl (S,Z)-2-(5-oxo-4-phenyl-4,5-dihydroindeno[1,2-b]pyran-2(3H)-ylidene)acetate 35
4.2.1. Hydrogenation utilizing $LiBH_4$ 35
4.2.2. Hydrogenation utilizing $LiAlH_4$ 37
4.2.1. Hydrogenation utilizing the Wilkinson catalyst 39
5. Crystallographic data 40
6. References 42
7. NMR Spectra of New Compounds 44
8. Copies of HPLC chromatograms 92
9. Computational methods 152

1. General information

^{1}H-, ^{13}C- and ^{19}F-NMR spectra were recorded on a Bruker Avance III 300 MHz spectrometer with a broad band observe probe and a sample changer for 16 samples, on a Bruker Avance DRX 500 MHz spectrometer, and on a Bruker Avance III 700 MHz spectrometer with an Ascend magnet and TCI cryoprobe, which are both property of the Austro-Czech NMR-Research Center "RERI-uasb". The measurements were referenced on the solvent residual peak ($CDCl_3$: δ 7.26 ppm for ^{1}H-NMR and δ 77.16 ppm for ^{13}C-NMR; DMSO-d_6: 2.50 ppm for ^{1}H-NMR). NMR data are reported as follows: chemical shift (*δ* ppm), multiplicity (s = singlet, d = doublet, t = triplet, q = quartet, m = multiplet, br = broad), coupling constants (Hz) and integrals.

High resolution mass spectra were obtained using an Agilent QTOF 6520 with ESI source. Optical rotations were measured on a Schmidt+Haensch Unipol L 100 polarimeter ($[\alpha]_D$ values are listed in $deg \cdot cm^3 \cdot g^{-1} \cdot dm^{-1}$; concentration *c* is given in g/100 mL).

Preparative column chromatography was carried out using Davisil LC 60A 70– 200 MICRON silica gel. Thin layer chromatography was performed on Macherey-Nagel pre-coated TLC plates (silica gel, 60 F254, 0.20 mm, ALUGRAM® Xtra SIL). TLC plates were visualized under 254 nm UV lamp. Preparative thin layer chromatography was performed on Macherey-Nagel pre-coated TLC plates (silica gel, SIL-G50 F254, 0.5 mm, glass plates).

Diastereomers were separated via a semi preparative HPLC using a Dionex UltiMate 3000 HPLC system with a Grace Alltima Silica 10µ (250 × 10 mm, 10 µm) column.
Enantiomeric ratios (e.r.) were determined by HPLC analysis using a Dionex Summit HPLC system with a CHIRALPAK AD-H, OD-H, CHIRAL ART Amylose-SA, Cellulose-SB, or Cellulose-SZ (250 × 4.6 mm, 5 µm) chiral stationary phase.

Melting points were determined on a BÜCHI Melting Point apparatus M-560.

2. Starting materials and catalysts

2.1. Catalysts

ITU2 ([885051-07-0]), **ITU3** ([16595-80-5]), **ITU4** ([1316861-19-4]), and **ITU5** ([1203507-02-1]) are commercially available from standard suppliers and were used in the purchased quality without any further purification or treatment. Achiral **ITU1** was synthesized as described previously [1]. **IOU** and **ISeU** were recently introduced by Smith's group and synthesized as described by them [2].

Analytical details for **ITU1** [1]:

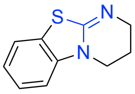

^{1}H-NMR (300 MHz, $CDCl_3$, 298 K): δ / ppm = 1.97-2.04 (m, 2 H), 3.55 (t, J = 5.6 Hz, 2 H), 3.75 (t, J = 6.1 Hz, 2 H), 6.72 (app. d, J = 8.0 Hz, 1 H), 6.97 (dt, J_1 = 7.6 Hz, J_2 = 1.0 Hz, 1 H), 7.17 (dt, J_1 = 7.8 Hz, J_2 = 1.2 Hz, 1 H), 7.26 (dd, J = 7.6 Hz, J_2 = 1.0 Hz, 1 H). ^{13}C-NMR (75 MHz, $CDCl_3$, 298 K): δ / ppm = 19.7, 42.0, 45.6, 107.3, 121.6, 121.8, 122.5, 125.9, 141.0, 157.9.

HRMS (ESI): m/z calc. for $C_{10}H_{10}N_2S$: 291.0637 $[M+H]^+$; found: 191.0640

Analytical details for **IOU** [2]:

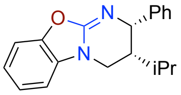

^{1}H-NMR (300 MHz, $CDCl_3$, 298 K): δ / ppm = 0.83 (d, J = 6.6 Hz, 3 H), 1.13 (d, J = 6.6 Hz, 3 H), 1.26-1.41 (m, 1 H), 2.00-1.90 (m, 1 H), 3.43 (t, J = 11.3 Hz, 1 H), 3.89 (ddd, J_1 = 11.5 Hz, J_2 = 5.4 Hz, J_3 = 1.5 Hz, 1 H), 4.96 (dd, J_1 = 4.2 Hz, J_2 = 1.5 Hz, 1 H), 6.84 (dd, J_1 = 7.5 Hz, 1.0 Hz, 1 H), 7.01 (dt, J_1 = 7.8 Hz, J_2 = 1.2 Hz, 1 H), 7.09-7.34 (m, 7 H). ^{13}C-NMR (75 MHz, $CDCl_3$, 298 K): δ / ppm = 20.3, 22.2, 26.9, 40.8, 41.8, 59.7, 106.0, 109.6, 121.5, 123.5, 127.4, 128.0 (2 C), 128.5 (2 C), 132.9, 140.9, 145.0, 155.1.

HRMS (ESI): m/z calc. for $C_{19}H_{20}N_2O$: 293.1648 $[M+H]^+$; found: 293.1652

Analytical details for **ISeU** [2]:

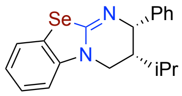

^{1}H-NMR (500 MHz, $CDCl_3$, 298 K): δ / ppm = 0.78 (d, J = 6.7 Hz, 3 H,), 1.07 (d, J = 6.7 Hz, 3 H), 1.17-1.29 (m, 1 H), 1.89-1.95 (m, 1 H), 3.26 (t, J = 11.5 Hz, 1 H), 3.78 (ddd, J_1 = 11.5 Hz, J_2 = 5.2 Hz, J_3 = 1.6 Hz, 1 H), 4.86 (dd, J_1 = 4.2 Hz, J_1 = 1.6 Hz, 1 H), 6.72 (d, J = 8.0 Hz, 1 H), 6.94 (dt, J_1 = 7.6 Hz, J_2 = 0.9 Hz, 1 H), 7.14-7.25 (m, 6 H), 7.34 (dd, J_1 = 7.6 Hz, J_2 = 0.9 Hz, 1 H). ^{13}C-NMR (125 MHz, $CDCl_3$, 298 K): δ / ppm = 20.1, 22.1, 27.1, 41.2, 42.7, 62.2, 109.0, 121.6, 122.3, 125.6, 126.5, 127.3, 128.2 (2 C), 128.4 (2C), 140.3, 142.3, 156.7. ^{77}Se-NMR (95 MHz, $CDCl_3$, 298 K): δ / ppm = 410.0.

HRMS (ESI): m/z calc. for $C_{19}H_{20}N_2Se$: 357.0864 $[M+H]^+$; found: 357.0867

2.2. Syntheses of benzylidene indanediones 2

2.2.1. Synthesis of 1H-indene-1,3(2H)-diones

2.2.1.1. Synthesis of Dimethyl 4,5-dimethylphthalate (13)

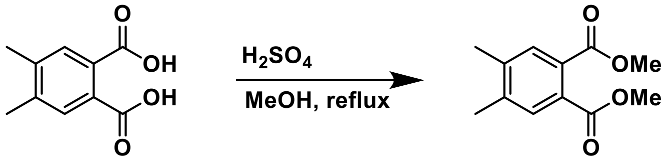

12a 13a

Product **13a** was synthesised by Fischer esterification on 5.5 mmol scale. 4,5-Dimethylphthalate (1 equ., **12a**) was dissolved in methanol (3 mL/mmol) and the solution was acidified with H_2SO_4 (0.5 equ.). The mixture was then heated to reflux for 16 h. After cooling to room temperature, the acidic solution was neutralised with a few spatula tips of Na_2CO_3. The methanol was then evaporated, and the crude was then dissolved in water and DCM and extracted two more times with DCM. The combined organic phases were dried over Na_2SO_4 and subsequent evaporation of the solvent and drying in vacuo yielded the product as a white gummy residue. The synthesis was conducted on a scale of 5.5 mmol and yielded 96% product. The analytical data matched those reported in literature [3].

^{1}H-NMR (300 MHz, $CDCl_3$, 298 K): δ / ppm = 2.31 (s, 6 H), 3.88 (s, 6 H), 7.49 (s, 2 H)

2.2.1.2. Synthesis of 5,6-dimethyl-1H-indene-1,3(2H)-dione (14a)

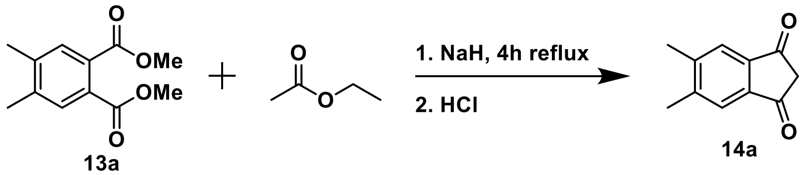

13a 14a

Product **14a** was synthesised according to literature [4] on a scale of 5.3 mmol. Dimethyl 4,5-dimethylphthalate (1 equ., **13a**) was dissolved in EtOAc (0.35 mL/mmol) and NaH (1.45 equ.) was added. The suspension was then refluxed for 4h. After cooling to room temperature, the suspension was filtered and the residue washed with EtOH:Et_2O (1:1) (10 mL). The residue was then suspended in 10 % HCl in water and heated to 80 °C for 7 min with a preheated oil bath. After cooling to room temperature, the suspension was filtered and the filtered crude washed with EtOH and dried in vacuo. Subsequent column chromatography (silica gel, DCM) gave the product as a pale yellow solid in a yield of 37 %. The analytical data matched those reported in literature [5].

^{1}H-NMR (300 MHz, $CDCl_3$, 298 K): δ / ppm = 2.44 (s, 6 H), 3.19 (s, 2 H), 7.72 (s, 2 H)

2.2.1.3. Synthesis of Diethyl naphthalene-2,3-dicarboxylate (13b)

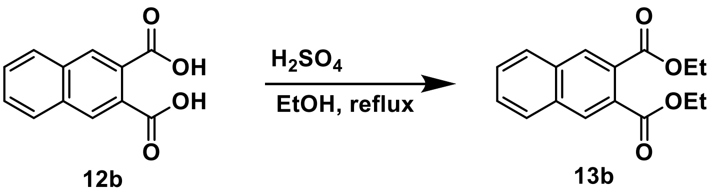

Product **13b** was synthesized by Fischer esterification in a scale of 10 mmol. Naphthalene-2,3-dicarboxylic acid (1 equ., **12b**) was dissolved in ethanol (1.5 mL/mmol) and the solution was acidified with H_2SO_4 (0.33 equ.). The mixture was then heated to reflux for 22 h. After cooling to room temperature, the acidic solution was neutralised with a few spatula tips of Na_2CO_3. The ethanol was then evaporated, and the crude was then dissolved in water and DCM and extracted two more times with DCM. The combined organic phases were dried over Na_2SO_4 and subsequent evaporation of the solvent and drying in vacuo yielded the product as a white gummy residue. Yield: 96% product. The analytical data matched those reported in literature [6].

^{1}H-NMR (300 MHz, $CDCl_3$, 298 K): δ / ppm = 1.41 (t, J = 7.2 Hz, 3 H), 4.43 (q, J =7.2 Hz, 2 H), 7.58-7.65 (m, 2 H), 7.88-7.96 (m, 2 H), 8.25 (s, 2 H)

2.2.1.4. Synthesis of 1H-cyclopenta[b]naphthalene-1,3(2H)-dione (14b)

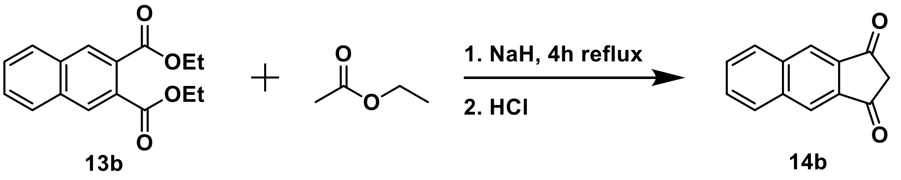

Product **14b** was synthesised according to literature [7] in a scale of 8.9 mmol. Diethyl naphthalene-2,3-dicarboxylate (1 equ., **13b**) was dissolved in EtOAc (0.6 mL/mmol) and NaH (2.5 equ.) was added. The suspension was then refluxed for 5 h. After cooling to room temperature, the suspension was filtered and the residue washed with EtOH:Et_2O (1:1) (10 mL). The residue was then suspended in 10 % HCl in water and heated to reflux for 1.5 h. After cooling to room temperature, the suspension was filtered, and the filtered crude washed with water and dried in vacuo. Subsequent recrystallisation gave the product in a yield of 37 %. The analytical data matched those reported in literature [7].

^{1}H-NMR (300 MHz, $CDCl_3$, 298 K): δ / ppm = 3.38 (s, 2 H), 7.70-7.79 (m, 2 H), 8.08-8.17 (m, 2 H), 8.52 (s, 2 H)

2.2.1.5. Synthesis of 5,6-Dimethoxy-1H-indene-1,3(2H)-dione (14c)

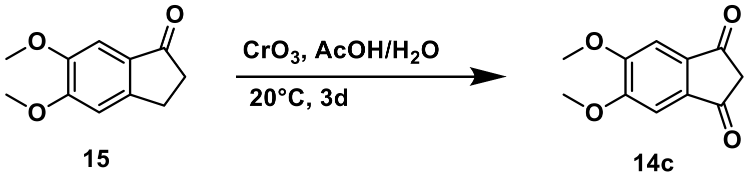

15 14c

5,6-Dimethoxy-1H-indene-1,3(2H)-dione **14c** was synthesised according to literature [8] in a scale of 2 mmol. 5,6-Dimethoxy indanone (1 equ., **15**) was dissolved in acetic acid (2.5 mL/mmol) and water (0.5 mL/mmol). The mixture was then cooled with an icebath and CrO_3 was added portionwise. The suspension was then stirred for 3 days at room temperature. The mixture was afterwards quenched with IPA and stirred for one hour before the solvent was removed. The crude residue was then dissolved in water and extracted six times with DCM. The combined organic phase was afterwards washed with water and dried with Na_2SO_4. The solvent was then concentrated to a few mL and hexanes was added to precipitate the product. The product was filtered off and dried in vacuo. 5,6-Dimethoxy-1H-indene-1,3(2H)-dione **14c** was attained as a yellow solid with a yield of 37 %. The spectral data matched those reported in literature [8].

^{1}H-NMR (300 MHz, $CDCl_3$, 298 K): δ / ppm = 3.18 (s, 2 H), 4.02 (s, 6 H), 7.33 (s, 2 H)

2.2.1.6. General procedure E for the synthesis of substituted 1H-indene-1,3(2H)-diones (14d-e)

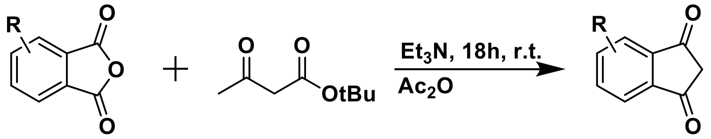

16 17 14

R = 3,4,5,6-tetrachloro; 4,5-dichloro

Products **14d** and **14e** were synthesised according to literature [9]. First the substituted phthalic anhydride (**16**, 1 equ.) was dissolved in Ac_2O (5 equ.), subsequently Et_3N (2 equ.) was added and the mixture placed in a water bath at room temperature. Afterwards, *tert*. butyl acetoacetate (**17**, 1 equ.) was added in three portions. The mixture was then stirred at r.t. for 18 h. Then ice (0.3 g/mmol **16**) was added with subsequent slow addition of conc. HCl (0.3 mL/mmol **16**). Afterwards 5 M HCl (1.4 mL/mmol **16**) were added and the mixture heated to 80 °C for 1 h. After cooling to r.t. the

mixture was extracted three times with DCM, the combined organic phase was pre-dried with Na_2SO_4 and the solvent evaporated with a rotavap to yield the product **14d** and **14e**.

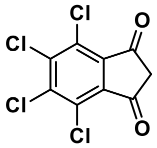

4,5,6,7-Tetrachloro-1H-indene-1,3(2H)-dione (**14d**) was prepared according to general procedure **E** on a scale of 7 mmol. The product was obtained after recrystallisation in *n*-hexane:EtOAc 1:1 with a yield of 86 %. The spectral data matched those reported in literature [10].

^{1}H-NMR (300 MHz, DMSO-d_6, 298 K): δ / ppm = 3.37 (s, 2 H)

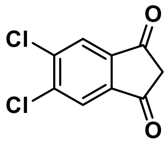

5,6-Dichloro-1H-indene-1,3(2H)-dione (**14e**) was prepared according to general procedure E on a scale of 5.9 mmol. The product was obtained after dissolving the crude in EtOAc, washing with water and evaporation of the solvent with a yield of 83 %. The spectral data matched those reported in literature [9].

^{1}H-NMR (300 MHz, DMSO-d_6, 298 K): δ / ppm = 3.27 (s, 2 H), 8.06 (s, 2 H)

2.2.2. General procedure F for the synthesis of Benzylidene indanediones (2a-h)

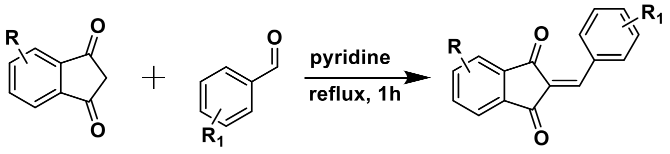

Products **2a-h** were synthesised according to literature [11]. Indanedione (**14**, 1 equ.) was dissolved in pyridine (5 mL/mmol **14**) and benzaldehyde (**18**, 1.2 equ.) was added via a syringe. The mixture was then refluxed at 100 °C for 1 h before pouring it on ice. The suspension was then filtered and washed with cold *n*-heptane to give the crude. The crude product was then dissolved in MeOH and crystallized with H_2O. After filtering the suspension (filter crucible PG 3) and drying of the residue in vacuo, it was dissolved eight times in EtOAc with subsequent evaporation of the solvent to yield the dry product as powder. Alternatively, products **2** can be purified by silica gel column chromatography where partial decomposition however may occur.

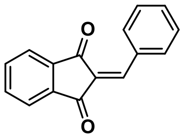

2-Benzylidene-1*H*-indene-1,3(2*H*)-diones (**2a**) was prepared according to general procedure F on a scale of 5.4 mmol. The analytical data matched those reported in literature [11]. Yield: 48 %

^{1}H-NMR (300 MHz, $CDCl_3$, 298 K): δ / ppm = 7.49-7.58 (m, 3 H), 7.80-7.87 (m, 2 H), 7.92 (s, 1 H), 7.99-8.07 (m, 2 H), 8.43-8.51 (m, 2 H)

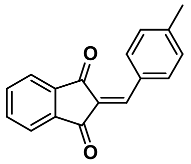

2-(4-Methylbenzylidene)-1*H*-indene-1,3(2*H*)-dione (**2b**) was prepared according to general procedure F on a scale of 6 mmol. The analytical data matched those reported in literature [12]. Yield: 79 %

^{1}H-NMR (300 MHz, $CDCl_3$, 298 K): δ / ppm = 2.46 (s, 3 H), 7.31-7.36 (m, 2 H), 7.77-7.85 (m, 2 H), 7.89 (s, 1 H), 7.97-8.05 (m, 2 H), 8.37-8.43 (m, 2 H)

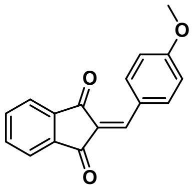

2-(4-Mthoxybenzylidene)-1*H*-indene-1,3(2*H*)-dione (**2c**) was prepared according to general procedure F on a scale of 6 mmol. The analytical data matched those reported in literature [12]. Yield: 84 %

^{1}H-NMR (300 MHz, $CDCl_3$, 298 K): δ / ppm = 3.92 (s, 3 H), 6.98-7.06 (m, 2 H), 7.77-7.83 (m, 2 H), 7.86 (s, 1 H), 7.95-8.03 (m, 2 H), 8.52-8.59 (m, 2 H)

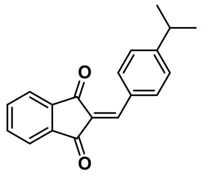

2-(4-Isopropylbenzylidene)-1*H*-indene-1,3(2*H*)-dione (**2d**) was prepared according to general procedure F on a scale of 6 mmol. The product was isolated as a dark ochre solid. Yield: 84 %

^{1}H-NMR (300 MHz, $CDCl_3$, 298 K): δ / ppm = 1.30 (d, *J* = 6.9 Hz, 6 H), 3.00 (sep, *J* = 6.9 Hz, 1 H), 7.36-7.42 (m, 2 H), 7.79-7.83 (m, 2 H), 7.90 (s, 1 H), 7.98-8.04 (m, 2 H), 8.39-8.47 (m, 2 H)

^{13}C-NMR (125 MHz, $CDCl_3$, 298 K): δ / ppm = 23.7 (2 C), 34.7, 123.4, 123.4, 127.2 (2 C), 128.4, 131.1, 134.8 (2 C), 135.2, 135.4, 140.2, 142.6, 147.3, 155.5, 189.4, 190.8

HRMS (ESI): m/z calc. for $C_{19}H_{16}O_2$: 277.1223 $[M+H]^+$; found: 277.1222

Melting point: 64.7-66.9 °C

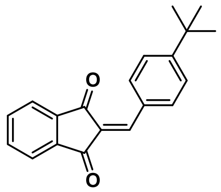

2-(4-(Tert-butyl)benzylidene)-1*H*-indene-1,3(2*H*)-dione (**2e**) was prepared according to general procedure F on a scale of 6 mmol. The product was isolated as an ochre solid. Yield: 94 %

^{1}H-NMR (300 MHz, $CDCl_3$, 298 K): δ / ppm = 1.37 (s, 9 H), 7.51-7.57 (m, 2 H), 7.78-7.84 (m, 2 H), 7.90 (s, 1 H), 7.98-8.04 (m, 2 H), 8.39-8.46 (m, 2 H)

^{13}C-NMR (125 MHz, $CDCl_3$, 298 K): δ / ppm = 23.7 (3 C), 34.7, 123.4, 123.4, 127.2 (2 C), 128.4, 131.1, 134.8 (2 C), 135.2, 135.4, 140.2, 142.6, 147.3, 155.5, 189.4, 190.8

HRMS (ESI): m/z calc. for $C_{20}H_{18}O_2$: 291.1380 $[M+H]^+$; found: 291.1378

Melting point: 108.2-117.5 °C

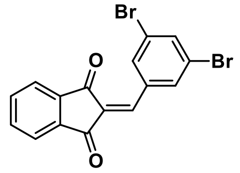

2-(3,5-Dibromobenzylidene)-1*H*-indene-1,3(2*H*)-dione (**2f**) was prepared according to general procedure F on a scale of 6 mmol. The product was isolated as a dark light beige solid. Yield: 88 %

^{1}H-NMR (300 MHz, $CDCl_3$, 298 K): δ / ppm = 7.71 (s, 1 H), 7.82-7.90 (m, 3 H), 8.00-8.09 (m, 2 H), 8.52-8.58 (m, 2 H)

^{13}C-NMR (125 MHz, $CDCl_3$, 298 K): δ / ppm = 123.3 (2 C), 123.8, 123.9, 131.5, 134.9 (2 C), 135.9, 136.0, 136.2, 137.8, 140.3, 142.7, 143.0, 188.7, 189.4

HRMS (ESI): m/z calc. for $C_{16}H_8Br_2O_2$: 392.8944 $[M+H]^+$; found: 392.8941

Melting point: 230.0-233.2 °C

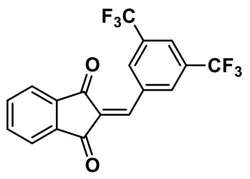

2-(3,5-bis(trifluoromethyl)benzylidene)-1*H*-indene-1,3(2*H*)-dione (**2g**) was prepared according to general procedure F on a scale of 6 mmol. The product was isolated as a dark yellow solid. Yield: 83 %

^{1}H-NMR (300 MHz, $CDCl_3$, 298 K): δ / ppm = 7.85-7.91 (m, 3 H), 8.02 (s, 1 H), 8.04-8.12 (m, 2 H), 8.91 (s, 2 H)

^{19}F-NMR (282 MHz, $CDCl_3$, 298 K): δ / ppm = -63.01

^{13}C-NMR (175 MHz, $CDCl_3$, 298 K): δ / ppm =123.2 (q, *J* = 273 Hz), 123.9, 124.0, 125.5-125.7 (m, 2 C), 132.4, 132.4 (q, *J* = 33.8 Hz, 2 C), 133.1-133.2 (m, 2 C), 134.7, 136.1, 136.3, 140.5, 142.1, 142.7, 188.6, 189.0

HRMS (ESI): m/z calc. for $C_{18}H_8F_6O_2$: 371.0501 $[M+H]^+$; found: 371.0501

Melting point: 153.5-156.0 °C

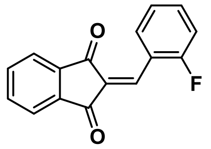

2-(2-Fluorobenzylidene)-1H-indene-1,3(2H)-dione (**2h**) was prepared according to general procedure F on a scale of 6 mmol. The product was isolated as a brown solid. Yield: 79 %

^{1}H-NMR (300 MHz, $CDCl_3$, 298 K): δ / ppm = 7.12-7.21 (m, 1 H), 7.27-7.35 (m, 1 H), 7.50-7.7.60 (m, 1 H), 7.79-7.88 (m, 2 H), 7.98-8.09 (m, 2 H), 8.24 (s, 1 H), 8.96 (td, J_1 = 7.8 Hz, J_2 = 1.7 Hz, 1 H)

^{19}F-NMR (282 MHz, $CDCl_3$, 298 K): δ / ppm = -110.52

^{13}C-NMR (125 MHz, $CDCl_3$, 298 K): δ / ppm = 115.8 (d, *J* = 22.4 Hz), 121.4 (d, *J* = 9.6 Hz), 123.6 (d, *J* = 6.5 Hz, 2 C), 124.4 (d, *J* = 4.0 Hz), 130.4, 133.9 (2 C), 135.4 (d, *J* = 9.0 Hz), 135.6 (d, *J* = 17.8 Hz, 2 C), 137.5 (d, *J* = 7.1 Hz), 140.4, 142.6, 189.0, 189.8

HRMS (ESI): m/z calc. for $C_{16}H_9FO_2$: 253.0660 $[M+H]^+$; found: 253.0657

Melting point: 126.4-128.1 °C

2.2.3. General procedure G for the synthesis of Benzylidene indanediones (2i-t)

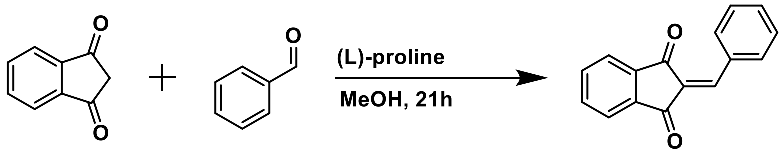

14 18 2

Products **2i-t** were synthesised according to literature [12]. Indanedione (1 equ., **14**) was dissolved in methanol (2 mL/mmol) and subsequently (*L*)-proline (0.33 equ.) and benzaldehyde (1.1 equ., **18**) were added. The mixture was then stirred at room temperature for 21 h. The precipitate, which formed, was filtered off and washed with cold methanol and dried in vacuo. After initial drying the product was dissolved in EtOAc with subsequent evaporation of the solvent giving products **2** as dry powders. The products **2i-t** can be purified by column chromatography too but accompanied with decomposition on silica gel.

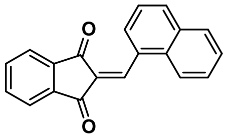

2-(Naphthalen-1-ylmethylene)-1*H*-indene-1,3(2*H*)-dione (**2i**) was prepared according to general procedure G on a scale of 6 mmol. The analytical data matched those reported in literature [13]. Yield: 79 %

^{1}H-NMR (300 MHz, DMSO-d$_6$, 298 K): δ / ppm = 7.62-7.73 (m, 3 H), 7.94-8.03 (m, 3 H), 8.04-8.11 (m, 2 H), 8.16-8.23 (m, 2 H), 8.50-8.57 (m, 2 H), 8.57 (s, 1 H)

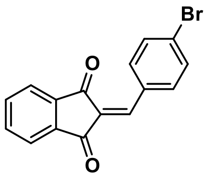

2-(4-Bromobenzylidene)-1*H*-indene-1,3(2*H*)-dione (**2j**) was prepared according to general procedure G on a scale of 6 mmol. The analytical data matched those reported in literature [12]. Yield: 82 %

^{1}H-NMR (300 MHz, DMSO-d$_6$, 298 K): δ / ppm = 7.76-7.83 (m, 2 H), 7.84 (s, 1 H), 7.93-8.06 (m, 4 H), 8.40-8.48 (m, 2 H)

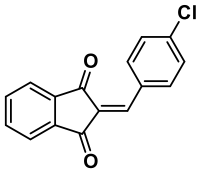

2-(4-Chlorobenzylidene)-1*H*-indene-1,3(2*H*)-dione (**2k**) was prepared according to general procedure G on a scale of 6 mmol. The analytical data matched those reported in literature [13]. Yield: 88 %

^{1}H-NMR (300 MHz, DMSO-d$_6$, 298 K): δ / ppm = 7.62-7.69 (m, 2 H), 7.86 (s, 1 H), 7.94-8.05 (m, 4 H), 8.50-8.57 (m, 2 H)

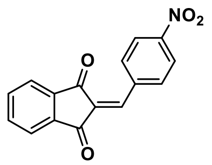

2-(4-Nitrobenzylidene)-1*H*-indene-1,3(2*H*)-dione (**2l**) was prepared according to general procedure G on a scale of 6 mmol. The analytical data matched those reported in literature [12]. Yield: 90 %

^{1}H-NMR (300 MHz, DMSO-d$_6$, 298 K): δ / ppm = 7.96 (s, 1 H), 7.97-8.09 (m, 4 H), 8.31-8.40 (m, 2 H), 8.56-8.66 (m, 2 H)

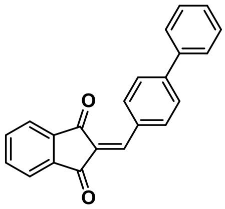

2-([1,1'-Biphenyl]-4-ylmethylene)-1*H*-indene-1,3(2*H*)-dione (**2m**) was prepared according to general procedure G on a scale of 6 mmol. The analytical data matched those reported in literature [13]. Yield: 52 %

^{1}H-NMR (300 MHz, DMSO-d$_6$, 298 K): δ / ppm = 7.44-7.48 (m, 1 H), 7.50-7.67 (m, 2 H), 7.80-7.87 (m, 2 H), 7.89-7.95 (m, 3 H), 7.97-8.03 (m, 4 H), 8.60-8.68 (m, 2 H)

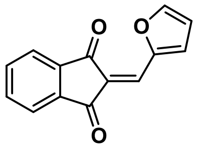

2-(Furan-2-ylmethylene)-1*H*-indene-1,3(2*H*)-dione (**2n**) was prepared according to general procedure G on a scale of 6 mmol. The analytical data matched those reported in literature [11]. Yield: 75 %

^{1}H-NMR (300 MHz, CDCl$_3$, 298 K): δ / ppm = 6.73 (ddd, J_1 = 3.7 Hz, J_2 = 1.6 Hz, J_3 = 0.7 Hz, 1 H), 7.75-7.82 (m, 4 H), 7.93-8.02 (m, 2 H), 8.59 (d, J = 3.7 Hz, 1 H)

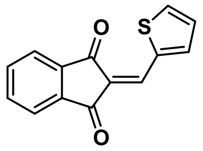

2-(Thiophen-2-ylmethylene)-1*H*-indene-1,3(2*H*)-dione (**2o**) was prepared according to general procedure G on a scale of 6 mmol. The analytical data matched those reported in literature [12]. Yield: 85 %

^{1}H-NMR (300 MHz, CDCl$_3$, 298 K): δ / ppm = 7.23-7.27 (m, 1 H), 7.77-7.82 (m, 2 H), 7.85-7.90 (m, 1 H), 7.96-8.02 (m, 2 H), 8.03 (s, 1 H), 8.06-8.09 (m, 1 H)

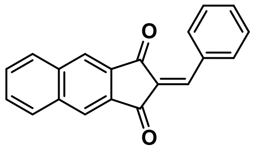

2-Benzylidene-1H-cyclopenta[b]naphthalene-1,3(2H)-dione (**2p**) was prepared according to general procedure G on a scale of 6 mmol. The analytical data matched those reported in literature [14]. Yield: 95 %

^{1}H-NMR (300 MHz, CDCl$_3$, 298 K): δ / ppm = 7.50-7.64 (m, 3 H), 7.67-7.76 (m, 2 H), 8.01 (s, 1 H), 8.08-8.16 (m, 2 H), 8.50-8.60 (m, 4 H)

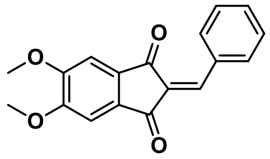

2-Benzylidene-5,6-dimethoxy-1H-indene-1,3(2H)-dione (**2q**) was prepared according to general procedure G on a scale of 6 mmol. The analytical data matched those reported in literature [15]. Yield: 86 %

^{1}H-NMR (300 MHz, $CDCl_3$, 298 K): δ / ppm = 4.04-4.08 (m, 6 H), 7.37-7.43 (m, 2 H), 7.45-7.56 (m, 3 H), 7.76 (s, 1 H), 8.37-8.45 (m, 2 H)

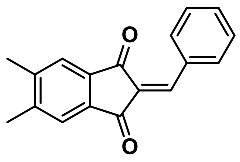

2-Benzylidene-5,6-dimethyl-1H-indene-1,3(2H)-dione (**2r**) was prepared according to general procedure G on a scale of 2 mmol. The product was isolated as a bright yellow solid. Yield: 81 %

^{1}H-NMR (300 MHz, $CDCl_3$, 298 K): δ / ppm = 2.46 (s, 6 H), 7.46-7.55 (m, 3 H), 7.77 (s, 2 H), 7.84 (s, 1 H), 8.40-8.49 (m, 2 H)

^{13}C-NMR (175 MHz, $CDCl_3$, 298 K): δ / ppm = 21.0, 21.1, 124.1, 124.1, 128.9 (2 C), 130.1, 132.9, 133.4, 134.1 (2 C), 138.8, 141.3, 145.8, 145.9, 146.1, 189.3, 190.5

HRMS (ESI): m/z calc. for $C_{18}H_{14}O_2$: 263.1067 $[M+H]^+$; found: 263.1068

Melting point: 224.8-227.7 C

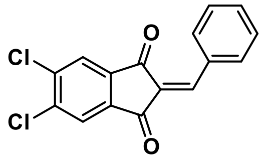

2-Benzylidene-5,6-dichloro-1H-indene-1,3(2H)-dione (**2s**) was prepared according to general procedure G on a scale of 4.8 mmol. The product was isolated as a light green solid. Yield: 62 %

^{1}H-NMR (300 MHz, $CDCl_3$, 298 K): δ / ppm = 7.49-7.65 (m, 3 H), 7.93 (s, 1 H), 8.08 (s, 2 H), 8.40-8.50 (m, 2 H)

^{13}C-NMR (175 MHz, $CDCl_3$, 298 K): δ / ppm = 125.4, 125.5, 128.4, 129.1 (2 C), 133.0, 134.0, 134.6 (2 C), 138.9, 140.4, 140.6, 141.3, 148.9, 186.9, 188.3

HRMS (ESI): m/z calc. for $C_{16}H_8Cl_2O_2$: 302.9974 $[M+H]^+$; found: 302.9968

Melting point: 208.2-210.7 °C

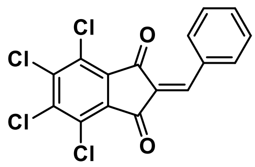

2-Benzylidene-4,5,6,7-tetrachloro-1H-indene-1,3(2H)-dione (**2t**) was prepared according to general procedure G on a scale of 3.1 mmol. The product was isolated as a light-yellow solid. Yield: 78 %

^{1}H-NMR (300 MHz, $CDCl_3$, 298 K): δ / ppm = 7.50-7.66 (m, 3 H), 7.99 (s, 1 H), 8.41-8.52 (m, 2 H)

^{13}C-NMR (175 MHz, $CDCl_3$, 298 K): δ / ppm = 127.3, 129.2 (2 C), 130.0, 130.3, 132.7, 134.5, 134.9 (2 C), 135.0, 136.6, 141.1, 141.3, 150.6, 184.2, 185.1

HRMS (ESI): m/z calc. for $C_{16}H_6Cl_4O_2$: 372.9165 $[M+H]^+$; found: 372.9162

Melting point: 258.0-260.7

2.3. Syntheses of Allenoates (1)

2.3.1. General procedure A for the synthesis of phosphonium salts (21)

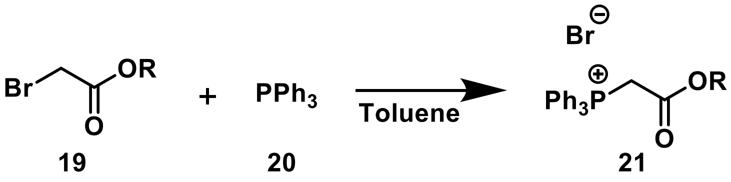

The phosphonium salts **21** were synthesized according to literature [16]. First triphenylphosphine (1 equ., **20**) was dissolved in toluene (3 mL/mmol **20**). Then the corresponding bromo acetic ester (1.1 equ., **19**) was dissolved in toluene (0.5 mL/mmol **19**) and added dropwise. The solution was stirred over night at room temperature. The product crystallized and formed a white suspension, which was filtered, washed with toluene, and dried in vacuo to yield a white solid.

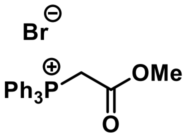

(2-Methoxy-2-oxoethyl)triphenylphosphonium bromide (**21a**) was synthesised in accordance with general procedure A on a scale of 30 mmol. The product was obtained as a white powder in a yield of 85 %. It was used for the next step without further purification. The analytical data matched those reported in literature [17].

^{1}H-NMR (300 MHz, $CDCl_3$, 298 K): δ / ppm = 3.59 (s, 3 H), 5.62 (d, J = 13.5 Hz, 2 H), 7.62-7.71 (m, 6 H), 7.74-7.82 (m, 3 H), 7.84-7.94 (m, 6 H)

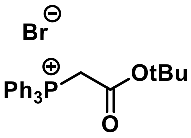

(2-(*tert*.-Butoxy)-2-oxoethyl)triphenylphosphonium bromide (**21b**) was synthesised in accordance with general procedure A on a scale of 30 mmol. The product was obtained as a white powder with a yield of 99 %. It was used for the next step without further purification. The analytical data matched those reported in literature [18].

^{1}H-NMR (300 MHz, $CDCl_3$, 298 K): δ / ppm = 1.21 (s, 9H), 5.44 (d, J = 14.0 Hz, 2 H), 7.64-7.71 (m, 6 H), 7.74-7.81 (m, 3 H), 7.87-7.97 (m, 6 H)

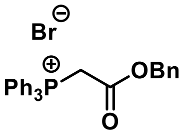

(2-(Benzyloxy)-2-oxoethyl)triphenylphosphonium bromide (**21c**) was synthesised in accordance with general procedure A on a scale of 30 mmol. The product was obtained as a white powder with a yield of 87 %. It was used for the next step without further purification. The analytical data matched those reported in literature [18].

^{1}H-NMR (300 MHz, $CDCl_3$, 298 K): δ / ppm = 5.02 (s, 2 H), 5.67 (d, J = 13.9 Hz, 2 H), 7.08-7.16 (m, 2 H), 7.21-7.34 (m, 3 H), 7.56-7.66 (m, 6 H), 7.70-7.87 (m, 9 H)

2.3.2. General procedure B for the synthesis of Wittig reagents (22)

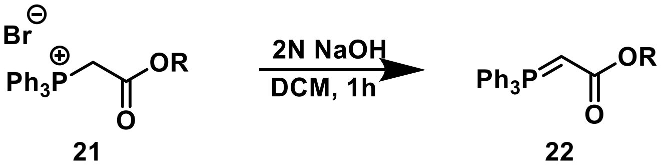

The Wittig reagents **22** were synthesized according to literature [16]. The phosphonium salt (**22**) was dissolved in DCM (3 mL/mmol **21**) then aqueous NaOH (2 M, 2 equ.) was added and the mixture stirred vigorously for 1 hour. After phase separation, the aqueous phase was extracted 3 times with DCM. The combined organic phases were washed with brine and dried over Na_2SO_4 and evaporated to dryness to yield the product **22** as an off-white solid.

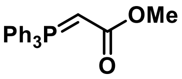

Methyl 2-(triphenylphosphaneylidene) acetate (**22a**) was synthesised in accordance with general procedure B on a scale of 26 mmol. The product was obtained as an off-white solid with a yield of 99 %. It was used for the next step without further purification [17].
^{1}H-NMR (300 MHz, $CDCl_3$, 298 K): δ / ppm = 2.90 (br s, 1 H), 3.53 (br s, 3 H,), 7.42-7.50 (m, 6 H), 7.51-7.58 (m, 3 H), 7.61-7.70 (m, 6 H)

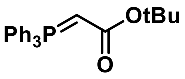

tert. Butyl 2-(triphenylphosphaneylidene) acetate (**22b**) was synthesised in accordance with general procedure B on a scale of 30 mmol. The product was obtained as an off-white solid with a yield of 89 %. It was used for the next step without further purification. The analytical data matched those reported in literature [18].
^{1}H-NMR (300 MHz, $CDCl_3$, 298 K): δ / ppm = 1.21 (br s, 9H), 2.67 (br s, 1 H) 7.38-7.59 (m, 9 H), 7.60-7.72 (m, 6 H)

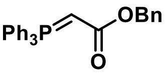

Benzyl 2-(triphenylphosphaneylidene) acetate (**22c**) was synthesised in accordance with general procedure B on a scale of 26 mmol. The product was obtained as an off-white solid with a yield of 98 %. It was used for the next step without further purification. The analytical data matched those reported in literature [18].
^{1}H-NMR (300 MHz, $CDCl_3$, 298 K): δ / ppm = 2.97 (br s, 1H), 5.02 (br s, 2 H) 7.12-7.67 (m, 20 H)

2.3.3. General procedure C for the synthesis of allenoates (1)

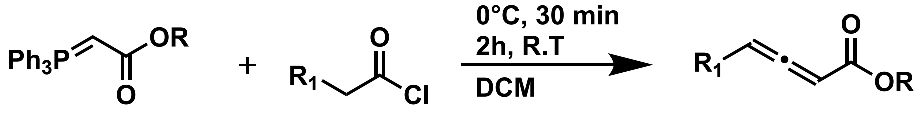

22 23 1

The allenoates **1** were synthesised according to literature [19]. The Wittig reagent (1 equ., **22**) was dissolved in DCM (2 mL/mmol **22**) and Et_3N (1 equ.) was added. Subsequently the acyl chloride (1 equ., **23**) was added over the course of 20 minutes. After addition, the reaction mixture was stirred at room temperature for 3 h, and then concentrated to 1/3 of its original volume via distillation with a Hempel column. Next, *n*-hexane (3 mL/mmol **22**) and silica gel (0.17 g/mmol **22**) were added to the solution and the suspension was stirred for 1 h. Afterwards the mixture was passed through a column filled with silica gel (13 cm filling level, 3 cm in diameter) and washed off the column with Et_2O:*n*-hexane 1:20 (200 mL). Finally, the allenoate was acquired by removal of the solvent via distillation (1 atm) in a distillation apparatus with a Hempel column (31 cm height, 5 mm glass beads), closed via a balloon and a temperature gradient (starting at 60 °C, then going up to 90 °C). Subsequent ambient pressure Kugelrohr distillation with a temperature gradient (starting at 60 °C, then going up to 90 °C) and in a closed system (with ballon) to remove the residual solvent yields the allenoate **1** as a liquid.

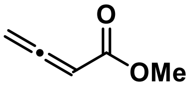

Methyl buta-1,2-dienoate (**1b**) was prepared according to general procedure C on a scale of 26 mmol. The product was obtained as a colourless liquid with a yield of 39%. The analytical data was similar to those reported in literature [19].

^{1}H-NMR (300 MHz, $CDCl_3$, 298 K): δ / ppm = 3.75 (s, 3H), 5.22 (d, J = 6.5 Hz, 2 H), 5.64 (t, J = 6.6 Hz, 1 H)

^{13}C-NMR (75 MHz, $CDCl_3$, 298 K): δ / ppm = 52.3, 79.4, 87.8, 166.3, 216.0

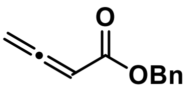

Benzyl buta-1,2-dienoate (**1c**) was prepared according to general procedure C on a scale of 26 mmol. The product was obtained as a colourless liquid with a yield of 62 %. The analytical data matched those reported in literature [19].

^{1}H-NMR (300 MHz, $CDCl_3$, 298 K): δ / ppm = 5.20 (s, 2H), 5.24 (d, J = 6.5 Hz, 2 H),
5.69 (t, J = 6.5 Hz, 1 H), 7.28-7.43 (m, 5 H)

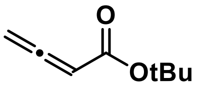

tert. Butyl buta-1,2-dienoate (**1d**) was prepared according to general procedure C on a scale of 27 mmol. The product was obtained as a colourless liquid with a yield of 22 %. The analytical data matched those reported in literature [19].

^{1}H-NMR (300 MHz, $CDCl_3$, 298 K): δ / ppm = 1.48 (s, 9H), 5.16 (d, J = 6.5 Hz, 2 H), 5.54 (t, J = 6.5 Hz, 1 H)

^{13}C-NMR (75 MHz, $CDCl_3$, 298 K): δ / ppm = 28.1, 79.0, 81.1, 89.6, 165.1, 215.4

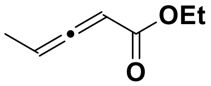

Ethyl penta-2,3-dienoate (**1e**) was prepared according to general procedure *C* on a scale of 33.5 mmol. The product was obtained as a colourless liquid with a yield of 63 %. The analytical data matched those reported in literature [19].

^{1}H-NMR (300 MHz, $CDCl_3$, 298 K): δ / ppm = 1.26 (t, *J* = 7.1 Hz, 3 H), 1.76 (dd, *J* = 3.5 Hz, 3 H), 4.17 (q, *J* = 7.1 Hz, 2 H), 5.49-5.62 (m, 2 H)

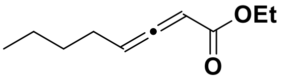

Ethyl octa-2,3-dienoate (**1f**) was prepared according to general procedure C on a scale of 35.2 mmol. The product was obtained as a colourless liquid with a yield of 68 %.

^{1}H-NMR (700 MHz, $CDCl_3$, 298 K): δ / ppm = 0.89 (t, *J* = 7.3 Hz, 3 H), 1.26 (t, *J* = 7.1 Hz, 3 H), 1.34-1.40 (m, 2 H), 1.40-1.45 (m, 2 H), 2.12 (dq, J_1 = 7.2 Hz, *J* = 3.0 Hz, 2 H), 4.14-4.21 (m, 2 H), 5.55 (quin, *J* = 3.0 Hz, 1 H), 5.59 (q, *J* = 5.7 Hz, 1 H)

^{13}C-NMR (175 MHz, $CDCl_3$, 298 K): δ / ppm = 13.9, 14.4, 22.1, 27.3, 30.9, 60.9, 88.3, 95.5, 166.5, 212.5

HRMS (ESI): m/z calc. for $C_{10}H_{16}O_2$: 169.1223 $[M+H]^+$; found: 169.1212

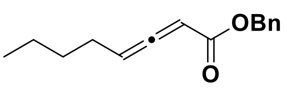

Benzyl octa-2,3-dienoate (**1g**) was prepared according to general procedure *C* on a scale of 19.4 mmol. The product was obtained as a colourless liquid with a yield of 78 %.

^{1}H-NMR (300 MHz, $CDCl_3$, 298 K): δ / ppm = 0.89 (t, *J* = 7.2 Hz, 3 H), 1.31-1.49 (m, 4 H), 2.07-2.20 (m, 2 H), 5.13-5.24 (m, 2 H), 5.58-5.67 (m, 2 H), 7.27-7.43 (m, 5 H)

^{13}C-NMR (125 MHz, $CDCl_3$, 298 K): δ / ppm = 13.9, 22.2, 27.3, 31.0, 66.6, 88.2, 95.7, 128.2 (2 C), 128.3, 128.6 (2 C), 136.2, 166.3, 212.8

HRMS (ESI): m/z calc. for $C_{15}H_{18}O_2$: 231.138 $[M+H]^+$; found: 231.1376

3. (4+2)-Cycloaddition reactions

3.1. General procedure D for the syntheses of products 3

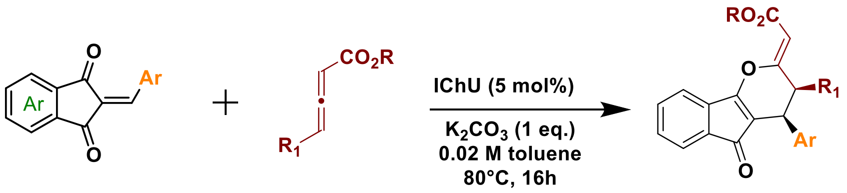

In a flame dried Schlenk tube, under N_2 atmosphere, starting materials **2** (1 equ.), catalysts **ITU5** or **ISeU** (5 mol%) and K_2CO_3 (1 equ.) were dissolved in dry toluene (0.02 M). Via a Hamilton syringe, the corresponding allenoate **1** (1.5 equ.) was added, the Schlenk tube closed and heated to 80 °C for 16 h. After cooling to room temperature, the mixture was filtered over Na_2SO_4 and washed with DCM before evaporation of the solvent via a rotavap. The obtained crude product was purified by preparative TLC (0.5 mm silica gel, 20x20 cm, *n*-heptane:EtOAc 2:1) or silica gel column chromatography.

3.2. Analytical details of products 3

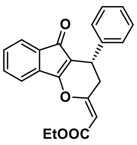

Ethyl (S)-6-oxo-5-phenyl-5,6-dihydro-4H-indeno [1,2-b] oxepine-2-carboxylate (**3a**) was synthesised according to general procedure D. The synthesis was carried out on a scale of 1 mmol and gave the product in 86% and 95:5 er using **ITU5** and 88% and 98:2 er using **ISeU**.

HPLC (YMC-SA, n-hexane/IPA = 4/1, flow = 0.5 mL min-1, T_{Column} = 10 °C, *l* = 220 nm): t_R: 16.5 min (minor), 20.8 min (major).

^{1}H-NMR (500 MHz, $CDCl_3$, 298 K): δ / ppm = 1.26 (t, J = 7.1 Hz, 3 H), 2.60 (dd, J_1 = 14.8 Hz, J_2 = 2.8 Hz, 1 H), 2.96 (ddd, J_1 = 14.8 Hz, J_2 = 6.9 Hz, J_3 = 1.6 Hz, 1 H), 3.97 (dd, J_1 = 7.0 Hz, J_2 = 2.8 Hz, 1 H), 4.16 (q, J = 7.0 Hz, 2 H), 5.18 (d, J = 1.5 Hz, 1 H), 7.11-7.18 (m, 3 H), 7.20-7.27 (m, 3 H), 7.27-7.39 (m, 3 H)

^{13}C-NMR (125 MHz, $CDCl_3$, 298 K): δ / ppm = 14.5, 33.1, 35.8, 60.4, 103.9, 111.1, 118.7, 122.1, 127.3 (2 C), 127.4, 128.9 (2 C), 130.6, 132.5, 132.7, 136.6, 141.2, 159.0, 163.8, 170.5, 191.4

HRMS (ESI): m/z calc. for $C_{22}H_{18}O_4$: 347.1278 $[M+H]^+$; found: 347.1264

$[\alpha]_D^{20}$= -129.8 (*c* 1.0, $CHCl_3$, 97:3 *e.r.*)

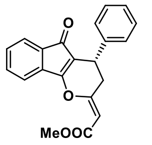

Methyl (S)-6-oxo-5-phenyl-5,6-dihydro-4H-indeno [1,2-b] oxepine-2-carboxylate (**3b**) was synthesised according to general procedure D. The synthesis was carried out on a scale of 0.1 mmol and gave the product in 76% and 95:5 er using **ITU5** and 94% and 96:4 er using **ISeU**.

HPLC (YMC-SA, n-hexane/IPA = 4/1, flow = 0.5 mL min-1, T_{Column} = 10 °C, *l* = 220 nm): t_R: 15.9 min (minor), 23.6 min (major).

^{1}H-NMR (300 MHz, $CDCl_3$, 298 K): δ / ppm = 2.56 (dd, J_1 = 14.8 Hz, J_2 = 2.8 Hz, 1 H), 2.91 (ddd, J_1 = 14.8 Hz, J_2 = 6.9 Hz, J_3 = 1.6 Hz, 1 H), 3.65 (s, 3 H), 3.92 (dd, J_1 = 6.9 Hz, J_2 = 2.8 Hz, 1 H), 5.15 (d, J = 1.6 Hz, 1 H), 7.06-7.14 (m, 3 H), 7.14-7.23 (m, 3 H), 7.25-7.35 (m, 3 H)

^{13}C-NMR (75 MHz, CDCl3, 298 K): δ / ppm = 33.1, 35.8, 51.5, 103.4, 111.1, 118.8, 122.1, 127.3 (2 C), 127.4, 128.9 (2 C), 130.6, 132.5, 132.7, 136.6, 141.2, 159.3, 164.1, 170.5, 191.4

HRMS (ESI): m/z calc. for $C_{21}H_{16}O_4$: 333.1122 [M+H]+; found: 333.1126

$[\alpha]_D^{20}$= -139.8 (*c* 1.0, $CHCl_3$, 95:5 *e.r.*)

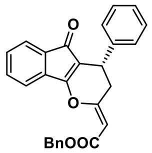

Benzyl (S)-6-oxo-5-phenyl-5,6-dihydro-4H-indeno [1,2-b] oxepine-2-carboxylate (**3c**) was synthesised according to general procedure D. The synthesis was carried out on a scale of 0.1 mmol and gave the product in 76% and 96:4 er using **ITU5** and 84% and 97:3 er using **ISeU**.

HPLC (YMC-SA, n-hexane/IPA = 4/1, flow = 0.5 mL min-1, T_{Column} = 10 °C, *l* = 220 nm): t_R: 21.2 min (minor), 30.1 min (major).

^{1}H-NMR (300 MHz, $CDCl_3$, 298 K): δ / ppm = 2.58 (dd, J_1 = 15.0 Hz, J_2 = 2.9 Hz, 1 H), 2.92 (ddd, J_1 = 15.0 Hz, J_2 = 6.9 Hz, J_3 = 1.6 Hz, 1 Hz), 3.93 (dd, J_1 = 6.9 Hz, J_2 = 2.9 Hz, 1 H), 5.12 (s, 2 H), 5.21 (d, J_1 = 1.6 Hz, 1 H), 6.97-7.02 (m, 1 H), 7.09-7.23 (m, 7 H), 7.25-7.35 (m, 6 H)

^{13}C-NMR (75 MHz, $CDCl_3$, 298 K): δ / ppm =, 33.0, 35.7, 66.4, 103.5, 111.0, 118.8, 122.0, 127.3 (2 C), 127.4, 128.4, 128.6 (2 C), 128.7 (2 C), 128.9 (2 C), 130.5, 132.4, 132.6, 136.0, 136.5, 141.1, 159.4, 163.6, 170.4, 191.4

HRMS (ESI): m/z calc. for $C_{27}H_{20}O_4$: 409.1435 $[M+H]^+$; found: 409.1443

$[\alpha]_D^{20}$= -129.9 (*c* 1.0, $CHCl_3$, 97:3 *e.r.*)

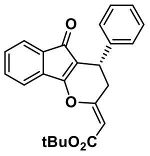

Tert. butyl (S)-6-oxo-5-phenyl-5,6-dihydro-4H-indeno [1,2-b] oxepine-2-carboxylate (**3d**) was synthesised according to general procedure D. The synthesis was carried out on a scale of 0.1 mmol and gave the product in 64% and 93:7 er using **ITU5** and 93% and 95:5 er using **ISeU**.

HPLC (YMC-SA, n-hexane/IPA = 4/1, flow = 0.5 mL min-1, T_{Column} = 10 °C, *l* = 220 nm):

t_R: 10.5 min (minor), 14.5 min (major).

^{1}H-NMR (300 MHz, $CDCl_3$, 298 K): δ / ppm = 1.44 (s, 9 H), 2.54 (dd, J_1 = 14.8 Hz, J_2 = 3.0 Hz, 1 H), 2.89 (ddd, J_1 = 14.8 Hz, J_2 = 6.8 Hz, J_3 = 1.6 Hz, 1 H), 3.92 (dd, J_1 = 6.8 Hz, J_2 = 3 Hz, 1 H), 5.08 (d, J = 1.6 Hz, 1 H), 7.08-7.16 (m, 3 H), 7.16-7.25 (m, 3 H), 7.25-7.35 (m, 3 H)

^{13}C-NMR (125 MHz, $CDCl_3$, 298 K): δ / ppm = 28.4 (3 C), 33.2, 35.7, 80.8, 105.7, 111.0, 118.8, 122.0, 127.3, 127.4 (2 C), 128.9 (2 C), 130.5, 132.6 (2 C), 136.7, 141.3, 157.7, 163.1, 170.7, 191.5

HRMS (ESI): m/z calc. for $C_{24}H_{22}O_4$: 375.1591 $[M+H]^+$; found: 375.1601

$[\alpha]_D^{20}$= -161.4 (*c* 1.0, $CHCl_3$, 95:5 *e.r.*)

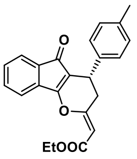

Ethyl (S,Z)-2-(5-oxo-4-(p-tolyl)-4,5-dihydroindeno[1,2-b]pyran-2(3H)-ylidene) acetate (**3e**) was synthesised according to general procedure D. The synthesis was carried out on a scale of 0.1 mmol and gave the product in 67% and 95:5 er using **ITU5** and 70% and 96:4 er using **ISeU**.

HPLC (YMC-SA, n-hexane/IPA = 4/1, flow = 0.5 mL min-1, T_{Column} = 10 °C, *l* = 220 nm):

t_R: 14.03 min (minor), 20.50 min (major)

^{1}H-NMR (300 MHz, $CDCl_3$, 298 K): δ / ppm = 1.33 (t, *J* = 7.1 Hz, 3 H), 2.30 (s, 3 H), 2.64 (dd, J_1 = 14.8 Hz, J_2 = 2.9 Hz, 1 H), 3.00 (ddd, J_1 = 14.8 Hz, J_2 = 6.8 Hz, J_3 = 1.6 Hz, 1 H), 3.99 (dd, J_1 = 6.8 Hz, J_2 = 2.9 Hz, 1 H), 4.23 (q, *J* = 7.1 Hz, 2 H), 5.25 (d, *J* = 1.6 Hz, 1 H), 7.10 (s, 4 H), 7.28-7.43 (m, 4 H)

^{13}C-NMR (75 MHz, $CDCl_3$, 298 K): δ / ppm = 14.4, 21.1, 32.7, 35.9, 60.3, 103.8, 111.3, 118.7, 122.0, 127.2 (2 C), 129.6, 130.5 (2 C), 132.5, 132.6, 136.7, 136.9, 138.2, 159.1, 163.8, 170.4, 191.4

HRMS (ESI): m/z calc. for $C_{23}H_{20}O_4$: 361.1435 $[M+H]^+$; found: 361.1443

$[\alpha]_D^{20}$= -198.0 (*c* 1.0, $CHCl_3$, 96:4 *e.r.*)

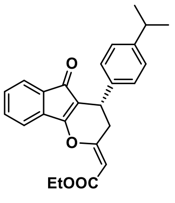

Ethyl (S,Z)-2-(4-(4-isopropylphenyl)-5-oxo-4,5-dihydroindeno[1,2-b]pyran-2(3H)-ylidene)acetate (**3f**) was synthesised according to general procedure D. The synthesis was carried out on a scale of 0.1 mmol and gave the product in 59% and 95:5 er using **ITU5** and 51% and 96:4 er using **ISeU**.

HPLC (YMC-SA, n-hexane/IPA = 4/1, flow = 0.5 mL min-1, T_{Column} = 10 °C, *l* = 220 nm):

t_R: 11.2 min (minor), 15.7 min (major).

^{1}H-NMR (300 MHz, $CDCl_3$, 298 K): δ / ppm = 1.20 (d, *J* = 6.9 Hz, 6 H), 1.34 (t, *J* = 7.1 Hz, 3 H), 2.68 (dd, J_1 = 14.9 Hz, J_2 = 2.8 Hz, 1 H), 2.86 (sep, *J* = 6.9 Hz, 1 H), 3.01 (ddd, J_1 = 14.9 Hz, J_2 = 6.8 Hz, J_3 = 1.6 Hz, 1 H), 4.00 (dd, J_1 = 6.8 Hz, J_2 = 2.8 Hz, 1 H), 4.23 (q, *J* = 7.1 Hz, 2 H), 5.27 (d, *J* = 1.6 Hz, 1 H), 7.14 (s, 4 H), 7.27-7.43 (m, 4 H)

^{13}C-NMR (75 MHz, $CDCl_3$, 298 K): δ / ppm = 14.5, 24.0, 32.7, 33.8, 35.6, 60.4, 103.8, 111.4, 118.7, 122.0, 126.9 (2 C), 127.2 (2 C), 130.5, 132.5, 132.6, 136.7, 138.5, 147.8, 159.2, 163.9, 170.3, 191.5

HRMS (ESI): m/z calc. for $C_{25}H_{24}O_4$: 389.1748 $[M+H]^+$; found: 389.1754

$[\alpha]_D^{20}$ = -165.2 (*c* 1.0, $CHCl_3$, 96:4 *e.r.*)

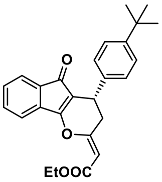

Ethyl (S,Z)-2-(4-(4-(tert-butyl)phenyl)-5-oxo-4,5-dihydroindeno[1,2-b]pyran-2(3H)-ylidene)acetate (**3g**) was synthesised according to general procedure D. The synthesis was carried out on a scale of 0.1 mmol and gave the product in 70% and 94:6 er using **ITU5** and 72% and 95:5 er using **ISeU**.

HPLC (YMC-SA, n-hexane/IPA = 4/1, flow = 0.5 mL min-1, T_{Column} = 10 °C, *l* = 220 nm):

t_R: 10.7 min (minor), 14.8 min (major).

^{1}H-NMR (300 MHz, $CDCl_3$, 298 K): δ / ppm = 1.28 (s, 9 H), 1.34 (t, *J* = 7.1 Hz, 3 H), 2.69 (dd, J_1 = 14.9 Hz, J_2 = 2.7 Hz, 1 H), 3.01 (ddd, J_1 = 14.9 Hz, J_2 = 6.8 Hz, J_3 = 1.6 Hz, 1 H), 4.00 (dd, J_1 = 6.8 Hz, J_2 = 2.7 Hz, 1 H), 4.24 (q, *J* = 7.1 Hz, 2 H), 5.28 (d, *J* = 1.6 Hz, 1 H), 7.12-7.18 (m, 2 H), 7.27-7.44 (m, 6 H)

^{13}C-NMR (75 MHz, $CDCl_3$, 298 K): δ / ppm = 14.5, 31.4, 32.5, 34.5, 35.5, 60.3, 103.7, 111.4, 118.7, 122.0, 125.8 (2 C), 126.9 (2 C), 130.5, 132.5, 132.6, 136.7, 138.1, 150.0, 159.2, 163.9, 170.2, 191.5

HRMS (ESI): m/z calc. for $C_{26}H_{26}O_4$: 403.1904 $[M+H]^+$; found: 403.1905

$[\alpha]_D^{20}$ = -198.3 (*c* 1.0, $CHCl_3$, 95:5 *e.r.*)

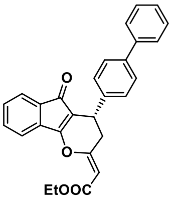

Ethyl (S,Z)-2-(4-([1,1'-biphenyl]-4-yl)-5-oxo-4,5-dihydroindeno[1,2-b]pyran-2(3H)-ylidene)acetate (**3h**) was synthesised according to general procedure D. The synthesis was carried out on a scale of 0.1 mmol and gave the product in 75% and 92:8 er using **ITU5** and 51% and 96:4 er using **ISeU**.

HPLC (YMC-SA, n-hexane/IPA = 4/1, flow = 0.5 mL min-1, T_{Column} = 10 °C, *l* = 220 nm):

t_R: 20.8 min (minor), 30.5 min (major).

^{1}H-NMR (300 MHz, $CDCl_3$, 298 K): δ / ppm = 1.34 (t, *J* = 7.1 Hz, 3 H), 2.71 (dd, J_1 = 14.9 Hz, J_2 = 2.8 Hz, 1 H), 3.06 (ddd, J_1 = 14.9 Hz, J_2 = 6.8 Hz, J_3 = 1.5 Hz, 1 H), 4.08 (dd, J_1 = 6.8 Hz, J_2 = 2.8 Hz, 1 H), 4.24 (q, *J* = 7.1 Hz, 2 H), 5.30 (d, *J* = 1.5 Hz, 1 H), 7.27-7.36 (m, 4 H), 7.37-7.47 (m, 5 H), 7.50-7.58 (m, 4 H)

^{13}C-NMR (75 MHz, CDCl$_3$, 298 K): δ / ppm = 14.4, 32.8, 35.7, 60.4, 104.0, 111.0, 118.8, 122.1, 127.2 (2 C), 127.4, 127.6 (2 C), 127.7 (2 C), 128.8 (2 C), 130.6, 132.5, 132.7, 136.6, 140.2, 140.3, 140.8, 158.9, 163.8, 170.6, 191.4

HRMS (ESI): m/z calc. for $C_{28}H_{22}O_4$: 423.1591 [M+H]$^+$; found: 423.1597

$[\alpha]_D^{20}$= -250.9 (*c* 1.0, CHCl$_3$, 96:4 *e.r.*)

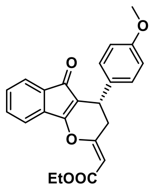

Ethyl (S,Z)-2-(4-(4-methoxyphenyl)-5-oxo-4,5-dihydroindeno[1,2-b]pyran-2(3H)-ylidene)acetate (**3i**) was synthesised according to general procedure D. The synthesis was carried out on a scale of 0.1 mmol and gave the product in 76% and 95:5 er using **ITU5** and 67% and 96:4 er using **ISeU**.

HPLC (YMC-SA, n-hexane/IPA = 4/1, flow = 0.5 mL min-1, T_{Column} = 10 °C, *l* = 220 nm):

t_R: 21.83 min (minor), 30.81 min (major).

^{1}H-NMR (300 MHz, CDCl$_3$, 298 K): δ / ppm = 1.33 (t, *J* = 7.1 Hz, 3 H), 2.63 (dd, J_1 = 14.8 Hz, J_2 = 2.6 Hz, 1 H), 3.00 (ddd, J_1 = 14.8 Hz, J_2 = 6.6 Hz, J_3 = 1.1 Hz, 1 H), 3.76 (s, 3 H), 3.98 (dd, J_1 = 6.6 Hz, J_2 = 2.6 Hz, 1 H), 4.22 (q, *J* = 7.1 Hz, 2 H), 5.25 (d, *J* = 1.1 Hz, 1 H), 6.77-6.88 (m, 2 H), 7.08-7.18 (m, 2 H), 7.27-7.45 (m, 4 H)

^{13}C-NMR (75 MHz, CDCl$_3$, 298 K): δ / ppm = 14.4, 32.3, 36.0, 55.4, 60.3, 103.8, 111.4, 114.3 (2 C), 118.7, 122.0, 128.3 (2 C), 130.5, 132.5, 132.6, 133.3, 136.7, 158.8, 159.1, 163.8, 170.4, 191.5

HRMS (ESI): m/z calc. for $C_{23}H_{20}O_5$: 377.1384 [M+H]$^+$; found: 377.1390

$[\alpha]_D^{20}$= -194.1 (*c* 1.0, CHCl$_3$, 96:4 *e.r.*)

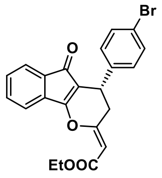

Ethyl (S,Z)-2-(4-(4-bromophenyl)-5-oxo-4,5-dihydroindeno[1,2-b]pyran-2(3H)-ylidene)acetate (**3j**) was synthesised according to general procedure D. The synthesis was carried out on a scale of 0.1 mmol and gave the product in 75% and 90:10 er using **ITU5** and 71% and 92:8 er using **ISeU**.

HPLC (YMC-SA, n-hexane/IPA = 4/1, flow = 0.5 mL min-1, T_{Column} = 10 °C, *l* = 220 nm):

t_R: 17.4 min (minor), 31.3 min (major).

^{1}H-NMR (300 MHz, CDCl$_3$, 298 K): δ / ppm = 1.32 (t, *J* = 7.1 Hz, 3 H), 2.61 (dd, J_1 = 14.9 Hz, J_2 = 2.9 Hz, 1 H), 3.01 (ddd, J_1 = 14.9 Hz, J_2 = 6.8 Hz, J_3 = 1.6 Hz, 1 H), 3.99 (dd, J_1 = 6.8 Hz, J_2 = 2.9 Hz, 1 H), 4.22 (q, *J* = 7.1 Hz, 2 H), 5.25 (d, *J* = 1.6 Hz, 1 H), 7.05-7.13 (m, 2 H), 7.28-7.46 (m, 6 H)

^{13}C-NMR (75 MHz, CDCl$_3$, 298 K): δ / ppm = 14.4, 32.7, 35.6, 60.4, 104.2, 110.4, 118.9, 121.3, 122.1, 129.1 (2 C), 130.7, 132.0 (2 C), 132.4, 132.7, 136.4, 140.2, 158.4, 163.6, 170.8, 191.2

HRMS (ESI): m/z calc. for $C_{22}H_{17}BrO_4$: 425.0383 [M+H]$^+$; found: 425.0389

$[\alpha]_D^{20}$= -191.5 (*c* 1.0, CHCl$_3$, 92:8 *e.r.*)

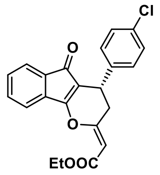

Ethyl (S,Z)-2-(4-(4-chlorophenyl)-5-oxo-4,5-dihydroindeno [1,2-b]pyran-2(3H)-ylidene)acetate (**3k**) was synthesised according to general procedure D. The synthesis was carried out on a scale of 0.1 mmol and gave the product in 80% and 91:9 er using **ITU5** and 72% and 93:7 er using **ISeU**.

HPLC (YMC-SA, n-hexane/IPA = 4/1, flow = 0.5 mL min-1, T_{Column} = 10 °C, *l* = 220 nm):

t_R: 16.7 min (minor), 29.3 min (major).

^{1}H-NMR (300 MHz, $CDCl_3$, 298 K): δ / ppm = 1.33 (t, *J* = 7.1 Hz, 3 H),
2.61 (dd, J_1 = 14.8 Hz, J_2 = 2.9 Hz, 1 H), 3.01 (ddd, J_1 = 14.8 Hz, J_2 = 6.8 Hz, J_3 = 1.6 Hz, 1 H),
4.00 (dd, J_1 = 6.7 Hz, J_2 = 2.9 Hz, 1 H), 4.22 (q, *J* = 7.1 Hz, 2 H), 5.25 (d, *J* = 1.6 Hz, 1 H),
7.10-7.19 (m, 2 H), 7.23-7.46 (m, 6 H)

^{13}C-NMR (75 MHz, $CDCl_3$, 298 K): δ / ppm = 14.4, 32.6, 35.7, 60.4, 104.2, 110.5, 118.9, 122.1,
128.7 (2 C), 129.0 (2 C), 130.7, 132.4, 132.7, 133.2, 136.5, 139.7, 158.5, 163.6, 170.7, 191.3

HRMS (ESI): m/z calc. for $C_{22}H_{17}ClO_4$: 381.0888 $[M+H]^+$; found: 381.0893

$[\alpha]_D^{20}$= -172.3 (*c* 1.0, $CHCl_3$, 93:7 *e.r.*)

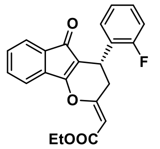

Ethyl (R,Z)-2-(4-(2-fluorophenyl)-5-oxo-4,5-dihydroindeno[1,2-b]pyran-2(3H)-ylidene)acetate (**3l**) was synthesised according to general procedure D. The synthesis was carried out on a scale of 0.1 mmol and gave the product in 81% and 90:10 er using **ITU5** and 79% and 93:7 er using **ISeU**.

HPLC (YMC-SZ, n-hexane/IPA = 10/1, flow = 0.5 mL min-1, T_{Column} = 10 °C, *l* = 220 nm): t_R: min (minor), min (major).

^{1}H-NMR (700 MHz, $CDCl_3$, 298 K): δ / ppm = 1.31 (t, *J* = 7.1 Hz, 3 H),
2.67 (dd, J_1 = 14.8 Hz, J_2 = 3.2 Hz, 1 H), 3.01 (ddd, J_1 = 14.8 Hz, J_2 = 6.7 Hz, J_3 = 1.4 Hz, 1 H),
4.20 (q, *J* = 7.1 Hz, 2 H), 4.36 (dd, J_1 = 6.7 Hz, J_2 = 3.2 Hz, 1 H), 5.19 (d, *J* = 1.4 Hz, 1 H),
7.00-7.08 (m, 3 H), 7.19-7.24 (m, 1 H), 7.31-7.35 (m, 1 H), 7.38-7.43 (m, 2 H), 7.42-7.46 (m, 1 H)

^{19}F-NMR (282 MHz, $CDCl_3$ 298 K): -118.80

^{13}C-NMR (175 MHz, $CDCl_3$, 298 K): δ / ppm = 14.4, 26.9 (d, *J* = 3.3 Hz), 34.9, 60.4, 104.1, 109.5,
115.8 (d, *J* = 21.7 Hz), 118.8, 122.1, 124.4 (d, *J* = 3.3 Hz), 127.7 (d, *J* = 13.8 Hz), 129.0 (d, *J* = 3.7 Hz),
129.1 (d, *J* = 8.2 Hz), 130.7, 132.5, 132.7, 136.5, 158.6, 159.7, 161.1, 163.6, 171.7

HRMS (ESI-TOF): m/z calc. for $C_{22}H_{17}FO_4$: $[M+H]^+$; found:

$[\alpha]_D^{20}$= -240.3 (*c* 1.0, $CHCl_3$, *e.r.*)

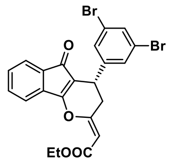

Ethyl (S,Z)-2-(4-(3,5-dibromophenyl)-5-oxo-4,5-dihydroindeno[1,2-b]pyran-2(3H)-ylidene)acetate (**3m**) was synthesised according to general procedure D. The synthesis was carried out on a scale of 0.1 mmol and gave the product in 62% and 77:23 er using **ITU5** and 63% and 84:16 er using **ISeU**.

HPLC (YMC-SA, n-hexane/IPA = 4/1, flow = 0.5 mL min-1, T_{Column} = 10 °C, *l* = 220 nm):

t_R: 15.1 min (minor), 23.9 min (major).

^{1}H-NMR (300 MHz, $CDCl_3$, 298 K): δ / ppm = 1.33 (t, J = 7.1 Hz, 3 H),
2.61 (dd, J_1 = 14.9 Hz, J_2 = 3.2 Hz, 1 H), 3.08 (ddd, J_1 = 14.9 Hz, J_2 = 6.8 Hz, J_3 = 1.5 Hz, 1 H),
3.96 (dd, J_1 = 6.8 Hz, J_2 = 3.0 Hz, 1 H), 4.23 (q, J = 7.1 Hz, 2 H), 5.25 (d, J = 1.5 Hz, 1 H),
7.27 (d, J = 1.7 Hz, 2 H), 7.31-7.47 (m, 4 H), 7.54 (t, J = 1.7 Hz, 1 H)

^{13}C-NMR (75 MHz, $CDCl_3$, 298 K): δ / ppm = 14.4, 32.7, 35.5, 60.5, 104.7, 109.3, 119.1, 122.3,
123.4 (2 C), 129.3 (2 C), 130.9, 132.4, 132.8, 133.3, 136.3, 145.1, 157.6, 163.4, 171.3, 191.0

HRMS (ESI-TOF): m/z calc. for $C_{22}H_{16}Br_2O_4$: 504.9464 $[M+H]^+$; found: 504.9468

$[\alpha]_D^{20}$= -121.5 (*c* 1.0, $CHCl_3$, 84:16 *e.r.*)

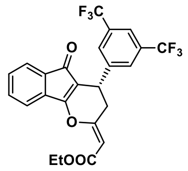

Ethyl (S,Z)-2-(4-(3,5-bis(trifluoromethyl)phenyl)-5-oxo-4,5-dihydroindeno[1,2-b]pyran-2(3H)-ylidene)acetate (**3n**) was synthesised according to general procedure D. The synthesis was carried out on a scale of 0.1 mmol and gave the product in 44% and 69:31 er using **ITU5** and 58% and 81:19 er using **ISeU**.

HPLC (YMC-SA, n-hexane/IPA = 4/1, flow = 0.5 mL min-1, T_{Column} = 10 °C, *l* = 220 nm):

t_R: 10.11 min (minor), 13.14 min (major).

^{1}H-NMR (300 MHz, $CDCl_3$, 298 K): δ / ppm = 1.33 (t, J = 7.1 Hz, 3 H),
2.65 (dd, J_1 = 14.8 Hz, J_2 = 3.6 Hz, 1 H), 3.07 (ddd, J_1 = 14.8 Hz, J_2 = 6.6 Hz, J_3 = 1.3 Hz, 1 H),
4.17 (dd, J_1 = 6.6 Hz, J_2 = 3.6 Hz, 1 H), 4.24 (q, J = 7.1 Hz, 2 H), 5.30 (d, J = 1.3 Hz, 1 H),
7.33-7.48 (m, 4 H), 7.67 (s, 2 H), 7.77 (s, 1 H)

^{19}F-NMR (282 MHz, $CDCl_3$ 298 K): 62.80

^{13}C-NMR (75 MHz, $CDCl_3$, 298 K): δ / ppm = 14.4, 33.3, 35.4, 60.6, 105.1, 108.9, 119.3,
121.7 (m, J = 7.4 Hz, 2 C), 122.4, 127.67 (m, J = 2.8 Hz, 2 C), 131.1, 132.0, 132.3, 132.4, 132.9, 136.2,
143.8, 157.3, 163.3, 171.6, 190.9

HRMS (ESI-TOF): m/z calc. for $C_{24}H_{16}F_6O_4$: 483.1026 $[M+H]^+$; found: 483.1031

$[\alpha]_D^{20}$= -71.8 (*c* 1.0, $CHCl_3$, 81:19 *e.r.*)

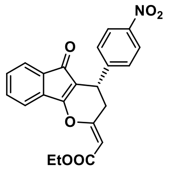

Ethyl (S,Z)-2-(4-(4-nitrophenyl)-5-oxo-4,5-dihydroindeno[1,2-b]pyran-2(3H)-ylidene)acetate (**3o**) was synthesised according to general procedure D. The synthesis was carried out on a scale of 0.1 mmol and gave the product in 67% and 79:21 er using **ITU5** and 56% and 82:18 er using **ISeU**.

HPLC (YMC-SA, n-hexane/IPA = 4/1, flow = 0.5 mL min-1, T_{Column} = 10 °C, *l* = 220 nm):

t_R: 17.47 min (minor), 45.13 min (major).

^{1}H-NMR (300 MHz, $CDCl_3$, 298 K): δ / ppm = 1.33 (t, *J* = 7.1 Hz, 3 H),

2.66 (dd, J_1 = 14.9 Hz, J_2 = 3.0 Hz, 1 H), 3.08 (ddd, J_1 = 14.9 Hz, J_2 = 6.8 Hz, J_3 = 1.2 Hz, 1 H),

4.14 (dd, J_1 = 6.8 Hz, J_2 = 3.0 Hz, 1 H), 4.23 (q, *J* = 7.1 Hz, 2 H), 5.29 (d, *J* = 1.2 Hz, 1 H),

7.29-7.47 (m, 6 H), 8.10-8.23 (m, 2 H)

^{13}C-NMR (75 MHz, $CDCl_3$, 298 K): δ / ppm = 14.4, 33.1, 35.2, 60.6, 104.7, 109.5, 119.1, 122.3, 124.2 (2 C), 128.4 (2 C), 131.0, 132.3, 132.9, 136.2, 147.3, 148.6, 157.7, 163.4, 171.2, 191.0

HRMS (ESI): m/z calc. for $C_{22}H_{17}NO_6$: 392.1129 $[M+H]^+$; found: 392.1136

$[\alpha]_D^{20}$= -162.1 (*c* 1.0, $CHCl_3$, 96:4 *e.r.*)

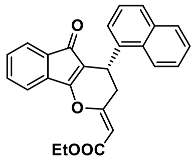

Ethyl (S,Z)-2-(4-(naphthalen-1-yl)-5-oxo-4,5-dihydroindeno[1,2-b]pyran-2(3H)-ylidene)acetate (**3p**) was synthesised according to general procedure D. The synthesis was carried out on a scale of 0.1 mmol and gave the product in 85% and 88:12 er using **ITU5** and 87% and 83:17 er using **ISeU**.

HPLC (YMC-SA, n-hexane/IPA = 4/1, flow = 0.5 mL min-1, T_{Column} = 10 °C, *l* = 220 nm):

t_R: 18.3 min (minor), 23.8 min (major).

^{1}H-NMR (300 MHz, $CDCl_3$, 298 K): δ / ppm = 1.28 (t, *J* = 7.1 Hz, 3 H),

2.77 (dd, J_1 = 14.6 Hz, J_2 = 2.1 Hz, 1 H), 3.17 (ddd, J_1 = 14.6 Hz, J_2 = 6.9 Hz, J_3 = 1.6 Hz, 1 H),

4.17 (q, *J* = 7.1 Hz, 2 H), 4.87 (dd, J_1 = 6.9 Hz, J_2 = 2.1 Hz, 1 H), 5.01 (d, *J* = 1.5 Hz, 1 H),

7.15-7.21 (m, 1 H), 7.30-7.39 (m, 2 H), 7.42-7.62 (m, 5 7.75 (d, *J* = 8.2 Hz, 1 H),7.87-7.93 (m, 1 H), 8.11 (d, *J* = 8.2 Hz, 1 H)

^{13}C-NMR (75 MHz, $CDCl_3$, 298 K): δ / ppm = 14.4, 29.2, 35.4, 60.3, 104.4, 110.5, 118.7, 122.2, 122.7, 124.9, 125.5, 125.8, 126.6, 128.2, 129.4, 130.6, 130.6, 132.7, 132.7, 134.4, 136.0, 136.7, 158.7, 163.6, 171.8, 191.4

HRMS (ESI-TOF): m/z calc. for $C_{26}H_{20}O_4$: 397.1435 $[M+H]^+$; found: 397.1441

$[\alpha]_D^{20}$= -277.0 (*c* 1.0, $CHCl_3$, 83:17 *e.r.*)

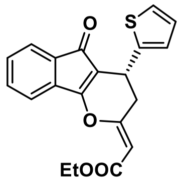

Ethyl (R,Z)-2-(5-oxo-4-(thiophen-2-yl)-4,5-dihydroindeno[1,2-b]pyran-2(3H)-ylidene)acetate (S,Z)-2-(4-(3,5-dibromophenyl)-5-oxo-4,5-dihydroindeno[1,2-b]pyran-2(3H)-ylidene)acetate (**3q**) was synthesised according to general procedure D. The synthesis was carried out on a scale of 0.1 mmol and gave the product in 71% and 91:9 er using **ITU5** and 70% and 94:6 er using **ISeU**.

HPLC (YMC-SA, n-hexane/IPA = 4/1, flow = 0.5 mL min-1, T_{Column} = 10 °C, l = 220 nm):

t_R: 17.97 min (minor), 21.72 min (major).

^{1}H-NMR (700 MHz, $CDCl_3$, 298 K): δ / ppm = 1.34 (t, J = 7.1 Hz, 3 H),
2.78 (dd, J_1 = 14.8 Hz, J_2 = 2.1 Hz, 1 H), 3.03 (ddd, J_1 = 14.8 Hz, J_2 = 6.4 Hz, J_3 = 1.1 Hz, 1 H),
4.22 (q, J = 7.1 Hz, 2 H), 4.31 (dd, J_1 = 6.4 Hz, J_2 = 2.0 Hz, 1 H), 5.33 (d, J = 1.1 Hz, 1 H),
6.86-6.92 (m, 2 H), 7.13-7.16 (m, 1 H), 7.30-7.37 (m, 2 H), 7.37-7.40 (m, 1 H), 7.43-7.46 (m, 1 H)

^{13}C-NMR (175 MHz, $CDCl_3$, 298 K): δ / ppm = 14.4, 28.4, 36.0, 60.4, 104.7, 111.0, 119.0, 122.2, 124.5, 124.8, 127.1, 130.7, 132.4, 132.7, 136.4, 144.5, 158.35, 163.6, 170.2, 191.1

HRMS (ESI-TOF): m/z calc. for $C_{20}H_{16}O_4S$: 353.0842 $[M+H]^+$; found: 353.0848

$[\alpha]_D^{20}$= -277.2 (*c* 0.2, $CHCl_3$, 94:6 *e.r.*)

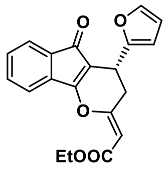

Ethyl (R,Z)-2-(4-(furan-2-yl)-5-oxo-4,5-dihydroindeno[1,2-b]pyran-2(3H)-ylidene)acetate (**3r**) was synthesised according to general procedure D. The synthesis was carried out on a scale of 0.1 mmol and gave the product in 76% and 86:14 er using **ITU5** and 59% and 90:10 er using **ISeU**.

HPLC (Chiracel-ODH, n-hexane/IPA = 4/1, flow = 0.5 mL min-1, T_{Column} = 10 °C, l = 220 nm):

t_R: 28.6 min (minor), 36.4 min (major).

^{1}H-NMR (700 MHz, $CDCl_3$, 298 K): δ / ppm = 1.32 (t, J = 7.1 Hz, 3 H), 2.85-2.93 (m, 2 H),
4.10 (dd, J_1 = 5.8 Hz, J_2 = 3.2 Hz, 1 H), 4.21 (q, J = 7.1 Hz, 2 H), 5.31 (d, J = 0.8 Hz, 1 H),
6.05 (d, J = 3.1 Hz, 1 H), 6.25 (dd, J_1 = 3.1 Hz, J = 1.8 Hz, 1 H), 7.30-7.35 (m, 3 H), 7.37-7.40 (m,1 H),
7.44-7.46 (m, 1 H)

^{13}C-NMR (175 MHz, $CDCl_3$, 298 K): δ / ppm = 14.4, 27.1, 32.85, 60.38, 104.1, 106.7, 108.9, 110.5, 118.9, 122.1, 130.7, 132.5, 132.6, 136.5, 142.2, 153.1, 158.7, 163.7, 170.9, 191.1

HRMS (ESI-TOF): m/z calc. for $C_{20}H_{16}O_5$: 337.1071 $[M+H]^+$; found: 337.1073

$[\alpha]_D^{20}$= -195.6 (*c* 0.2, $CHCl_3$, 90:10 *e.r.*)

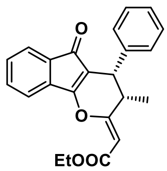

Ethyl (Z)-2-((3S,4S)-3-methyl-5-oxo-4-phenyl-4,5-dihydroindeno[1,2-b] pyran-2(3H)-ylidene)acetate (**3s**$_{\mathbf{major}}$) was synthesised according to general procedure D. The synthesis was carried out on a scale of 0.1 mmol and gave the product in 49% and 99.5:0.5 er using **ITU5** and 57% and 99.5:0.5 er using **ISeU**.

HPLC (YMC-SA, n-hexane/IPA = 4/1, flow = 0.5 mL min-1, T_{Column} = 10 °C, *l* = 220 nm):

t_R: 11.8 min (minor), 19.6 min (major).

^{1}H-NMR (300 MHz, $CDCl_3$, 298 K): δ / ppm = 0.99 (d, *J* = 6.8 Hz, 3 H), 1.35 (t, *J* = 7.1 Hz, 3 H), 3.01-3.10 (m, 1 H), 3.84 (d, *J* = 6.1 , 1 H), 4.25 (q, *J* = 7.1 Hz, 2 H), 5.22 (d, *J* = 1.7 Hz, 1 H), 7.09-7.12 (m, 2 H), 7.19-7.33 (m, 4 H), 7.36-7.41 (m, 3 H)

^{13}C-NMR (75 MHz, $CDCl_3$, 298 K): δ / ppm = 14.5, 14.6, 36.4, 39.4, 60.4, 102.4, 112.3, 118.7, 122.0, 127.5, 128.5 (2 C), 128.7 (2 C), 130.5, 132.6, 132.7, 136.7, 137.7, 163.7, 164.1, 170.0, 191.3

HRMS (ESI): m/z calc. for $C_{23}H_{20}O_4$: 361.1435 $[M+H]^+$; found: 361.1436

$[\alpha]_D^{20}$= -136.7 (*c* 1.0, $CHCl_3$, >99.5:0.5 *e.r.*)

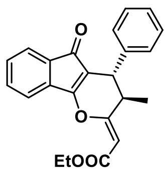

Ethyl (Z)-2-((3R,4S)-3-methyl-5-oxo-4-phenyl-4,5-dihydroindeno[1,2-b] pyran-2(3H)-ylidene)acetate (**3s**$_{\mathbf{minor}}$) was synthesised according to general procedure D. The synthesis was carried out on a scale of 0.1 mmol and gave the product in 21% and 79:21 er using **ITU5** and 13% and 89:11 er using **ISeU**.

HPLC (YMC-SA, n-hexane/IPA = 4/1, flow = 0.5 mL min-1, T_{Column} = 10 °C, *l* = 220 nm):

t_R: 12.23 min (minor), 14.86 min (major).

^{1}H-NMR (300 MHz, $CDCl_3$, 298 K): δ / ppm = 1.33 (t, *J* = 7.1 Hz, 3 H), 1.35 (t, *J* = 7.0 Hz, 3 H), 2.75 (qd, J_1 = 7.0 Hz, J_2 = 2.5 Hz, 1 H), 3.66 (d, *J* = 2.5 , 1 H), 4.22 (q, *J* = 7.1 Hz, 2 H), 5.24 (s, 1 H), 7.16-7.44 (m, 9 H)

^{13}C-NMR (75 MHz, $CDCl_3$, 298 K): δ / ppm = 14.5, 20.3, 40.9, 41.1, 60.4, 103.1, 109.8, 118.8, 122.0, 127.4, 127.4 (2 C), 128.9 (2 C), 130.5, 132.6, 132.7, 136.5, 141.6, 163.3, 164.0, 169.6, 192.1

HRMS (ESI): m/z calc. for $C_{23}H_{20}O_4$: 361.1435 $[M+H]^+$; found: 361.1434

$[\alpha]_D^{20}$= -117.5 (*c* 0.5, $CHCl_3$, 89:11 *e.r.*)

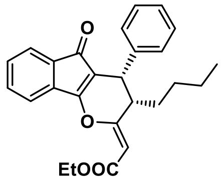

Ethyl (Z)-2-((3S,4S)-3-butyl-5-oxo-4-phenyl-4,5-dihydroindeno[1,2-b]pyran-2(3H)-ylidene)acetate (**3t**$_{major}$) was synthesised according to general procedure D. The synthesis was carried out on a scale of 0.1 mmol. For the experiment with the **ITU5** catalyst only around 10 % conversion could be achieved. Using **ISeU** gave the product in 66 % isolated yield and 99:1 er.

HPLC (YMC-SA, n-hexane/IPA = 4/1, flow = 0.5 mL min-1, T_{Column} = 10 °C, *l* = 220 nm):

t_R: 10.3 min (minor), 13.7 min (major).

^{1}H-NMR (300 MHz, $CDCl_3$, 298 K): *δ* / ppm = 0.88 (t, *J* = 7.2 Hz, 3 H), 1.13-1.51 (m, 6 H), 1.35 (t, *J* = 7.1 , 3 H), 2.75-2.87 (m, 1 H), 3.99 (d, *J* = 5.8 Hz, 1 H), 4.26 (q, *J* = 7.1 Hz, 2 H), 5.25 (d, *J* = 1.5 Hz, 1 H), 7.12-7.40 (m, 9 H),

^{13}C-NMR (75 MHz, $CDCl_3$, 298 K): *δ* / ppm = 14.0, 14.5, 22.6, 27.3, 29.2, 37.2, 41.9, 60.5, 103.0, 112.5, 118.7, 122.0, 127.5, 128.5 (2 C), 128.8 (2 C), 130.5, 132.6, 132.7, 136.6, 137.9, 163.4, 164.1, 170.0, 191.4

HRMS (ESI): m/z calc. for $C_{26}H_{26}O_4$: 403.1904 $[M+H]^+$; found: 403.1901

$[\alpha]_D^{20}$= -87.2 (*c* 0.18, $CHCl_3$, 99:1 *e.r.*)

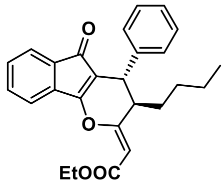

Ethyl (Z)-2-((3R,4S)-3-butyl-5-oxo-4-phenyl-4,5-dihydroindeno[1,2-b]pyran-2(3H)-ylidene)acetate (**3t**$_{minor}$) was synthesised according to general procedure D. The synthesis was carried out on a scale of 0.1 mmol. Using **ISeU** gave the product in 9% isolated yield and 54:46 er.

HPLC (YMC-SA, n-hexane/IPA = 4/1, flow = 0.5 mL min-1, T_{Column} = 10 °C, *l* = 220 nm):

t_R: 9.56 min (minor), 15.84 min (major).

^{1}H-NMR (300 MHz, $CDCl_3$, 298 K): *δ* / ppm = 0.91 (t, *J* = 71 Hz, 3 H), 1.27-1.47 (m, 4 H), 1.33 (t, *J* = 7.1 , 3 H), 1.60-1.72 (m, 2 H), 2.50 (td, J_1 = 7.4 Hz, *J* = 0.8 Hz, 1 H), 3.80 (s, 1 H), 4.22 (q, *J* = 7.1 Hz, 2 H), 5.15 (s, 1 H), 7.16-7.44 (m, 9 H)

^{13}C-NMR (75 MHz, $CDCl_3$, 298 K): *δ* / ppm = 14.0, 14.4, 22.5, 29.7, 33.8, 39.3, 47.2, 60.4, 104.5, 110.0, 118.8, 122.0, 127.3, 127.3 (2 C), 128.9 (2 C), 130.5, 132.6, 132.8, 136.6, 141.9, 161.9, 163.8, 169.8, 192.2

HRMS (ESI): m/z calc. for $C_{26}H_{26}O_4$: 403.1904 $[M+H]^+$; found: 403.1904

$[\alpha]_D^{20}$= -18.9 (*c* 0.18, $CHCl_3$, 54:46 *e.r.*)

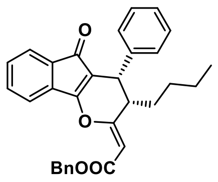

Benzyl (Z)-2-((3S,4S)-3-butyl-5-oxo-4-phenyl-4,5-dihydroindeno[1,2-b] pyran-2(3H)-ylidene)acetate (**3u$_{major}$**) was synthesised according to general procedure D. The synthesis was carried out on a scale of 0.1 mmol and gave the product in 41% and 99:1 er using **ITU5** and 82% and 99:1 er using **ISeU**.

HPLC (YMC-SA, n-hexane/IPA = 4/1, flow = 0.5 mL min-1, T_{Column} = 10 °C, *l* = 220 nm):

t_R: 12.71 min (minor), 19.31 min (major).

^{1}H-NMR (300 MHz, $CDCl_3$, 298 K): δ / ppm = 0.87 (t, *J* = 7.3 Hz, 3 H), 1.14-1.54 (m, 6 H), 2.73-2.90 (m, 1 H), 3.98 (d, *J* = 5.8 Hz, 1 H), 5.24 (s, 2 H), 5.30 (d, J_1 = 1.4 Hz, 1 H), 7.07-7.17 (m, 3 H), 7.17-7.35 (m, 5 H), 7.35-7.47 (m, 6 H)

^{13}C-NMR (125 MHz, $CDCl_3$, 298 K): δ / ppm = 14.0, 22.6, 27.2, 29.2, 37.1, 42.0, 66.5, 102.7, 112.5, 118.8, 122.0, 127.5, 128.5, 128.5 (4 C), 128.6, 128.7 (4 C), 128.8, 130.4, 132.5, 132.6, 136.1, 136.5, 137.8, 163.8, 164.0, 169.9, 191.4

HRMS (ESI): m/z calc. for $C_{31}H_{28}O_4$: 465.2061 $[M+H]^+$; found: 465.2063

$[\alpha]_D^{20}$= -88.3 (*c* 0.83, $CHCl_3$, 99:1 *e.r.*)

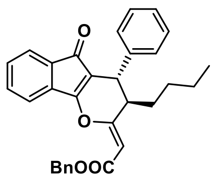

Benzyl (Z)-2-((3R,4S)-3-butyl-5-oxo-4-phenyl-4,5-dihydroindeno[1,2-b]pyran-2(3H)-ylidene)acetate(**3u$_{minor}$**) was synthesised according to general procedure D. The synthesis was carried out on a scale of 0.1 mmol and gave the product in 20% and 60:40 er using **ITU5** and 8% and 80:20 er using **ISeU**.

HPLC (YMC-SA, n-hexane/IPA = 4/1, flow = 0.5 mL min-1, T_{Column} = 10 °C, *l* = 220 nm):

t_R: 12.23 min (minor), 19.55 min (major).

^{1}H-NMR (700 MHz, $CDCl_3$, 298 K): δ / ppm = 0.90 (t, *J* = 7.3 Hz, 3 H), 1.32 (sex, *J* = 7.3 Hz, 2 H), 1.37-1.47 (m, 2 H), 1.64 (q, *J* = 7.7 Hz, 2 H), 2.17 (s, 1, 2.51 (t, *J* = 7.4 Hz, 1 H), 3.79 (s, 1 H), 5.20 (s, 2 H), 7.12 (d, J_1 = 6.7 Hz, 1 H), 7.15-7.24 (m, 3 H), 7.24-7.34 (m, 5 H), 7.34-7.44 (m, 5 H)

^{13}C-NMR (175 MHz, $CDCl_3$, 298 K): δ / ppm = 14.1, 22.5, 29.7, 31.1, 33.8, 39.2, 47.2, 66.4, 104.1, 109.9, 118.9, 122.0, 127.3 (2 C), 128.4, 128.6 (2 C), 128.7 (2 C), 128.9 (2 C), 130.5, 132.6, 132.7, 136.0, 136.4, 141.8, 162.4, 163.7, 169.8, 192.2

HRMS (ESI): m/z calc. for $C_{31}H_{28}O_4$: 465.2061 $[M+H]^+$; found: 465.2076

$[\alpha]_D^{20}$= -55.5 (*c* 0.3, $CHCl_3$, 80:20 *e.r.*)

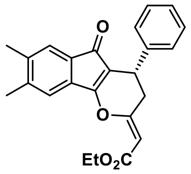

Ethyl (S,Z)-2-(7,8-dimethyl-5-oxo-4-phenyl-4,5-dihydroindeno[1,2 b] pyran -2(3H)-ylidene)acetate (**3v**) was synthesised according to general procedure D. The synthesis was carried out on a scale of 0.1 mmol and gave the product in 84% and 95:5 er using **ITU5** and 83% and 97:3 er using **ISeU**.

HPLC (YMC-SA, n-hexane/IPA = 4/1, flow = 0.5 mL min-1, T_{Column} = 10 °C, *l* = 220 nm):

t_R: 18.3 min (minor), 21.6 min (major).

^{1}H-NMR (300 MHz, $CDCl_3$, 298 K): δ / ppm = 1.32 (t, *J* = 7.1 Hz, 3H), 2.26 (s, 3H), 2.29 (s, 3H), 2.64 (dd, J_1 = 14.8 Hz, J_2 = 2.7 Hz, 1 H), 3.01 (ddd, J_1 = 14.8 Hz, J_2 = 6.7 Hz, J_3 = 1.5 Hz, 1 H), 3.99 (dd, J_1 = 6.7 Hz, J_2 = 2.7 Hz, 1 H), 4.22 (q, *J* = 7.1, 2 H), 5.23 (d, J_1 = 1.5 Hz, 1 H), 7.14 (s, 1 H), 7.17-7.25 (m, 4 H), 7.25-7.32 (m, 2 H)

^{13}C-NMR (75 MHz, $CDCl_3$, 298 K): δ / ppm = 14.4, 20.2, 20.3, 33.1, 35.9, 60.3, 103.5, 110.3, 120.5, 123.9, 127.2, 127.3 (2 C), 128.8 (2 C), 130.5, 134.3, 138.7, 141.3, 141.4, 159.4, 163.8, 170.6, 191.9

HRMS (ESI): m/z calc. for $C_{24}H_{22}O_4$: 375.1591 $[M+H]^+$; found: 375.1592

$[\alpha]_D^{20}$= -224.5 (*c* 1.0, $CHCl_3$, 97:3 *e.r.*)

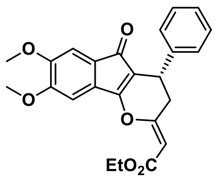

Ethyl (S,Z)-2-(7,8-dimethoxy-5-oxo-4-phenyl-4,5-dihydroindeno[1,2-b]pyran-2(3H)-ylidene)acetate (**3w**) was synthesised according to general procedure D. The synthesis was carried out on a scale of 0.1 mmol. During preparative TLC, decomposition of the product occurred and therefore the yield was determined using the internal NMR standard nitromethane. The product was obtained in 70% (42 % isolated) and 94:6 er using **ITU5** and 80 % (56 % isolated) and 93:7 er using **ISeU**.

HPLC (YMC-SA, n-hexane/IPA = 4/1, flow = 0.5 mL min-1, T_{Column} = 10 °C, *l* = 220 nm):

t_R: 24.51 min (major), 28.82 min (minor).

^{1}H-NMR (300 MHz, $CDCl_3$, 298 K): δ / ppm = 1.33 (t, *J* = 7.1 Hz, 3 H), 2.64 (dd, J_1 = 14.8 Hz, J_2 = 2.8 Hz, 1 H), 3.01 (ddd, J_1 = 14.8 Hz, J_2 = 6.8 Hz, J_3 = 1.5 Hz, 1 H), 3.89 (s, 3 H), 3.95-4.02 (m, 4 H, -CH, $-CH_3$), 4.21 (q, *J* = 7.1 Hz, 2 H), 5.23 (d, J_1 = 1.5 Hz, 1 H), 6.96 (s, 1H), 7.07 (s, 1 H), 7.18-7.25 (m, 3 H), 7.26-7.32 (m, 2 H)

^{13}C-NMR (125 MHz, $CDCl_3$, 298 K): δ / ppm = 14.4, 33.2, 36.0, 56.6, 56.7, 60.3, 103.7 (2 C), 107.2, 109.2, 125.1, 127.3, 127.3 (2 C) 128.9 (2 C), 130.4, 141.5, 150.3, 152.0, 159.4, 163.8, 169.9, 191.3

HRMS (ESI): m/z calc. for $C_{24}H_{22}O_6$: 407.1489 $[M+H]^+$; found: 407.1491

$[\alpha]_D^{20}$= -283.3 (*c* 1.0, $CHCl_3$, 94:6 *e.r.*)

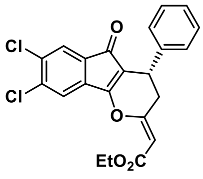

Ethyl (S,Z)-2-(7,8-dichloro-5-oxo-4-phenyl-4,5-dihydroindeno[1,2-b] pyran-2(3H)-ylidene)acetate (**3x**) was synthesised according to general procedure D. The synthesis was carried out on a scale of 0.1 mmol and gave the product in 65% and 77:23 er using **ITU5** and 77% and 86:14 er using **ISeU**.

HPLC (YMC-SA, n-hexane/IPA = 4/1, flow = 0.5 mL min-1, T_{Column} = 10 °C, *l* = 220 nm):

t_R: 14.75 min (minor), 17.66 min (major).

^{1}H-NMR (300 MHz, $CDCl_3$, 298 K): δ / ppm = 1.26 (t, *J* = 7.1 Hz, 3 H),
2.61 (dd, J_1 = 15.0 Hz, J_2 = 2.8 Hz, 1 H), 2.95 (ddd, J_1 = 15.0 Hz, J_2 = 6.8 Hz, J_3 = 1.5 Hz, 1 H),
3.96 (dd, J_1 = 6.8 Hz, J_2 = 2.8 Hz, 1 H), 4.16 (q, *J* = 7.2 Hz, 2 H), 5.22 (d, J_1 = 1.5 Hz, 1 H),
7.08-7.27 (m, 5 H), 7.39 (d, *J* = 5.5 Hz, 2 H)

^{13}C-NMR (75 MHz, $CDCl_3$, 298 K): δ / ppm =14.4, 33.3, 35.4, 60.5, 104.4, 112.8, 120.9, 124.3, 127.2 (2 C), 127.6, 129.0 (2 C), 131.9, 134.6, 135.8, 136.6, 140.7, 158.5, 163.5, 169.1, 188.8

HRMS (ESI): m/z calc. for $C_{22}H_{16}Cl_2O_4$: 415.0499 $[M+H]^+$; found: 415.0500

$[\alpha]_D^{20}$= -83.7 (*c* 1.0, $CHCl_3$, 77:23 *e.r.*)

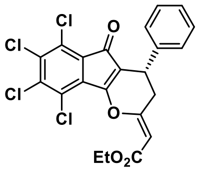

Ethyl (S,Z)-2-(6,7,8,9-tetrachloro-5-oxo-4-phenyl-4,5-dihydroindeno [1,2-b]pyran-2(3H)-ylidene)acetate (**3y**) was synthesised according to general procedure D. The synthesis was carried out on a scale of 0.1 mmol and gave the product in 12% and 63:37 er using **ITU5** and 40% and 75:25 er using **ISeU**.

HPLC (YMC-SA, n-hexane/IPA = 4/1, flow = 0.5 mL min-1, T_{Column} = 10 °C, *l* = 220 nm):

t_R: 19.49 min (minor), 21.64 min (major).

^{1}H-NMR (300 MHz, $CDCl_3$, 298 K): δ / ppm = 1.31 (t, *J* = 7.1 Hz, 3 H),
2.70 (dd, J_1 = 15.0 Hz, J_2 = 2.8 Hz, 1 H), 3.01 (ddd, J_1 = 15.0 Hz, J_2 = 6.7 Hz, J_3 = 1.6 Hz, 1 H),
4.05 (dd, J_1 = 6.7 Hz, *J* = 2.8 Hz, 1 H), 4.24 (q, *J* = 7.1 Hz, 2 H), 5.31 (d, J_1 = 1.6 Hz, 1 H),
7.16-7.34 (m, 5 H)

^{13}C-NMR (75 MHz, $CDCl_3$, 298 K): δ / ppm = 14.5, 33.2, 35.0, 60.7, 105.2, 114.0, 125.5, 127.3 (2 C), 127.4, 127.7, 128.6, 129.1 (2 C), 133.7, 136.1, 138.2, 140.3, 157.5, 163.3, 167.8, 185.5

HRMS (ESI): m/z calc. for $C_{22}H_{14}Cl_4O_4$: 484.9690w $[M+H]^+$; found: 484.9689

$[\alpha]_D^{20}$= -29.3 (*c* 1.0, $CHCl_3$, 75:25 *e.r.*)

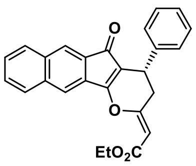

Ethyl (S,Z)-2-(5-oxo-4-phenyl-4,5-dihydrobenzo[5,6]indeno[1,2-b]pyran-2(3H)-ylidene)acetate (**3z**) was synthesised according to general procedure D. The synthesis was carried out on a scale of 0.1 mmol and gave the product in 66% and 87:13 er using **ITU5** and 71% and 91:9 er using **ISeU**.

HPLC (YMC-SA, n-hexane/IPA = 4/1, flow = 0.5 mL min-1, T_{Column} = 10 °C, *l* = 220 nm): t_R: 30.23 min (major), 37.48 min (minor).

^{1}H-NMR (300 MHz, $CDCl_3$, 298 K): δ / ppm = 1.35 (t, *J* = 7.1 Hz, 3 H),
2.71 (dd, J_1 = 14.9 Hz, J_2 = 2.7 Hz, 1 H), 3.07 (ddd, J_1 = 14.9 Hz, J_2 = 6.8 Hz, J_3 = 1.6 Hz, 1 H),
4.12 (dd, J_1 = 6.8 Hz, *J* = 2.7 Hz, 1 H), 4.26 (q, *J* = 7.1 Hz, 2 H), 5.29 (d, J_1 = 1.6 Hz, 1 H),
7.20-7.32 (m, 5 H), 7.46-7.56 (m, 2 H), 7.72 (s, 1 H), 7.78-7.91 (m, 3 H)

^{13}C-NMR (75 MHz, $CDCl_3$, 298 K): δ / ppm = 14.4, 33.3, 35.6, 60.3, 103.7, 116.2, 118.6, 123.3, 127.4 (2 C), 127.4, 127.7, 128.8, 128.9 (2 C), 129.5, 130.8, 131.2, 131.7, 134.4, 135.4, 141.0, 159.2, 163.8, 170.1, 189.9

HRMS (ESI): m/z calc. for $C_{26}H_{20}O_4$: 397.1435 $[M+H]^+$; found: 397.1437

$[\alpha]_D^{20}$= -20.8 (*c* 1.0, $CHCl_3$, 91:9 *e.r.*)

3.3. Robustness Screen

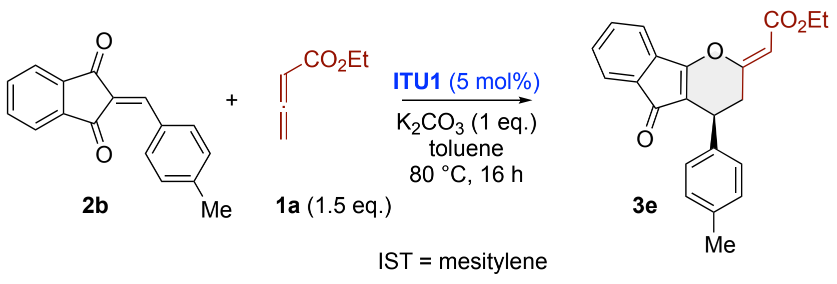

no additive: 65% yield (IST), 0% remaining **2b**
Isobutanol (1 eq.): 63% yield (IST), 0% remaining **2b**
N-Benzylbenzamide (1 eq.): 59% yield (IST), 0% remaining **2b**
Aniline (1 eq.): 54% yield (IST), 0% remaining **2b**
Benzoic acid (1 eq.): 12% yield (IST), 70% remaining **2b**
Phenol (1 eq.): 25% yield (IST), 20% remaining **2b**
Benzaldehyde (1 eq.): 37% yield (IST), 30% remaining **2b**

4. Further Transformations

4.1. Hydrolysis of tert. butyl (S)-6-oxo-5-phenyl-5,6-dihydro-4H-indeno [1,2-b] oxepine-2-carboxylate

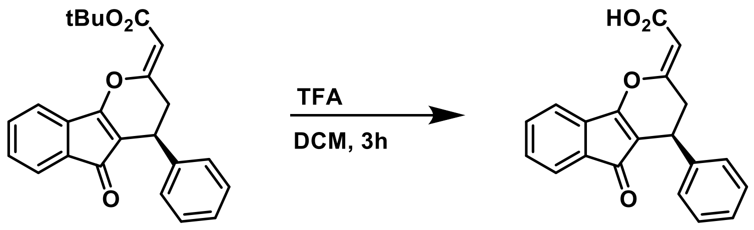

(S,Z)-2-(5-oxo-4-phenyl-4,5-dihydroindeno[1,2-b]pyran-2(3H)-ylidene)acetic acid **7** was prepared using a reported literature procedure [20].First, tert. butyl ester **rac-3d** (28.1 mg, 0.75 mmol) was dissolved in DCM (18 mL/mmol **rac-3d**). Afterwards, trifluoro acetic acid (11.7- mL/mmol **3d**) was added dropwise. The mixture was then stirred at room temperature for 3 h. Subsequently, 15 mL of water was added and extracted three times with DCM. The organic phase is dried with Na_2SO_4 and evaporated to afford the crude product. The crude was afterwards dissolved in DCM and extracted four times with $NaHCO_3$(sat.). The aqueous phase was then acidified with 1 M HCl and extracted three times with DCM. The organic phase was again dried and evaporated to afford the clean product as a yellow waxy solid (23.5 mg, 0.74 mmol, 98 %).

^{1}H-NMR (300 MHz, $CDCl_3$, 298 K): δ / ppm = 2.72 (dd, J_1 = 14.9 Hz, J_2 = 2.7 Hz, 1 H),
3.06 (ddd, J_1 = 14.9 Hz, J_2 = 6.8 Hz, J_3 = 1.6 Hz, 1 H), 4.06 (dd, J_1 = 6.8 Hz, J_2 = 2.7 Hz, 1 H),
5.28 (d, J = 1.4 Hz, 1 H), 7.20-7.45 (m, 9 H)

^{13}C-NMR (75 MHz, $CDCl_3$, 298 K): δ / ppm = 33.0, 35.9, 103.1, 111.3, 118.9, 122.2, 127.3 (2 C), 127.5, 129.0 (2 C), 130.7, 132.4, 132.7, 136.5, 141.0, 161.1, 168.7, 170.3, 191.4

HRMS (ESI-TOF): m/z calc. for $C_{20}H_{14}O_4$: 319.0965 $[M+H]^+$; found: 319.0965

4.2. Hydrogenations of ethyl (S,Z)-2-(5-oxo-4-phenyl-4,5-dihydroindeno[1,2-b]pyran-2(3H)-ylidene)acetate

4.2.1. Hydrogenation utilizing $LiBH_4$

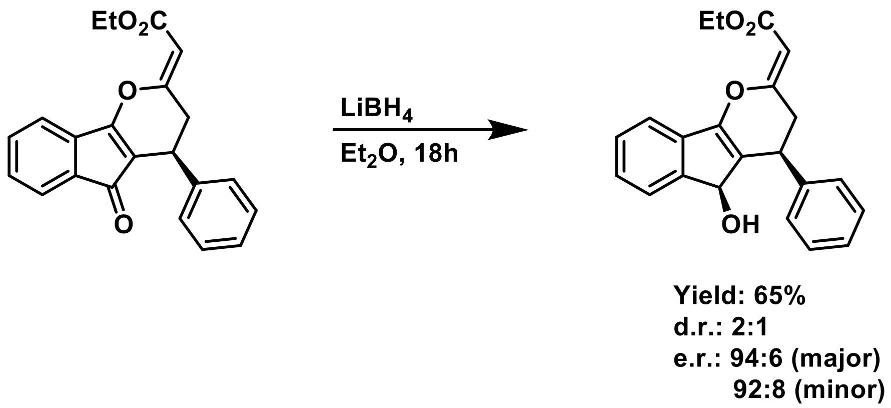

Ethyl (Z)-2-((4S,5S)-5-hydroxy-4-phenyl-4,5-dihydroindeno[1,2-b]pyran-2(3H)-ylidene)acetate **8_{major}** and its minor diastereomer were prepared in analogy to literature [22].

First, the starting material (17.2 mg, 0.05 mmol) was dissolved in in 3 mL Et_2O, then $LiBH_4$ (8.9 mg, 0.4 mmol) were added, and the mixture stirred at room temperature for 18 h. The suspension was then quenched with MeOH. Subsequently, it was transferred to a separation funnel, diluted with DCM and washed once with 1M aqueous HCl. The aqueous phase was then extracted thrice with DCM. After pre-drying the combined organic phase with Na_2SO_4, it was evaporated to dryness. Subsequent preparative TLC gave the diastereomers of the product with a combined yield of 65% (d.r.: 2:1, 95:5 er (major), 92:8 er (minor)).

Major:

HPLC (YMC-SA, n-hexane/IPA = 1/1, flow = 0.5 mL min-1, T_{Column} = 10 °C, *l* = 220 nm): t_R: 12.50 min (major), 18.31 min (minor).

^{1}H-NMR (300 MHz, $CDCl_3$, 298 K): δ / ppm = 1.33 (t, *J* = 7.1 Hz, 3 H),
2.61 (dd, J_1 = 14.7 Hz, J_2 = 3.1 Hz, 1 H), 3.06 (ddd, J_1 = 14.7 Hz, J_2 = 6.7 Hz, J_3 = 1.5 Hz, 1 H),
3.53-3.69 (m, 1 H), 3.98 (dd, J_1 = 6.7 Hz, J_2 = 3.1 Hz, 1 H), 4.22 (q, *J* = 6.1 Hz, 1 H),
5,06 (d, *J* = 1.4 Hz, 2 H), 5.16 (s, 1 H), 7.23-7.39 (m, 7 H), 7.44-7.51 (m, 2 H)

^{13}C-NMR (125 MHz, $CDCl_3$, 298 K): δ / ppm = 14.5, 36.7, 37.1, 60.0, 75.7, 101.4, 117.7, 118.3, 123.6, 127.4, 127.5, 127.6 (2 C), 128.7, 129.1 (2 C), 135.4, 142.1, 144.4, 151.2, 160.2, 164.6

HRMS (ESI-TOF): m/z calc. for $C_{22}H_{20}O_4$: 349.1435 $[M+H]^+$; found: 349.1435

$[\alpha]_D^{20}$= -177.3 (*c* 0.29, $CHCl_3$)

Minor:

HPLC (YMC-SA, n-hexane/IPA = 1/1, flow = 0.5 mL min-1, T_{Column} = 10 °C, l = 220 nm): t_R: 10.39 min (major), 13.21 min (minor).

^{1}H-NMR (300 MHz, $CDCl_3$, 298 K): δ / ppm = 1.33 (t, J = 7.1 Hz, 3 H), 2.68 (dd, J_1 = 14.8 Hz, J_2 = 3.8 Hz, 1 H), 3.10 (ddd, J_1 = 14.7 Hz, J_2 = 6.9 Hz, J_3 = 1.3 Hz, 1 H), 3.54-3.70 (m, 1 H), 4.02 (dd, J_1 = 6.9 Hz, J_2 = 3.8 Hz, 1 H), 4.21 (q, J = 6.1 Hz, 1 H), 4.94 (bs, 1 H), 5.11 (d, J = 1.3 Hz, 2 H), 7.19-7.38 (m, 7 H), 7.44-7.48 (m, 2 H)

^{13}C-NMR (125 MHz, $CDCl_3$, 298 K): δ / ppm = 14.5, 22.8, 35.5, 36.6, 60.1, 72.9, 101.1, 118.3, 119.1, 123.8, 127.3, 127.4, 127.7, 128.8, 129.0, 135.8, 142.3, 151.0, 160.6, 164.6

HRMS (ESI-TOF): m/z calc. for $C_{22}H_{20}O_4$: 349.1435 $[M+H]^+$; found: 349.1433

$[\alpha]_D^{20}$= 8.0 (c 0.31, $CHCl_3$)

4.2.2. Hydrogenation utilizing $LiAlH_4$

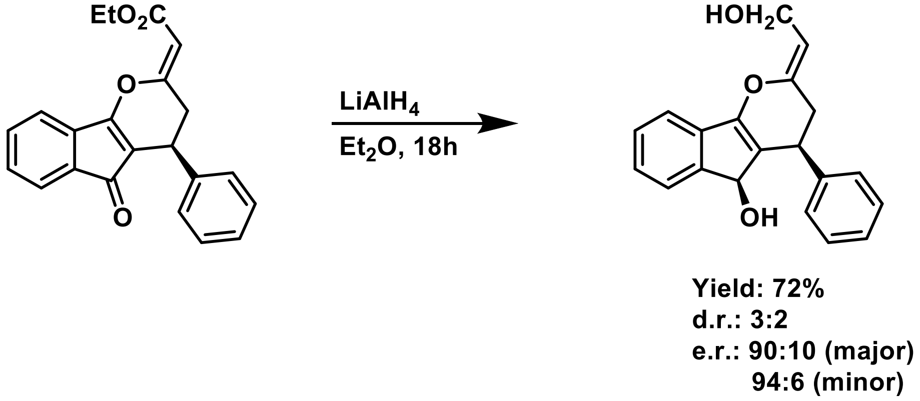

(4S,5S,Z)-2-(2-Hydroxyethylidene)-4-phenyl-2,3,4,5-tetrahydroindeno[1,2-b]pyran-5-ol **9** was prepared in analogy to literature [22].

First, the starting material (18.3 mg, 0.06 mmol) was dissolved in in 3 mL Et_2O, then $LiAlH_4$ (15.9 mg, 0.4 mmol) were added and the mixture stirred at room temperature for 18 h. The suspension was then quenched with MeOH. Subsequently, it was transferred to a separation funnel, diluted with DCM and washed once with 1M aqueous HCl. The aqueous phase was then extracted thrice with DCM. After pre-drying the combined organic phase with Na_2SO_4, it was evaporated to dryness. Subsequent preparative TLC gave the diastereomers of the product with a combined yield of 72% (d.r.: 3:2, 90:10 er (major), 94:6 er (minor)).

Major:

HPLC (YMC-SA, n-hexane/IPA = 1/1, flow = 0.5 mL min-1, T_{Column} = 10 °C, l = 220 nm): t_R: 14.43 min (major), 16.27 min (minor).

^{1}H-NMR (300 MHz, $CDCl_3$, 298 K): δ / ppm = 2.52 (dd, J_1 = 14.1 Hz, J_2 = 3.3 Hz, 1 H), 2.94 (ddd, J_1 = 14.1 Hz, J_2 = 6.3 Hz, J_3 = 1.3 Hz, 1 H),3.54-3.70 (m, 1 H), 3.94 (dd, J_1 = 6.3 Hz, J_2 = 3.2 Hz, 1 H), 4.33-4.52 (m, 2 H), 4.85 (dt, J_1 = 6.8 Hz, J_2 = 3.2 Hz, 1 H), 5.12 (s, 1 H), 7.22-7.38 (m, 7 H), 7.43-7.48 (m, 1 H)

^{13}C-NMR (175 MHz, $CDCl_3$, 298 K): δ / ppm = 35.7, 38.0, 56.5, 75.8, 110.1, 116.7, 117.6, 123.6, 127.2 (2 C), 127.8 (2 C), 128.5, 128.9 (2 C), 136.0, 143.1, 144.7, 148.7, 151.7

HRMS (ESI-TOF): m/z calc. for $C_{20}H_{18}O_3$: 307.1329 $[M+H]^+$; found: 307.1327

$[\alpha]_D^{20}$= -100.5 (c 0.39, $CHCl_3$)

Minor:

HPLC (YMC-SA, n-hexane/IPA = 1/1, flow = 0.5 mL min-1, T_{Column} = 10 °C, *l* = 220 nm): t_R: 9.00min (major), 13.42 min (minor).

^{1}H-NMR (300 MHz, $CDCl_3$, 298 K): δ / ppm = 2.59 (dd, J_1 = 14.3 Hz, J_2 = 4.2 Hz, 1 H), 2.97 (ddd, J_1 = 14.3 Hz, J_2 = 6.5 Hz, J_3 = 1.1 Hz, 1 H), 3.56-3.70 (m, 1 H), 3.97 (dd, J_1 = 6.4 Hz, J_2 = 4.4 Hz, 1 H), 4.32-4.50 (m, 2 H), 4.85-4.93 (m, 2 H), 7.19-7.35 (m, 7 H), 7.42-7.46 (m, 1 H)

^{13}C-NMR (175 MHz, $CDCl_3$, 298 K): δ / ppm = 29.4, 35.7, 36.4, 56.5, 73.0, 109.7, 117.7, 118.4, 123.8, 127.0, 127.1, 127.9 (2 C), 128.6, 128.8 (2 C), 143.2, 144.8, 149.2, 151.4

HRMS (ESI-TOF): m/z calc. for $C_{20}H_{18}O_3$: 307.1329 $[M+H]^+$; found: 307.1330

$[\alpha]_D^{20}$ = (*c* 1.0, $CHCl_3$)

4.2.1. Hydrogenation utilizing the Wilkinson catalyst

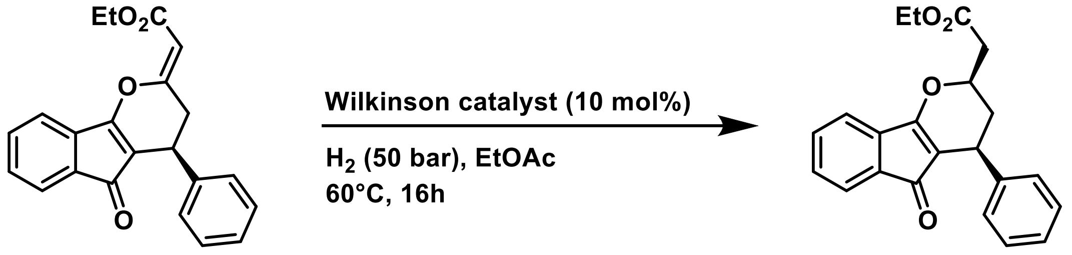

Yield: 71%
d.r.: >95:5
e.r.: 95:5

Ethyl 2-((2R,4S)-5-oxo-4-phenyl-2,3,4,5-tetrahydroindeno[1,2-b]pyran-2-yl)acetate **10** was synthesised in analogy to literature [21]. First, the starting material (34.9 mg, 0.10 mmol) and the Wilkinson catalyst (9.3 mg, 0,01 mmol) were dissolved in 2 mL EtOAc. The vial was put into an autoclave and subsequently flushed three times with H_2. After the third flushing step the hydrogen pressure was set to 50 bar and the autoclave heated to 60 °C for 16 h. After cooling to room temperature, the suspension was filtered over a silica gel plug and washed with acetone before evaporating to dryness. Subsequent preparative TLC gave the product as a white waxy solid in a yield of 71 % (24.9 mg, 0.071 mmol) and 95:5 er.

HPLC (YMC-SB, n-hexane/IPA = 2/1, flow = 0.5 mL min-1, T_{Column} = 10 °C, l = 220 nm): t_R: 40.50 min (major), 51.56 min (minor).

^{1}H-NMR (700 MHz, $CDCl_3$, 298 K): δ / ppm = 1.30 (t, J = 7.5 Hz, 3 H), 1.80 (m, 1 H), 2.37 (ddd, J_1 = 14.1 Hz, J_2 = 6.2 Hz, J_3 = 1.5 Hz, 1 H), 2.67 (dd, J_1 = 16.0 Hz, J_2 = 6.1 Hz, 1 H), 2.91 (dd, J_1 = 16.0 Hz, J_2 = 7.3 Hz, 1 H), 3.85 (dd, J_1 = 10.4 Hz, J_2 = 6.1 Hz, 1 H), 4.19-4.24 (m, 2 H), 4.80-4.85 (m, 1 H), 7.14-7.16 (m, 1 H), 7.18-7.24 (m, 3 H), 7.25-7.36 (m, 5 H)

^{13}C-NMR (175 MHz, $CDCl_3$, 298 K): δ / ppm = 14.4, 36.1, 38.6, 40.0, 61.1, 76.6, 110.1, 117.9, 121.3, 126.9, 127.4 (2 C), 128.7 (2 C), 130.2, 132.0, 133.1, 137.6, 142.1, 169.9, 174.9, 191.9

HRMS (ESI-TOF): m/z calc. for $C_{22}H_{20}O_4$: 349.1435 $[M+H]^+$; found: 349.1433

$[\alpha]_D^{20}$ = -251.2 (c 1.0, $CHCl_3$)

5. Crystallographic data

The X-ray intensity data of **3p** were measured on a 'XtaLAB Synergy R, HyPix-Arc 150' system equipped with a mirror monochromator and a Cu Kα 'Rotating-anode X-ray tube' (λ = 1.54184 Å). The frames were integrated with the CrysAlisPro software package [23]. Data were corrected for absorption effects using the Multi-Scan method (SCALE3 ABSPACK) integrated in CrysAlisPro. The structure was solved with OLEX2.SOLVE and refined using the SHELXLE Software Package [24]. All C-bound hydrogen atoms have been calculated in ideal geometry riding on their parent atoms. The figures have been drawn at the 25% ellipsoid probability level [25].

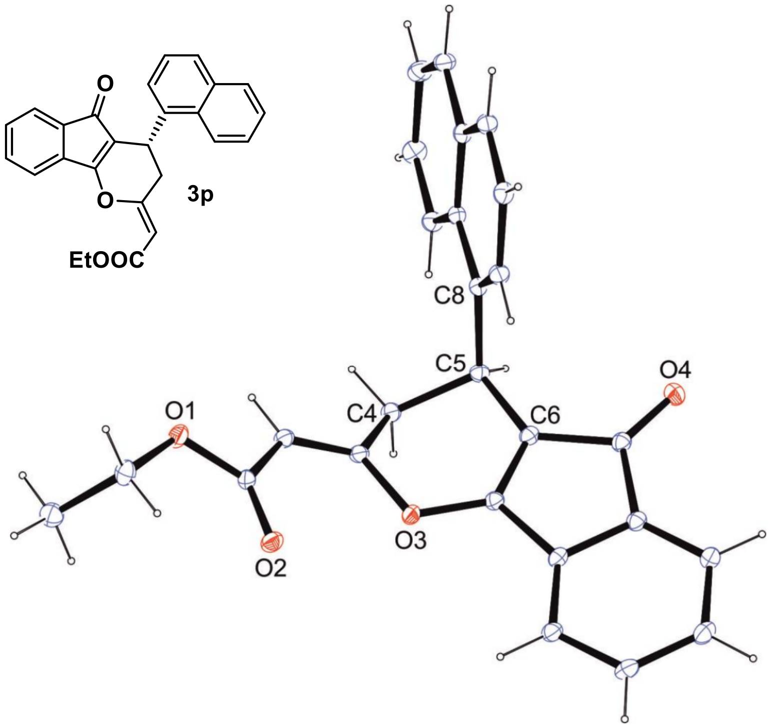

	3p
net formula	$C_{26}H_{20}O_4$
M_r/g mol^{-1}	396.42
crystal size/mm	0.15 × 0.10 × 0.07
T/K	293(2)
radiation	CuKα
diffractometer	'XtaLAB Synergy R, HyPix-Arc 150'
crystal system	orthorhombic
space group	'P 21 21 21'
a/Å	7.75790(7)
b/Å	10.35909(10)
c/Å	24.3667(2)
α/°	90
β/°	90
γ/°	90
V/Å^3	1958.22(3)
Z	4
calc. density/g cm^{-3}	1.345
μ/mm^{-1}	0.729
absorption correction	multi-scan
transmission factor range	0.87843–1.00000
refls. measured	22027
R_{int}	0.0268
mean σ(I)/I	0.0178
θ range	3.628–75.442
observed refls.	3879
x, y (weighting scheme)	0.0493, 0.4001
hydrogen refinement	constr
Flack parameter	0.04(12)
refls in refinement	4003
parameters	273
restraints	0
$R(F_{obs})$	0.0319
$R_w(F^2)$	0.0851
S	1.069
shift/error$_{max}$	0.001
max electron density/e Å^{-3}	0.226
min electron density/e Å^{-3}	–0.206

6. References

[1] M. Kobayashi, S. Okamoto, *Tetrahedron Lett*. (**2006**), 47, 4347-4350.

[2] C. M. Young, A. Elmi, D. J. Pascoe, R. K. Morris, C. McLaughlin, A. M. Woods, A. B. Frost, A. Houpliere, K. B. Ling, T. K. Smith, A. M. Z. Slawin, P. H. Willoughby, S. Cockroft, A. D. Smith, *Angew. Chem., Int. Ed*. (**2020**), *59*, 3705−3710.

[3] S. Li, H. Qu, L. Zhou, K.-i. Kanno, Q. Guo, B. Shen, T. Takahashi, *Org. Lett.* (**2009**), 11, 15, 3318-3321.

[4] D. R. Buckle, N. J. Morgan, J. W. Ross, H. Smith, B. A. Spicer, *J. Med. Chem.* (**1973**) 16, 12, 1334-1339.

[5] S. Li, L. Ye, W. Zhao, S. Zhang, S. Mukherjee, H. Ade, J. Hou, *Adv. Mat.* (**2016**), 28, 42, 9423-9429.

[6] C.-H. Lin, K.-H. Lin, B. Pal, L.-D. Tsou, *Chem. Commun.* (**2009**), 803-805.

[7] C. Pigot, G. Noirbent, T.-T. Bui, S. Péralta, D. Gigmes, M. Nechab, F. Dumur, *Mater.* (**2019**), 12, 1342-1365.

[8] M. Harig, B. Neumann, H.-G. Stammler, D. Kuck, *ChemPlusChem* (**2017**), 82, 1078-1095.

[9] C. Fetzter, V. S. Korotkov, R. Thänert, K. M. Lee, M. Neuenschwander, J. P. von Kries, E. Medina, S. A. Sieber, *Angew. Chem. Int. Ed.* (**2017**), 56, 15746-15750.

[10] S. Li, L. Zhan, Y. Jin, G. Zhou, T.-K. Lau. R. Qin, M. Shi, C.-Z. Li, H. Zhu, X. Lu, F. Zhang, H. Chen, *Adv. Mater.* (**2020**), 32, 24, 2001160.

[11] B. Bano, Kanwal, K. M. Khan, F. Begum, M. A. Lodhi, U. Salar, R. Khalil, Z. Ul-Haq, S. Perveen, *Bioorg. Chem.* (**2018**), 81, 658 671.

[12] C.-J. Lee, C.-N. Sheu, C.-C. Tsai, Z.-Z. Wu, W. Lin, *Chem. Commun.* (**2014**), 50, 5304-5306.

[13] A. Alizadeh, Z. Beiranvand, Z. Safaei, M. M. Khodaei, E. Repo, *ACS Omega* (**2020**), 5 ,44, 28632-28636.

[14] N. Parui, T. Mandal, J. Dash, *Eur. J. Org. Chem.* (**2023**), 26, e202201285.

[15] A. Mondal, R. Hazra, J. Grover, M. Raghu, S. S. V. Ramasastry, *ACS Catal.* (**2018**), 8, 4, 2748-2753.

[16] Y. Mao, F. Mathey, *Org. Lett.* (**2012**), 14, 1162–1163.

[17] T. M. Werkhoven, R. van Nispen, and J. Lugtenburg, Eur. J. Org. Chem., 1999, 2909.

[18] R. A. Aitken, J. M. Armstrong, M. J. Drysdale, F. C. Ross, and B. M. Ryan, J. Chem. Soc., Perkin Trans.1,1999, 593.

[19] G. Wang, X. Liu, Y. Chen, J. Yang, J. Li, L. Lin, X. Feng. *ACS Catal.* (**2016**), 6, 2482-2486.

[20] S. Petersen, J. M. Alonso, A. Specht, P. Duodu, M. Goeldner, A. del Campo, *Angew. Chem. Int. Ed.* (**2018**), 47, 17, 3192-3195.

[21] K. Kasten, D. B. Cordes, A. M. Z. Slawin, A. D. Smith, *Eur. J. Org. Chem.* (**2016**), 21, 3619-3624.

[22] M. N. Iskander, P. R. Andrews, *J. Chem. Edu.* (**1985**), 62, 913.

[23] Rigaku Oxford Diffraction (**2023**). CrysAlisPro.

[24] Huebschle, C. B., Sheldrick, G.M., Dittrich, B. *J. Appl. Cryst.* (**2011**). 44, 1281-1284.

[25] Farrugia, L. J. *J. Appl. Cryst.* (**2012**). 45, 849-854.

7. NMR Spectra of New Compounds

NMR of **1f**

^{1}H-NMR (700 MHz, $CDCl_3$, 298 K):

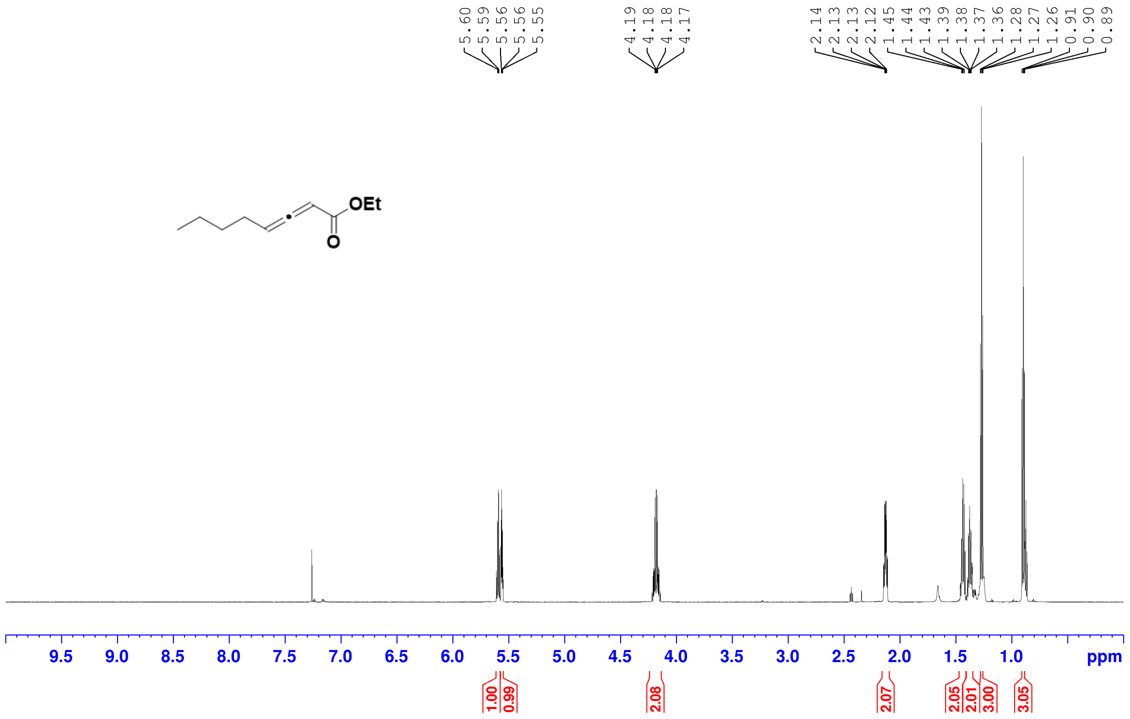

^{13}C-NMR (175 MHz, $CDCl_3$, 298 K):

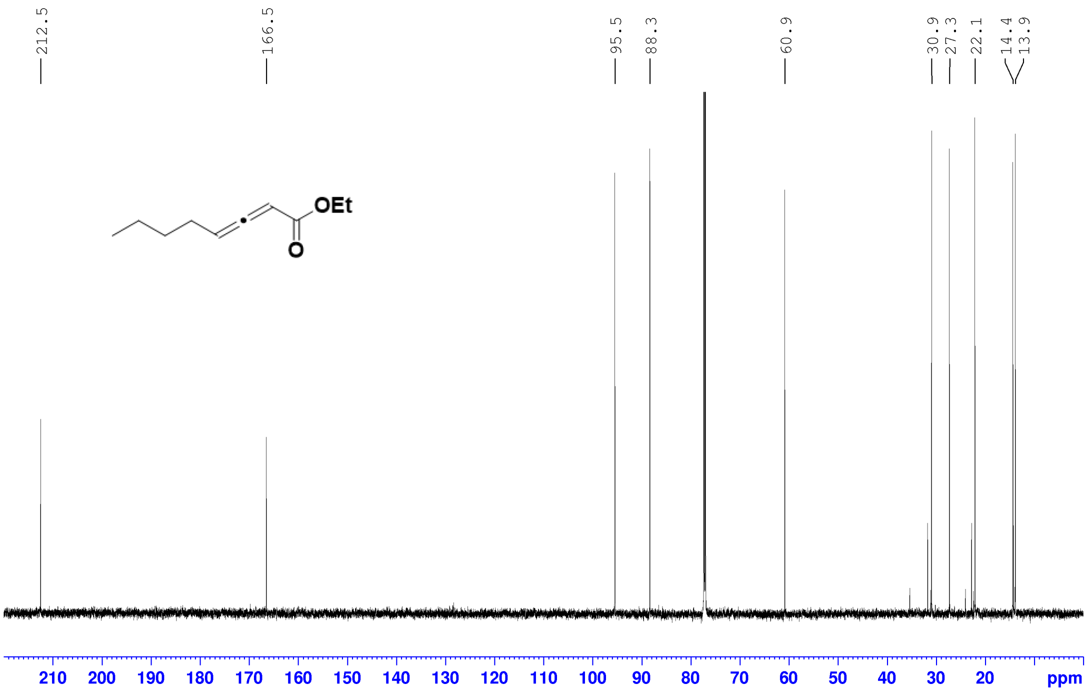

NMR of **1g**

^{1}H-NMR (700 MHz, $CDCl_3$, 298 K):

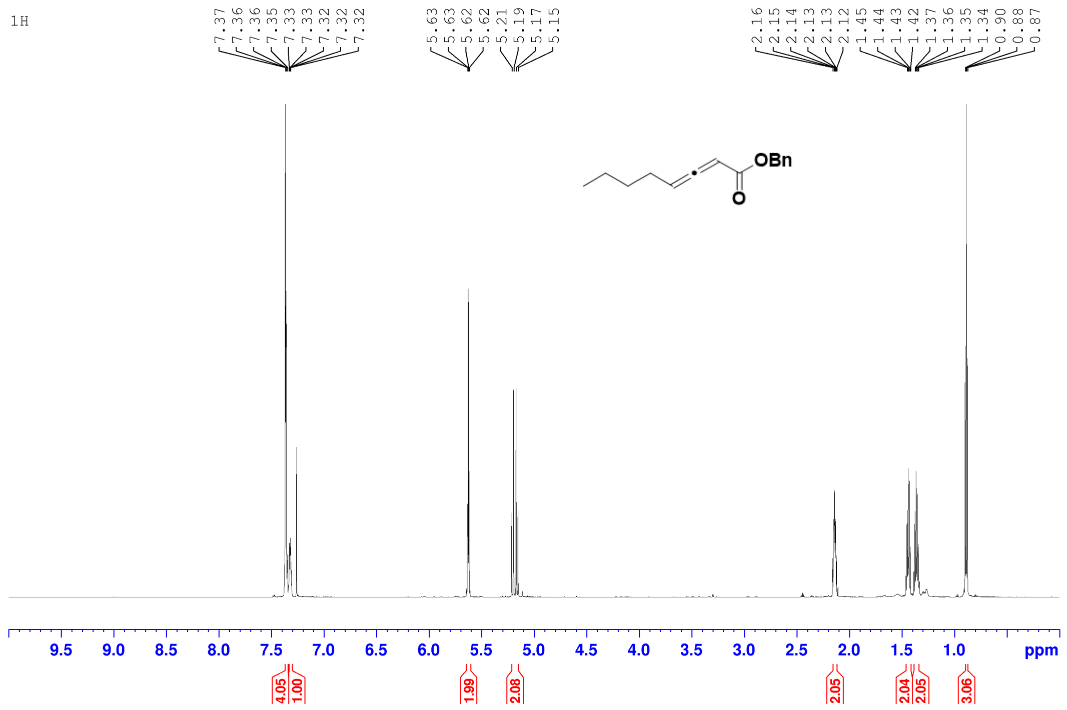

^{13}C-NMR (175 MHz, $CDCl_3$, 298 K):

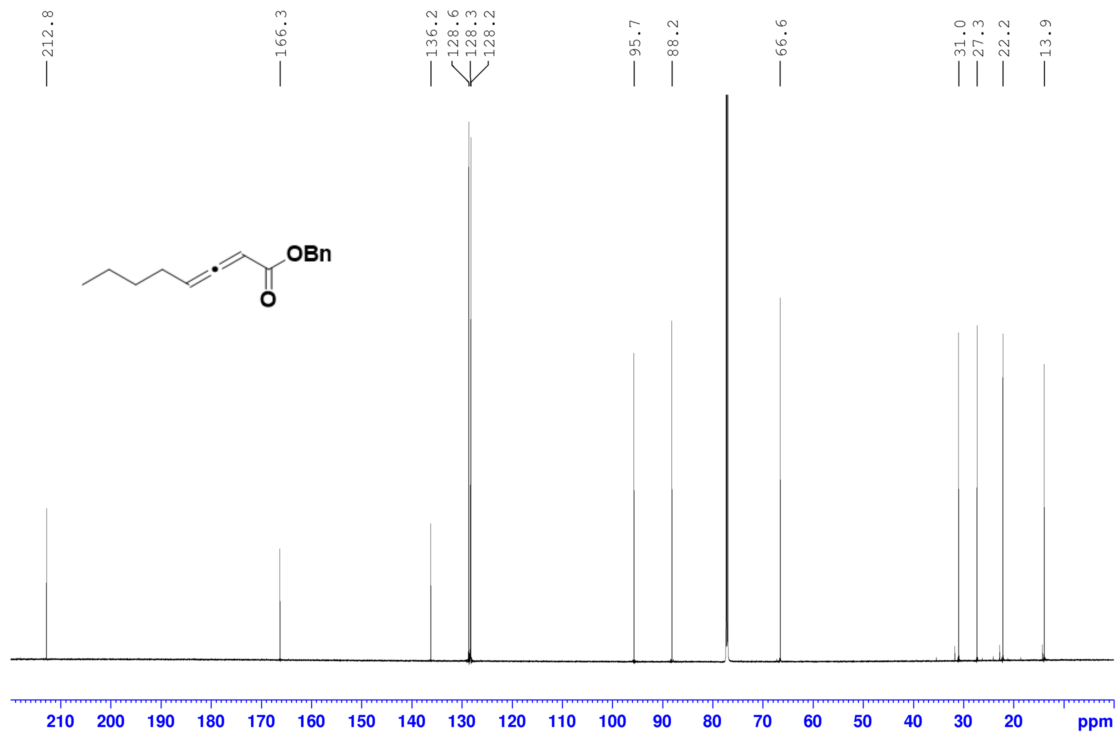

NMR of **2d**

^{1}H-NMR (300 MHz, $CDCl_3$, 298 K):

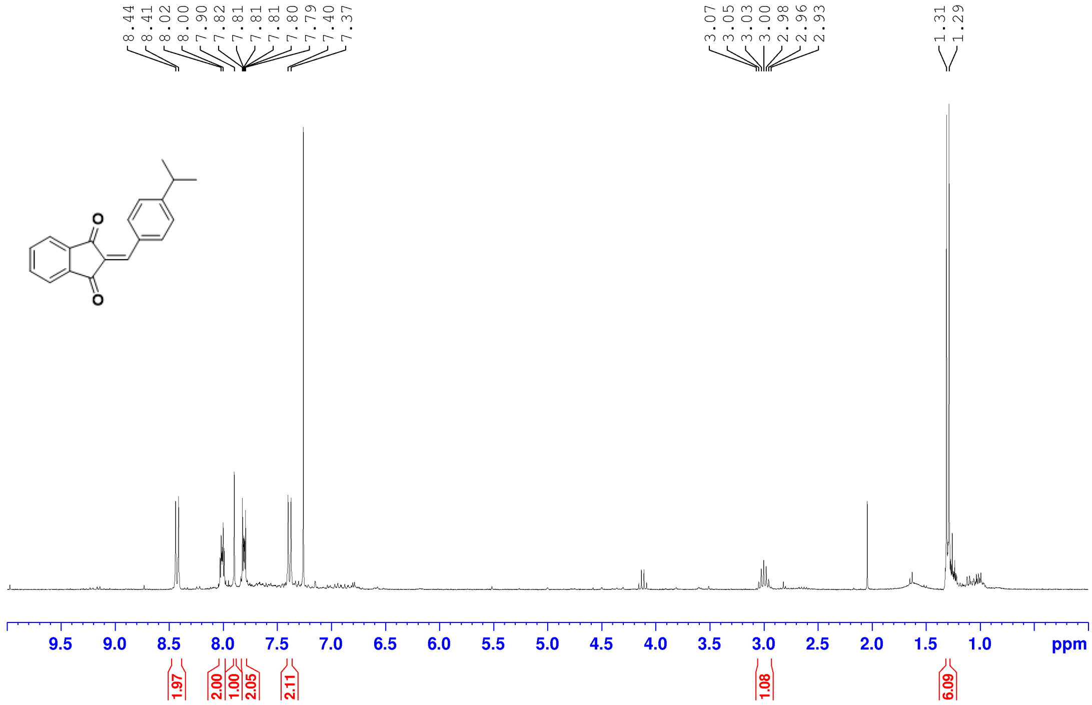

^{13}C-NMR (175 MHz, $CDCl_3$, 298 K):

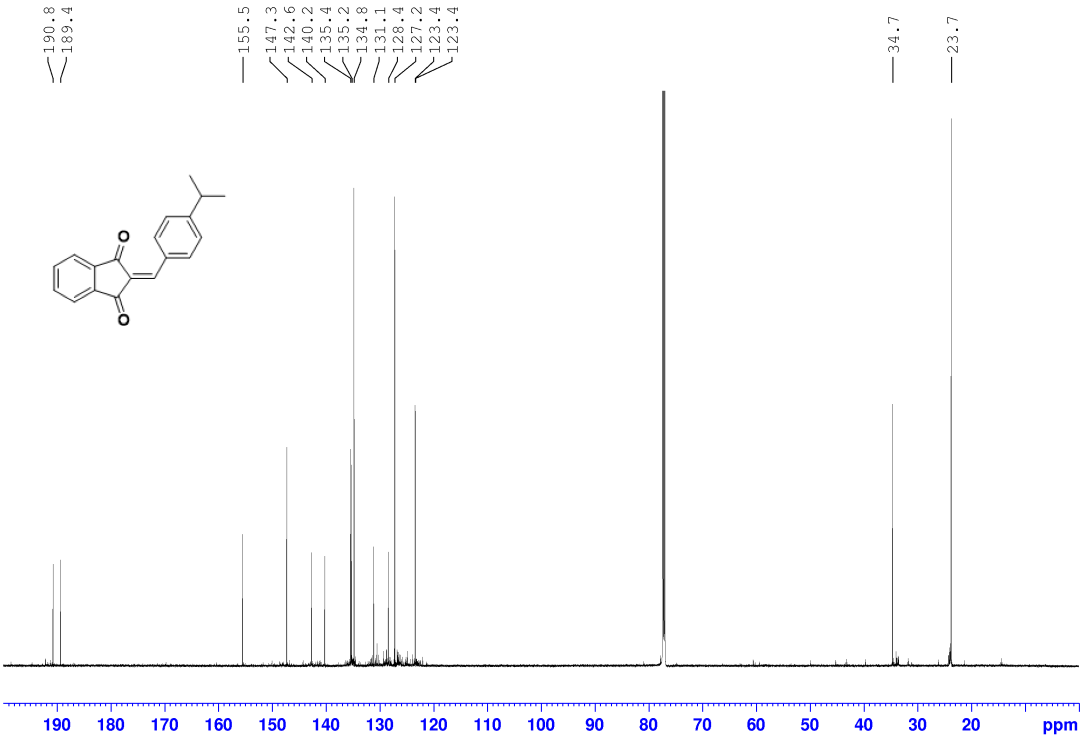

NMR of **2e**

^{1}H-NMR (700 MHz, $CDCl_3$, 298 K):

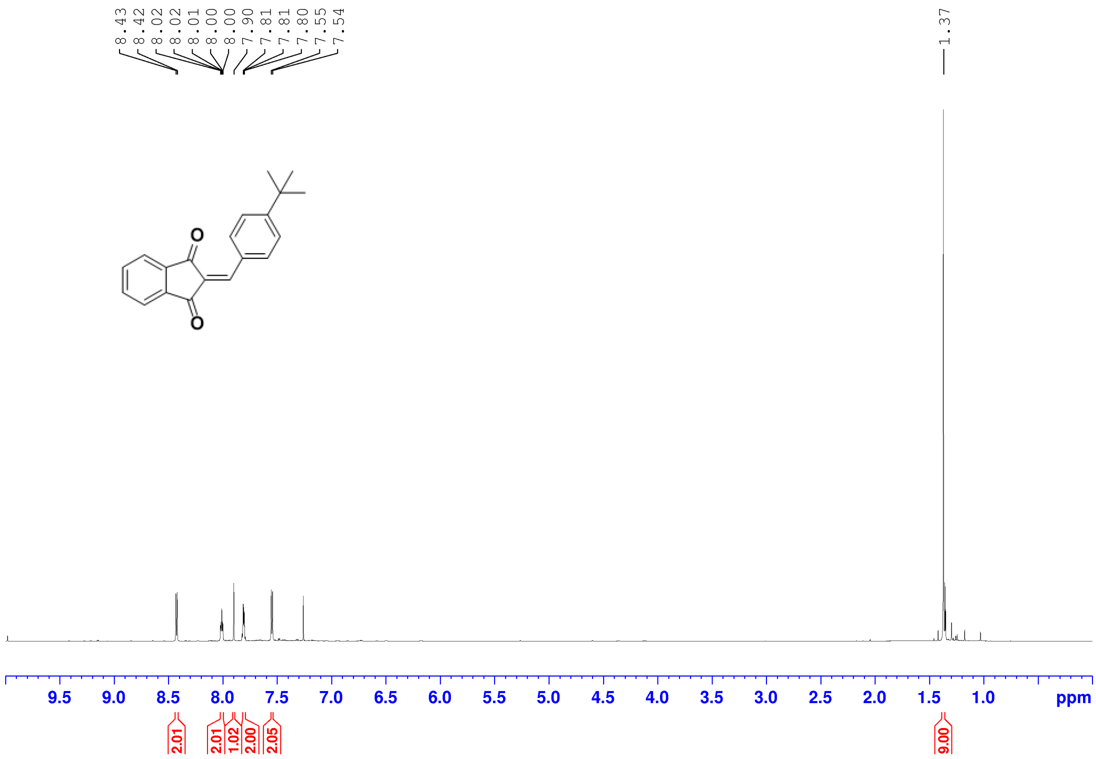

^{13}C-NMR (175 MHz, $CDCl_3$, 298 K):

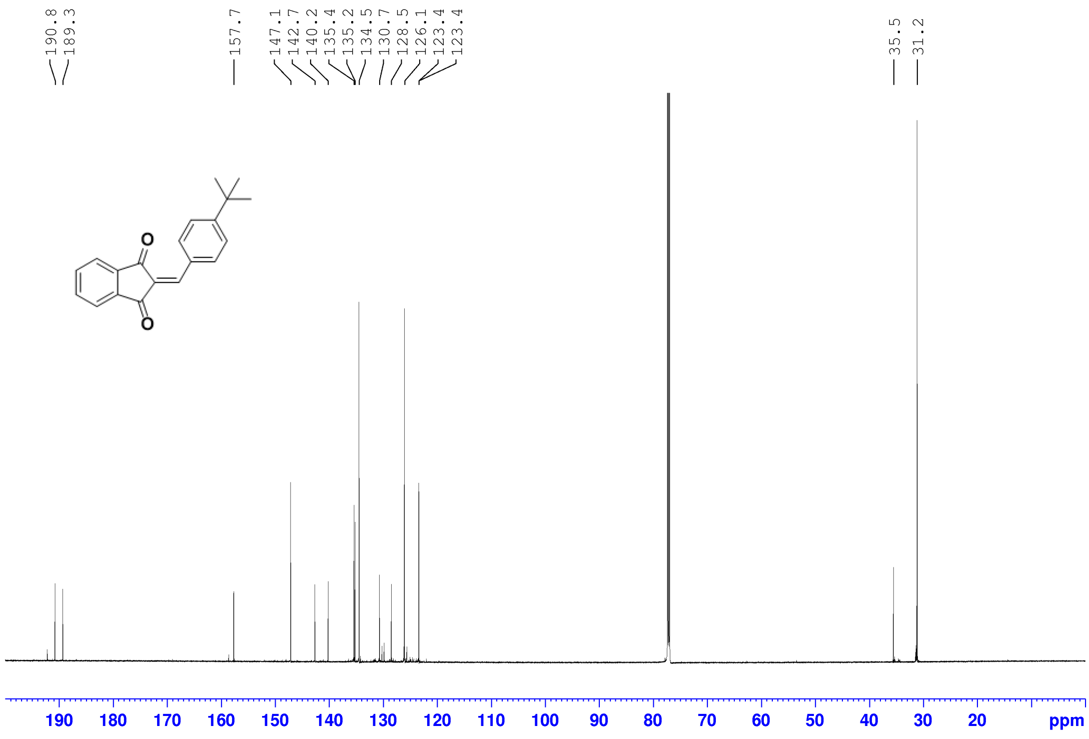

NMR of **2f**

^{1}H-NMR (300 MHz, $CDCl_3$, 298 K):

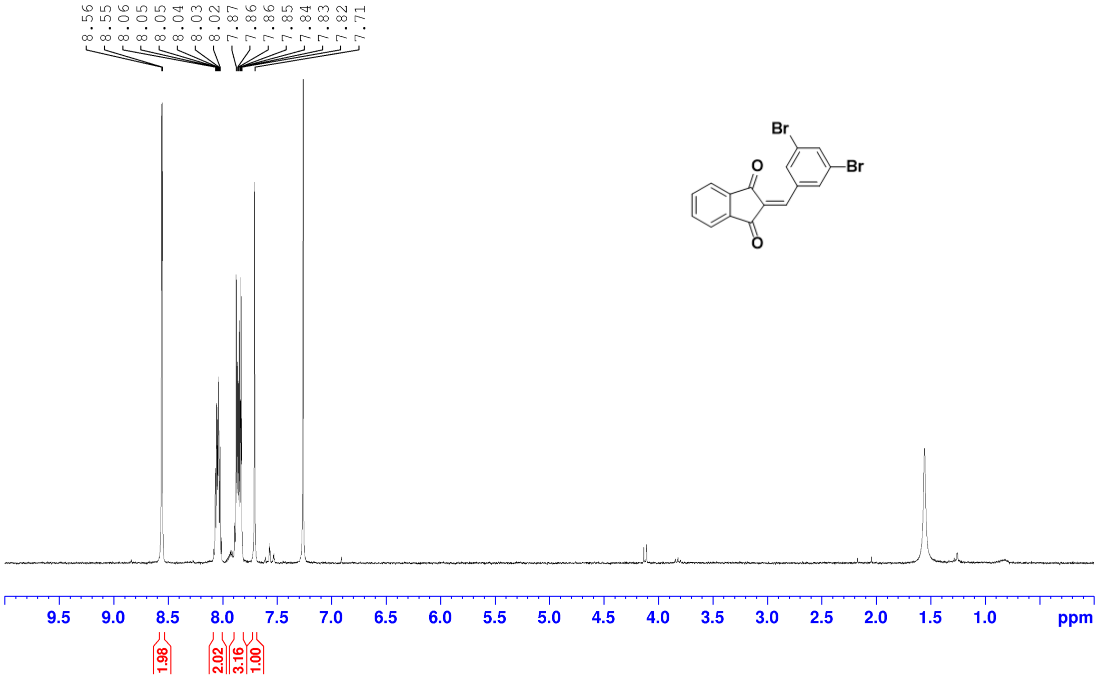

^{13}C-NMR (175 MHz, $CDCl_3$, 298 K):

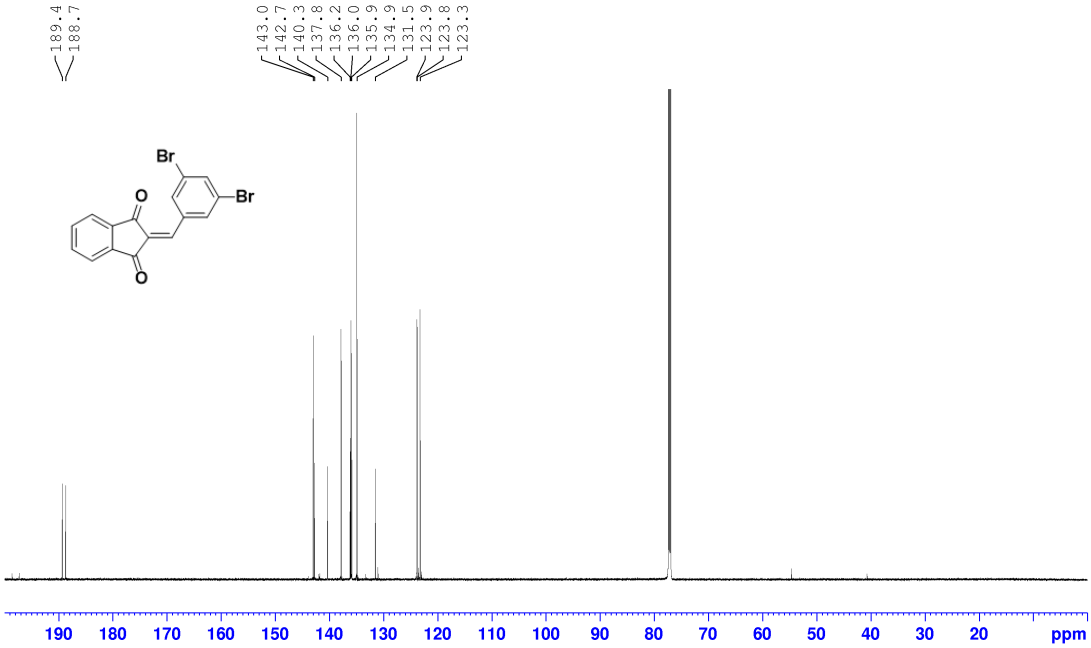

NMR of **2g**

^{1}H-NMR (300 MHz, $CDCl_3$, 298 K):

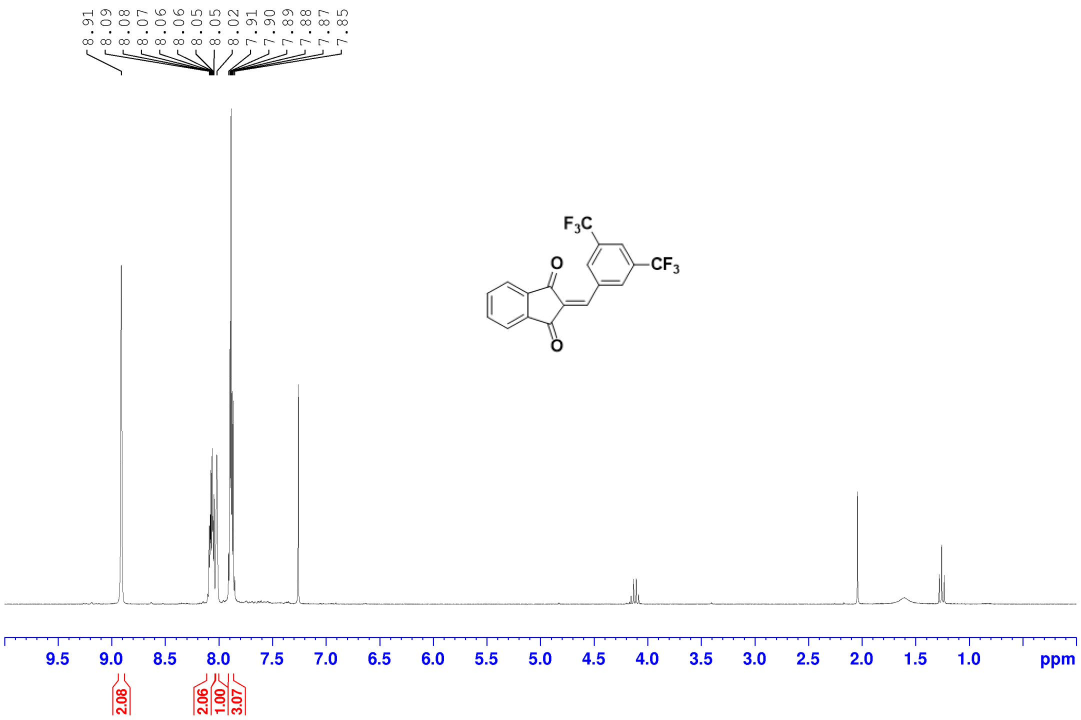

^{19}F-NMR (282 MHz, $CDCl_3$ 298 K)

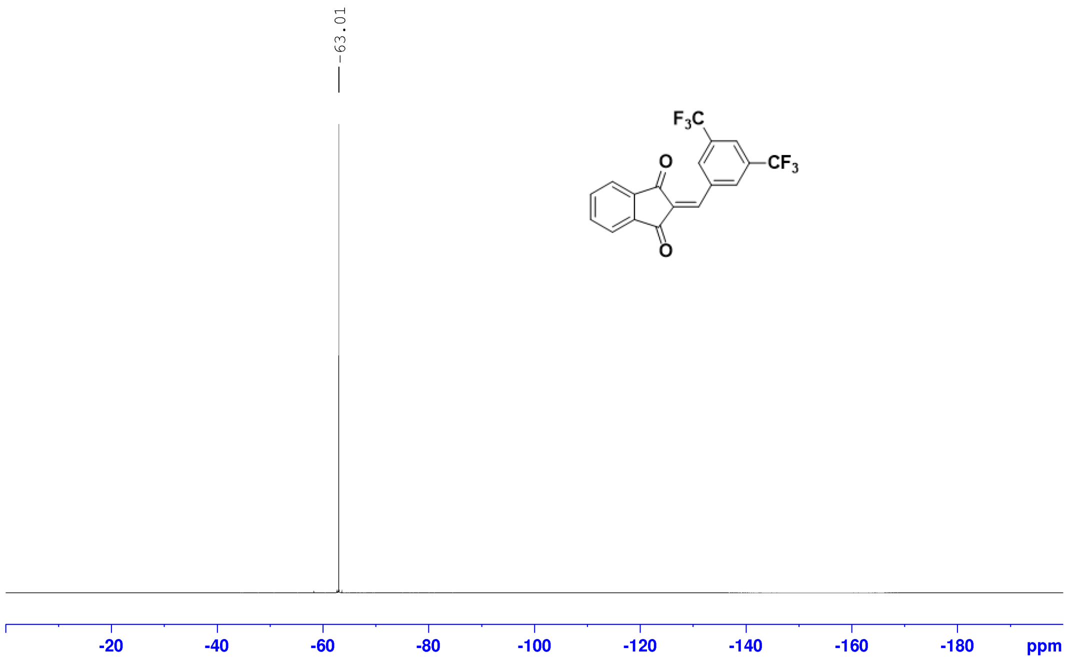

^{13}C-NMR (175 MHz, $CDCl_3$, 298 K):

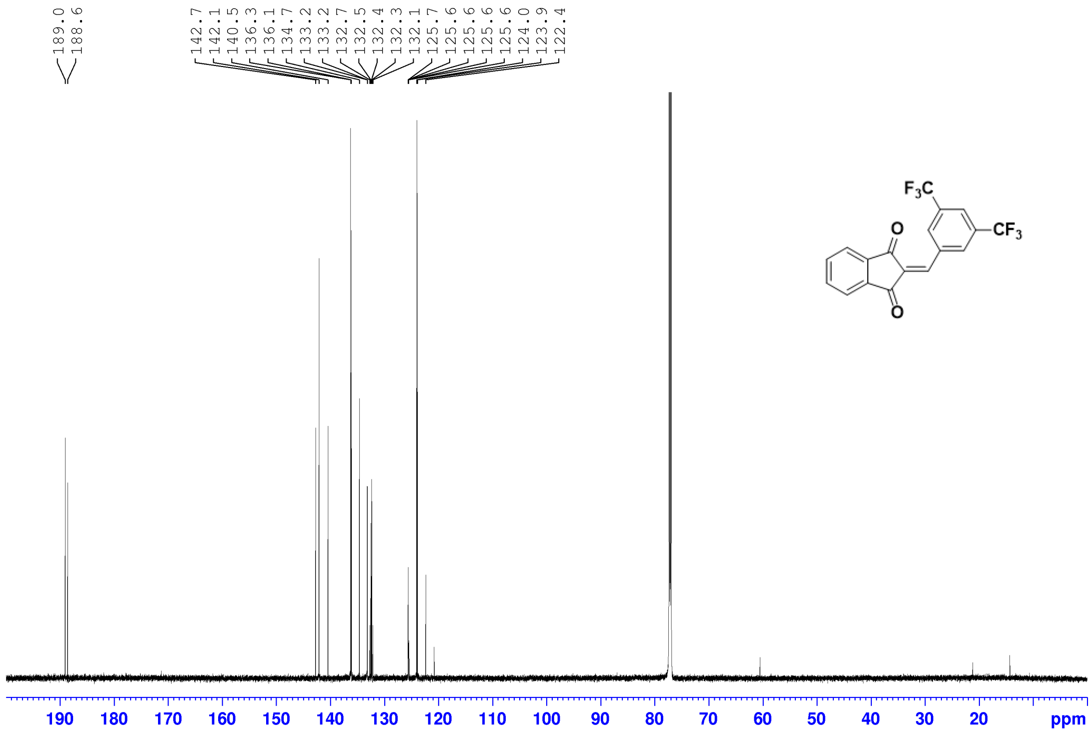

NMR of **2h**

^{1}H-NMR (300 MHz, $CDCl_3$, 298 K):

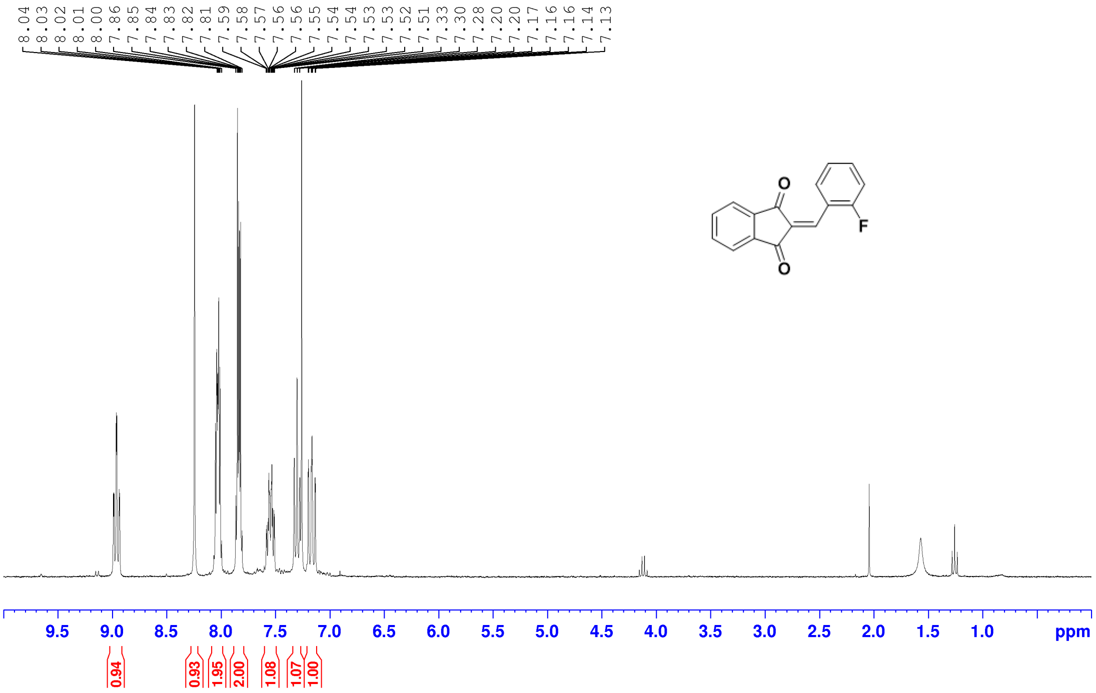

^{19}F-NMR (282 MHz, $CDCl_3$ 298 K):

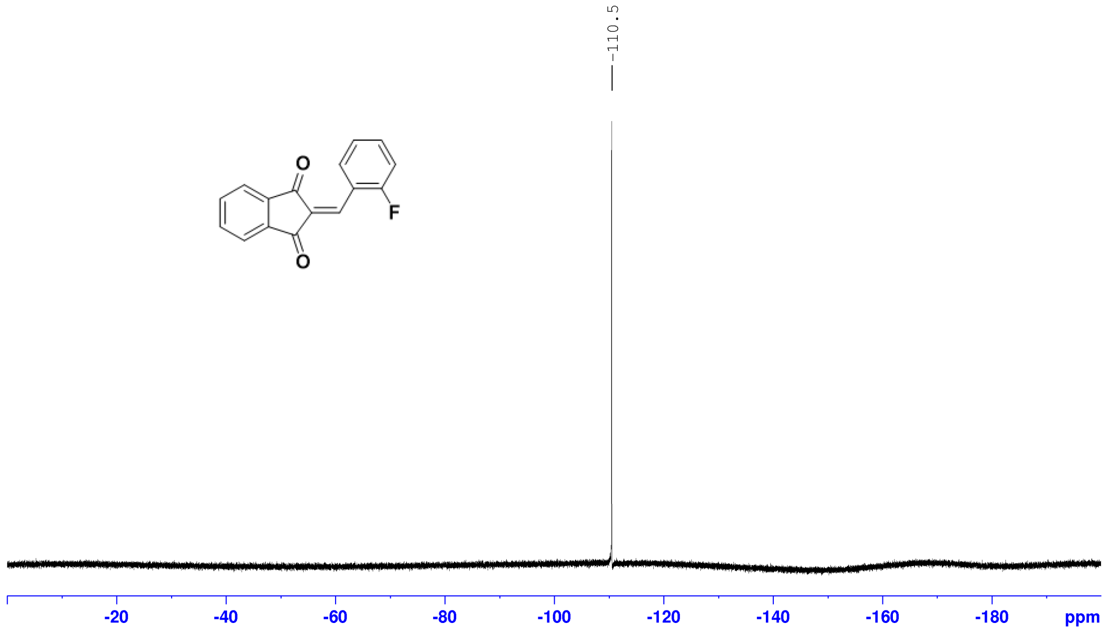

^{13}C-NMR (175 MHz, $CDCl_3$, 298 K):

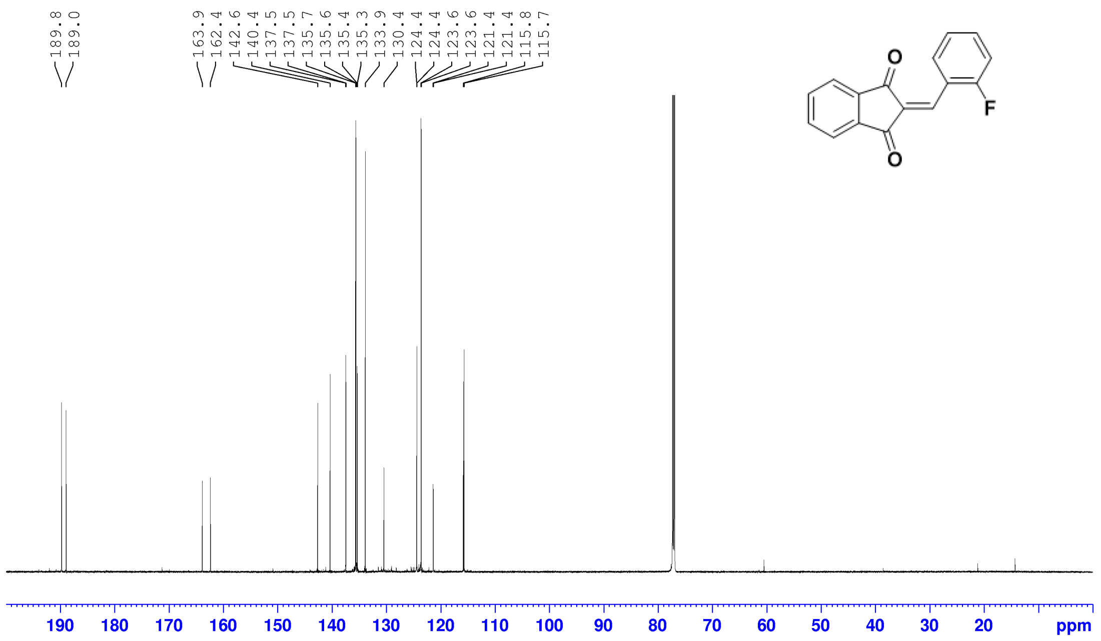

NMR of **2r**

^{1}H-NMR (300 MHz, $CDCl_3$, 298 K):

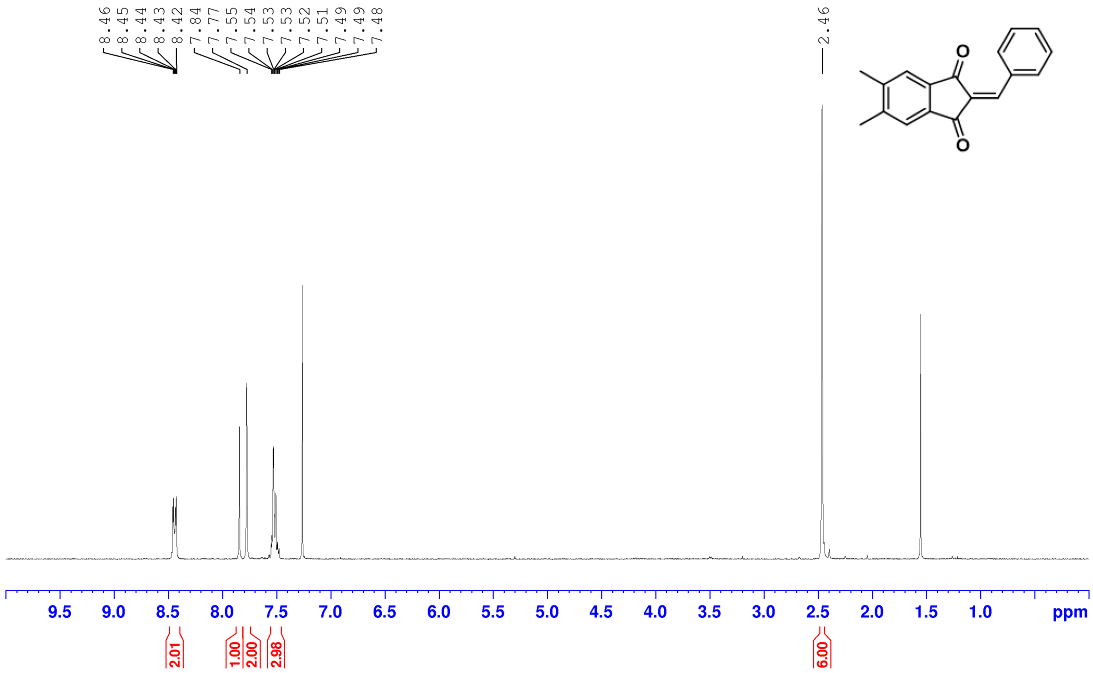

^{13}C-NMR (175 MHz, $CDCl_3$, 298 K):

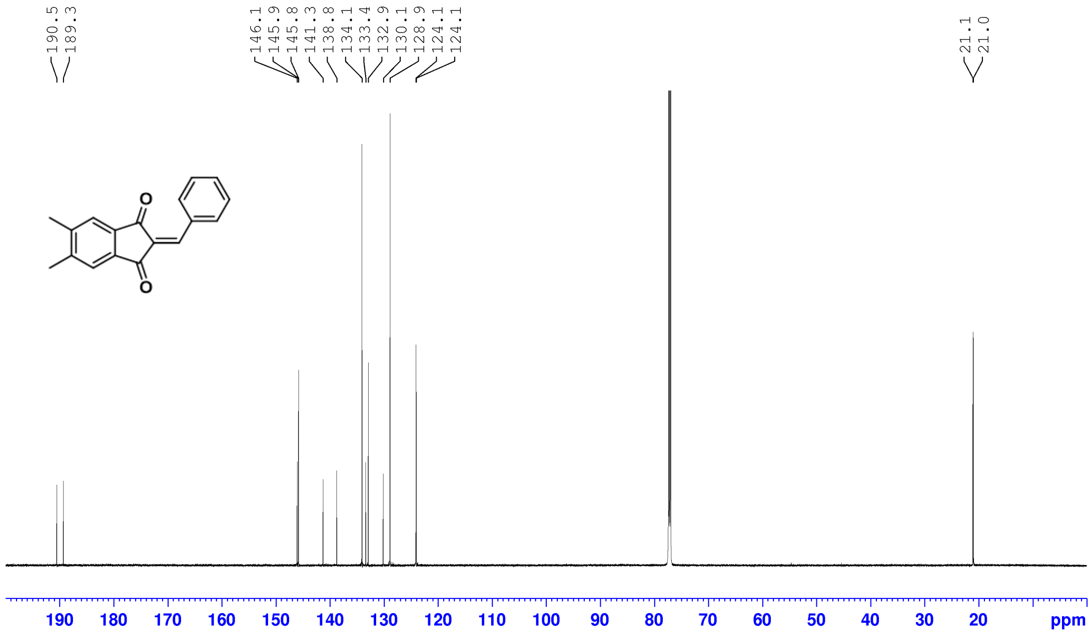

NMR of **2s**

^{1}H-NMR (300 MHz, $CDCl_3$, 298 K):

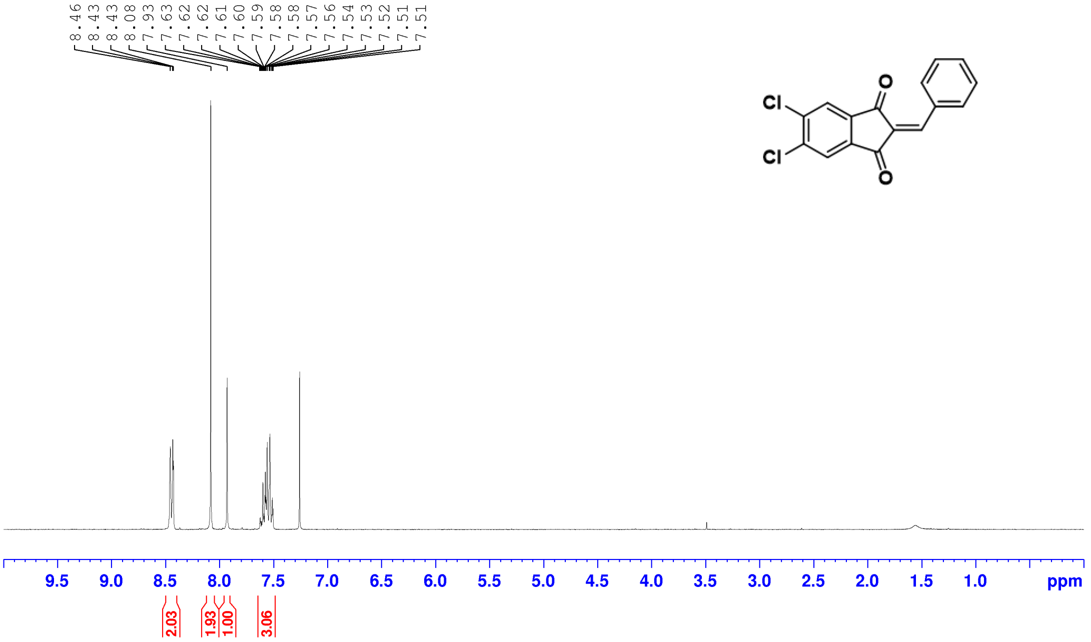

^{13}C-NMR (175 MHz, $CDCl_3$, 298 K):

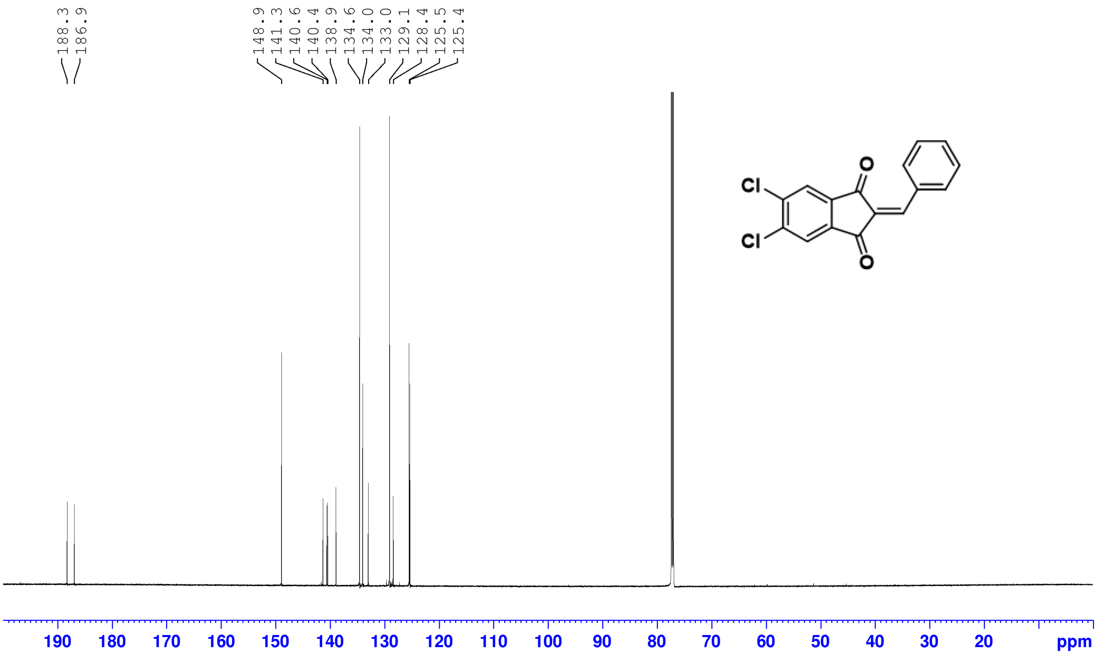

NMR of **2t**

^{1}H-NMR (300 MHz, $CDCl_3$, 298 K):

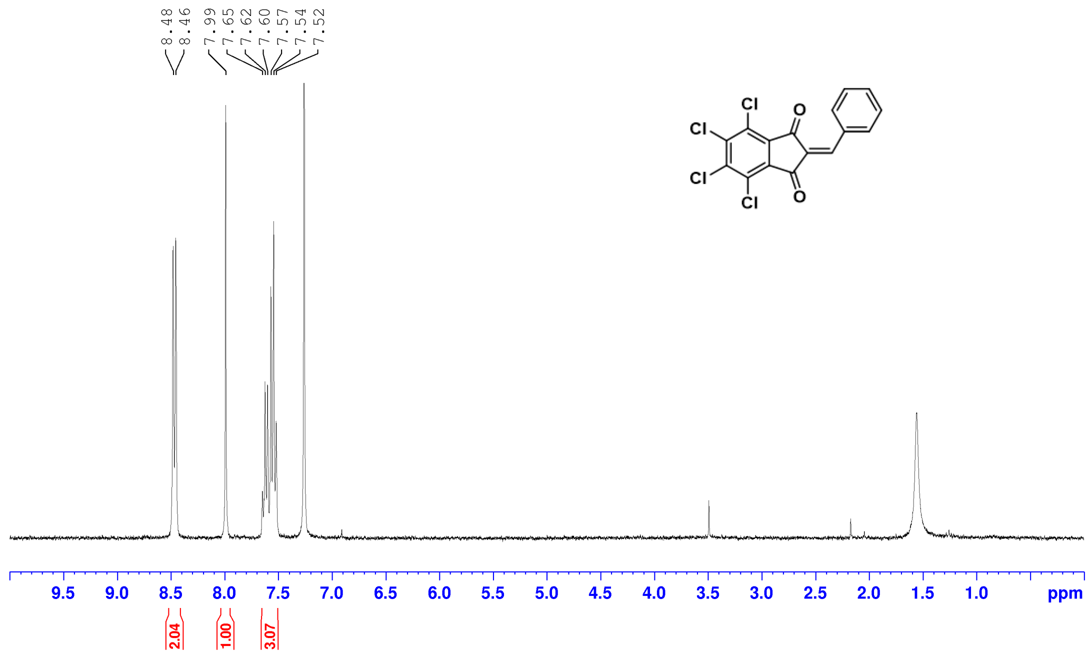

^{13}C-NMR (175 MHz, $CDCl_3$, 298 K):

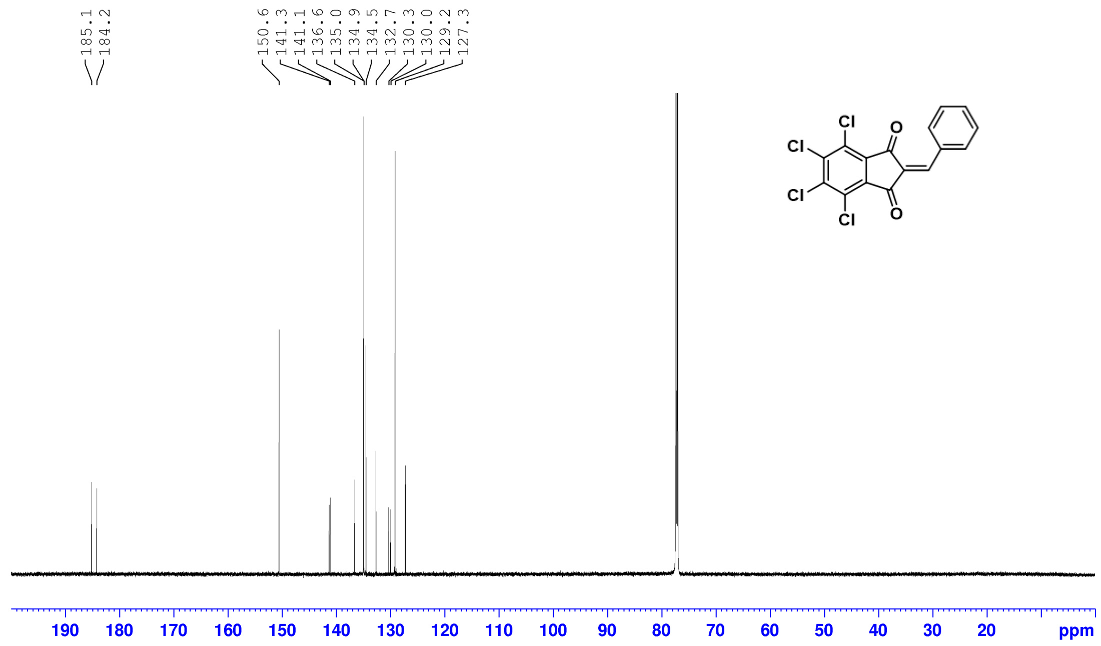

NMR of **3a**

^{1}H-NMR (500 MHz, $CDCl_3$, 298 K):

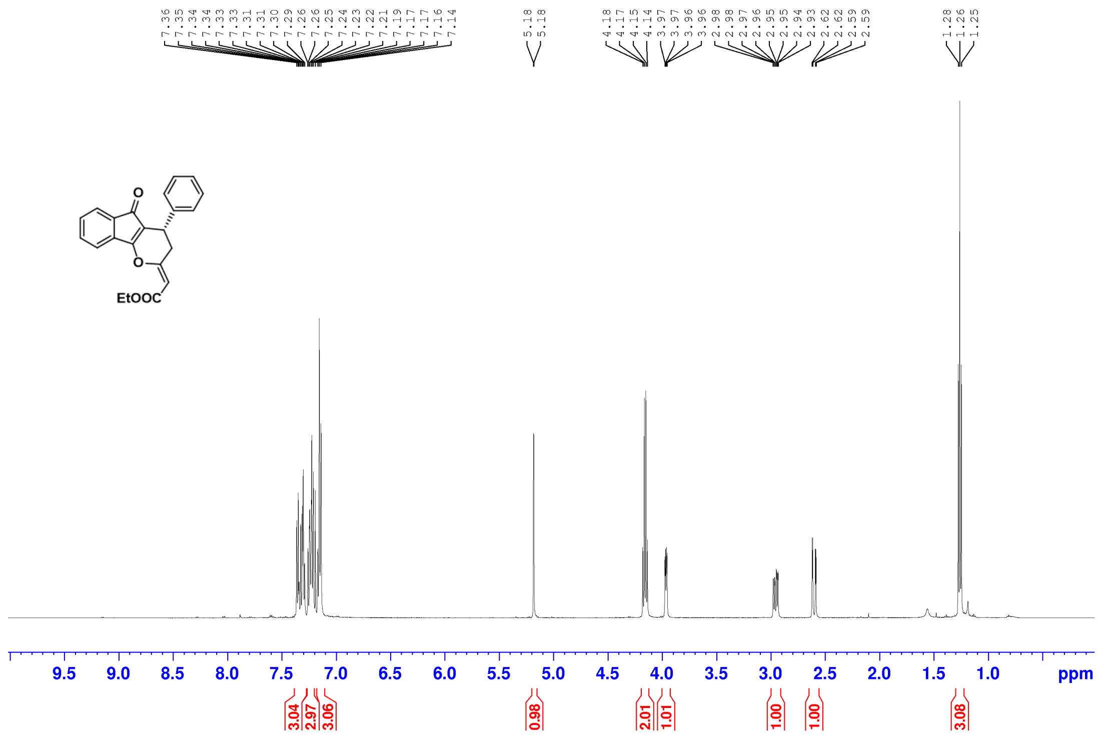

^{13}C-NMR (125 MHz, $CDCl_3$, 298 K)

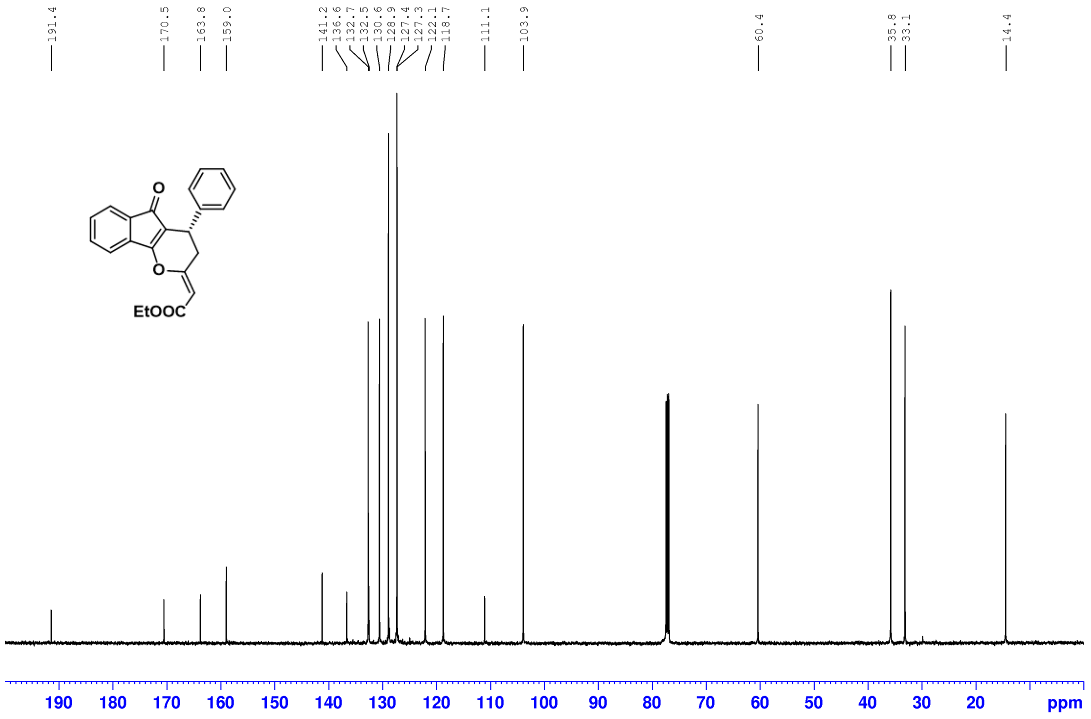

NMR of **3b**

H-NMR (300 MHz, $CDCl_3$, 298 K)

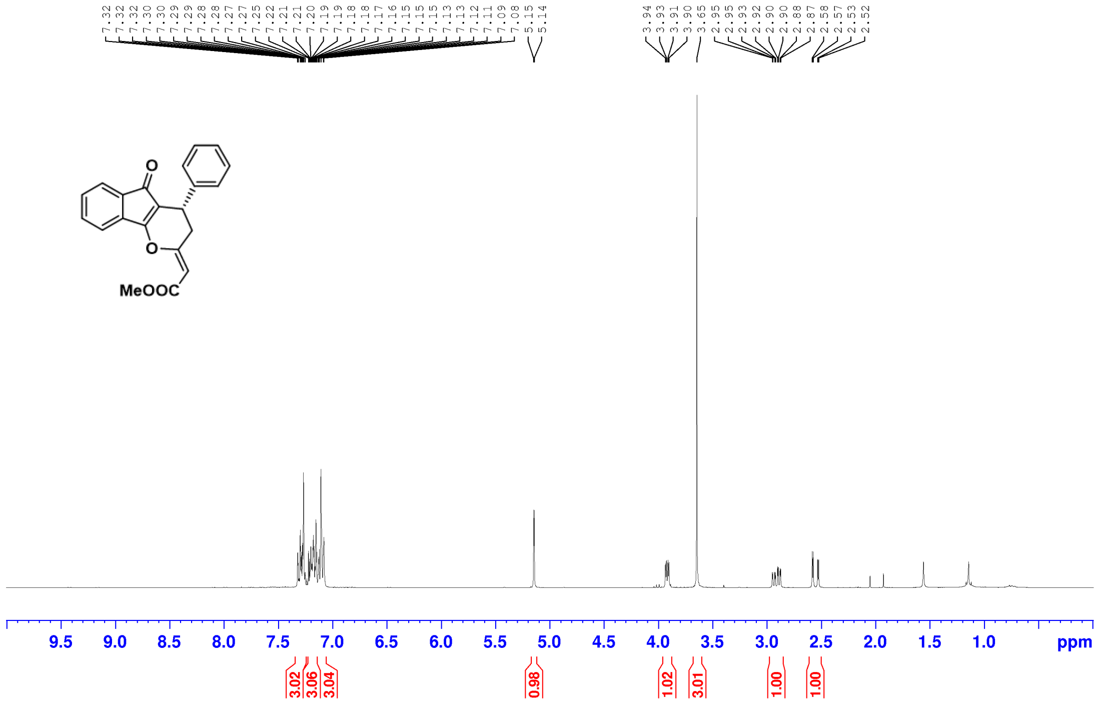

^{13}C-NMR (75 MHz, CDCl3, 298 K)

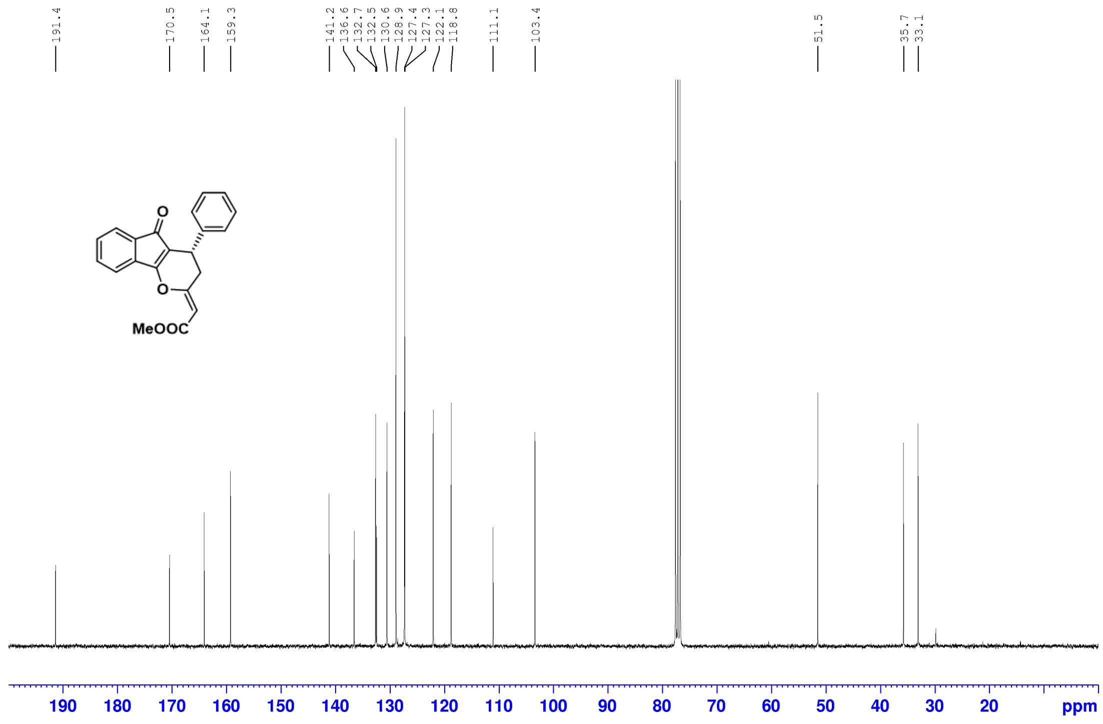

NMR of **3c**

^{1}H-NMR (300 MHz, $CDCl_3$, 298 K)

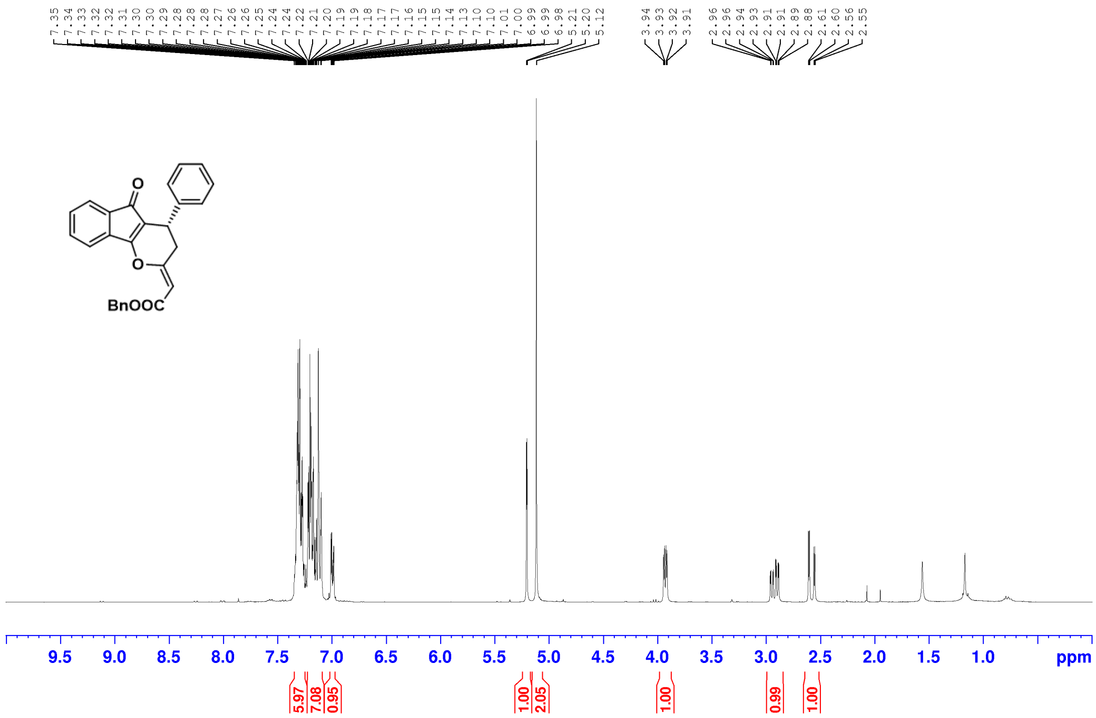

^{13}C-NMR (75 MHz, $CDCl_3$, 298 K)

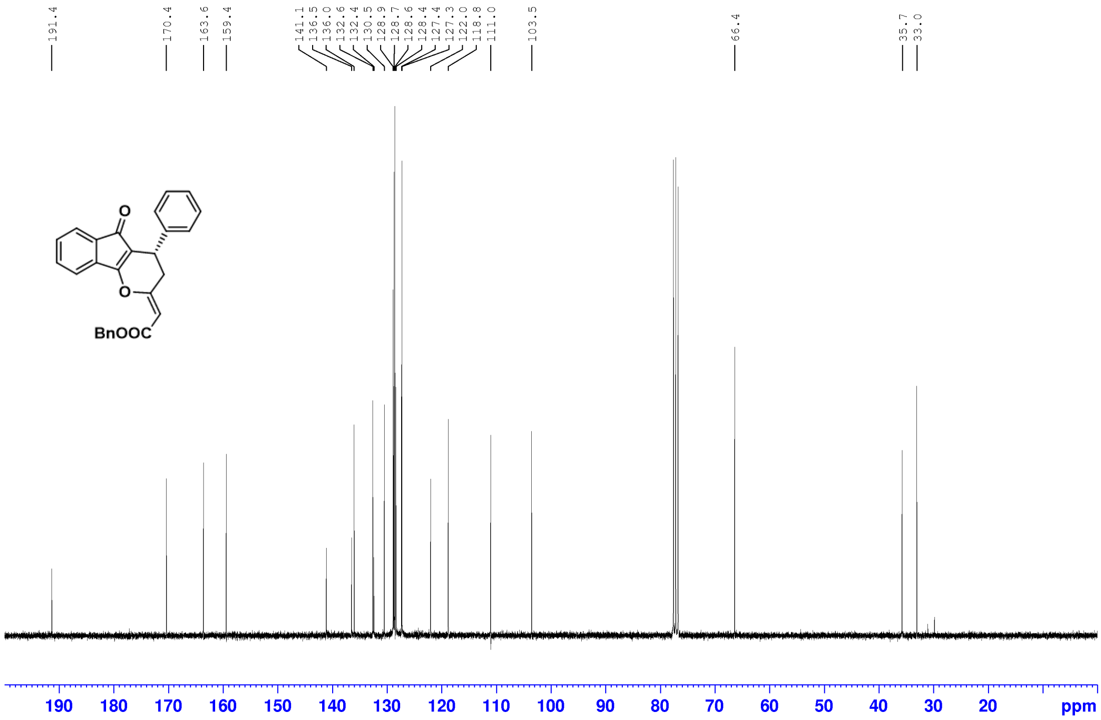

NMR of **3d**

^{1}H-NMR (300 MHz, $CDCl_3$, 298 K)

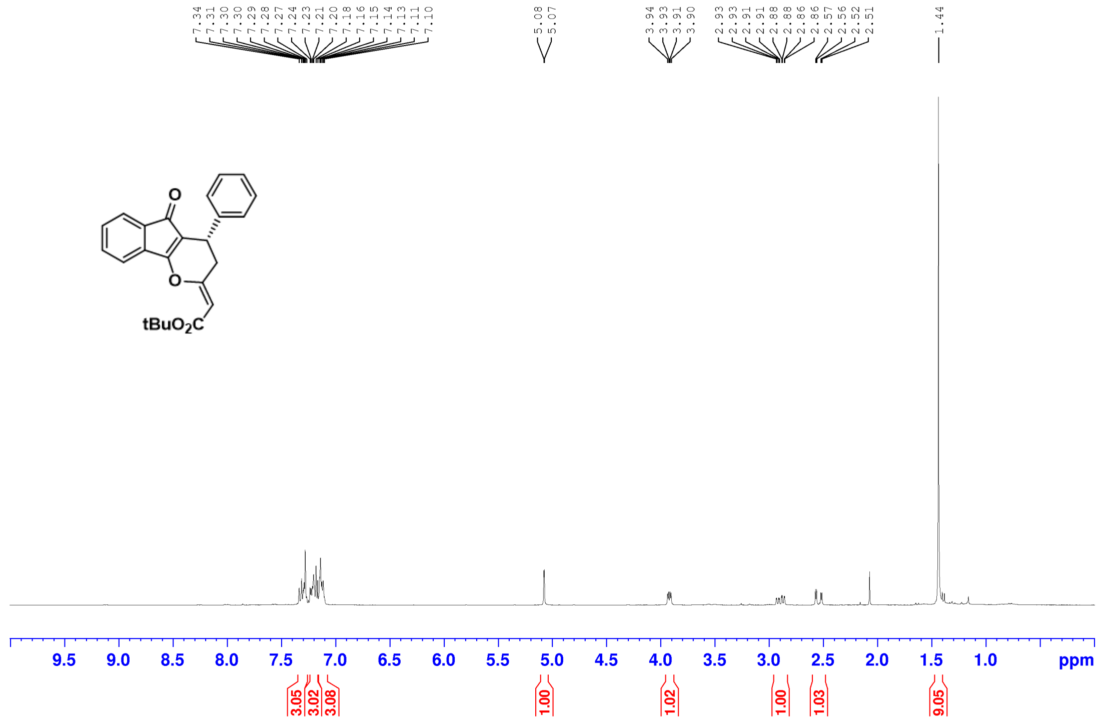

^{13}C-NMR (125 MHz, $CDCl_3$, 298 K)

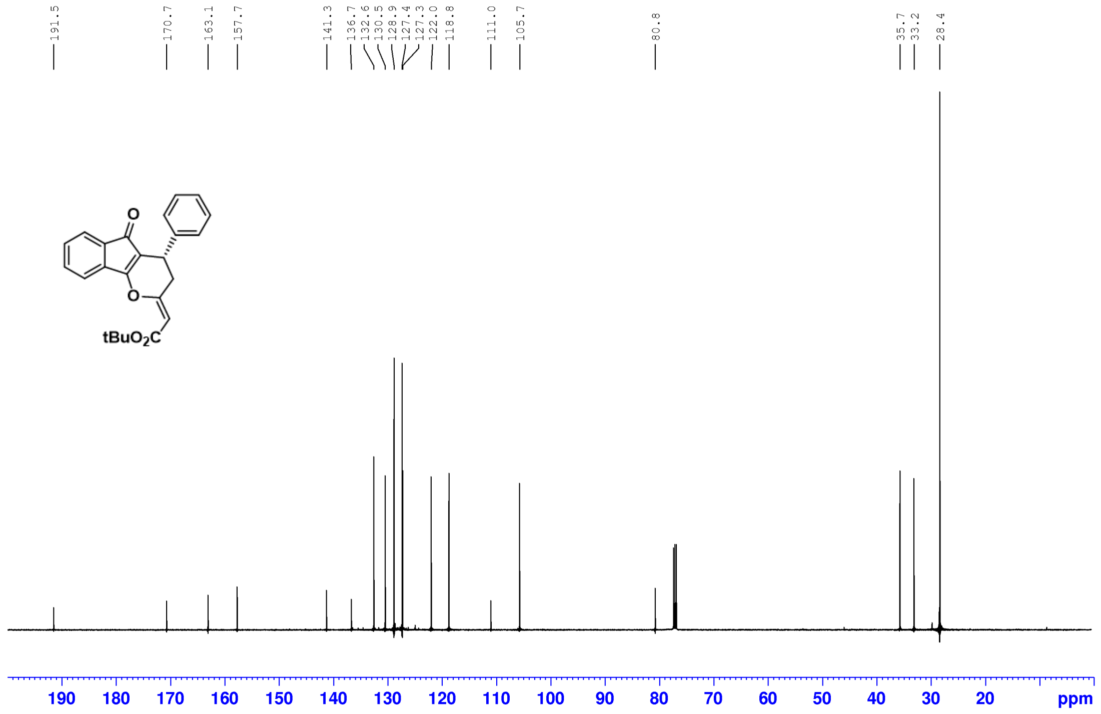

NMR of **3e**

^{1}H-NMR (300 MHz, $CDCl_3$, 298 K)

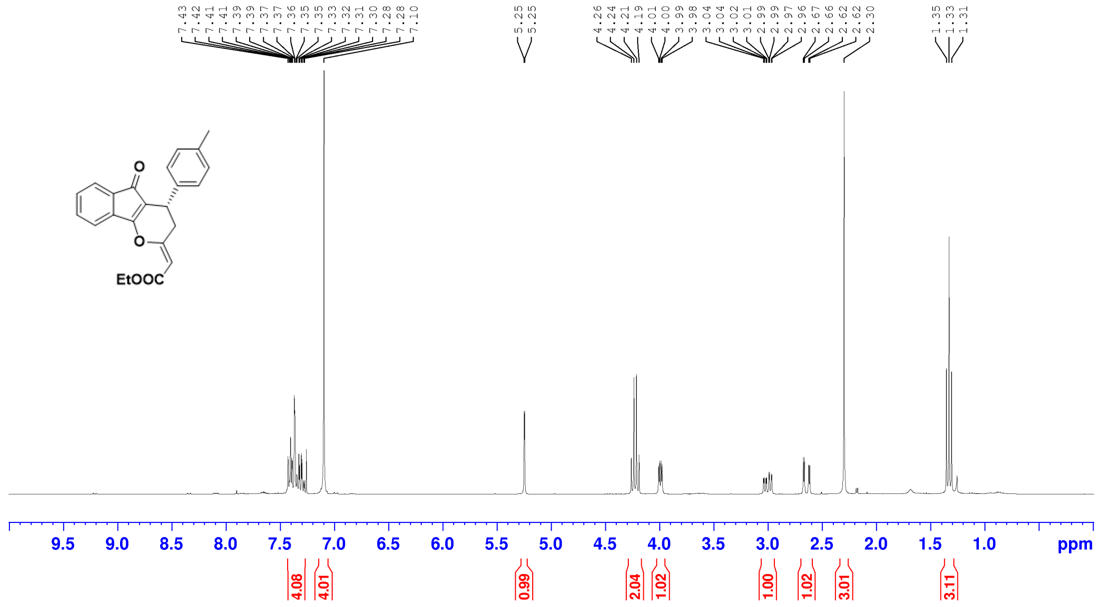

^{13}C-NMR (75 MHz, $CDCl_3$, 298 K)

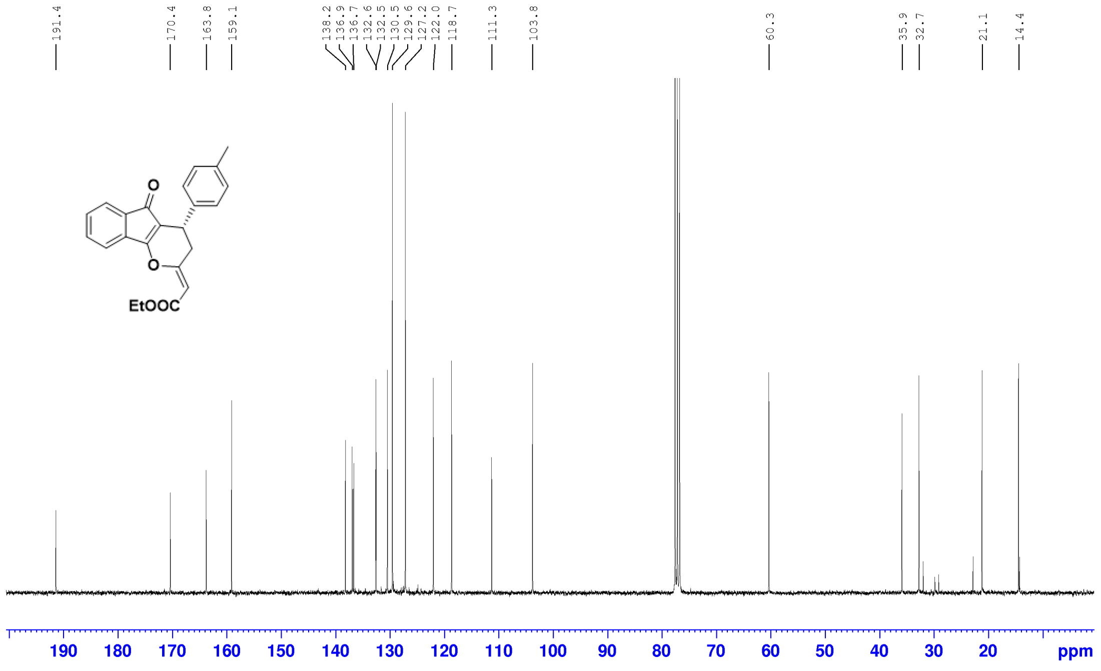

NMR of **3f**

^{1}H-NMR (300 MHz, $CDCl_3$, 298 K)

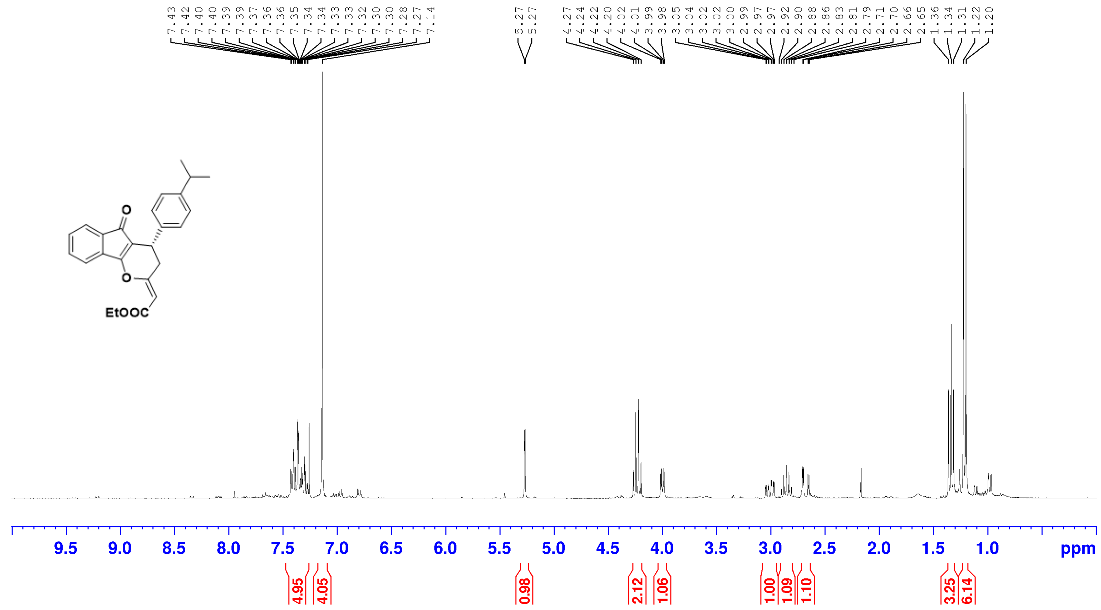

^{13}C-NMR (75 MHz, $CDCl_3$, 298 K)

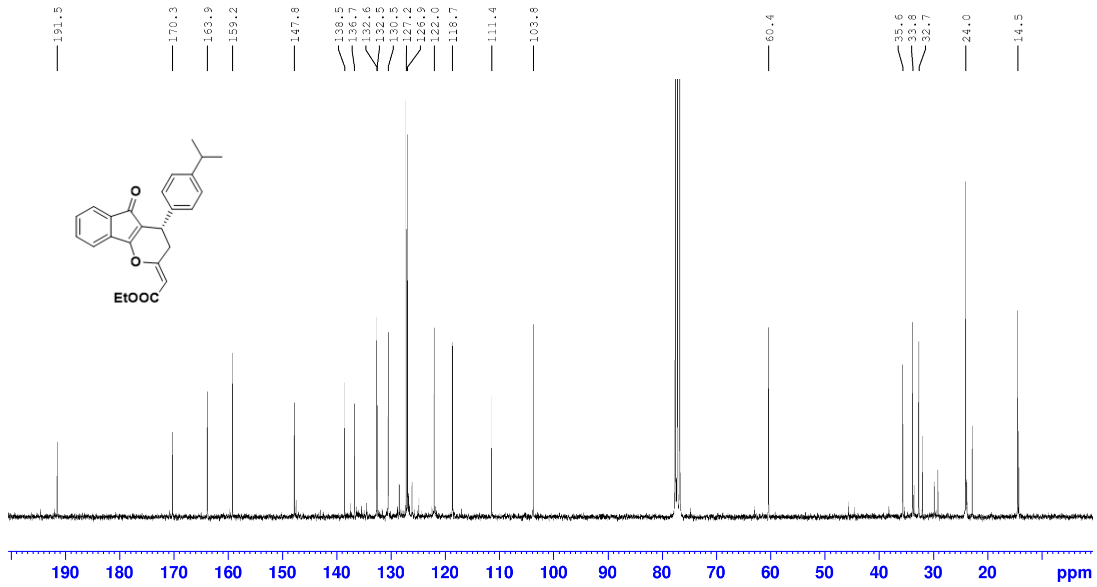

NMR of **3g**

^{1}H-NMR (300 MHz, $CDCl_3$, 298 K)

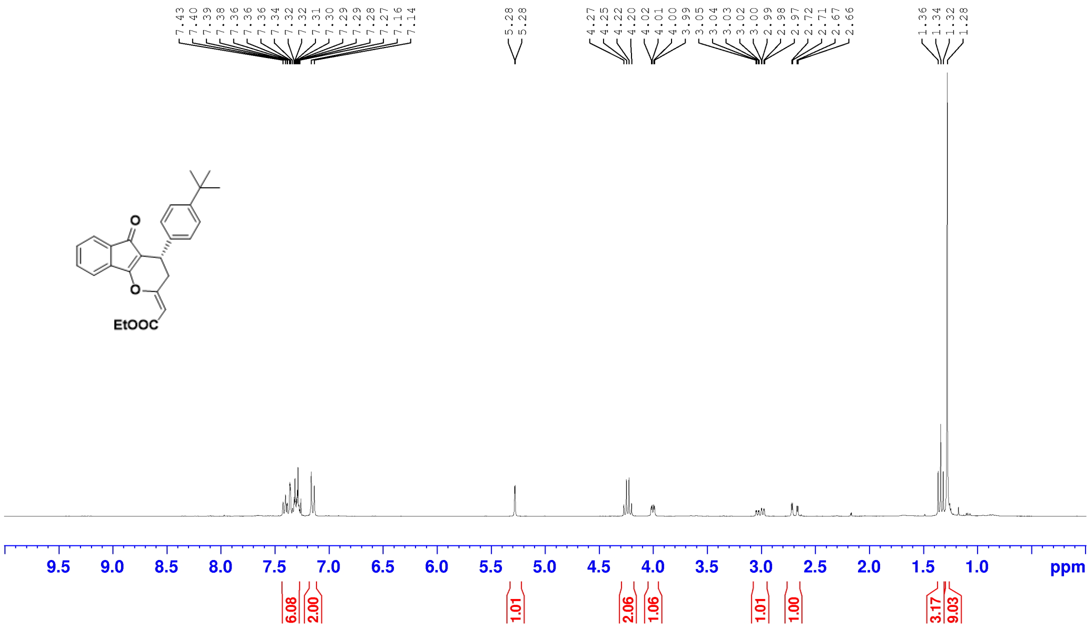

^{13}C-NMR (75 MHz, $CDCl_3$, 298 K)

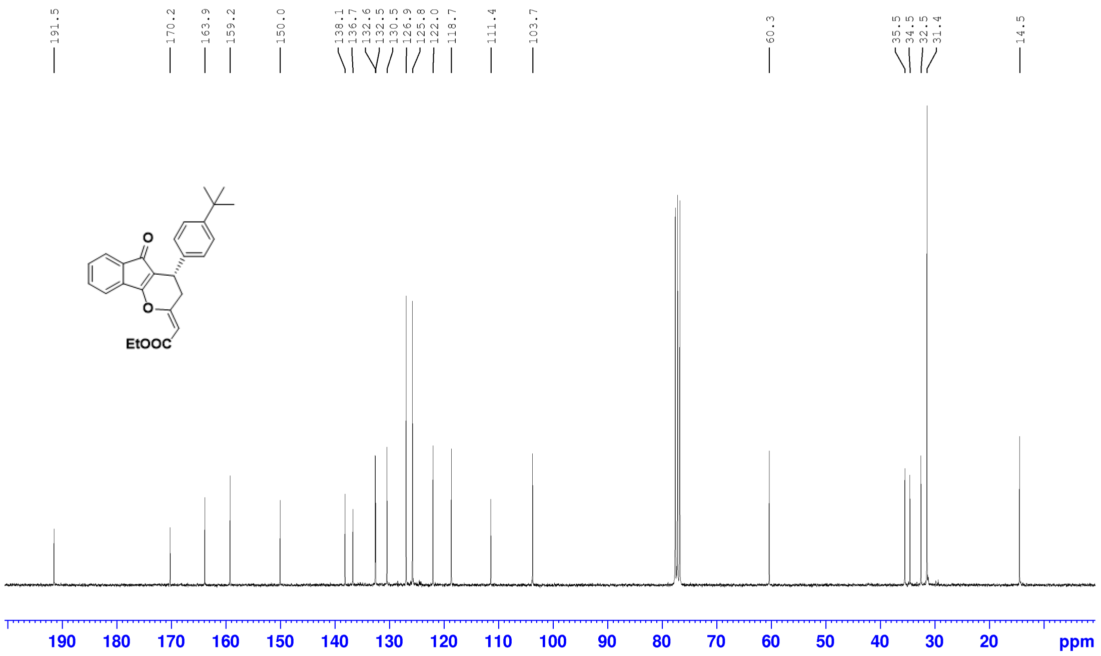

NMR of **3h**

^{1}H-NMR (300 MHz, $CDCl_3$, 298 K)

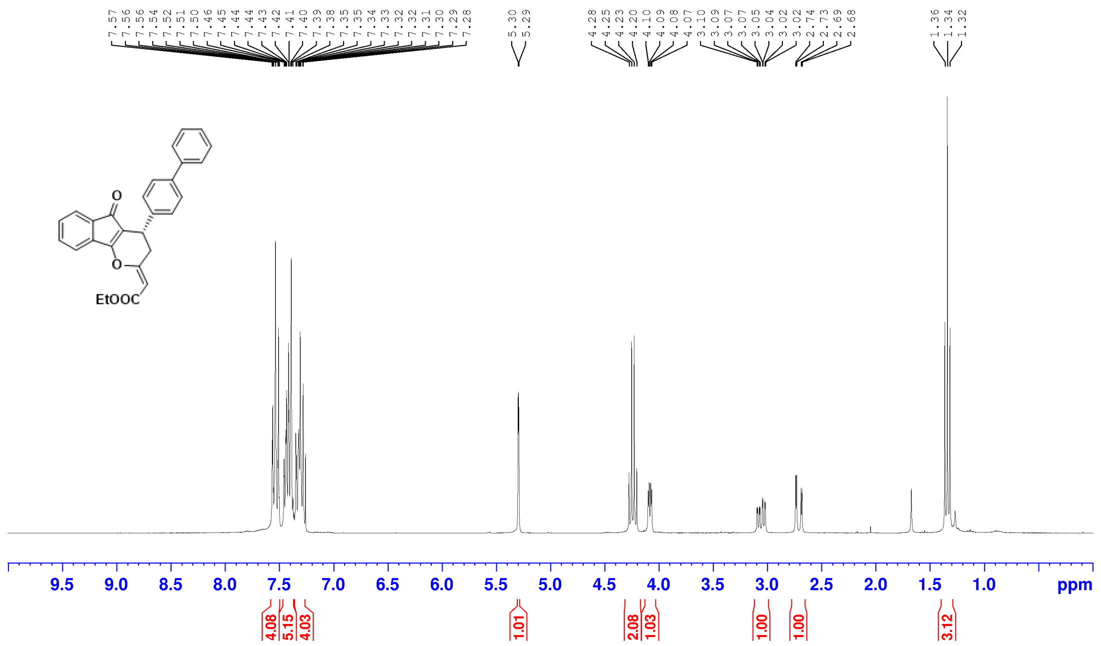

^{13}C-NMR (75 MHz, $CDCl_3$, 298 K)

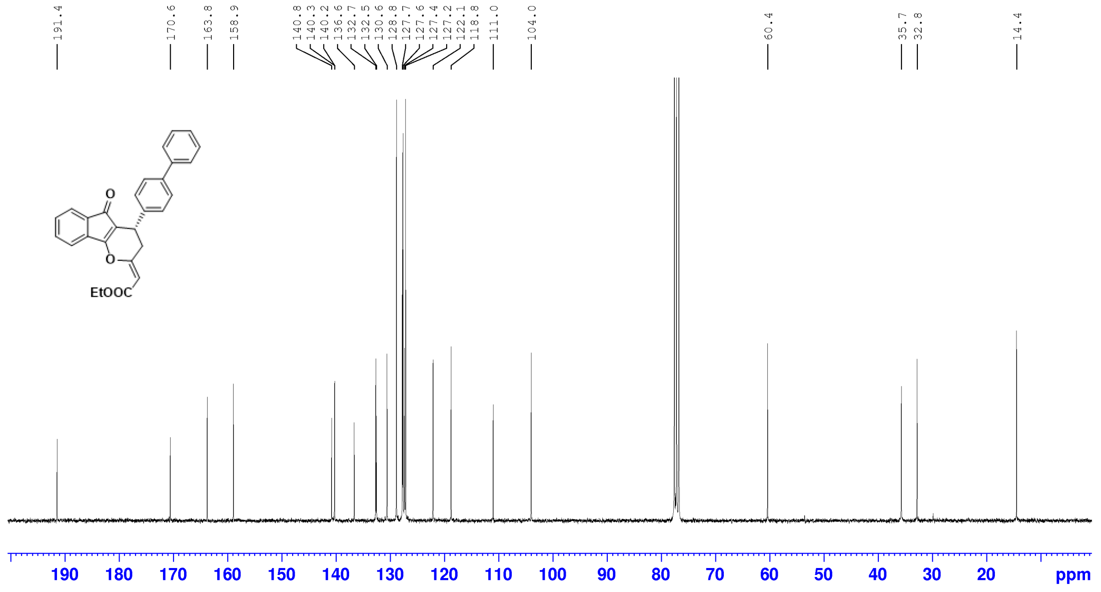

NMR of **3i**

^{1}H-NMR (300 MHz, $CDCl_3$, 298 K)

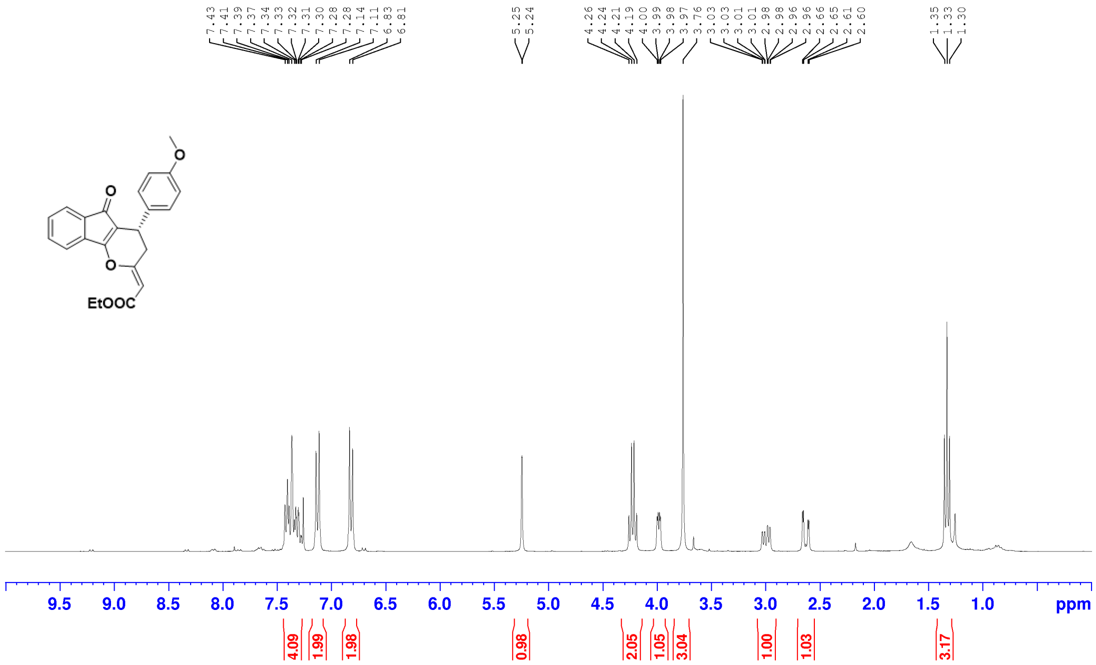

^{13}C-NMR (75 MHz, $CDCl_3$, 298 K)

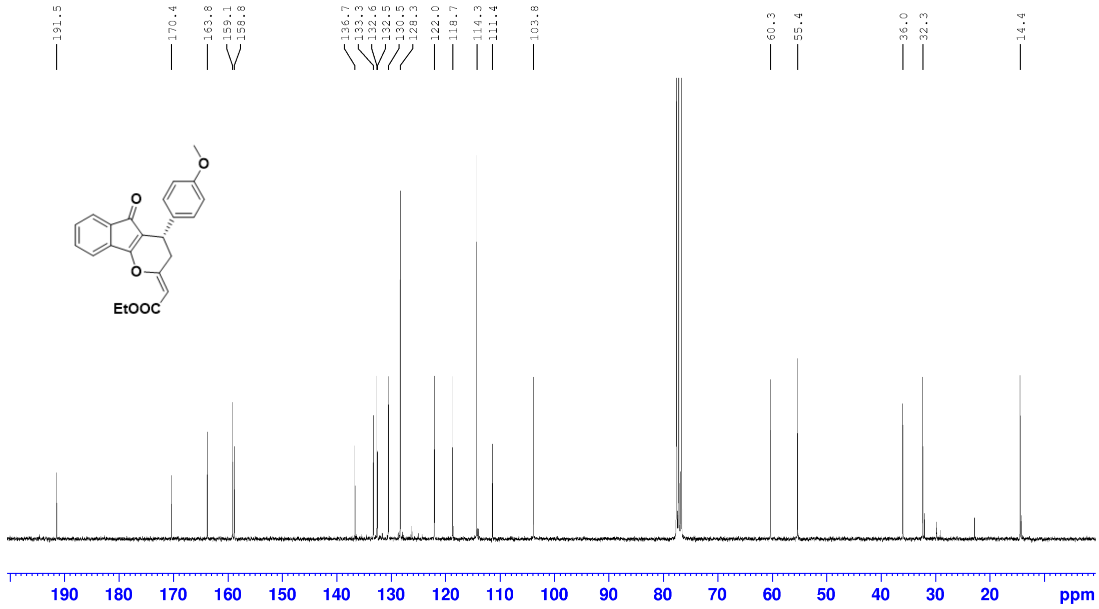

NMR of **3j**

^{1}H-NMR (300 MHz, $CDCl_3$, 298 K)

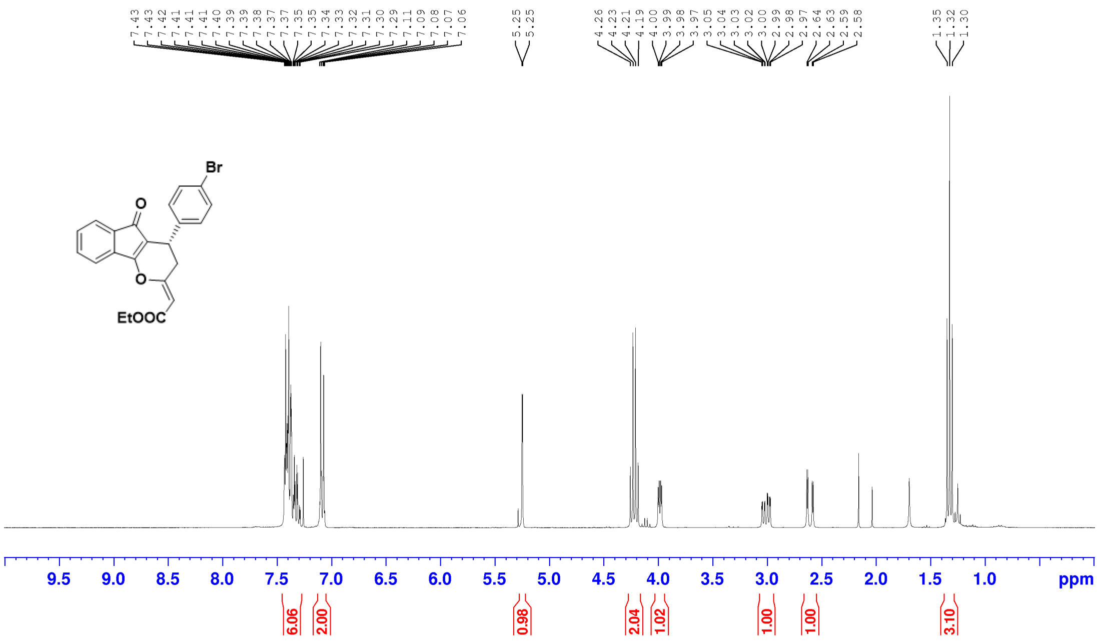

^{13}C-NMR (75 MHz, $CDCl_3$, 298 K)

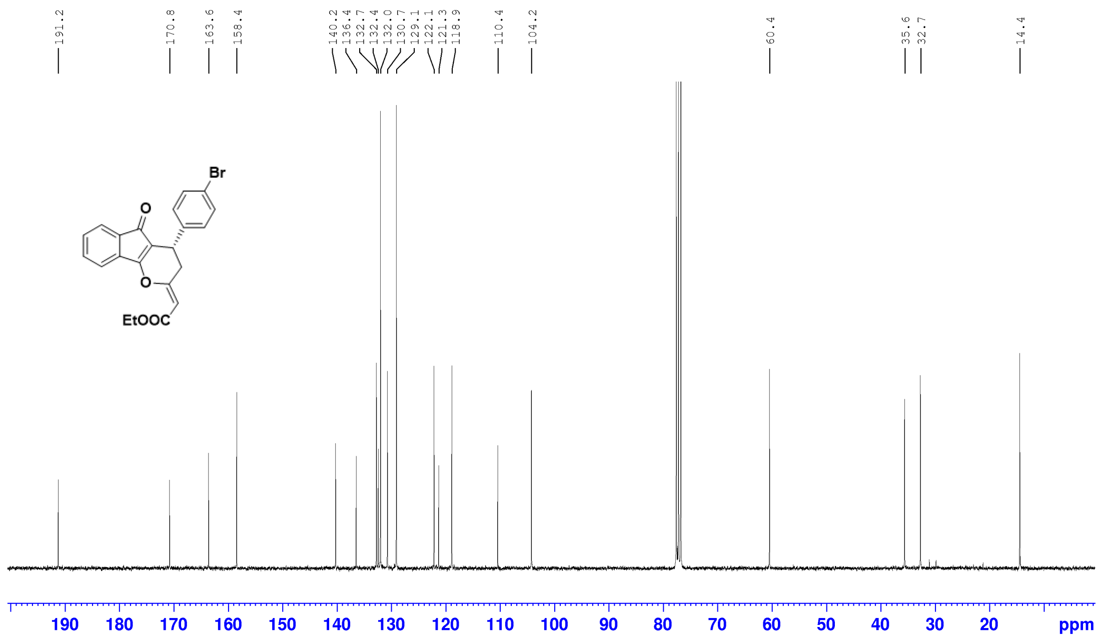

NMR of **3k**

^{1}H-NMR (300 MHz, $CDCl_3$, 298 K)

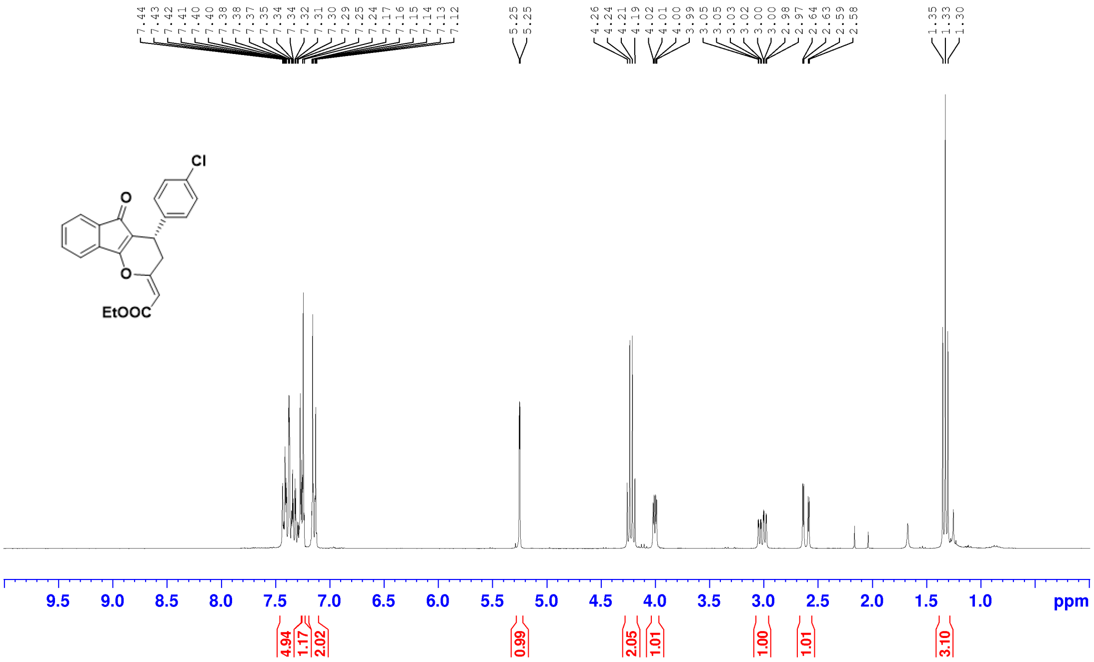

^{13}C-NMR (75 MHz, $CDCl_3$, 298 K)

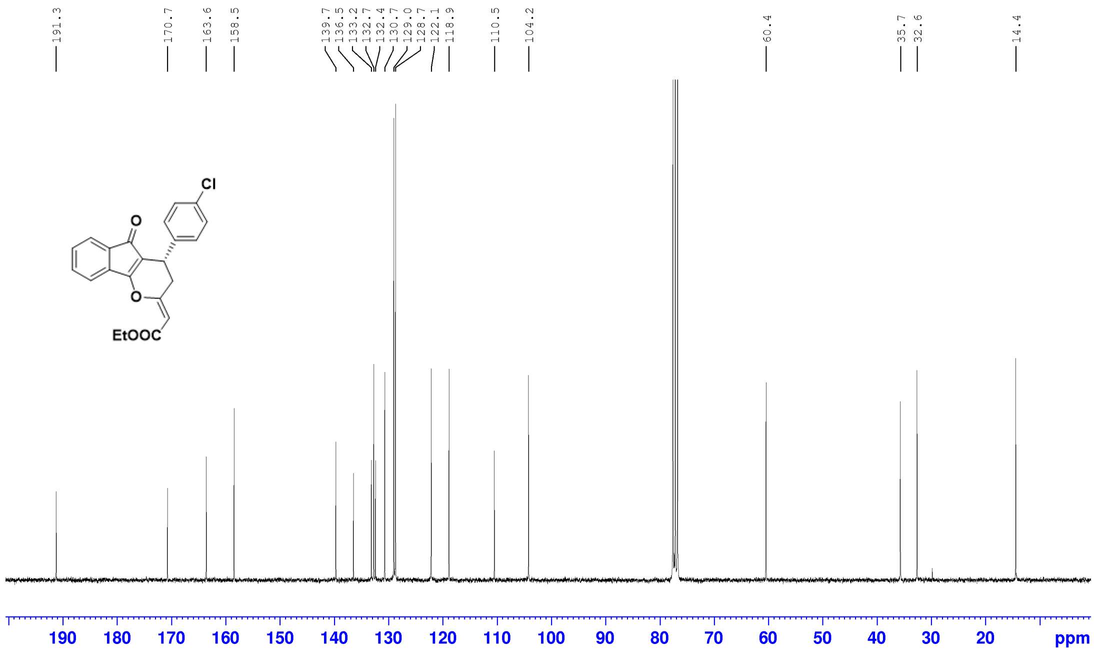

NMR of **3l**

^{1}H-NMR (300 MHz, $CDCl_3$, 298 K)

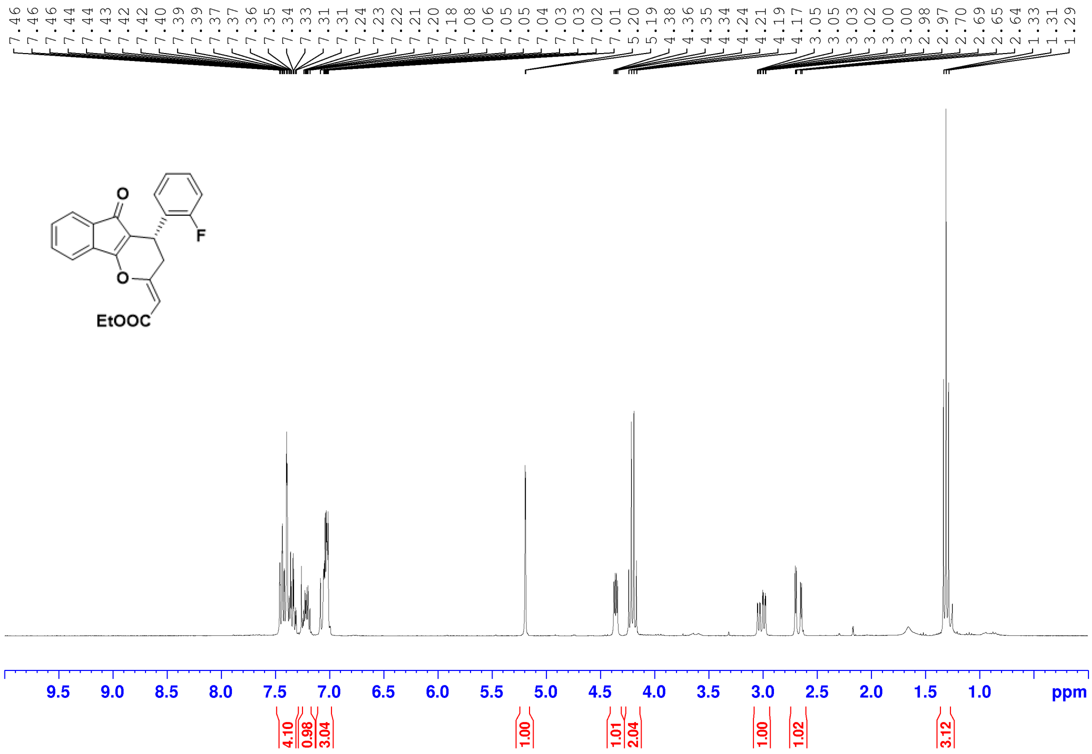

^{19}F-NMR (282 MHz, $CDCl_3$ 298 K)

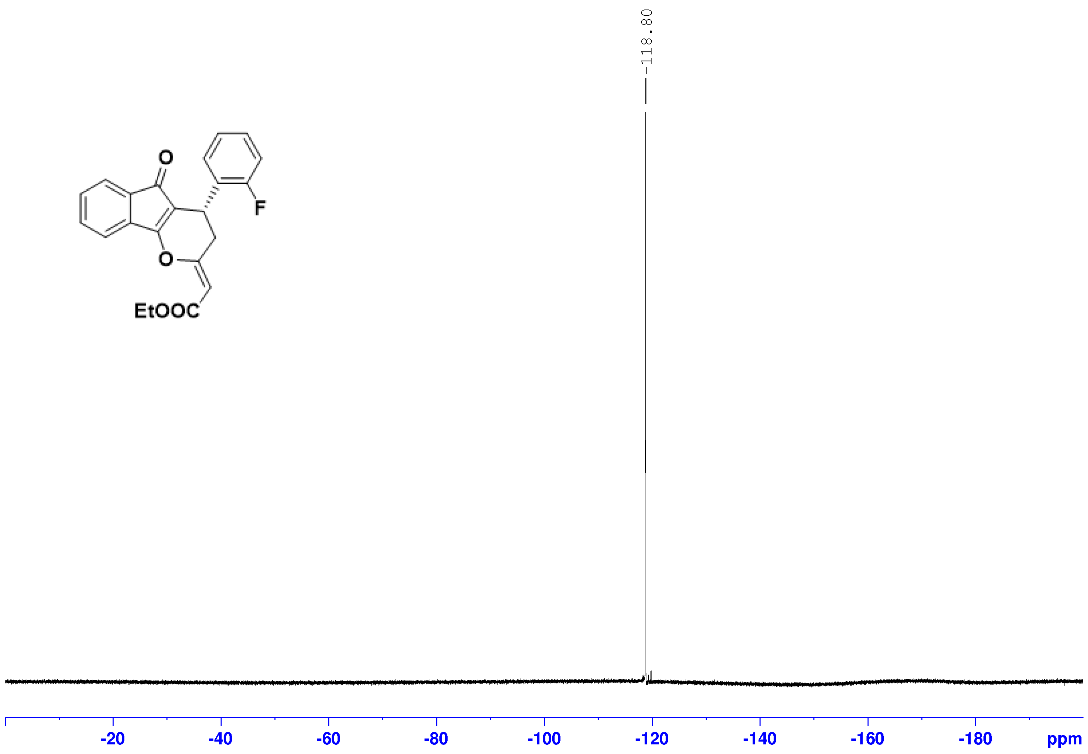

^{13}C-NMR (75 MHz, $CDCl_3$, 298 K)

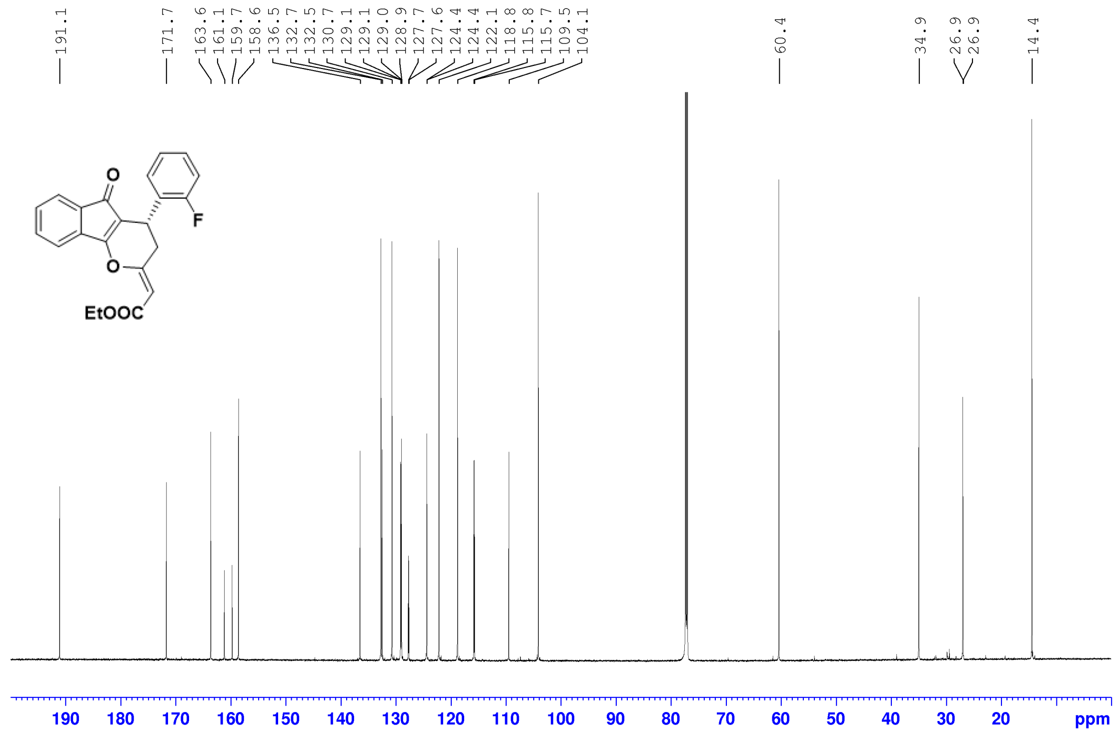

NMR of **3m**

^{1}H-NMR (300 MHz, $CDCl_3$, 298 K)

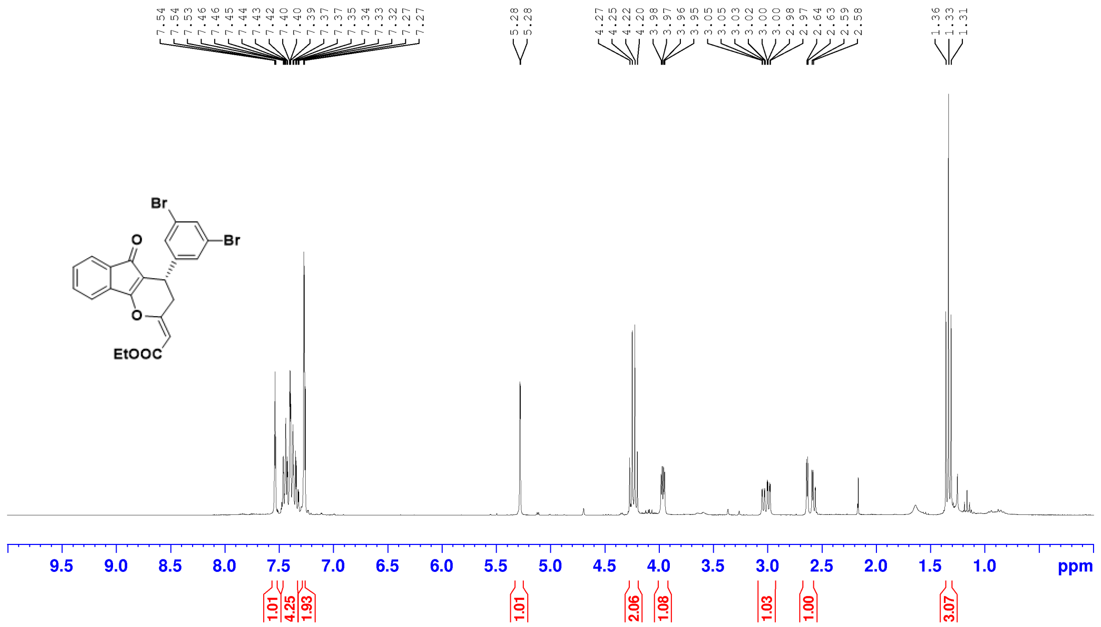

^{13}C-NMR (75 MHz, $CDCl_3$, 298 K)

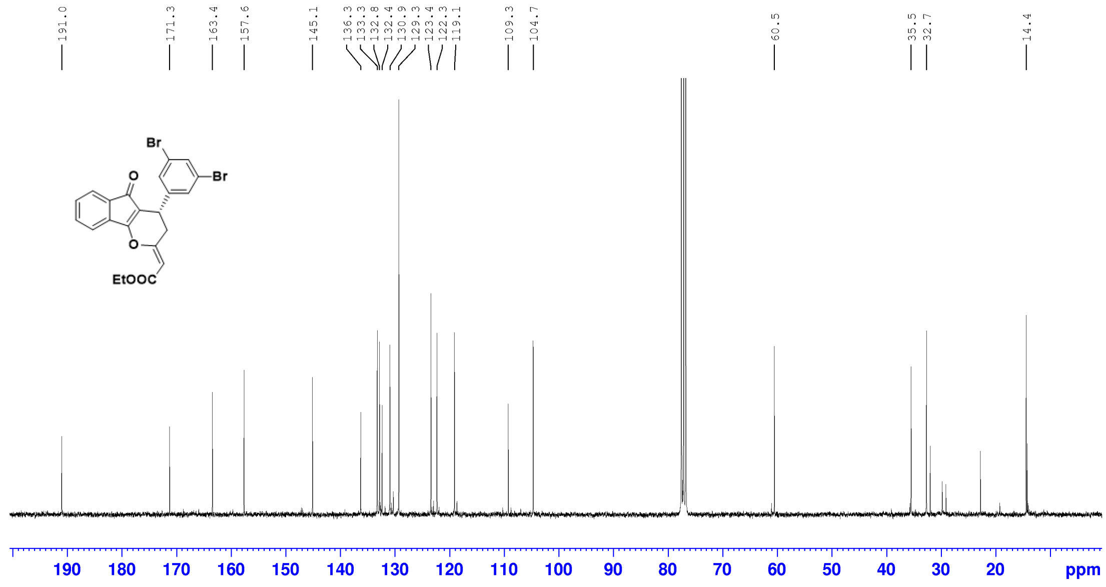

NMR of **3n**

^{1}H-NMR (300 MHz, $CDCl_3$, 298 K)

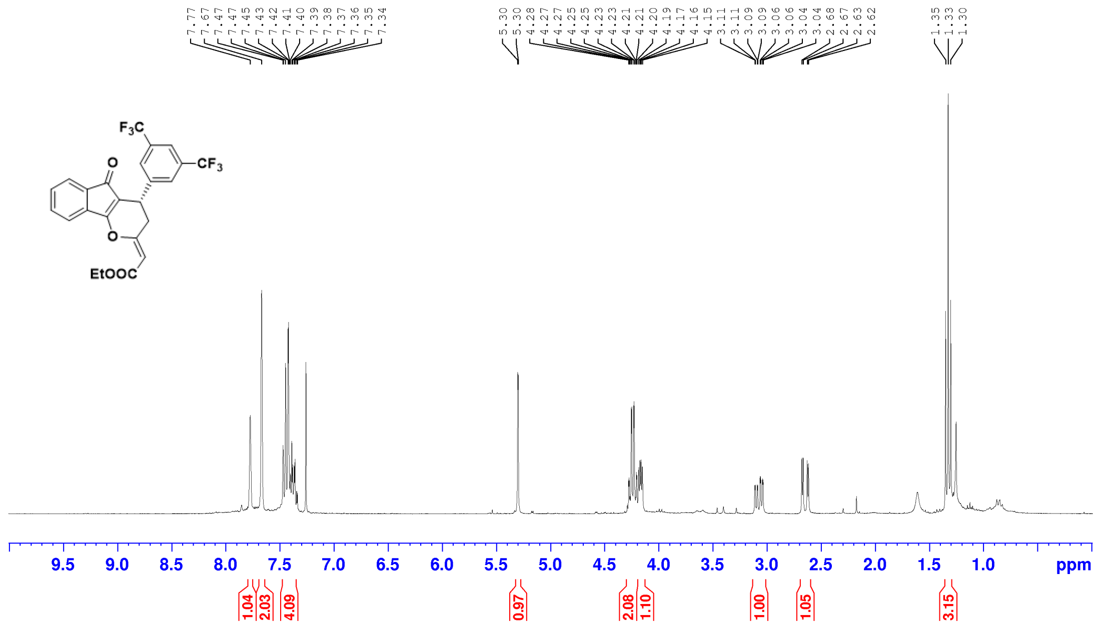

^{19}F-NMR (282 MHz, CDCl3 298 K)

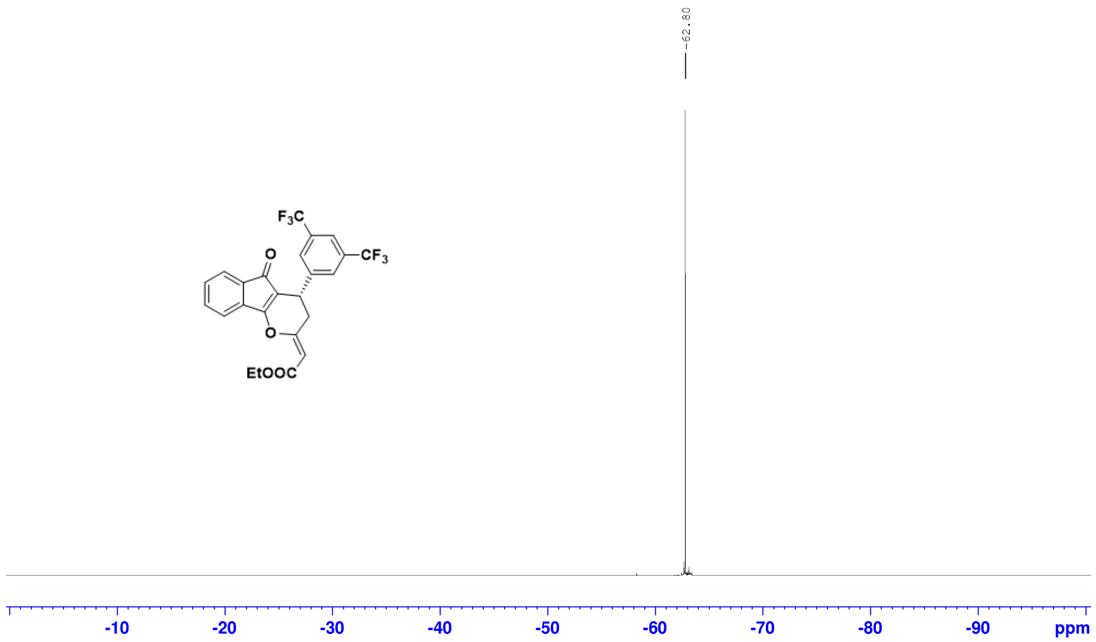

^{13}C-NMR (75 MHz, CDCl3, 298 K)

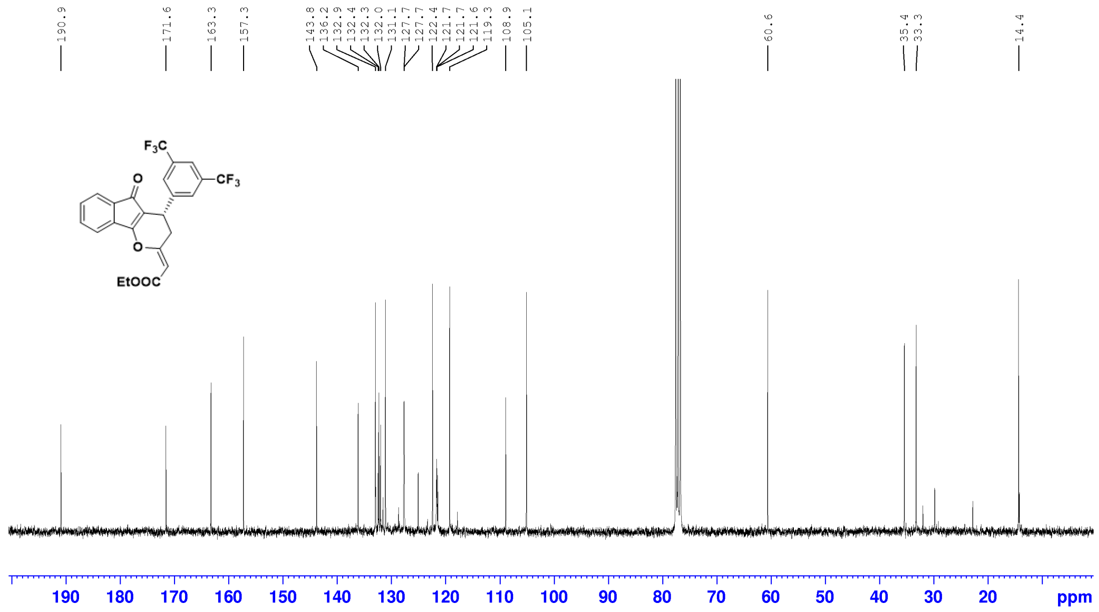

NMR of **3o**

^{1}H-NMR (300 MHz, $CDCl_3$, 298 K)

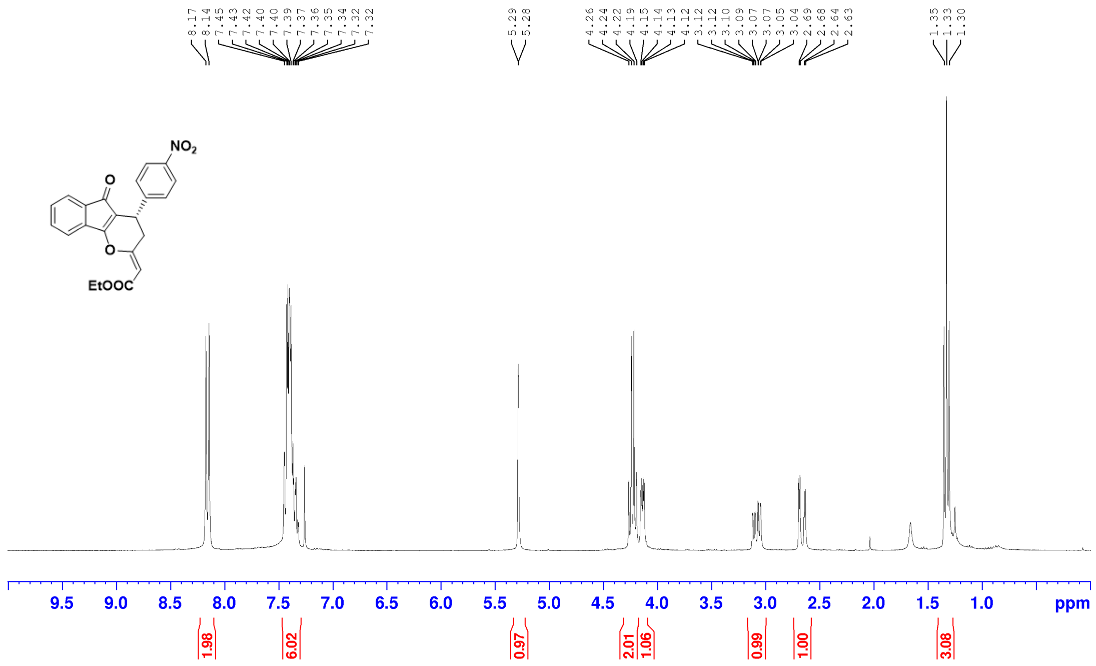

^{13}C-NMR (75 MHz, $CDCl_3$, 298 K)

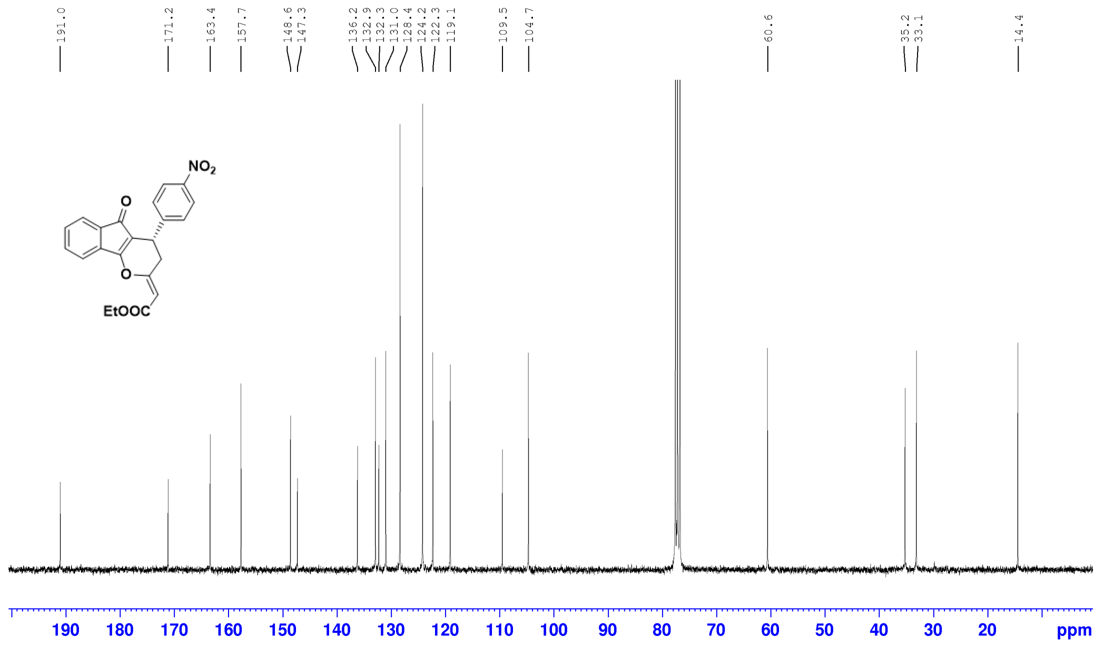

NMR of **3p**

^{1}H-NMR (300 MHz, $CDCl_3$, 298 K)

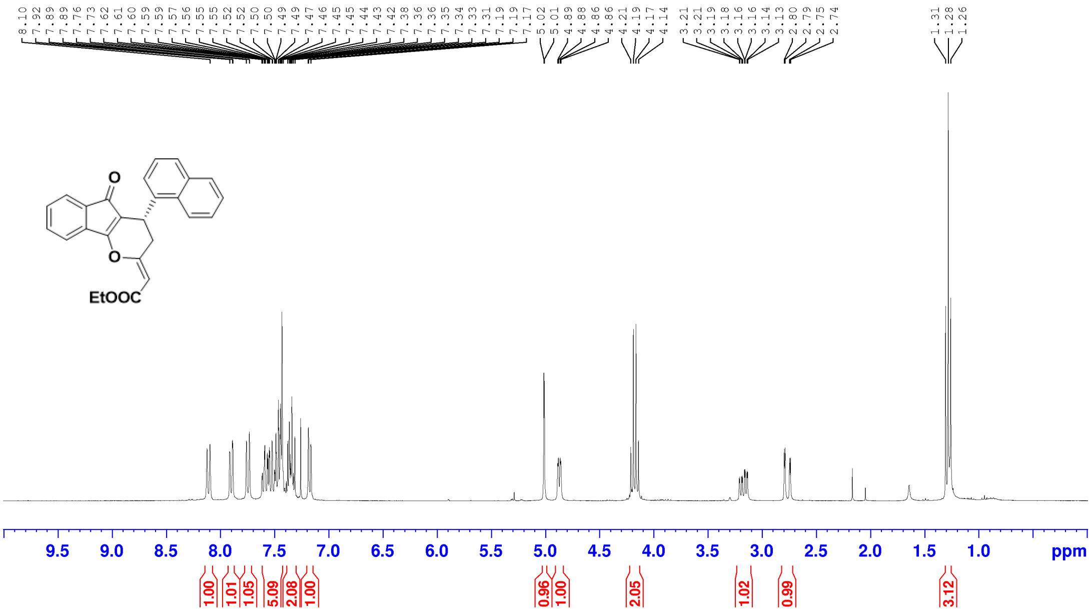

^{13}C-NMR (75 MHz, $CDCl_3$, 298 K)

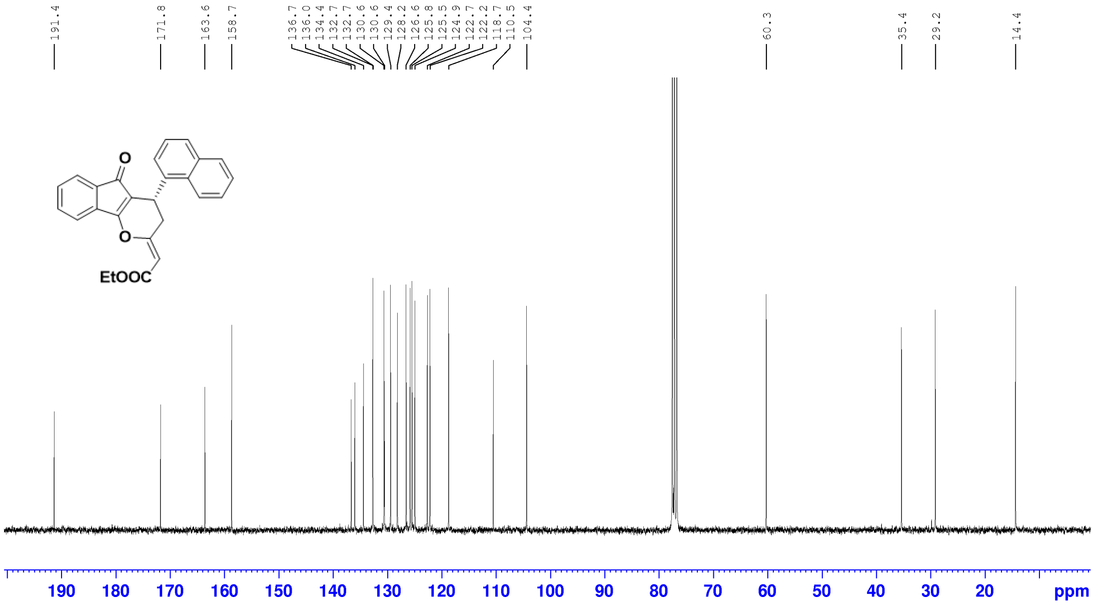

NMR of **3q**

^{1}H-NMR (700 MHz, $CDCl_3$, 298 K)

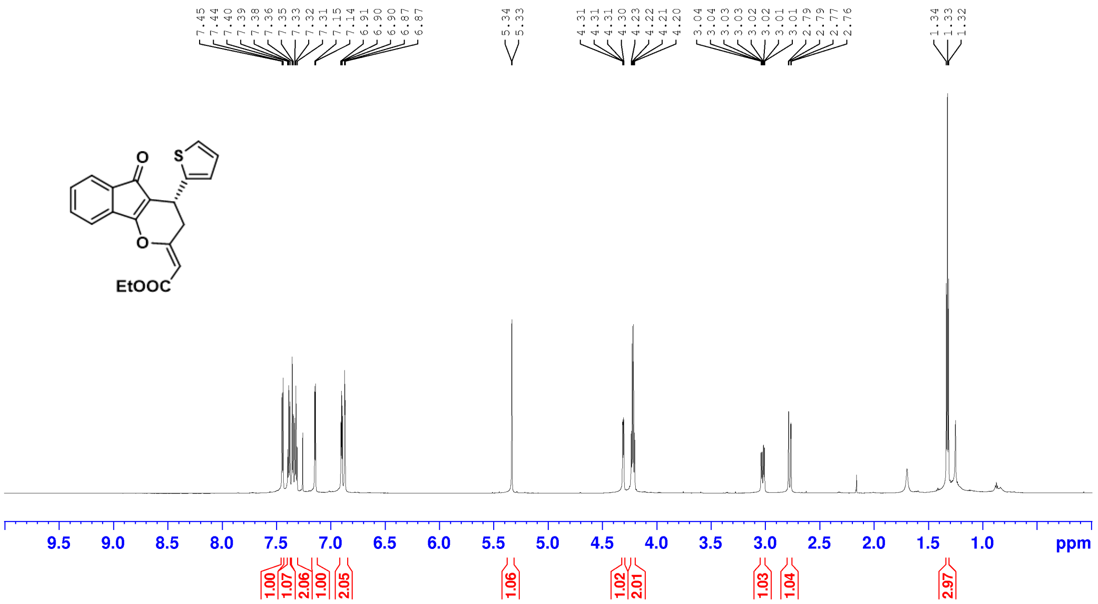

^{13}C-NMR (175 MHz, $CDCl_3$, 298 K)

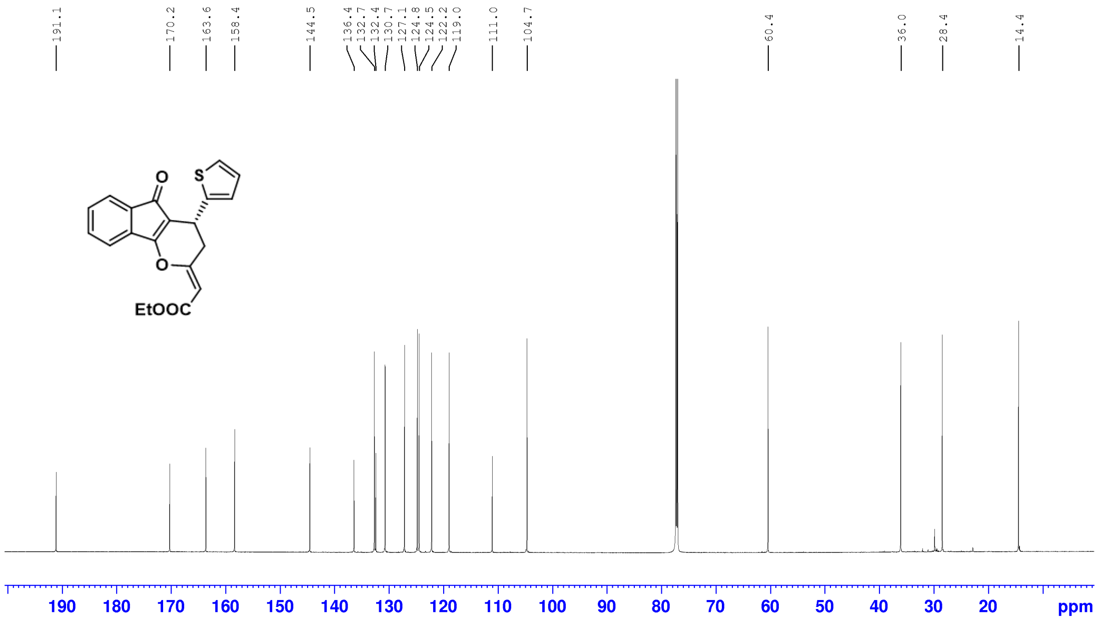

NMR of **3r**

^{1}H-NMR (700 MHz, $CDCl_3$, 298 K)

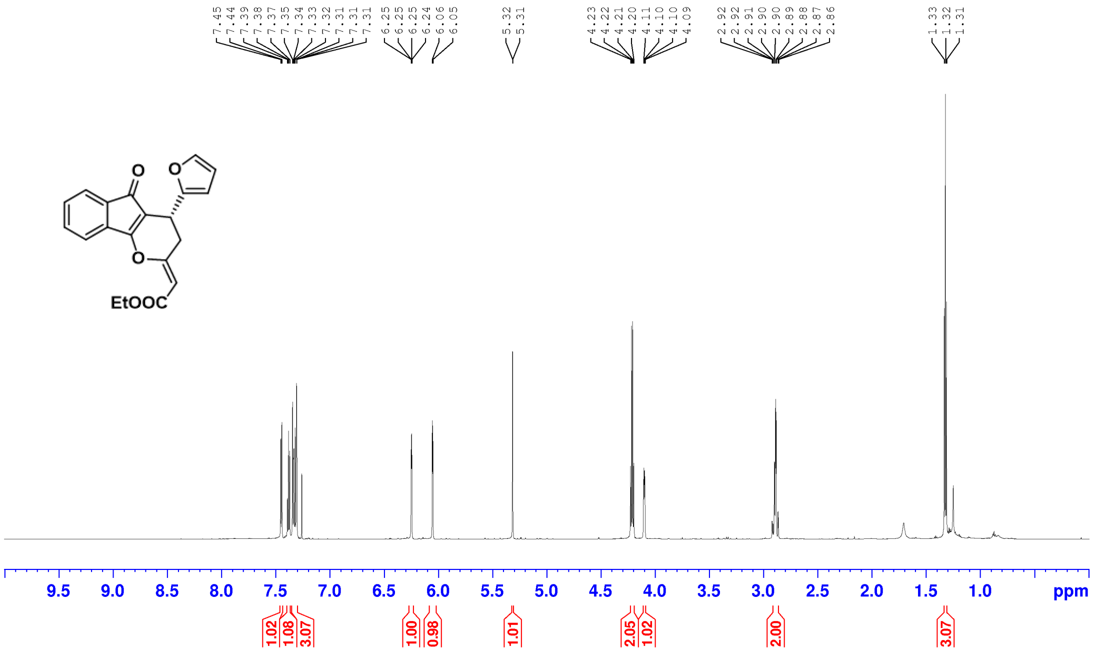

^{13}C-NMR (175 MHz, $CDCl_3$, 298 K)

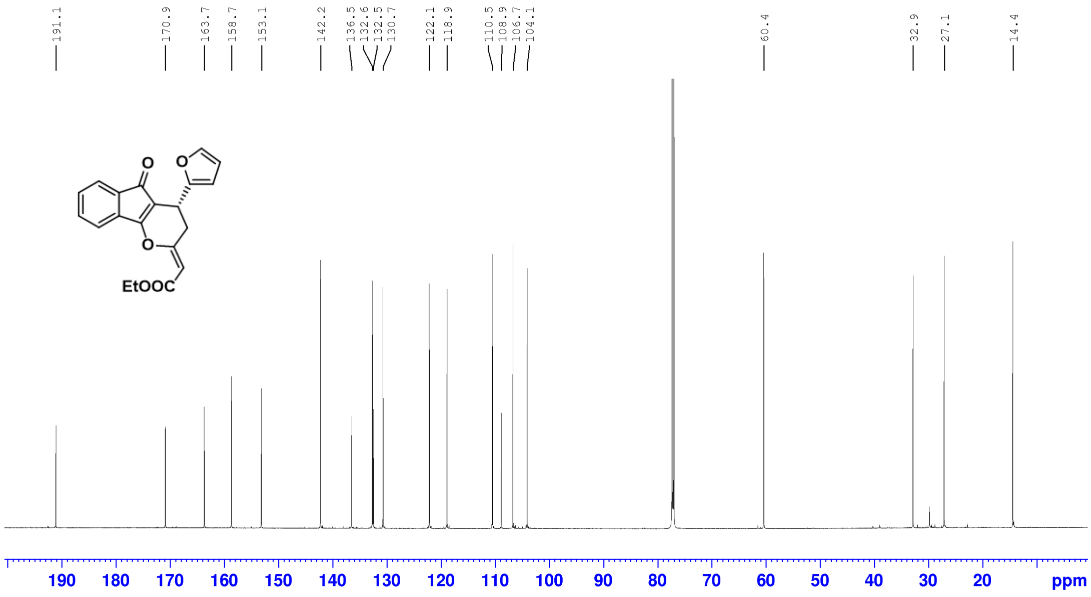

NMR of **3s**$_{major}$

^{1}H-NMR (300 MHz, $CDCl_3$, 298 K)

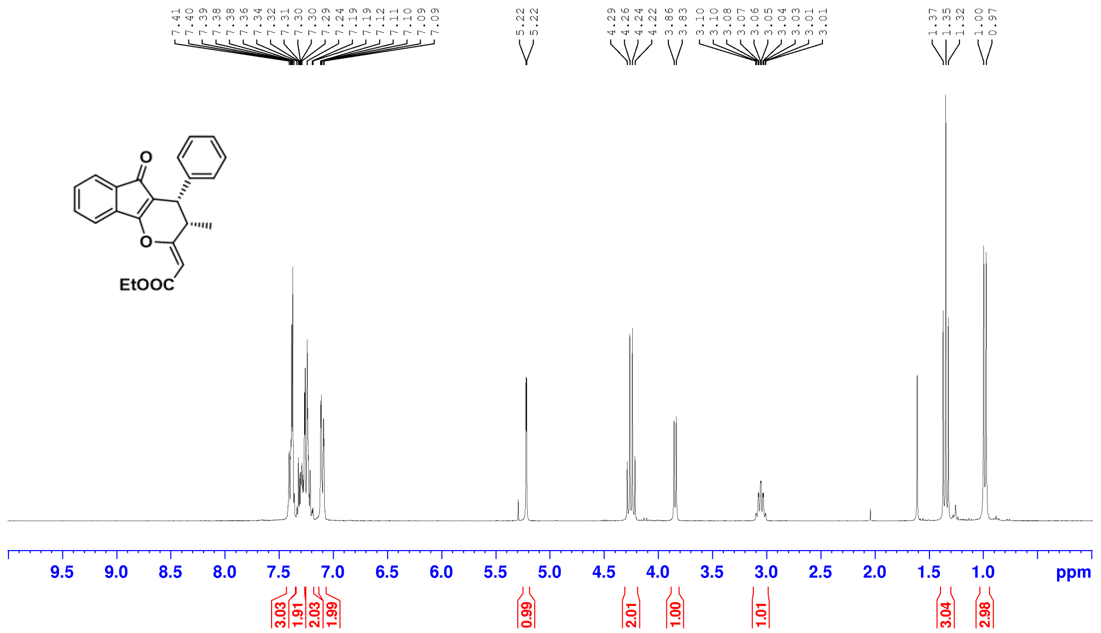

^{13}C-NMR (75 MHz, $CDCl_3$, 298 K)

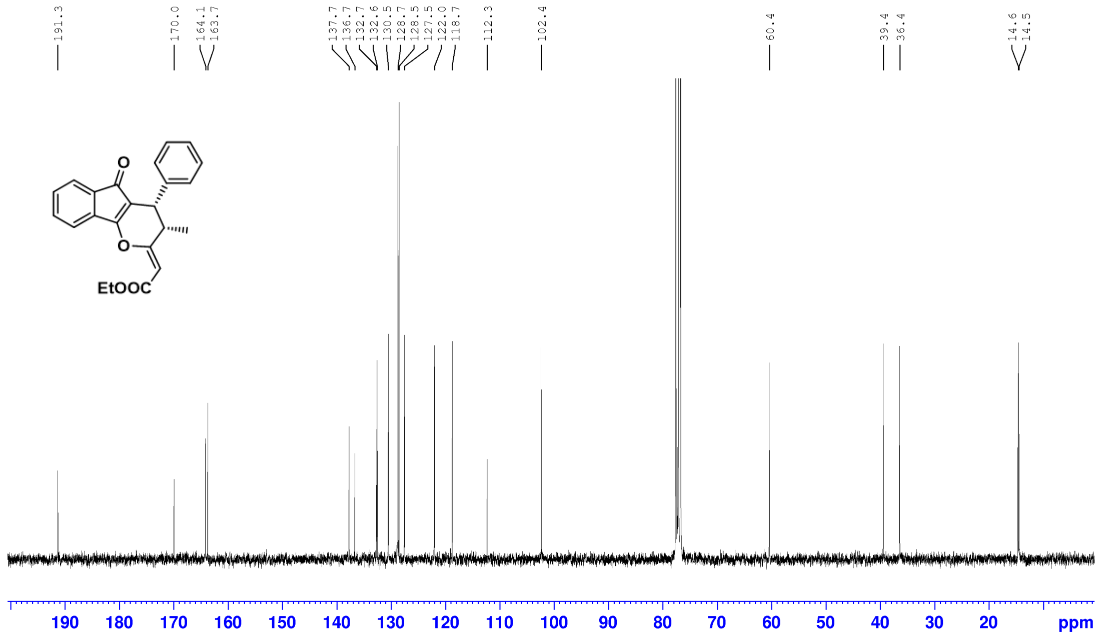

NMR of **3s**$_{minor}$

^{1}H-NMR (300 MHz, $CDCl_3$, 298 K)

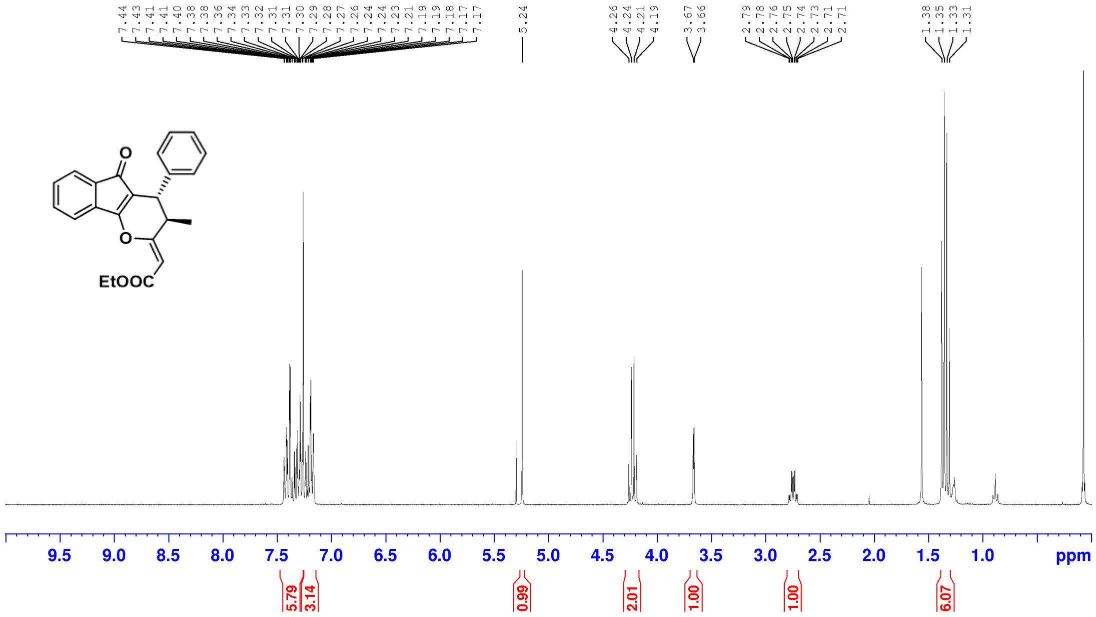

^{13}C-NMR (75 MHz, $CDCl_3$, 298 K)

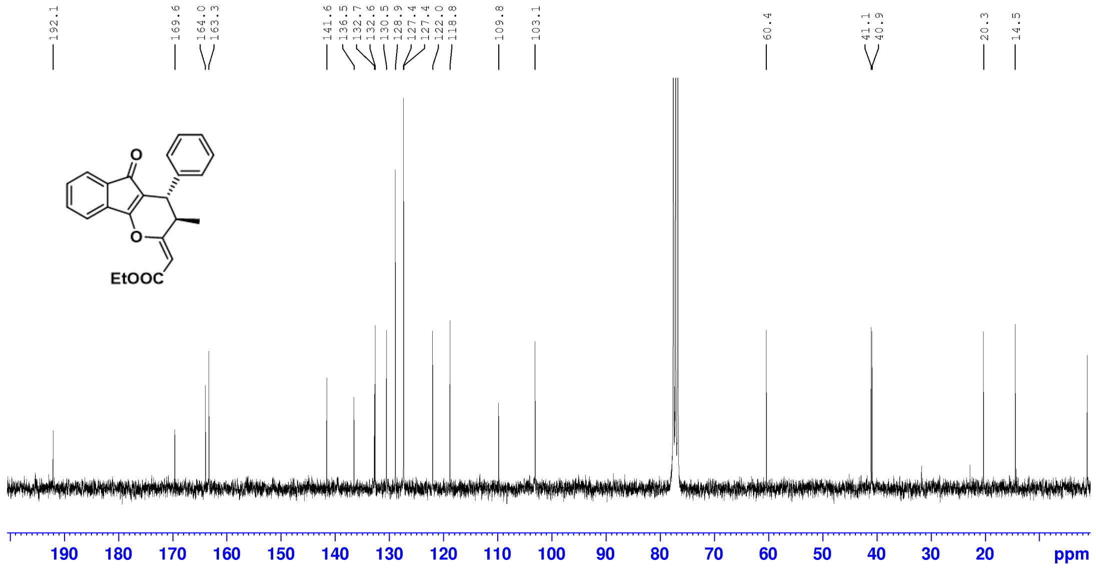

NMR of **3t**$_{\mathbf{major}}$

^{1}H-NMR (300 MHz, CDCl$_3$, 298 K)

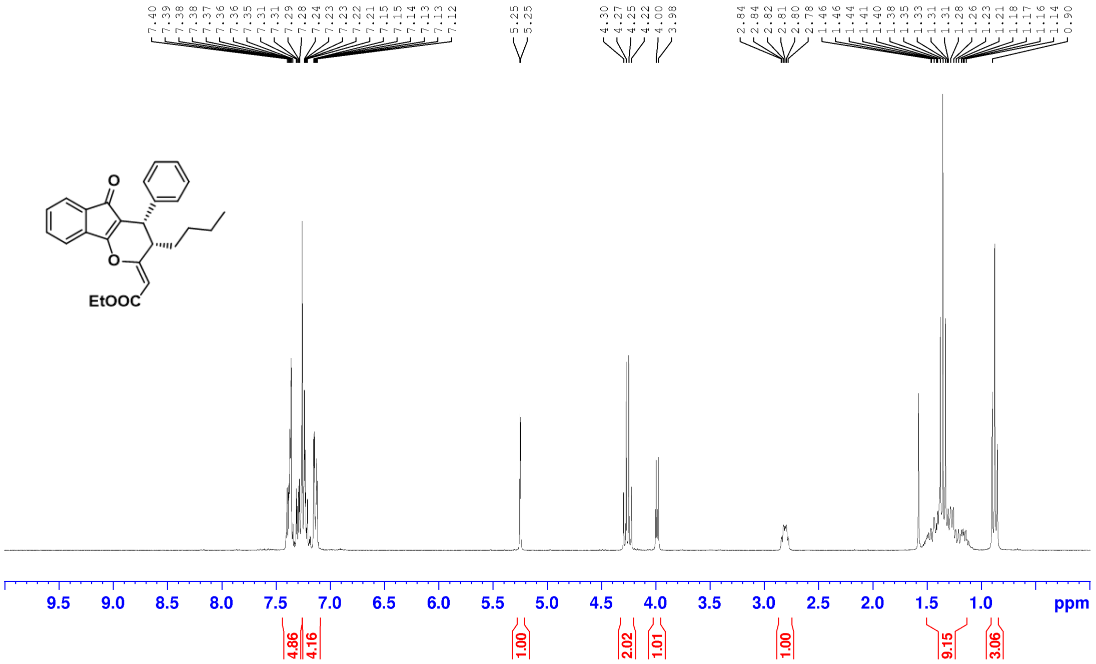

^{13}C-NMR (75 MHz, CDCl$_3$, 298 K)

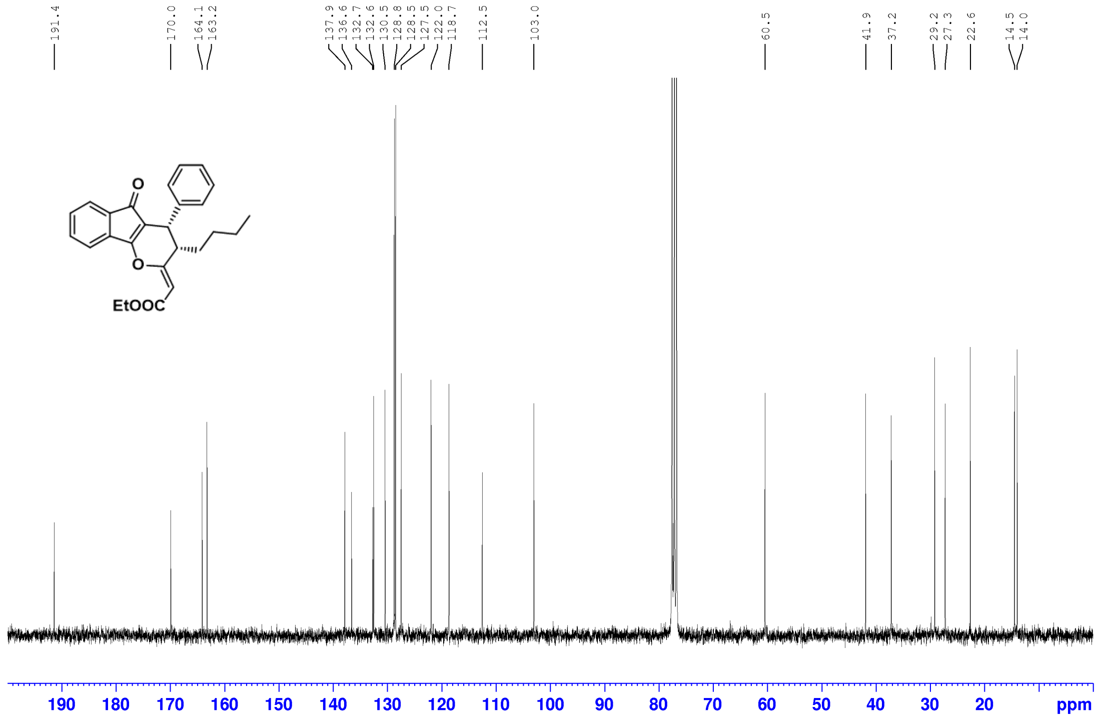

NMR of **3t**$_{minor}$

^{1}H-NMR (300 MHz, $CDCl_3$, 298 K)

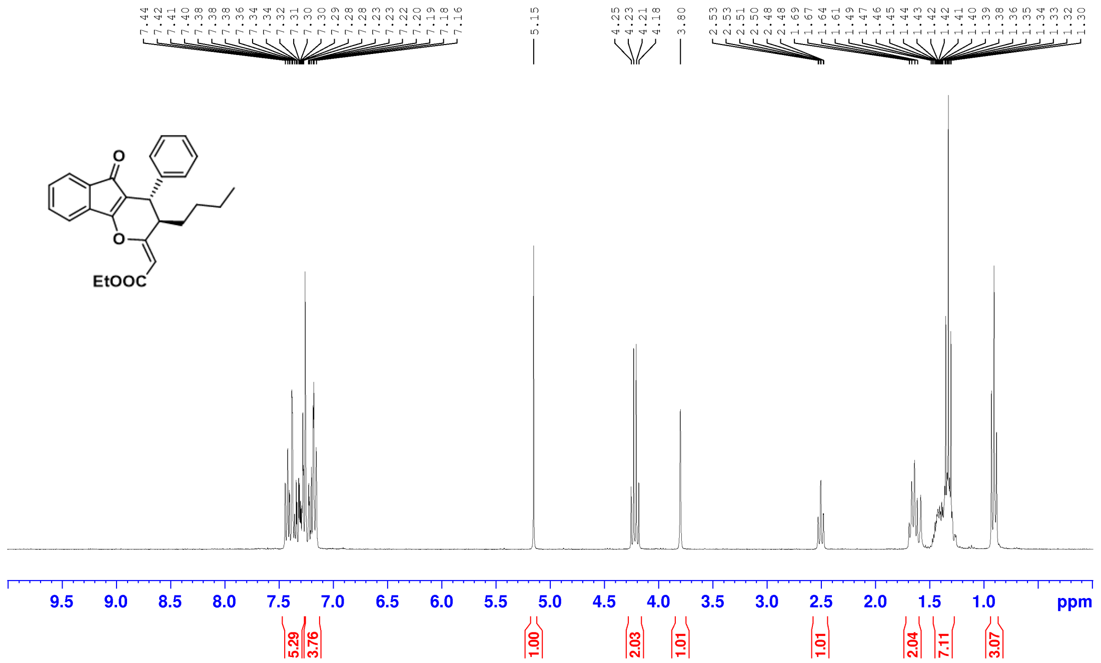

^{13}C-NMR (75 MHz, $CDCl_3$, 298 K)

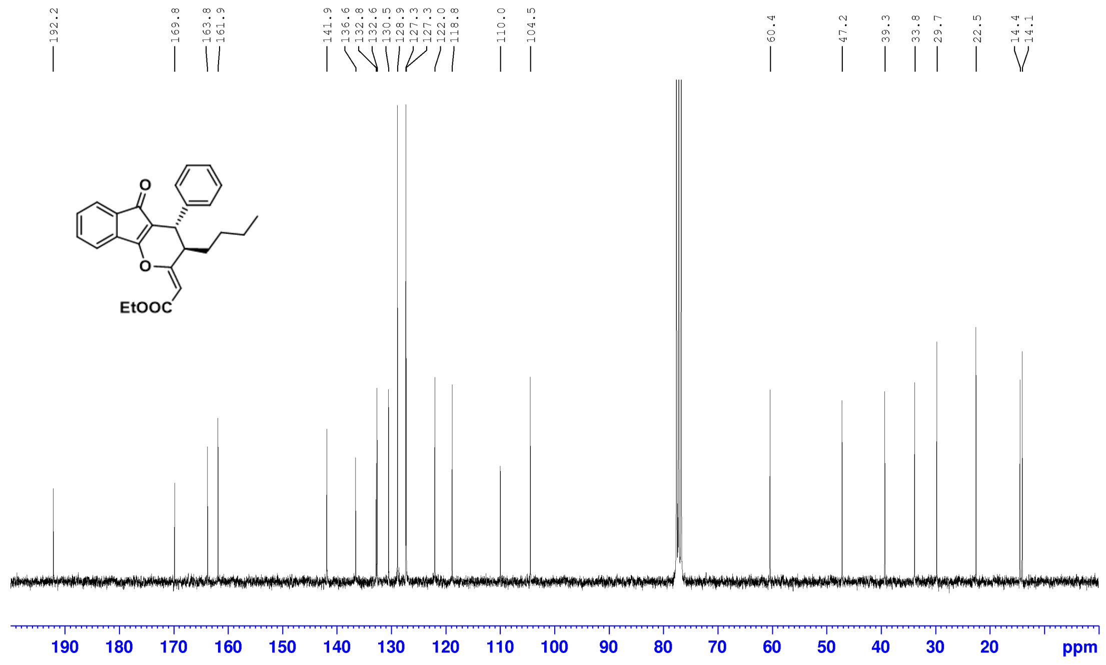

NMR of **3u**$_{major}$

^{1}H-NMR (300 MHz, $CDCl_3$, 298 K)

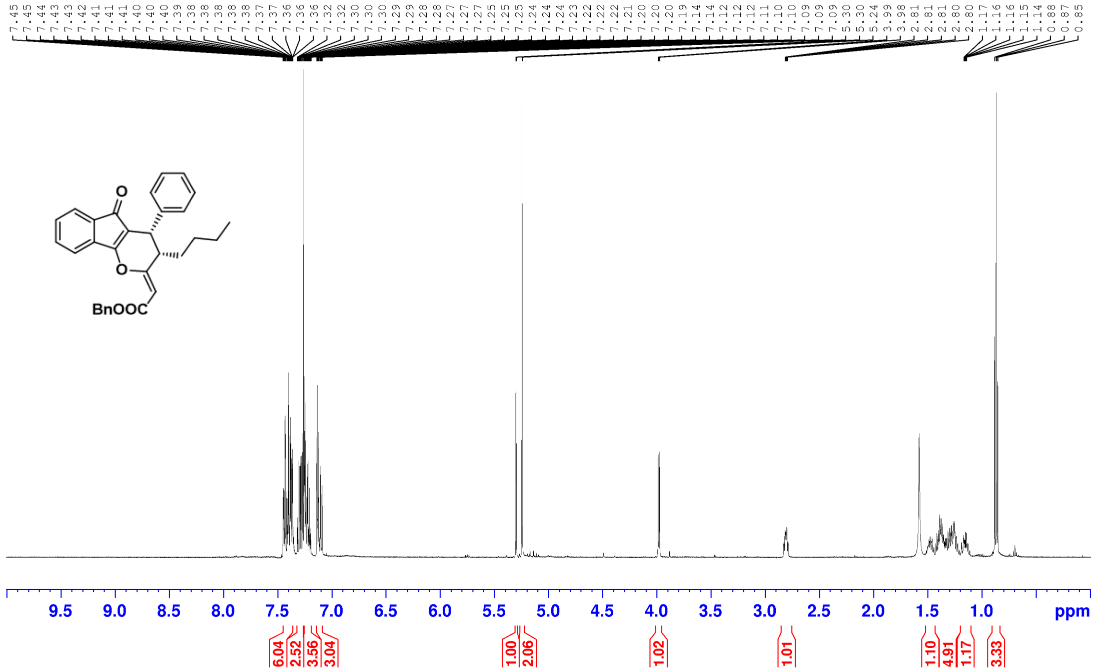

^{13}C-NMR (125 MHz, $CDCl_3$, 298 K)

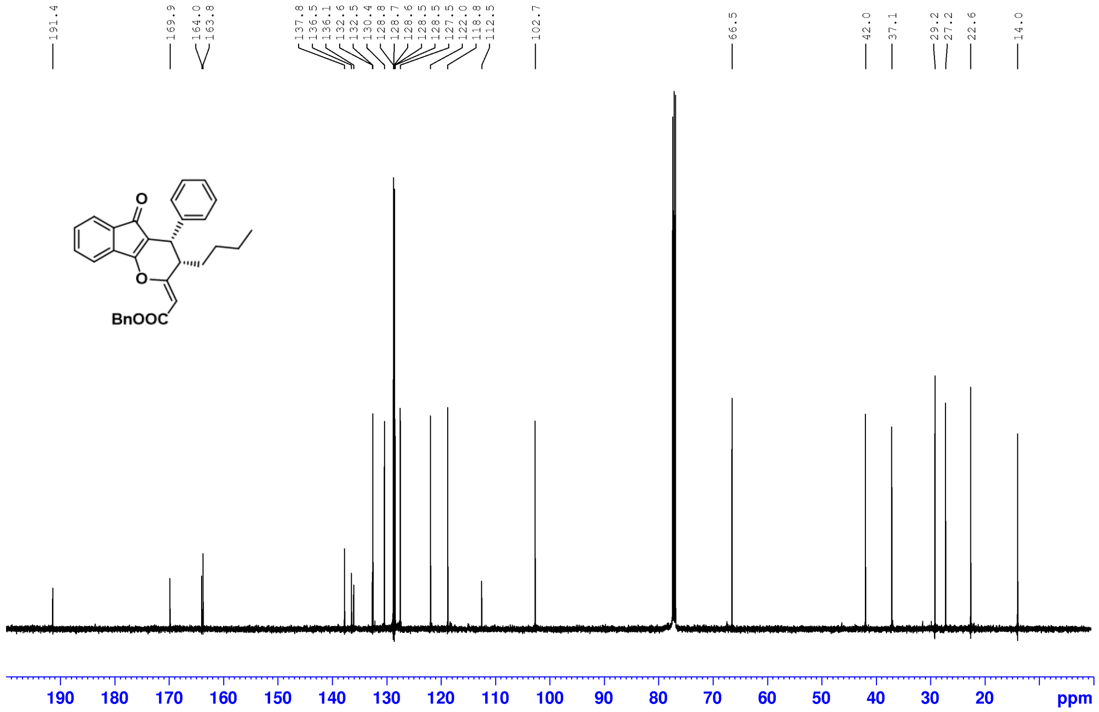

NMR of **3u**$_{\mathbf{minor}}$

^{1}H-NMR (700 MHz, $CDCl_3$, 298 K)

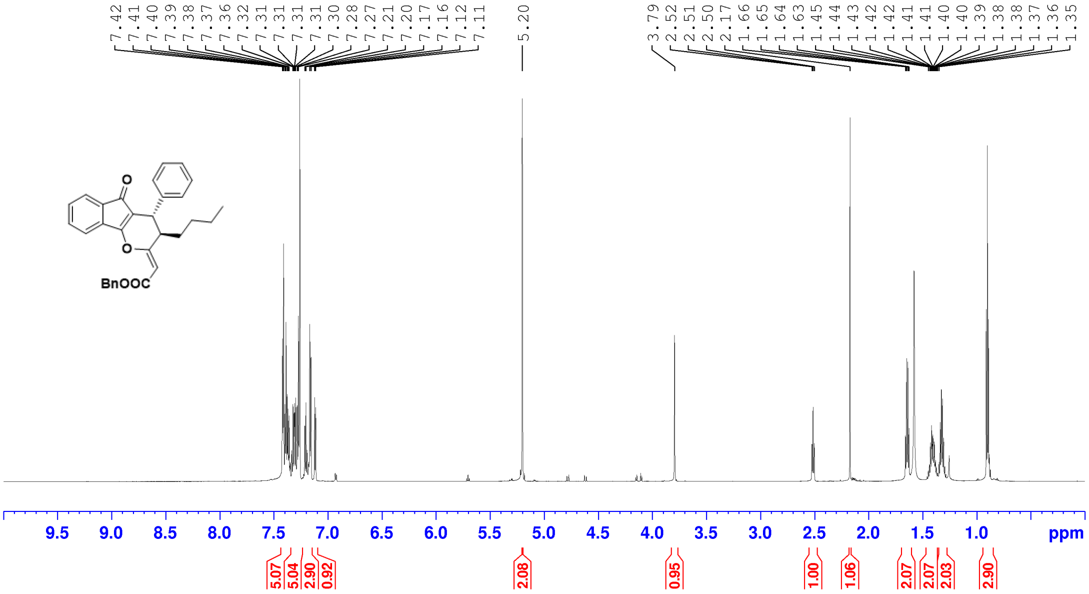

^{13}C-NMR (175 MHz, $CDCl_3$, 298 K)

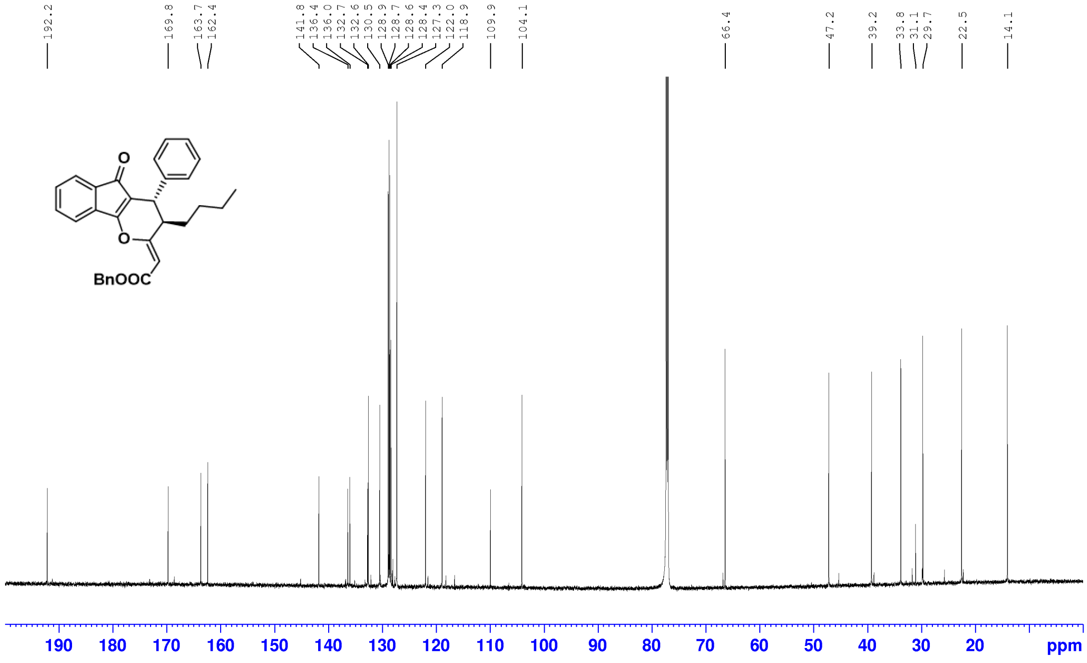

NMR of **3v**

^{1}H-NMR (300 MHz, $CDCl_3$, 298 K)

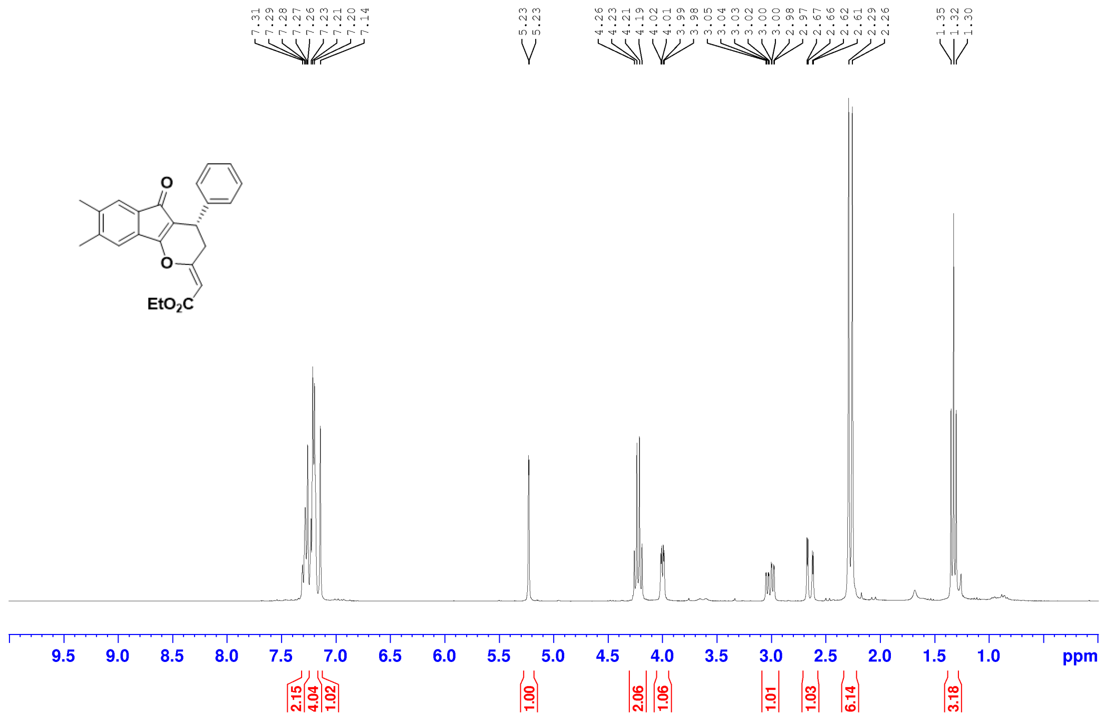

^{13}C-NMR (75 MHz, $CDCl_3$, 298 K)

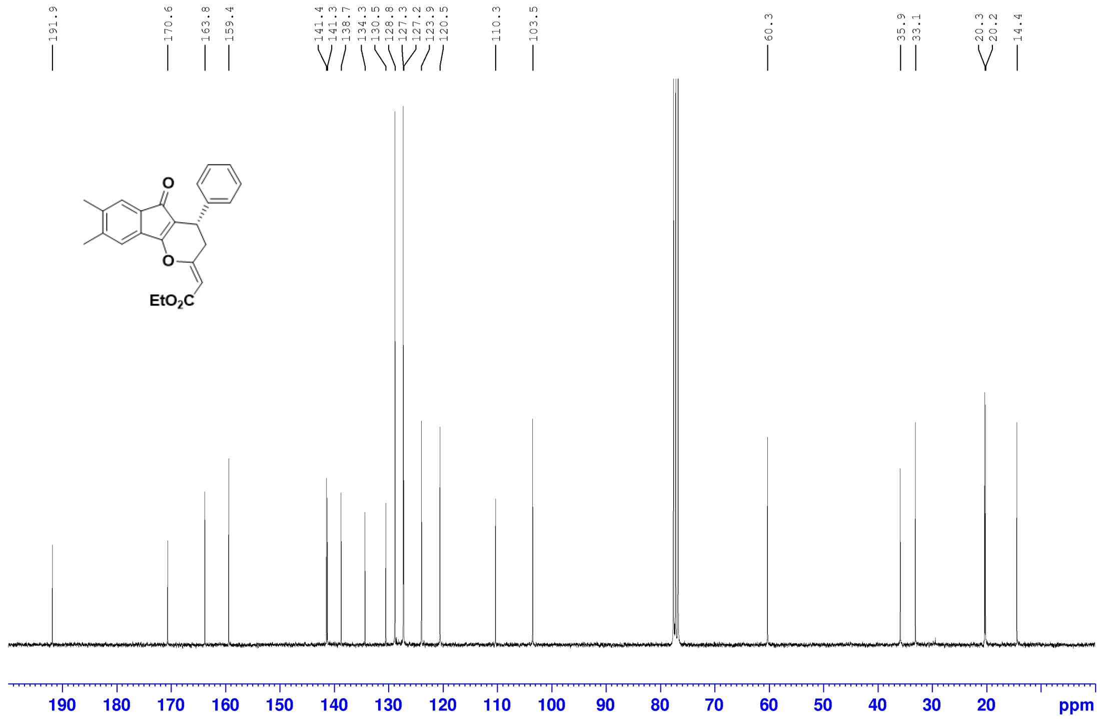

NMR of **3w**

^{1}H-NMR (300 MHz, $CDCl_3$, 298 K)

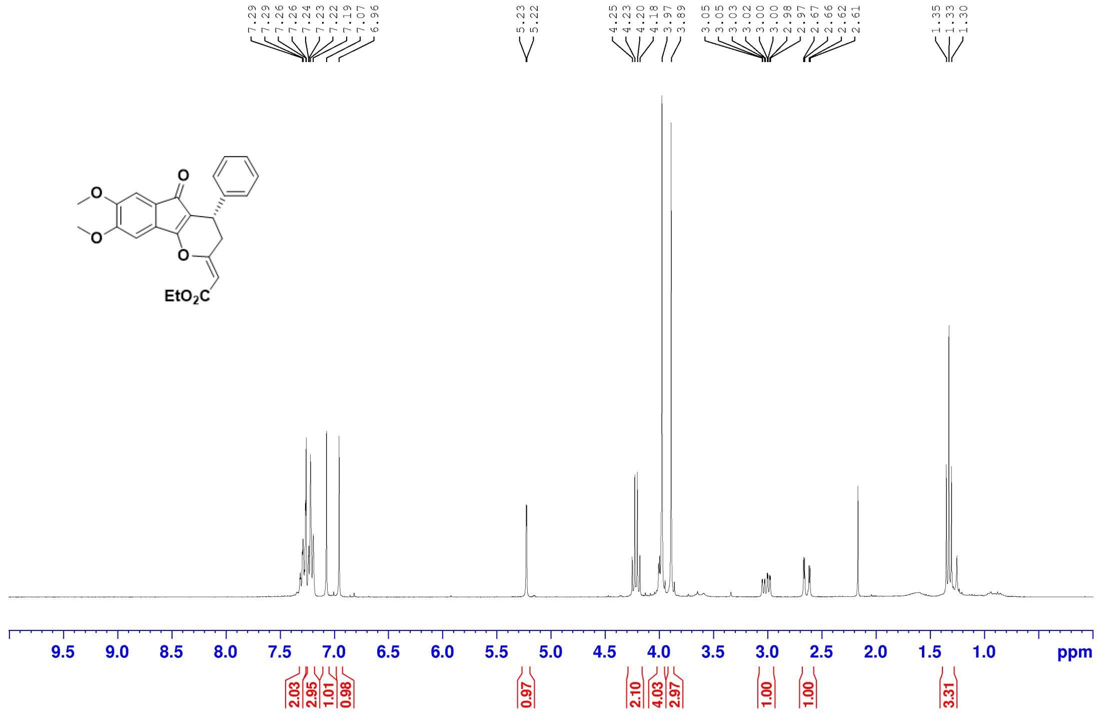

^{13}C-NMR (125 MHz, $CDCl_3$, 298 K)

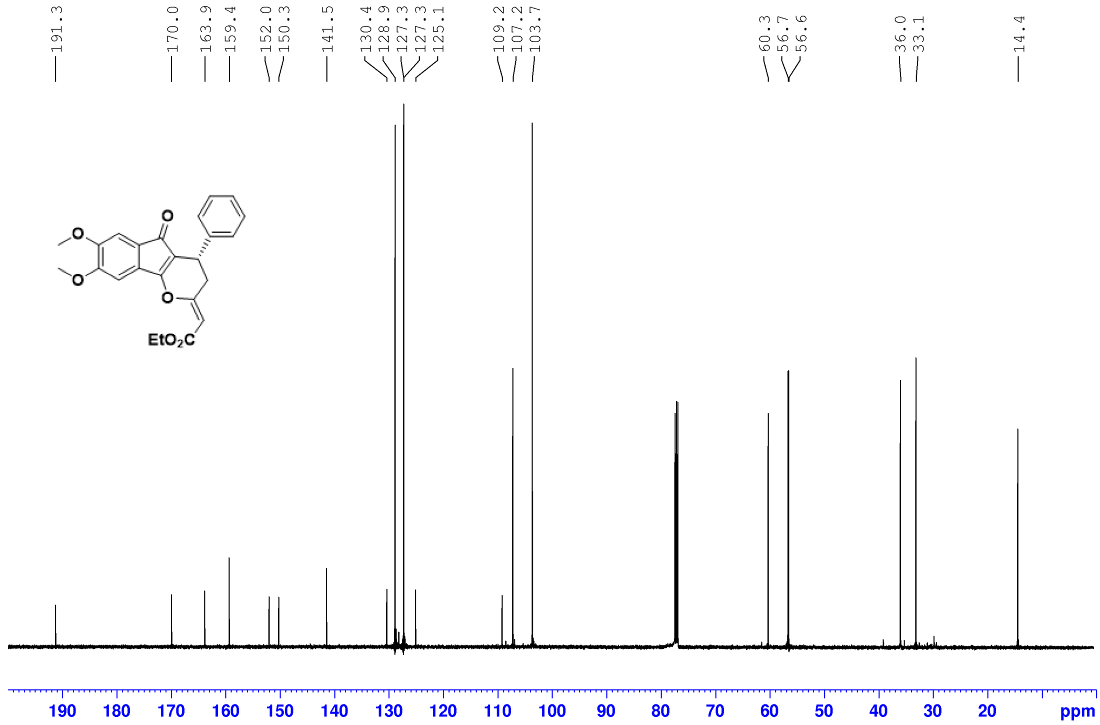

NMR of **3x**

^{1}H-NMR (300 MHz, $CDCl_3$, 298 K)

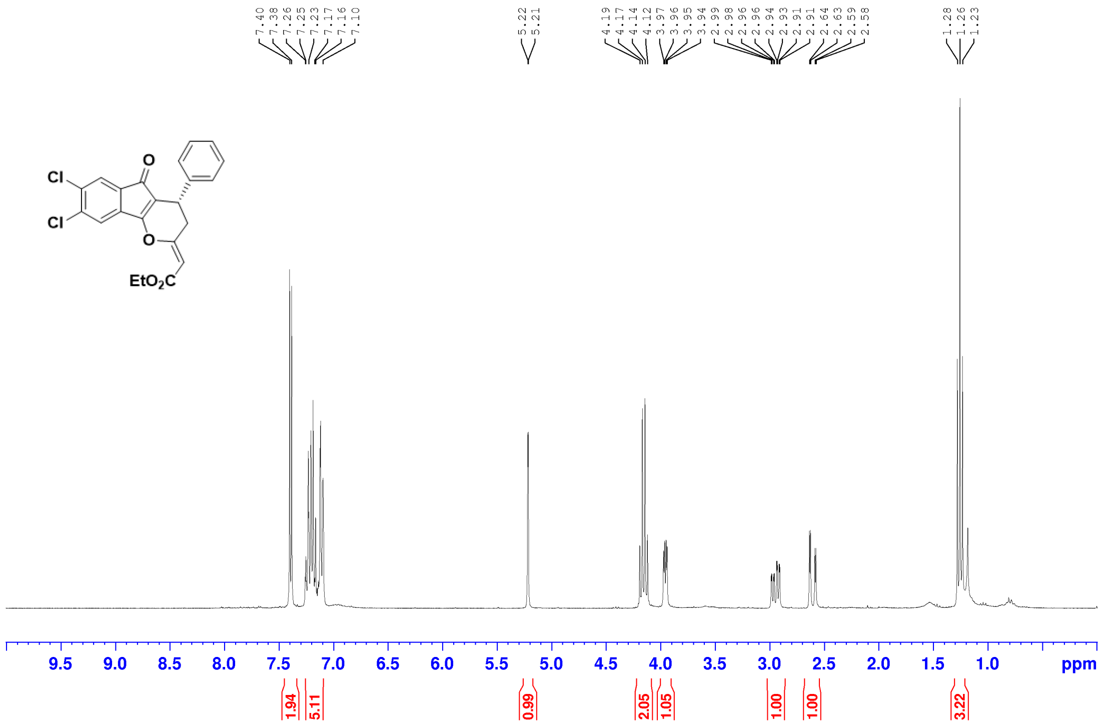

^{13}C-NMR (75 MHz, $CDCl_3$, 298 K)

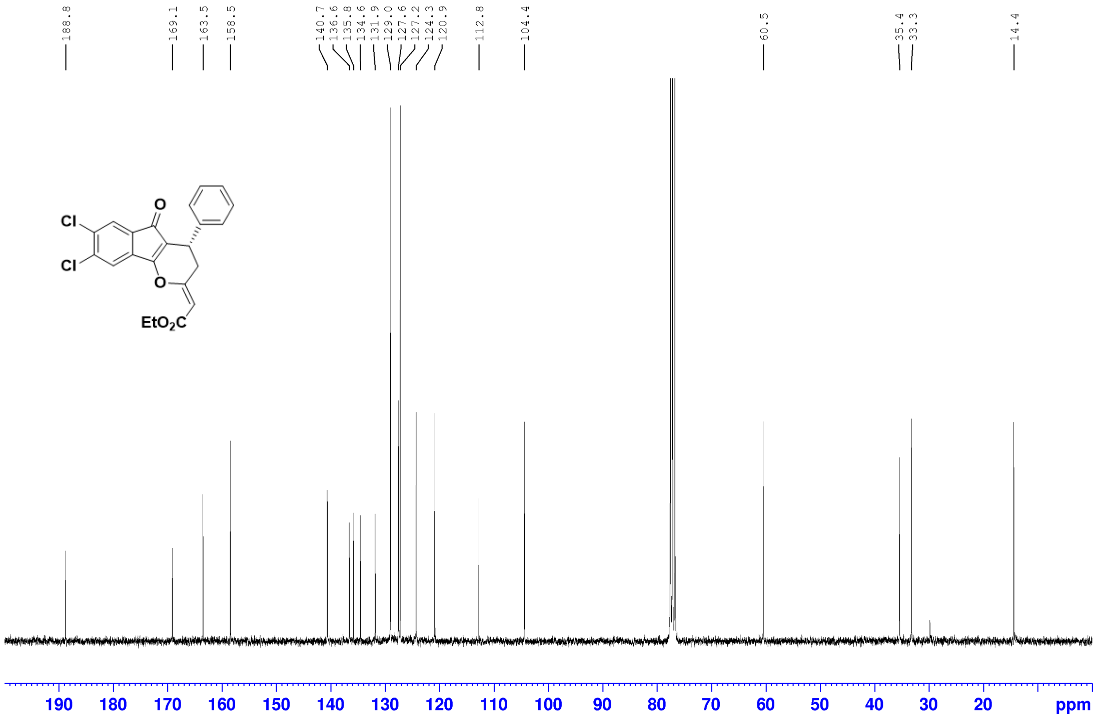

NMR of **3y**

^{1}H-NMR (300 MHz, $CDCl_3$, 298 K)

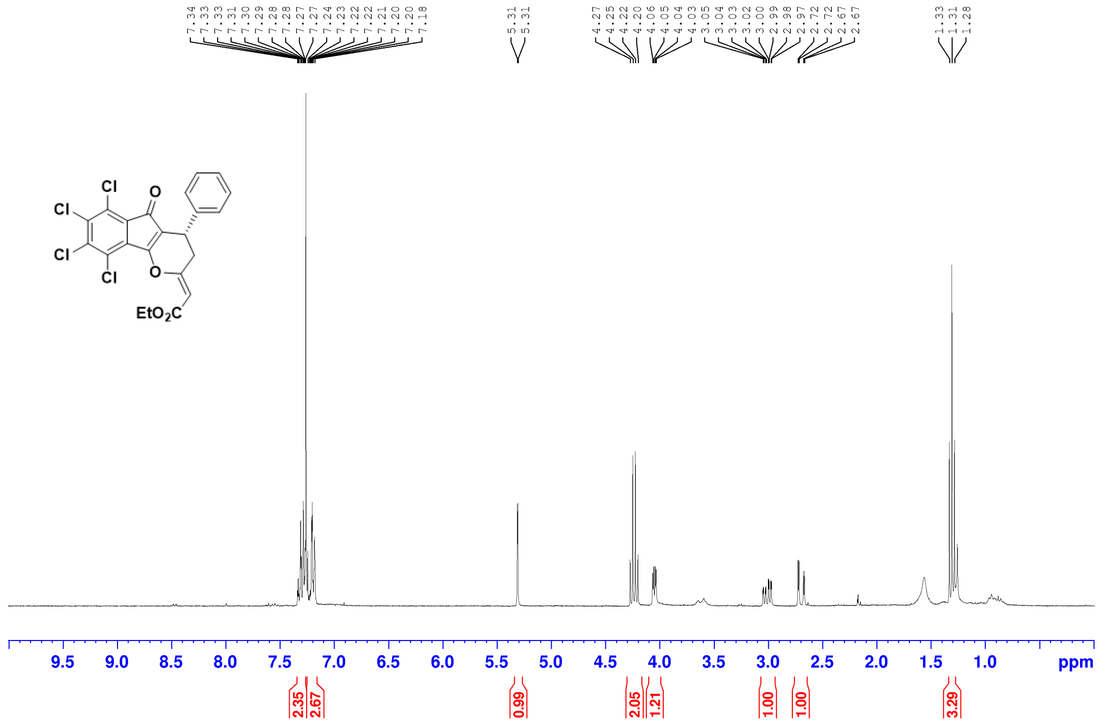

^{13}C-NMR (75 MHz, $CDCl_3$, 298 K)

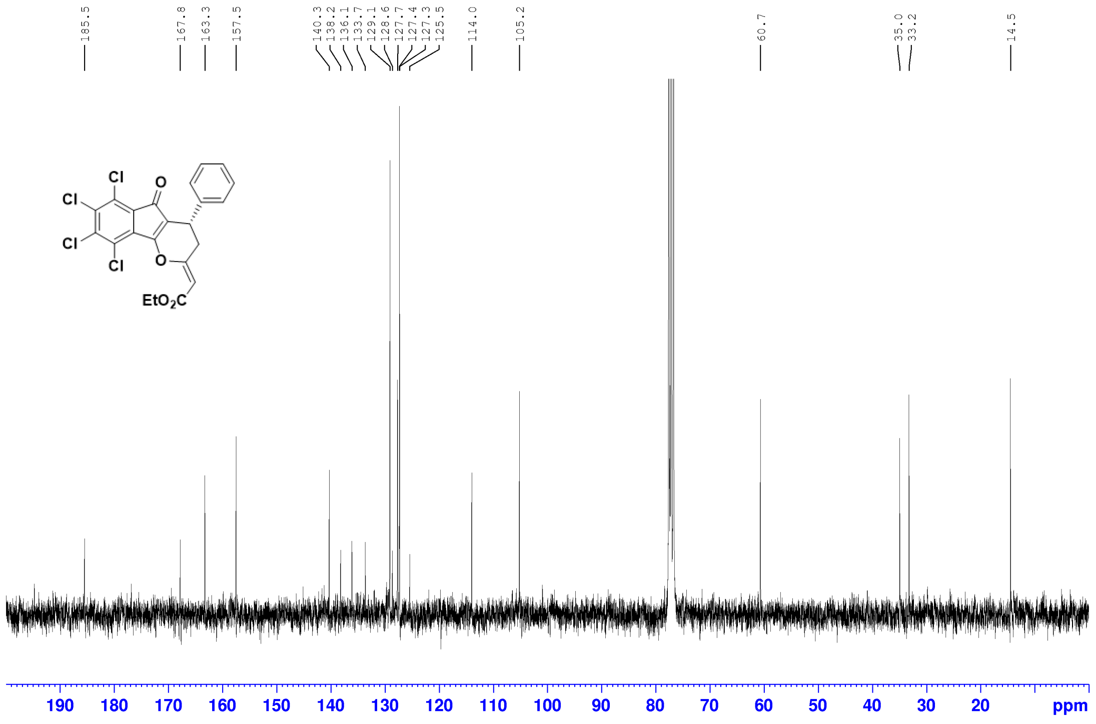

NMR of **3z**

^{1}H-NMR (300 MHz, $CDCl_3$, 298 K)

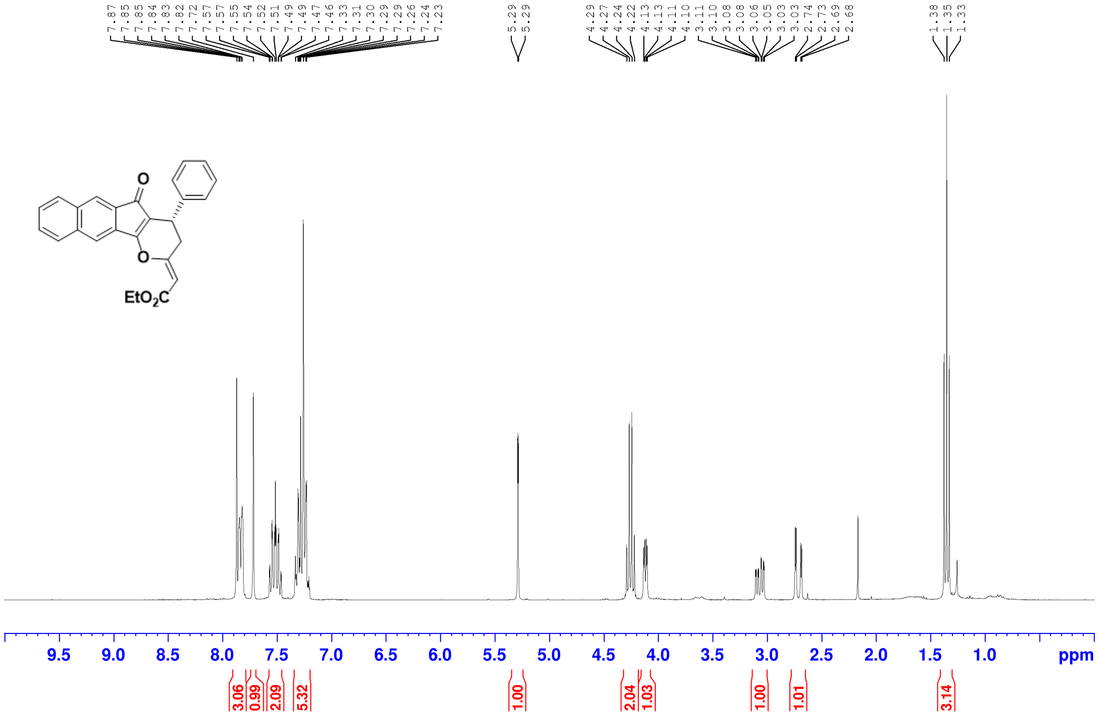

^{13}C-NMR (75 MHz, $CDCl_3$, 298 K)

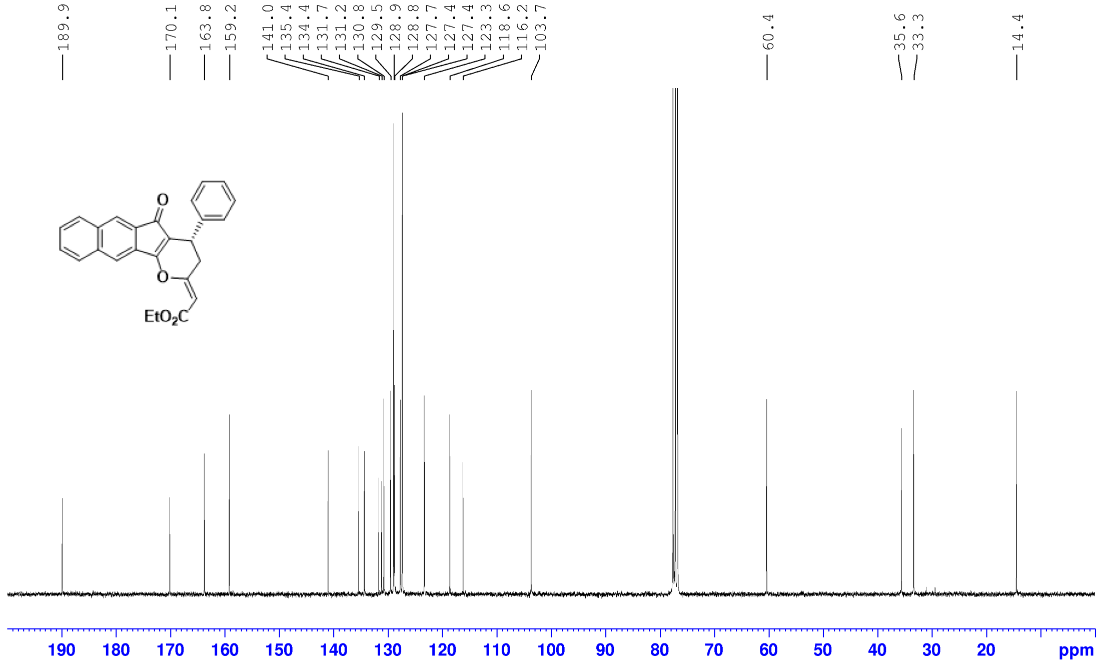

NMR of **7**

^{1}H-NMR (300 MHz, $CDCl_3$, 298 K)

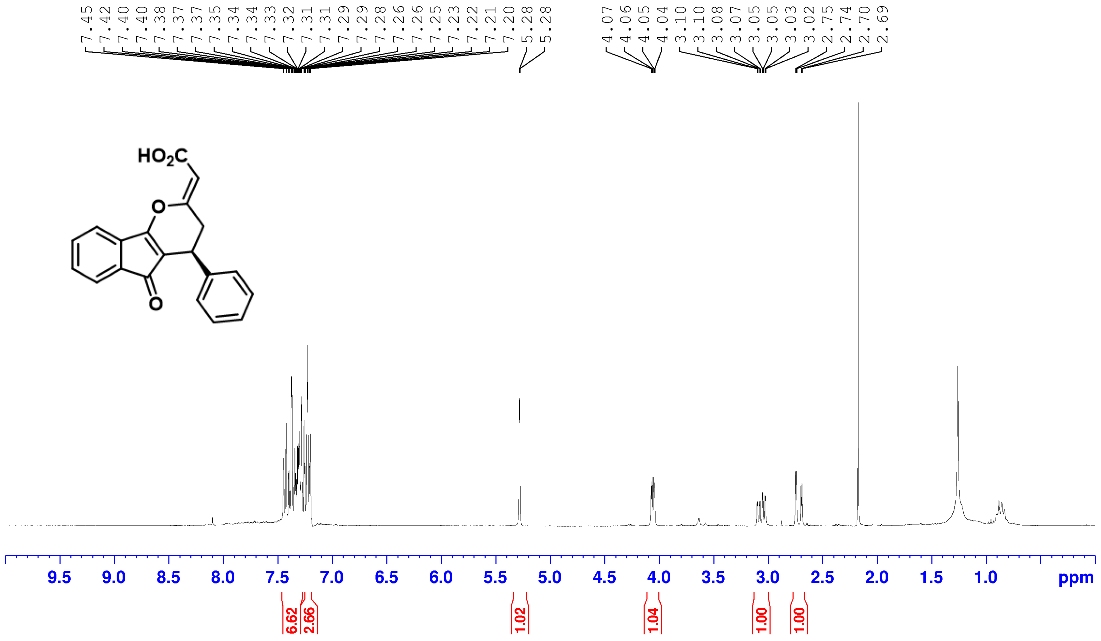

^{13}C-NMR (75 MHz, $CDCl_3$, 298 K)

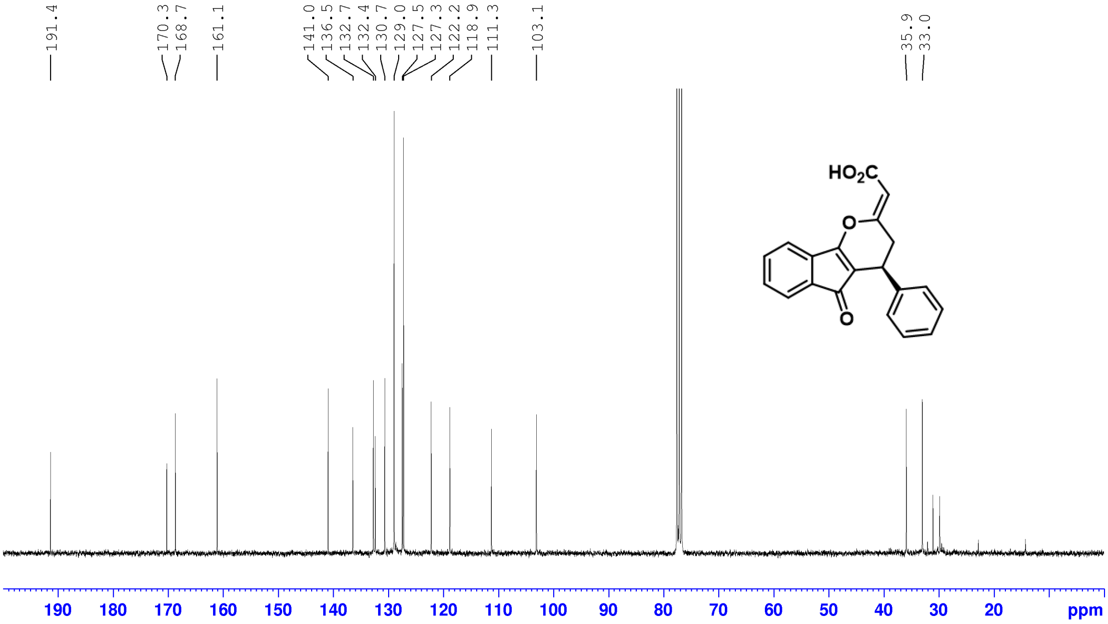

NMR of $\mathbf{8}_{major}$

^{1}H-NMR (300 MHz, $CDCl_3$, 298 K)

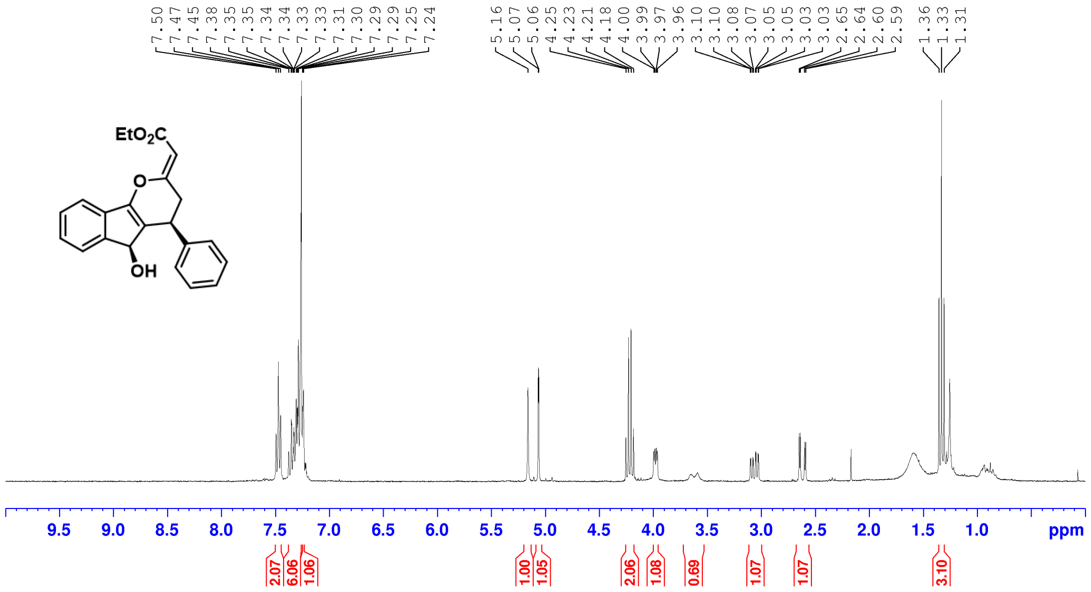

^{13}C-NMR (125 MHz, $CDCl_3$, 298 K)

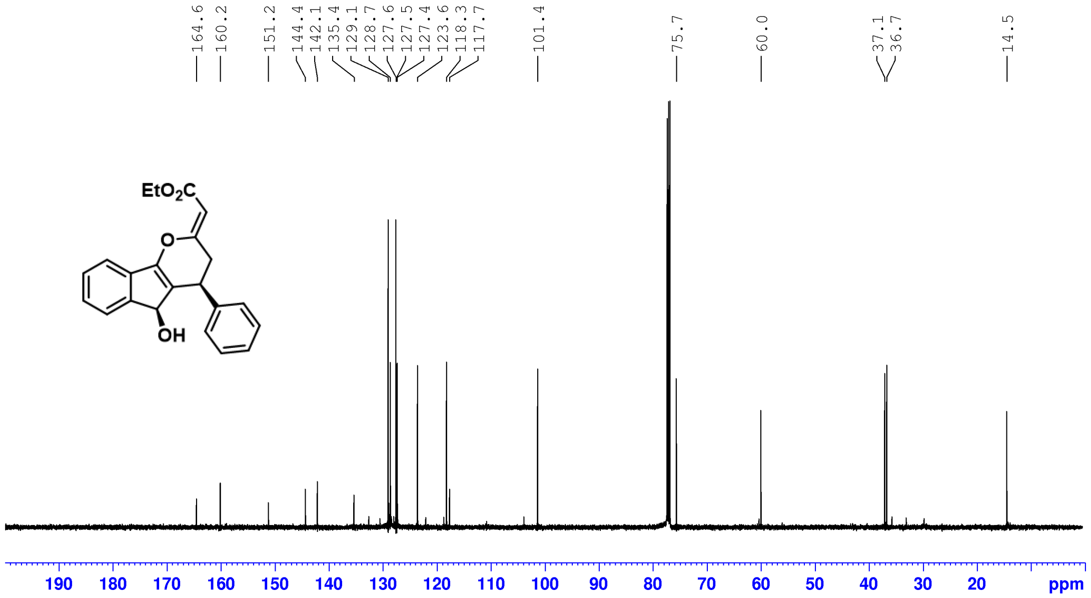

NMR of **8**$_{minor}$

^{1}H-NMR (300 MHz, $CDCl_3$, 298 K)

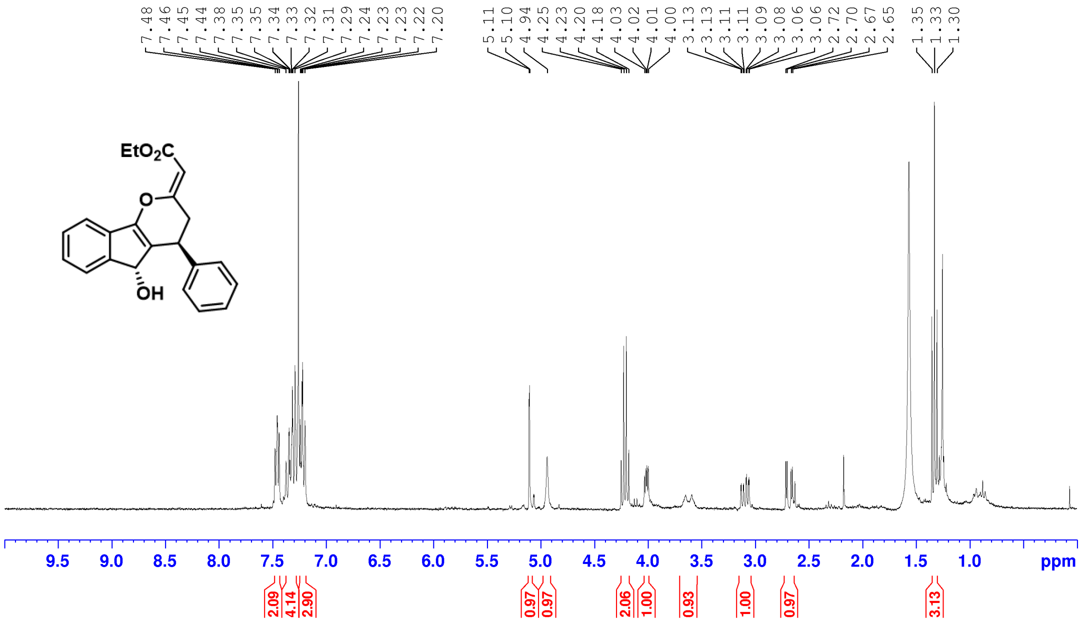

^{13}C-NMR (175 MHz, $CDCl_3$, 298 K)

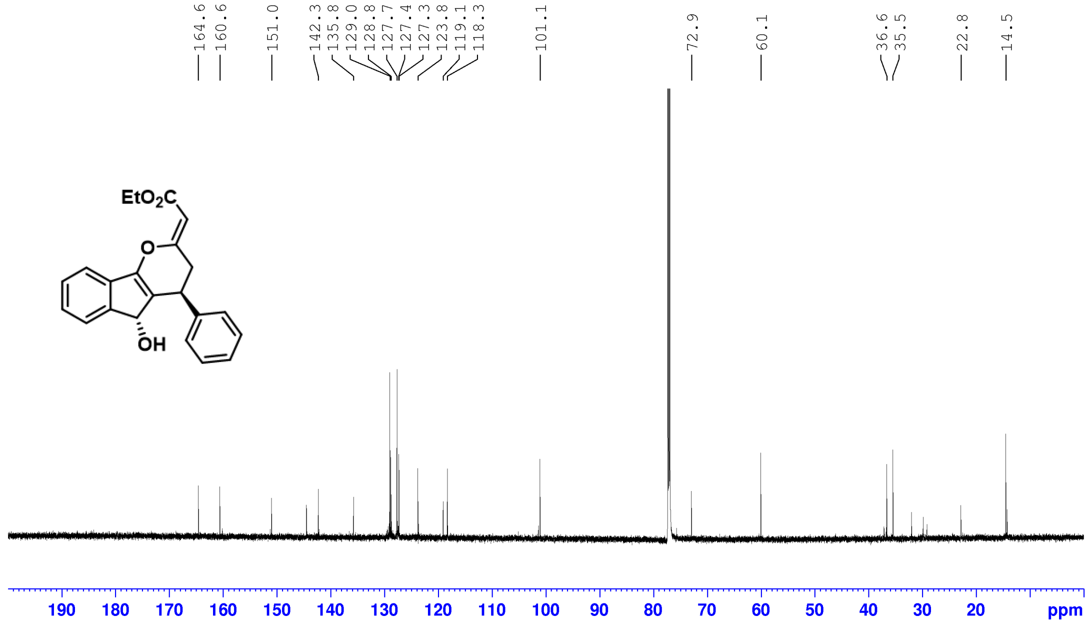

NMR of **9**$_{major}$

^{1}H-NMR (300 MHz, $CDCl_3$, 298 K)

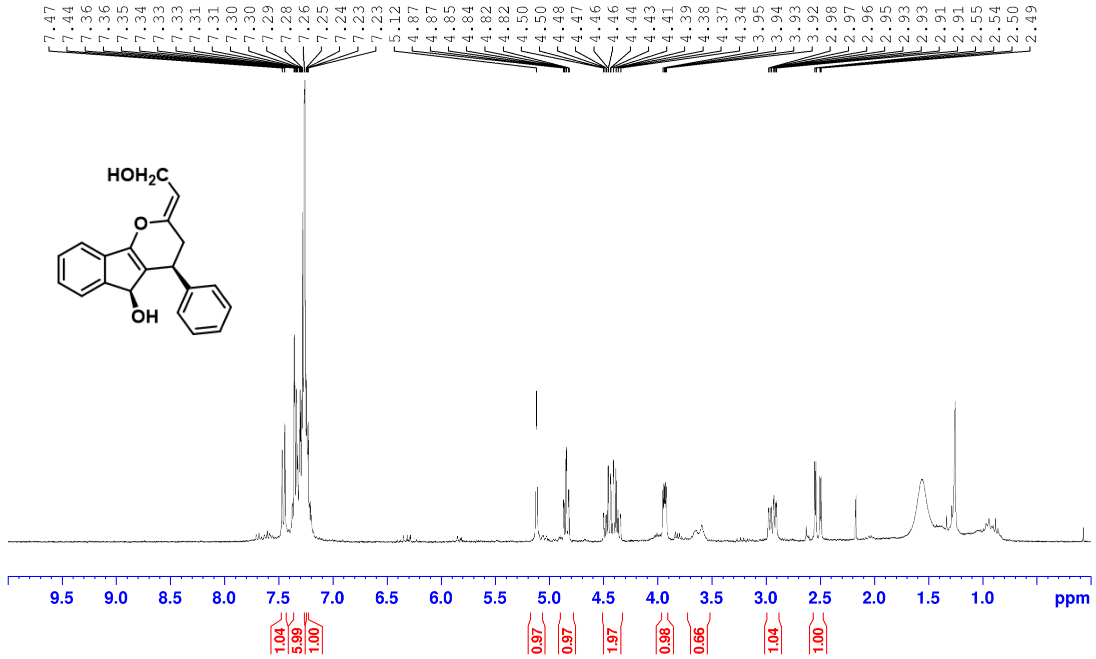

^{13}C-NMR (175 MHz, $CDCl_3$, 298 K)

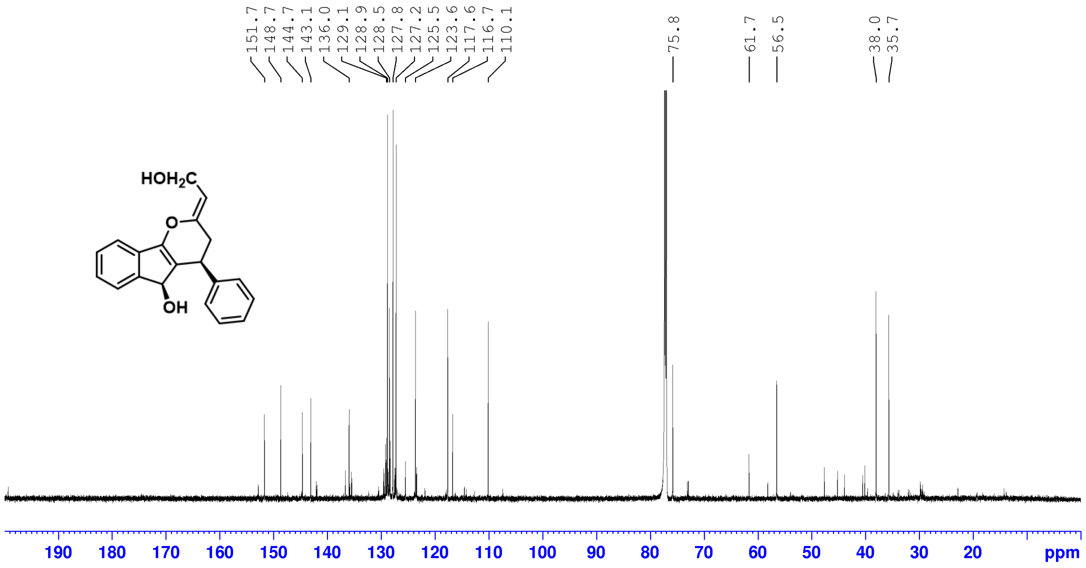

NMR of $\mathbf{9}_{minor}$

^{1}H-NMR (300 MHz, $CDCl_3$, 298 K)

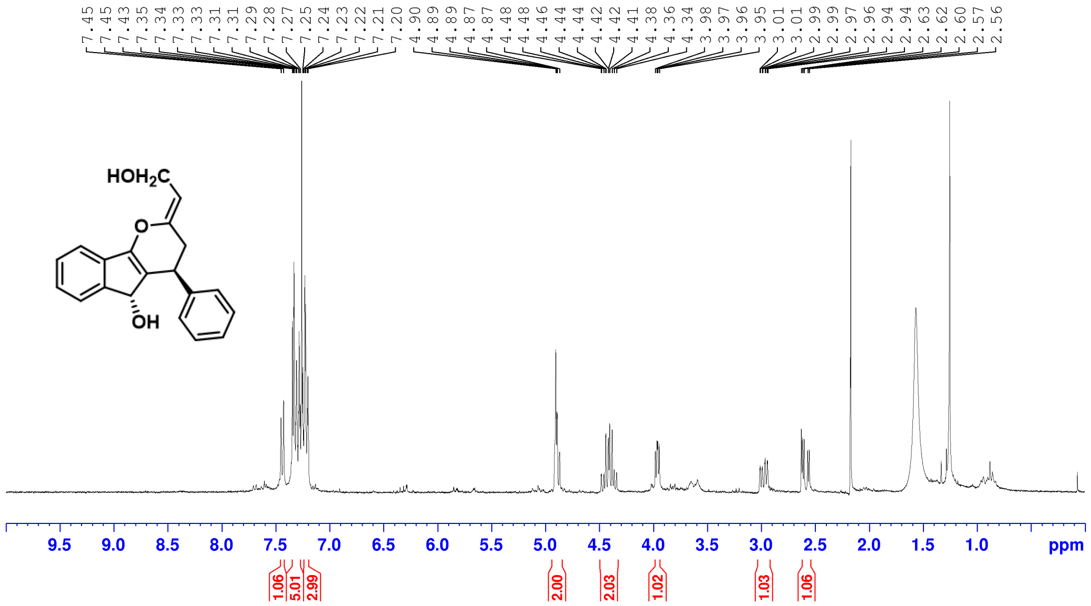

^{13}C-NMR (175 MHz, $CDCl_3$, 298 K)

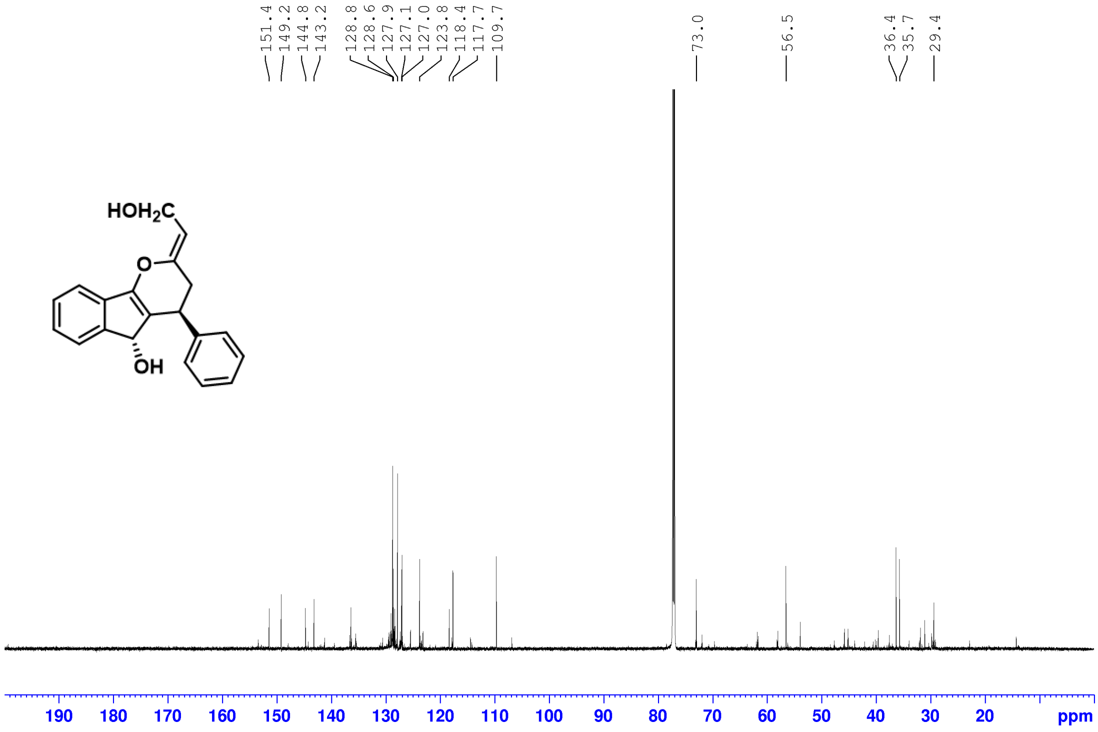

NMR of **10**

^{1}H-NMR (700 MHz, $CDCl_3$, 298 K)

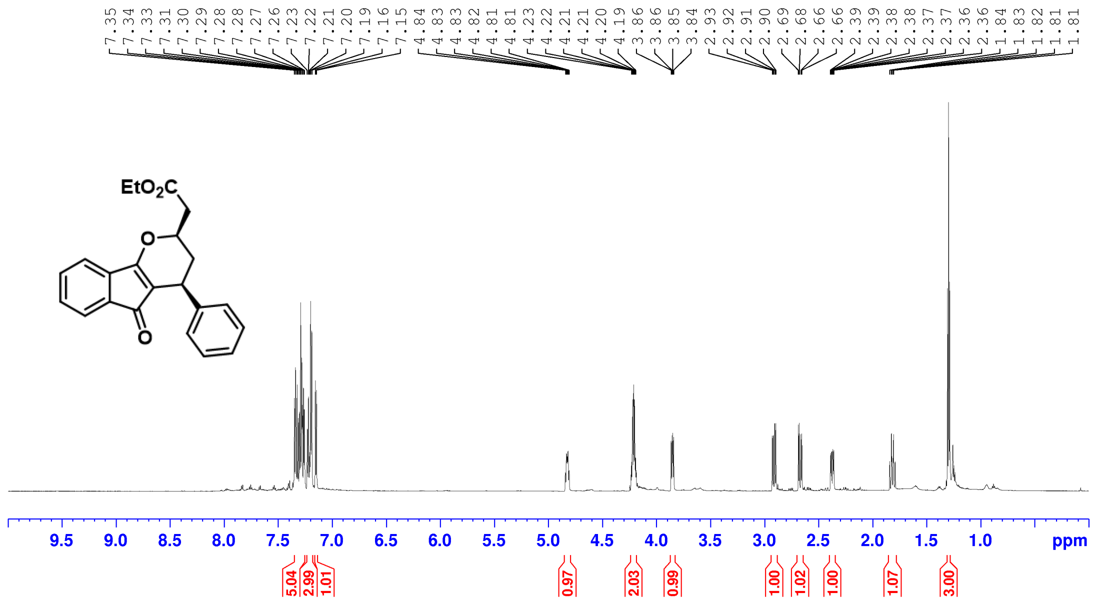

^{13}C-NMR (175 MHz, $CDCl_3$, 298 K)

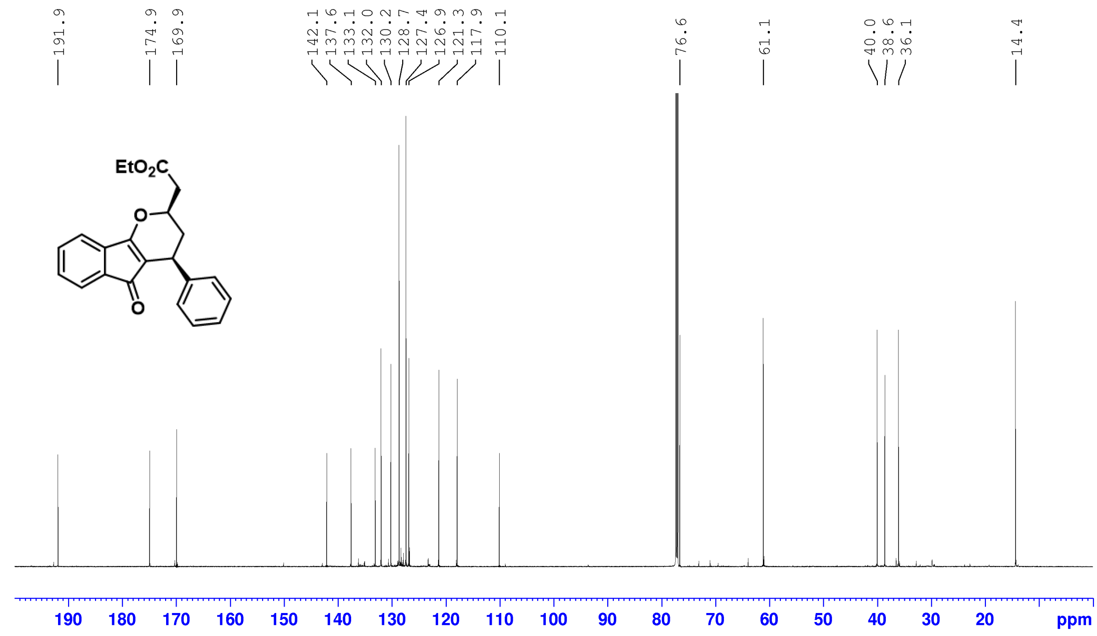

8. Copies of HPLC chromatograms

Chromatograms of **3a**

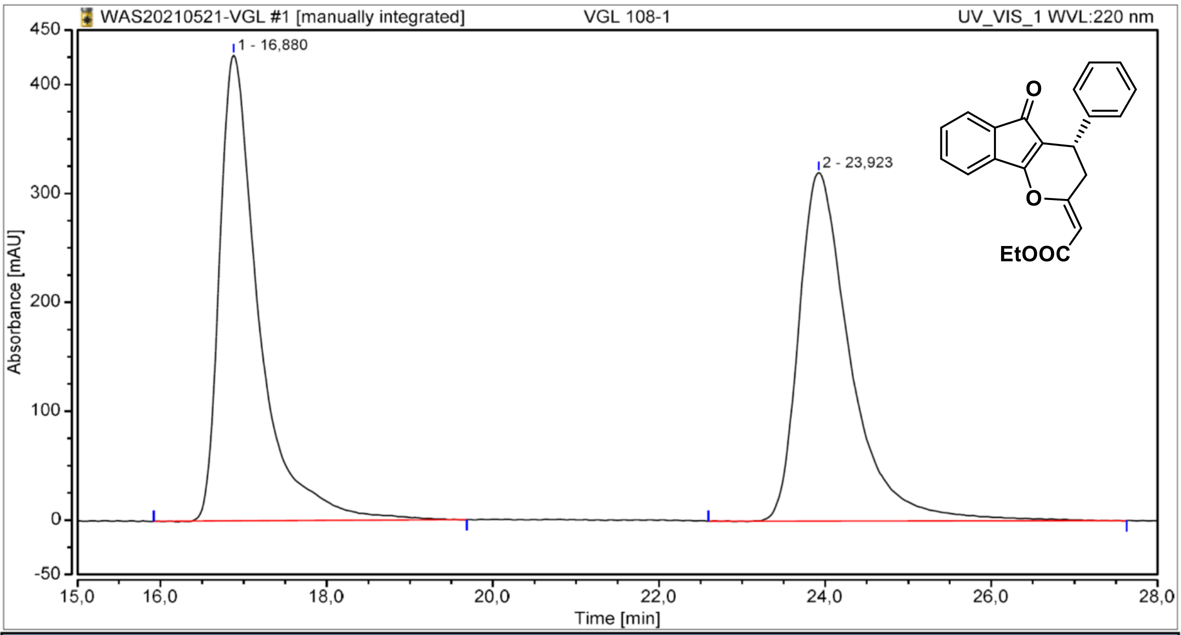

Integration Results							
No.	Peak Name	Retention Time min	Area mAU*min	Height mAU	Relative Area %	Relative Height %	Amount n.a.
1		16,880	234,477	427,675	50,38	57,21	n.a.
2		23,923	230,922	319,857	49,62	42,79	n.a.
Total:			465,399	747,532	100,00	100,00	

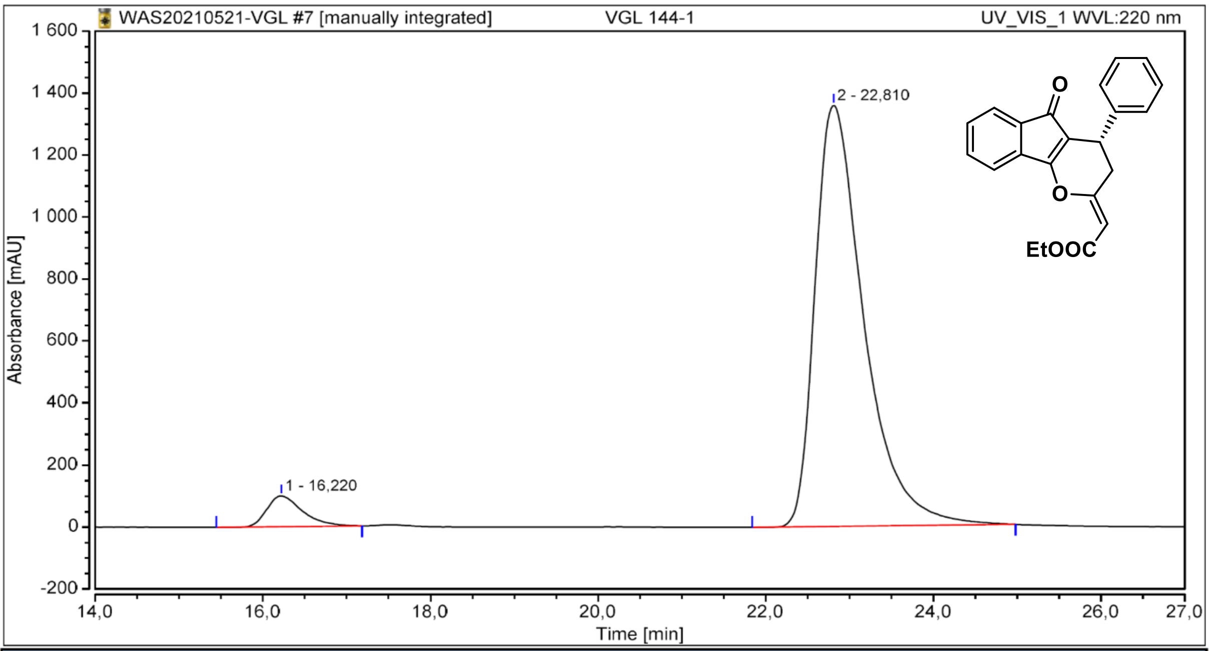

Integration Results							
No.	Peak Name	Retention Time min	Area mAU*min	Height mAU	Relative Area %	Relative Height %	Amount n.a.
1		16,220	49,177	99,591	5,10	6,83	n.a.
2		22,810	915,739	1358,057	94,90	93,17	n.a.
Total:			964,916	1457,648	100,00	100,00	

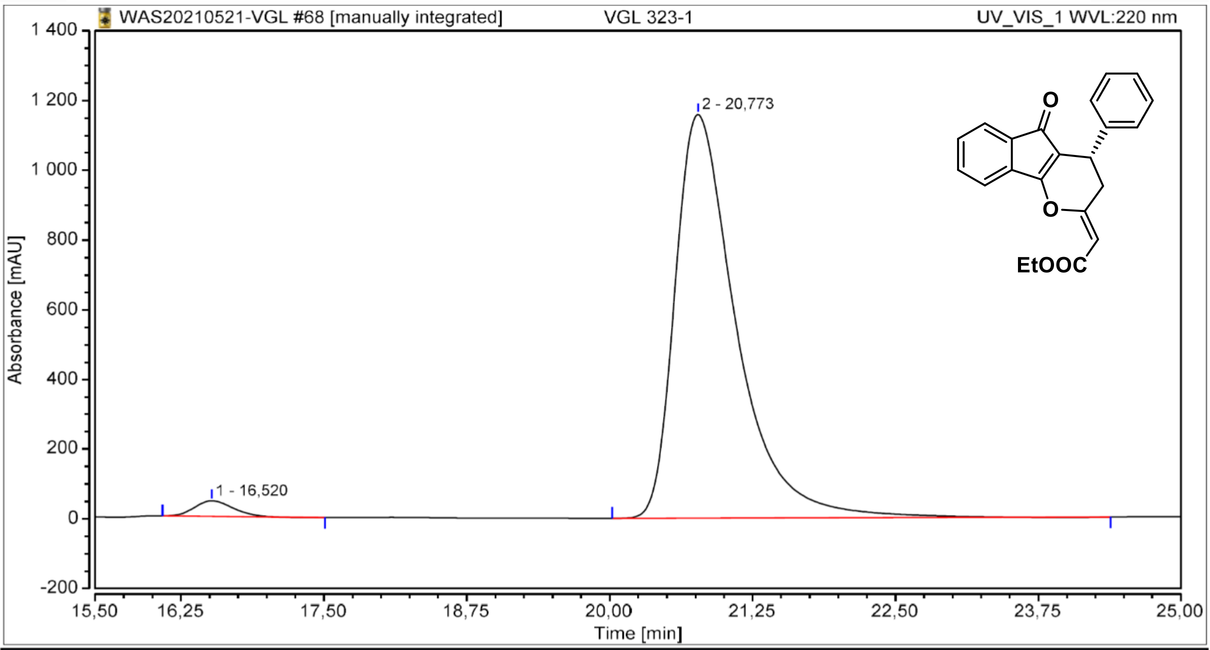

Integration Results							
No.	Peak Name	Retention Time min	Area mAU*min	Height mAU	Relative Area %	Relative Height %	Amount n.a.
1		16,520	17,429	44,594	2,36	3,71	n.a.
2		20,773	720,309	1158,589	97,64	96,29	n.a.
**Total:**			**737,737**	**1203,183**	**100,00**	**100,00**	

Chromatograms of **3b**

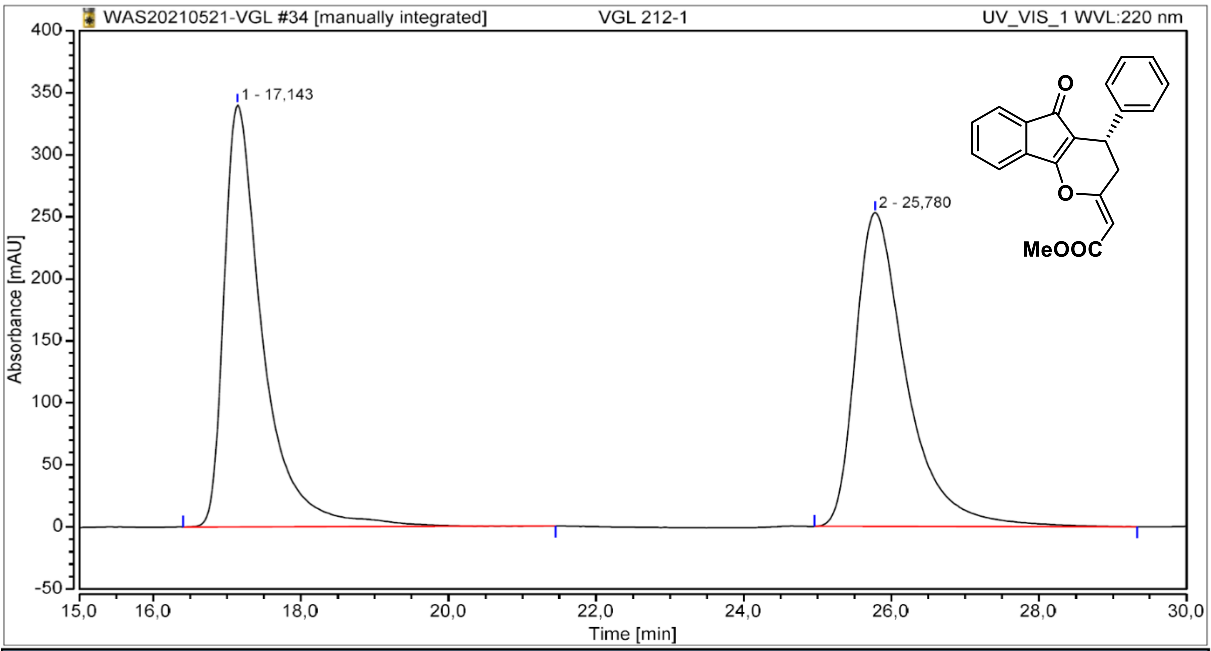

Integration Results							
No.	Peak Name	Retention Time min	Area mAU*min	Height mAU	Relative Area %	Relative Height %	Amount n.a.
1		17,143	206,336	340,099	50,41	57,34	n.a.
2		25,780	202,971	253,003	49,59	42,66	n.a.
**Total:**			**409,307**	**593,102**	**100,00**	**100,00**	

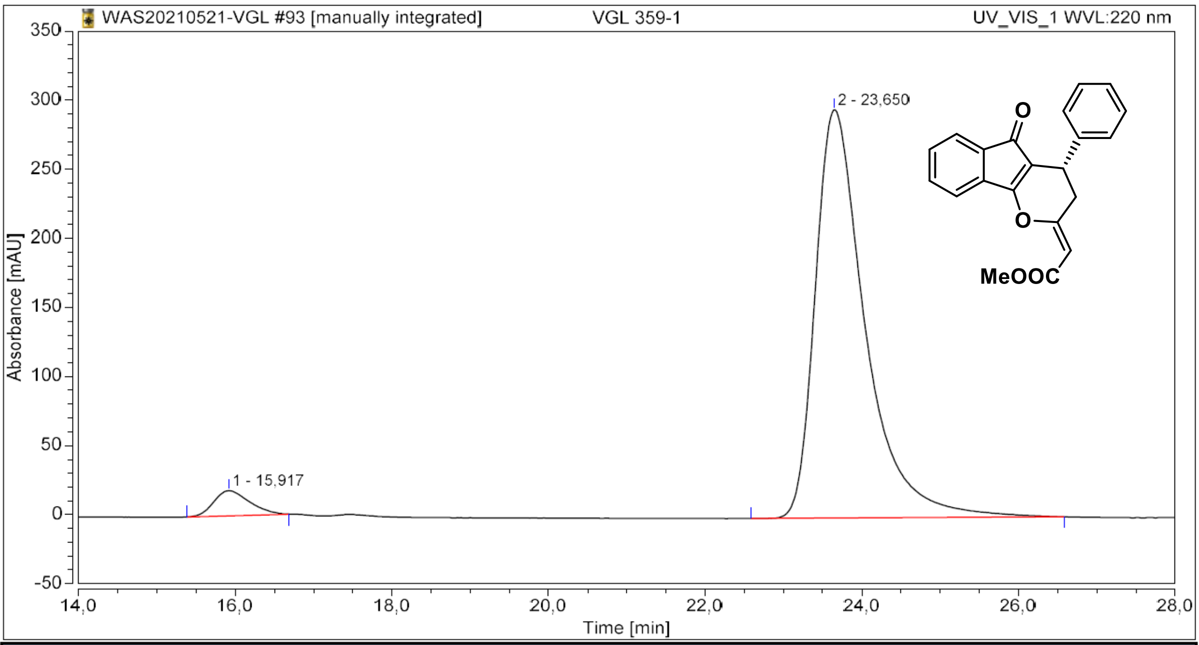

Integration Results							
No.	Peak Name	Retention Time min	Area mAU*min	Height mAU	Relative Area %	Relative Height %	Amount n.a.
1		15,917	9,577	18,151	4,20	5,79	n.a.
2		23,650	218,249	295,524	95,80	94,21	n.a.
**Total:**			**227,826**	**313,674**	**100,00**	**100,00**	

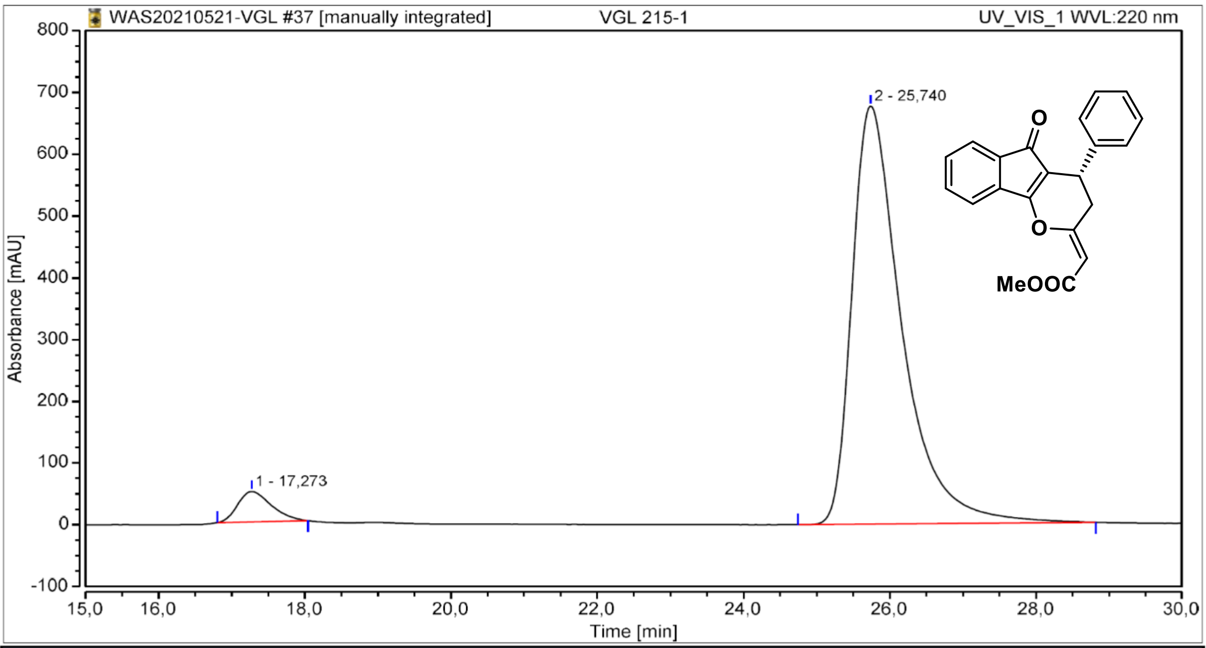

Integration Results							
No.	Peak Name	Retention Time min	Area mAU*min	Height mAU	Relative Area %	Relative Height %	Amount n.a.
1		17,273	26,117	49,074	4,67	6,76	n.a.
2		25,740	533,053	677,306	95,33	93,24	n.a.
**Total:**			**559,170**	**726,380**	**100,00**	**100,00**	

Chromatograms of **3c**

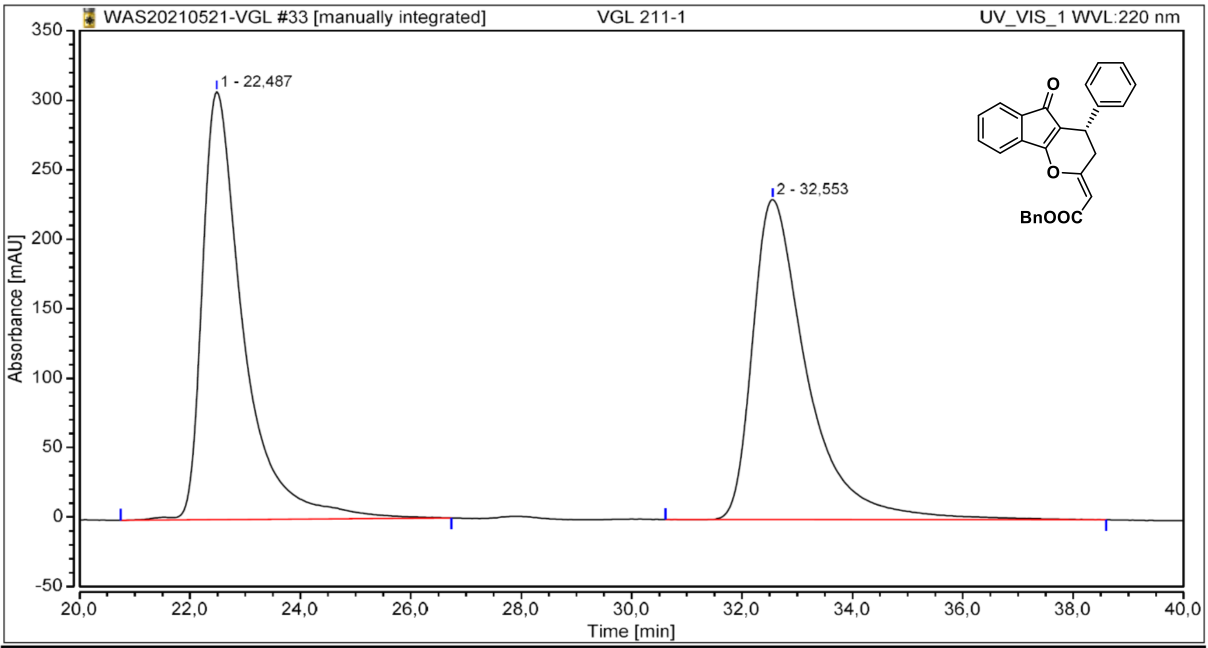

Integration Results							
No.	Peak Name	Retention Time<br>min	Area<br>mAU*min	Height<br>mAU	Relative Area<br>%	Relative Height<br>%	Amount<br>n.a.
1		22,487	264,407	307,936	50,14	57,20	n.a.
2		32,553	262,959	230,442	49,86	42,80	n.a.
**Total:**			**527,366**	**538,378**	**100,00**	**100,00**	

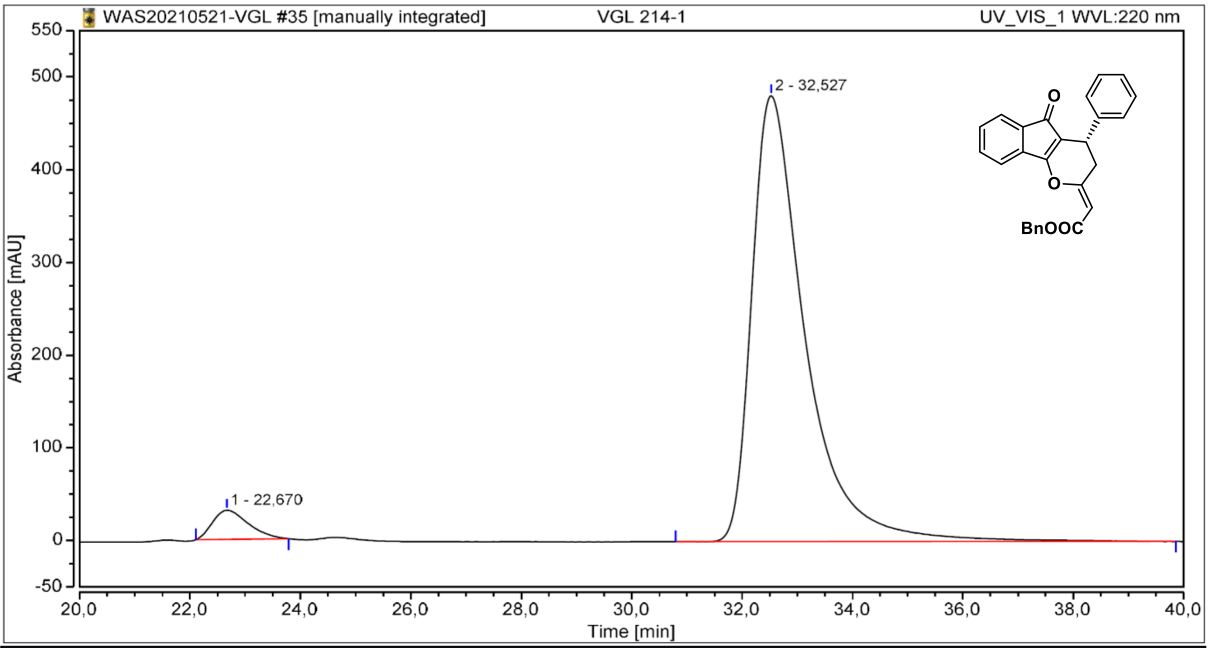

Integration Results							
No.	Peak Name	Retention Time<br>min	Area<br>mAU*min	Height<br>mAU	Relative Area<br>%	Relative Height<br>%	Amount<br>n.a.
1		22,670	23,491	31,372	4,18	6,13	n.a.
2		32,527	538,461	480,733	95,82	93,87	n.a.
**Total:**			**561,952**	**512,105**	**100,00**	**100,00**	

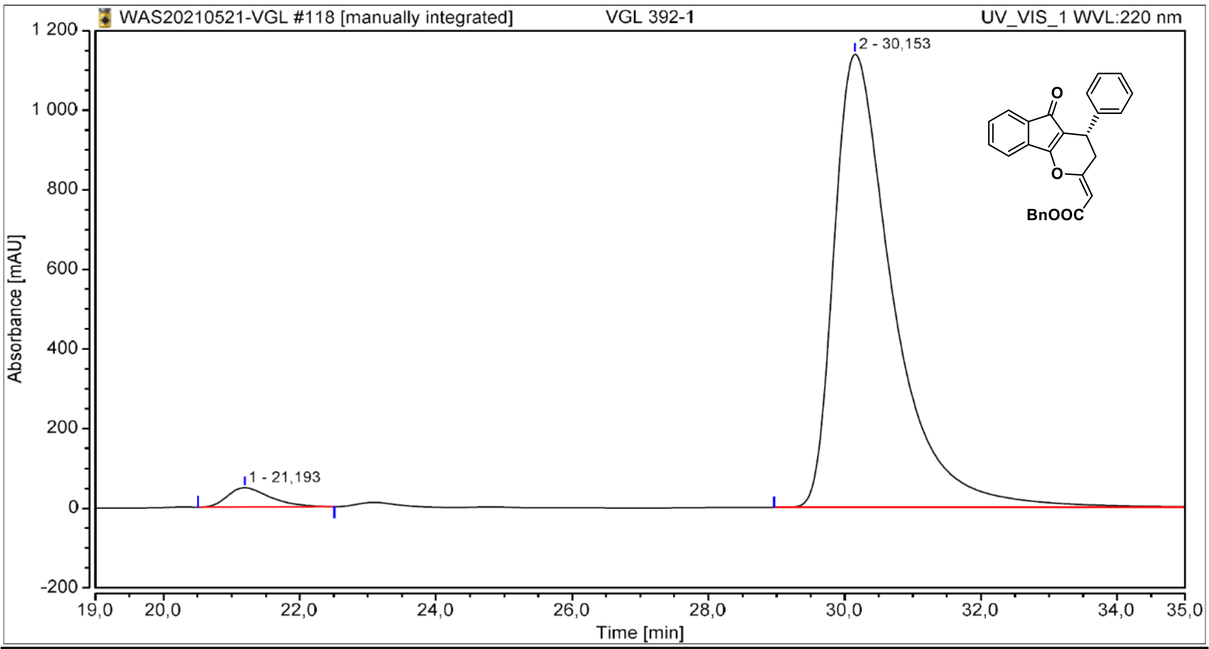

Integration Results							
No.	Peak Name	Retention Time min	Area mAU*min	Height mAU	Relative Area %	Relative Height %	Amount n.a.
1		21,193	35,028	48,431	2,96	4,08	n.a.
2		30,153	1148,919	1139,076	97,04	95,92	n.a.
**Total:**			**1183,947**	**1187,507**	**100,00**	**100,00**	

Chromatograms of **3d**

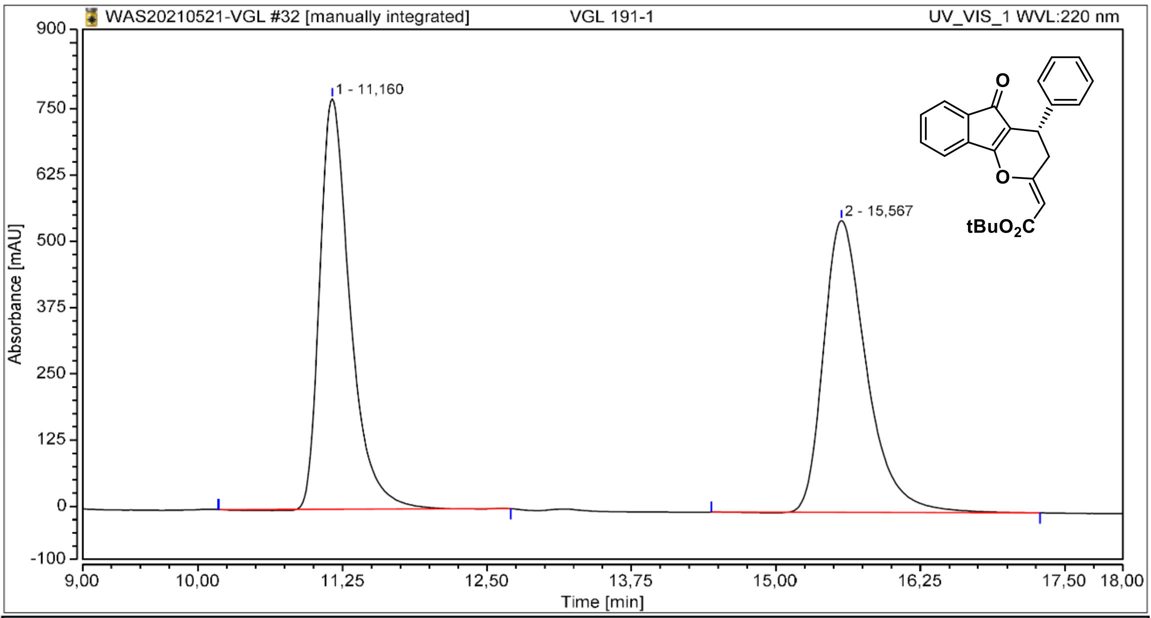

Integration Results							
No.	Peak Name	Retention Time min	Area mAU*min	Height mAU	Relative Area %	Relative Height %	Amount n.a.
1		11,160	234,643	774,239	49,76	58,44	n.a.
2		15,567	236,913	550,666	50,24	41,56	n.a.
**Total:**			**471,556**	**1324,905**	**100,00**	**100,00**	

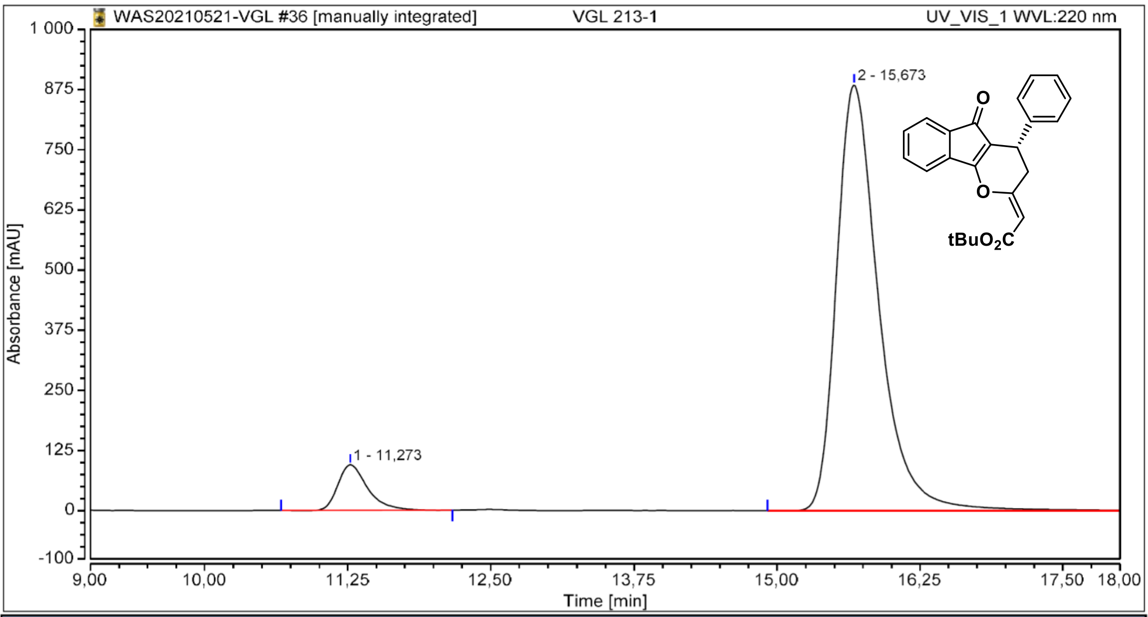

Integration Results							
No.	Peak Name	Retention Time min	Area mAU*min	Height mAU	Relative Area %	Relative Height %	Amount n.a.
1		11,273	28,289	94,458	6,93	9,65	n.a.
2		15,673	379,759	884,433	93,07	90,35	n.a.
**Total:**			**408,048**	**978,891**	**100,00**	**100,00**	

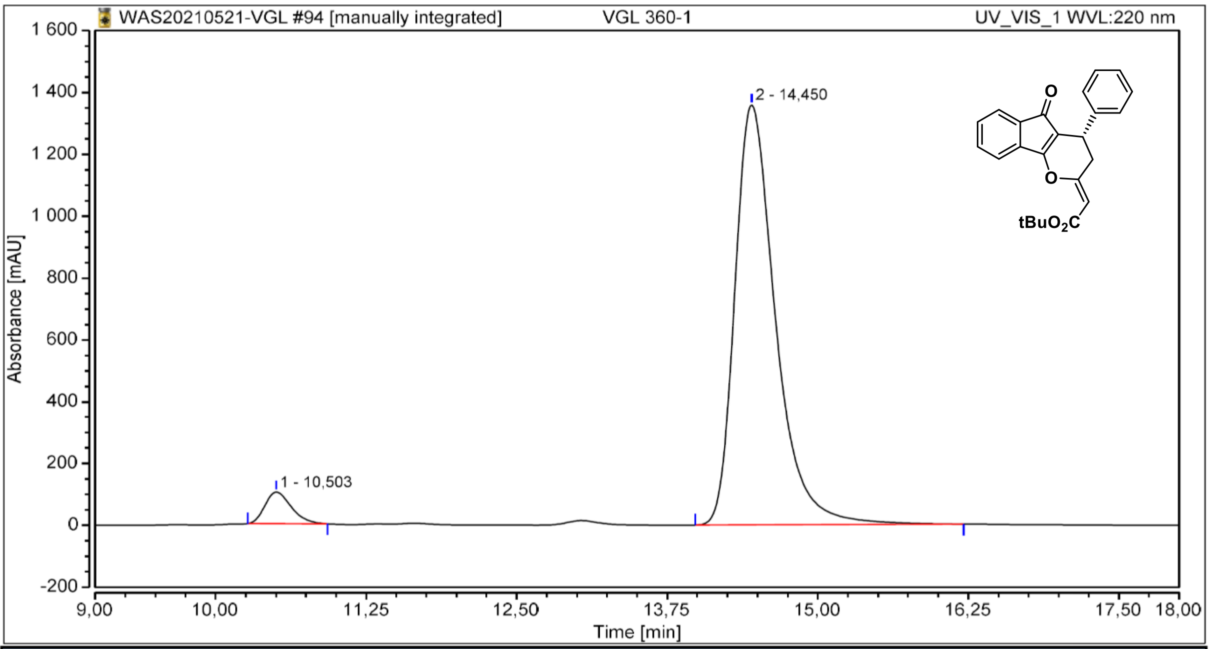

Integration Results							
No.	Peak Name	Retention Time min	Area mAU*min	Height mAU	Relative Area %	Relative Height %	Amount n.a.
1		10,503	26,107	102,321	4,69	7,01	n.a.
2		14,450	530,437	1358,201	95,31	92,99	n.a.
**Total:**			**556,544**	**1460,521**	**100,00**	**100,00**	

Chromatograms of **3e**

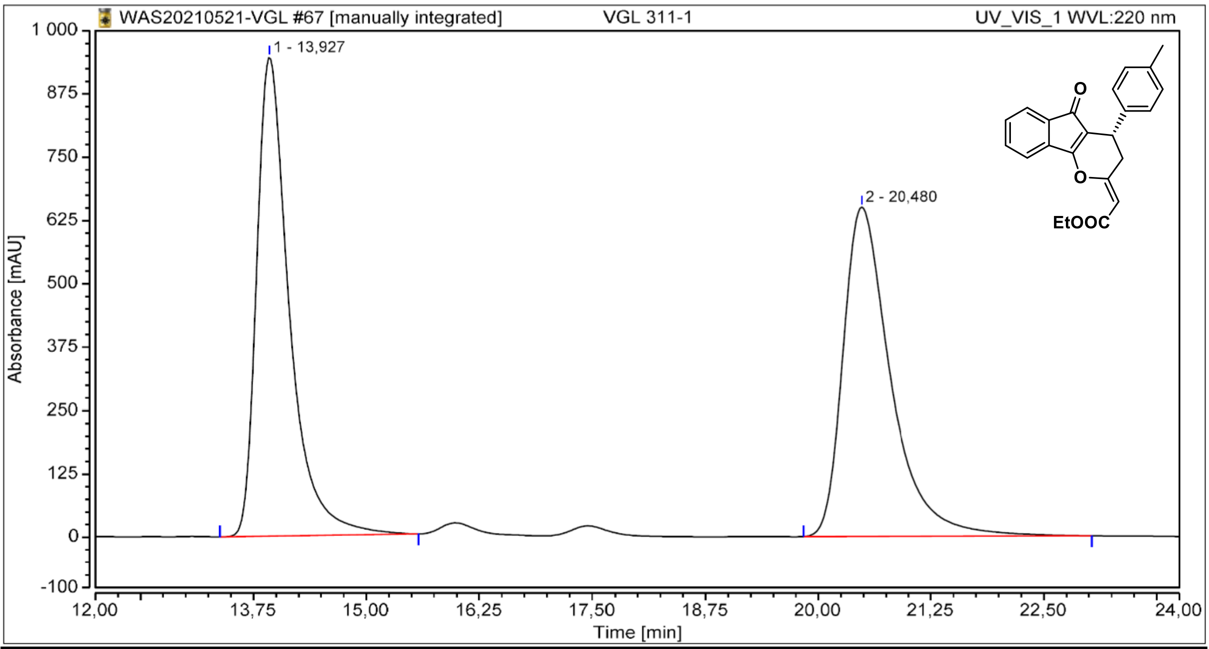

Integration Results							
No.	Peak Name	Retention Time min	Area mAU*min	Height mAU	Relative Area %	Relative Height %	Amount n.a.
1		13,927	395,728	945,189	49,79	59,27	n.a.
2		20,480	399,050	649,583	50,21	40,73	n.a.
**Total:**			**794,778**	**1594,772**	**100,00**	**100,00**	

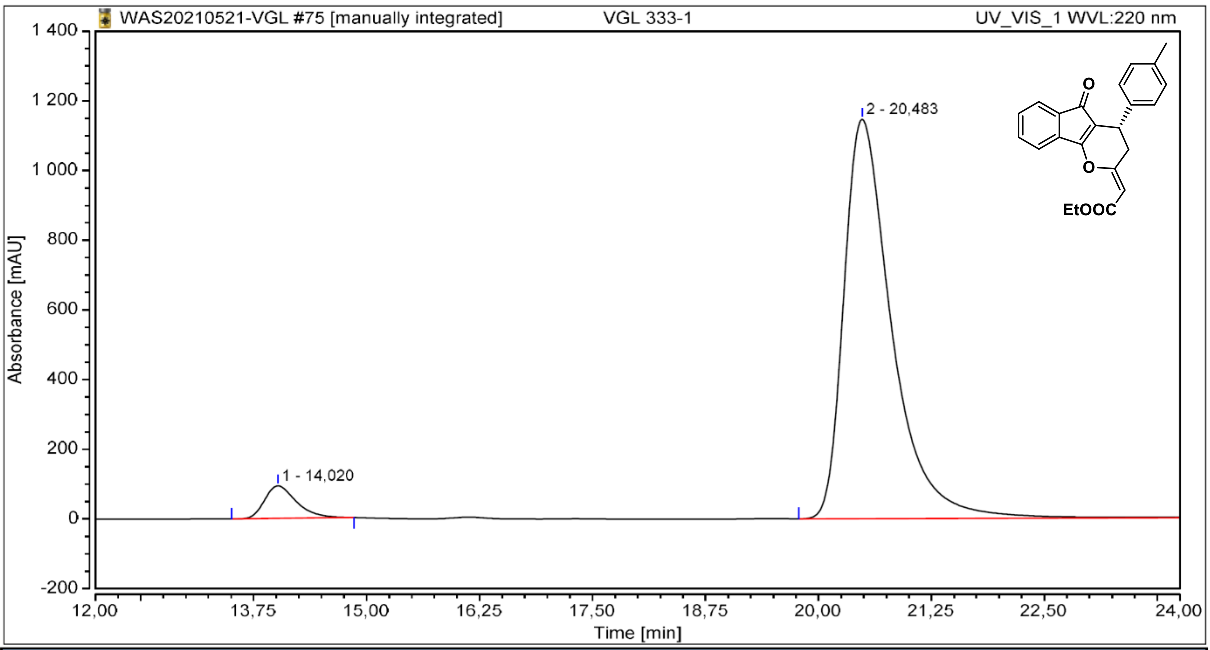

Integration Results							
No.	Peak Name	Retention Time min	Area mAU*min	Height mAU	Relative Area %	Relative Height %	Amount n.a.
1		14,020	38,326	93,555	5,15	7,55	n.a.
2		20,483	706,010	1146,373	94,85	92,45	n.a.
**Total:**			**744,336**	**1239,928**	**100,00**	**100,00**	

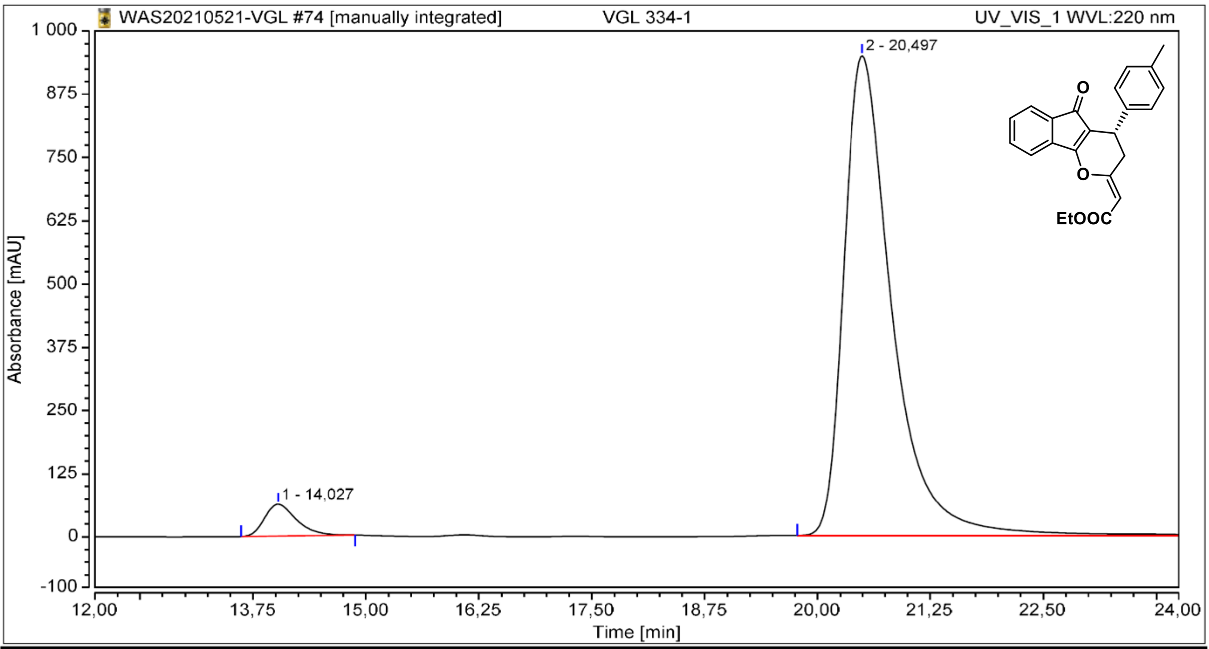

Integration Results

No.	Peak Name	Retention Time min	Area mAU*min	Height mAU	Relative Area %	Relative Height %	Amount n.a.
1		14,027	25,774	63,007	4,17	6,23	n.a.
2		20,497	592,084	948,365	95,83	93,77	n.a.
**Total:**			**617,859**	**1011,371**	**100,00**	**100,00**	

Chromatograms of **3f**

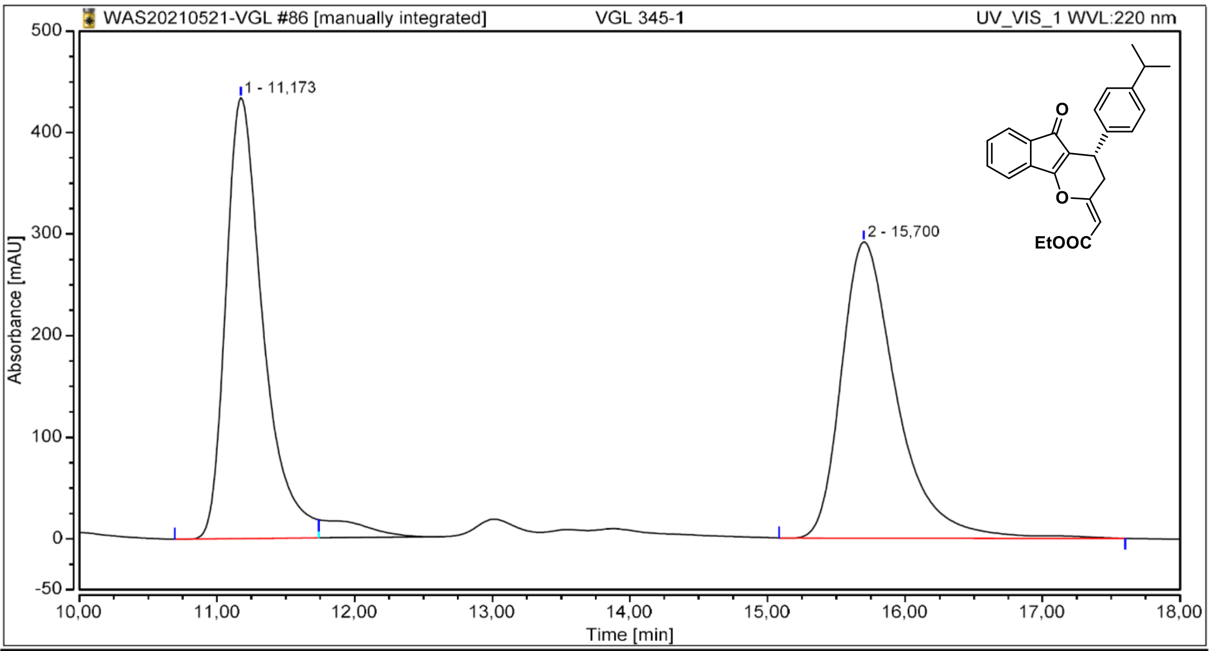

Integration Results							
No.	Peak Name	Retention Time min	Area mAU*min	Height mAU	Relative Area %	Relative Height %	Amount n.a.
1		11,173	135,421	434,130	49,88	59,84	n.a.
2		15,700	136,085	291,401	50,12	40,16	n.a.
**Total:**			**271,506**	**725,531**	**100,00**	**100,00**	

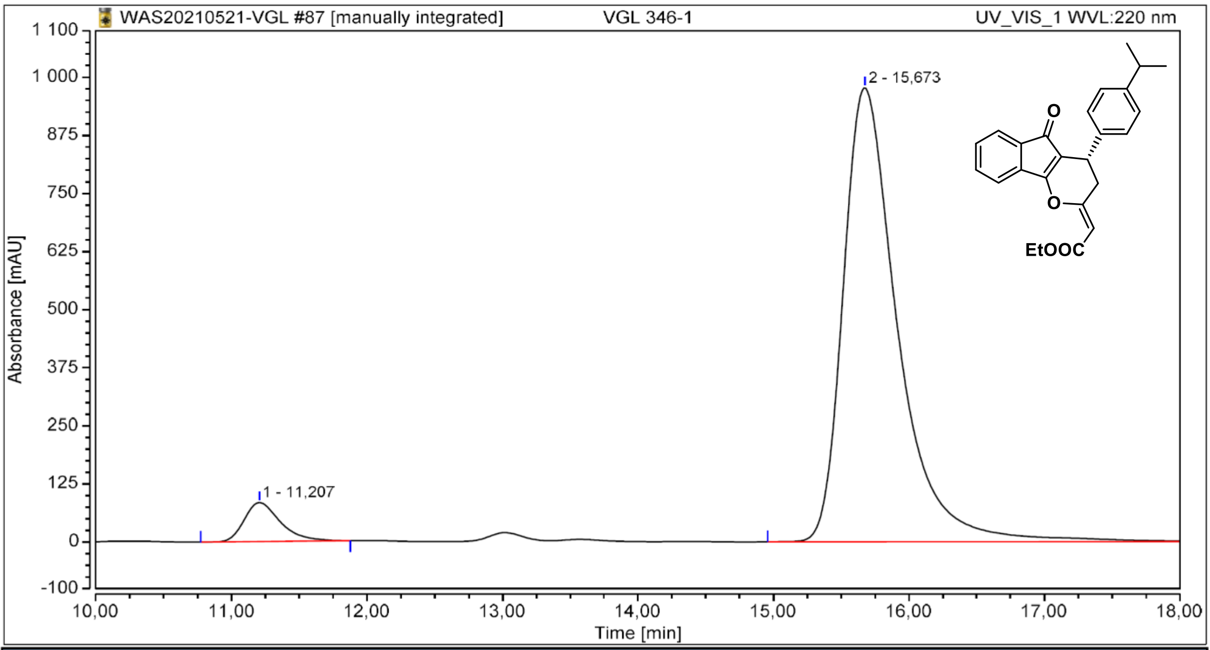

Integration Results							
No.	Peak Name	Retention Time min	Area mAU*min	Height mAU	Relative Area %	Relative Height %	Amount n.a.
1		11,207	25,825	83,649	5,38	7,89	n.a.
2		15,673	454,185	976,553	94,62	92,11	n.a.
**Total:**			**480,010**	**1060,202**	**100,00**	**100,00**	

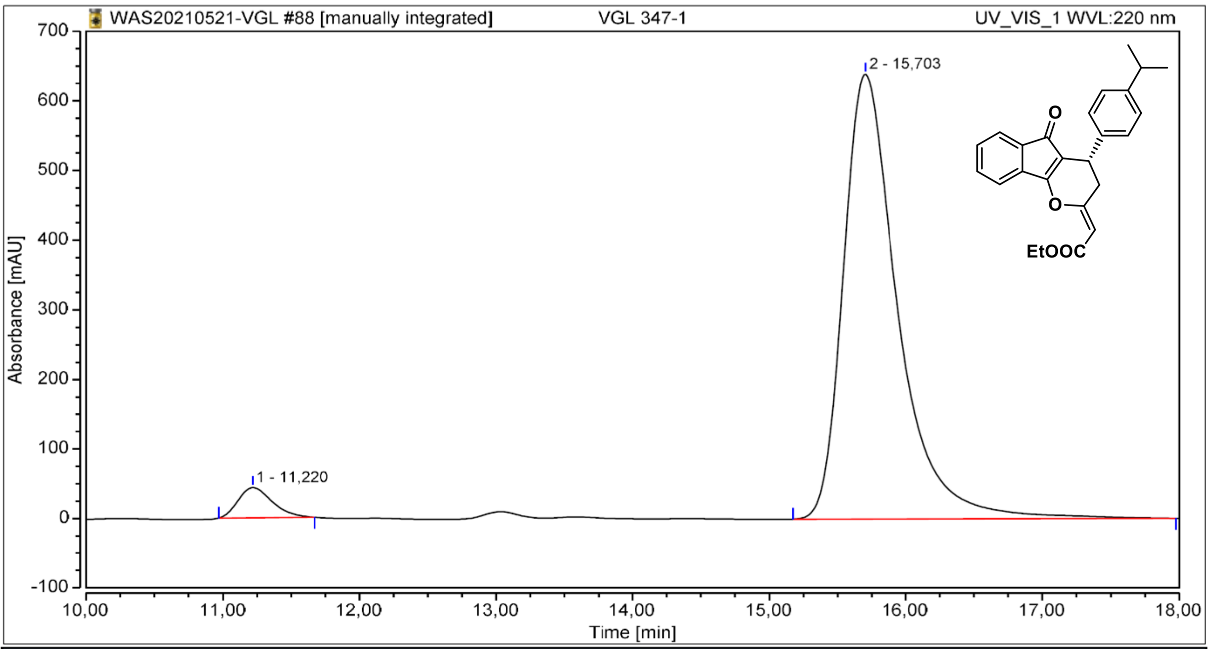

Integration Results							
No.	Peak Name	Retention Time min	Area mAU*min	Height mAU	Relative Area %	Relative Height %	Amount n.a.
1		11,220	12,532	43,364	4,06	6,35	n.a.
2		15,703	296,198	639,106	95,94	93,65	n.a.
**Total:**			**308,730**	**682,470**	**100,00**	**100,00**	

Chromatograms of **3g**

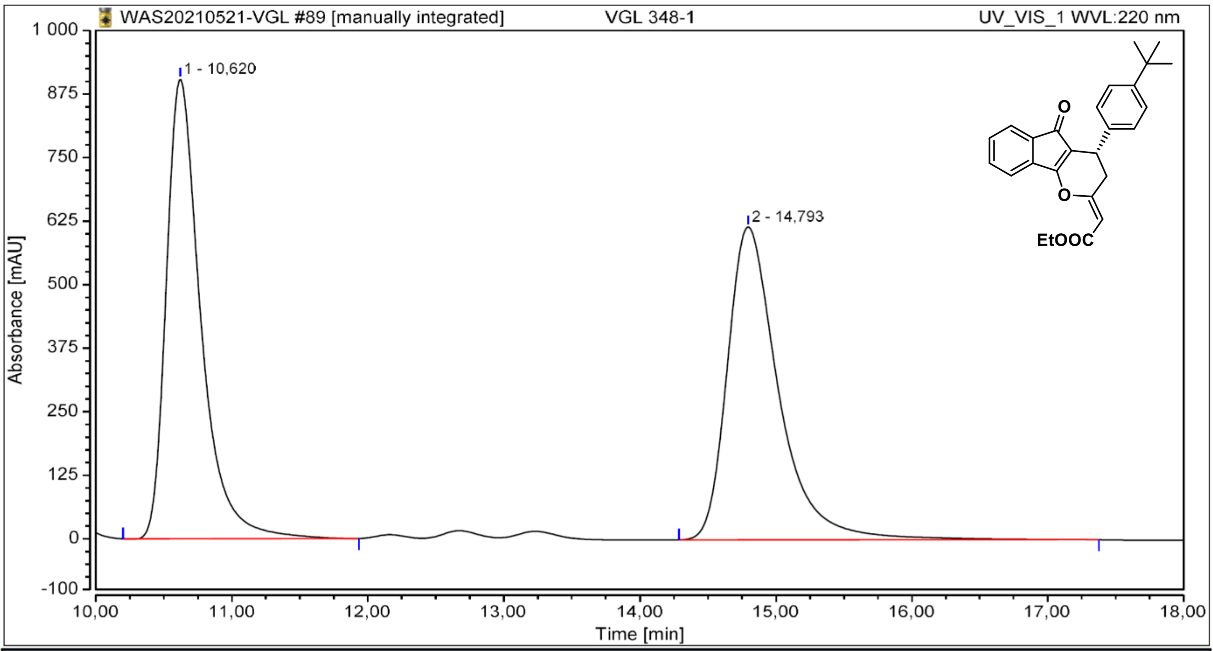

Integration Results							
No.	Peak Name	Retention Time min	Area mAU*min	Height mAU	Relative Area %	Relative Height %	Amount n.a.
1		10,620	266,123	904,826	49,67	59,51	n.a.
2		14,793	269,666	615,748	50,33	40,49	n.a.
**Total:**			**535,789**	**1520,574**	**100,00**	**100,00**	

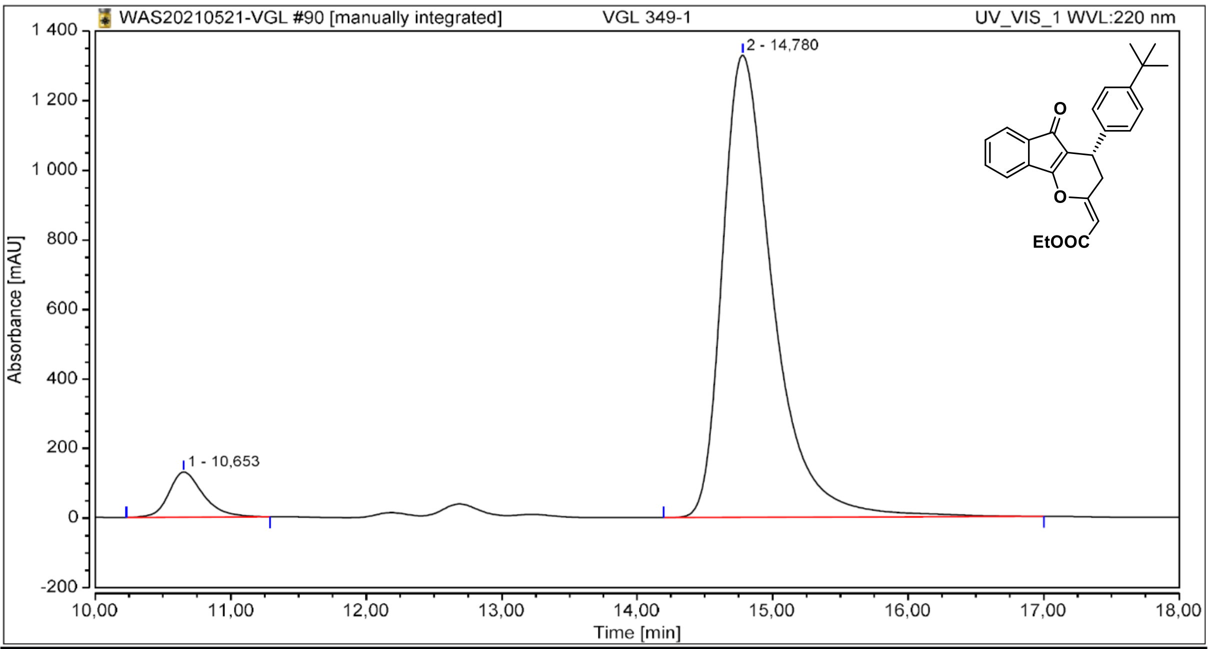

Integration Results							
No.	Peak Name	Retention Time min	Area mAU*min	Height mAU	Relative Area %	Relative Height %	Amount n.a.
1		10,653	38,076	129,786	6,21	8,90	n.a.
2		14,780	575,457	1328,550	93,79	91,10	n.a.
**Total:**			**613,533**	**1458,336**	**100,00**	**100,00**	

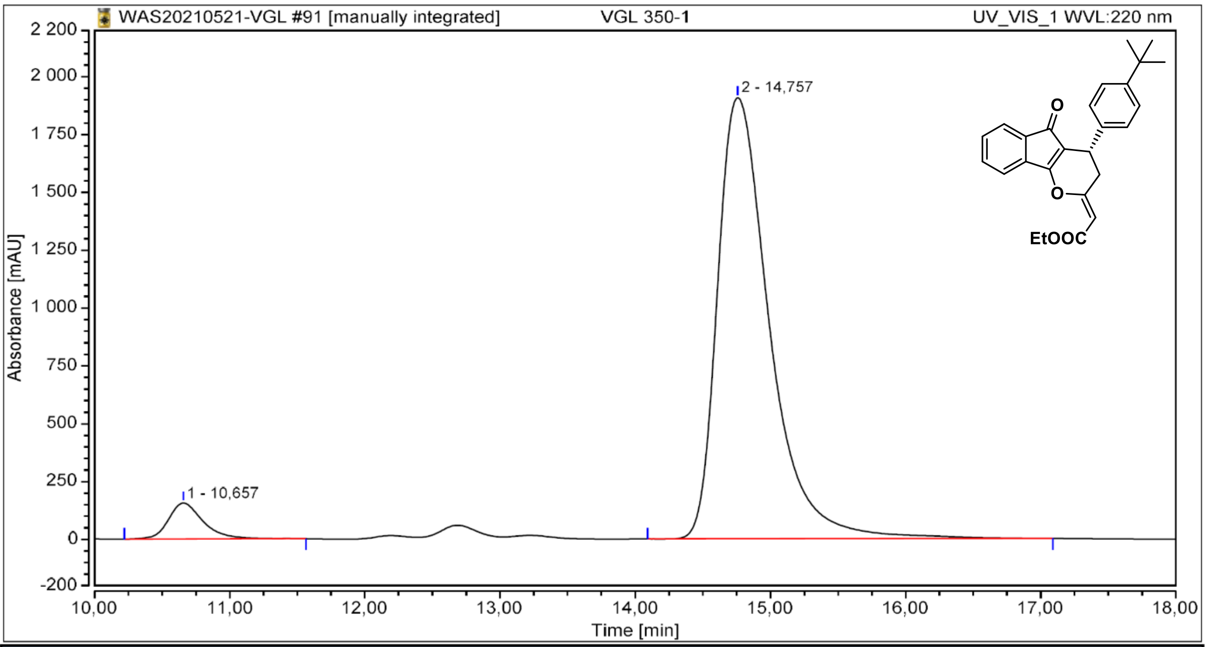

Integration Results							
No.	Peak Name	Retention Time min	Area mAU*min	Height mAU	Relative Area %	Relative Height %	Amount n.a.
1		10,657	46,734	155,377	5,22	7,53	n.a.
2		14,757	848,919	1907,106	94,78	92,47	n.a.
**Total:**			**895,653**	**2062,483**	**100,00**	**100,00**	

Chromatograms of **3h**

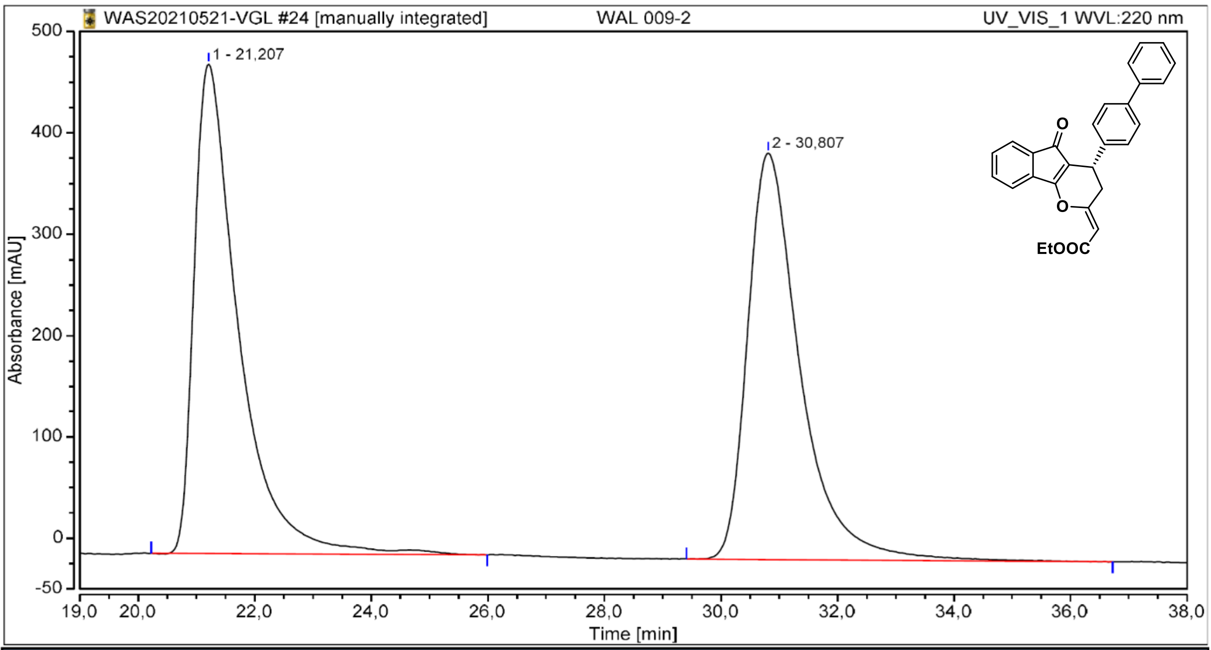

Integration Results							
No.	Peak Name	Retention Time min	Area mAU*min	Height mAU	Relative Area %	Relative Height %	Amount n.a.
1		21,207	425,481	482,803	50,37	54,63	n.a.
2		30,807	419,294	401,021	49,63	45,37	n.a.
**Total:**			**844,776**	**883,824**	**100,00**	**100,00**	

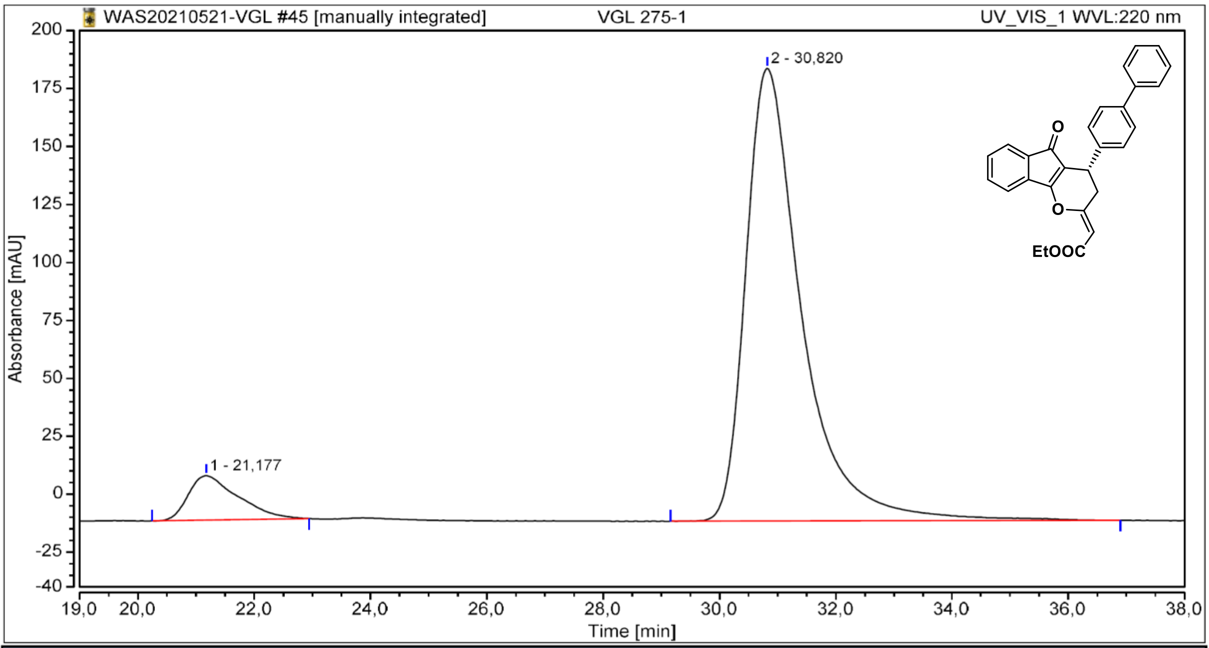

Integration Results							
No.	Peak Name	Retention Time min	Area mAU*min	Height mAU	Relative Area %	Relative Height %	Amount n.a.
1		21,177	18,864	19,162	7,97	8,94	n.a.
2		30,820	217,820	195,178	92,03	91,06	n.a.
**Total:**			**236,684**	**214,340**	**100,00**	**100,00**	

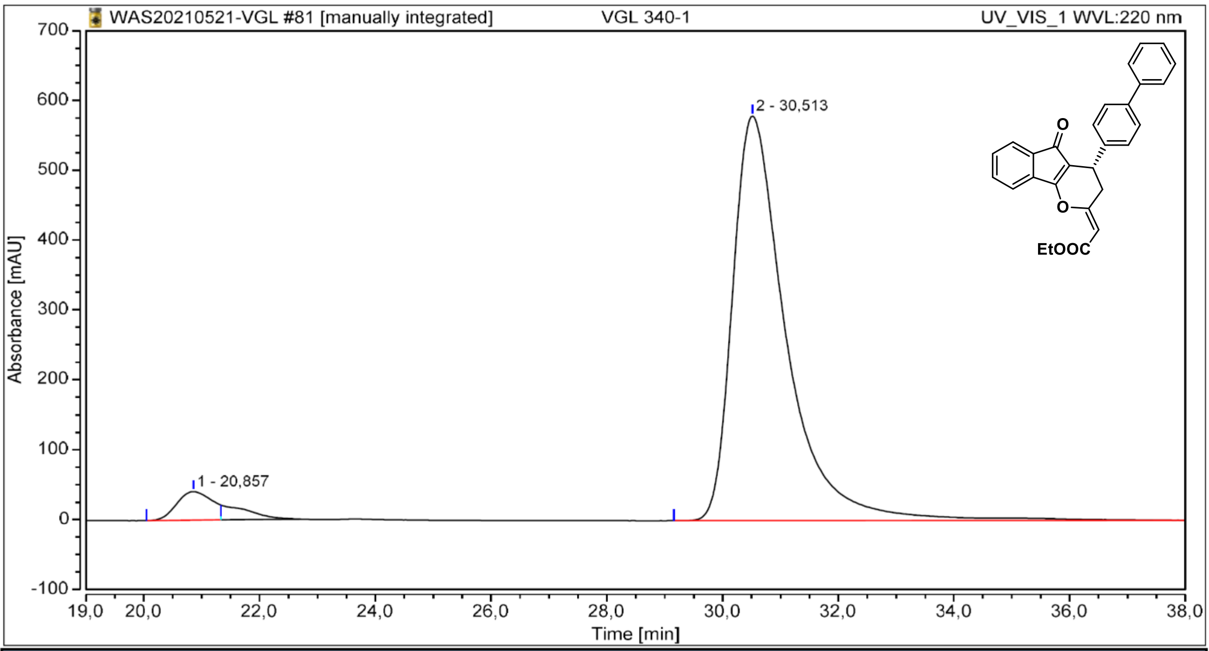

Integration Results							
No.	Peak Name	Retention Time min	Area mAU*min	Height mAU	Relative Area %	Relative Height %	Amount n.a.
1		20,857	28,815	40,848	4,42	6,59	n.a.
2		30,513	623,285	579,355	95,58	93,41	n.a.
**Total:**			**652,101**	**620,203**	**100,00**	**100,00**	

Chromatograms of **3i**

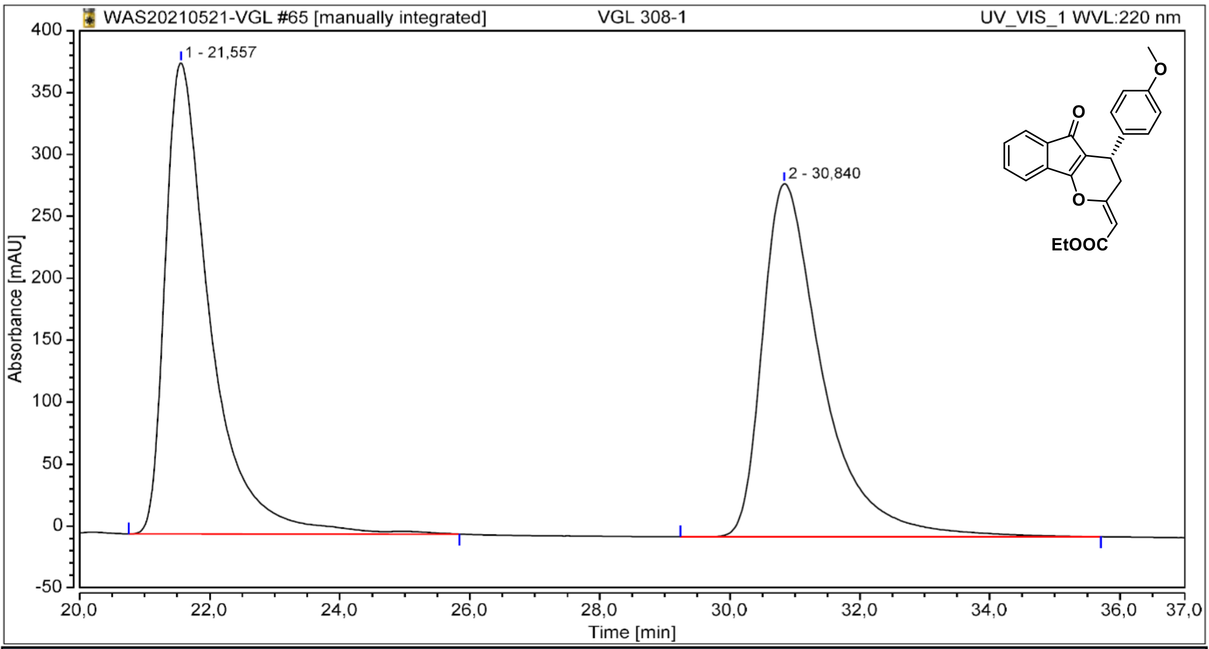

Integration Results							
No.	Peak Name	Retention Time min	Area mAU*min	Height mAU	Relative Area %	Relative Height %	Amount n.a.
1		21,557	306,560	380,376	50,15	57,17	n.a.
2		30,840	304,720	284,944	49,85	42,83	n.a.
**Total:**			**611,280**	**665,320**	**100,00**	**100,00**	

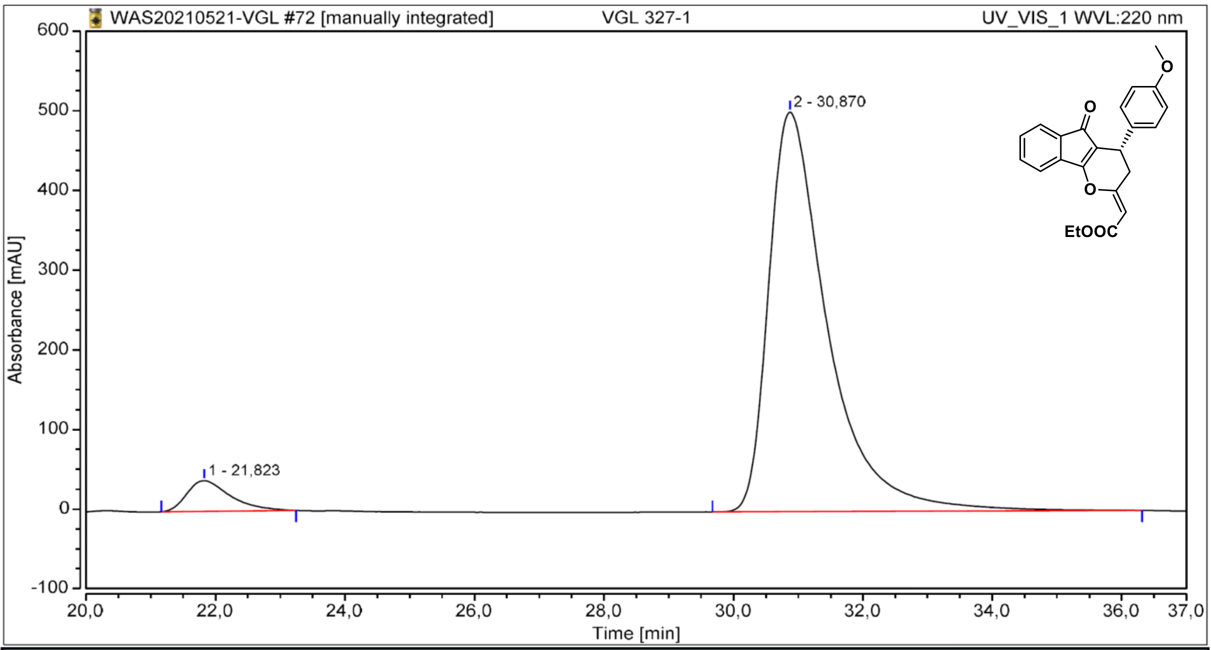

Integration Results							
No.	Peak Name	Retention Time min	Area mAU*min	Height mAU	Relative Area %	Relative Height %	Amount n.a.
1		21,823	29,971	38,531	5,37	7,14	n.a.
2		30,870	527,666	501,378	94,63	92,86	n.a.
**Total:**			**557,637**	**539,908**	**100,00**	**100,00**	

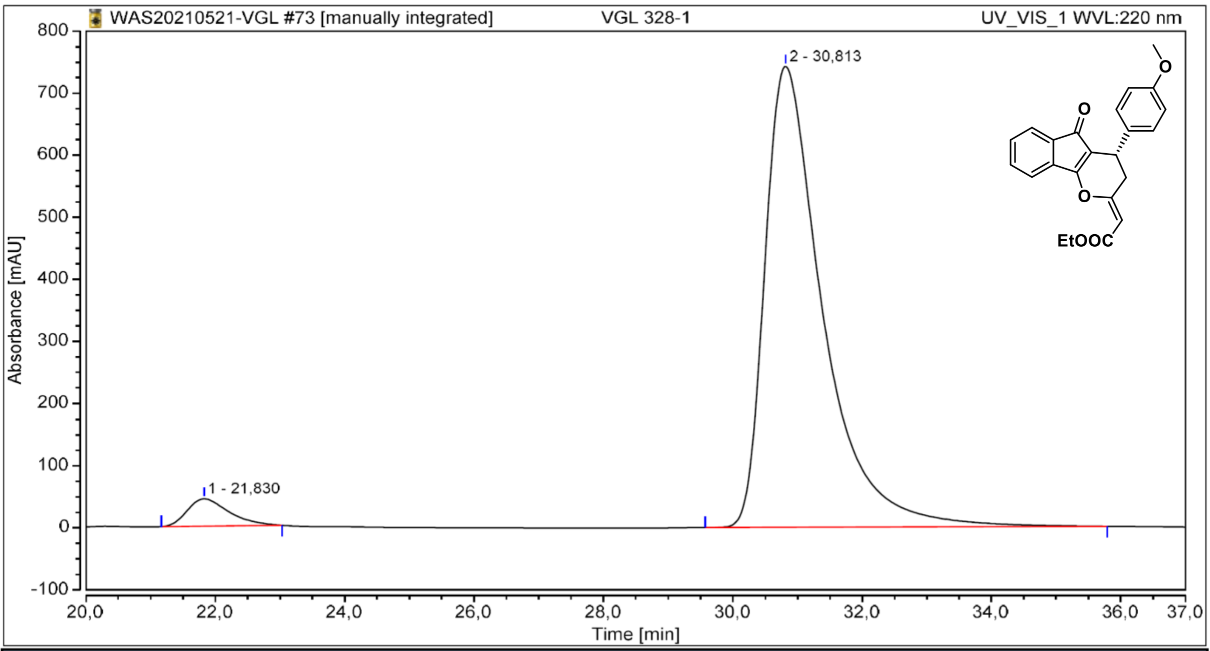

Integration Results							
No.	Peak Name	Retention Time min	Area mAU*min	Height mAU	Relative Area %	Relative Height %	Amount n.a.
1		21,830	33,088	44,124	4,13	5,61	n.a.
2		30,813	768,850	742,263	95,87	94,39	n.a.
**Total:**			**801,938**	**786,387**	**100,00**	**100,00**	

Chromatograms of **3j**

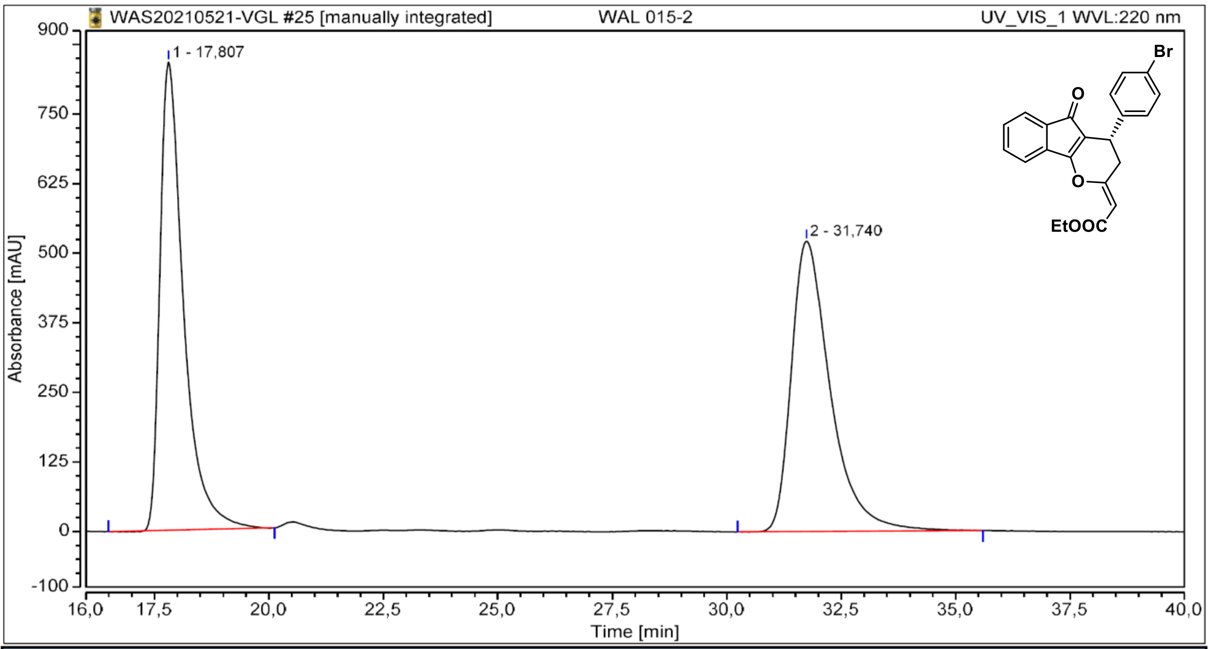

Integration Results							
No.	Peak Name	Retention Time min	Area mAU*min	Height mAU	Relative Area %	Relative Height %	Amount n.a.
1		17,807	502,122	841,329	49,35	61,71	n.a.
2		31,740	515,347	522,041	50,65	38,29	n.a.
**Total:**			**1017,470**	**1363,370**	**100,00**	**100,00**	

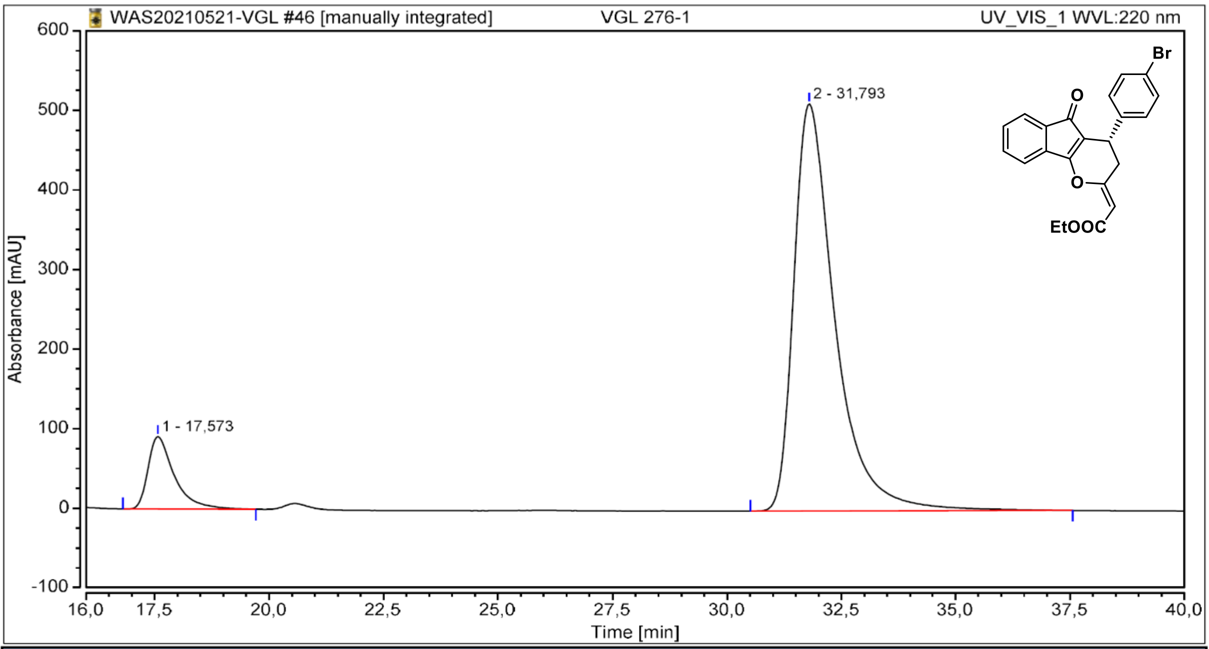

Integration Results							
No.	Peak Name	Retention Time min	Area mAU*min	Height mAU	Relative Area %	Relative Height %	Amount n.a.
1		17,573	60,467	90,919	10,18	15,09	n.a.
2		31,793	533,520	511,454	89,82	84,91	n.a.
**Total:**			**593,987**	**602,373**	**100,00**	**100,00**	

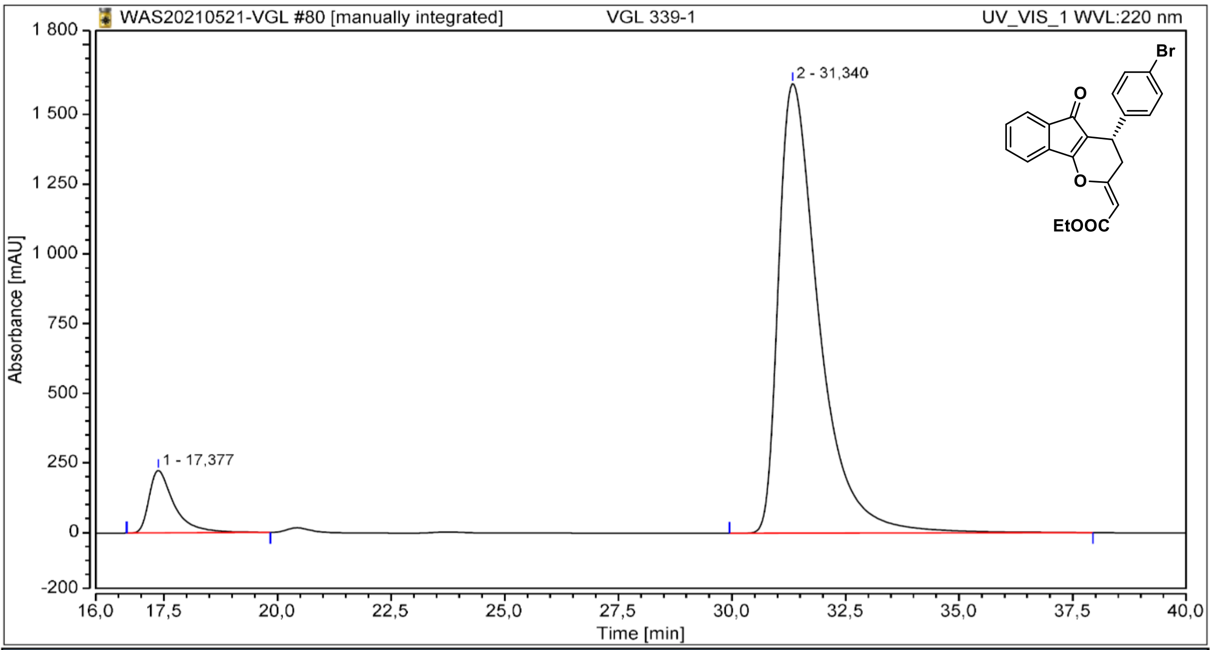

Integration Results							
No.	Peak Name	Retention Time min	Area mAU*min	Height mAU	Relative Area %	Relative Height %	Amount n.a.
1		17,377	143,771	223,978	7,95	12,19	n.a.
2		31,340	1665,477	1612,884	92,05	87,81	n.a.
**Total:**			**1809,248**	**1836,862**	**100,00**	**100,00**	

Chromatograms of **3k**

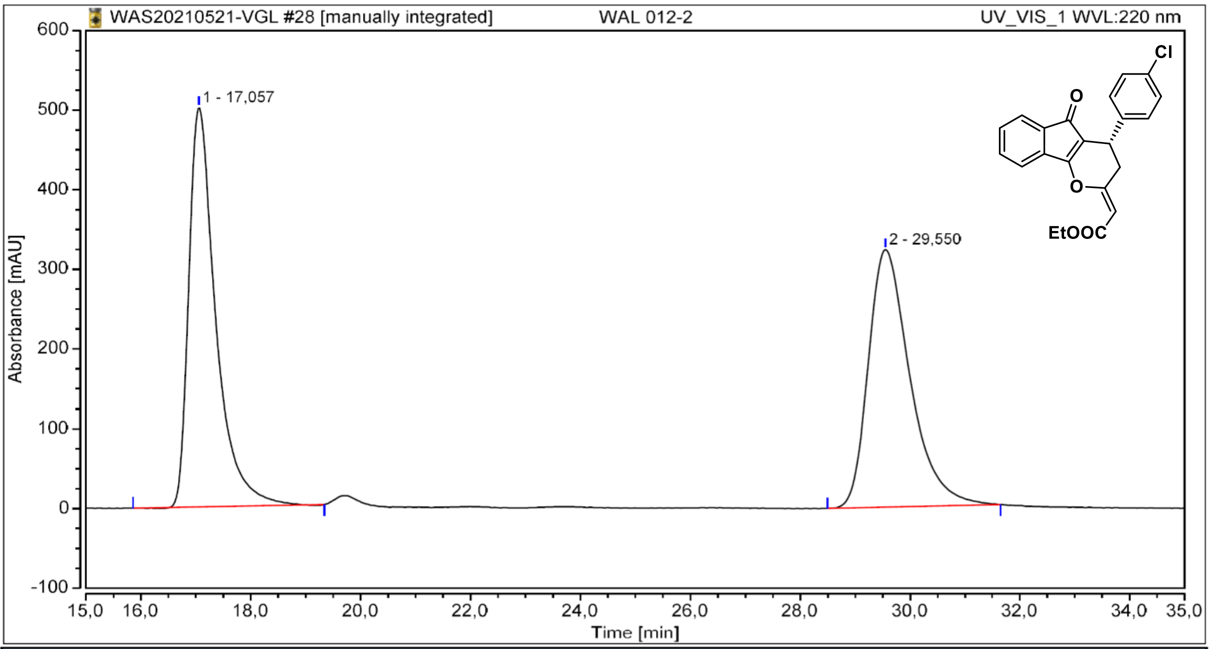

Integration Results							
No.	Peak Name	Retention Time min	Area mAU*min	Height mAU	Relative Area %	Relative Height %	Amount n.a.
1		17,057	283,011	501,392	49,96	60,81	n.a.
2		29,550	283,477	323,082	50,04	39,19	n.a.
**Total:**			**566,487**	**824,474**	**100,00**	**100,00**	

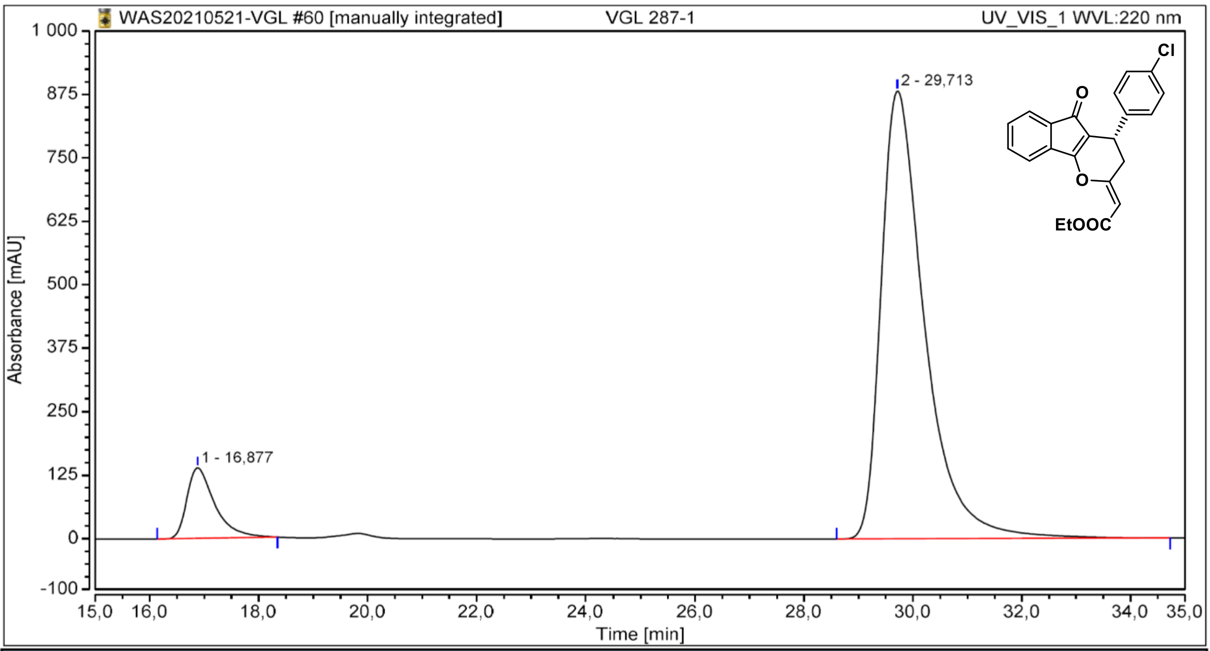

Integration Results							
No.	Peak Name	Retention Time min	Area mAU*min	Height mAU	Relative Area %	Relative Height %	Amount n.a.
1		16,877	82,559	139,217	9,19	13,63	n.a.
2		29,713	815,881	882,547	90,81	86,37	n.a.
**Total:**			**898,440**	**1021,764**	**100,00**	**100,00**	

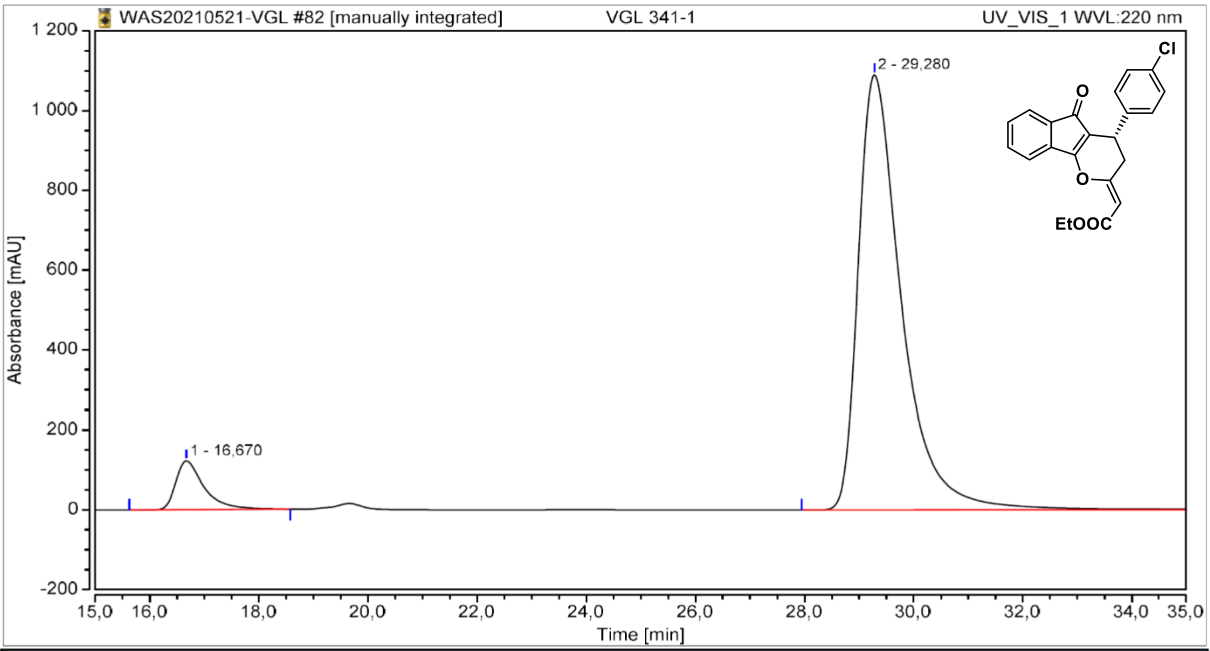

Integration Results							
No.	Peak Name	Retention Time min	Area mAU*min	Height mAU	Relative Area %	Relative Height %	Amount n.a.
1		16,670	73,540	122,587	6,82	10,11	n.a.
2		29,280	1004,490	1090,366	93,18	89,89	n.a.
**Total:**			**1078,030**	**1212,953**	**100,00**	**100,00**	

Chromatograms of enantioenriched **3l**

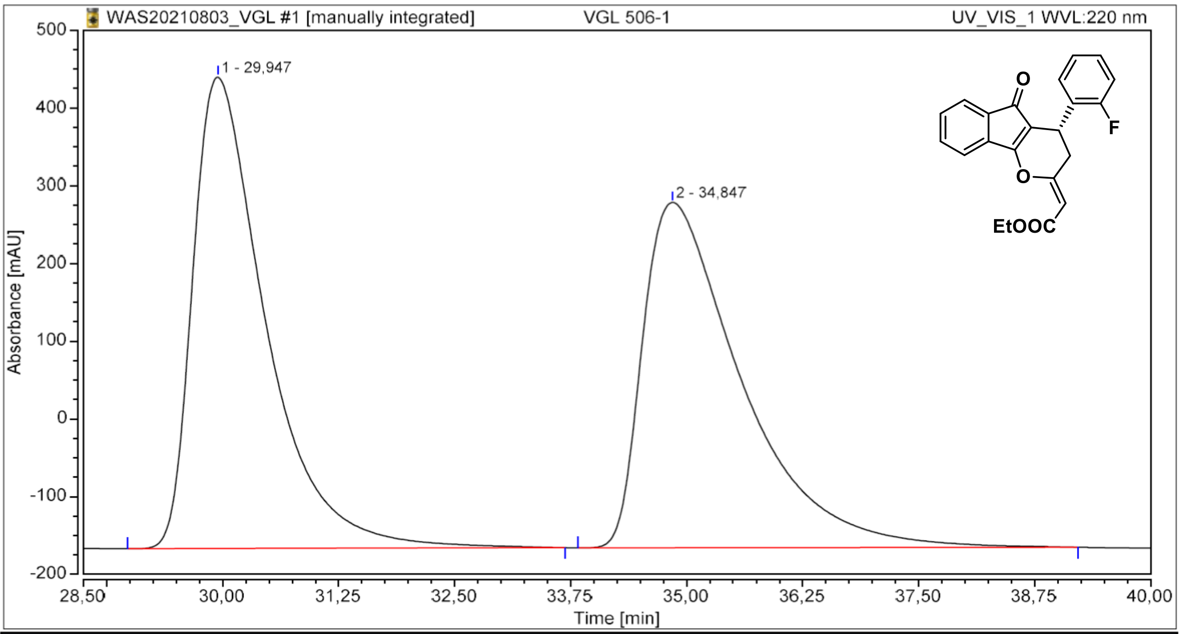

Integration Results							
No.	Peak Name	Retention Time min	Area mAU*min	Height mAU	Relative Area %	Relative Height %	Amount n.a.
1		29,947	552,647	606,432	50,20	57,71	n.a.
2		34,847	548,352	444,373	49,80	42,29	n.a.
**Total:**			**1100,999**	**1050,806**	**100,00**	**100,00**	

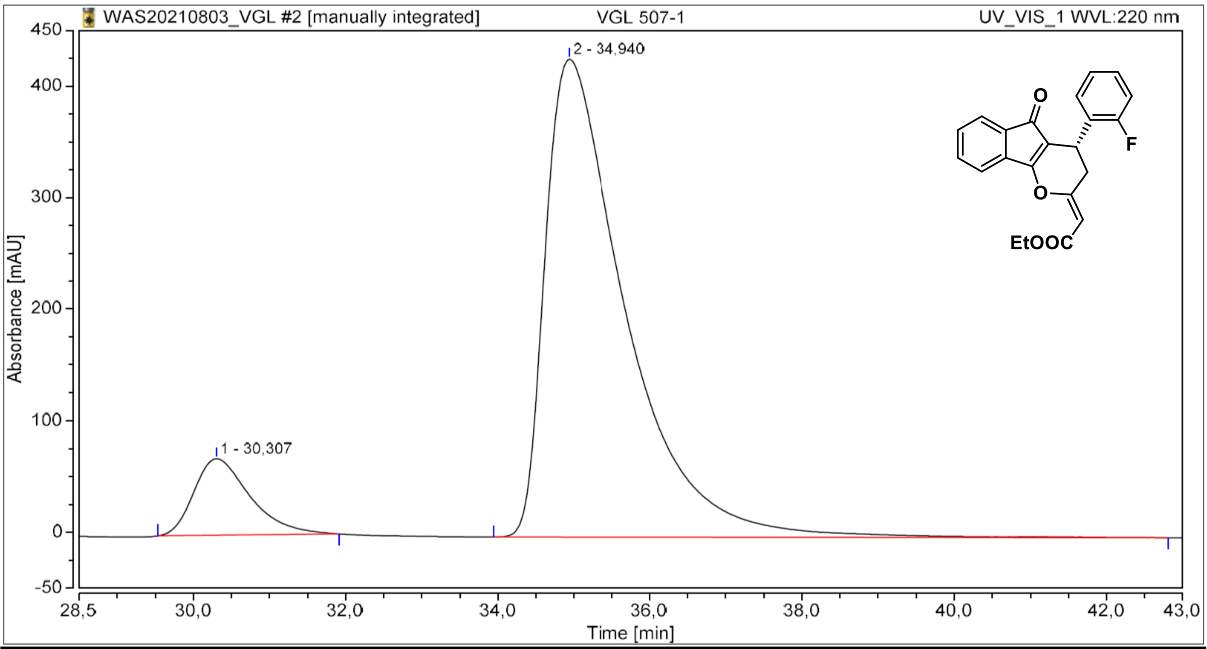

Integration Results							
No.	Peak Name	Retention Time min	Area mAU*min	Height mAU	Relative Area %	Relative Height %	Amount n.a.
1		30,307	58,584	68,719	10,01	13,82	n.a.
2		34,940	526,457	428,482	89,99	86,18	n.a.
**Total:**			**585,041**	**497,201**	**100,00**	**100,00**	

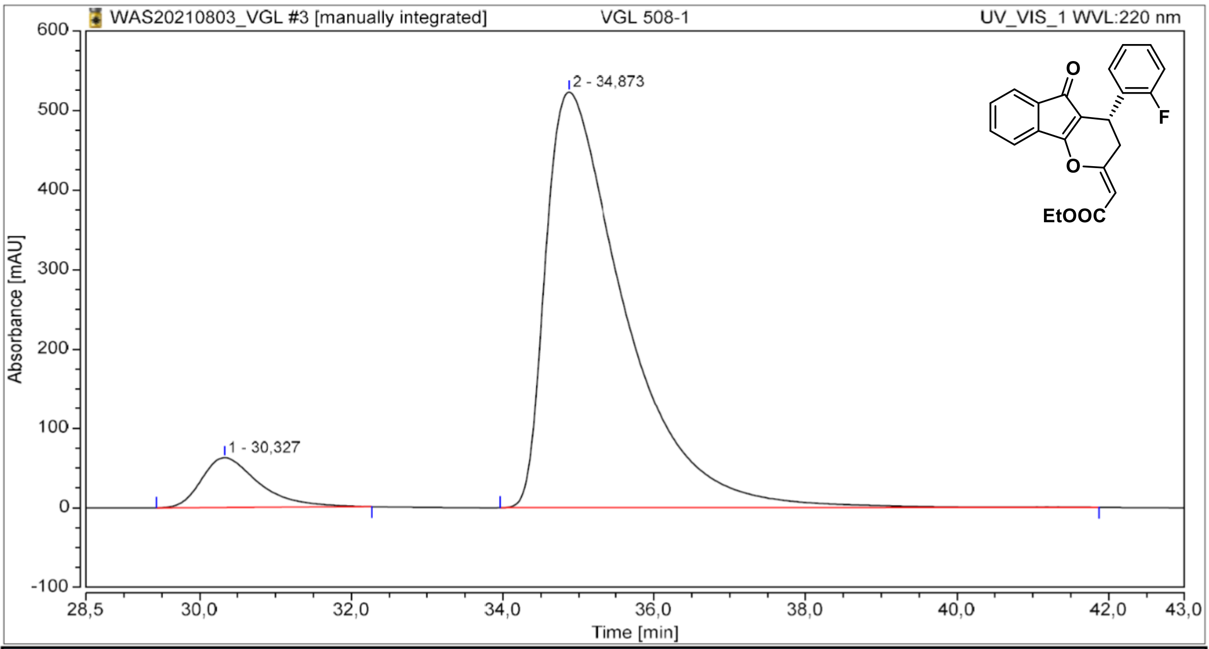

Integration Results							
No.	Peak Name	Retention Time min	Area mAU*min	Height mAU	Relative Area %	Relative Height %	Amount n.a.
1		30,327	54,883	62,572	7,92	10,69	n.a.
2		34,873	638,421	522,843	92,08	89,31	n.a.
**Total:**			**693,304**	**585,415**	**100,00**	**100,00**	

Chromatograms of **3m**

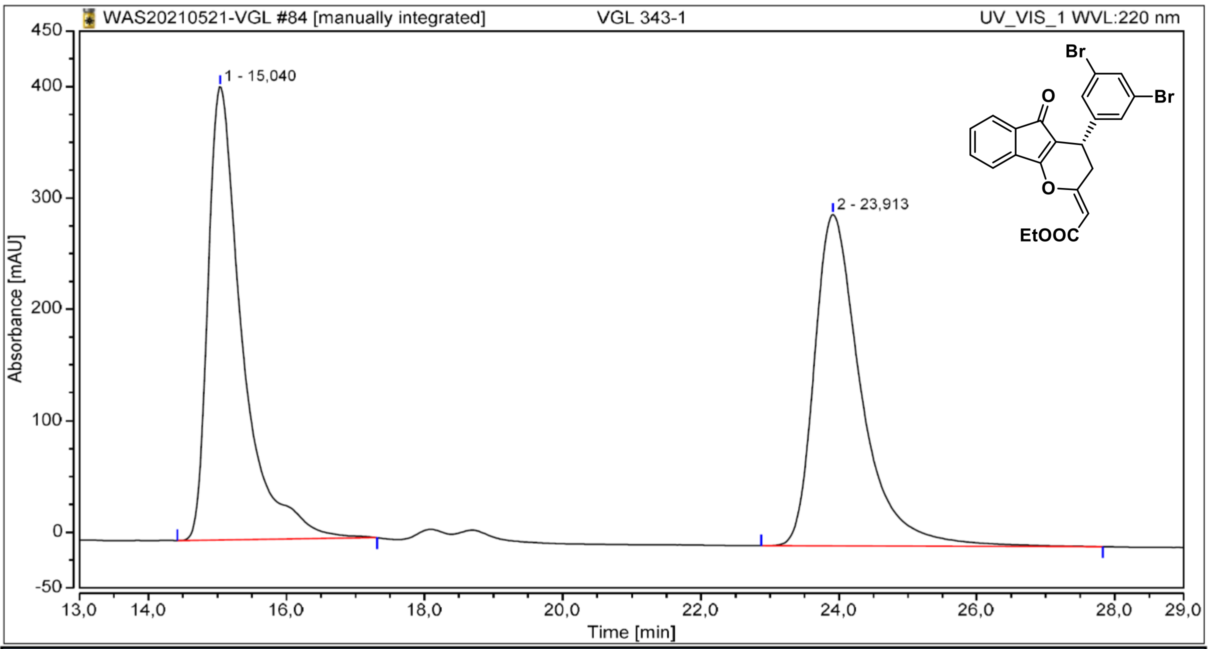

Integration Results							
No.	Peak Name	Retention Time min	Area mAU*min	Height mAU	Relative Area %	Relative Height %	Amount n.a.
1		15,040	233,503	407,206	49,97	57,79	n.a.
2		23,913	233,761	297,432	50,03	42,21	n.a.
**Total:**			**467,264**	**704,638**	**100,00**	**100,00**	

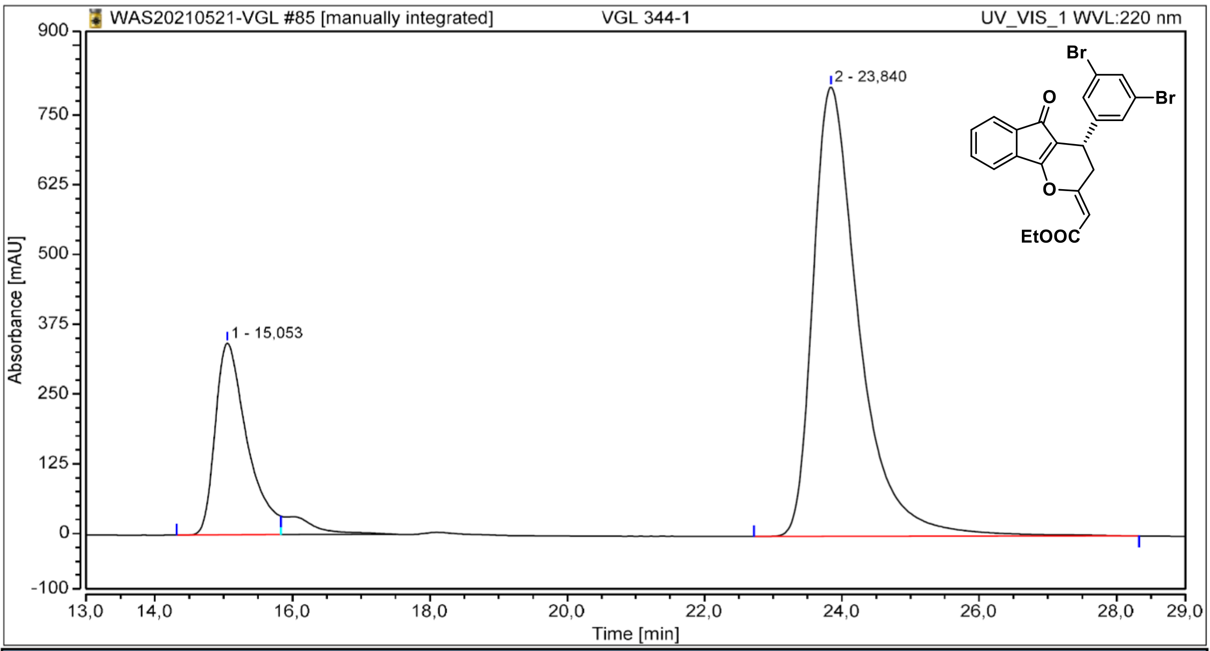

Integration Results							
No.	Peak Name	Retention Time min	Area mAU*min	Height mAU	Relative Area %	Relative Height %	Amount n.a.
1		15,053	184,110	343,393	22,79	29,90	n.a.
2		23,840	623,747	805,225	77,21	70,10	n.a.
**Total:**			**807,857**	**1148,619**	**100,00**	**100,00**	

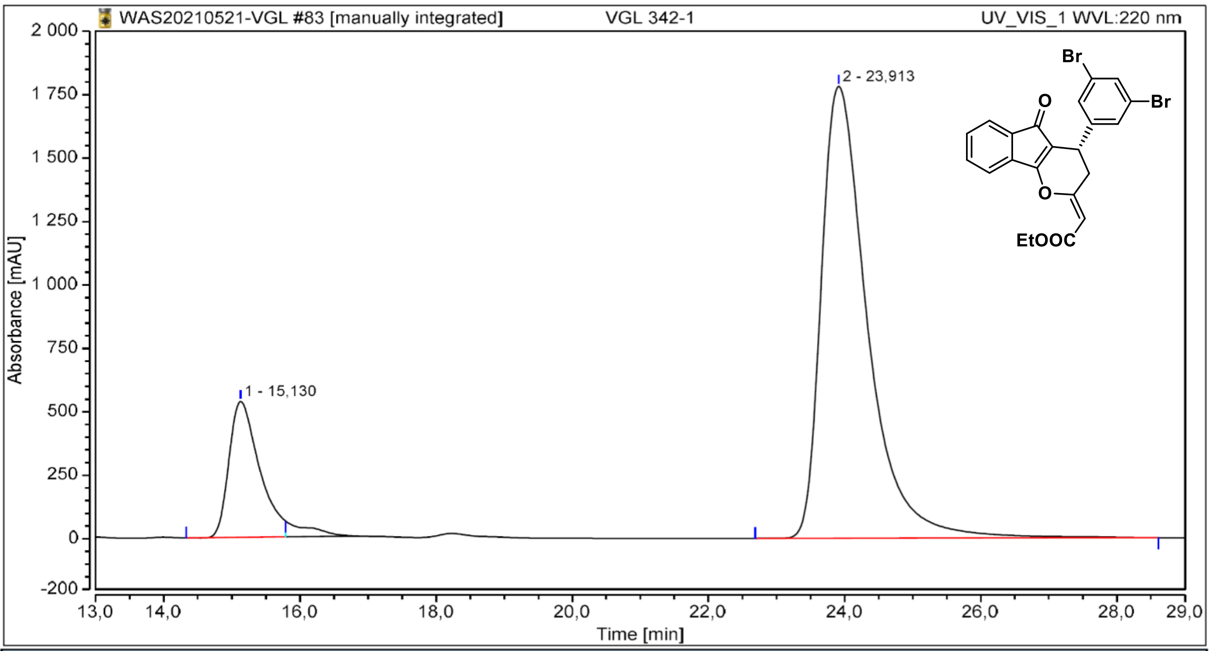

Integration Results							
No.	Peak Name	Retention Time min	Area mAU*min	Height mAU	Relative Area %	Relative Height %	Amount n.a.
1		15,130	275,277	535,593	16,46	23,12	n.a.
2		23,913	1396,873	1780,918	83,54	76,88	n.a.
**Total:**			**1672,150**	**2316,511**	**100,00**	**100,00**	

Chromatograms of **3n**

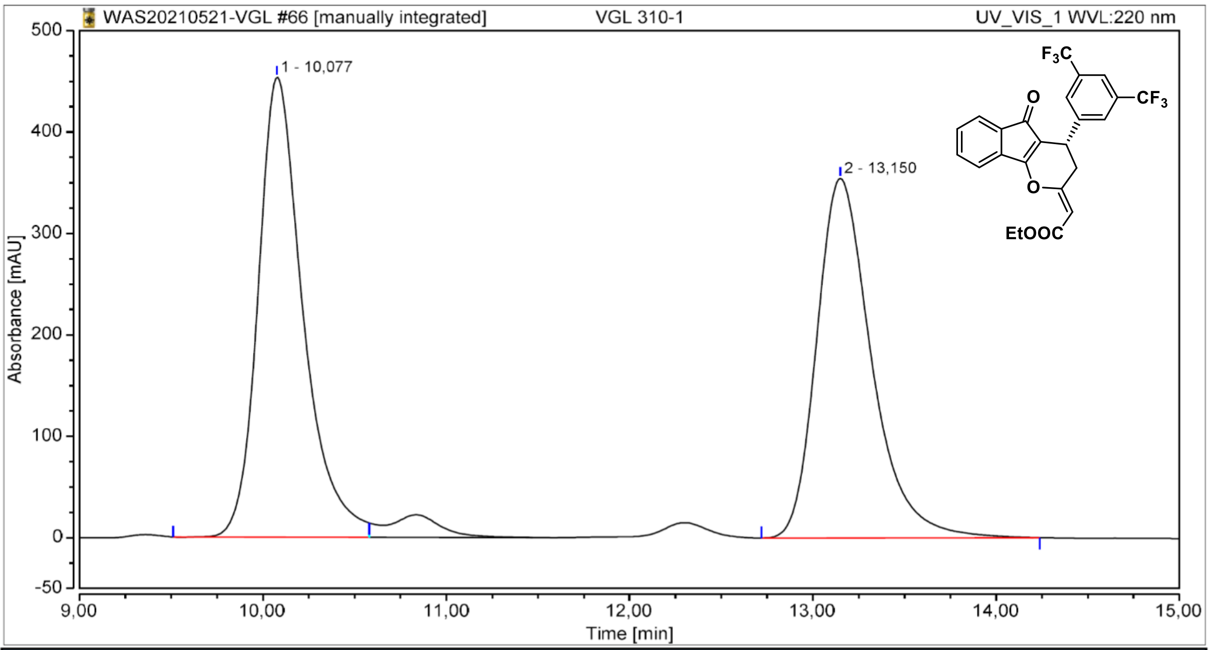

Integration Results							
No.	Peak Name	Retention Time min	Area mAU*min	Height mAU	Relative Area %	Relative Height %	Amount n.a.
1		10,077	128,286	453,351	51,14	56,13	n.a.
2		13,150	122,578	354,367	48,86	43,87	n.a.
**Total:**			**250,865**	**807,717**	**100,00**	**100,00**	

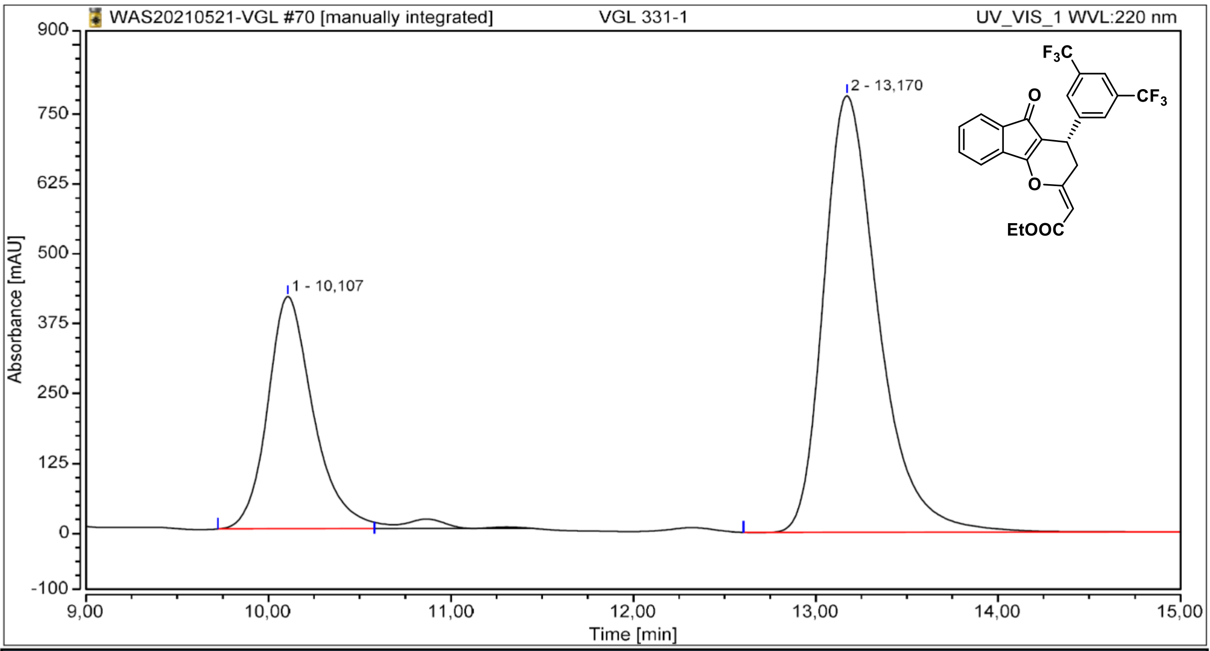

Integration Results							
No.	Peak Name	Retention Time min	Area mAU*min	Height mAU	Relative Area %	Relative Height %	Amount n.a.
1		10,107	120,463	415,226	30,69	34,70	n.a.
2		13,170	272,017	781,242	69,31	65,30	n.a.
**Total:**			**392,480**	**1196,468**	**100,00**	**100,00**	

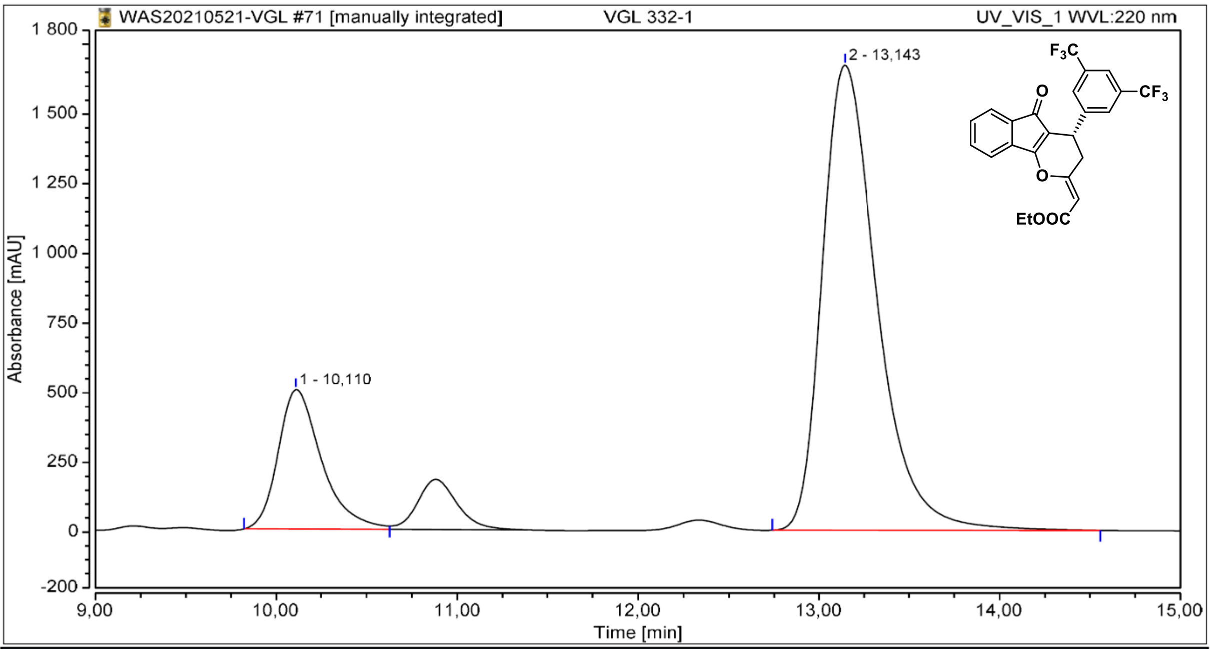

Integration Results

No.	Peak Name	Retention Time min	Area mAU*min	Height mAU	Relative Area %	Relative Height %	Amount n.a.
1		10,110	139,767	501,734	19,19	23,11	n.a.
2		13,143	588,650	1669,403	80,81	76,89	n.a.
**Total:**			**728,417**	**2171,137**	**100,00**	**100,00**	

Chromatograms of **3o**

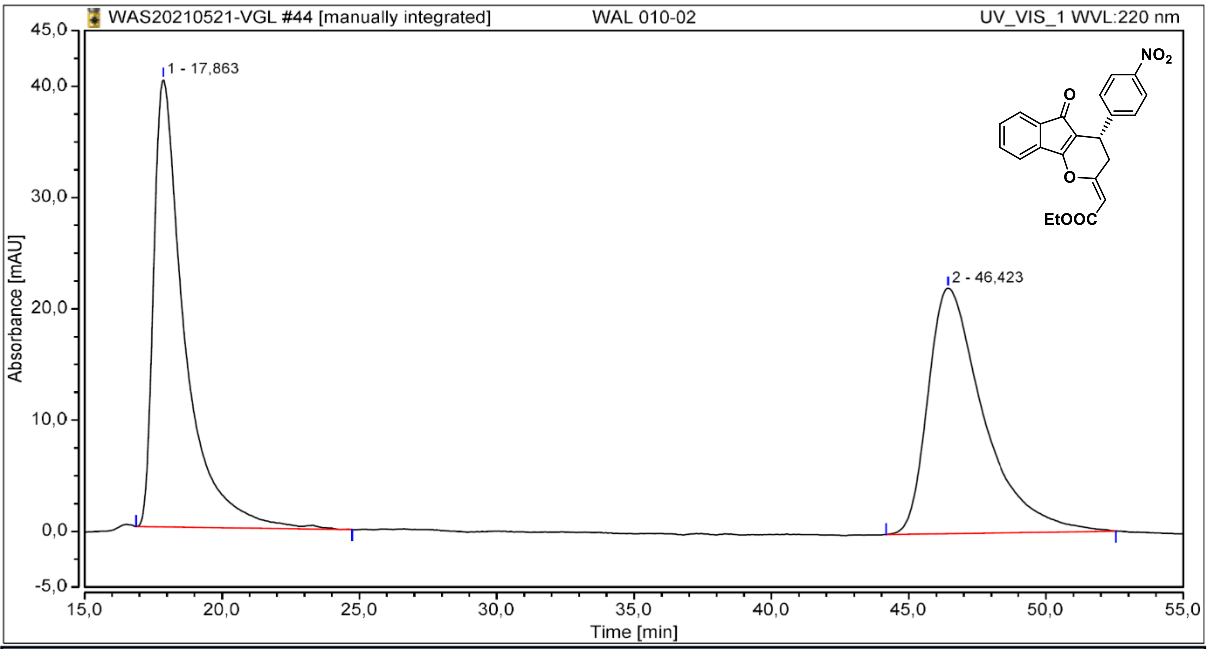

Integration Results

No.	Peak Name	Retention Time min	Area mAU*min	Height mAU	Relative Area %	Relative Height %	Amount n.a.
1		17,863	52,254	40,231	50,12	64,57	n.a.
2		46,423	52,004	22,076	49,88	35,43	n.a.
**Total:**			**104,258**	**62,307**	**100,00**	**100,00**	

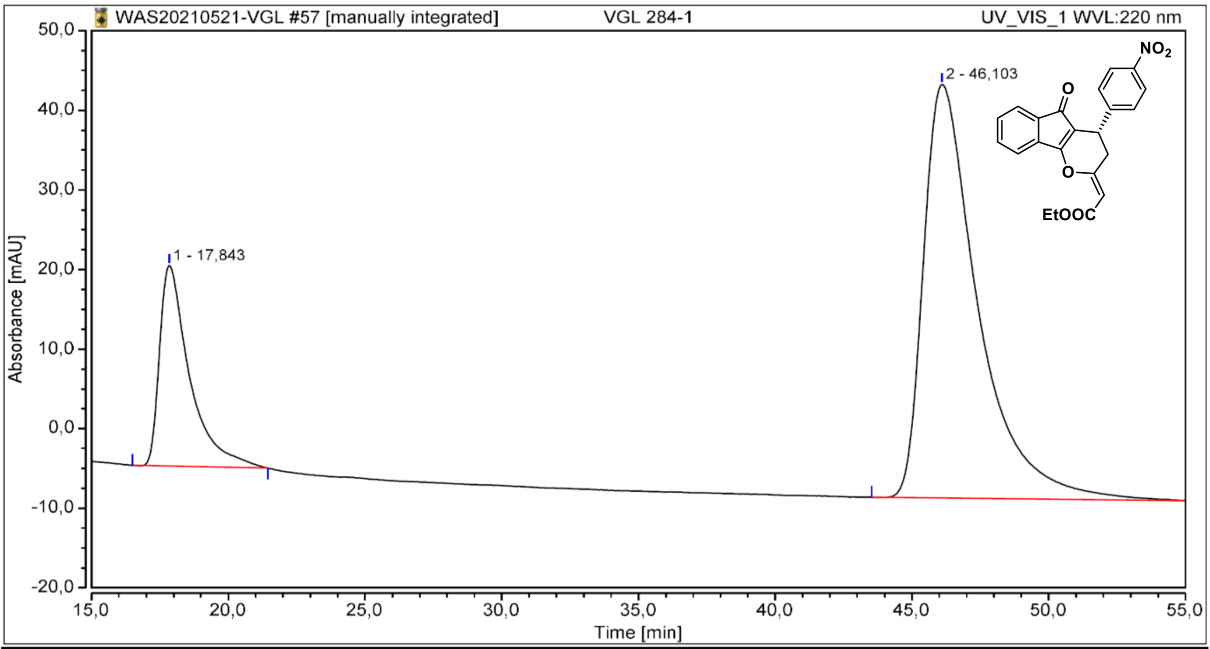

Integration Results

No.	Peak Name	Retention Time min	Area mAU*min	Height mAU	Relative Area %	Relative Height %	Amount n.a.
1		17,843	32,079	25,242	21,03	32,68	n.a.
2		46,103	120,478	51,989	78,97	67,32	n.a.
**Total:**			**152,556**	**77,231**	**100,00**	**100,00**	

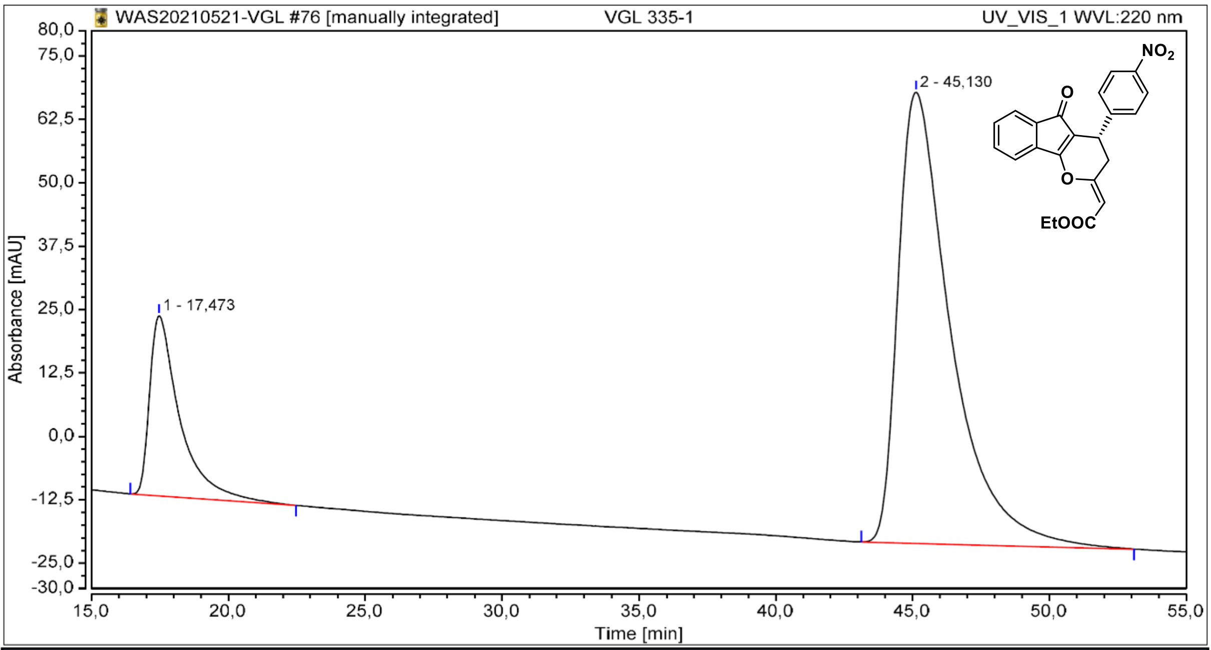

Integration Results							
No.	Peak Name	Retention Time min	Area mAU*min	Height mAU	Relative Area %	Relative Height %	Amount n.a.
1		17,473	45,395	35,549	18,61	28,55	n.a.
2		45,130	198,584	88,976	81,39	71,45	n.a.
**Total:**			**243,979**	**124,526**	**100,00**	**100,00**	

Chromatograms of **3p**

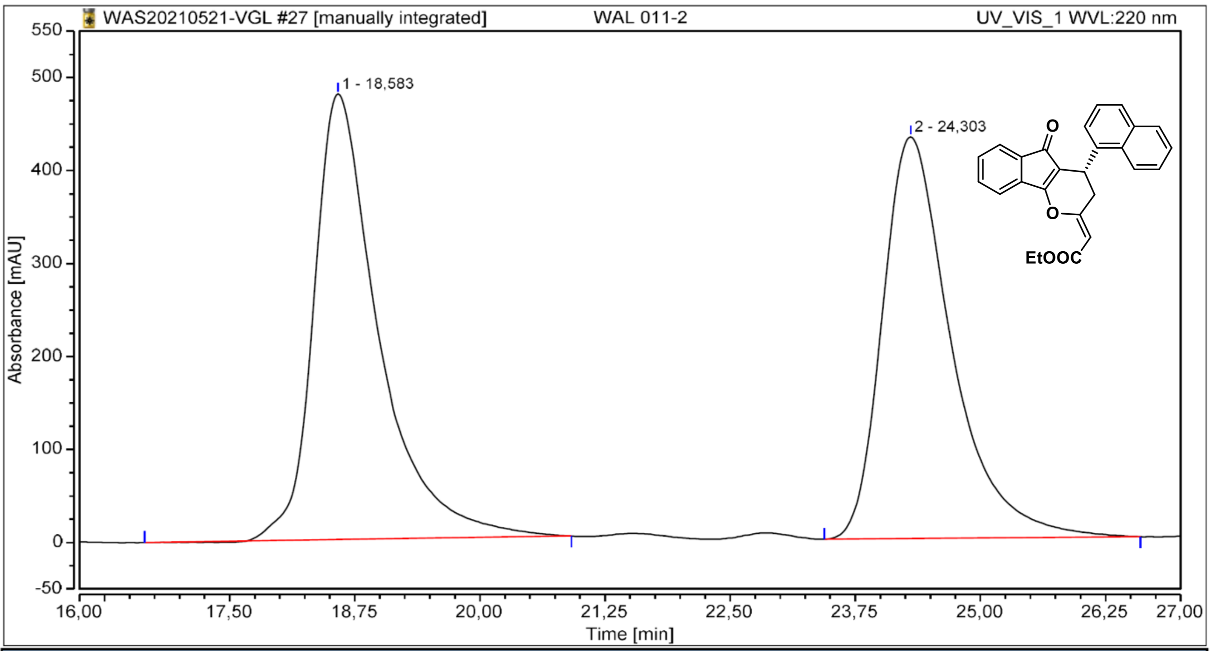

Integration Results

No.	Peak Name	Retention Time min	Area mAU*min	Height mAU	Relative Area %	Relative Height %	Amount n.a.
1		18,583	358,945	479,062	50,95	52,59	n.a.
2		24,303	345,523	431,957	49,05	47,41	n.a.
**Total:**			**704,468**	**911,019**	**100,00**	**100,00**	

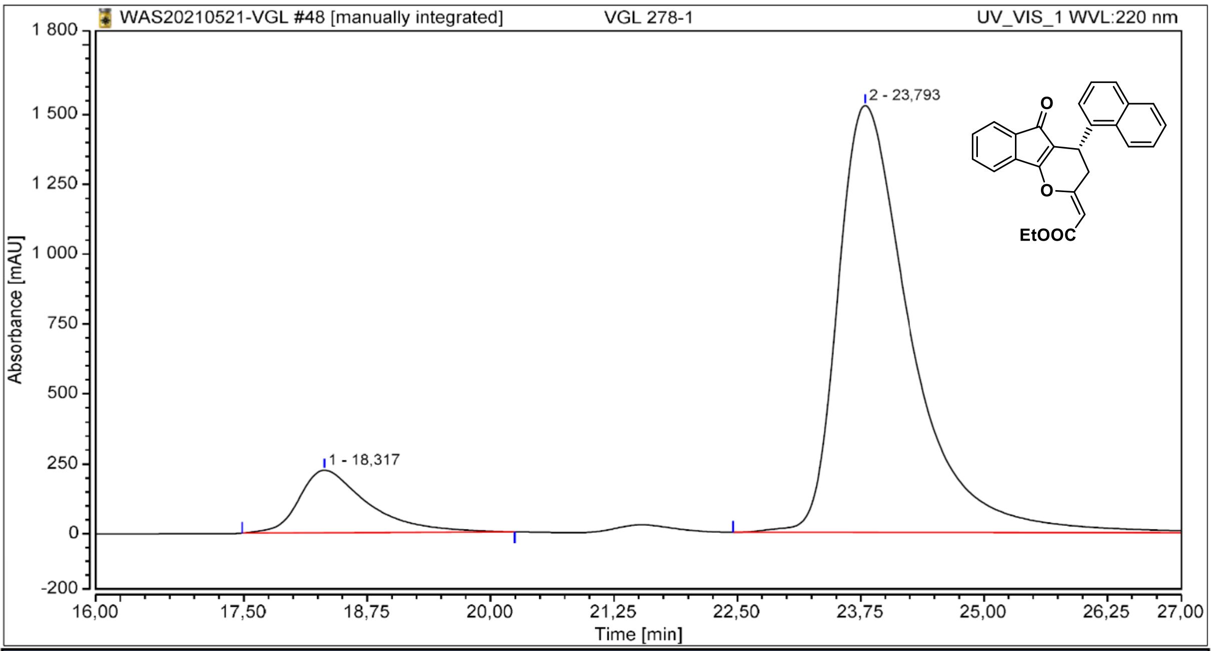

Integration Results

No.	Peak Name	Retention Time min	Area mAU*min	Height mAU	Relative Area %	Relative Height %	Amount n.a.
1		18,317	179,772	223,881	11,98	12,78	n.a.
2		23,793	1320,341	1527,266	88,02	87,22	n.a.
**Total:**			**1500,113**	**1751,147**	**100,00**	**100,00**	

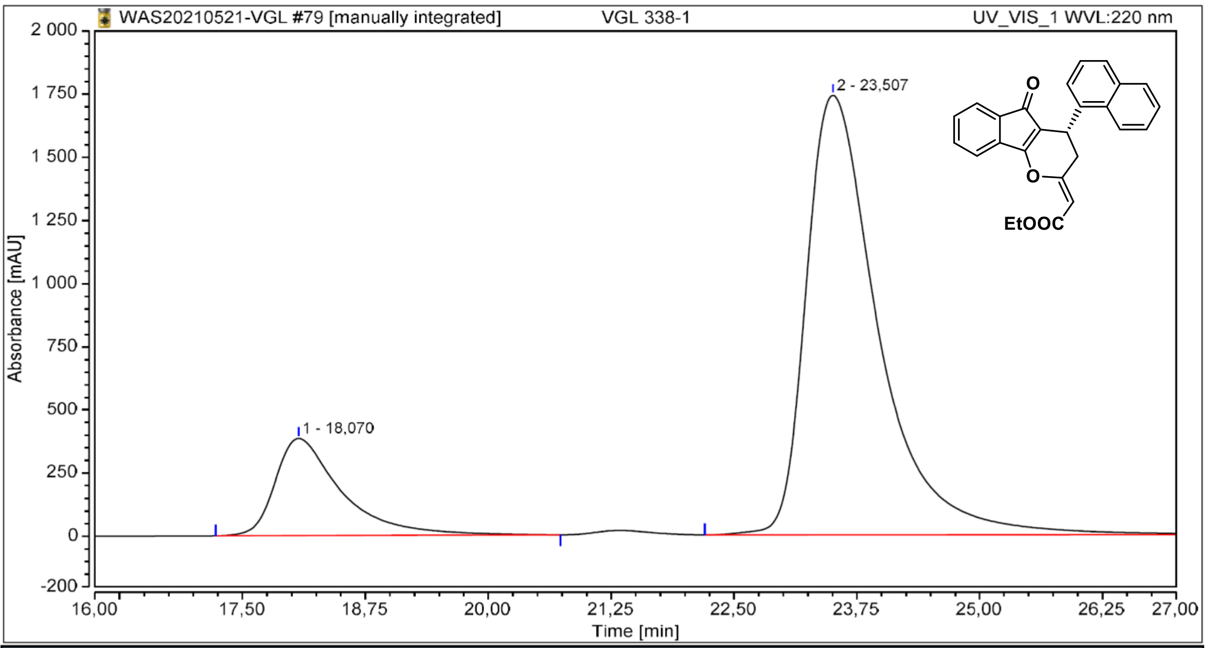

Integration Results

No.	Peak Name	Retention Time min	Area mAU*min	Height mAU	Relative Area %	Relative Height %	Amount n.a.
1		18,070	300,600	384,348	16,80	18,09	n.a.
2		23,507	1488,642	1739,976	83,20	81,91	n.a.
**Total:**			**1789,242**	**2124,324**	**100,00**	**100,00**	

Chromatograms of **3q**

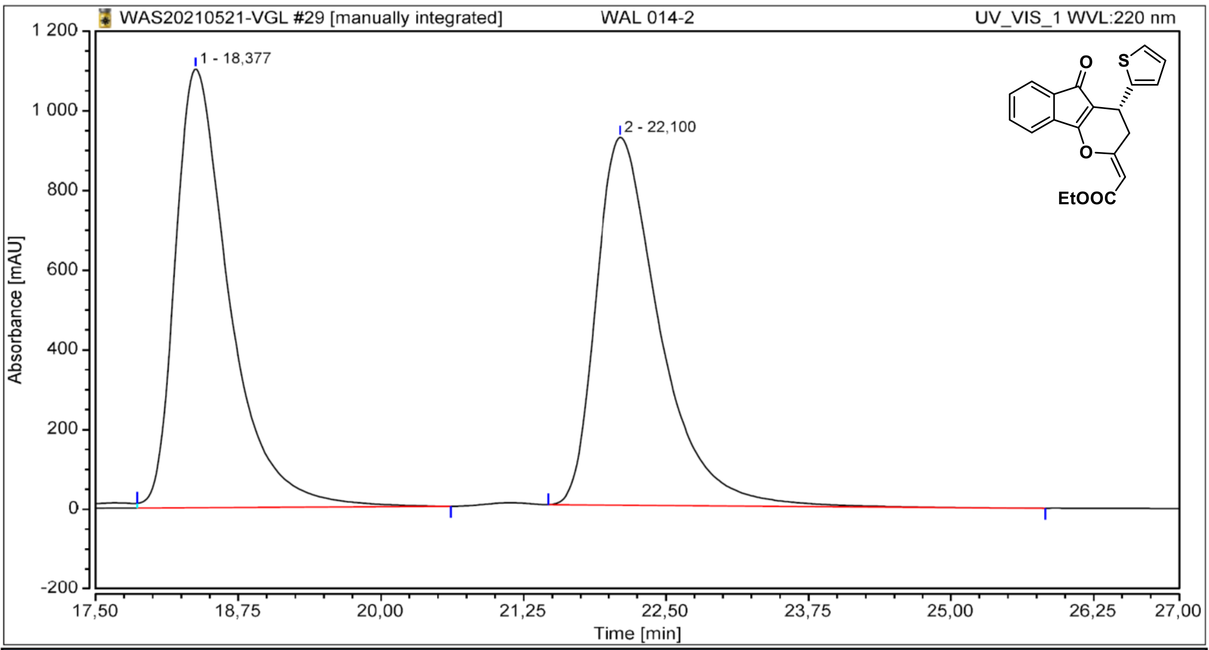

Integration Results							
No.	Peak Name	Retention Time min	Area mAU*min	Height mAU	Relative Area %	Relative Height %	Amount n.a.
1		18,377	605,257	1100,949	50,31	54,38	n.a.
2		22,100	597,697	923,462	49,69	45,62	n.a.
**Total:**			**1202,953**	**2024,411**	**100,00**	**100,00**	

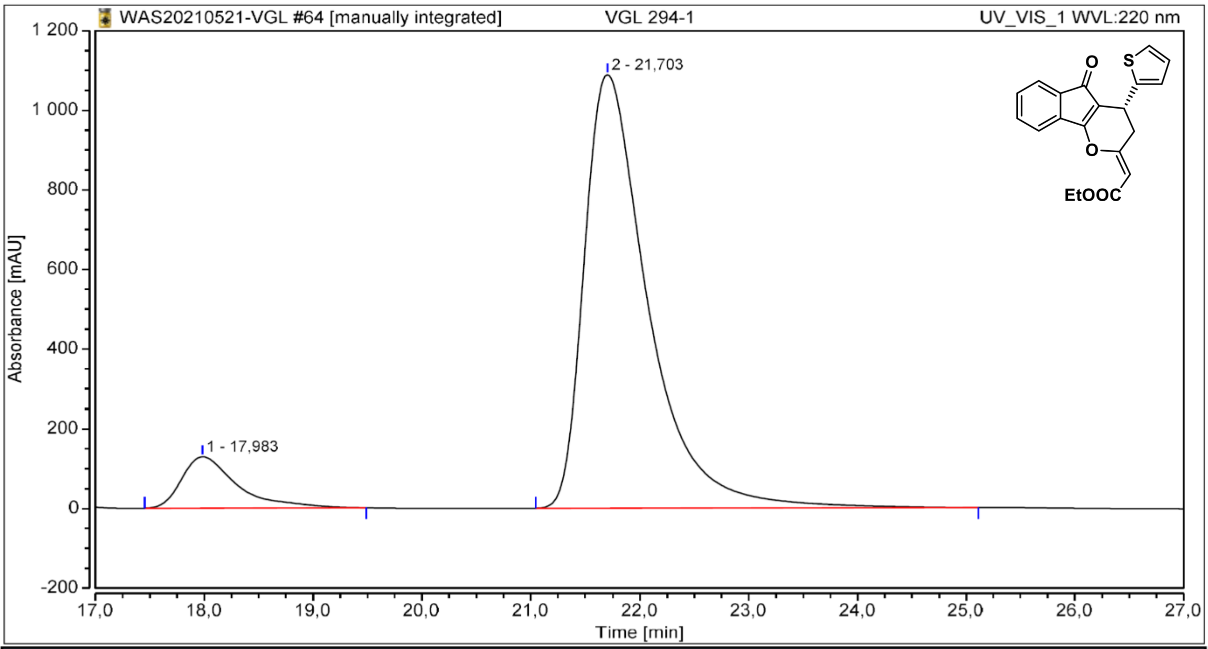

Integration Results							
No.	Peak Name	Retention Time min	Area mAU*min	Height mAU	Relative Area %	Relative Height %	Amount n.a.
1		17,983	76,324	129,324	9,42	10,62	n.a.
2		21,703	734,037	1088,560	90,58	89,38	n.a.
**Total:**			**810,362**	**1217,884**	**100,00**	**100,00**	

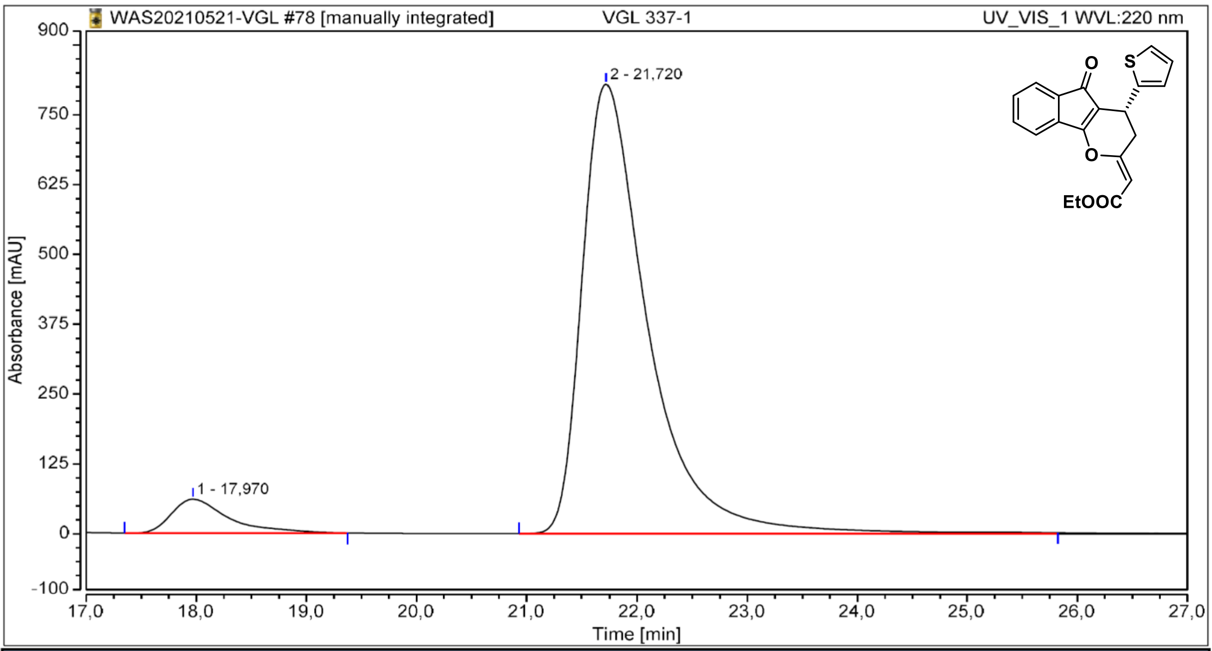

Integration Results

No.	Peak Name	Retention Time min	Area mAU*min	Height mAU	Relative Area %	Relative Height %	Amount n.a.
1		17,970	36,432	60,653	6,16	7,01	n.a.
2		21,720	555,168	804,421	93,84	92,99	n.a.
**Total:**			**591,600**	**865,075**	**100,00**	**100,00**	

Chromatograms of **3r**

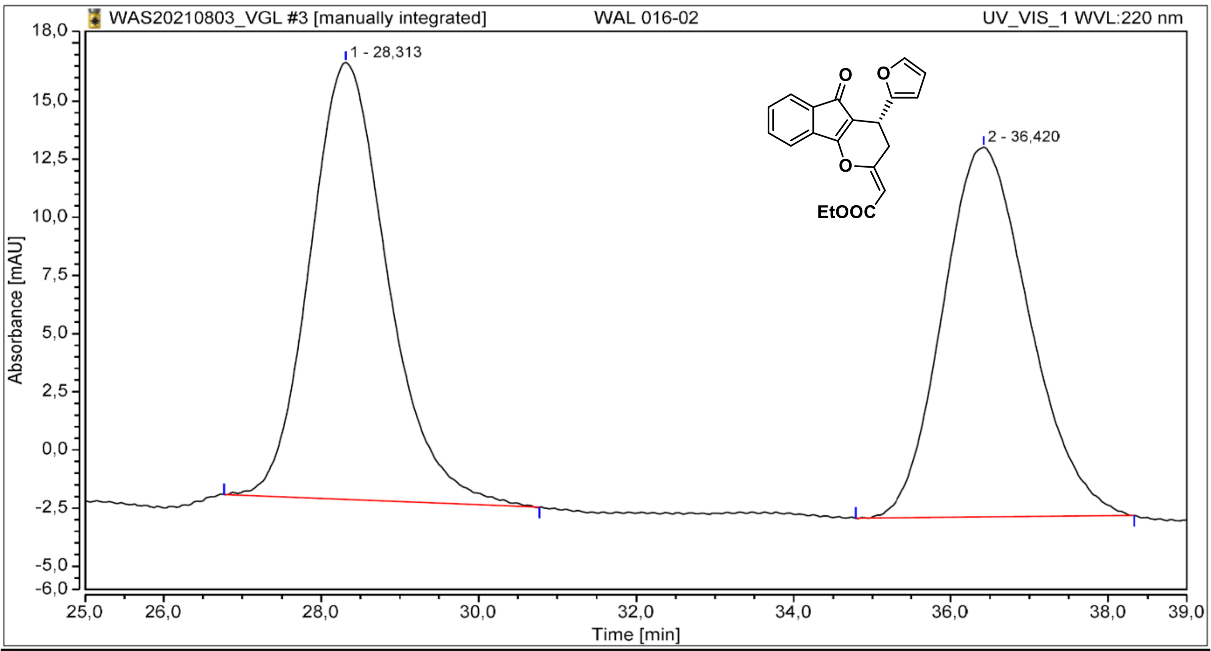

Integration Results

No.	Peak Name	Retention Time min	Area mAU*min	Height mAU	Relative Area %	Relative Height %	Amount n.a.
1		28,313	21,523	18,788	51,47	54,17	n.a.
2		36,420	20,292	15,898	48,53	45,83	n.a.
**Total:**			**41,816**	**34,687**	**100,00**	**100,00**	

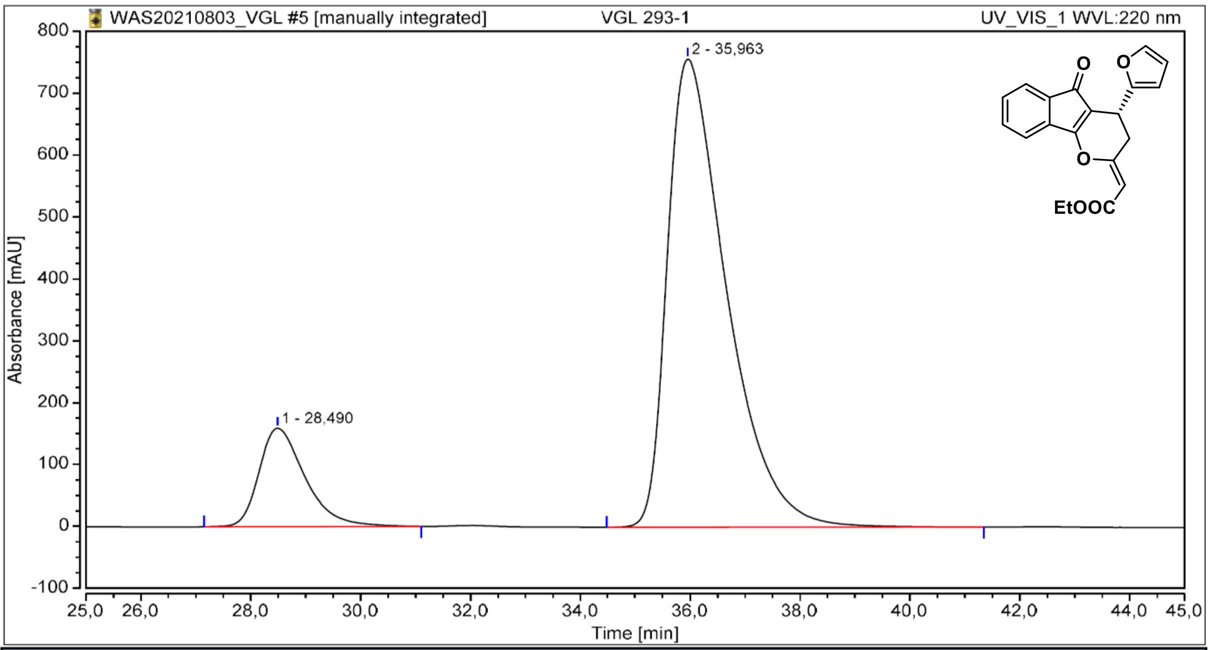

Integration Results

No.	Peak Name	Retention Time min	Area mAU*min	Height mAU	Relative Area %	Relative Height %	Amount n.a.
1		28,490	153,991	158,745	14,02	17,34	n.a.
2		35,963	944,470	756,540	85,98	82,66	n.a.
**Total:**			**1098,461**	**915,285**	**100,00**	**100,00**	

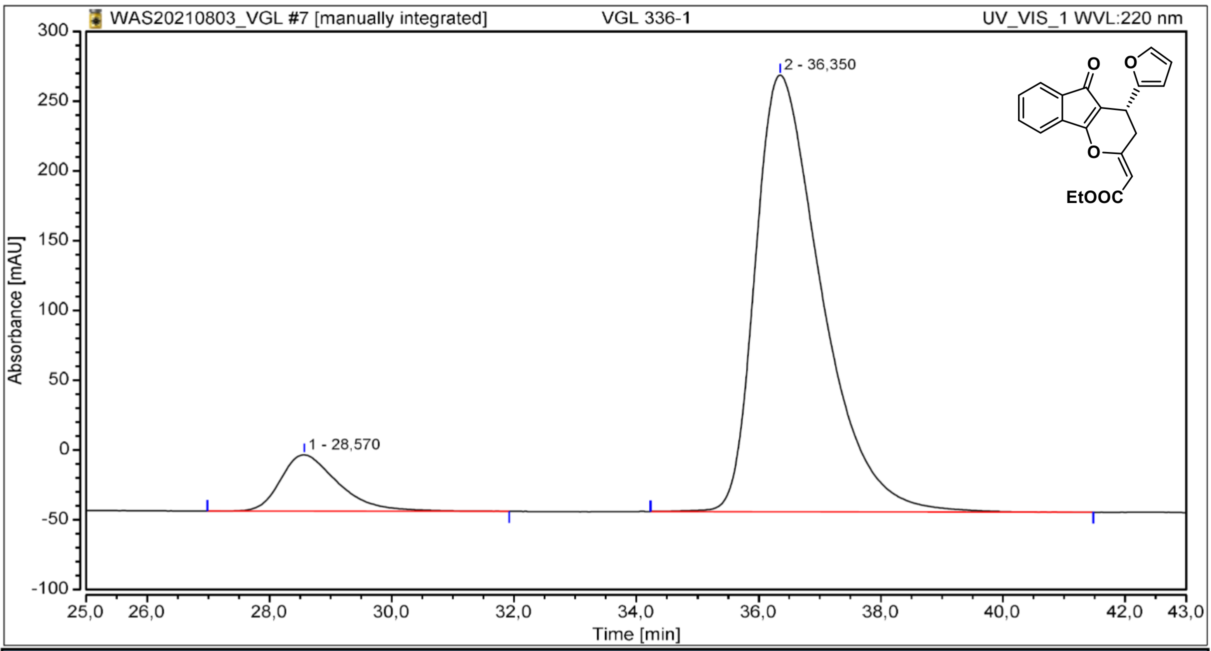

Integration Results							
No.	Peak Name	Retention Time min	Area mAU*min	Height mAU	Relative Area %	Relative Height %	Amount n.a.
1		28,570	44,031	40,383	10,11	11,43	n.a.
2		36,350	391,658	312,961	89,89	88,57	n.a.
**Total:**			**435,690**	**353,344**	**100,00**	**100,00**	

Chromatograms of **3s**$_{major}$

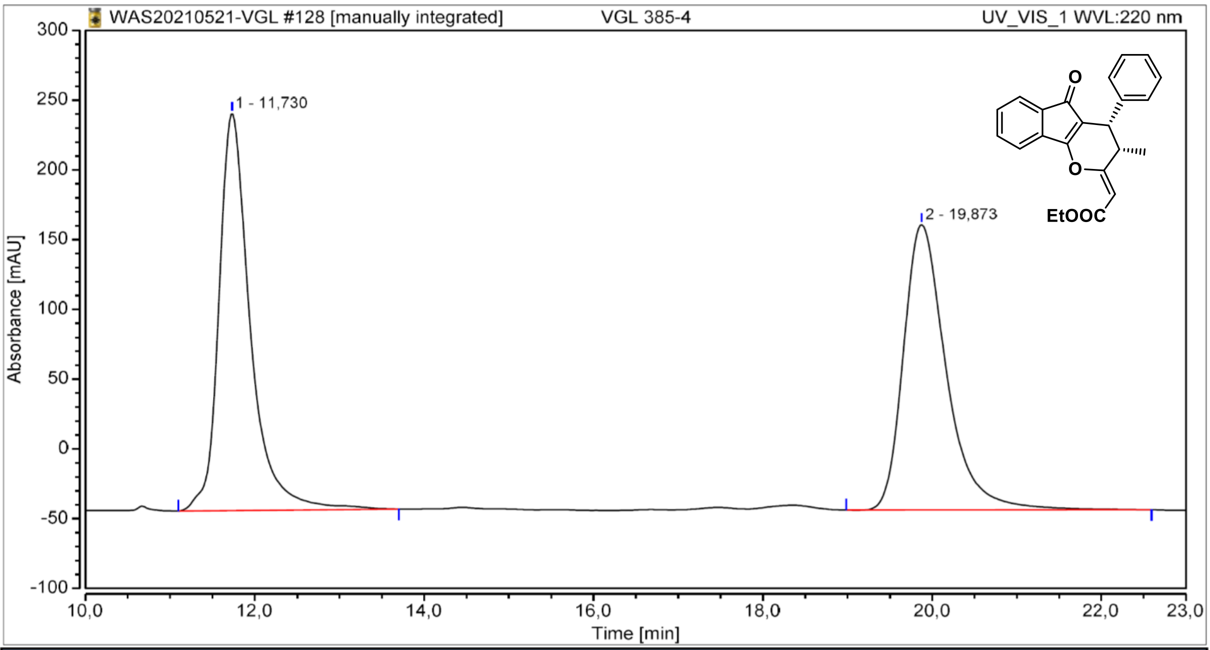

Integration Results							
No.	Peak Name	Retention Time min	Area mAU*min	Height mAU	Relative Area %	Relative Height %	Amount n.a.
1		11,730	122,151	284,794	50,39	58,20	n.a.
2		19,873	120,276	204,522	49,61	41,80	n.a.
**Total:**			**242,428**	**489,316**	**100,00**	**100,00**	

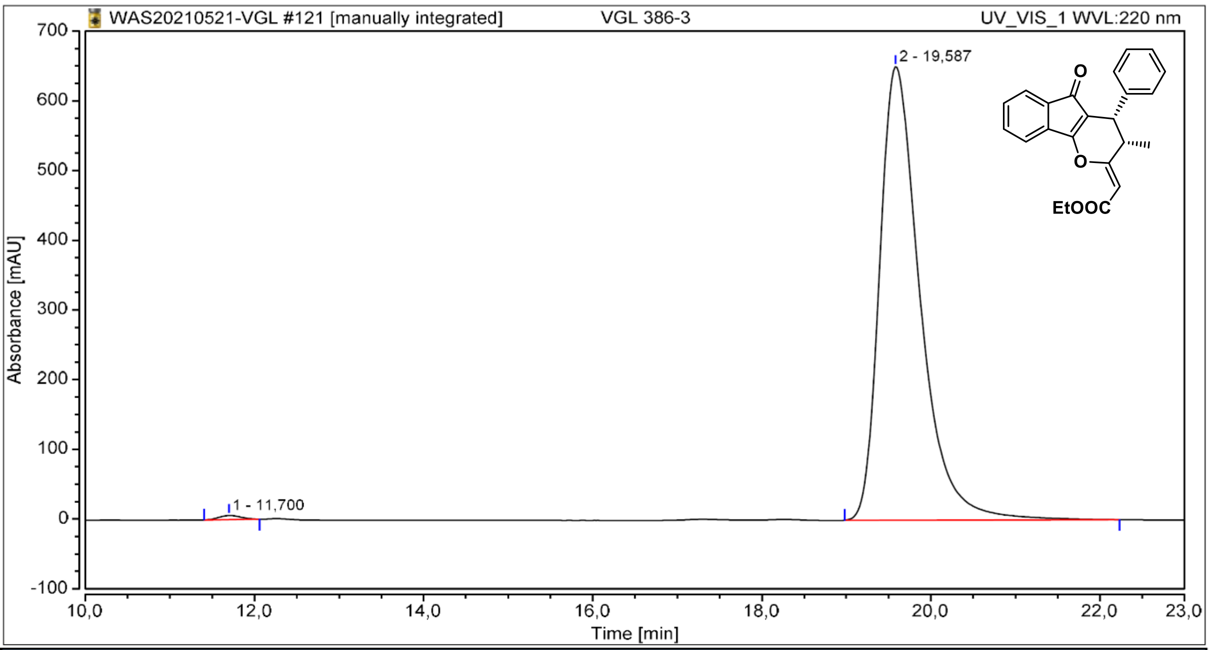

Integration Results							
No.	Peak Name	Retention Time min	Area mAU*min	Height mAU	Relative Area %	Relative Height %	Amount n.a.
1		11,700	1,811	5,975	0,50	0,91	n.a.
2		19,587	360,498	651,006	99,50	99,09	n.a.
**Total:**			**362,309**	**656,981**	**100,00**	**100,00**	

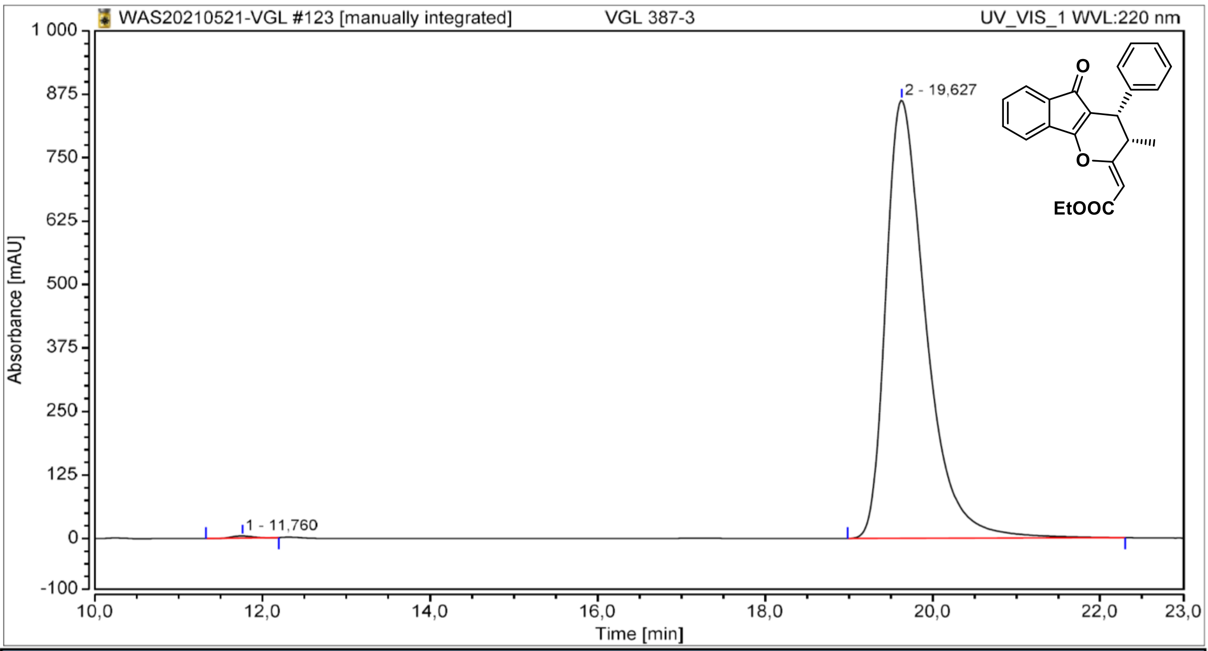

Integration Results

No.	Peak Name	Retention Time min	Area mAU*min	Height mAU	Relative Area %	Relative Height %	Amount n.a.
1		11,760	1,121	4,194	0,23	0,48	n.a.
2		19,627	478,456	863,249	99,77	99,52	n.a.
**Total:**			**479,577**	**867,443**	**100,00**	**100,00**	

Chromatograms of **3s**$_{minor}$

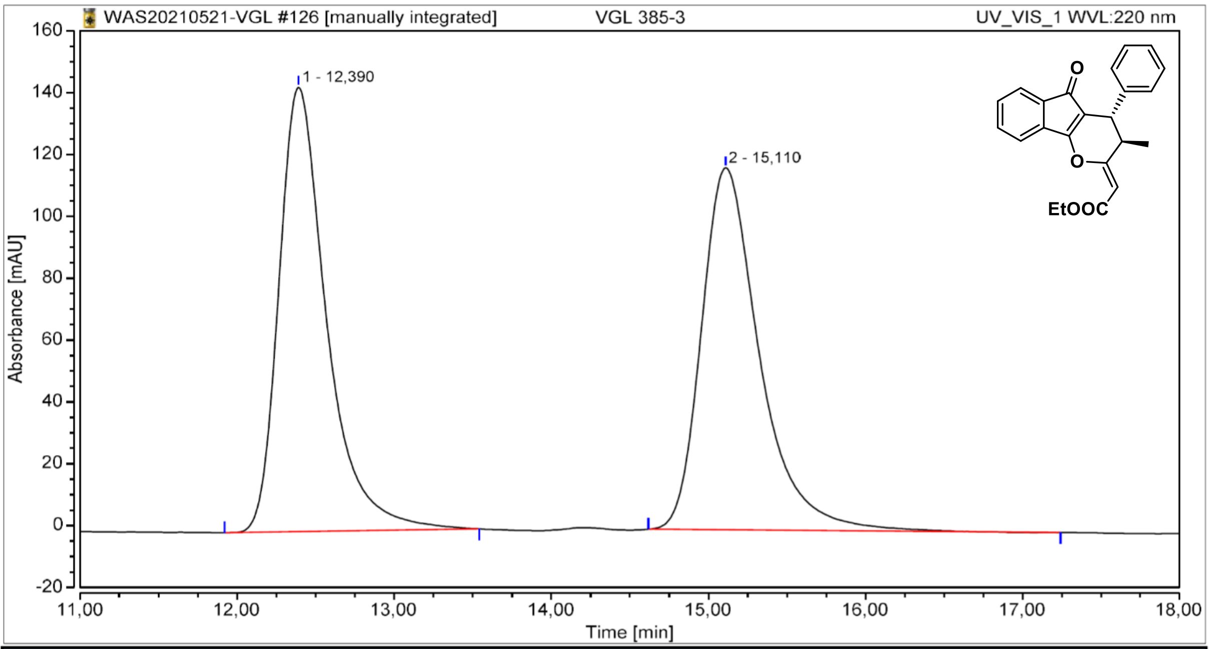

Integration Results							
No.	Peak Name	Retention Time min	Area mAU*min	Height mAU	Relative Area %	Relative Height %	Amount n.a.
1		12,390	50,513	143,678	50,18	55,11	n.a.
2		15,110	50,159	117,056	49,82	44,89	n.a.
**Total:**			**100,672**	**260,735**	**100,00**	**100,00**	

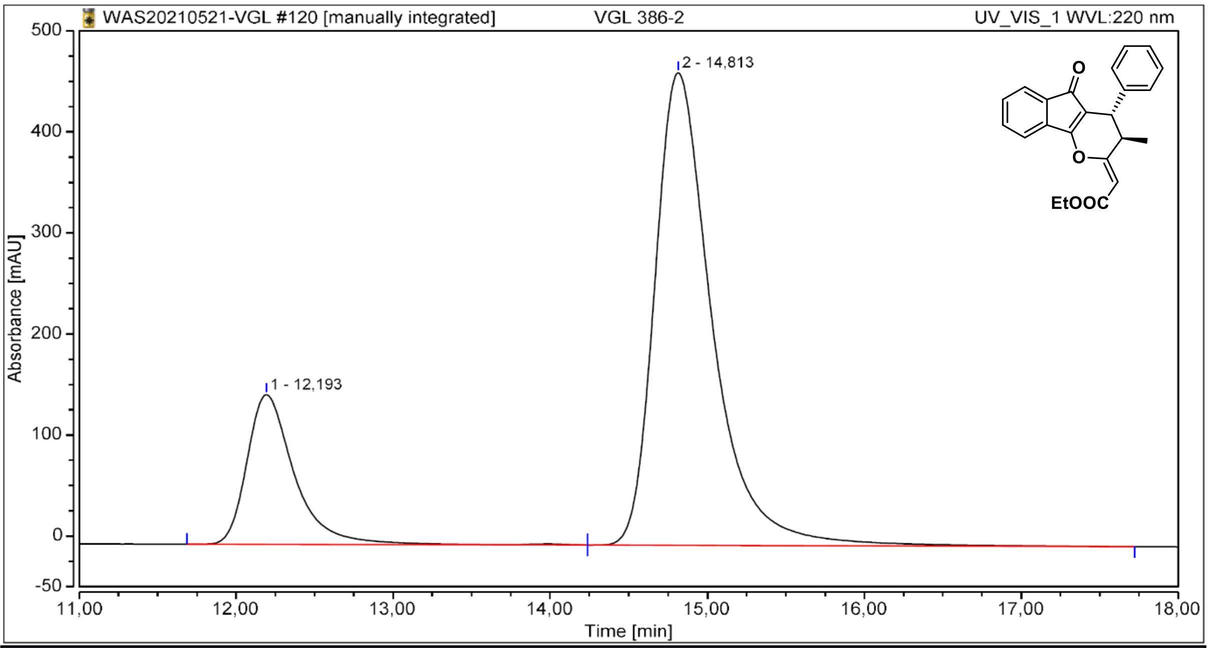

Integration Results							
No.	Peak Name	Retention Time min	Area mAU*min	Height mAU	Relative Area %	Relative Height %	Amount n.a.
1		12,193	52,271	148,185	20,85	24,06	n.a.
2		14,813	198,379	467,703	79,15	75,94	n.a.
**Total:**			**250,650**	**615,888**	**100,00**	**100,00**	

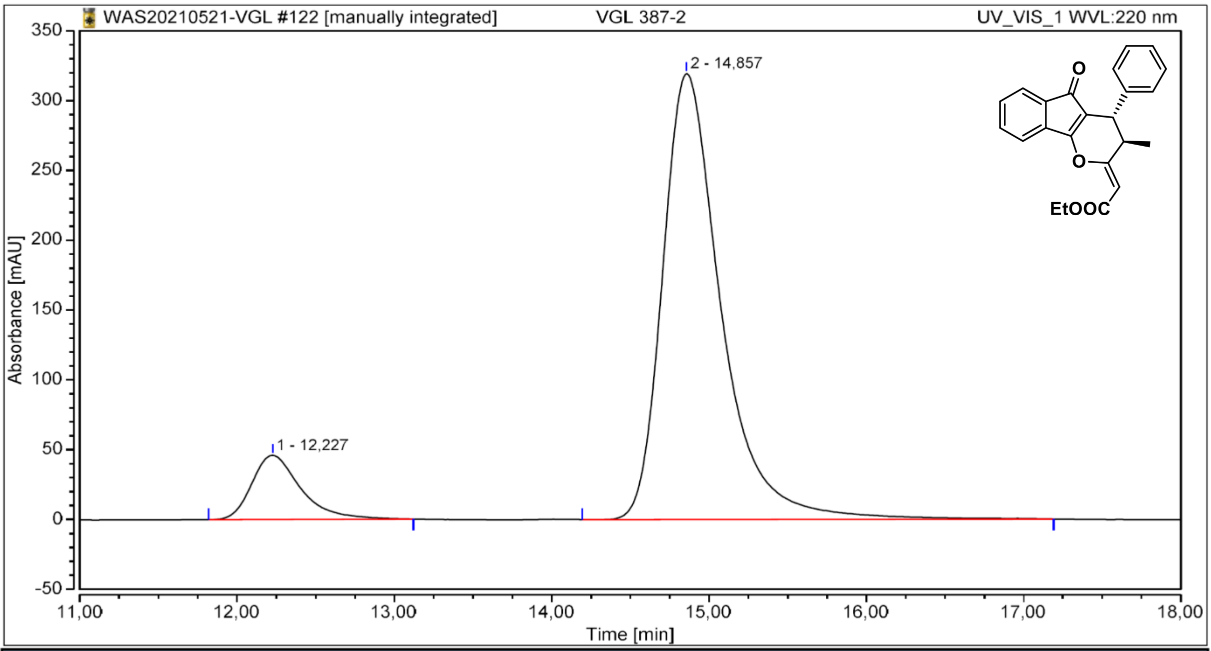

Integration Results							
No.	Peak Name	Retention Time min	Area mAU*min	Height mAU	Relative Area %	Relative Height %	Amount n.a.
1		12,227	16,541	45,962	10,73	12,58	n.a.
2		14,857	137,545	319,322	89,27	87,42	n.a.
**Total:**			**154,086**	**365,284**	**100,00**	**100,00**	

Chromatograms of **3t**$_{\mathbf{major}}$

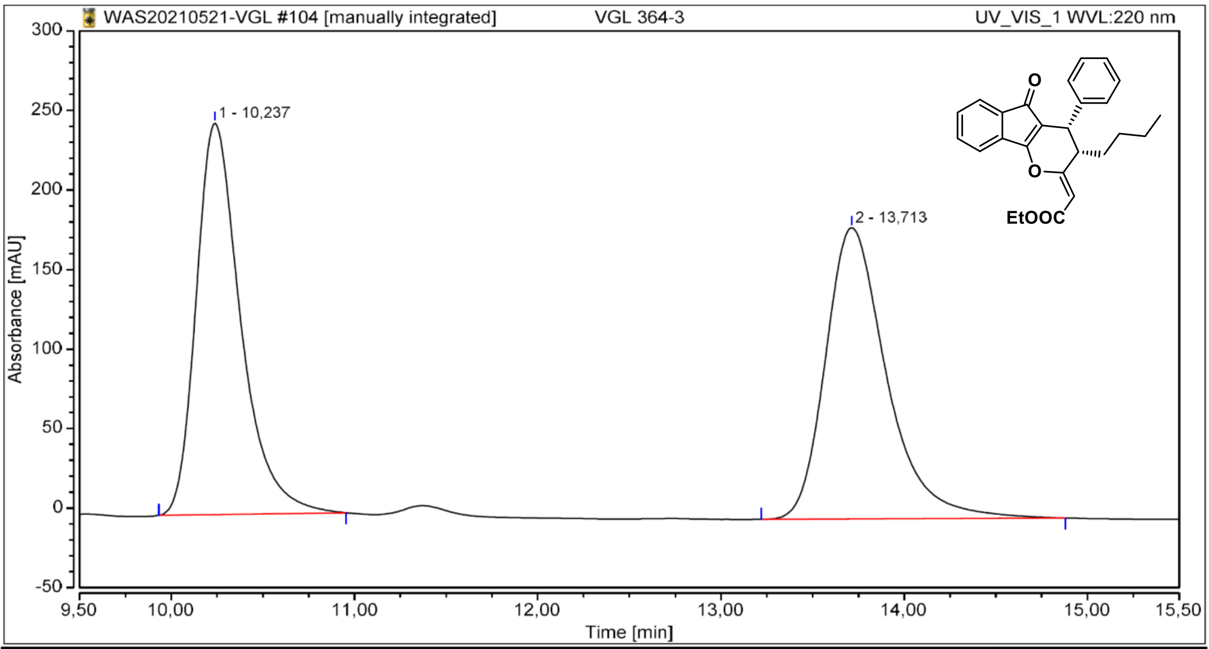

Integration Results							
No.	Peak Name	Retention Time min	Area mAU*min	Height mAU	Relative Area %	Relative Height %	Amount n.a.
1		10,237	69,198	246,073	49,40	57,32	n.a.
2		13,713	70,881	183,205	50,60	42,68	n.a.
**Total:**			**140,080**	**429,278**	**100,00**	**100,00**	

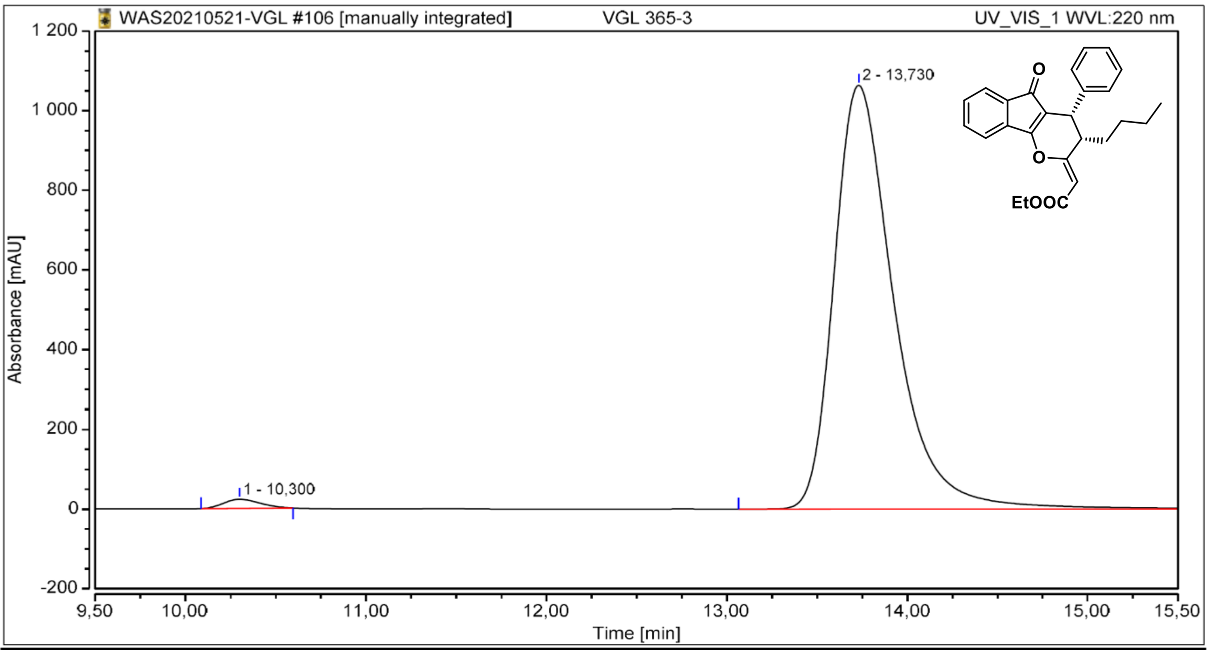

Integration Results							
No.	Peak Name	Retention Time min	Area mAU*min	Height mAU	Relative Area %	Relative Height %	Amount n.a.
1		10,300	5,308	22,788	1,27	2,10	n.a.
2		13,730	411,778	1064,271	98,73	97,90	n.a.
**Total:**			**417,086**	**1087,058**	**100,00**	**100,00**	

Chromatograms of **3t**$_{minor}$

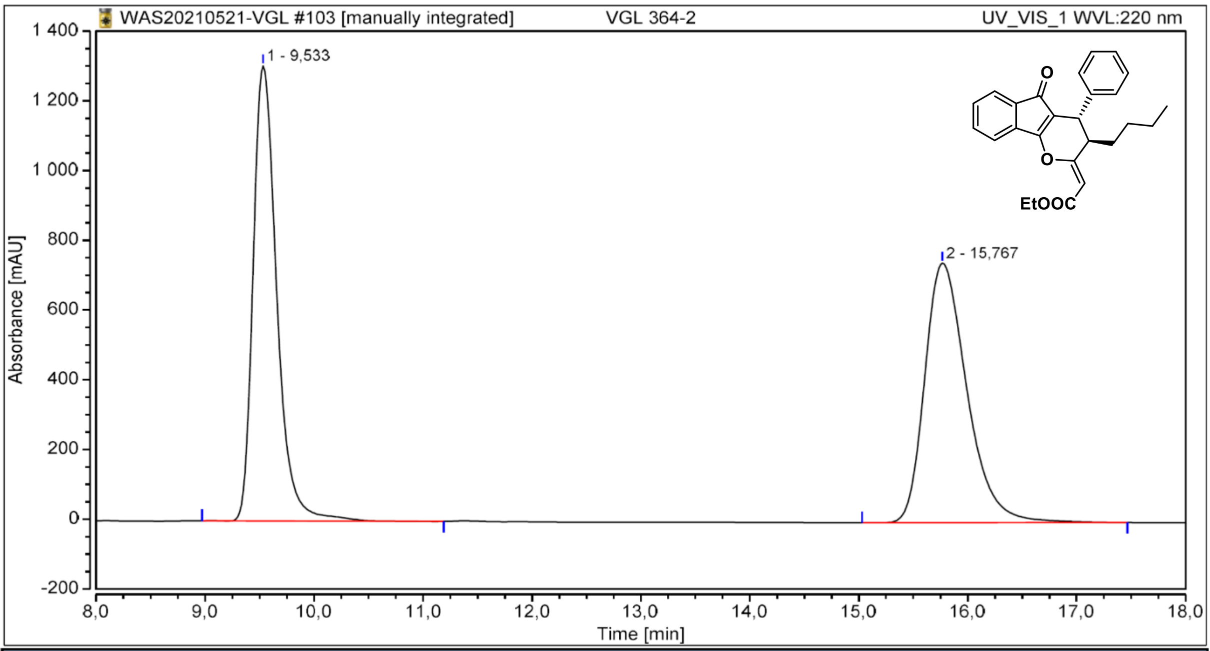

Integration Results							
No.	Peak Name	Retention Time min	Area mAU*min	Height mAU	Relative Area %	Relative Height %	Amount n.a.
1		9,533	327,443	1305,400	49,53	63,68	n.a.
2		15,767	333,645	744,518	50,47	36,32	n.a.
**Total:**			**661,088**	**2049,918**	**100,00**	**100,00**	

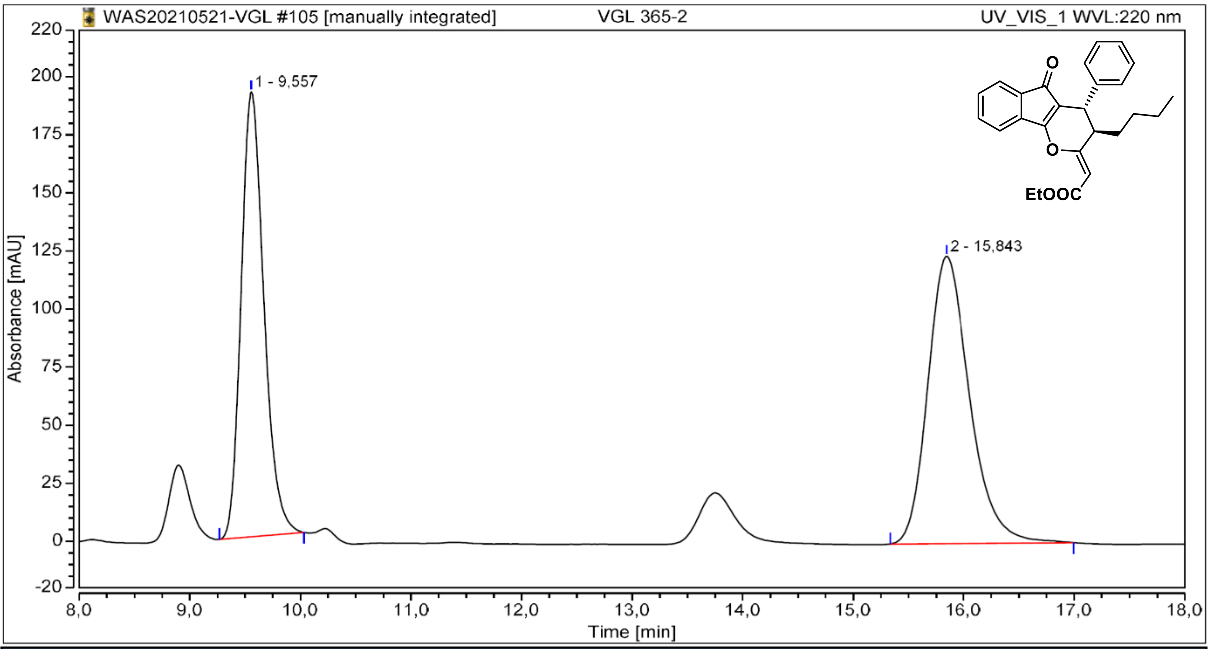

Integration Results							
No.	Peak Name	Retention Time min	Area mAU*min	Height mAU	Relative Area %	Relative Height %	Amount n.a.
1		9,557	45,643	191,469	45,61	60,76	n.a.
2		15,843	54,420	123,645	54,39	39,24	n.a.
**Total:**			**100,063**	**315,113**	**100,00**	**100,00**	

Chromatorgams of **3u**$_{\mathbf{major}}$

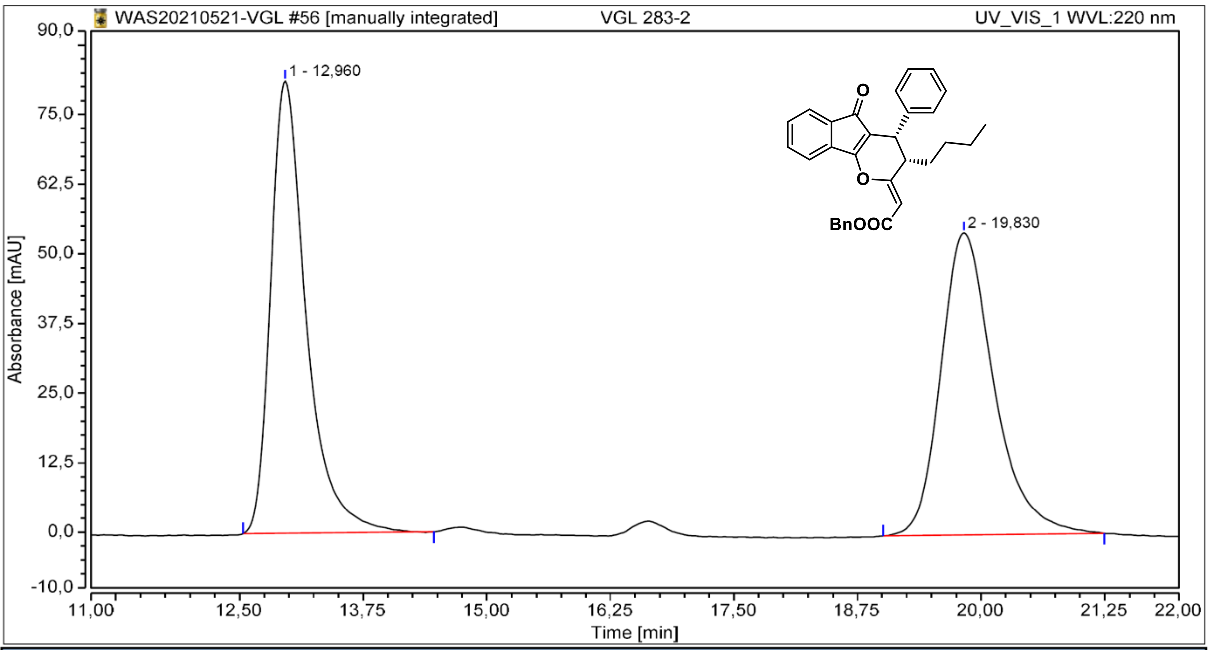

Integration Results

No.	Peak Name	Retention Time min	Area mAU*min	Height mAU	Relative Area %	Relative Height %	Amount n.a.
1		12,960	33,800	81,218	50,07	59,93	n.a.
2		19,830	33,711	54,297	49,93	40,07	n.a.
**Total:**			**67,510**	**135,515**	**100,00**	**100,00**	

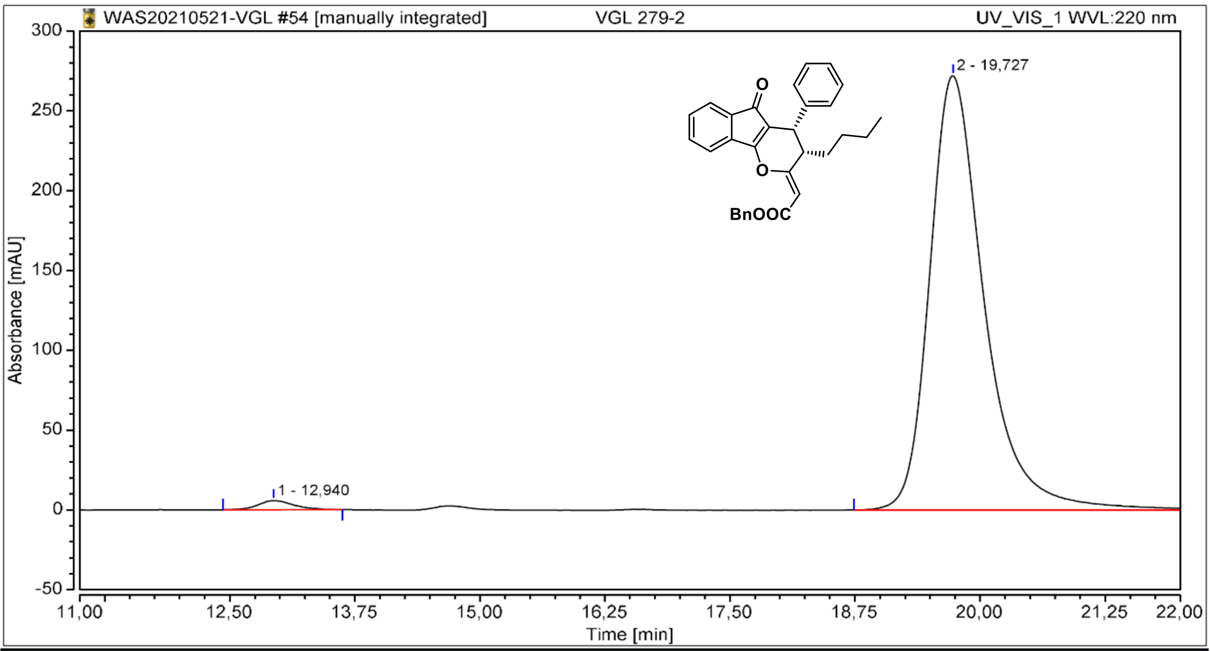

Integration Results

No.	Peak Name	Retention Time min	Area mAU*min	Height mAU	Relative Area %	Relative Height %	Amount n.a.
1		12,940	2,386	5,707	1,36	2,05	n.a.
2		19,727	172,864	272,232	98,64	97,95	n.a.
**Total:**			**175,250**	**277,939**	**100,00**	**100,00**	

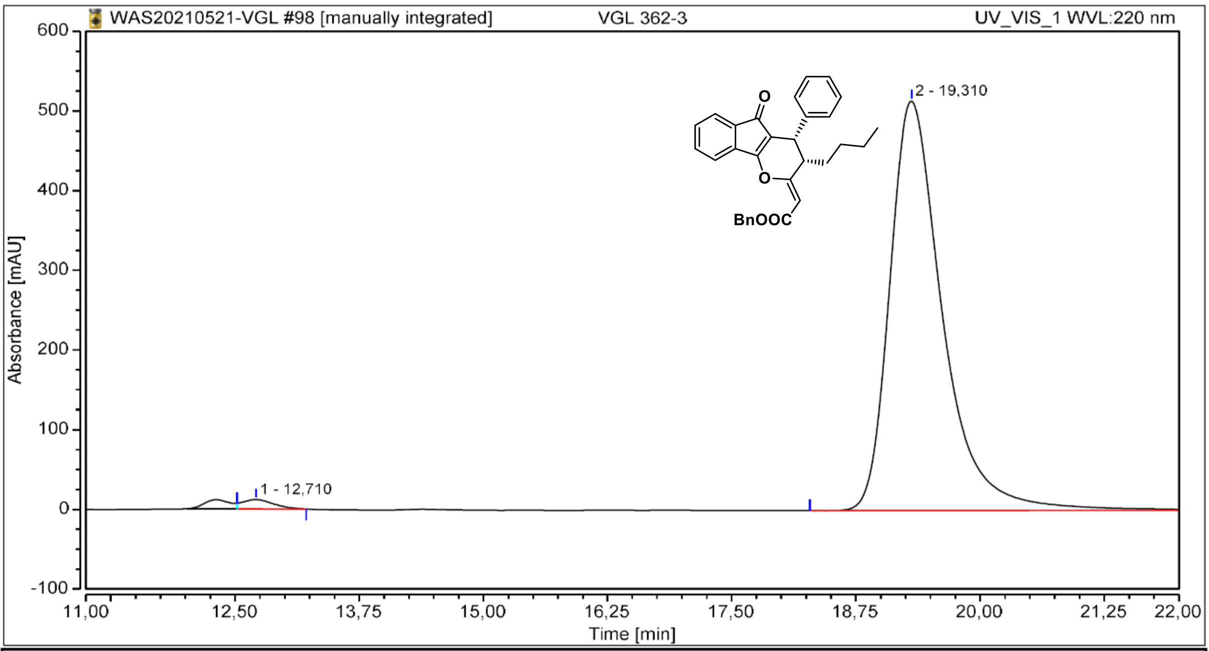

Integration Results							
No.	Peak Name	Retention Time min	Area mAU*min	Height mAU	Relative Area %	Relative Height %	Amount n.a.
1		12,710	4,280	11,774	1,33	2,24	n.a.
2		19,310	317,115	513,762	98,67	97,76	n.a.
**Total:**			**321,395**	**525,535**	**100,00**	**100,00**	

Chromatograms of **3u**$_{minor}$

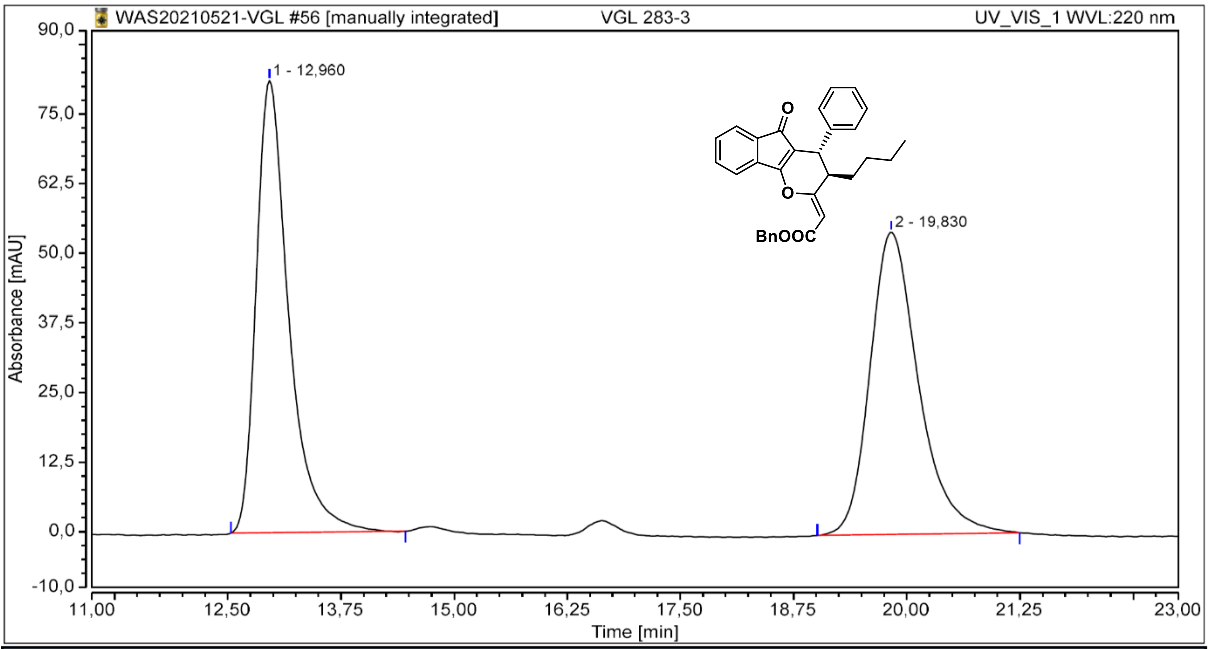

Integration Results							
No.	Peak Name	Retention Time<br>min	Area<br>mAU*min	Height<br>mAU	Relative Area<br>%	Relative Height<br>%	Amount<br>n.a.
1		12,960	33,800	81,218	50,07	59,93	n.a.
2		19,830	33,711	54,297	49,93	40,07	n.a.
**Total:**			**67,510**	**135,515**	**100,00**	**100,00**	

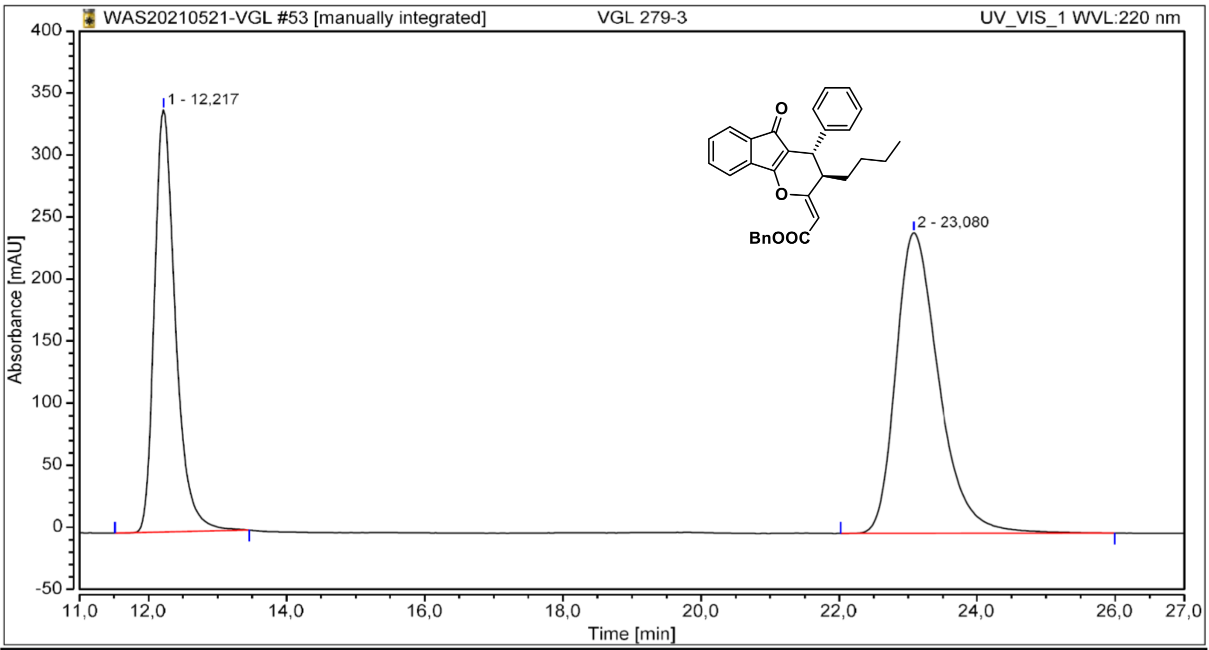

Integration Results							
No.	Peak Name	Retention Time<br>min	Area<br>mAU*min	Height<br>mAU	Relative Area<br>%	Relative Height<br>%	Amount<br>n.a.
1		12,217	120,150	340,394	40,66	58,43	n.a.
2		23,080	175,339	242,215	59,34	41,57	n.a.
**Total:**			**295,489**	**582,609**	**100,00**	**100,00**	

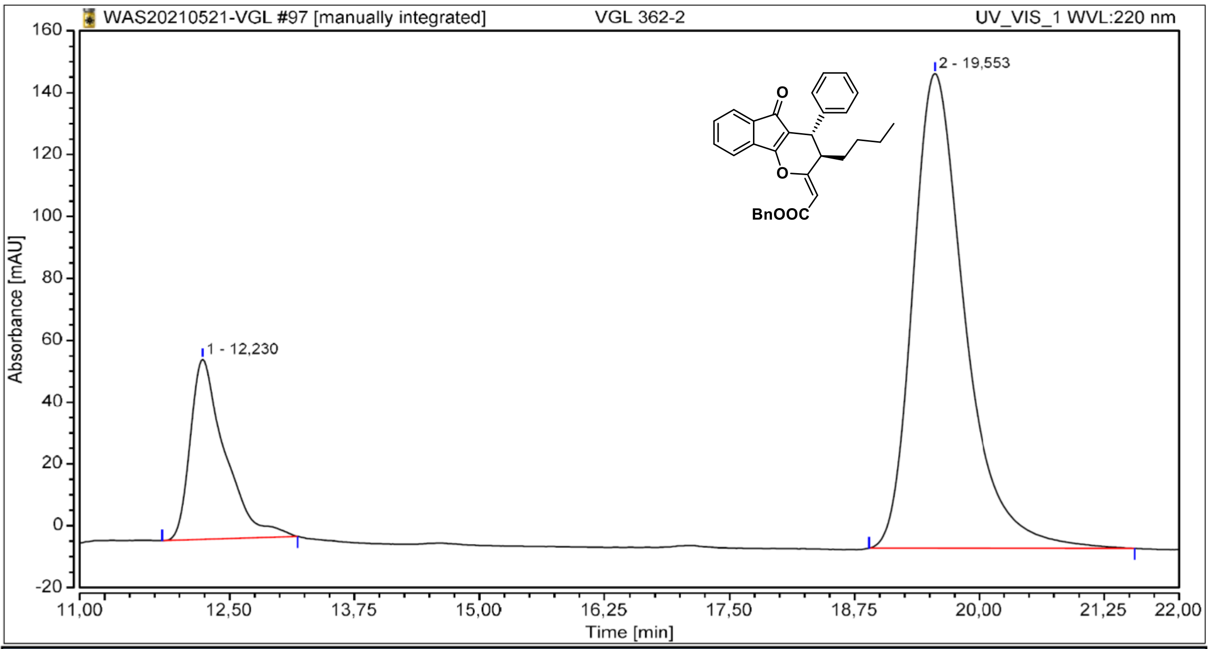

Integration Results							
No.	Peak Name	Retention Time min	Area mAU*min	Height mAU	Relative Area %	Relative Height %	Amount n.a.
1		12,230	23,327	58,264	20,03	27,52	n.a.
2		19,553	93,137	153,482	79,97	72,48	n.a.
**Total:**			**116,464**	**211,747**	**100,00**	**100,00**	

Chromatogram of **3v**

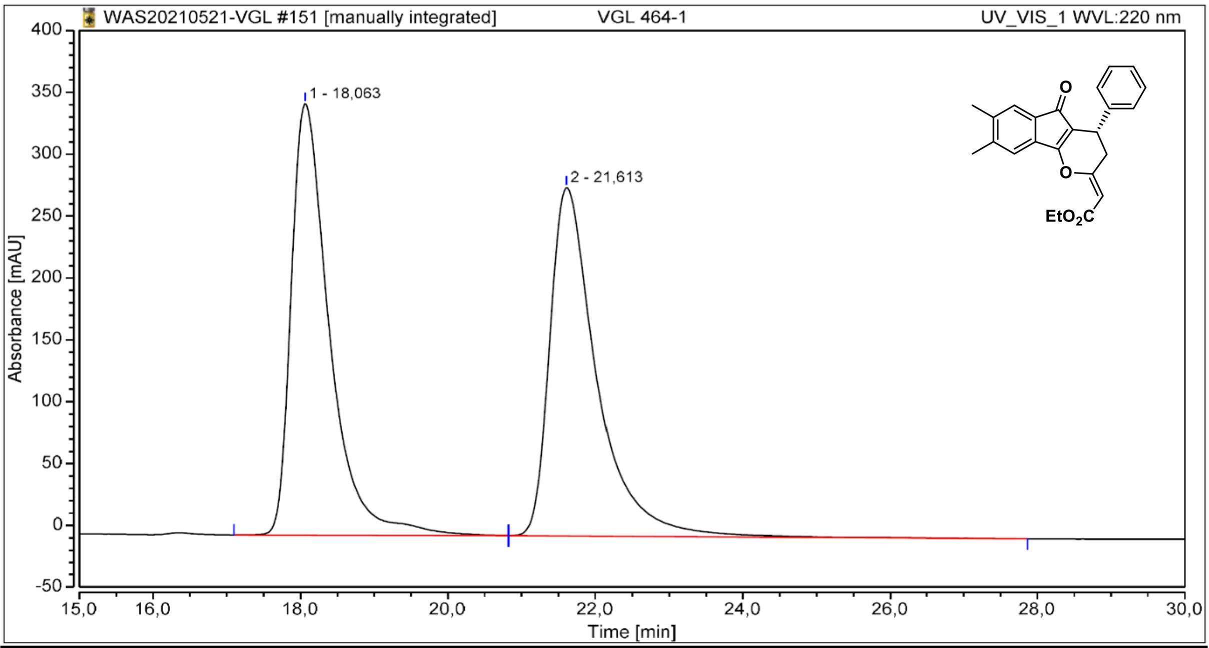

Integration Results							
No.	Peak Name	Retention Time<br>min	Area<br>mAU*min	Height<br>mAU	Relative Area<br>%	Relative Height<br>%	Amount<br>n.a.
1		18,063	209,364	348,753	50,17	55,33	n.a.
2		21,613	207,915	281,573	49,83	44,67	n.a.
**Total:**			**417,279**	**630,327**	**100,00**	**100,00**	

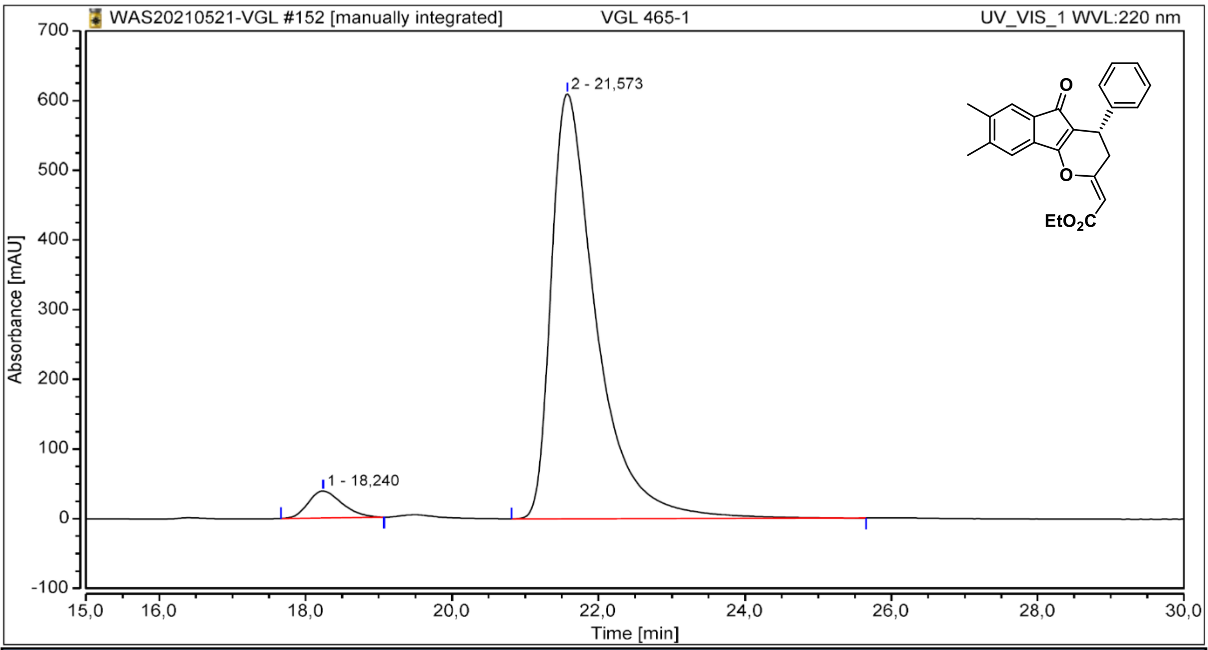

Integration Results							
No.	Peak Name	Retention Time<br>min	Area<br>mAU*min	Height<br>mAU	Relative Area<br>%	Relative Height<br>%	Amount<br>n.a.
1		18,240	20,727	38,707	4,55	5,97	n.a.
2		21,573	434,754	609,928	95,45	94,03	n.a.
**Total:**			**455,481**	**648,635**	**100,00**	**100,00**	

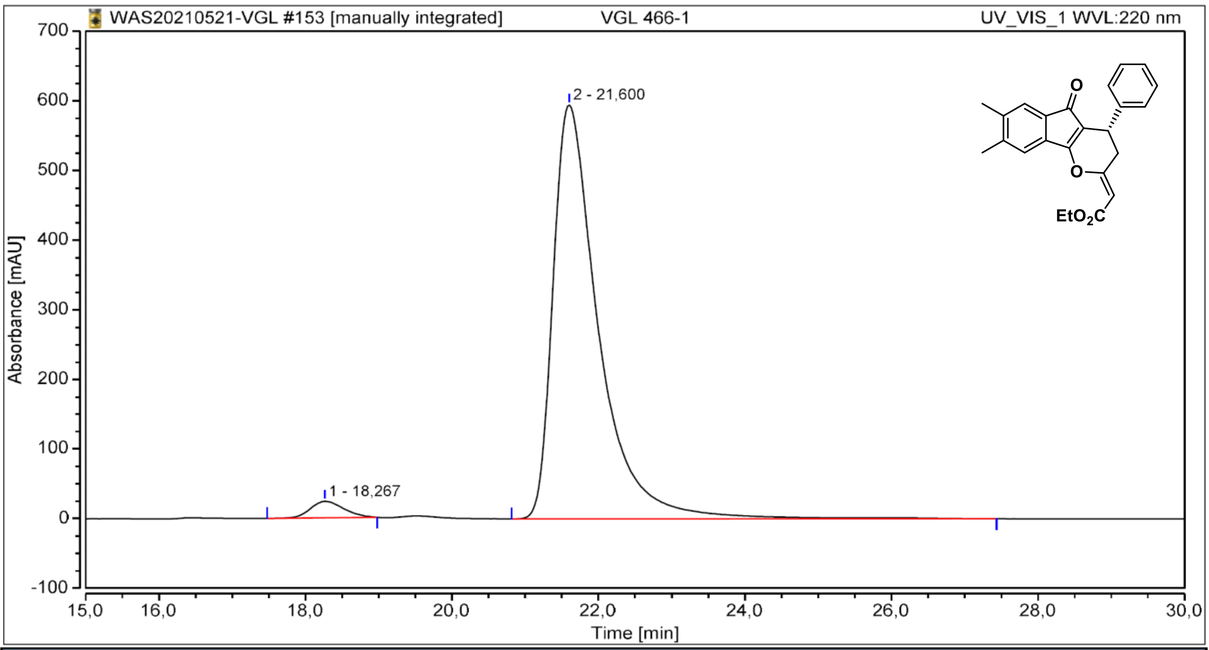

Integration Results

No.	Peak Name	Retention Time min	Area mAU*min	Height mAU	Relative Area %	Relative Height %	Amount n.a.
1		18,267	12,558	23,747	2,85	3,84	n.a.
2		21,600	428,356	594,747	97,15	96,16	n.a.
**Total:**			**440,914**	**618,495**	**100,00**	**100,00**	

Chromatograms of **3w**

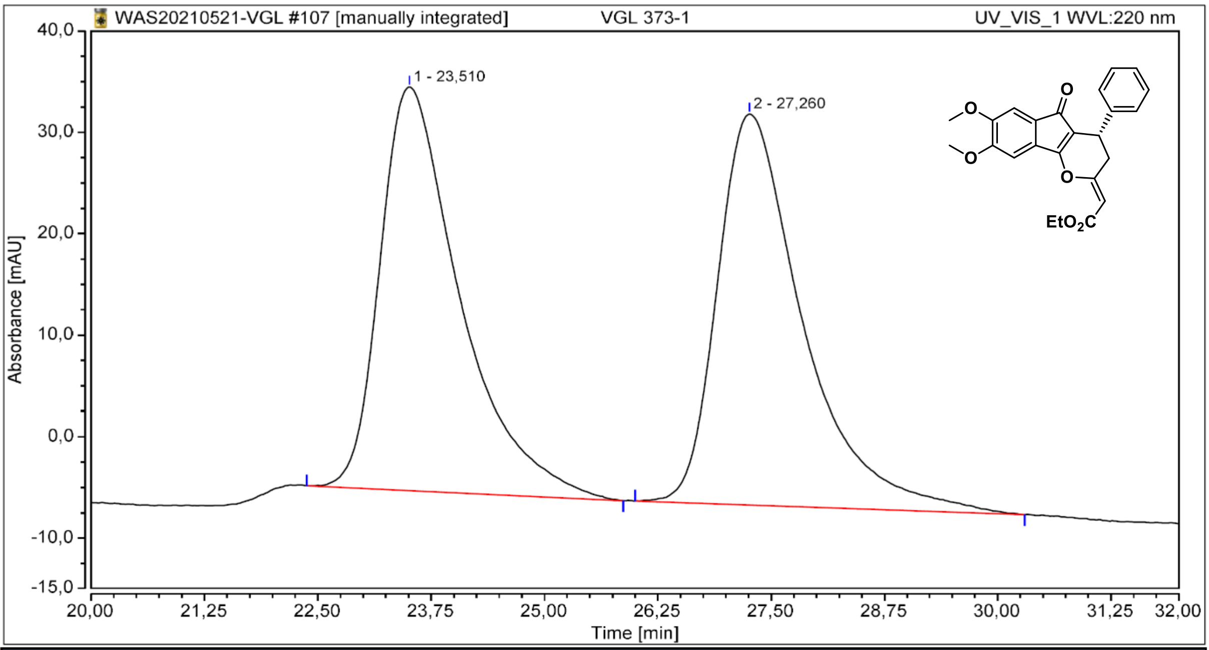

Integration Results							
No.	Peak Name	Retention Time min	Area mAU*min	Height mAU	Relative Area %	Relative Height %	Amount n.a.
1		23,510	40,610	39,777	49,07	50,79	n.a.
2		27,260	42,157	38,536	50,93	49,21	n.a.
**Total:**			**82,767**	**78,313**	**100,00**	**100,00**	

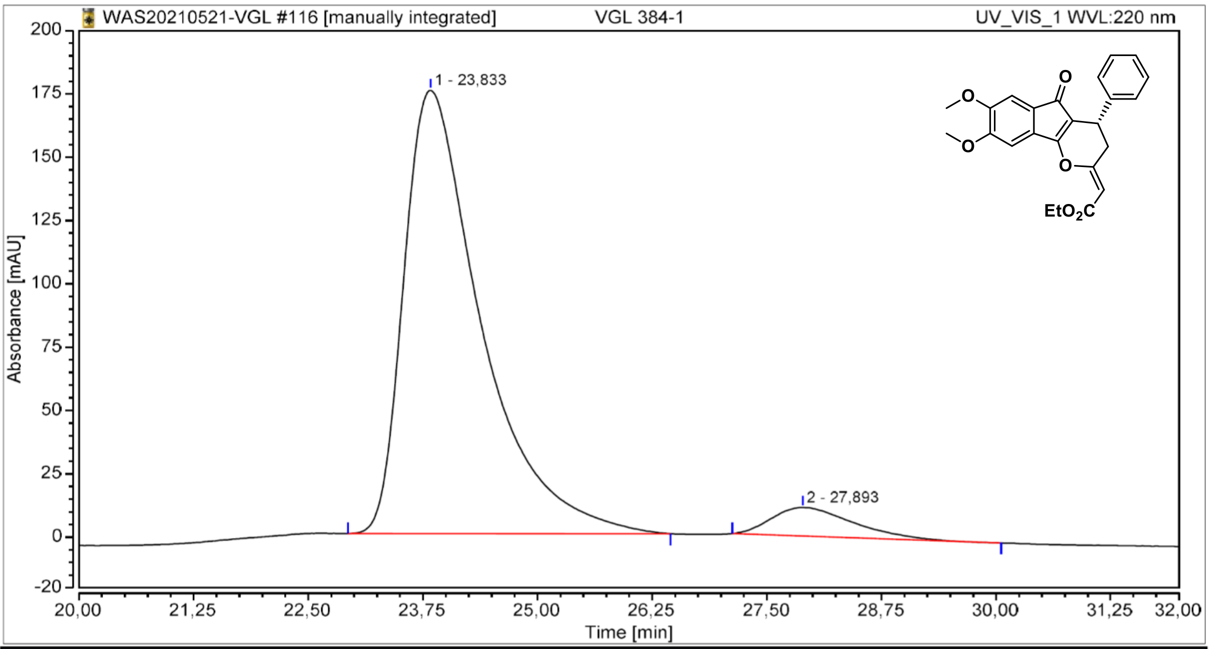

Integration Results							
No.	Peak Name	Retention Time min	Area mAU*min	Height mAU	Relative Area %	Relative Height %	Amount n.a.
1		23,833	170,617	175,026	93,59	93,95	n.a.
2		27,893	11,692	11,280	6,41	6,05	n.a.
**Total:**			**182,309**	**186,306**	**100,00**	**100,00**	

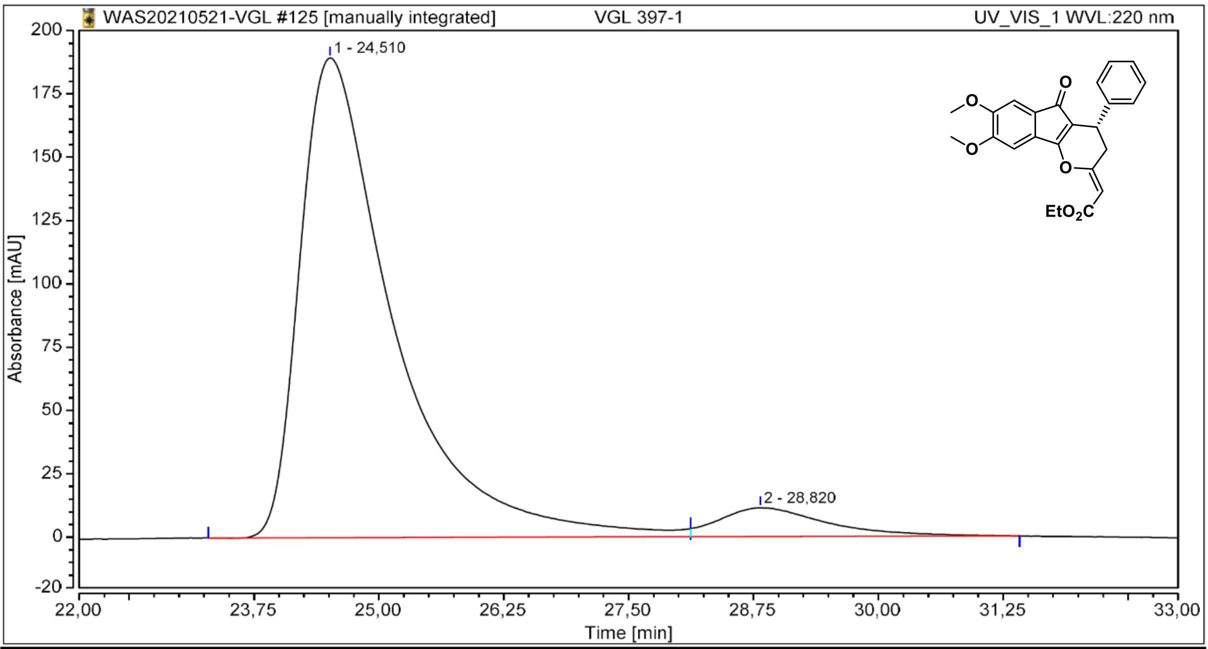

Integration Results							
No.	Peak Name	Retention Time min	Area mAU*min	Height mAU	Relative Area %	Relative Height %	Amount n.a.
1		24,510	206,185	189,379	93,63	94,36	n.a.
2		28,820	14,024	11,326	6,37	5,64	n.a.
**Total:**			**220,209**	**200,705**	**100,00**	**100,00**	

Chromatograms of **3x**

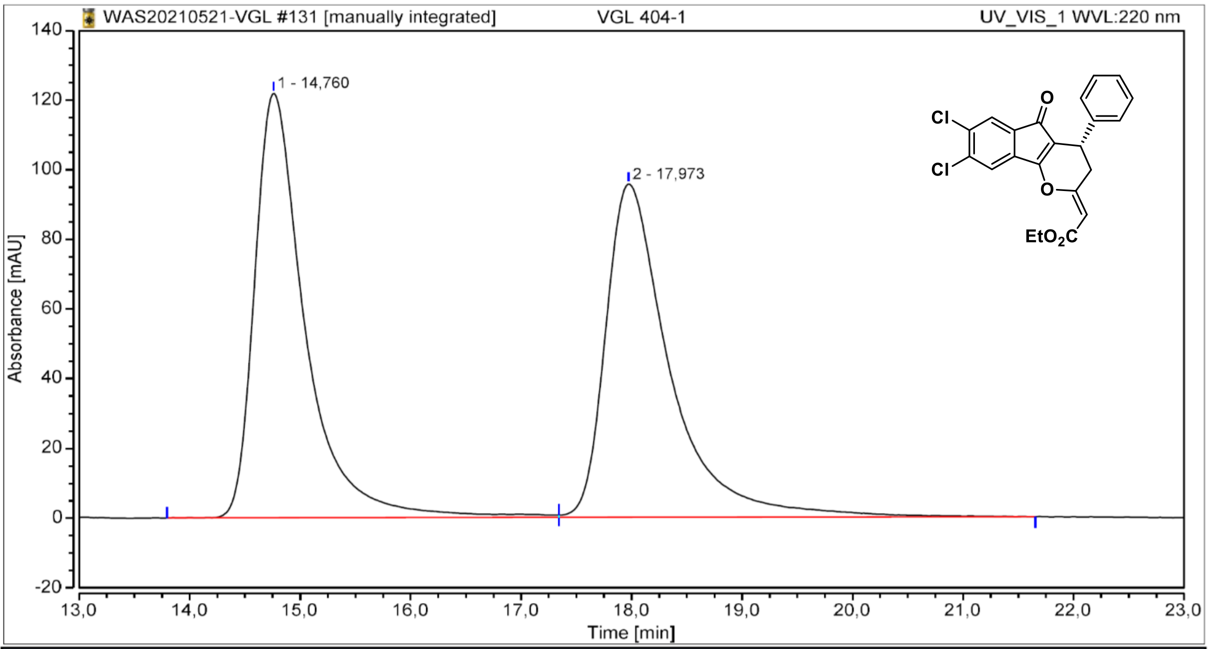

Integration Results							
No.	Peak Name	Retention Time min	Area mAU*min	Height mAU	Relative Area %	Relative Height %	Amount n.a.
1		14,760	64,833	121,896	50,14	56,04	n.a.
2		17,973	64,462	95,637	49,86	43,96	n.a.
**Total:**			**129,295**	**217,533**	**100,00**	**100,00**	

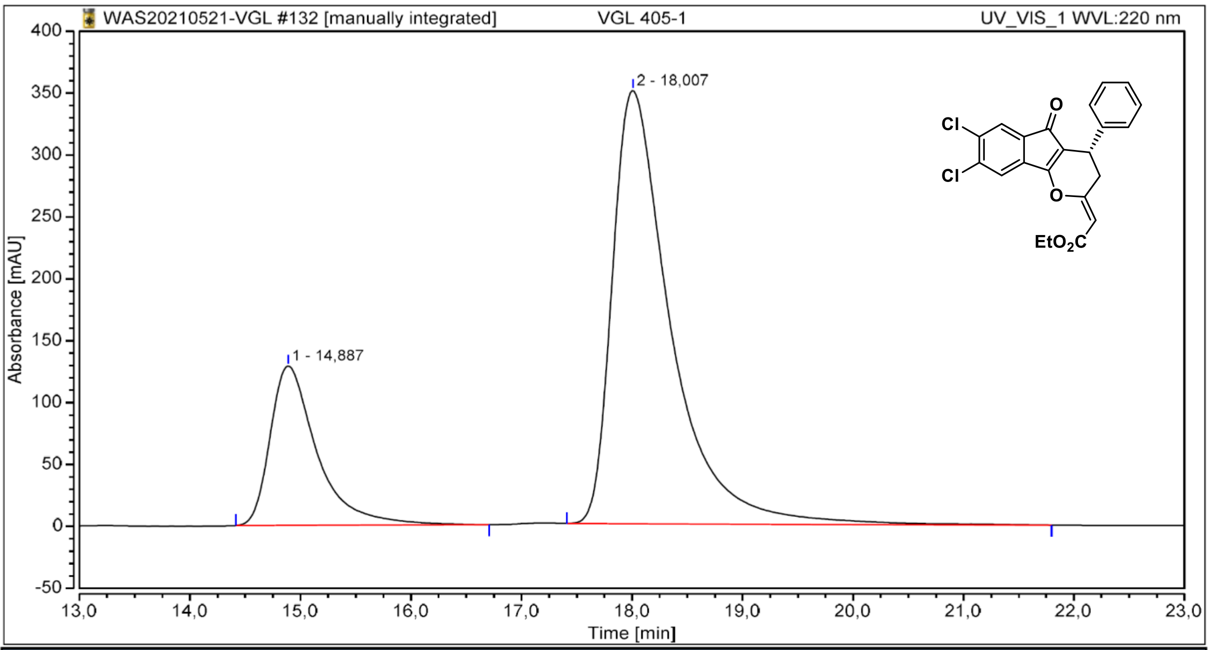

Integration Results							
No.	Peak Name	Retention Time min	Area mAU*min	Height mAU	Relative Area %	Relative Height %	Amount n.a.
1		14,887	63,259	128,796	22,78	26,91	n.a.
2		18,007	214,437	349,844	77,22	73,09	n.a.
**Total:**			**277,696**	**478,640**	**100,00**	**100,00**	

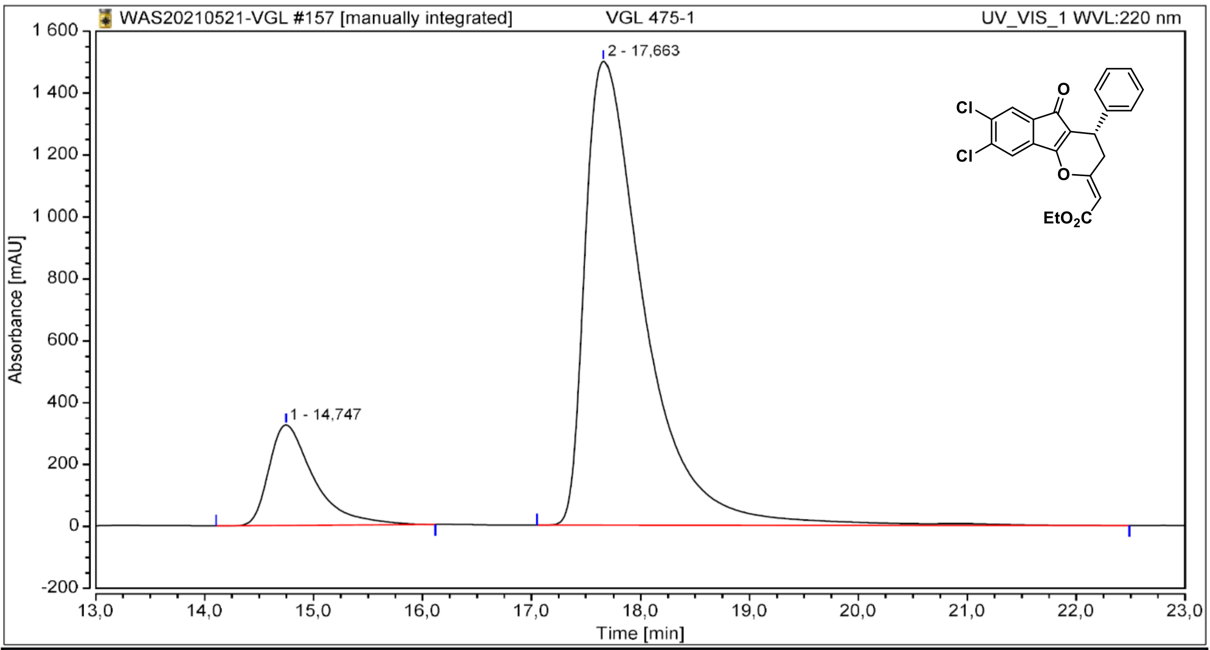

Integration Results							
No.	Peak Name	Retention Time min	Area mAU*min	Height mAU	Relative Area %	Relative Height %	Amount n.a.
1		14,747	154,898	324,620	14,14	17,80	n.a.
2		17,663	940,281	1498,729	85,86	82,20	n.a.
**Total:**			**1095,179**	**1823,349**	**100,00**	**100,00**	

Chromatograms of **3y**

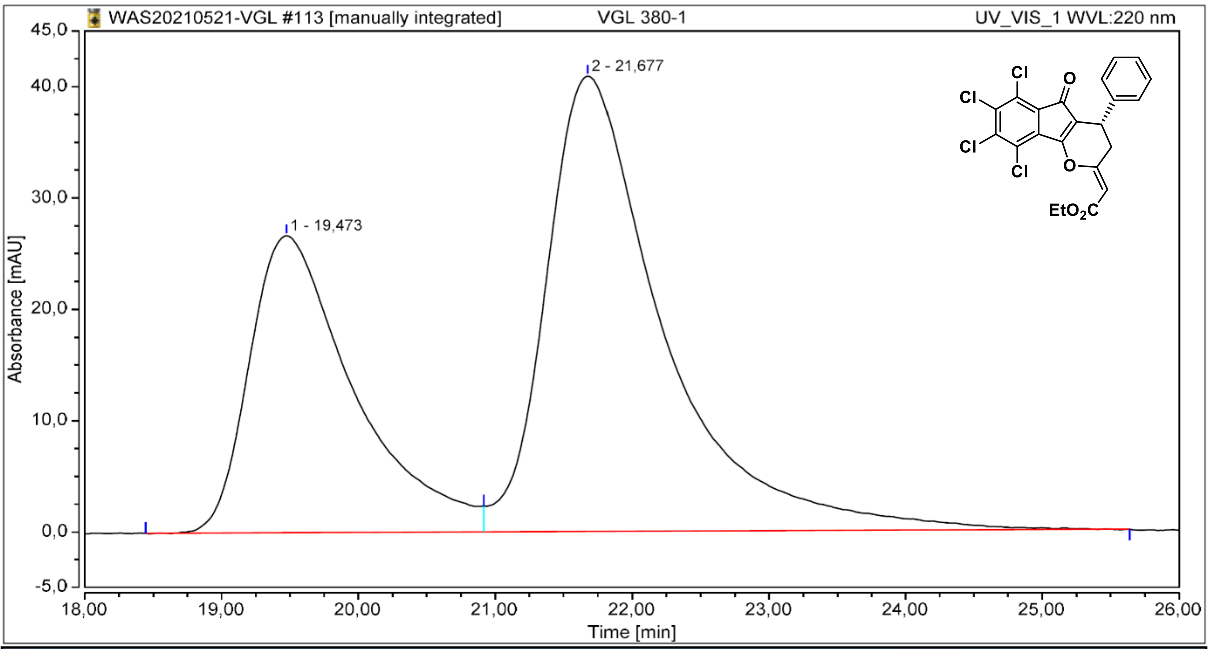

Integration Results							
No.	Peak Name	Retention Time min	Area mAU*min	Height mAU	Relative Area %	Relative Height %	Amount n.a.
1		19,473	23,584	26,663	36,76	39,47	n.a.
2		21,677	40,575	40,881	63,24	60,53	n.a.
**Total:**			**64,158**	**67,543**	**100,00**	**100,00**	

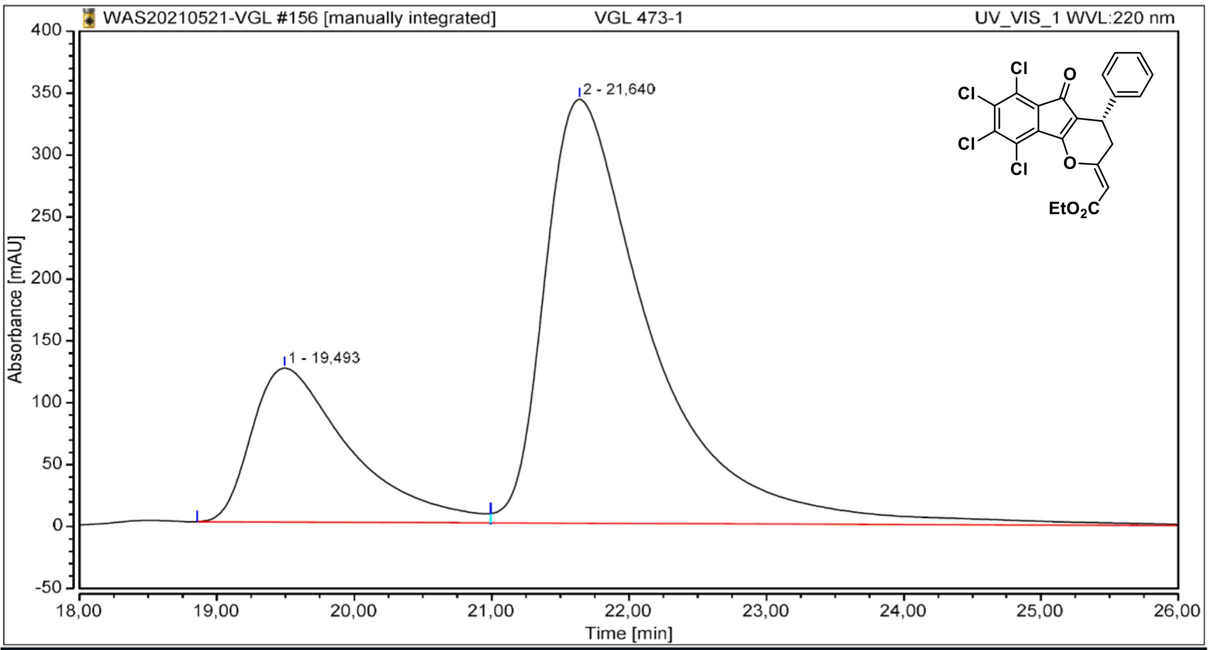

Integration Results							
No.	Peak Name	Retention Time min	Area mAU*min	Height mAU	Relative Area %	Relative Height %	Amount n.a.
1		19,493	103,014	124,283	24,94	26,64	n.a.
2		21,640	310,096	342,292	75,06	73,36	n.a.
**Total:**			**413,110**	**466,576**	**100,00**	**100,00**	

Chromatograms of **3z**

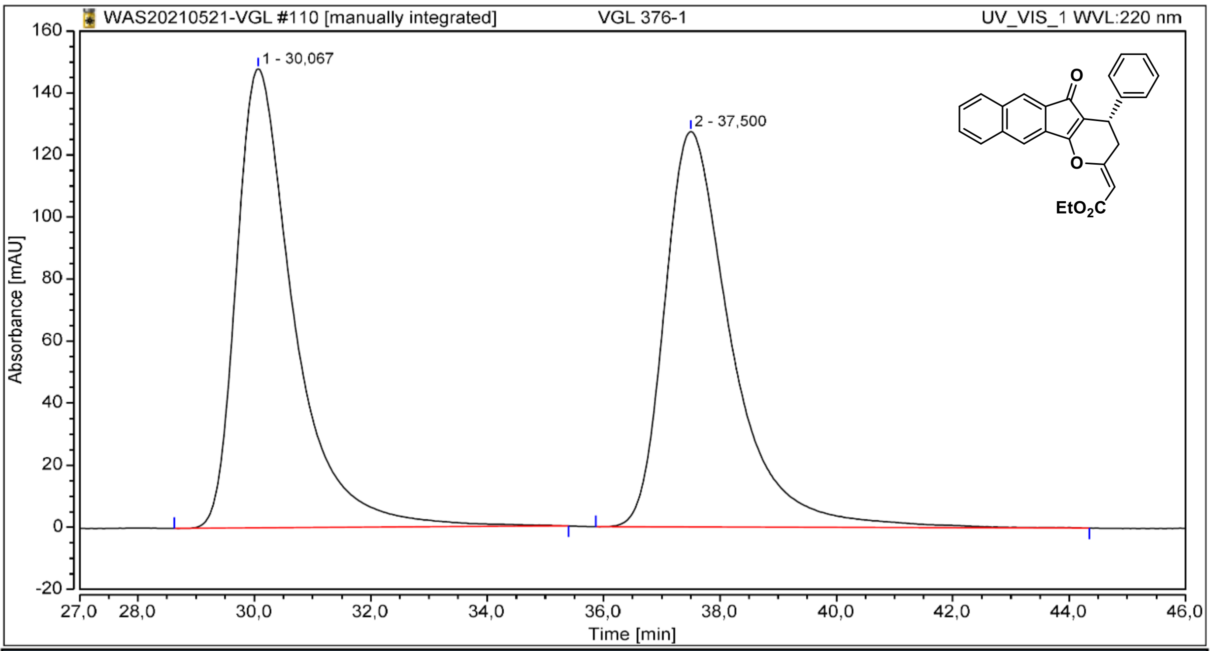

Integration Results							
No.	Peak Name	Retention Time<br>min	Area<br>mAU*min	Height<br>mAU	Relative Area<br>%	Relative Height<br>%	Amount<br>n.a.
1		30,067	169,650	148,004	49,91	53,72	n.a.
2		37,500	170,284	127,482	50,09	46,28	n.a.
**Total:**			**339,934**	**275,486**	**100,00**	**100,00**	

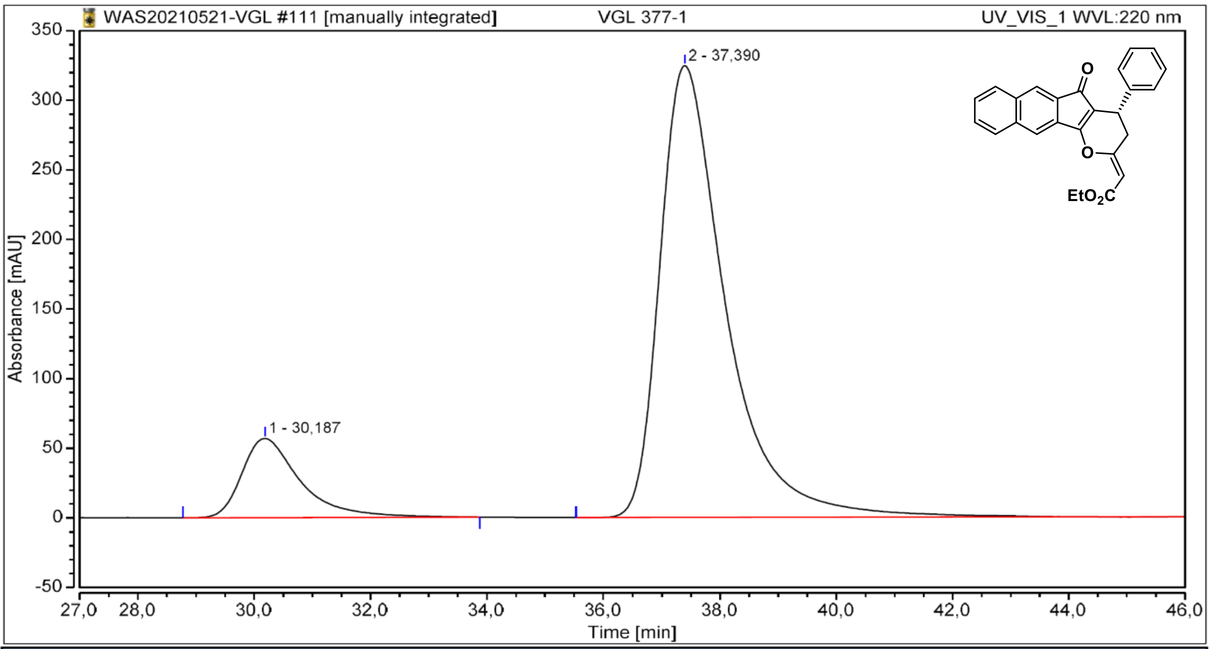

Integration Results							
No.	Peak Name	Retention Time<br>min	Area<br>mAU*min	Height<br>mAU	Relative Area<br>%	Relative Height<br>%	Amount<br>n.a.
1		30,187	66,228	56,914	13,46	14,92	n.a.
2		37,390	425,723	324,520	86,54	85,08	n.a.
**Total:**			**491,952**	**381,433**	**100,00**	**100,00**	

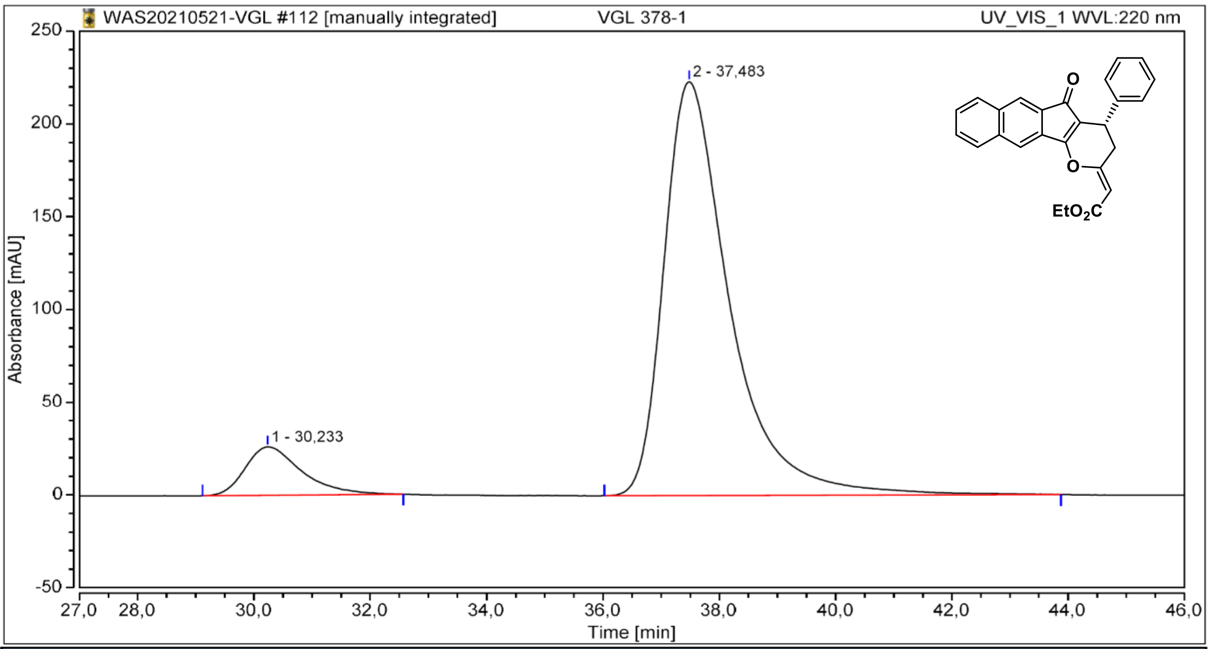

Integration Results

No.	Peak Name	Retention Time min	Area mAU*min	Height mAU	Relative Area %	Relative Height %	Amount n.a.
1		30,233	29,410	26,043	9,13	10,46	n.a.
2		37,483	292,652	223,042	90,87	89,54	n.a.
**Total:**			**322,062**	**249,085**	**100,00**	**100,00**	

Chromatograms of $\mathbf{8}_{major}$

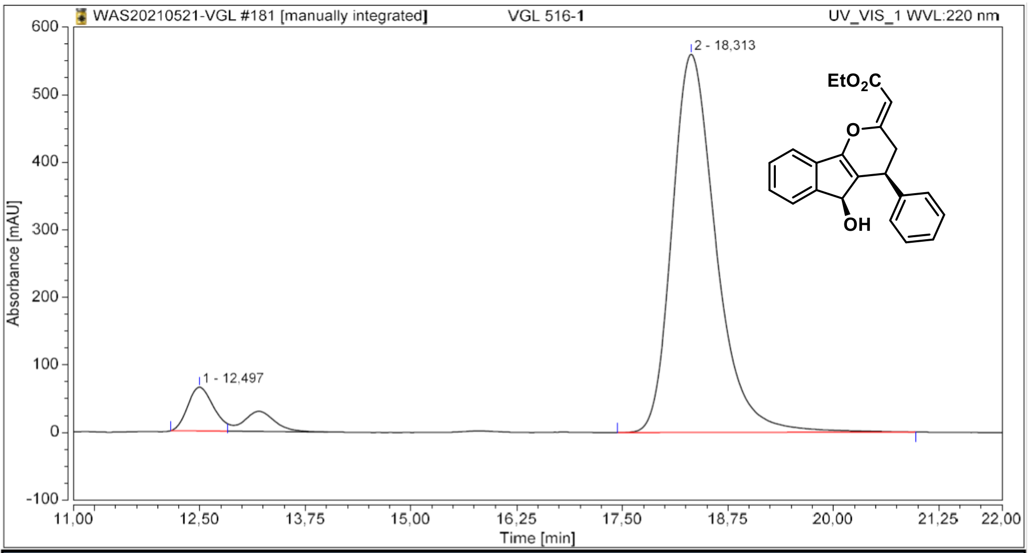

Integration Results							
No.	Peak Name	Retention Time min	Area mAU*min	Height mAU	Relative Area %	Relative Height %	Amount n.a.
1		12,497	22,677	64,761	6,30	10,37	n.a.
2		18,313	337,189	559,949	93,70	89,63	n.a.
**Total:**			**359,866**	**624,710**	**100,00**	**100,00**	

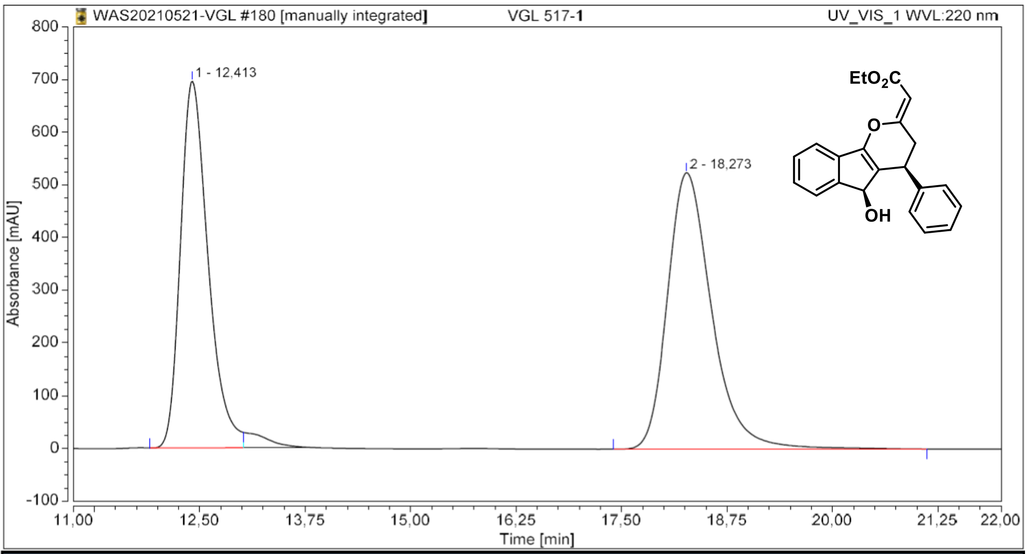

Integration Results							
No.	Peak Name	Retention Time min	Area mAU*min	Height mAU	Relative Area %	Relative Height %	Amount n.a.
1		12,413	264,605	696,310	45,36	57,04	n.a.
2		18,273	318,722	524,334	54,64	42,96	n.a.
**Total:**			**583,326**	**1220,644**	**100,00**	**100,00**	

Chromatograms of **8**$_{minor}$

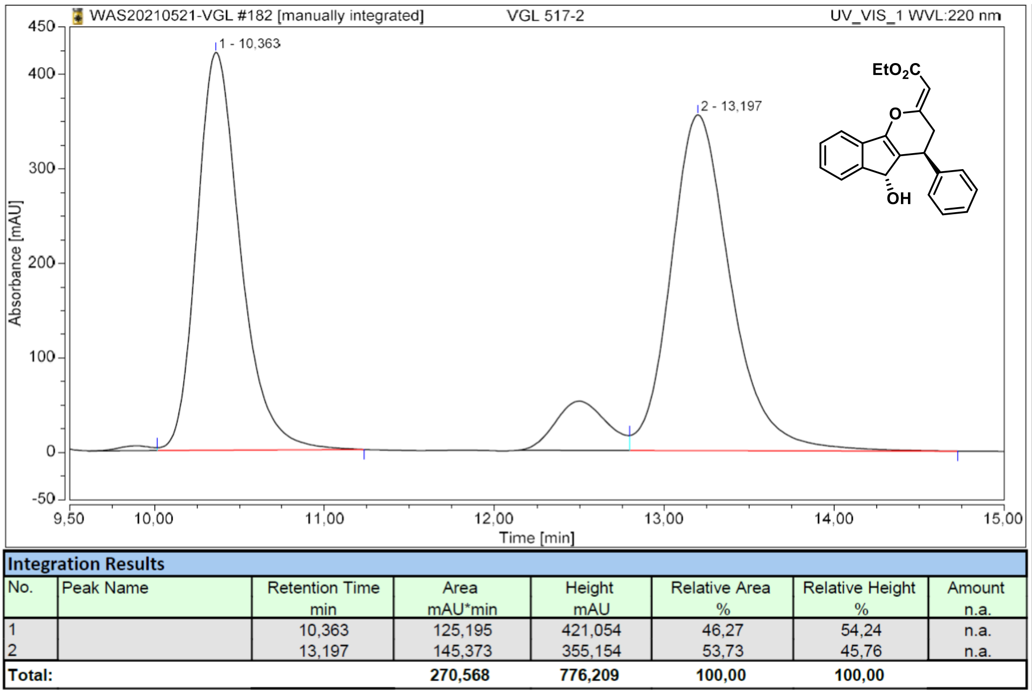

Integration Results							
No.	Peak Name	Retention Time min	Area mAU*min	Height mAU	Relative Area %	Relative Height %	Amount n.a.
1		10,363	125,195	421,054	46,27	54,24	n.a.
2		13,197	145,373	355,154	53,73	45,76	n.a.
**Total:**			**270,568**	**776,209**	**100,00**	**100,00**	

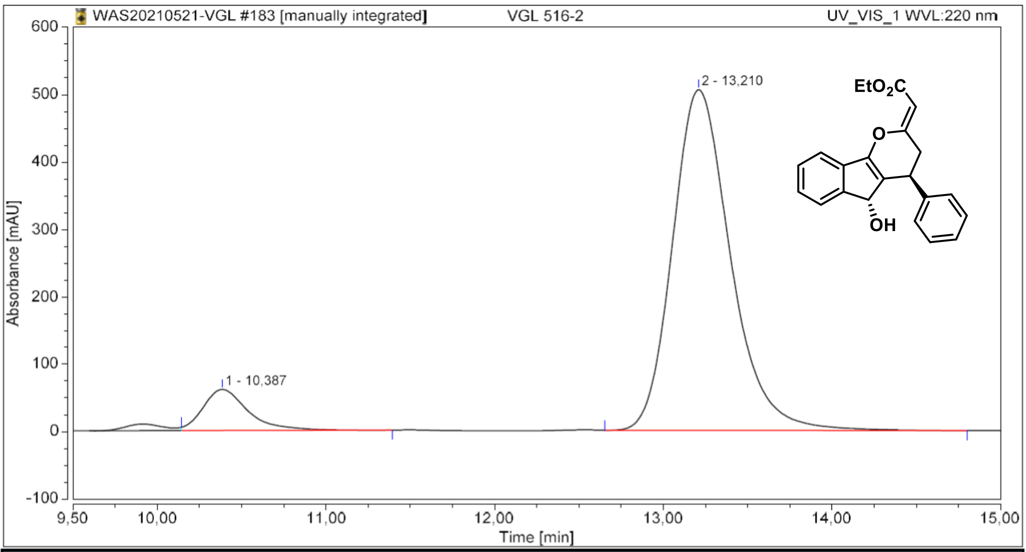

Integration Results							
No.	Peak Name	Retention Time min	Area mAU*min	Height mAU	Relative Area %	Relative Height %	Amount n.a.
1		10,387	18,836	60,959	8,55	10,76	n.a.
2		13,210	201,382	505,533	91,45	89,24	n.a.
**Total:**			**220,218**	**566,492**	**100,00**	**100,00**	

Chromatograms of **9**$_{major}$

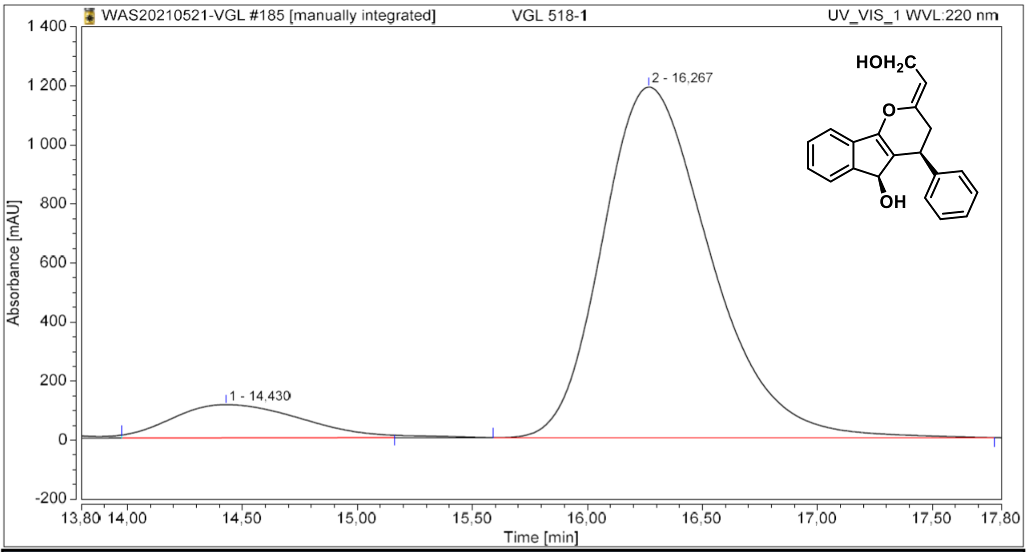

Integration Results							
No.	Peak Name	Retention Time<br>min	Area<br>mAU*min	Height<br>mAU	Relative Area<br>%	Relative Height<br>%	Amount<br>n.a.
1		14,430	72,634	112,831	10,10	8,68	n.a.
2		16,267	646,283	1187,191	89,90	91,32	n.a.
**Total:**			**718,917**	**1300,022**	**100,00**	**100,00**	

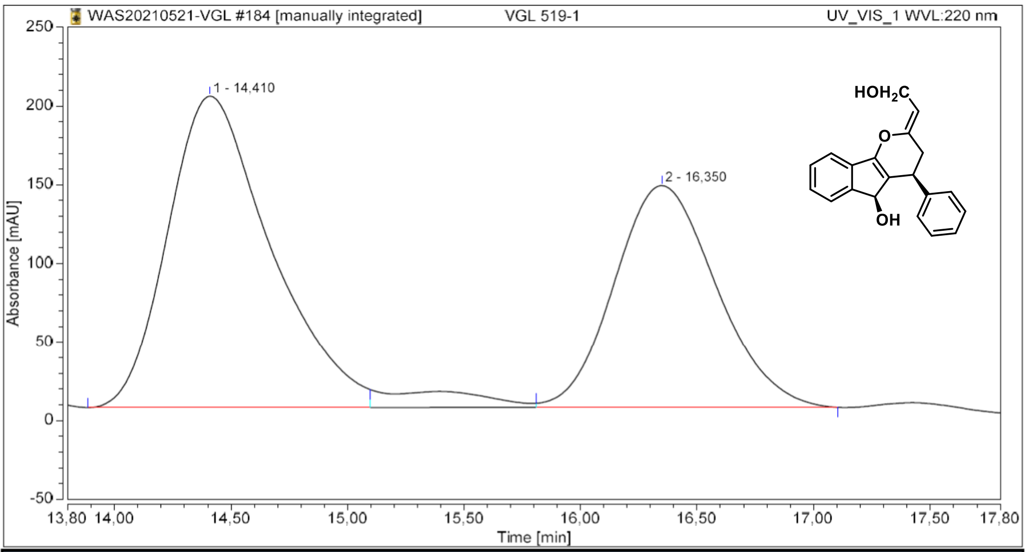

Integration Results							
No.	Peak Name	Retention Time<br>min	Area<br>mAU*min	Height<br>mAU	Relative Area<br>%	Relative Height<br>%	Amount<br>n.a.
1		14,410	101,237	197,978	58,57	58,37	n.a.
2		16,350	71,618	141,179	41,43	41,63	n.a.
**Total:**			**172,855**	**339,156**	**100,00**	**100,00**	

Chromatograms of **9**$_{\mathbf{minor}}$

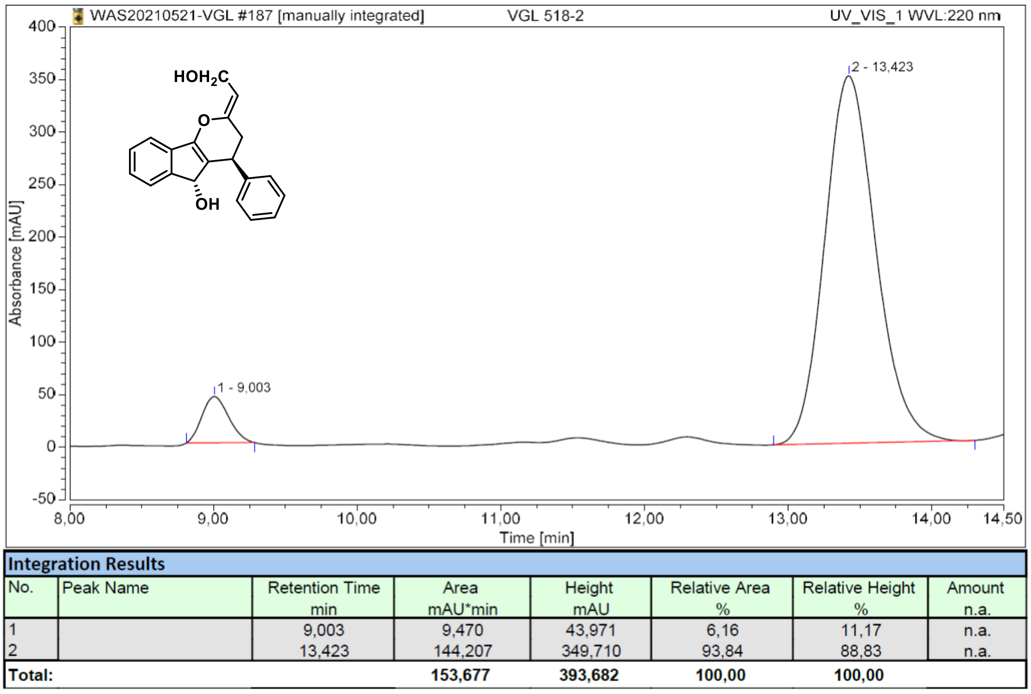

Integration Results							
No.	Peak Name	Retention Time min	Area mAU*min	Height mAU	Relative Area %	Relative Height %	Amount n.a.
1		9,003	9,470	43,971	6,16	11,17	n.a.
2		13,423	144,207	349,710	93,84	88,83	n.a.
**Total:**			**153,677**	**393,682**	**100,00**	**100,00**	

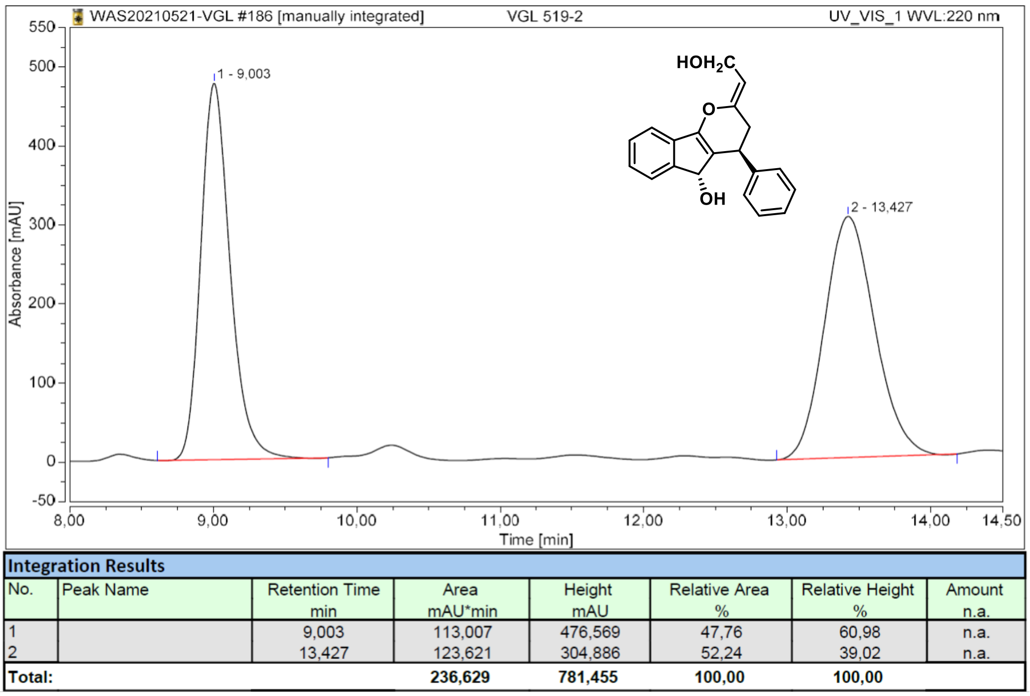

Integration Results							
No.	Peak Name	Retention Time min	Area mAU*min	Height mAU	Relative Area %	Relative Height %	Amount n.a.
1		9,003	113,007	476,569	47,76	60,98	n.a.
2		13,427	123,621	304,886	52,24	39,02	n.a.
**Total:**			**236,629**	**781,455**	**100,00**	**100,00**	

Chromatograms of **10**

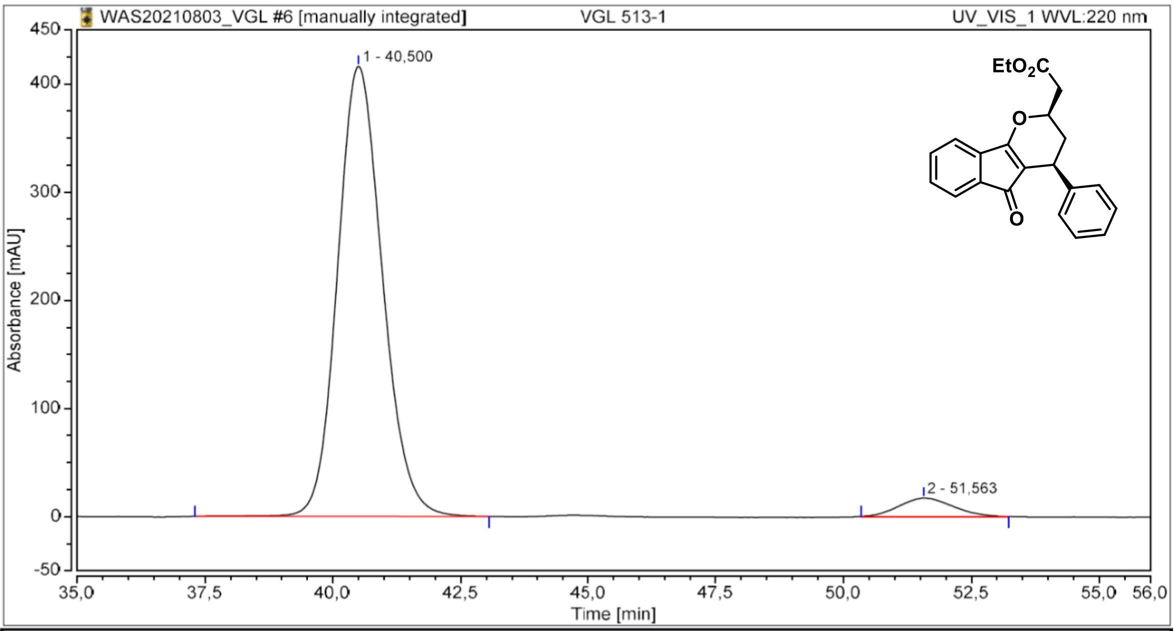

Integration Results							
No.	Peak Name	Retention Time min	Area mAU*min	Height mAU	Relative Area %	Relative Height %	Amount n.a.
1		40,500	423,547	416,074	95,33	96,10	n.a.
2		51,563	20,742	16,890	4,67	3,90	n.a.
**Total:**			**444,289**	**432,964**	**100,00**	**100,00**	

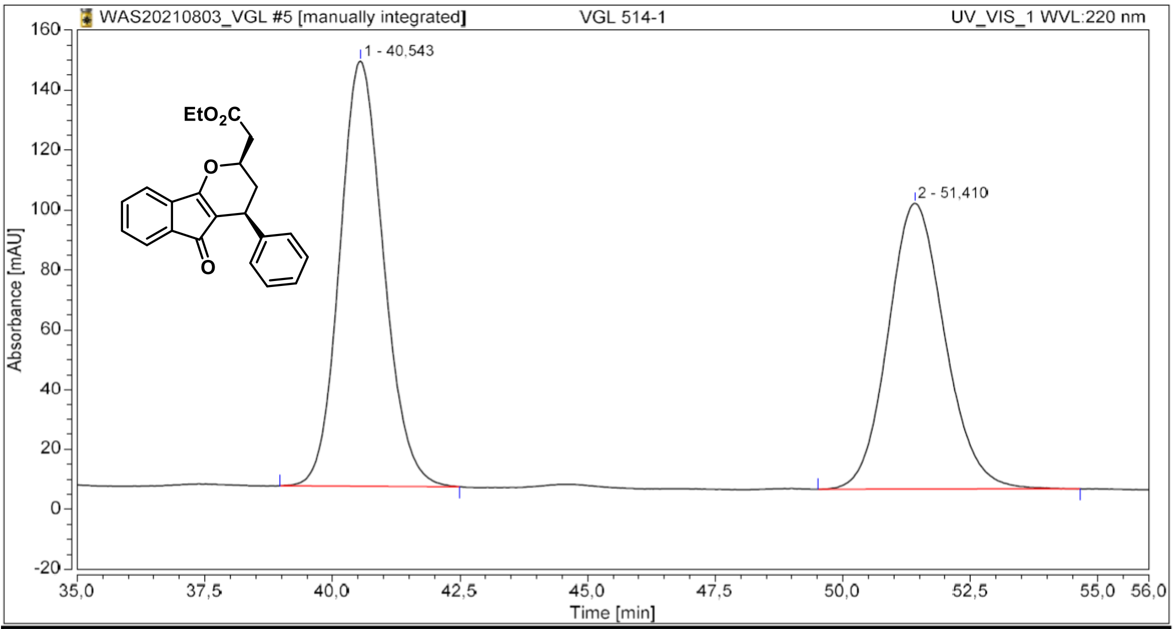

Integration Results							
No.	Peak Name	Retention Time min	Area mAU*min	Height mAU	Relative Area %	Relative Height %	Amount n.a.
1		40,543	141,442	141,998	53,44	59,77	n.a.
2		51,410	123,254	95,566	46,56	40,23	n.a.
**Total:**			**264,696**	**237,564**	**100,00**	**100,00**	

9. Computational methods

Calculations were performed using the Jaguar 8.5 pseudospectral program package.[1] Geometry optimizations were carried out at the M06-2X/6-31G(d) level of theory including an implicit description of tetrahydrofuran solvent using the Poisson–Boltzmann polarizable continuum method as incorporated in Jaguar. Electronic energies were refined by single point calculations at the M06-2X/6-311+G(d,p) level of theory.

The stationary points were characterized by full calculation of vibrational frequencies at the M06-2X/6-31G(d)(THF). IRC calculations were performed on representative transition states in order to check the energy profiles connecting each TS to both associated minima.

The reactants show no stereochemical centres and the different cycloaddition products possess only one asymmetric carbon centre. Some intermediates on the path toward cycloadducts have however two (or three) stereogenic centres, thus existing in two (or four) diastereomeric forms. We have systematically explored all possible conformers and diastereomers for all minima and transition states, with the data presented in the text referring to the lowest free energy pathway (see the following sections for full data).

NCI calculations were carried out using the free open-source program IGMPlot 1.0.[2] Results were visualized using VMD software.[3]

---

[1] Jaguar 8.5; Schrodinger, Inc.: New York, NY, 2014.

[2] IGMPlot is a software which enables a graphical visualization of NCI in molecules; each NCI being depicted by a green surface. See (a) Lefebvre, C.; Rubez, G.; Khartabil, H.; Boisson, J.-C.; Contreras-García, J.;, Hénon, E. *Phys Chem Chem Phys* **2017**, *19*, 17928; (b) Lefebvre, C.; Khartabil, H.; Boisson, J.-C.; Contreras-García, J.; Piquemal, J.-P.; Hénon, E. *Chem Phys Chem* **2018**, *19*, 724.

[3] Humphrey, W.; Dalke, A.; Schulten, K. *J. Molec. Graphics* **1996**, *14*, 33.

9.1 Full data for the ITU1-catalysed cycloadditions

9.1.1 Mechanism

We have explored all the possible mechanisms and products for the reaction of methyl allenoate **1b** with **2a** catalysed by **ITU1** (Scheme S1).

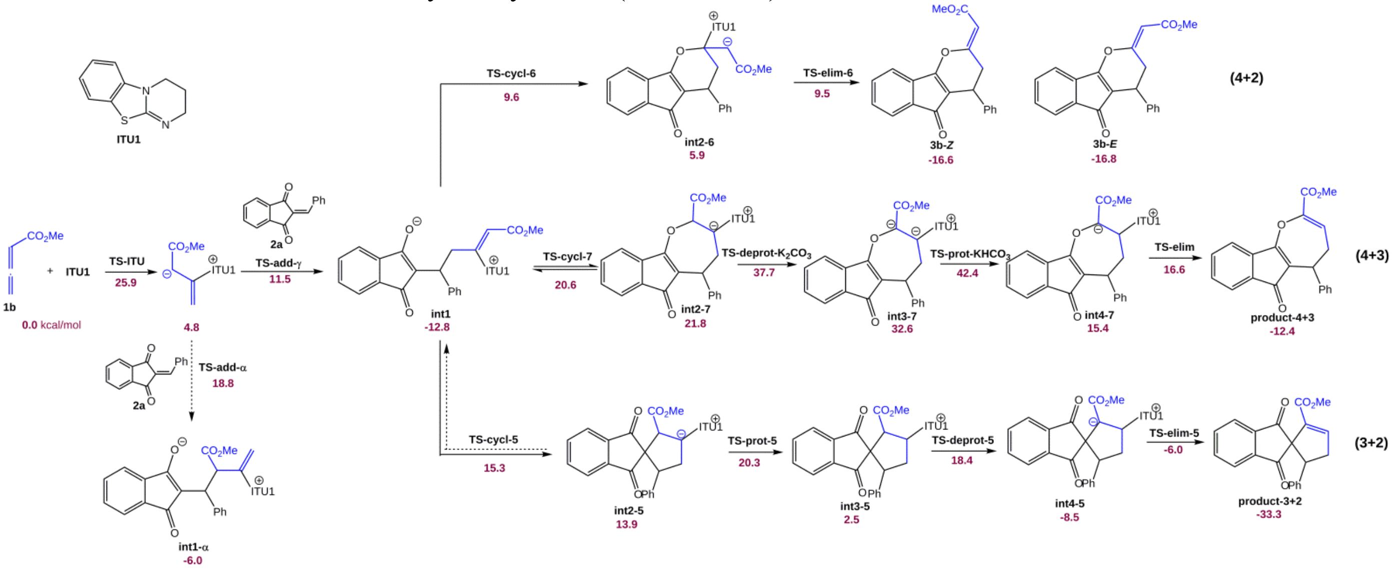

Scheme S1. Possible products and mechanisms for the reaction of methyl allenoate **1b** and **2a** catalysed by **ITU1.**

In the case of the (3+2) cycloaddition, different possibilities were explored for the proton transfer mechanism (**int2-5** -> **int4-5**). First, we envisaged an intramolecular 1,2-H-migration as assumed by Anwar for the PPh_3-catalysed (3+2) cycloaddition.[4] This mechanism involves however a very high free energy barrier (Scheme S2). Next, we investigated the possibility of an acid-catalysed protonation-deprotonation mechanism. A series of potential acids were envisaged but the one providing the most favourable pathway is **3b**.[5] The possibility of going through a base-catalysed deprotonation-protonation pathway was also examined but all the investigated bases led to a less favourable pathway than the protonation-deprotonation mechanism.

[4] S. Anwar, L.-T. Lin, V. Srinivasadesikan, V. B. Gudise, K. Chen, *RSC Adv.* **2021**, *11*, 38648-38653.

[5] Frequency calculations of the two proton transfer transition states involving **int2-5** and **3b** was not possible due to the size of these latter (>100 atoms). Accordingly, $\Delta S^{\ddagger}$ for these steps was estimated using simplified models.

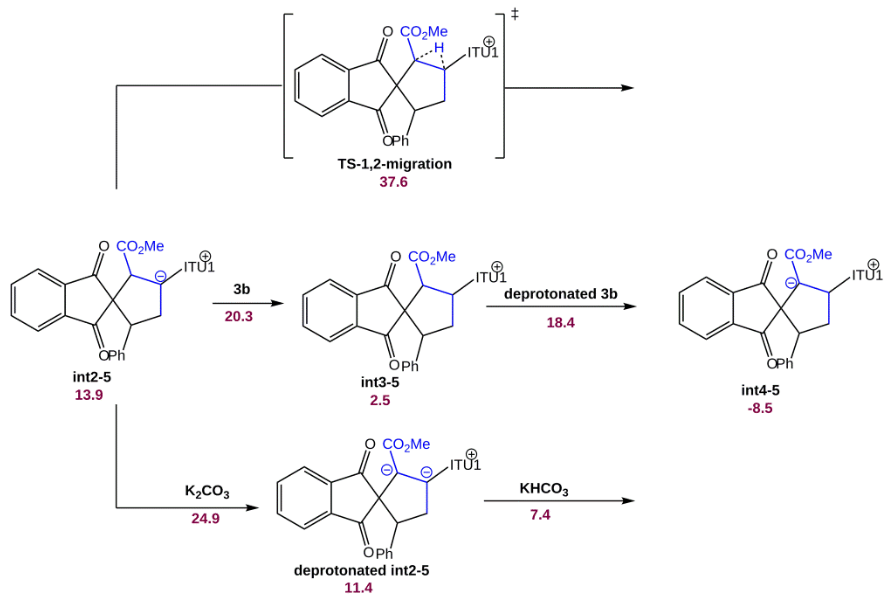

Scheme S2. Mechanism of the proton transfer for the (3+2) cycloaddition.

For the (4+3) annulation, that is the deprotonation-protonation mechanism involving K_2CO_3 which was found to be the most favourable for the proton transfer (see Scheme 1).

9.1.2 Diastereomeric pathways

All possible diastereomeric forms of each minima and transition state were explored (Tables S1).

Table S1. Diastereomeric pathways (electronic energies relative to reactants obtained at the M06-2X/6-31G(d)(THF) level).

	(4+2) annulation			
	Dia 1	Dia2	/	/
**TS-cycl-6**	-29.5	-25.5	/	/
**Int2-6**	-34.2	-29.9	/	/
**TS-elim-6-*Z***	-29.0	-24.9	/	/
**TS-elim-6-*E***	-28.7	-26.3	/	/
	(3+2) annulation			
	Dia 1	Dia2	Dia3	Dia4
**TS-cycl-5**	-22.9	-17.5	/	/
**Int2-5**	-25.6	-20.2	/	/
**TS-prot-5**	-32.6	-24.7	-24.2	-30.9
**Int3-5**	-17.5	n.d.	n.d.	n.d.
**TS-deprot-5**	-37.6	n.d.	n.d.	n.d.
**Int4-5**	-45.8	n.d.	n.d.	n.d.
**TS-elim-5**	-43.0	n.d.	n.d.	n.d.
	(4+3) annulation			
**TS-cycl-7**	-18.5	-15.5	/	/
**Int2-7**	-18.9	-16.4	/	/

**TS-deprot-7**	-17.8	n.d.	/	/
**Int3-7**	-2.2	/	/	/
**TS-prot-7**	-12.3	-13.7	/	/
**Int4-7**	-23.3	-24.6	/	/
**TS-elim-7**	-21.5	-22.0	/	/

9.1.3 Benchmark calculations

In order to identify the most suitable method to describe the reaction of interest, we have performed single point calculations on most relevant M06-2X/6-31G(d)(THF) geometries (Table S2). *ab initio* calculations were carried out using ORCA 4.1.0 program package.[6]

Table S2. Benchmark calculations (free energy relative to reactants)

	**TS-ITU**	**TS-add-γ**	**TS-cycl-6**	**TS-cycl-5**	**TS-elim-*E***	**TS-elim-*Z***	**(*E*)-3b**	**(Z)-3b**
M06-2X/6-31G(d)	25.0	10.8	6.9	10.9	5.6	5.8	-21.5	-20.5
M06-2X/6-311+G(d,p)	25.9	11.5	9.6	15.3	9.5	9.5	-16.8	-16.6
M06-2X/Def2-TZVP	29.6	13.1	14.7	18.4	13.9	13.9	-16.3	-16.2
B3LYP-D3/6-311+G(d,p)	26.2	12.7	19.9	24.3	23.0	23.0	-5.7	-5.6
SCS-MP2/6-311+G(d,p)	25.6	3.7	-0.6	0.5	-4.2	-2.0	-17.7	-19.6
LCCSD/cc-pVTZ	27.5	n.d.	12.1	10.7	11.9	11.4	-17.9	-18.2

9.2 Data for the enantioselective reaction (catalysed by ITU5)

The selectivity between the two diastereomeric **TS-add-γ** with **ITU5** (see Scheme 5) can be explained by a series of stabilizing non-covalent interactions in **(*R*)-TS-add-γ**: CH···O hydrogen bonding interactions between one ketone function and the N^{+}C-H and one Ph-H, a CH-π interaction and a π(enolate)-π(dione) interaction (Figure S1). In **(*S*)-TS-add-γ**, the enolate function of the ylide is interacting with the phenyl group of the Michael acceptor instead, what is expected to be less favourable.

[6] F. Neese. *Wiley Interdiscip. Rev.: Comput. Mol. Sci.* **2018**, *8*, e1327.

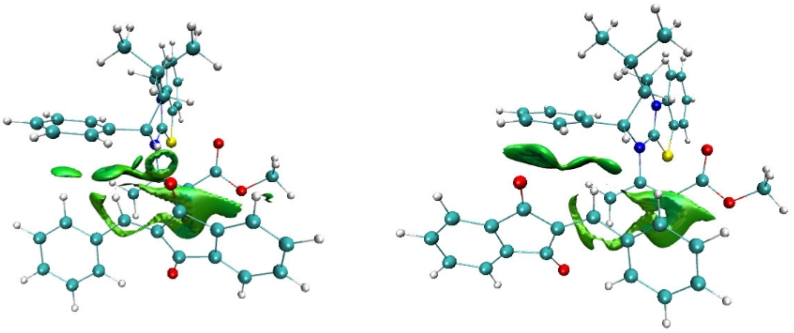

(*S*)-TS-add-γ
9.4

	Distance (Å)
C=O···HCN$^+$	2.09
C=O···H-Ph	2.61
Ph-H···π(phenyl)	2.86
π(dione)···π(enolate)	2.98

(*R*)-TS-add-γ
19.7

	Distance (Å)
C=O···H-Ph	2.37
C=O···H-Ph	2.69
π(dione)···π(phenyl)	3.26

Figure S1. Non-covalent interactions in **(*S*)-TS-add-γ** and **(*R*)-TS-add-γ** (figures made using NCIPlot)

9.3 Full data for the DABCO-catalysed cycloadditions

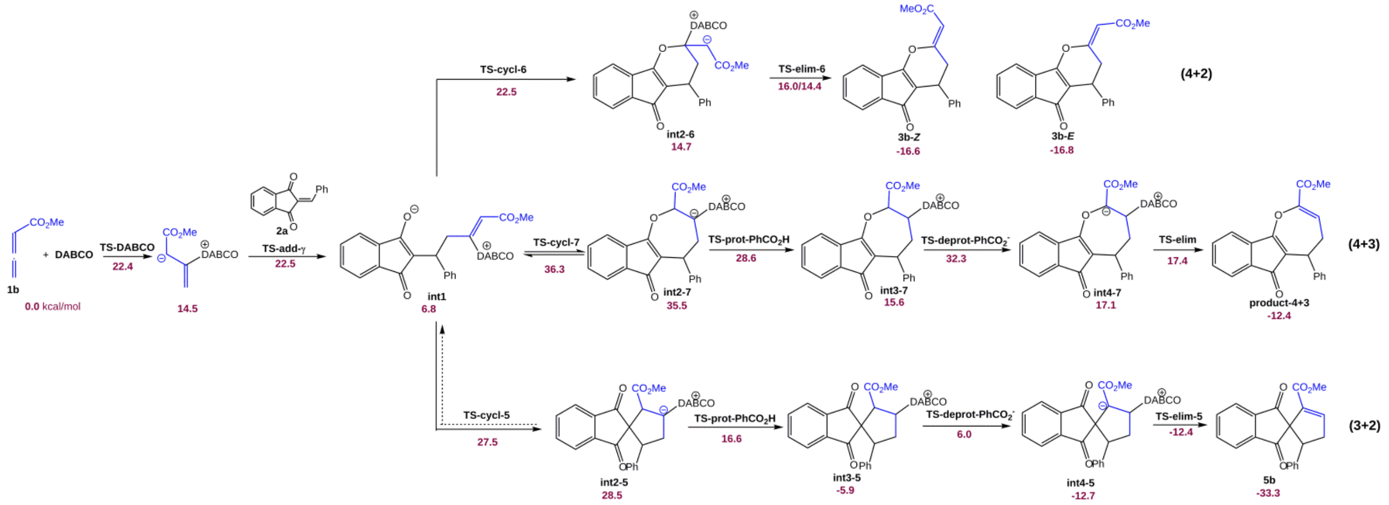

Scheme S3. Possible products and mechanisms for the reaction of methyl allenoate **1b** and **2a** catalysed by DABCO**.**

9.4 Full data for the PPh3-catalysed cycloadditions

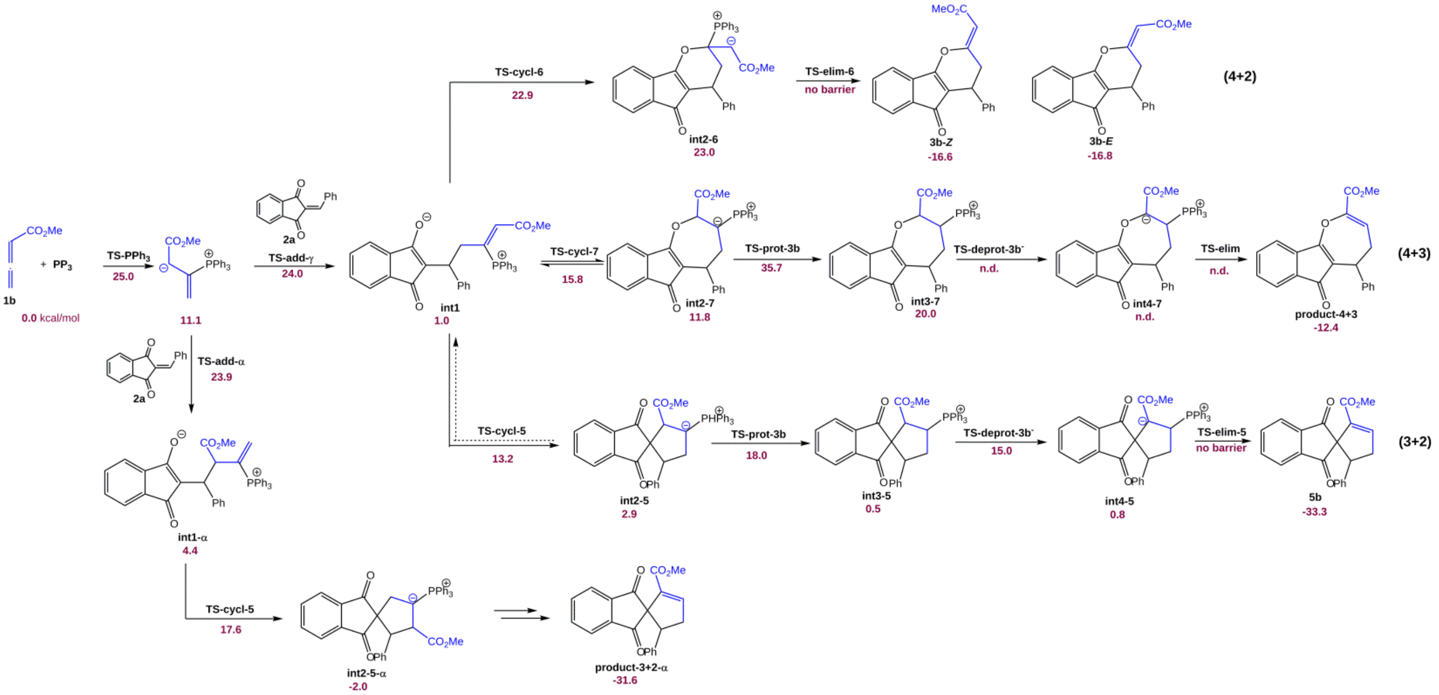

Scheme S4. Possible products and mechanisms for the PPh_3-catalysed reaction of methyl allenoate **1b** and **2a.**

9.5 Cartesian coordinates and energies

ITU1-catalysed cycloaddition

ITU1
E(M06-2X/6-31G(d)(THF)) = -894.54763724
E(M06-2X/6-311+G(d,p)) = -894.69737207
G(M06-2X/6-311+G(d,p)(THF)) = -894.552362

C1 -0.44941361 0.27968122 -2.30834158
C2 -1.69132370 0.72640528 -2.76640497
C3 -2.86028589 0.07001826 -2.38793109
C4 -2.82164851 -1.04598280 -1.55137564
C5 -1.58338909 -1.49730713 -1.10368096
C6 -0.40732109 -0.82954457 -1.47824205
H7 0.46549818 0.78979715 -2.59545726
H8 -1.74203540 1.59346528 -3.41763804
H9 -3.82074963 0.43057212 -2.74362404
H10 -3.73798147 -1.54414357 -1.25060990
S11 1.00800343 -1.59298045 -0.75510946
C12 -0.02095799 -2.80268835 0.06259338
N13 0.46773544 -3.68999693 0.83011364
C14 -0.50696265 -4.59315467 1.44482383
C15 -1.73551923 -4.82885596 0.56650483
C16 -2.38471840 -3.49815281 0.19541027
H17 -0.00473852 -5.54163489 1.65419730
H18 -0.81477392 -4.17752620 2.41491964
H19 -1.43038447 -5.34768037 -0.34879037
H20 -2.46579063 -5.45891415 1.08145031
H21 -2.90135859 -3.05288276 1.05471433
H22 -3.11361192 -3.63498974 -0.60965388
N23 -1.34646963 -2.58829047 -0.27839856

1a
E(M06-2X/6-31G(d)(THF)) = -344.389492263
E(M06-2X/6-311+G(d,p)) = -344.488687367
G(M06-2X/6-311+G(d,p)(THF)) = -344.425678

C1 2.55035569 -3.67632286 -1.31672667
H2 3.29336967 -4.44102578 -1.53272277
H3 1.76866168 -3.91432430 -0.59866402
C4 2.60140538 -2.51043054 -1.89245146
C5 2.64804352 -1.33696895 -2.47998432
H6 2.13874357 -1.15462140 -3.42283350
C7 3.40746480 -0.21434710 -1.87306879
O8 4.01325872 -0.24816203 -0.82722483
O9 3.33567757 0.87614461 -2.65355115
C10 4.03654784 2.02225144 -2.16144867
H11 3.89279564 2.80127584 -2.90914575
H12 3.62613773 2.33408958 -1.19833495
H13 5.09811181 1.79560015 -2.04151830

2a
E(M06-2X/6-31G(d)(THF)) = -765.860674007
E(M06-2X/6-311+G(d,p)) = -766.051368188
G(M06-2X/6-311+G(d,p)(THF)) = -765.883224

C1 -0.83429996 1.42281274 0.21422988
C2 -1.70287449 0.35006648 0.01736171
C3 -1.21482391 -0.93361052 -0.26633539
C4 0.15444203 -1.17833294 -0.36033368
H5 -1.19899490 2.42194969 0.43455686
H6 -2.77582102 0.50603832 0.08362728
H7 -1.91886722 -1.74779795 -0.41409054
H8 0.54613828 -2.16740123 -0.57983935
C9 1.01308853 -0.10487143 -0.16303673
C10 0.52788805 1.17037827 0.11870605
C11 2.50790072 -0.09799462 -0.20920442
O12 3.18424772 -1.08163657 -0.4429288
C13 1.67641113 2.10575390 0.28082461
O14 1.60797443 3.29149028 0.53284856
C15 4.11825766 1.93622452 0.19549194
C16 2.92801037 1.30514688 0.07608004
H17 4.00088689 2.99765745 0.43059484
C18 8.24165571 0.91532999 -0.08275549
C19 7.83648365 2.21432046 0.21992178
C20 6.48244205 2.50969596 0.30104034
C21 5.50968286 1.51596547 0.08256745
C22 5.93210452 0.21004915 -0.22324157
C23 7.28844028 -0.07998039 -0.30277456
H24 9.30005064 0.67795478 -0.14714980
H25 8.57466651 2.99254137 0.39258343
H26 6.16257137 3.52214729 0.53735955
H27 5.19476434 -0.56548249 -0.3916139
H28 7.60562127 -1.09168792 -0.5391654

TS-ITU
E(M06-2X/6-31G(d)(THF)) = -1238.92397094
E(M06-2X/6-311+G(d,p)) = -1239.1761187
G(M06-2X/6-311+G(d,p)(THF)) = -1238.94034

C1 5.08257819 -6.58590008 -4.35069662
C2 5.59883545 -6.03439390 -5.52300067
C3 6.10525387 -4.73474565 -5.53105013
C4 6.11776650 -3.95977236 -4.37335951
C5 5.61334972 -4.51671492 -3.20203843
C6 5.09473533 -5.81604636 -3.19681105
H7 4.67363313 -7.59215973 -4.34052961
H8 5.59678619 -6.62037708 -6.43675552
H9 6.49198716 -4.31269362 -6.45365906
H10 6.49993473 -2.94426207 -4.39289997
C11 4.90643046 -4.64135747 -0.99007274
C12 4.99469690 -2.90662834 0.60939743
C13 6.13602950 -2.33083723 -0.22380202
C14 5.83756905 -2.50495217 -1.70786446
H15 4.10110852 -2.27670019 0.51284859
H16 5.26601858 -2.92351246 1.66933950
H17 6.26851877 -1.26983478 0.00425858
H18 7.07369662 -2.84458017 0.01547040
H19 6.70009049 -2.21904705 -2.31503227
H20 4.96647580 -1.91396430 -2.01747171
N21 5.55162103 -3.91425158 -1.94955515
S22 4.47877655 -6.24370930 -1.60504253
N23 4.64031664 -4.27034003 0.22074702
C24 2.91553909 -5.97472817 1.21953381
C25 2.79630046 -4.75249685 0.73043381
H26 3.87156398 -6.38869343 1.51958642
H27 2.02287825 -6.58379352 1.33034928
C28 1.93853068 -3.08261036 -0.83369780
C29 1.97590445 -3.69920376 0.46808484
O30 2.60733776 -3.38693821 -1.81399509

H31	1.42534246	-3.22927186	1.27594123
O32	1.06026952	-2.04311551	-0.87717553
C33	0.97491142	-1.38349424	-2.13473201
H34	0.21061883	-0.61451687	-2.01990257
H35	1.93228797	-0.92718260	-2.40122734
H36	0.69304634	-2.08441646	-2.92406746

ITU-allene adduct
E(M06-2X/6-31G(d)(THF)) = -1238.955225048
E(M06-2X/6-311+G(d,p)) = -1239.20553053
G(M06-2X/6-311+G(d,p)(THF)) = -1238.97044

C1	4.77664829	-6.86109266	-4.23732538
C2	5.45195215	-6.59532600	-5.42576596
C3	6.28766375	-5.48177008	-5.53801805
C4	6.47746403	-4.61115579	-4.46856126
C5	5.80871259	-4.88445701	-3.27945868
C6	4.96230182	-5.99200955	-3.16967835
H7	4.11761460	-7.71963498	-4.14716543
H8	5.32020848	-7.25870321	-6.27476508
H9	6.79769334	-5.28493660	-6.47571264
H10	7.12002583	-3.74232826	-4.5679071
C11	5.06963910	-4.61867218	-1.11956461
C12	5.57757782	-2.78293017	0.36388293
C13	6.82762700	-2.60397271	-0.48485942
C14	6.50265999	-2.83349474	-1.95691225
H15	4.84064688	-2.00317179	0.15098529
H16	5.81275452	-2.78611208	1.43088887
H17	7.21466440	-1.59065062	-0.3521819
H18	7.60592161	-3.30655299	-0.1685143
H19	7.41465239	-2.83711495	-2.5577103
H20	5.80727524	-2.07995312	-2.3400217
N21	5.86710972	-4.14728005	-2.0918500
S22	4.22451737	-6.07444407	-1.57394711
N23	4.94810485	-4.07702761	0.07025363
C24	3.97368485	-5.34940617	1.82593806
C25	3.76317176	-4.43114918	0.85369023
H26	4.96514801	-5.74779316	2.00505456
H27	3.15560461	-5.69126381	2.45059312
C28	2.45826167	-2.95136554	-0.70070526
C29	2.58031584	-3.74713091	0.45438587
O30	3.31242932	-2.64057781	-1.5554471
H31	1.68639703	-3.91559723	1.04200788
O32	1.17099748	-2.47052549	-0.8479169
C33	0.95615210	-1.68836849	-2.00810343
H34	-0.09722315	-1.40268391	-1.9888560
H35	1.58440171	-0.79273051	-2.0087915
H36	1.17191173	-2.25710903	-2.9173296

TS-add-γ
E(M06-2X/6-31G(d)(THF)) = -2004.83075657
E(M06-2X/6-311+G(d,p)) = -2005.271960853
G(M06-2X/6-311+G(d,p)(THF)) = -2004.84297

C1	-0.54638628	-8.69847501	1.88711294
C2	-0.62062569	-8.98340326	3.24824457
C3	-1.04424734	-8.01264972	4.15902458
C4	-1.40713923	-6.73635732	3.73720394
C5	-1.33761368	-6.45753321	2.37615789
C6	-0.90585192	-7.42512973	1.46527495
H7	-0.21552631	-9.44826073	1.17420595
H8	-0.34230194	-9.97091963	3.60264822
H9	-1.08952163	-8.25250404	5.21663151
H10	-1.72571675	-5.98336457	4.45096242
C11	-1.44364066	-5.24969585	0.42092515
C12	-2.11879106	-2.93606395	0.20057564
C13	-2.95970830	-3.21781057	1.43812295
C14	-2.18932453	-4.06742091	2.44183299
H15	-1.27527798	-2.27078183	0.42291254
H16	-2.72914838	-2.48722396	-0.58532742
H17	-3.24605706	-2.27357232	1.90712682
H18	-3.86285844	-3.74783336	1.12675590
H19	-2.85199300	-4.42527114	3.23385083
H20	-1.35872547	-3.51708584	2.89782256
N21	-1.65279845	-5.24574774	1.75013564
S22	-0.91031770	-6.80041200	-0.18118408
N23	-1.58373304	-4.19686813	-0.34558788
C24	-1.40118468	-4.35562543	-1.77988673
H25	-2.20904646	-5.29800494	-3.51278340
C26	-2.40675526	-5.01710628	-2.48545353
C27	-1.33385341	-1.85332654	-3.38156970
H28	-1.55331962	-1.32287875	-2.45320163
C29	2.38875629	-0.49750489	-5.00845868
C30	1.57186000	-1.32244242	-5.77857387
C31	0.35957822	-1.79052158	-5.28058195
C32	-0.05861184	-1.43687661	-3.98994346
C33	0.77366299	-0.60290649	-3.22535119
C34	1.98330056	-0.13770256	-3.72412038
H35	3.33308650	-0.13577066	-5.40568963
H36	1.87979170	-1.60610864	-6.78133479
H37	-0.26228231	-2.44402205	-5.88094960
H38	0.45708148	-0.31935126	-2.22343962
H39	2.60885172	0.50816722	-3.11399596
C40	-2.46369417	-2.43120301	-3.92796253
C41	-4.77201402	-3.06014191	-4.00895499
C42	-4.14500703	-3.64479971	-5.10565480
C43	-4.84483806	-4.42848386	-6.00408682
C44	-6.21522258	-4.60284945	-5.78139450
C45	-6.84609611	-4.00931582	-4.68581880
C46	-6.12493789	-3.22759579	-3.77591067
H47	-4.33975866	-4.88830798	-6.84896757
H48	-6.79961248	-5.20778678	-6.46930963
H49	-7.91183685	-4.16257097	-4.53800871
H50	-6.60017668	-2.76856856	-2.91329789
C51	-2.67854657	-3.31305122	-5.08389664
O52	-1.87513556	-3.77577311	-5.88508069
C53	-3.74315105	-2.32009100	-3.20854385
O54	-3.96861089	-1.71044191	-2.16836261
C55	-0.29593879	-3.74348483	-2.32510877
H56	0.46971503	-3.31039878	-1.68990661
H57	-0.02603150	-3.97279861	-3.34941955
C58	-3.67809332	-5.40916227	-1.92998585
O59	-4.09856850	-5.22623946	-0.78962281
O60	-4.43424004	-6.04821020	-2.85961913
C61	-5.71897706	-6.45978439	-2.41382733
H62	-6.21057849	-6.89333799	-3.28519091
H63	-5.63311979	-7.20209653	-1.61474706
H64	-6.29269965	-5.60674706	-2.04364042

TS-add-α
E(M06-2X/6-31G(d)(THF)) = -2004.82101265
E(M06-2X/6-311+G(d,p)) = -2005.2599125922

G(M06-2X/6-311+G(d,p)(THF)) = -2004.831236266

C1	-3.02326978	0.86921869	-5.02097810
C2	-2.78015040	2.01483565	-5.77385541
C3	-1.50790464	2.59320067	-5.80879898
C4	-0.44595827	2.04352699	-5.09657030
C5	-0.69552819	0.89910734	-4.34463633
C6	-1.96632441	0.32557216	-4.30086764
H7	-4.00891670	0.41310219	-4.99365173
H8	-3.59078598	2.46264278	-6.34021569
H9	-1.34063688	3.48696249	-6.40168437
H10	0.53977893	2.49738620	-5.12500846
C11	-0.29066066	-0.86749592	-2.91703819
C12	1.70490986	-1.03482666	-1.62111294
C13	2.45114420	-0.54580634	-2.85221434
C14	1.65486657	0.56505591	-3.52141516
H15	1.53846429	-0.22040188	-0.90391554
H16	2.24418900	-1.84116107	-1.13116300
H17	3.43562225	-0.16489448	-2.56971304
H18	2.59682697	-1.39420770	-3.52691173
H19	1.98532342	0.72527575	-4.55203790
H20	1.74011139	1.51076233	-2.97433663
N21	0.23128217	0.19385010	-3.56693867
S22	-1.98206969	-1.12367390	-3.29811679
N23	0.39661269	-1.57479869	-2.03701598
C24	-0.17291323	-2.74755014	-1.38254854
H25	-0.27447673	-3.76057643	0.45819944
C26	0.08587544	-2.88543101	-0.06496016
H27	0.57742730	-2.10625797	0.50786656
C28	0.58882883	-5.59745780	-2.22159770
H29	-0.18506755	-6.20047930	-1.74178800
C30	0.52302305	-6.15821863	-6.46280397
C31	1.60661228	-5.50126481	-5.88153282
C32	1.65376592	-5.28962412	-4.50814209
C33	0.60922978	-5.73951498	-3.68640315
C34	-0.47414083	-6.40598818	-4.28254060
C35	-0.51917709	-6.60958292	-5.65637303
H36	0.49341996	-6.32119846	-7.53674785
H37	2.42616579	-5.15183662	-6.50401338
H38	2.49188039	-4.76586646	-4.06481957
H39	-1.29174062	-6.75075528	-3.65692579
H40	-1.36862859	-7.12384005	-6.09690899
C41	1.59208520	-5.24402139	-1.33357991
C42	2.67504567	-5.08130919	0.79660276
C43	3.39512209	-4.29959017	-0.10280151
C44	4.53554971	-3.61767731	0.28413725
C45	4.94701819	-3.74395592	1.61583758
C46	4.22534058	-4.52922759	2.51746935
C47	3.07108442	-5.21167891	2.11531589
H48	5.09004807	-3.01569805	-0.43112206
H49	5.84122023	-3.22837984	1.95550576
H50	4.56802400	-4.60976798	3.54539007
H51	2.49941758	-5.82326260	2.80781994
C52	2.72799976	-4.33783087	-1.45132670
O53	3.11042969	-3.64748323	-2.39687733
C54	1.49369319	-5.66994236	0.07427494
O55	0.64180065	-6.37778034	0.58863714
C56	-0.84649249	-3.64410943	-2.27158110
H57	-0.69629577	-3.50799639	-3.33429136
C58	-2.02748068	-4.40763331	-1.95558649
O59	-2.77125095	-4.91836211	-2.78573784
O60	-2.24812242	-4.55840146	-0.62440848
C61	-3.37952860	-5.35211442	-0.28868468
H62	-3.41815083	-5.36996339	0.80038569
H63	-4.29613761	-4.91820609	-0.69653932
H64	-3.26825871	-6.36708942	-0.67888514

Int1

E(M06-2X/6-31G(d)(THF)) = -2004.8768258
E(M06-2X/6-311+G(d,p)) = -2005.31485836
G(M06-2X/6-311+G(d,p)(THF)) = -2004.881698373

C1	3.96445360	-1.04458147	-1.06770320
C2	4.62555264	-0.25506359	-2.00252592
C3	4.02788294	0.05927846	-3.22657880
C4	2.75806590	-0.40500546	-3.55042934
C5	2.09561833	-1.18696220	-2.60830757
C6	2.69029550	-1.49938872	-1.38593962
H7	4.42916752	-1.30456633	-0.12136178
H8	5.62258653	0.11241058	-1.78030492
H9	4.56659567	0.66754071	-3.94670511
H10	2.31482934	-0.17891859	-4.51472835
C11	0.44204523	-2.49466603	-1.67695698
C12	-1.67868559	-3.04987532	-2.76390803
C13	-1.45683416	-1.76160250	-3.53721629
C14	0.00483686	-1.67578623	-3.94882137
H15	-1.51380843	-3.90823045	-3.41832403
H16	-2.68010565	-3.08699166	-2.32827300
H17	-2.08880936	-1.77118632	-4.42866397
H18	-1.72760155	-0.88675866	-2.93627487
H19	0.22449999	-0.72288732	-4.43274912
H20	0.28712833	-2.51036085	-4.59959627
N21	0.82294616	-1.75977755	-2.73238880
S22	1.63428167	-2.50237213	-0.40378518
N23	-0.72028810	-3.14405192	-1.63377812
C24	-0.99653185	-4.13825684	-0.62820427
C25	-1.20413658	-4.68978412	1.78955849
C26	-0.99511569	-3.75448748	0.65698026
O27	-1.47557652	-5.86765840	1.71920958
H28	-0.84173777	-2.71392077	0.92580447
O29	-1.04927301	-4.04022439	2.95304938
C30	-1.19276963	-4.85235886	4.12395002
H31	-1.00936628	-4.18932360	4.96840787
H32	-2.20080496	-5.26987013	4.17285909
H33	-0.46671959	-5.66794679	4.10725101
C34	0.09630740	-6.37495259	-1.03449976
H35	0.35696859	-6.42819497	0.02932586
C36	-1.00434101	-10.35166428	-2.31253652
C37	-0.64221914	-9.39073250	-3.25571048
C38	-0.26586133	-8.11407537	-2.84762066
C39	-0.24835356	-7.78175506	-1.49053764
C40	-0.62066841	-8.74610719	-0.55277928
C41	-0.99252418	-10.02604408	-0.95869475
H42	-1.29451315	-11.34898963	-2.63186555
H43	-0.65096500	-9.63824613	-4.31409397
H44	0.01031363	-7.35866578	-3.57968266
H45	-0.61876312	-8.48374035	0.50344499
H46	-1.27255574	-10.76939187	-0.21690231
C47	1.26595560	-5.74136299	-1.72791058
C48	3.46065161	-4.88786146	-2.02294178

C49	2.72188627	-4.57076790	-3.16303082
C50	3.28399291	-3.84811336	-4.19525446
C51	4.61781101	-3.42582534	-4.05556924
C52	5.34876078	-3.73402856	-2.91215157
C53	4.77010371	-4.48455627	-1.87323335
H54	2.70883421	-3.61297629	-5.08825591
H55	5.08468660	-2.84706819	-4.84866062
H56	6.37787127	-3.39454547	-2.82730027
H57	5.32644146	-4.73850054	-0.97446014
C58	1.31105973	-5.10588427	-2.98806366
O59	0.41549952	-4.90508721	-3.84709247
C60	2.54541066	-5.64288981	-1.07773240
O61	2.88689001	-6.02011794	0.04514264
C62	-1.20728386	-5.53085497	-1.14639771
H63	-1.99289463	-6.02021636	-0.56982966
H64	-1.49855448	-5.50446212	-2.19713444

Int1-α

E(M06-2X/6-31G(d)(THF)) = -2004.86528765
E(M06-2X/6-311+G(d,p)) = -2005.3008604
G(M06-2X/6-311+G(d,p)(THF)) =
-2004.873997561

C1	-1.49183377	0.64406388	-5.25277526
C2	-1.00588390	1.88835690	-5.64282280
C3	0.00883698	2.52083946	-4.91831551
C4	0.56894795	1.92817999	-3.79124657
C5	0.08469826	0.68098824	-3.40717063
C6	-0.93730597	0.05621829	-4.12241821
H7	-2.27928220	0.14631899	-5.81104720
H8	-1.42268960	2.37203390	-6.52055786
H9	0.37058988	3.49292333	-5.23827706
H10	1.35417318	2.42666784	-3.23226295
C11	-0.15344332	-1.24429396	-2.16815114
C12	1.02735136	-1.72787544	-0.11013781
C13	2.14514722	-0.88798618	-0.71103199
C14	1.57965701	0.34222382	-1.40104435
H15	0.51308590	-1.19325063	0.69796751
H16	1.41091493	-2.67538977	0.27221025
H17	2.83848386	-0.58555626	0.07724467
H18	2.68461909	-1.48889398	-1.44606850
H19	2.34937829	0.83076379	-2.00353690
H20	1.16532266	1.07109644	-0.69540465
N21	0.50938641	-0.08347341	-2.31354794
S22	-1.35994664	-1.49180004	-3.40518977
N23	0.03923053	-2.07465896	-1.15661284
C24	-0.72543577	-3.29303101	-1.02576645
H25	-1.91931274	-4.43642043	0.26257308
C26	-1.39330105	-3.50093057	0.10671041
H27	-1.41679321	-2.76352319	0.90331659
C28	0.32554883	-5.48016785	-1.73845828
H29	-0.12367198	-5.96658331	-0.86352757
C30	0.41447995	-8.32945075	-4.99055273
C31	1.04419737	-7.09169343	-5.12035272
C32	1.02310497	-6.18085039	-4.06974991
C33	0.37045621	-6.49422608	-2.87204073
C34	-0.26105349	-7.73235522	-2.75183567
C35	-0.23659104	-8.64838325	-3.80292703
H36	0.43519671	-9.04131574	-5.81124769
H37	1.55789855	-6.83778704	-6.04386813
H38	1.52423145	-5.21930562	-4.16330240
H39	-0.76959725	-7.98074482	-1.82296151
H40	-0.72487410	-9.61292090	-3.69100968
C41	1.67337822	-4.97807610	-1.32231076
C42	3.67481692	-4.77330322	-0.07445505
C43	3.76034398	-3.90195413	-1.16047375
C44	4.88697797	-3.13328311	-1.37287746
C45	5.94356179	-3.24621001	-0.45317152
C46	5.85209640	-4.10921278	0.63499322
C47	4.70286777	-4.89242580	0.83659615
H48	4.95151261	-2.47040521	-2.23294547
H49	6.84661966	-2.65789785	-0.59437191
H50	6.68318947	-4.18164011	1.33167785
H51	4.62093101	-5.57706768	1.67684480
C52	2.47077050	-3.99942030	-1.96074534
O53	2.23913072	-3.26760944	-2.95113628
C54	2.33380847	-5.47978021	-0.15143656
O55	1.93949736	-6.30034171	0.68127598
C56	-0.60012815	-4.30266286	-2.15543897
H57	-0.13182967	-3.82415695	-3.01900260
C58	-1.96659963	-4.78911004	-2.61305142
O59	-2.53209616	-4.41291153	-3.61375618
O60	-2.49169190	-5.69053033	-1.77332432
C61	-3.73840539	-6.26016803	-2.18717216
H62	-4.02360500	-6.95531256	-1.39866017
H63	-4.49304079	-5.48003928	-2.30762804
H64	-3.60680221	-6.78649937	-3.13538388

TS-cycl-6

E(M06-2X/6-31G(d)(THF)) = -2004.844829861
E(M06-2X/6-311+G(d,p)) = -2005.28283139
G(M06-2X/6-311+G(d,p)(THF)) =
-2004.845995221

H1	1.25861038	-4.20556282	0.26903015
C2	2.31052487	-2.42966224	-0.17742197
C3	1.42514671	-1.89431947	-1.10472945
H4	0.53115736	-2.46123337	-1.33552941
C5	1.56230601	-0.60729062	-1.72539513
O6	2.49349991	0.19134617	-1.64694676
O7	0.49689079	-0.34408812	-2.53460300
C8	0.57310242	0.87996028	-3.25365018
H9	-0.34257851	0.93803504	-3.84299839
H10	0.63770952	1.73148068	-2.57154233
H11	1.44695233	0.89231140	-3.91068512
C12	2.29105382	-3.95376450	0.00512374
H13	2.47301776	-4.41474332	-0.97001059
C14	3.23424308	-4.57300549	1.06229309
C15	2.98820683	-3.86974059	2.35760567
C16	3.25368743	-4.38149434	3.69439804
O17	3.84598901	-5.40229086	4.01615008
C18	2.25237748	-2.70999028	2.45743641
O19	1.79518260	-1.95793169	1.51263838
C20	2.64325896	-3.38458143	4.66017330
C21	2.03673410	-2.37754714	3.90422162
C22	1.38770711	-1.31855819	4.50314807
C23	1.35666856	-1.28839397	5.90872386
C24	1.96101595	-2.28937812	6.66194972
C25	2.62113756	-3.36219745	6.03662705
H26	0.91434466	-0.54199854	3.90861087
H27	0.85050414	-0.47124508	6.41527380
H28	1.92011476	-2.24143547	7.74653845

H29	3.09687069	-4.15110029	6.61315888
C30	5.54476544	-0.90264778	1.09672083
C31	4.09453929	-0.69460450	0.69000325
N32	3.66155652	-1.83145345	-0.15743134
C33	6.44336753	-1.05417488	-0.12768605
H34	5.61294835	-1.79091141	1.72592168
H35	5.88763887	-0.04466639	1.68103362
H36	3.95221231	0.21258522	0.09532036
H37	3.43901217	-0.65296645	1.55172673
H38	6.75185774	-0.07990013	-0.52223005
H39	7.33757307	-1.63675631	0.11309134
C40	4.44288804	-2.08356659	-1.19394904
N41	5.74578387	-1.75564980	-1.21694916
S42	3.91404191	-2.83304800	-2.68244870
C43	6.41497245	-2.16476508	-2.37505635
C44	5.56720169	-2.81043521	-3.27576191
C45	6.05379163	-3.33731285	-4.46645376
C46	7.41149763	-3.19603646	-4.73820143
C47	8.25700947	-2.54068065	-3.83790756
C48	7.77199385	-2.01392375	-2.64529424
H49	5.39201573	-3.84497005	-5.16224573
H50	7.81674224	-3.60080007	-5.66036944
H51	9.31298440	-2.44156087	-4.06948159
H52	8.43315047	-1.50949517	-1.94807130
C53	7.38921392	-4.80731396	-0.22110595
C54	6.36344053	-5.27798085	-1.04021499
C55	5.04032820	-5.22217489	-0.60853726
C56	4.70148322	-4.66978409	0.63338328
C57	5.74426219	-4.26317643	1.46718086
C58	7.07324080	-4.32258666	1.04519748
H59	8.42105721	-4.84227523	-0.55909803
H60	6.59407608	-5.68852141	-2.01992002
H61	4.25795414	-5.60900842	-1.25800217
H62	5.51537215	-3.90037220	2.46463583
H63	7.86247942	-3.99284688	1.71684827
H64	2.89747188	-5.61571209	1.17491432

TS-cycl-6 dia
E(M06-2X/6-31G(d)(THF)) = -2004.83838526
E(M06-2X/6-311+G(d,p)) = -2005.275168626
G(M06-2X/6-311+G(d,p)(THF)) =
-2004.843092497

H1	1.08082883	-4.30597825	0.26188000
C2	2.15293536	-2.52995164	-0.20566760
C3	1.35493639	-1.93782069	-1.17792353
H4	0.46912675	-2.47106132	-1.50194548
C5	1.58197969	-0.63080831	-1.72399703
O6	2.52281938	0.13390606	-1.51981846
O7	0.60335288	-0.29729678	-2.61303624
C8	0.77541355	0.95849433	-3.25651016
H9	-0.07866523	1.07195979	-3.92513965
H10	0.79477903	1.77225031	-2.52700683
H11	1.70745082	0.98005616	-3.82793296
C12	2.09694537	-4.05697478	-0.06274793
H13	2.23501891	-4.52816498	-1.03994420
C14	3.11216990	-4.64552196	0.93693171
H15	4.12841854	-4.46933487	0.54684328
C16	2.92524397	-3.94319436	2.24379145
C17	3.47923898	-4.30789238	3.54556393
O18	4.25906656	-5.20542887	3.82256678
C19	2.14520232	-2.81959477	2.38828611
O20	1.56783008	-2.11860709	1.46438620
C21	2.91328326	-3.30918588	4.53730422
C22	2.10866587	-2.41204832	3.82926545
C23	1.45886254	-1.37213711	4.46085756
C24	1.62921705	-1.25127463	5.85120642
C25	2.42925430	-2.14364735	6.55686791
C26	3.09192654	-3.19404250	5.89735817
H27	0.83482676	-0.67911334	3.90341215
H28	1.12649413	-0.44866662	6.38355082
H29	2.54273864	-2.02629555	7.63078169
H30	3.72406530	-3.89492611	6.43584248
C31	2.59044026	-8.93611702	1.04170421
C32	3.54135304	-8.36099740	0.20286413
C33	3.71052771	-6.97878316	0.18925033
C34	2.93925740	-6.15377590	1.00880236
C35	1.98470525	-6.73941937	1.84198883
C36	1.81154339	-8.12005547	1.85985042
H37	2.46012655	-10.01458285	1.06214829
H38	4.15722574	-8.98895034	-0.43545825
H39	4.46008634	-6.53124577	-0.46144183
H40	1.38221270	-6.10566440	2.48882716
H41	1.07082549	-8.56197187	2.52076427
C42	5.36344985	-1.32962599	1.35798173
C43	3.98959139	-0.93096626	0.83769765
N44	3.52956704	-1.98246920	-0.09626887
C45	6.34595361	-1.38930161	0.19447549
H46	5.29163324	-2.30122587	1.85840103
H47	5.72063979	-0.59830575	2.08724932
H48	4.01637535	0.00589815	0.27226530
H49	3.25605118	-0.83263314	1.62857425
H50	6.68050216	-0.38846828	-0.09859762
H51	7.22258296	-1.99469251	0.44289125
C52	4.38537008	-2.29171172	-1.05081485
N53	5.70115884	-2.00334205	-0.97919644
S54	3.95496882	-3.08528650	-2.54853758
C55	6.43926434	-2.42927359	-2.08980861
C56	5.64558522	-3.07878242	-3.03582955
C57	6.19844733	-3.60770079	-4.19608320
C58	7.56932996	-3.46597514	-4.38997043
C59	8.36216434	-2.80860007	-3.44468752
C60	7.81006012	-2.27999287	-2.28203257
H61	5.57739037	-4.11638099	-4.92759270
H62	8.02641907	-3.87221335	-5.28677624
H63	9.42928796	-2.70819579	-3.61646209
H64	8.42964880	-1.76856823	-1.55244991

TS-cycl-5
E(M06-2X/6-31G(d)(THF)) = -2004.83422239
E(M06-2X/6-311+G(d,p)) = -2005.27188856
G(M06-2X/6-311+G(d,p)(THF)) = -2004.83684802

H1	2.71159513	-5.87962889	-0.15423998
C2	3.27835960	-3.84826007	-0.49620390
C3	2.25479986	-3.25765514	0.32898881
H4	1.28391764	-3.74589740	0.22764078
C5	2.09964779	-1.77341824	0.47218541
O6	2.96029870	-0.93466231	0.29977379
O7	0.86791856	-1.46978594	0.90677138
C8	0.65140929	-0.09883950	1.24656145
H9	-0.37299407	-0.04369273	1.61250889

H10	1.35372292	0.20944603	2.02485493
H11	0.78242541	0.53681725	0.36804876
C12	3.54562242	-5.20321995	0.07344887
H13	4.45875779	-5.67824347	-0.30397152
C14	3.60275439	-5.01817912	1.63816211
H15	4.57569220	-4.56673760	1.86796284
C16	2.60072434	-3.93801184	2.03781065
C17	3.12618292	-2.86740968	2.93256445
O18	4.27623128	-2.45934754	3.00548104
C19	1.22548232	-4.23012444	2.53862198
O20	0.46165352	-5.09126925	2.13830920
C21	1.98673150	-2.36382508	3.76302247
C22	0.87616474	-3.17643511	3.54696575
C23	-0.32751265	-2.94061489	4.19242486
C24	-0.38721516	-1.85895870	5.07454341
C25	0.72909637	-1.04463967	5.29441841
C26	1.93788665	-1.28892213	4.63802378
H27	-1.18665271	-3.58076712	4.01330939
H28	-1.31229378	-1.64590904	5.60311101
H29	0.65129844	-0.21261877	5.98884771
H30	2.81351909	-0.66641053	4.80059772
C31	3.49511225	-8.72142438	3.86895860
C32	4.49017274	-7.77722368	4.10669700
C33	4.50449354	-6.58617798	3.38580271
C34	3.53052900	-6.31216403	2.42050473
C35	2.53549882	-7.26922925	2.19077218
C36	2.51892948	-8.46115319	2.90959115
H37	3.47955185	-9.65339668	4.42715242
H38	5.25729201	-7.96715254	4.85268760
H39	5.28306372	-5.84951687	3.57504059
H40	1.75601228	-7.07126637	1.46244125
H41	1.73594458	-9.19035792	2.71964482
N42	4.41677545	-3.02578006	-0.83823070
C43	6.16074138	-0.82953386	-1.40430111
C44	6.69693774	-2.07172086	-0.70006485
C45	5.57929014	-2.79844828	0.04076343
H46	6.92640249	-0.38323810	-2.04254991
H47	5.80697710	-0.07903968	-0.68900935
H48	7.15141868	-2.73589010	-1.44320931
H49	7.47412262	-1.78416300	0.01276105
H50	5.23069857	-2.24123965	0.91659264
H51	5.92919322	-3.77895965	0.37420521
C52	4.24370374	-2.26197780	-1.88753061
N53	5.04079594	-1.23875576	-2.25391731
S54	2.87885167	-2.50792644	-2.94842351
C55	4.64562717	-0.61939259	-3.44622534
C56	3.49196104	-1.20253851	-3.98237099
C57	2.94157922	-0.72966082	-5.16534706
C58	3.56351536	0.34061945	-5.80621220
C59	4.71028372	0.92348101	-5.26463891
C60	5.26683008	0.45323795	-4.07699514
H61	2.04511211	-1.18412330	-5.57847941
H62	3.14710500	0.72619362	-6.73165267
H63	5.17918987	1.76068226	-5.77229383
H64	6.15359048	0.91874791	-3.65871190

TS-cycl-7

E(M06-2X/6-31G(d)(THF)) = -2004.82721724

E(M06-2X/6-311+G(d,p)) = -2005.26597697

G(M06-2X/6-311+G(d,p)(THF)) = -2004.828395721

C1	-6.26148148	-7.64603229	-4.34064290
C2	-6.67811091	-8.89375893	-4.80113296
C3	-5.75538313	-9.81157228	-5.30641270
C4	-4.39599407	-9.51265799	-5.35829676
C5	-3.98471370	-8.27054845	-4.88633454
C6	-4.90770716	-7.34293124	-4.39097850
H7	-6.97660441	-6.92683437	-3.95144833
H8	-7.73218630	-9.15105580	-4.76859460
H9	-6.09887044	-10.77579498	-5.66816683
H10	-3.68352808	-10.22572236	-5.76109438
C11	-2.57838839	-6.52757680	-4.36515714
C12	-0.23004594	-6.42577546	-4.80157687
C13	-0.24929612	-7.93868599	-4.61997137
C14	-1.49014354	-8.54459542	-5.26944562
H15	-0.16466122	-6.12911316	-5.85284043
H16	0.60000969	-5.97400513	-4.25982636
H17	0.64309096	-8.38063068	-5.07070623
H18	-0.24037982	-8.17358765	-3.55029118
H19	-1.62570022	-9.58314107	-4.95515520
H20	-1.43540178	-8.51263593	-6.36341730
N21	-2.67160719	-7.78883767	-4.83708661
S22	-4.11212990	-5.83907370	-3.89730713
N23	-1.47078498	-5.84380777	-4.24571937
C24	-1.56126606	-4.53569668	-3.63459535
C25	-1.57841328	-3.42119608	-5.84268345
C26	-1.11437921	-3.44468546	-4.40668151
O27	-1.46054346	-4.31919146	-6.64730390
H28	-1.29126561	-2.48765356	-3.91549443
O29	-2.20423658	-2.26968361	-6.12466615
C30	-2.74381180	-2.17425812	-7.44520060
H31	-3.21324201	-1.19305086	-7.50471432
H32	-1.94910986	-2.26505129	-8.18848880
H33	-3.48137425	-2.96310608	-7.61117757
C34	0.06177023	-4.72526038	-1.61225179
H35	0.07134934	-4.39341042	-0.56215137
C36	1.10436713	-8.94155913	-1.22320251
C37	1.98199036	-8.06557877	-1.85432644
C38	1.64975676	-6.71984439	-2.00253387
C39	0.43540890	-6.21328655	-1.52987746
C40	-0.43352412	-7.10840473	-0.89039870
C41	-0.10840997	-8.45379826	-0.74000492
H42	1.36347742	-9.98972490	-1.10210956
H43	2.93583615	-8.42559735	-2.23121610
H44	2.35005037	-6.05383328	-2.49770129
H45	-1.37212164	-6.74710425	-0.47764471
H46	-0.80141021	-9.12086894	-0.23399720
C47	1.10930126	-3.88980991	-2.30625594
C48	3.27181044	-2.83933952	-2.45891082
C49	2.61690954	-2.71566323	-3.68126707
C50	3.20198690	-2.06311628	-4.74702497
C51	4.48896329	-1.53400677	-4.55419551
C52	5.14401171	-1.65896718	-3.33216478
C53	4.53177370	-2.31971117	-2.25441461
H54	2.68169855	-1.96434185	-5.69511576
H55	4.98163315	-1.01575634	-5.37235543
H56	6.13795011	-1.23742952	-3.21185738
H57	5.02584528	-2.41979255	-1.29178671
C58	1.26631611	-3.36493754	-3.57450547

O59	0.52235963	-3.30259050	-4.64737402
C60	2.33593085	-3.58194084	-1.54287168
O61	2.56637178	-3.87373966	-0.38017409
C62	-1.37139143	-4.47873519	-2.14167185
H63	-1.66583503	-3.47299749	-1.81662844
H64	-2.06270454	-5.16691182	-1.63883021

Int2-6

E(M06-2X/6-31G(d)(THF)) = -2004.852250626
E(M06-2X/6-311+G(d,p)) = -2005.289902544
G(M06-2X/6-311+G(d,p)(THF)) = -2004.85178

H1	1.58261393	-4.41702407	0.27490347
C2	2.62903372	-2.58063503	0.08051455
C3	1.79124372	-2.09111210	-0.99966605
H4	0.98380010	-2.72223503	-1.34740588
C5	1.80428056	-0.72005072	-1.33715984
O6	2.60015903	0.14981651	-0.95991702
O7	0.83211852	-0.40816066	-2.26027257
C8	0.86012946	0.93001699	-2.72751590
H9	0.03768079	1.01867361	-3.43964871
H10	0.72267200	1.64011327	-1.90738505
H11	1.80887773	1.15607362	-3.22319664
C12	2.63119187	-4.12132338	0.16660588
H13	2.96132602	-4.51402365	-0.79625763
C14	3.42223827	-4.73026330	1.33963068
C15	2.97267294	-3.97253918	2.55295308
C16	2.85859199	-4.42339471	3.95188637
O17	3.24276455	-5.47402156	4.43217966
C18	2.36819555	-2.76056686	2.46027949
O19	2.20201973	-2.01244993	1.37892952
C20	2.15909559	-3.30811927	4.69894280
C21	1.86310437	-2.29726199	3.77731255
C22	1.21099280	-1.14203289	4.15416284
C23	0.85535053	-1.01702555	5.50871647
C24	1.15179510	-2.01810165	6.42753070
C25	1.81595210	-3.19106745	6.02690395
H26	0.97778312	-0.36374296	3.43343665
H27	0.33795880	-0.12219112	5.84263917
H28	0.86236961	-1.89284422	7.46684416
H29	2.04858229	-3.98252322	6.73397368
C30	6.24325151	-1.46040222	0.87665653
C31	4.73386452	-1.29901608	0.97862781
N32	4.05619302	-2.06725173	-0.07957361
C33	6.74767082	-1.10919793	-0.51063697
H34	6.51169861	-2.49345759	1.11152430
H35	6.72320354	-0.80936117	1.61196971
H36	4.40389825	-0.25876354	0.88192390
H37	4.41450402	-1.68657572	1.94274445
H38	6.75915023	-0.02796451	-0.68704529
H39	7.75766483	-1.50053734	-0.65743720
C40	4.60621156	-2.10283787	-1.27540104
N41	5.88103447	-1.72933107	-1.51579019
S42	3.79650727	-2.71093631	-2.71435806
C43	6.32644732	-1.99898084	-2.81624175
C44	5.34042456	-2.60938094	-3.58775324
C45	5.59312002	-2.97765933	-4.90358206
C46	6.85901511	-2.73027512	-5.42830256
C47	7.84070971	-2.10624954	-4.65416497
C48	7.58806859	-1.72568574	-3.33903289
H49	4.82074226	-3.44967773	-5.50472655
H50	7.08212546	-3.01994949	-6.45060893
H51	8.81858430	-1.90940759	-5.08275251
H52	8.35228944	-1.22776935	-2.75114964
C53	7.74468301	-4.96585232	0.89423619
C54	6.92722634	-4.97617133	-0.23379467
C55	5.54227980	-4.90318818	-0.09784486
C56	4.94242046	-4.79040553	1.16136404
C57	5.77491409	-4.79586014	2.28557065
C58	7.15965488	-4.88697633	2.15587856
H59	8.82456402	-5.02718643	0.79152065
H60	7.36464219	-5.05036227	-1.22620173
H61	4.93324128	-4.93081827	-0.99753525
H62	5.33626891	-4.74553200	3.27752166
H63	7.78192820	-4.89217793	3.04683624
H64	3.08273422	-5.77042529	1.44931107

Int2-6 dia

E(M06-2X/6-31G(d)(THF)) = -2004.845530250
E(M06-2X/6-311+G(d,p)) = -2005.280867486
G(M06-2X/6-311+G(d,p)(THF)) = -2004.84810

H1	1.31199096	-4.47065755	0.22159725
C2	2.35096167	-2.61997202	0.03933068
C3	1.60075662	-2.06770953	-1.07146521
H4	0.83847871	-2.68238939	-1.53223036
C5	1.73308177	-0.71545643	-1.44047075
O6	2.53862193	0.12360225	-1.01046537
O7	0.86932799	-0.37048071	-2.45782098
C8	1.02573695	0.94995589	-2.94852960
H9	0.27571155	1.07245155	-3.73211494
H10	0.86548405	1.68961469	-2.15921155
H11	2.02658574	1.10284101	-3.36366162
C12	2.35156115	-4.16306691	0.05748713
H13	2.64542325	-4.54508146	-0.92216155
C14	3.24371752	-4.77471047	1.15355342
H15	4.29672942	-4.60676125	0.88339841
C16	2.90815079	-4.04048953	2.41313259
C17	3.17582170	-4.39944814	3.82319470
O18	3.80097974	-5.34793538	4.25254846
C19	2.21752538	-2.87347567	2.40558937
O20	1.84418608	-2.15445980	1.34883605
C21	2.52803111	-3.31332683	4.65746216
C22	1.94961171	-2.38642482	3.78203723
C23	1.29254158	-1.26542632	4.24526685
C24	1.21793049	-1.09340775	5.63873288
C25	1.79015321	-2.01350902	6.51047861
C26	2.46359635	-3.14740208	6.02230726
H27	0.84696356	-0.54871529	3.56143667
H28	0.70329179	-0.22556849	6.04101961
H29	1.71666531	-1.85256480	7.58210915
H30	2.91936754	-3.87089195	6.69278929
C31	2.60150866	-9.04453464	1.26180456
C32	3.71725240	-8.51747547	0.61804474
C33	3.92746574	-7.14042803	0.60469838
C34	3.03081194	-6.27515027	1.23131657
C35	1.91186406	-6.81276604	1.87151811
C36	1.69848831	-8.18722549	1.88834635
H37	2.43658697	-10.11819114	1.27843563
H38	4.42815139	-9.17809565	0.12914768
H39	4.80336934	-6.73178159	0.10447176
H40	1.20638969	-6.14739446	2.36556476

H41	0.82697450	-8.59184249	2.39560266
C42	5.81162410	-1.62692860	1.29856221
C43	4.37784070	-1.19780123	1.02243687
N44	3.79509199	-2.11896333	0.02872507
C45	6.62541496	-1.53692319	0.01430907
H46	5.81492321	-2.65006494	1.68965937
H47	6.26359692	-0.97843066	2.05331856
H48	4.31246916	-0.18919628	0.60000877
H49	3.78133401	-1.22043763	1.92746628
H50	6.93079248	-0.50519375	-0.19132245
H51	7.52212011	-2.16157130	0.06651879
C52	4.50240379	-2.26930298	-1.07398407
N53	5.82491590	-1.99968381	-1.12928301
S54	3.85820259	-2.81839029	-2.61039152
C55	6.41366971	-2.27212993	-2.37012881
C56	5.49500009	-2.78721982	-3.28279295
C57	5.89298777	-3.15366896	-4.56330563
C58	7.23236072	-2.99419826	-4.90772275
C59	8.14833893	-2.46947287	-3.99124975
C60	7.75293123	-2.09844397	-2.70949724
H61	5.17583862	-3.55521343	-5.27374497
H62	7.56763502	-3.27983404	-5.90003817
H63	9.18771208	-2.34820962	-4.28024810
H64	8.46720647	-1.68663009	-2.00361760

Int2-5
E(M06-2X/6-31G(d)(THF)) = -2004.838598493
E(M06-2X/6-311+G(d,p)) = -2005.276773199
G(M06-2X/6-311+G(d,p)(THF)) = -2004.83914

H1	2.59980460	-5.71810555	-0.18807387
C2	3.20191296	-3.71519209	-0.66282254
C3	2.24785309	-2.99912034	0.29238491
H4	1.21837033	-3.25137370	0.00384420
C5	2.27824905	-1.48658495	0.38447154
O6	3.01898645	-0.71292723	-0.17834981
O7	1.32597079	-1.06686810	1.24314477
C8	1.33232826	0.33301940	1.53157389
H9	0.52118727	0.49354139	2.24092287
H10	2.29146306	0.61794332	1.97146316
H11	1.16665715	0.91254747	0.62093854
C12	3.43666113	-5.04030817	0.02586297
H13	4.35110994	-5.54806125	-0.30484615
C14	3.46748506	-4.75757361	1.55348933
H15	4.41896955	-4.26131895	1.77879880
C16	2.39319233	-3.64328377	1.75175224
C17	2.82956527	-2.69641298	2.86472269
O18	3.83194062	-2.00744985	2.86026614
C19	1.05187673	-4.17474635	2.25110855
O20	0.28495107	-4.86757417	1.61784212
C21	1.84884707	-2.78680879	3.97998851
C22	0.82371490	-3.66208845	3.63185762
C23	-0.22067944	-3.93687410	4.50691404
C24	-0.20150907	-3.30887279	5.75017597
C25	0.83114826	-2.42628407	6.10039648
C26	1.87190473	-2.15164692	5.21653332
H27	-1.01555870	-4.62016428	4.22242124
H28	-0.99672485	-3.50330067	6.46455381
H29	0.81506704	-1.95365350	7.07859939
H30	2.67858258	-1.47139286	5.47498886
C31	3.17093145	-8.06143701	4.32498075
C32	4.03743644	-6.99987664	4.57305445
C33	4.12360495	-5.94805823	3.66568831
C34	3.35051627	-5.93206939	2.49965812
C35	2.48002906	-7.00184030	2.26502955
C36	2.39374879	-8.05735695	3.16904693
H37	3.10167286	-8.88696931	5.02796119
H38	4.64836523	-6.99146021	5.47175097
H39	4.80436761	-5.12070838	3.86066300
H40	1.85450388	-7.00653034	1.37747670
H41	1.71318114	-8.88046709	2.96881282
N42	4.42734180	-2.96561200	-0.94157765
C43	6.20090581	-0.80163126	-1.56221162
C44	6.69390904	-1.98437153	-0.73259094
C45	5.53651150	-2.68055889	-0.01679066
H46	7.00813128	-0.39714831	-2.17728400
H47	5.79813544	-0.00292440	-0.92900163
H48	7.20066975	-2.69438010	-1.39514931
H49	7.42195899	-1.63875658	0.00633871
H50	5.14462594	-2.07544893	0.80965673
H51	5.88342078	-3.63514232	0.38920294
C52	4.32393117	-2.27153763	-2.04416244
N53	5.14272167	-1.28311022	-2.44801991
S54	2.97197283	-2.59065326	-3.09386915
C55	4.78082323	-0.74021049	-3.68856970
C56	3.63603018	-1.35554888	-4.21094654
C57	3.12553020	-0.95222125	-5.43646185
C58	3.76883641	0.07374675	-6.12888803
C59	4.90427297	0.68546223	-5.59690147
C60	5.42636704	0.28706715	-4.36721166
H61	2.23593872	-1.42593417	-5.84387653
H62	3.37793538	0.40254787	-7.08703177
H63	5.39073801	1.48751902	-6.14357518
H64	6.30562782	0.77223933	-3.95454943

Int2-7
E(M06-2X/6-31G(d)(THF)) = -2004.827924341
E(M06-2X/6-311+G(d,p)) = -2005.266239697
G(M06-2X/6-311+G(d,p)(THF)) = -2004.82659

C1	-6.27732483	-7.54687548	-4.53912190
C2	-6.68970297	-8.78996301	-5.01807451
C3	-5.75681796	-9.72094865	-5.47873735
C4	-4.39197414	-9.43953817	-5.46571814
C5	-3.98800578	-8.20193033	-4.97544316
C6	-4.91900215	-7.25851330	-4.52456912
H7	-7.00148655	-6.81856407	-4.18436676
H8	-7.74769167	-9.03347558	-5.03485564
H9	-6.09569993	-10.68122054	-5.85566582
H10	-3.66991651	-10.16261605	-5.83254188
C11	-2.58593935	-6.47789483	-4.38822721
C12	-0.22993260	-6.38915850	-4.73437068
C13	-0.26174333	-7.90321510	-4.55124856
C14	-1.48116625	-8.50898062	-5.24040294
H15	-0.13446539	-6.09459746	-5.78482368
H16	0.59203105	-5.94752261	-4.17274571
H17	0.64545636	-8.35180414	-4.96471985
H18	-0.29338807	-8.13200284	-3.48087760
H19	-1.63415094	-9.54368058	-4.91993654
H20	-1.38379371	-8.49121198	-6.33194073

N21	-2.67077694	-7.74012159	-4.86032529
S22	-4.12448799	-5.75937475	-3.98960836
N23	-1.48181978	-5.80022590	-4.21098046
C24	-1.63466497	-4.48931355	-3.60147458
C25	-1.63539539	-3.39599769	-5.77943431
C26	-1.07255061	-3.39435207	-4.37395016
O27	-1.33629478	-4.17830630	-6.65506136
H28	-1.30855633	-2.44506262	-3.88251512
O29	-2.58003409	-2.45613370	-5.92651709
C30	-3.27819875	-2.48369557	-7.17305566
H31	-3.99932519	-1.66787818	-7.12921202
H32	-2.58369435	-2.33761406	-8.00374723
H33	-3.79029253	-3.44120714	-7.29732810
C34	-0.00814138	-4.70171883	-1.55205584
H35	-0.00210620	-4.34637538	-0.50961710
C36	0.96493429	-8.92696358	-1.09446824
C37	1.85851652	-8.07677634	-1.73933672
C38	1.55112092	-6.72692534	-1.90694883
C39	0.34675242	-6.19084939	-1.44032972
C40	-0.53690244	-7.05926074	-0.78403035
C41	-0.23737917	-8.40851348	-0.61533213
H42	1.20385815	-9.97854560	-0.95950104
H43	2.80466509	-8.46049451	-2.11274578
H44	2.26477474	-6.08470477	-2.41546522
H45	-1.46732278	-6.67365458	-0.37460528
H46	-0.94215752	-9.05461659	-0.09818250
C47	1.05504388	-3.90684280	-2.26030691
C48	3.24461656	-2.91447897	-2.47947173
C49	2.55809735	-2.79083320	-3.68593721
C50	3.13055339	-2.16951777	-4.77708121
C51	4.43693791	-1.67495699	-4.62727690
C52	5.12306614	-1.79989598	-3.42245605
C53	4.52340239	-2.42672181	-2.31794335
H54	2.58906932	-2.06755610	-5.71262907
H55	4.92014148	-1.18320570	-5.46685843
H56	6.13119417	-1.40474343	-3.33611173
H57	5.04113954	-2.52534932	-1.36778634
C58	1.20080445	-3.39872922	-3.52363337
O59	0.42271733	-3.33362316	-4.59448065
C60	2.31615734	-3.61588307	-1.52602990
O61	2.55933374	-3.89676087	-0.36713494
C62	-1.43383220	-4.44436717	-2.10880898
H63	-1.72703943	-3.43921168	-1.77858393
H64	-2.13212008	-5.13012878	-1.61128016

TS-elim-6-*Z*

E(M06-2X/6-31G(d)(THF)) = -2004.84397533

E(M06-2X/6-311+G(d,p)) = -2005.283159787

G(M06-2X/6-311+G(d,p)(THF)) = -2004.84617

H1	0.90915988	-3.76937887	1.24897642
C2	2.36577360	-2.63162657	0.23783455
C3	1.60795723	-2.17393366	-0.84158435
H4	0.81189103	-2.79621416	-1.22696928
C5	1.99206413	-0.99781473	-1.55541657
O6	2.99370338	-0.31367316	-1.37407282
O7	1.11109576	-0.70387952	-2.56031315
C8	1.46157030	0.42031979	-3.35297692
H9	0.67344453	0.52066153	-4.10035394
H10	1.52062089	1.32614113	-2.74472298
H11	2.42672973	0.26854738	-3.84277205
C12	1.92552817	-3.94737838	0.87920062
H13	1.83740040	-4.69304301	0.08669894
C14	2.76950513	-4.45486061	2.06860712
C15	3.02056591	-3.24607026	2.91754368
C16	3.22768947	-3.13166516	4.37558695
O17	3.30669095	-4.03195185	5.18964211
C18	2.95980831	-1.98973098	2.41749559
O19	2.73548448	-1.61814734	1.16114785
C20	3.33059105	-1.64983249	4.67174481
C21	3.16658864	-0.96194292	3.46400713
C22	3.20591550	0.41494878	3.40005919
C23	3.42124103	1.10699955	4.60472818
C24	3.58630411	0.42742906	5.80695143
C25	3.54151127	-0.97749847	5.85458552
H26	3.07361973	0.94079631	2.45892419
H27	3.45791682	2.19259175	4.59530073
H28	3.75002738	0.99074174	6.72107528
H29	3.66592088	-1.51831823	6.78863073
C30	6.55722061	-3.05757360	-0.04045497
C31	5.25930558	-2.33473838	0.30179691
N32	4.11127361	-2.97747024	-0.34015937
C33	6.73119836	-3.16741396	-1.54758855
H34	6.52941931	-4.06180769	0.39501746
H35	7.40752350	-2.52097550	0.38897722
H36	5.27234243	-1.28255982	-0.00797659
H37	5.11105422	-2.37232902	1.38233531
H38	6.96931268	-2.19890624	-2.00296027
H39	7.53055576	-3.87259473	-1.79136503
C40	4.28592144	-3.51730518	-1.49682295
N41	5.48842682	-3.67552499	-2.12849668
S42	2.97047684	-4.19885577	-2.47778790
C43	5.43900953	-4.38959454	-3.32545156
C44	4.14432417	-4.80427631	-3.64547799
C45	3.88993001	-5.54185442	-4.79350409
C46	4.96183369	-5.86708876	-5.62322699
C47	6.25398755	-5.44264222	-5.31024885
C48	6.50965598	-4.69566848	-4.16207530
H49	2.87902023	-5.85715458	-5.03668123
H50	4.78602250	-6.44939415	-6.52238142
H51	7.07775664	-5.69177937	-5.97245543
H52	7.51582261	-4.35658714	-3.93701700
C53	6.34046349	-6.76480273	1.15050019
C54	5.24504316	-6.79684839	0.28939842
C55	4.10254527	-6.05484960	0.57774724
C56	4.03583184	-5.24108436	1.71373417
C57	5.13942515	-5.22274522	2.57197200
C58	6.27773090	-5.97993775	2.29839707
H59	7.23015923	-7.34828113	0.93038012
H60	5.27362874	-7.40786739	-0.60907872
H61	3.26154735	-6.11270798	-0.10752299
H62	5.10843786	-4.62545396	3.47805483
H63	7.11845541	-5.94983209	2.98659820
H64	2.13099276	-5.14499418	2.63889850

TS-elim-6-*E*

E(M06-2X/6-31G(d)(THF)) = -2004.843480814

E(M06-2X/6-311+G(d,p)) = -2005.280225042

G(M06-2X/6-311+G(d,p)(THF)) = -2004.84607

H1	1.02664385	-3.83347177	0.92058982
C2	2.56385242	-2.74718063	-0.01371347

C3	1.84174459	-2.23418117	-1.11462051
H4	2.08139908	-1.22571757	-1.43000077
C5	0.78020454	-2.90221688	-1.77362128
O6	0.34524035	-4.04393190	-1.60137377
O7	0.22557616	-2.09995548	-2.74275032
C8	-0.84277860	-2.69100312	-3.46519020
H9	-1.17587581	-1.93651027	-4.17974714
H10	-0.51239036	-3.58928786	-3.99468965
H11	-1.66588617	-2.96707145	-2.80038933
C12	2.07174112	-4.02690489	0.65809293
H13	2.02508293	-4.81288402	-0.09404475
C14	2.81662154	-4.46269056	1.93685286
C15	3.00820925	-3.21671946	2.74703759
C16	3.10856847	-3.03344732	4.20991618
O17	3.12660119	-3.89276791	5.07021985
C18	2.98879166	-1.98492194	2.18637435
O19	2.87035155	-1.67983292	0.89707708
C20	3.20042158	-1.53984341	4.44280748
C21	3.12677925	-0.90766223	3.19612750
C22	3.18677005	0.46531036	3.07645439
C23	3.32890686	1.21052404	4.26053906
C24	3.40423736	0.58412329	5.50016082
C25	3.33889726	-0.81680001	5.60604400
H26	3.12354967	0.95260327	2.10791209
H27	3.37965582	2.29405100	4.20553414
H28	3.51348531	1.18557113	6.39795576
H29	3.39410974	-1.31721268	6.56888522
C30	6.66294842	-3.32206278	0.05022481
C31	5.38350280	-2.52616027	0.27574638
N32	4.25631835	-3.10065071	-0.47137946
C33	6.96372692	-3.44309924	-1.43404427
H34	6.54413749	-4.32136235	0.48000889
H35	7.49686330	-2.82900061	0.55700052
H36	5.50753836	-1.47609963	-0.01670852
H37	5.13882542	-2.55724801	1.33736940
H38	7.28571539	-2.48885010	-1.86723621
H39	7.74610414	-4.18632554	-1.60975723
C40	4.50787174	-3.70316324	-1.59364307
N41	5.75147587	-3.89636741	-2.11762153
S42	3.26151851	-4.41498603	-2.62596115
C43	5.77788267	-4.60472862	-3.32081824
C44	4.50326343	-4.99751039	-3.73118401
C45	4.31934641	-5.71270362	-4.90822269
C46	5.44115492	-6.03400816	-5.66978376
C47	6.71512228	-5.63384932	-5.26094286
C48	6.90107354	-4.91178867	-4.08456704
H49	3.32416346	-6.01106749	-5.22542167
H50	5.32043223	-6.59432625	-6.59148358
H51	7.57891531	-5.88101392	-5.87054046
H52	7.89311389	-4.58996443	-3.78390190
C53	6.39273515	-6.89085712	1.43577995
C54	5.38661396	-6.91744101	0.47206063
C55	4.24756441	-6.13004473	0.62273652
C56	4.09444385	-5.28170744	1.72502843
C57	5.11032171	-5.26738596	2.68602858
C58	6.24527445	-6.06529911	2.54707711
H59	7.27812128	-7.51063925	1.32376730
H60	5.48070424	-7.56056333	-0.39907203
H61	3.47434231	-6.18728384	-0.13767952
H62	5.00795913	-4.64392003	3.56884995
H63	7.01501745	-6.03926300	3.31386420
H64	2.12774274	-5.11967128	2.48751213

TS-elim-6-*Z* dia

E(M06-2X/6-31G(d)(THF)) = -2004.845530250
E(M06-2X/6-311+G(d,p)) = -2005.280867486
G(M06-2X/6-311+G(d,p)(THF)) = -2004.84810

H1	0.59920040	-3.08383685	1.45622161
C2	2.08024856	-2.41498582	0.11542755
C3	1.39452248	-1.83773809	-0.96679454
H4	0.47684182	-2.29242389	-1.31245462
C5	1.99289785	-0.77995171	-1.70281387
O6	3.10824262	-0.28925173	-1.53021895
O7	1.18356637	-0.32949726	-2.71468098
C8	1.74370425	0.70079418	-3.51318806
H9	0.98868178	0.94908747	-4.26038853
H10	1.97943382	1.58189228	-2.91109123
H11	2.66041835	0.36411887	-4.00466733
C12	1.38134828	-3.56130672	0.85174068
H13	0.87187544	-4.22354787	0.15055072
C14	2.30479174	-4.35543398	1.79599311
H15	3.00312990	-4.95446527	1.19616078
C16	3.05564636	-3.31702304	2.57273951
C17	3.79886005	-3.42422712	3.85102125
O18	4.01614808	-4.41906537	4.51213660
C19	3.10820023	-2.02189616	2.17862421
O20	2.62942665	-1.48934770	1.05230998
C21	4.27274944	-2.02132839	4.17489125
C22	3.84846771	-1.17556897	3.14330634
C23	4.14028896	0.17309347	3.14220732
C24	4.87793951	0.67233364	4.23032281
C25	5.29842803	-0.16509549	5.25851725
C26	4.99960833	-1.53945945	5.24003296
H27	3.81017648	0.82103048	2.33522202
H28	5.12245915	1.72977261	4.26767445
H29	5.86659329	0.24839363	6.08666627
H30	5.32781788	-2.20321913	6.03530222
C31	-0.05684484	-7.11918275	4.12579872
C32	0.67942321	-7.56829034	3.03345995
C33	1.45151922	-6.67113176	2.29892499
C34	1.49949777	-5.32058149	2.64430030
C35	0.75467125	-4.87837472	3.73961621
C36	-0.01754013	-5.77056010	4.47630712
H37	-0.65637056	-7.81654071	4.70416342
H38	0.65734194	-8.61837691	2.75451689
H39	2.02974249	-7.02437382	1.44720349
H40	0.78695694	-3.82740837	4.02089278
H41	-0.58721165	-5.41397319	5.32996083
C42	6.03718983	-3.44999542	-0.05042368
C43	4.94609679	-2.40239339	-0.24287455
N44	3.65895275	-3.07863820	-0.44823294
C45	6.14325635	-4.30567479	-1.30787114
H46	5.78880466	-4.07319882	0.81621818
H47	7.00248249	-2.97438180	0.14162078
H48	5.13489869	-1.76736780	-1.11624680
H49	4.86769915	-1.74094337	0.61632474
H50	6.68276424	-3.77731237	-2.10177548
H51	6.66156425	-5.24758793	-1.10268085
C52	3.66951112	-4.04580753	-1.30870862
N53	4.79889470	-4.63165702	-1.80293672
S54	2.21878996	-4.76341262	-2.02374950

C55 4.57067657 -5.67429771 -2.70321084
C56 3.21092555 -5.91168566 -2.91583713
C57 2.78176720 -6.92937078 -3.75757452
C58 3.74639015 -7.71108876 -4.39075087
C59 5.10614832 -7.46800015 -4.18532235
C60 5.53703397 -6.44740882 -3.34059921
H61 1.72226732 -7.11048470 -3.91568319
H62 3.43428880 -8.51430235 -5.05111455
H63 5.84454684 -8.08272426 -4.69083848
H64 6.59554368 -6.25927600 -3.19027096

TS-elim-6-*E* dia
E(M06-2X/6-31G(d)(THF)) = -2004.83968898
E(M06-2X/6-311+G(d,p)) = -2005.2756182099
G(M06-2X/6-311+G(d,p)(THF)) = -2004.84484

H1 1.53967026 -5.40879418 1.18010814
C2 1.31728612 -3.29583303 1.10214664
C3 -0.05748660 -3.25239868 0.83885658
H4 -0.53394974 -2.28041211 0.80947032
C5 -0.88916726 -4.40664793 0.66228602
O6 -0.57043541 -5.58601748 0.55844205
O7 -2.20099128 -4.03336397 0.56503969
C8 -3.11588340 -5.11343609 0.43935912
H9 -4.10852144 -4.66257131 0.39676432
H10 -2.92344167 -5.68763017 -0.47093051
H11 -3.03875078 -5.78288221 1.30028326
C12 1.94575209 -4.54452164 1.70547088
H13 3.03042813 -4.51693943 1.56582655
C14 1.66424441 -4.63503325 3.23315249
H15 2.40446114 -5.33037681 3.65034789
C16 1.91804647 -3.27373628 3.79197362
C17 2.19268589 -2.87450943 5.18865365
O18 2.26634226 -3.58670708 6.16927205
C19 1.96399416 -2.15743179 3.03099829
O20 1.78120708 -2.07281921 1.71234273
C21 2.39936099 -1.37328897 5.16091638
C22 2.26212031 -0.94676778 3.83387088
C23 2.39658022 0.38171042 3.48728954
C24 2.68251941 1.29376722 4.51831225
C25 2.82136317 0.87158304 5.83572098
C26 2.67800030 -0.48557335 6.17503477
H27 2.28157683 0.71368717 2.45958234
H28 2.79490159 2.34781704 4.28062524
H29 3.04109856 1.59981617 6.61101063
H30 2.78034602 -0.82941351 7.20067170
C31 -2.23555465 -6.30120068 4.08022929
C32 -1.14193715 -7.13139206 3.84229635
C33 0.10884289 -6.57810033 3.59187889
C34 0.28963846 -5.19414570 3.57197657
C35 -0.80773367 -4.37104470 3.82440697
C36 -2.06401623 -4.91960171 4.06973320
H37 -3.21502535 -6.72911082 4.27722969
H38 -1.26460915 -8.21091356 3.85171910
H39 0.95963189 -7.22894232 3.39806667
H40 -0.68438985 -3.29230472 3.80980902
H41 -2.90984735 -4.26264531 4.25452510
C42 4.61097294 -3.11655228 -1.28389800
C43 3.71402775 -2.56922204 -0.17942188
N44 2.34474644 -3.04692938 -0.36061053
C45 4.07884119 -2.65659059 -2.63491674
H46 4.61656216 -4.21073865 -1.23607675
H47 5.63834164 -2.76493649 -1.15965169
H48 3.71523512 -1.47131008 -0.18212209
H49 4.06004767 -2.89075794 0.80516964
H50 4.30869039 -1.59974236 -2.81528667
H51 4.51322662 -3.24741789 -3.44728989
C52 1.87163735 -3.07468479 -1.56482115
N53 2.62375915 -2.83832076 -2.67935754
S54 0.18085069 -3.42137178 -1.98920795
C55 1.92297561 -2.90348762 -3.88683458
C56 0.58157760 -3.23270395 -3.69924691
C57 -0.28131423 -3.34755012 -4.78117414
C58 0.22710037 -3.12560938 -6.05989186
C59 1.57016060 -2.79230658 -6.24427742
C60 2.43725435 -2.67470027 -5.15971630
H61 -1.32685824 -3.60351521 -4.63182691
H62 -0.43087511 -3.21095912 -6.91907448
H63 1.94945846 -2.61670377 -7.24639100
H64 3.47795150 -2.40251241 -5.30742312

***Z*-3b**
E(M06-2X/6-31G(d)(THF)) = -1110.312130385
E(M06-2X/6-311+G(d,p)) = -1110.598840140
G(M06-2X/6-311+G(d,p)(THF)) = -1110.33532

C1 -0.82816058 1.35390358 0.40673565
C2 -1.63909345 0.20676031 0.34475714
C3 -1.09316763 -1.03782230 0.04999952
C4 0.28258860 -1.19183845 -0.19435956
H5 -1.24405236 2.33182494 0.63268534
H6 -2.70620479 0.29289404 0.52709943
H7 -1.74111451 -1.90840875 0.00545658
H8 0.71124382 -2.16123535 -0.43095356
C9 1.06468484 -0.05793544 -0.12838978
C10 0.51840454 1.19879094 0.16634448
C11 1.64375503 2.21154128 0.15047850
O12 1.55176432 3.39882569 0.37769051
C13 2.51236890 0.15662306 -0.33183219
C14 2.87065706 1.44595977 -0.18360064
C15 4.27998985 1.90890285 -0.35563814
C16 5.16387279 0.65418489 -0.12655675
O17 3.31884807 -0.86726197 -0.67106304
H18 6.19706579 0.83691244 -0.43083475
H19 5.16086767 0.42611993 0.94793316
C20 5.41219083 -1.39168056 -1.57808385
C21 4.64962170 -0.56756223 -0.84632507
H22 6.46333562 -1.15316819 -1.69063724
C23 4.92974865 -2.61622713 -2.24849813
O24 5.95226759 -3.21977515 -2.88635106
O25 3.80168341 -3.05507618 -2.25504044
C26 5.60878642 -4.42432703 -3.57407902
H27 6.53391956 -4.78491670 -4.02257172
H28 5.20828082 -5.16176553 -2.87467870
H29 4.86125780 -4.22300497 -4.34483143
C30 5.07373649 3.73549968 -4.19231296
C31 4.36275822 2.53894931 -4.12369948
C32 4.09060558 1.95716947 -2.88877160
C33 4.53294190 2.55759641 -1.70653298
C34 5.24302774 3.75688602 -1.78511511
C35 5.51191746 4.34483484 -3.01911662
H36 5.27994305 4.19308814 -5.15549233

H37	4.01256882	2.06004345	-5.03360003
H38	3.51906482	1.03221391	-2.84665125
H39	5.58301470	4.23731381	-0.87029520
H40	6.05982856	5.28174002	-3.06311668
H41	4.52377855	2.64002086	0.42465913

***E*-3b**

E(M06-2X/6-31G(d)(THF)) = -1110.315684519
E(M06-2X/6-311+G(d,p)) = -1110.602592786
G(M06-2X/6-311+G(d,p)(THF)) = -1110.33560

C1	4.19308232	3.44565846	3.45831868
C2	5.37926208	4.19413824	3.35811967
C3	6.34044614	3.87875347	2.40479988
C4	6.16086490	2.80587762	1.51354906
H5	3.43321555	3.68153858	4.19805254
H6	5.54711057	5.02842842	4.03272480
H7	7.24879475	4.47141437	2.34611030
H8	6.91407930	2.56226040	0.77039749
C9	4.99414841	2.07861389	1.62457987
C10	4.02373029	2.39569512	2.58478475
C11	2.88250739	1.41451023	2.43594400
O12	1.85922482	1.40136962	3.08961973
C13	4.47173848	0.91050597	0.88163555
C14	3.27078240	0.50035389	1.33391292
C15	2.55170279	-0.66229918	0.71411517
C16	3.07279993	-0.74030480	-0.73698752
O17	5.17787471	0.35415537	-0.12090819
H18	2.71885060	-1.62342506	-1.26515285
H19	2.69653103	0.14200363	-1.27226435
C20	5.42275716	-1.44957654	-1.51587517
C21	4.57576824	-0.68179063	-0.81429033
H22	6.48309660	-1.22347530	-1.47647272
C23	5.01995353	-2.60113989	-2.34063542
O24	6.09322350	-3.13487035	-2.94494715
O25	3.90124901	-3.05751024	-2.47647002
C26	5.82945854	-4.28101260	-3.75993757
H27	6.79344948	-4.58157274	-4.16841659
H28	5.39933363	-5.08449668	-3.15795980
H29	5.13424640	-4.02466554	-4.56218385
C30	3.07941946	-4.21383420	3.14021668
C31	3.45693681	-4.23556788	1.80287540
C32	3.28750472	-3.10527332	1.00170760
C33	2.74168690	-1.93738573	1.53359485
C34	2.35291388	-1.92775134	2.87867193
C35	2.51986746	-3.05376948	3.67543761
H36	3.21589624	-5.09359948	3.76266148
H37	3.88694890	-5.13583748	1.37262167
H38	3.57961343	-3.15526915	-0.04269681
H39	1.91269836	-1.02621355	3.29829389
H40	2.21358090	-3.02644181	4.71747382
H41	1.47775163	-0.43979045	0.67364447

TS-prot-5

E(M06-2X/6-31G(d)(THF)) = -3115.161875100
E(M06-2X/6-311+G(d,p)) = -3115.891278228

H1	1.68509070	-5.28645638	-0.25635834
C2	2.37597057	-2.57770984	0.25125526
H3	1.36434146	-2.20431958	0.05855959
C4	3.22192845	-1.33513380	0.49980052
O5	4.37378312	-1.13111768	0.19257292
O6	2.50280280	-0.45758991	1.21995151
C7	3.22980355	0.67339599	1.70872503
H8	2.51160100	1.26403810	2.27618198
H9	4.04874407	0.33666774	2.34956753
H10	3.63483418	1.25446536	0.87784519
C11	2.72518456	-4.94401678	-0.19041841
H12	3.34074669	-5.71033039	-0.67410109
C13	3.08350282	-4.71443605	1.28008293
H14	4.14517187	-4.44088169	1.35368974
C15	2.32013151	-3.40534231	1.60817007
C16	2.91302403	-2.71267252	2.84208822
O17	4.08346798	-2.42603002	2.98657526
C18	0.86030405	-3.64393478	2.01148195
O19	0.01624197	-4.19849869	1.34278306
C20	1.82943502	-2.50714628	3.83777260
C21	0.64254628	-3.05638079	3.36029729
C22	-0.53078398	-3.01203122	4.10547142
C23	-0.47642002	-2.39278193	5.35089091
C24	0.72005665	-1.83966653	5.83379092
C25	1.89181094	-1.89215111	5.08404028
H26	-1.44764409	-3.44713125	3.71819992
H27	-1.37192655	-2.33463743	5.96344083
H28	0.72622351	-1.36405216	6.81069902
H29	2.82446531	-1.47158119	5.44923322
C30	2.35395733	-7.82143356	4.18851457
C31	3.40727376	-6.92502595	4.35259565
C32	3.63861871	-5.94231009	3.39503748
C33	2.82339297	-5.83429988	2.26334851
C34	1.76583070	-6.73609696	2.11029718
C35	1.53774151	-7.72428880	3.06466804
H36	2.17086198	-8.59160879	4.93279922
H37	4.05098485	-6.99200632	5.22550863
H38	4.46313061	-5.24310375	3.52342415
H39	1.10510263	-6.66929663	1.25032330
H40	0.71230388	-8.41659450	2.92418048
N41	4.08898065	-3.27434795	-1.47031694
C42	6.57186077	-2.14993179	-2.39941824
C43	6.48742576	-3.60564208	-1.96070363
C44	5.36244880	-3.79721496	-0.95356135
H45	7.23088231	-2.04414056	-3.26529701
H46	6.94596487	-1.50856834	-1.59278094
H47	6.32152081	-4.25315112	-2.82677112
H48	7.43517733	-3.89984089	-1.50159312
H49	5.57985222	-3.27296392	-0.01462191
H50	5.22879587	-4.86109477	-0.74995984
C51	4.11677187	-2.26529932	-2.31100571
N52	5.23962867	-1.68684527	-2.78352315
S53	2.66066290	-1.42575921	-2.80252028
C54	5.00275794	-0.60252393	-3.62928972
C55	3.63925685	-0.32529239	-3.76703956
C56	3.20059415	0.71986057	-4.56782773
C57	4.15375803	1.49795399	-5.22170725
C58	5.51502605	1.22728496	-5.07301242
C59	5.95820604	0.17531589	-4.27505707
H60	2.13981723	0.92616461	-4.67633217
H61	3.83184447	2.32328174	-5.84923353
H62	6.24548618	1.84618314	-5.58492003
H63	7.01896678	-0.02303317	-4.15840601
C64	2.79214584	-3.57804654	-0.86376230
H65	2.03004776	-3.94202186	-2.09195406

C66	2.42578237	-5.76254819	-3.27627214
C67	3.72068877	-5.96165100	-3.69829824
H68	4.18400514	-6.92473859	-3.52358879
C69	4.46134780	-4.97825320	-4.43669400
O70	4.15074753	-3.82127480	-4.69884985
O71	5.67356383	-5.47420136	-4.84231289
C72	6.44603547	-4.58159939	-5.63316822
H73	7.38405316	-5.09694407	-5.84172646
H74	5.93005476	-4.34074196	-6.56653129
H75	6.63661420	-3.64592089	-5.09719180
C76	1.65118833	-4.56668857	-3.28858989
H77	2.03590528	-3.85881448	-4.01773591
C78	0.11653983	-4.67784111	-3.30247524
H79	-0.23875740	-4.90219812	-4.32045738
C80	-0.22905592	-5.81914782	-2.39829075
C81	-1.47485857	-6.16111325	-1.66686001
O82	-2.54013355	-5.58140109	-1.67821344
C83	0.66923157	-6.79168218	-2.13596808
O84	1.91876830	-6.88397279	-2.60997244
C85	-1.16423206	-7.42540091	-0.88951441
C86	0.14546247	-7.81144976	-1.19984849
C87	0.72281998	-8.92818170	-0.63101616
C88	-0.05690995	-9.66751013	0.27570736
C89	-1.35685906	-9.28370049	0.58750098
C90	-1.93120284	-8.14257920	-0.00044367
H91	1.74185563	-9.22095020	-0.86625423
H92	0.36632408	-10.55236275	0.74317584
H93	-1.93458779	-9.87301008	1.29393208
H94	-2.94299448	-7.82876441	0.23917392
C95	-1.36978035	-0.77552193	-2.20889762
C96	-1.24401877	-1.13244381	-3.54919145
C97	-0.80382922	-2.40985566	-3.89314453
C98	-0.48000812	-3.34094646	-2.90706886
C99	-0.61210263	-2.97744207	-1.56697656
C100	-1.05657566	-1.70583242	-1.21842644
H101	-1.71796010	0.21765434	-1.93763416
H102	-1.49540387	-0.41868846	-4.32964917
H103	-0.70155440	-2.68600979	-4.94121324
H104	-0.36591517	-3.69615535	-0.78891536
H105	-1.16341886	-1.44433291	-0.16793434

Int3-5
E(M06-2X/6-31G(d)(THF)) = -2005.355357948
E(M06-2X/6-311+G(d,p)) = -2005.760333452
G(M06-2X/6-311+G(d,p)(THF)) = -2005.33228

H1	2.58770899	-5.81465332	-0.04391032
C2	2.19048200	-3.03991033	0.29108951
H3	1.16522351	-3.27384526	-0.02376696
C4	2.27772037	-1.51961441	0.28998744
O5	3.05492925	-0.82584872	-0.32440674
O6	1.33443779	-1.03396068	1.09831483
C7	1.36059828	0.38857992	1.30566757
H8	0.52962797	0.60270004	1.97553016
H9	2.31119120	0.67382664	1.76163768
H10	1.23599599	0.90911969	0.35470861
C11	3.42492701	-5.13231901	0.13572621
H12	4.33776537	-5.63863420	-0.19373680
C13	3.45757502	-4.72700435	1.61194706
H14	4.39346758	-4.19560669	1.81273524
C15	2.34062691	-3.62694883	1.72501451
C16	2.74650340	-2.65566324	2.83953798
O17	3.68841363	-1.89271481	2.77630293
C18	1.01956987	-4.21499136	2.23675786
O19	0.25220733	-4.87011879	1.56833411
C20	1.84654291	-2.87958488	3.99457498
C21	0.85705528	-3.80459922	3.65382064
C22	-0.11013793	-4.20014389	4.57087316
C23	-0.05159008	-3.64242145	5.84590603
C24	0.94146752	-2.71164888	6.18804355
C25	1.90551114	-2.31519060	5.26481513
H26	-0.87674076	-4.91829165	4.29533918
H27	-0.78689432	-3.92937483	6.59219178
H28	0.95390422	-2.29627393	7.19168117
H29	2.67922548	-1.59620361	5.51831735
C30	3.17131729	-7.78723124	4.63936170
C31	3.99678940	-6.68016237	4.81947423
C32	4.08900048	-5.71475364	3.82179681
C33	3.35922943	-5.83706361	2.63541234
C34	2.53112471	-6.95037190	2.46493866
C35	2.44075147	-7.91932318	3.46112525
H36	3.09882800	-8.54531413	5.41384588
H37	4.57170599	-6.56947483	5.73440621
H38	4.73717083	-4.85114041	3.96424869
H39	1.93851546	-7.06505099	1.56148653
H40	1.79506123	-8.78040567	3.31456499
N41	4.33665579	-3.12713255	-1.10082019
C42	6.20577398	-1.01336558	-1.59614120
C43	6.62847384	-2.24908000	-0.82005733
C44	5.42929708	-2.89042965	-0.13893979
H45	7.02937274	-0.63872227	-2.20680523
H46	5.84679707	-0.21370361	-0.93948846
H47	7.10144507	-2.96464164	-1.50065515
H48	7.36234564	-1.97365258	-0.05899881
H49	5.04651605	-2.26362887	0.67720119
H50	5.72634011	-3.86086812	0.26320271
C51	4.24223685	-2.35776248	-2.18099912
N52	5.11231436	-1.39386675	-2.49912046
S53	2.95588917	-2.50534891	-3.35428154
C54	4.80106359	-0.69918327	-3.67825324
C55	3.64599019	-1.18734316	-4.28820552
C56	3.15733574	-0.63966748	-5.46747291
C57	3.85951639	0.42475140	-6.02400423
C58	5.01197669	0.92313257	-5.40874162
C59	5.49971184	0.37117970	-4.22865933
H60	2.25746981	-1.02759935	-5.93571040
H61	3.50220339	0.87608051	-6.94408511
H62	5.53822378	1.76053466	-5.85565718
H63	6.38859036	0.77449205	-3.75483509
C64	3.13167177	-3.85002163	-0.65359830
H65	2.59090072	-4.12290148	-1.56210148

TS-deprot-5
E(M06-2X/6-31G(d)(THF)) = -3115.169908030
E(M06-2X/6-311+G(d,p)) = -3115.89482234

C1	2.04894778	-2.95019037	0.47238555
H2	0.87916130	-3.34345749	-0.13738296
C3	1.85081056	-1.50334259	0.39485200
O4	2.23326812	-0.75910227	-0.49702781
O5	1.10972836	-1.06345061	1.43122449
C6	0.73427951	0.30974823	1.38657631

H7	-0.01851234	0.43635569	2.16382407
H8	1.60182232	0.94679237	1.58251715
H9	0.31637355	0.55228317	0.40864700
C10	3.19974887	-4.78076306	1.66473951
H11	4.13842805	-4.42839392	2.11050363
C12	2.18419551	-3.58513266	1.86633394
C13	2.75066051	-2.62754097	2.93926556
O14	3.83317289	-2.07807299	2.89169776
C15	0.82708799	-3.99529715	2.48191792
O16	0.00546545	-4.73248553	1.99150060
C17	1.79351760	-2.53916733	4.06943262
C18	0.68284978	-3.33197335	3.80609805
C19	-0.36725222	-3.43392774	4.71142019
C20	-0.26594790	-2.70909376	5.89529350
C21	0.85556921	-1.90831102	6.16222260
C22	1.90361197	-1.81370981	5.25145164
H23	-1.22886893	-4.05661215	4.48764375
H24	-1.06670156	-2.75888306	6.62807580
H25	0.90041419	-1.35448019	7.09597152
H26	2.77715883	-1.19746975	5.44455084
C27	2.05993827	-8.27294586	3.94988684
C28	2.64264448	-7.16558877	4.56201712
C29	3.01959816	-6.06904817	3.79451049
C30	2.81904679	-6.04826905	2.40944934
C31	2.23890632	-7.16601547	1.80545715
C32	1.86439141	-8.26857490	2.57259664
H33	1.76318537	-9.13346775	4.54291854
H34	2.80579930	-7.15624716	5.63619882
H35	3.48072792	-5.20797171	4.27755809
H36	2.06226717	-7.20678550	0.73390839
H37	1.41404952	-9.12715698	2.08175587
N38	4.17384137	-2.74715199	-0.90045177
C39	6.14066284	-0.73969683	-1.52730136
C40	6.41994712	-1.85187679	-0.52557853
C41	5.12076242	-2.29871566	0.12765907
H42	7.00894037	-0.56577649	-2.16845413
H43	5.87389101	0.19898757	-1.02908448
H44	6.89462603	-2.69952191	-1.03142295
H45	7.10864095	-1.48609002	0.23976143
H46	4.65735120	-1.49005400	0.70293961
H47	5.29221248	-3.13346597	0.80891592
C48	4.12767820	-2.08509306	-2.03920794
N49	5.02355506	-1.14284728	-2.39063149
S50	2.91477608	-2.34730350	-3.27129210
C51	4.78213733	-0.56323345	-3.64189748
C52	3.66905743	-1.11853085	-4.27485781
C53	3.26594866	-0.68326728	-5.53017301
C54	4.00574030	0.32519619	-6.14198748
C55	5.11639436	0.88428132	-5.50527855
C56	5.51930123	0.45063139	-4.24594128
H57	2.39459715	-1.11134226	-6.01652504
H58	3.70905587	0.68394593	-7.12225836
H59	5.67529344	1.67525934	-5.99557382
H60	6.37429570	0.89965616	-3.75089541
C61	3.01891953	-3.58738014	-0.49916425
H62	2.49936708	-3.81323723	-1.44011589
C63	-0.45224957	-3.15263628	-2.07824468
C64	-0.98967711	-1.90447777	-2.29822566
H65	-0.98812555	-1.50686618	-3.30462177
C66	-1.54070984	-1.09481945	-1.25032882
O67	-1.58170980	-1.32780609	-0.04798617
O68	-2.06295897	0.07025157	-1.75196636
C69	-2.68592552	0.90640564	-0.78837768
H70	-3.05559667	1.77525724	-1.33507853
H71	-3.51556782	0.38930472	-0.29775731
H72	-1.97546153	1.22204610	-0.01874244
C73	-0.28440243	-3.86542264	-0.86022174
H74	-0.98553771	-3.53129260	-0.09868741
C75	-0.02303199	-5.37046937	-0.86547227
C76	0.67563234	-5.70483865	-2.13837674
C77	1.48355120	-6.88431055	-2.49526605
O78	1.84012743	-7.79409737	-1.76933825
C79	0.61940299	-4.89964903	-3.22009318
O80	0.03889250	-3.70137893	-3.28654062
C81	1.85494732	-6.70996599	-3.95422005
C82	1.32328029	-5.48675022	-4.38697567
C83	1.49938659	-5.04810515	-5.68229604
C84	2.23172516	-5.87376873	-6.55428645
C85	2.76136651	-7.08441234	-6.12465411
C86	2.57730610	-7.51925574	-4.79954925
H87	1.07726687	-4.10615281	-6.02140194
H88	2.38246218	-5.56040176	-7.58350974
H89	3.31989730	-7.70264402	-6.82131985
H90	2.98451705	-8.46384149	-4.44930498
C91	-3.62023107	-7.72876993	-0.42267433
C92	-2.59377089	-7.83875199	0.51004888
C93	-1.43305169	-7.07942912	0.36862236
C94	-1.28710686	-6.20452981	-0.70631111
C95	-2.32408110	-6.09644692	-1.63663991
C96	-3.48263947	-6.85196142	-1.49902829
H97	-4.52649111	-8.31794959	-0.31121137
H98	-2.69772378	-8.51375654	1.35541789
H99	-0.63723364	-7.14778513	1.10653802
H100	-2.22169201	-5.40491594	-2.47171513
H101	-4.28228455	-6.75597912	-2.22870498
H102	0.62000977	-5.61499418	-0.01276831
C103	3.42583875	-4.91762301	0.14898447
H104	4.45981784	-5.19176831	-0.08674620
H105	2.79314327	-5.69735375	-0.27931937

Int4-5

E(M06-2X/6-31G(d)(THF)) = -2004.870810949
E(M06-2X/6-311+G(d,p)) = -2005.309219780
G(M06-2X/6-311+G(d,p)(THF)) = -2004.87478

H1	2.54084659	-5.78575517	-0.06256694
C2	2.53905308	-2.95891657	0.51337194
C3	2.17716099	-1.63692505	0.27052404
O4	2.32543620	-0.99976041	-0.79120306
O5	1.62753889	-1.01927662	1.38033125
C6	1.35149322	0.35992751	1.22476364
H7	0.92166851	0.69122204	2.17172419
H8	2.26368993	0.92582755	1.01102362
H9	0.64235857	0.53252308	0.41036687
C10	3.40699518	-5.14637181	0.12935890
H11	4.29343448	-5.65583499	-0.26477271
C12	3.51889136	-4.83658310	1.63048485
H13	4.50330452	-4.39220256	1.81705117
C14	2.52490066	-3.64898799	1.84806566
C15	3.00007598	-2.79510965	3.03115598
O16	4.09199312	-2.27346875	3.13468095
C17	1.12543578	-4.10987181	2.28305378

O18	0.36967934	-4.80663419	1.63907664
C19	1.93090976	-2.78406005	4.06631132
C20	0.84880706	-3.54986960	3.63814656
C21	-0.27353349	-3.72258888	4.44126370
C22	-0.28012853	-3.10318580	5.68771305
C23	0.81011906	-2.33220652	6.11802874
C24	1.93199741	-2.16475638	5.31171304
H25	-1.10940800	-4.32435092	4.09645856
H26	-1.13972971	-3.21650416	6.34246768
H27	0.77370742	-1.86255755	7.09713235
H28	2.78493703	-1.57350061	5.63281369
C29	3.13264867	-8.05688928	4.48429802
C30	4.02150642	-7.00788263	4.70436380
C31	4.13689917	-5.98754328	3.76510696
C32	3.37051097	-5.99221164	2.59440262
C33	2.47349702	-7.04487879	2.39039585
C34	2.35923503	-8.06990428	3.32628678
H35	3.04208788	-8.85892091	5.21155620
H36	4.62813990	-6.98513450	5.60561962
H37	4.83298417	-5.16819057	3.93775121
H38	1.84568828	-7.06121251	1.50459917
H39	1.65871552	-8.88148104	3.14971606
N40	4.40284406	-3.12988793	-1.11037388
C41	6.01006047	-0.81793129	-1.47849975
C42	6.59862467	-2.02286228	-0.75972061
C43	5.48383332	-2.85754577	-0.14620380
H44	6.78570160	-0.25547787	-2.00098081
H45	5.47738317	-0.15033920	-0.79105908
H46	7.17995299	-2.61649545	-1.47357777
H47	7.27519588	-1.69282614	0.03264949
H48	5.04411437	-2.36156488	0.72588172
H49	5.88020031	-3.82687623	0.17256700
C50	4.26643758	-2.36977785	-2.17442723
N51	5.05968208	-1.31892760	-2.47154916
S52	3.09524581	-2.67986237	-3.44406302
C53	4.72078338	-0.65139502	-3.65137042
C54	3.66525476	-1.27133001	-4.32406917
C55	3.17958651	-0.76769619	-5.52304214
C56	3.77750096	0.37712050	-6.04382809
C57	4.82798809	1.00197172	-5.36836231
C58	5.31275621	0.49900884	-4.16426856
H59	2.35410210	-1.25275327	-6.03567201
H60	3.41573360	0.78999623	-6.98011830
H61	5.27426591	1.90075573	-5.78225915
H62	6.11841920	1.00244476	-3.64012707
C63	3.13078582	-3.79420633	-0.54160462
H64	2.50892911	-3.93184752	-1.43461759

TS-elim-5
E(M06-2X/6-31G(d)(THF)) = -2004.866379670
E(M06-2X/6-311+G(d,p)) = -2005.306383574
G(M06-2X/6-311+G(d,p)(THF)) = -2004.87088

H1	2.28536691	-5.95113107	-0.01820300
C2	2.40671218	-3.11746541	0.46317225
C3	2.00387275	-1.79708224	0.15395161
O4	2.14392835	-1.22573761	-0.93007495
O5	1.44824478	-1.15507739	1.23029517
C6	1.08453727	0.19841371	1.00435379
H7	0.66396923	0.55847036	1.94399386
H8	1.95746813	0.79637799	0.72748999
H9	0.34346221	0.27498270	0.20442510
C10	3.17157896	-5.33006556	0.15053392
H11	4.03997435	-5.86497474	-0.24695788
C12	3.32628369	-4.98039305	1.64156816
H13	4.33834306	-4.58577225	1.78943197
C14	2.39820984	-3.73152476	1.83563334
C15	2.92702273	-2.85980238	2.98092838
O16	4.02600902	-2.34584127	3.03226190
C17	0.98606364	-4.11456681	2.30878799
O18	0.17984611	-4.76694152	1.68228847
C19	1.89711369	-2.80922111	4.05273048
C20	0.77787955	-3.54477959	3.67046482
C21	-0.32080011	-3.67559416	4.51330167
C22	-0.26371406	-3.04560861	5.75330252
C23	0.86446194	-2.30470292	6.13776863
C24	1.96162231	-2.17835162	5.29078073
H25	-1.18707971	-4.25327197	4.20409757
H26	-1.10284077	-3.12653406	6.43886491
H27	0.87701832	-1.82598726	7.11309479
H28	2.84295645	-1.61066689	5.57578638
C29	2.79577244	-8.05790023	4.62408310
C30	3.74884425	-7.05634670	4.79158890
C31	3.91156956	-6.08269776	3.81074747
C32	3.12938330	-6.08803876	2.65088945
C33	2.16954274	-7.09280390	2.49832677
C34	2.00736224	-8.07114451	3.47610512
H35	2.66787890	-8.82385360	5.38396425
H36	4.36887285	-7.03496386	5.68371929
H37	4.65872837	-5.30137117	3.94134030
H38	1.53158815	-7.10748037	1.61942230
H39	1.25857459	-8.84657020	3.33965502
N40	4.48869314	-3.35042834	-1.12457421
C41	5.85446512	-0.83271526	-1.24395835
C42	6.51121822	-2.01233613	-0.53900095
C43	5.44681538	-2.99821447	-0.06781123
H44	6.60464835	-0.17210699	-1.68472451
H45	5.22156334	-0.25175188	-0.56200898
H46	7.20763922	-2.49803505	-1.23137811
H47	7.08531453	-1.65824925	0.32120481
H48	4.89092766	-2.58059988	0.77971343
H49	5.92346875	-3.92725894	0.26395464
C50	4.37426018	-2.54881503	-2.14348772
N51	5.02533686	-1.36741127	-2.32011531
S52	3.35794807	-2.91878923	-3.53496030
C53	4.63614615	-0.64948450	-3.44784374
C54	3.71987403	-1.35053931	-4.23822616
C55	3.21419254	-0.80503079	-5.40933964
C56	3.64333193	0.46676665	-5.78365059
C57	4.55143466	1.17120224	-4.99185197
C58	5.05800750	0.62496812	-3.81553236
H59	2.49918636	-1.35550188	-6.01360758
H60	3.25949149	0.91397220	-6.69506619
H61	4.86616339	2.16649417	-5.29002910
H62	5.74981827	1.18795304	-3.19763394
C63	2.92731332	-3.98835725	-0.51966083
H64	2.45235407	-4.02023828	-1.49999750

5b
E(M06-2X/6-31G(d)(THF)) = -1110.335946926
E(M06-2X/6-311+G(d,p)) = -1110.62462908
G(M06-2X/6-311+G(d,p)(THF)) = -1110.36194

H1	4.59101493	-5.31491140	1.12996377
C2	4.20108480	-3.41448262	0.24078798
C3	3.17435410	-2.75091111	0.78040343
C4	4.90339978	-4.27098552	1.25835160
H5	5.99448498	-4.24666390	1.17553113
C6	4.41324144	-3.67082462	2.59171958
H7	5.02510103	-2.77922291	2.78115606
C8	3.00000417	-3.08464263	2.24254964
C9	2.65186444	-1.93430690	3.19305958
O10	3.28025890	-0.90596272	3.30488878
C11	1.86314743	-4.09521660	2.46922383
O12	1.69487501	-5.11205703	1.83485588
C13	1.47238760	-2.34417626	4.00204345
C14	1.02594487	-3.59991327	3.59376137
C15	-0.05425256	-4.22064853	4.21239955
C16	-0.67473046	-3.54269815	5.25785384
C17	-0.22572800	-2.27828003	5.66880307
C18	0.85555638	-1.66153249	5.04540060
H19	-0.39059634	-5.20035174	3.88570193
H20	-1.51978086	-3.99600702	5.76836165
H21	-0.73254111	-1.77754672	6.48894396
H22	1.21474531	-0.68474367	5.35677343
C23	4.41690544	-6.03231311	6.20325156
C24	4.72948064	-4.67571673	6.23722995
C25	4.74354207	-3.93623306	5.05813455
C26	4.44384018	-4.53504853	3.82990329
C27	4.12553150	-5.89648999	3.80820160
C28	4.11552307	-6.63905724	4.98641091
H29	4.40939794	-6.61463790	7.12035924
H30	4.96638094	-4.19285675	7.18121849
H31	4.99069722	-2.87629298	5.08563263
H32	3.87171679	-6.38320122	2.87053702
H33	3.86981907	-7.69675123	4.95221320
H34	4.46587173	-3.37069070	-0.81130843
C35	2.23661848	-1.89764950	0.01944481
O36	1.17769340	-1.56446726	0.77664292
O37	2.38012888	-1.54506951	-1.12882487
C38	0.19889914	-0.73951046	0.13510399
H39	-0.57472857	-0.56327048	0.88102420
H40	0.65122805	0.20221189	-0.18304843
H41	-0.21416962	-1.25435106	-0.73499290

Int2-7

E(M06-2X/6-31G(d)(THF)) = -2004.827924341

E(M06-2X/6-311+G(d,p)) = -2005.266239697

G(M06-2X/6-311+G(d,p)(THF)) = -2004.82659

C1	-6.27732483	-7.54687548	-4.53912190
C2	-6.68970297	-8.78996301	-5.01807451
C3	-5.75681796	-9.72094865	-5.47873735
C4	-4.39197414	-9.43953817	-5.46571814
C5	-3.98800578	-8.20193033	-4.97544316
C6	-4.91900215	-7.25851330	-4.52456912
H7	-7.00148655	-6.81856407	-4.18436676
H8	-7.74769167	-9.03347558	-5.03485564
H9	-6.09569993	-10.68122054	-5.85566582
H10	-3.66991651	-10.16261605	-5.83254188
C11	-2.58593935	-6.47789483	-4.38822721
C12	-0.22993260	-6.38915850	-4.73437068
C13	-0.26174333	-7.90321510	-4.55124856
C14	-1.48116625	-8.50898062	-5.24040294
H15	-0.13446539	-6.09459746	-5.78482368
H16	0.59203105	-5.94752261	-4.17274571
H17	0.64545636	-8.35180414	-4.96471985
H18	-0.29338807	-8.13200284	-3.48087760
H19	-1.63415094	-9.54368058	-4.91993654
H20	-1.38379371	-8.49121198	-6.33194073
N21	-2.67077694	-7.74012159	-4.86032529
S22	-4.12448799	-5.75937475	-3.98960836
N23	-1.48181978	-5.80022590	-4.21098046
C24	-1.63466497	-4.48931355	-3.60147458
C25	-1.63539539	-3.39599769	-5.77943431
C26	-1.07255061	-3.39435207	-4.37395016
O27	-1.33629478	-4.17830630	-6.65506136
H28	-1.30855633	-2.44506262	-3.88251512
O29	-2.58003409	-2.45613370	-5.92651709
C30	-3.27819875	-2.48369557	-7.17305566
H31	-3.99932519	-1.66787818	-7.12921202
H32	-2.58369435	-2.33761406	-8.00374723
H33	-3.79029253	-3.44120714	-7.29732810
C34	-0.00814138	-4.70171883	-1.55205584
H35	-0.00210620	-4.34637538	-0.50961710
C36	0.96493429	-8.92696358	-1.09446824
C37	1.85851652	-8.07677634	-1.73933672
C38	1.55112092	-6.72692534	-1.90694883
C39	0.34675242	-6.19084939	-1.44032972
C40	-0.53690244	-7.05926074	-0.78403035
C41	-0.23737917	-8.40851348	-0.61533213
H42	1.20385815	-9.97854560	-0.95950104
H43	2.80466509	-8.46049451	-2.11274578
H44	2.26477474	-6.08470477	-2.41546522
H45	-1.46732278	-6.67365458	-0.37460528
H46	-0.94215752	-9.05461659	-0.09818250
C47	1.05504388	-3.90684280	-2.26030691
C48	3.24461656	-2.91447897	-2.47947173
C49	2.55809735	-2.79083320	-3.68593721
C50	3.13055339	-2.16951777	-4.77708121
C51	4.43693791	-1.67495699	-4.62727690
C52	5.12306614	-1.79989598	-3.42245605
C53	4.52340239	-2.42672181	-2.31794335
H54	2.58906932	-2.06755610	-5.71262907
H55	4.92014148	-1.18320570	-5.46685843
H56	6.13119417	-1.40474343	-3.33611173
H57	5.04113954	-2.52534932	-1.36778634
C58	1.20080445	-3.39872922	-3.52363337
O59	0.42271733	-3.33362316	-4.59448065
C60	2.31615734	-3.61588307	-1.52602990
O61	2.55933374	-3.89676087	-0.36713494
C62	-1.43383220	-4.44436717	-2.10880898
H63	-1.72703943	-3.43921168	-1.77858393
H64	-2.13212008	-5.13012878	-1.61128016

TS-deprot-K2CO3

E(M06-2X/6-31G(d)(THF)) = -3468.44221151

E(M06-2X/6-311+G(d,p)) = -3468.942628037

G(M06-2X/6-311+G(d,p)(THF)) = -3468.53344

C1	4.58543634	-5.44644095	-7.96384406
C2	5.52170067	-4.93053804	-8.85905955
C3	5.55291463	-3.56474472	-9.14411322
C4	4.66161177	-2.68084424	-8.53992483

C5 3.73989182 -3.19800640 -7.63405744
C6 3.69638411 -4.56953775 -7.35865841
H7 4.55306177 -6.50946090 -7.74135060
H8 6.22940162 -5.59921590 -9.33871201
H9 6.28234306 -3.17901664 -9.84972036
H10 4.68600238 -1.62130466 -8.77461528
C11 1.97628119 -3.25935400 -6.14870557
C12 0.61228302 -1.42002596 -5.47391611
C13 1.80893142 -0.54467634 -5.83204195
C14 2.54515301 -1.04777797 -7.06500766
H15 -0.20821953 -1.30797436 -6.19343768
H16 0.23658722 -1.14650644 -4.48742465
H17 1.47172318 0.48098493 -6.00551506
H18 2.50400854 -0.53285519 -4.99018059
H19 3.51264429 -0.54627451 -7.15855279
H20 1.97417633 -0.87908198 -7.98648022
N21 2.78877384 -2.47852969 -6.91113199
S22 2.41517970 -4.95020356 -6.19611143
N23 0.98750307 -2.84999883 -5.40563556
C24 0.26223217 -3.84009762 -4.58732577
C25 -1.86144723 -3.53113245 -5.81970505
C26 -1.17246725 -3.60183541 -4.52487278
O27 -1.71711266 -4.35856088 -6.72110039
H28 -1.90686519 -4.67364468 -3.88677919
O29 -2.75036339 -2.52423911 -5.96283077
C30 -3.47875066 -2.52598484 -7.18650880
H31 -4.13915729 -1.65958677 -7.14032877
H32 -2.80465852 -2.43958076 -8.04290502
H33 -4.06279830 -3.44367397 -7.29211043
C34 1.22950554 -3.08575404 -2.27484317
H35 1.61321921 -3.56177015 -1.35793721
C36 4.49127390 -0.35118951 -3.27256541
C37 3.33466160 0.12710413 -2.66365175
C38 2.27501185 -0.73604414 -2.38138375
C39 2.34144166 -2.10207277 -2.67996567
C40 3.51691253 -2.56526351 -3.28973461
C41 4.57123160 -1.70608218 -3.59145303
H42 5.31819818 0.31822650 -3.49316929
H43 3.24957333 1.17953537 -2.40535385
H44 1.38318685 -0.32943140 -1.91542034
H45 3.62387484 -3.62000181 -3.52898178
H46 5.46480698 -2.10325083 -4.06657579
C47 -0.04144547 -2.39419089 -1.87269600
C48 -1.47609921 -1.09915288 -0.44726960
C49 -2.12082446 -1.31719253 -1.66357529
C50 -3.37460918 -0.79296823 -1.91556190
C51 -3.97282272 -0.02380197 -0.90058927
C52 -3.32548112 0.19416266 0.31300388
C53 -2.05284658 -0.35127992 0.55624593
H54 -3.86123364 -0.94971215 -2.87466848
H55 -4.95012943 0.42036948 -1.07059732
H56 -3.80633731 0.80025116 1.07581624
H57 -1.53366766 -0.18574339 1.49663330
C58 -1.22254071 -2.14513347 -2.53077104
O59 -1.71544918 -2.44207513 -3.70379210
C60 -0.13929836 -1.78816499 -0.53415517
O61 0.70119992 -1.80687886 0.35040092
C62 0.97038582 -4.21119367 -3.30140519
H63 0.33675076 -4.96180034 -2.81135439
H64 1.92536093 -4.72427251 -3.48755697
K65 -0.86287965 -6.55109081 -5.36927549
O66 -2.74727961 -5.43563537 -3.42517808
C67 -2.30632406 -6.42357401 -2.58048573
O68 -1.23569905 -7.02188401 -2.87429001
O69 -3.04509900 -6.66404157 -1.58480104
K70 -4.62158474 -4.65436600 -1.76683263

Int3-7

E(M06-2X/6-31G(d)(THF)) = -2604.142530344
E(M06-2X/6-311+G(d,p)) = -2604.56778780
G(M06-2X/6-311+G(d,p)(THF)) = -2604.16082

C1 -6.13242574 -7.98804144 -3.87532302
C2 -6.46459986 -9.23162033 -4.40332475
C3 -5.52996347 -9.97658828 -5.12963789
C4 -4.23834415 -9.50702172 -5.34399521
C5 -3.90335925 -8.26791035 -4.80441498
C6 -4.84058782 -7.52196552 -4.08931578
H7 -6.85567533 -7.40298416 -3.31510585
H8 -7.46485255 -9.62453878 -4.25122004
H9 -5.81536590 -10.94012755 -5.53984594
H10 -3.52578012 -10.08728023 -5.92131922
C11 -2.63047409 -6.40708682 -4.31787817
C12 -0.29066774 -6.09463012 -4.90050746
C13 -0.24873385 -7.61335637 -4.91005511
C14 -1.48837937 -8.19071065 -5.57171490
H15 -0.17667846 -5.66852959 -5.90174374
H16 0.51385414 -5.71746435 -4.27114710
H17 0.64346443 -7.93782315 -5.45119717
H18 -0.18003894 -7.98618233 -3.88358840
H19 -1.53108336 -9.27380038 -5.43848856
H20 -1.54194139 -7.96170653 -6.64152598
N21 -2.66453434 -7.61401755 -4.90218083
S22 -4.14753066 -5.99718084 -3.55089292
N23 -1.57095364 -5.60785395 -4.32152844
C24 -1.41043879 -3.16517457 -5.97679948
C25 -0.89519054 -3.20060295 -4.53371013
O26 -0.79387789 -3.54422161 -6.93784567
H27 -1.12266744 -2.23388891 -4.06381343
O28 -2.65277092 -2.68782712 -6.00205266
C29 -3.26321473 -2.60340487 -7.30401453
H30 -4.26167682 -2.20488103 -7.13363516
H31 -2.68289068 -1.93521694 -7.94304348
H32 -3.31135977 -3.59423135 -7.75959752
C33 -0.09794417 -4.63776193 -1.66255591
H34 -0.09249713 -4.34042551 -0.60342141
C35 0.47821031 -8.94899784 -1.40424532
C36 1.52756792 -8.12625146 -1.80250877
C37 1.33750940 -6.75001980 -1.92216551
C38 0.10052407 -6.16296017 -1.64017929
C39 -0.94526330 -7.00269525 -1.23446290
C40 -0.76269285 -8.37936896 -1.12372026
H41 0.62604299 -10.02063212 -1.30551648
H42 2.50370850 -8.55130264 -2.01954022
H43 2.17462867 -6.13480216 -2.23752837
H44 -1.91404450 -6.58805277 -0.96693534
H45 -1.58917599 -9.00544329 -0.79837660
C46 1.04590247 -3.89568094 -2.28790357
C47 3.27564130 -2.97911053 -2.41926177
C48 2.63585692 -2.82733092 -3.65159861
C49 3.27077876 -2.23833138 -4.72630259
C50 4.59253607 -1.80294736 -4.53171816

C51	5.23078094	-1.95341878	-3.30393987
C52	4.56947438	-2.54846268	-2.21708867
H53	2.77236843	-2.11876906	-5.68361536
H54	5.12588356	-1.33910561	-5.35651031
H55	6.25252947	-1.60534836	-3.18592188
H56	5.05499209	-2.66817127	-1.25256006
C57	1.26491738	-3.38739583	-3.52238576
O58	0.50341910	-3.35351229	-4.63513586
C59	2.29836341	-3.64365068	-1.49569624
O60	2.46300242	-3.94050376	-0.33330963
C61	-1.45658598	-4.18697236	-2.21233611
H62	-1.61374459	-3.13932996	-1.92955311
H63	-2.25875359	-4.74643658	-1.72270185
C64	-1.68256281	-4.25426455	-3.72911613
H65	-2.71933547	-3.93560981	-3.89654542

TS-prot-KHCO3

E(M06-2X/6-31G(d)(THF)) = -3468.43352844
E(M06-2X/6-311+G(d,p)) = -3468.927045090

C1	3.34477107	-3.19563266	-9.80760918
C2	3.49961318	-2.25186475	-10.82298181
C3	2.95870669	-0.97224209	-10.68987537
C4	2.25588588	-0.60321620	-9.54453730
C5	2.11494223	-1.54663119	-8.53026345
C6	2.65067406	-2.83026684	-8.66224528
H7	3.75265087	-4.19696787	-9.91537461
H8	4.04027928	-2.51951381	-11.72527980
H9	3.08042492	-0.24967868	-11.49077602
H10	1.82500179	0.38861932	-9.45380531
C11	1.44659056	-2.47227092	-6.50980587
C12	0.02929123	-1.39131638	-4.97021934
C13	0.66800067	-0.08717253	-5.41869912
C14	0.85816595	-0.09152529	-6.92496881
H15	-0.96594980	-1.53056129	-5.41120498
H16	-0.07433639	-1.40501935	-3.88808452
H17	0.03610352	0.75666100	-5.12896670
H18	1.63709568	0.02997273	-4.92499471
H19	1.53517559	0.70919335	-7.23688697
H20	-0.09166618	0.02566626	-7.45974241
N21	1.46431490	-1.36727610	-7.31125153
S22	2.30044384	-3.81947779	-7.24294645
N23	0.87046341	-2.55035319	-5.34828120
C24	0.73018503	-3.87363853	-4.58809996
C25	-1.51022377	-4.40521627	-5.53318264
C26	-0.71956361	-4.10453823	-4.40595700
O27	-1.11421887	-4.97578534	-6.57696036
H28	1.49359584	-5.22983410	-5.16819450
O29	-2.84489876	-4.06566607	-5.42262898
C30	-3.64249055	-4.40568835	-6.54108962
H31	-4.64953179	-4.05327510	-6.31037797
H32	-3.27974688	-3.92455860	-7.45393293
H33	-3.66119004	-5.48717674	-6.71156926
C34	1.52265144	-2.80468800	-2.24979679
H35	2.13231735	-3.16951676	-1.40748830
C36	3.47575965	1.00173872	-3.13998218
C37	2.25774835	0.97958880	-2.46680766
C38	1.61661444	-0.23066526	-2.20833604
C39	2.16860164	-1.45437004	-2.60747093
C40	3.39564859	-1.41186249	-3.28111469
C41	4.03996870	-0.20505182	-3.54796668
H42	3.98092383	1.94299354	-3.33831452
H43	1.80001115	1.90785486	-2.13447445
H44	0.66525846	-0.21421746	-1.68741089
H45	3.87218783	-2.33654087	-3.59256734
H46	4.99444902	-0.21066570	-4.06819184
C47	0.12818542	-2.66805271	-1.71542897
C48	-1.60084156	-2.05949902	-0.16646726
C49	-2.19195158	-2.54036919	-1.33424215
C50	-3.56433669	-2.56688808	-1.48933008
C51	-4.34544436	-2.08281621	-0.42683280
C52	-3.75599276	-1.60088009	0.73972118
C53	-2.35917432	-1.58678038	0.88461172
H54	-4.01508079	-2.94139902	-2.40387905
H55	-5.42834837	-2.08078272	-0.51710113
H56	-4.38535978	-1.23044129	1.54418282
H57	-1.88510880	-1.21323370	1.78826921
C58	-1.10153694	-2.94496495	-2.27159999
O59	-1.51267548	-3.44856966	-3.40011622
C60	-0.10855062	-2.15189497	-0.36029196
O61	0.73310344	-1.82942035	0.46682186
C62	1.62186570	-3.88810775	-3.34821107
H63	1.41965377	-4.85150274	-2.86446412
H64	2.67158469	-3.95474816	-3.66768082
K65	4.54940021	-6.47192936	-6.41504291
O66	2.09143543	-6.15903732	-5.38866128
C67	2.48589374	-6.86222878	-4.25489414
O68	3.72507880	-7.03178389	-4.11433916
O69	1.59454842	-7.31816438	-3.50285015
K70	-0.42787639	-7.11705925	-5.08993931

Int4-7

E(M06-2X/6-31G(d)(THF)) = -2004.834894180
E(M06-2X/6-311+G(d,p)) = -2005.268975464
G(M06-2X/6-311+G(d,p)(THF)) = -2004.83668

C1	-6.13530397	-7.94953684	-3.67674993
C2	-6.51900662	-9.20515955	-4.14181483
C3	-5.62910754	-9.99840008	-4.86959429
C4	-4.33512772	-9.56575792	-5.14611947
C5	-3.94872351	-8.31632389	-4.66887048
C6	-4.84414729	-7.51947026	-3.95124084
H7	-6.82375528	-7.32475813	-3.11557776
H8	-7.52199242	-9.56626050	-3.93849728
H9	-5.94897364	-10.97051593	-5.23197726
H10	-3.65534064	-10.18349769	-5.72381932
C11	-2.60861616	-6.46977045	-4.28601749
C12	-0.34657239	-6.18566849	-5.03533478
C13	-0.28276211	-7.70551202	-5.01246822
C14	-1.57423327	-8.31107202	-5.54687640
H15	-0.36062675	-5.79024199	-6.05657250
H16	0.51455231	-5.75678069	-4.51637303
H17	0.55770022	-8.04377659	-5.62322586
H18	-0.12150490	-8.04964546	-3.98673552
H19	-1.59899135	-9.38807753	-5.36269279
H20	-1.70145953	-8.13388070	-6.62096681
N21	-2.70093377	-7.70697989	-4.82863206
S22	-4.10170731	-5.98892166	-3.49549022
N23	-1.54771934	-5.71387750	-4.33382331
C24	-1.26983658	-2.94769982	-6.02262271
C25	-0.81570629	-3.35179953	-4.75076594
O26	-0.68209244	-2.26481100	-6.86124989

O27	-2.55275334	-3.41815059	-6.27241425
C28	-3.13141159	-2.92440026	-7.46906249
H29	-4.12454845	-3.37336917	-7.53071685
H30	-3.21665884	-1.83372710	-7.44936913
H31	-2.53636810	-3.20629434	-8.34192964
C32	-0.08495488	-4.66825795	-1.76799124
H33	-0.10406057	-4.39034192	-0.70232262
C34	0.30165108	-9.00249280	-1.49368561
C35	1.39300674	-8.22122168	-1.86123652
C36	1.25972368	-6.83872630	-1.99291057
C37	0.04113328	-6.20039106	-1.74510576
C38	-1.04544317	-7.00051427	-1.36540801
C39	-0.92412482	-8.38300250	-1.25246852
H40	0.40401324	-10.07930782	-1.39127819
H41	2.35773243	-8.68477301	-2.05014632
H42	2.12288514	-6.25083358	-2.28730723
H43	-2.00507144	-6.54380486	-1.13570120
H44	-1.78649518	-8.97541143	-0.95754639
C45	1.10331643	-3.96182726	-2.34377197
C46	3.30592909	-3.02073225	-2.39438069
C47	2.65390666	-2.69999987	-3.58458920
C48	3.26912260	-1.95842023	-4.57333686
C49	4.58841135	-1.54202536	-4.33585840
C50	5.24428771	-1.86390149	-3.14963277
C51	4.60140273	-2.61471760	-2.15268300
H52	2.74625740	-1.70879285	-5.49225646
H53	5.10667391	-0.95660963	-5.09032181
H54	6.26516330	-1.52549898	-2.99499877
H55	5.09893902	-2.86865616	-1.22067306
C56	1.28319297	-3.27752028	-3.52439142
O57	0.53720559	-3.02935847	-4.56275201
C58	2.33678010	-3.81750216	-1.56022688
O59	2.54702749	-4.25487347	-0.43782817
C60	-1.39930709	-4.13957988	-2.35922044
H61	-1.48265484	-3.07687538	-2.10673036
H62	-2.24334724	-4.63508149	-1.86733656
C63	-1.59945724	-4.22402284	-3.87810083
H64	-2.64028330	-3.95952650	-4.08653691

TS-elim-7
E(M06-2X/6-31G(d)(THF)) = -2004.834894180
E(M06-2X/6-311+G(d,p)) = -2005.26828048
G(M06-2X/6-311+G(d,p)(THF)) = -2004.83485

C1	-6.09502512	-7.92541731	-3.50359022
C2	-6.53472870	-9.12882764	-4.05236621
C3	-5.70443813	-9.87141449	-4.89333290
C4	-4.41732518	-9.43859243	-5.20394181
C5	-3.97533150	-8.24166809	-4.64713023
C6	-4.81263140	-7.49467547	-3.81221181
H7	-6.73694690	-7.33971432	-2.85207093
H8	-7.53379701	-9.48648941	-3.82422046
H9	-6.06511225	-10.80279202	-5.31879867
H10	-3.78268015	-10.01597950	-5.86868663
C11	-2.56513612	-6.45310950	-4.18861856
C12	-0.40629445	-6.11262243	-5.09590301
C13	-0.32148492	-7.63234967	-5.16498359
C14	-1.64790665	-8.22258145	-5.63408221
H15	-0.54934998	-5.67506767	-6.09176069
H16	0.50793648	-5.68618927	-4.67177144
H17	0.47302244	-7.93333930	-5.85273773
H18	-0.08184683	-8.02334878	-4.17118416
H19	-1.66097488	-9.30664861	-5.48952677
H20	-1.83770606	-8.00937557	-6.69253158
N21	-2.72730720	-7.64685899	-4.82809869
S22	-4.00656085	-6.02873684	-3.26074038
N23	-1.51433035	-5.70615270	-4.23510575
C24	-1.19784198	-2.86330792	-6.01461687
C25	-0.78221682	-3.21325103	-4.69116187
O26	-0.57788818	-2.19457114	-6.83063096
O27	-2.43565230	-3.37992269	-6.31276691
C28	-2.96080287	-2.96423350	-7.56671087
H29	-3.93272688	-3.45041968	-7.65968449
H30	-3.07938775	-1.87773769	-7.60153269
H31	-2.30425908	-3.26857487	-8.38556023
C32	-0.11580269	-4.43531608	-1.68801262
H33	-0.17170592	-4.16829225	-0.62117432
C34	0.37628585	-8.75483811	-1.50262220
C35	1.39612520	-7.96096844	-2.01611995
C36	1.23347621	-6.57785960	-2.11226194
C37	0.05804611	-5.95964023	-1.68404044
C38	-0.95222556	-6.76973336	-1.14914428
C39	-0.80540351	-8.15007799	-1.07226942
H40	0.50109153	-9.83155093	-1.42756158
H41	2.32732909	-8.41417097	-2.34639068
H42	2.03966113	-5.97471164	-2.51978261
H43	-1.86801764	-6.31597721	-0.77626120
H44	-1.60751206	-8.75472803	-0.65736505
C45	1.06347153	-3.69628218	-2.22982935
C46	3.26808994	-2.74944046	-2.24101935
C47	2.63738992	-2.44462300	-3.44710288
C48	3.26770450	-1.70808622	-4.42936489
C49	4.57876856	-1.27850527	-4.16766659
C50	5.21213220	-1.58343868	-2.96548771
C51	4.55433128	-2.33084588	-1.97526281
H52	2.76434093	-1.47221872	-5.36248646
H53	5.10802008	-0.69636683	-4.91685065
H54	6.22663600	-1.23584368	-2.79277129
H55	5.03418688	-2.57323259	-1.03106929
C56	1.27105074	-3.03661178	-3.41100876
O57	0.54882134	-2.82270353	-4.48149360
C58	2.28659637	-3.54477123	-1.42010528
O59	2.47163666	-3.97477318	-0.29377957
C60	-1.43472633	-3.94837634	-2.30786072
H61	-1.58027513	-2.90193858	-2.01532773
H62	-2.26464686	-4.50574531	-1.86094286
C63	-1.58454444	-3.98433198	-3.82193603
H64	-2.62848079	-3.85494119	-4.10911548

Product (4+3)
E(M06-2X/6-31G(d)(THF)) = -1110.30702177
E(M06-2X/6-311+G(d,p)) = -1110.593569289
G(M06-2X/6-311+G(d,p)(THF)) = -1110.32871

C1	1.00691563	0.30751856	-0.03348730
C2	0.17179296	1.42001705	-0.23470654
C3	-1.19664216	1.27016300	-0.43756559
C4	-1.78488936	-0.00553854	-0.44761361
H5	2.07453078	0.42514108	0.12507389
H6	0.60436898	2.41645182	-0.23137194
H7	-1.81561224	2.14947636	-0.58956013
H8	-2.85143954	-0.13945430	-0.60455838

C9 -0.95857433 -1.08989322 -0.25043218
C10 0.41521076 -0.93895782 -0.04686180
C11 0.99329127 -2.29795926 0.13160329
C12 0.04400555 -3.25814101 0.04910006
C13 -1.25133451 -2.56384267 -0.20920247
O14 -2.32843169 -3.09678585 -0.37227506
C15 0.12576334 -4.74551110 0.12312626
H16 -0.68278980 -5.13359724 -0.51009541
C17 1.46596211 -5.24373641 -0.46429457
H18 1.47037691 -6.33711646 -0.46739441
H19 1.52722397 -4.91909218 -1.51021430
C20 -0.44284312 -6.45395547 4.05744364
C21 -0.83906793 -7.14725017 2.91500903
C22 -0.66579065 -6.57536773 1.65868312
C23 -0.09553260 -5.30675139 1.52135069
C24 0.29562264 -4.61934979 2.67131686
C25 0.12438911 -5.18904023 3.93162811
H26 -0.58189643 -6.89545779 5.04018778
H27 -1.28932430 -8.13209089 3.00368012
H28 -0.98349547 -7.11580988 0.76874245
H29 0.72529409 -3.62449563 2.58513074
H30 0.42943702 -4.63833298 4.81729647
C31 3.00274783 -3.50420758 0.61149489
C32 2.66899963 -4.75725302 0.29043714
H33 3.39423698 -5.49306974 0.62554825
O34 2.31720481 -2.33954640 0.35336130
C35 4.29842354 -3.25150500 1.32412609
O36 5.08448904 -4.12782664 1.60333827
O37 4.48319781 -1.96476185 1.61521614
C38 5.70956052 -1.67095346 2.29647308
H39 5.70142973 -0.59490654 2.46229681
H40 5.75145264 -2.20849494 3.24604962
H41 6.56269871 -1.96120398 1.67974806

ITU5-catalysed cycloaddition

ITU5
E(M06-2X/6-31G(d)(THF)) = -1243.38648177
E(M06-2X/6-311+G(d,p)) = -1243.624534447
G(M06-2X/6-311+G(d,p)(THF)) = -1243.32263

C1 -0.53195137 0.18297939 -2.79102979
C2 -1.77281582 0.59739273 -3.27994290
C3 -2.94809071 0.02565946 -2.79727301
C4 -2.91671418 -0.97284784 -1.82330265
C5 -1.67942336 -1.39374521 -1.34545659
C6 -0.49666188 -0.80986672 -1.82465538
H7 0.38782501 0.62983481 -3.15771758
H8 -1.81761645 1.37434783 -4.03676425
H9 -3.90770512 0.36197456 -3.17828339
H10 -3.83713541 -1.40335129 -1.44105734
S11 0.91551297 -1.50325038 -1.02982158
C12 -0.12141248 -2.56565775 -0.03921223
N13 0.36508309 -3.34779509 0.83740639
C14 -0.59364026 -4.14417568 1.60799181
C15 -1.87294937 -4.45530975 0.78952546
C16 -2.48931219 -3.16090490 0.25642716
H17 -2.95197625 -2.57435786 1.06017544
N18 -1.44798435 -2.37056862 -0.38576564
H19 -1.51285829 -5.01343156 -0.08635885
H20 -3.26137080 -3.38615970 -0.48836519
C21 -1.59351035 -2.36343747 5.41828808
C22 -1.50204254 -1.56500805 4.28017481
C23 -1.14431915 -2.12660195 3.05716556
C24 -0.88666719 -3.49652267 2.95001137
C25 -0.95366666 -4.28234159 4.10295357
C26 -1.30904939 -3.72393963 5.32814773
H27 -1.87130758 -1.92445598 6.37238137
H28 -1.70313419 -0.49914341 4.34500588
H29 -1.05726506 -1.48794325 2.18099269
H30 -0.72379383 -5.34400836 4.03897860
H31 -1.35901136 -4.35133728 6.21387979
H32 -0.11023850 -5.10775938 1.81121294
C33 -2.89655143 -5.35137612 1.52580246
H34 -2.32244675 -5.98519118 2.21621357
C35 -3.94078045 -4.58804595 2.35074719
H36 -4.52765363 -5.29519937 2.94620640
H37 -3.49241620 -3.86183645 3.03488440
H38 -4.64228250 -4.05633823 1.69625328
C39 -3.60628661 -6.27682644 0.53211969
H40 -4.32582252 -6.92361073 1.04512095
H41 -4.15977727 -5.69597599 -0.21624212
H42 -2.89180704 -6.91439384 0.00191472

(S)-TS-add-γ
E(M06-2X/6-31G(d)(THF)) = -2353.675370080
E(M06-2X/6-311+G(d,p)) = -2354.206121956
G(M06-2X/6-311+G(d,p)(THF)) = -2353.61658

C1 -0.27855935 -8.70671596 1.93505251
C2 -0.31513150 -8.96426809 3.30312134
C3 -0.75102813 -7.98924870 4.20346612
C4 -1.16481932 -6.73518526 3.76375005
C5 -1.13339703 -6.48449235 2.39610807
C6 -0.68975872 -7.45559984 1.49482245
H7 0.06200467 -9.46042234 1.23116498
H8 0.00262882 -9.93431335 3.67169939
H9 -0.76601861 -8.20797716 5.26645085
H10 -1.49259927 -5.97708963 4.46796466
C11 -1.32632880 -5.32197969 0.42079490
C12 -2.09013157 -3.03112507 0.16740516
C13 -2.92569812 -3.34895690 1.42317417
C14 -2.09088081 -4.13550315 2.43050522
H15 -2.77190191 -2.67542321 -0.60988043
H16 -3.70752273 -4.02503820 1.06049809
H17 -2.72248204 -4.51246800 3.24053827
H18 -1.28287157 -3.53463713 2.86351710
N19 -1.50605289 -5.29793180 1.75379101
S20 -0.74548196 -6.86086623 -0.16250446
N21 -1.52698568 -4.29148343 -0.36250896
C22 -1.40156844 -4.46808932 -1.80075832
H23 -2.32504118 -5.28206161 -3.52831731
C24 -2.45695675 -5.07104251 -2.47433009
C25 -1.24032325 -1.97345788 -3.30819644
H26 -1.38474568 -1.47438889 -2.35034689
C27 2.48658334 -0.74329153 -5.01978998
C28 1.55317677 -1.39223758 -5.82444514
C29 0.33573327 -1.81918430 -5.30089240
C30 0.03247765 -1.59807184 -3.95173740

C31	0.98221983	-0.94331409	-3.14972690
C32	2.19557061	-0.52000935	-3.67456919
H33	3.43361705	-0.41208839	-5.43677086
H34	1.77219923	-1.56863492	-6.87378161
H35	-0.37825784	-2.33580264	-5.93090474
H36	0.75446142	-0.77471419	-2.09807979
H37	2.91476974	-0.01366833	-3.03639964
C38	-2.43531601	-2.43063880	-3.84926339
C39	-4.78390675	-2.86402651	-3.91523903
C40	-4.22313851	-3.46402964	-5.03897288
C41	-5.00042706	-4.15182797	-5.95207238
C42	-6.37836909	-4.21269343	-5.71514191
C43	-6.94148542	-3.60519229	-4.59099237
C44	-6.14300830	-2.92005567	-3.66774050
H45	-4.54872951	-4.62403546	-6.81997519
H46	-7.02226717	-4.73848539	-6.41492918
H47	-8.01475636	-3.66818697	-4.43351942
H48	-6.56600380	-2.44647523	-2.78611247
C49	-2.73267479	-3.25421507	-5.02512624
O50	-1.97403385	-3.75847151	-5.84608343
C51	-3.68679045	-2.23187476	-3.11249204
O52	-3.85927239	-1.62908668	-2.05338783
C53	-0.30557309	-3.87893020	-2.40144303
H54	0.49340580	-3.45616967	-1.80230537
H55	-0.08433971	-4.13588091	-3.43122191
C56	-3.70743350	-5.47629148	-1.88033647
O57	-4.03866181	-5.42965773	-0.69828211
O58	-4.55495060	-5.94776497	-2.82765942
C59	-5.84572677	-6.31478906	-2.36018558
H60	-6.40392403	-6.62258712	-3.24443389
H61	-5.78156432	-7.13755258	-1.64260731
H62	-6.33796840	-5.46454865	-1.88142159
C63	-3.60766188	-2.10553512	2.04013494
H64	-3.79063808	-1.40564423	1.21414881
C65	0.86399209	0.02833650	0.87278082
C66	-0.37746887	0.34892818	0.32577070
C67	-1.30801051	-0.65470032	0.06896325
C68	-1.01113780	-1.98757940	0.36982093
C69	0.24137844	-2.30438102	0.90063464
C70	1.17539255	-1.30127541	1.15063697
H71	1.59191201	0.81030054	1.06957772
H72	-0.61837287	1.38088890	0.08610552
H73	-2.26765186	-0.41471080	-0.38543231
H74	0.50155787	-3.34134347	1.10754799
H75	2.14834449	-1.55944220	1.55952362
C76	-4.97066952	-2.49535848	2.62058219
H77	-5.47509664	-1.62095401	3.04404256
H78	-5.61881311	-2.92655973	1.85220757
H79	-4.85968888	-3.23418026	3.42411337
C80	-2.77673919	-1.38103956	3.10587355
H81	-3.28396070	-0.45801283	3.40287802
H82	-2.67425501	-1.99647543	4.00819627
H83	-1.77574942	-1.11568991	2.75553849

(*R*)-TS-add-γ
E(M06-2X/6-31G(d)(THF)) = -2353.659355527
E(M06-2X/6-311+G(d,p)) = -2354.187762644
G(M06-2X/6-311+G(d,p)(THF)) = -2353.60009

C1	-0.90785444	-8.83904355	1.97612513
C2	-0.90019107	-9.04005156	3.35415670
C3	-1.13249977	-7.98013650	4.23414919
C4	-1.38102850	-6.69411188	3.76368768
C5	-1.39111817	-6.49987588	2.38649468
C6	-1.14962582	-7.55492441	1.50529656
H7	-0.72637605	-9.65979579	1.28834235
H8	-0.70792313	-10.03334293	3.74704067
H9	-1.11751468	-8.15779869	5.30480001
H10	-1.55345850	-5.87199269	4.45088329
C11	-1.52381623	-5.38387066	0.37636874
C12	-1.75261189	-2.98764226	0.12405119
C13	-2.60015220	-3.08103220	1.40750756
C14	-1.94857760	-4.03927476	2.39872466
H15	-2.35103298	-2.45940952	-0.62329406
H16	-3.53833096	-3.54941630	1.08372950
H17	-2.64327708	-4.27954739	3.20984798
H18	-1.02997384	-3.62832786	2.83250258
N19	-1.62053404	-5.29317965	1.71461641
S20	-1.22763822	-7.01791655	-0.17047505
N21	-1.57684312	-4.34452645	-0.42772885
C22	-1.59117810	-4.53692575	-1.86841665
H23	-2.59366021	-5.55653984	-3.43764913
C24	-2.63051299	-5.29772434	-2.38512944
C25	-1.49727748	-2.01475163	-3.56182839
H26	-1.89012290	-1.48775783	-2.68965727
C27	-0.61225532	-3.86229182	-2.59452300
H28	0.21071053	-3.38083743	-2.07878376
H29	-0.43911474	-4.15129433	-3.62437001
C30	-3.84827735	-5.62691689	-1.67937376
O31	-4.15502102	-5.36407618	-0.51893276
O32	-4.69804061	-6.31245439	-2.48495786
C33	-5.94926530	-6.64324329	-1.89633445
H34	-6.51119300	-7.17341104	-2.66554616
H35	-5.81018092	-7.28165334	-1.01975827
H36	-6.48496234	-5.74106567	-1.58911169
C37	-2.92285613	-1.69960011	2.02293948
H38	-2.98075121	-0.98841809	1.18756056
C39	2.01922932	-0.94426830	0.55150265
C40	0.93836031	-0.31605381	-0.05801488
C41	-0.27415519	-0.98824070	-0.19365266
C42	-0.41519712	-2.29411483	0.26915080
C43	0.68373781	-2.93160793	0.85674384
C44	1.89112887	-2.25678732	1.00461535
H45	2.96723019	-0.42459428	0.65478251
H46	1.02993697	0.68917965	-0.45636736
H47	-1.09941354	-0.49048338	-0.69401319
H48	0.61433948	-3.96956344	1.17718395
H49	2.73846326	-2.76245479	1.45898079
C50	-4.29738296	-1.73614912	2.69777366
H51	-4.54769450	-0.75649592	3.11643883
H52	-5.08271264	-2.01616551	1.98893922
H53	-4.31135988	-2.46097509	3.52103863
C54	-1.87281718	-1.17601088	3.01079518
H55	-2.11725430	-0.14696704	3.29096056
H56	-1.87060721	-1.77013051	3.93311850
H57	-0.86117898	-1.17882609	2.59584223
C58	-0.32683437	-1.41219749	-4.05045764
C59	0.67231059	-1.89940062	-4.98391814
O60	0.72920637	-2.96284650	-5.60097104
C61	1.75913414	-0.85598596	-5.03287258
C62	1.43312785	0.18612822	-4.16913392
C63	2.25791827	1.28622907	-4.02208350

C64	3.44275890	1.31419978	-4.76730323
C65	3.77147311	0.26679047	-5.62996363
C66	2.92634918	-0.83974453	-5.77396486
H67	1.98905292	2.09487847	-3.34816596
H68	4.11791426	2.16081944	-4.67706677
H69	4.69711135	0.31566460	-6.19685933
H70	3.16980001	-1.66121915	-6.44171638
C71	0.12290947	-0.13613091	-3.50620745
O72	-0.44558355	0.59630747	-2.69885622
C73	-4.61627416	-4.17513908	-5.52216789
C74	-3.35798136	-4.08916928	-6.12603954
C75	-2.32212200	-3.39951689	-5.51324369
C76	-2.52479316	-2.77625523	-4.26957860
C77	-3.78496302	-2.89061341	-3.66406605
C78	-4.82814252	-3.57066232	-4.28972449
H79	-5.42110998	-4.71686011	-6.01172099
H80	-3.18732034	-4.56431780	-7.08817658
H81	-1.34438070	-3.34616538	-5.97932816
H82	-3.95169418	-2.42297744	-2.69509336
H83	-5.79662926	-3.64094646	-3.80206839

DABCO-catalysed cycloaddition

DABCO

E(M06-2X/6-31G(d)(THF)) = -345.180031253
E(M06-2X/6-311+G(d,p)) = -345.267961985
G(M06-2X/6-311+G(d,p)(THF)) = -345.117082

N1	-0.24473408	-2.03834695	-0.00967676
C2	0.59767531	-2.19912596	1.18371634
H3	-0.03631946	-2.06707231	2.06768385
H4	1.33834979	-1.39157437	1.18959305
C5	0.59532832	-2.19476535	-1.20535742
H6	1.34247581	-1.39311129	-1.20374076
H7	-0.03771787	-2.04914957	-2.08787139
C8	-1.26561465	-3.09515455	-0.01071361
H9	-1.90195603	-2.95421444	0.87005374
H10	-1.89668653	-2.95665916	-0.89571174
C11	-0.58780763	-4.49913141	-0.00679264
H12	-0.86773739	-5.07752302	0.88068994
H13	-0.87762143	-5.08705814	-0.88475174
C14	1.26811051	-3.60111486	-1.21168620
H15	0.97444158	-4.18263996	-2.09260260
H16	2.36099097	-3.52323180	-1.22037100
C17	1.28168438	-3.60007512	1.17624203
H18	2.37387913	-3.51319805	1.16587708
H19	1.00821600	-4.18510668	2.06130828
N20	0.87502098	-4.35877778	-0.01514840

TS-DABCO

E(M06-2X/6-31G(d)(THF)) = -689.558875420
E(M06-2X/6-311+G(d,p)) = -689.74643857
G(M06-2X/6-311+G(d,p)(THF)) = -689.507041

C1	0.33900662	-2.44460345	1.23718732
H2	-0.50916565	-2.68673266	1.87910931
H3	0.83117819	-1.55860490	1.64733756
C4	0.90498337	-1.91881044	-1.03974621
H5	1.58864109	-1.17900313	-0.61186098
H6	0.51762334	-1.51640185	-1.97788743
C7	-1.09671939	-3.18610522	-0.54166303
H8	-1.97642013	-3.17846763	0.10648213
H9	-1.41629798	-2.95243258	-1.56173912
C10	-0.30029198	-4.52096957	-0.47209371
H11	-0.66028342	-5.14480419	0.35221339
H12	-0.42026281	-5.09244783	-1.39772608
C13	1.58871189	-3.30051098	-1.26085023
H14	1.34894850	-3.69971709	-2.25181246
H15	2.67719216	-3.20592161	-1.19490195
C16	1.30045950	-3.65090336	1.06812050
H17	2.34693701	-3.34328587	1.16594718
H18	1.10360109	-4.40396371	1.83723009
N19	1.13023626	-4.26314459	-0.25592909
N20	-0.21543147	-2.08578194	-0.08684724
C21	-1.67044478	-0.16759125	-1.09910748
C22	-1.30185655	-0.48844112	0.12779284
H23	-2.40569141	0.62279203	-1.22217667
H24	-1.29032743	-0.63168364	-2.00246581
C25	-1.42236454	-0.08710695	1.43727941
H26	-0.92837502	0.81487821	1.78020683
C27	-2.20693365	-0.84844546	2.35805643
O28	-2.74470404	-1.93790325	2.15707141
O29	-2.31539662	-0.22981615	3.57007395
C30	-3.08802953	-0.92679871	4.53801364
H31	-3.07239976	-0.30837192	5.43630116
H32	-4.11601386	-1.06422571	4.19320425
H33	-2.65736524	-1.90926706	4.74916035

DABCO-allene adduct

E(M06-2X/6-31G(d)(THF)) = -689.575520792
E(M06-2X/6-311+G(d,p)) = -689.761177342
G(M06-2X/6-311+G(d,p)(THF)) = -689.519710

C1	0.79672208	-2.25706661	1.07605213
H2	0.16865672	-2.33928096	1.96055671
H3	1.41148213	-1.35574827	1.12878198
C4	0.69048392	-2.11974535	-1.34765207
H5	1.50920091	-1.40668167	-1.23086655
H6	0.06294534	-1.81208424	-2.18435765
C7	-1.15781878	-3.16582163	-0.20788287
H8	-1.92187743	-3.01763165	0.54690009
H9	-1.58918923	-3.06260269	-1.20744728
C10	-0.40668092	-4.49481513	-0.00424378
H11	-0.53385295	-4.83549132	1.02765058
H12	-0.81525151	-5.26050182	-0.67057464
C13	1.18965054	-3.57930831	-1.50831313
H14	0.62986033	-4.09085469	-2.29728502
H15	2.24426865	-3.57563597	-1.79856862
C16	1.61641701	-3.54424995	0.82192500
H17	2.65158140	-3.31017315	0.55382962
H18	1.63549765	-4.15018233	1.73207181
N19	1.02931409	-4.33173970	-0.26536143
N20	-0.14702443	-2.03710409	-0.09600860
C21	-0.61532943	0.25674814	-0.81534937
C22	-0.85156282	-0.71503173	0.09658275
H23	-1.10291841	1.21408448	-0.67176682
H24	0.03122110	0.16695870	-1.67347444
C25	-1.69280492	-0.55548057	1.24345517
H26	-2.13299288	0.43385072	1.29374766
C27	-2.05592276	-1.39548359	2.30875105

O28	-1.74197862	-2.57670712	2.56961156
O29	-2.90605062	-0.73718723	3.17634617
C30	-3.33211979	-1.49796228	4.29242263
H31	-3.98677661	-0.84218312	4.86927937
H32	-3.88113938	-2.39138152	3.98117669
H33	-2.48430056	-1.81290660	4.90768503

TS-add-γ
E(M06-2X/6-31G(d)(THF)) = -1455.449736511
E(M06-2X/6-311+G(d,p)) = -1455.82743460
G(M06-2X/6-311+G(d,p)(THF)) = -1455.39009

C1	-1.50579568	-4.31109759	-1.63832041
H2	-2.17797263	-5.18473776	-3.39729521
C3	-2.47477649	-4.99610082	-2.37253938
C4	-1.35185357	-1.91805991	-3.35503926
H5	-1.57680828	-1.31259669	-2.47380249
C6	2.35677658	-0.62364728	-5.07080902
C7	1.61635739	-1.60993998	-5.71977134
C8	0.41349709	-2.06355963	-5.18771985
C9	-0.07494059	-1.52760129	-3.98746108
C10	0.68420213	-0.54045548	-3.33982426
C11	1.88551055	-0.08923571	-3.87356507
H12	3.29338986	-0.27322068	-5.49532216
H13	1.97772125	-2.03198768	-6.65363473
H14	-0.15460769	-2.83518473	-5.69498212
H15	0.31395562	-0.11494251	-2.40886794
H16	2.45106749	0.68253640	-3.35870371
C17	-2.50822867	-2.44731480	-3.91621503
C18	-4.85457700	-2.87015473	-4.09943060
C19	-4.22813561	-3.54931211	-5.14083598
C20	-4.95249092	-4.28231962	-6.06375685
C21	-6.34442169	-4.30632331	-5.92403877
C22	-6.97408750	-3.62204362	-4.88107630
C23	-6.23008699	-2.89286938	-3.94650273
H24	-4.44946964	-4.81374048	-6.86681821
H25	-6.94770855	-4.86348744	-6.63590316
H26	-8.05698097	-3.65851620	-4.79757888
H27	-6.70725367	-2.35887701	-3.12949768
C28	-2.73947217	-3.35632725	-5.04220984
O29	-1.93686516	-3.93374175	-5.76850118
C30	-3.80389000	-2.18455219	-3.27630219
O31	-4.02431499	-1.50124501	-2.28115024
C32	-0.43441612	-3.72088316	-2.28987829
H33	0.39862192	-3.24467888	-1.79366439
H34	-0.23345836	-4.08025047	-3.29272620
C35	-3.78187562	-5.52205157	-2.06147766
O36	-4.38852362	-5.54443119	-0.98915355
O37	-4.34004965	-6.04184468	-3.17996390
C38	-5.67218111	-6.52090724	-3.03869102
H39	-5.97199360	-6.85232058	-4.03290033
H40	-5.71387879	-7.35196784	-2.32928577
H41	-6.33230542	-5.72104869	-2.69422985
N42	-2.10801405	-3.63675219	2.34770045
C43	-2.98723160	-2.77908309	1.54824058
H44	-2.70038996	-1.73730630	1.72202543
H45	-4.01800304	-2.90254258	1.89176593
C46	-2.37091957	-5.03597598	1.99758326
H47	-3.45005786	-5.20800006	2.05341009
H48	-1.88404440	-5.69361782	2.72396907
C49	-0.72330812	-3.32467693	2.00091532
H50	-0.46514608	-2.33480182	2.38785518
H51	-0.07048808	-4.05411530	2.49005620
C52	-0.50926942	-3.35248772	0.46471737
H53	-0.45893142	-2.35086230	0.03193440
H54	0.38672082	-3.90963607	0.18850309
C55	-1.85883688	-5.35520290	0.57805090
H56	-0.86927590	-5.82038605	0.57085801
H57	-2.55900174	-5.97359446	0.02717094
C58	-2.89664039	-3.13695640	0.04608715
H59	-3.76477821	-3.69060517	-0.30306383
H60	-2.74892436	-2.27077351	-0.59962044
N61	-1.69245161	-4.04357642	-0.17153259

Int1
E(M06-2X/6-31G(d)(THF)) = -1455.47826945
E(M06-2X/6-311+G(d,p)) = -1455.851558634
G(M06-2X/6-311+G(d,p)(THF)) = -1455.41519

C1	0.30623353	-1.94792799	-1.59275610
H2	0.67785790	-2.34957273	-0.65514538
H3	0.90292008	-1.08979667	-1.91166865
C4	-1.66105075	-0.99079901	-2.65474891
H5	-0.91569017	-0.34708446	-3.12679454
H6	-2.58032208	-0.42974599	-2.48271584
C7	-2.01478268	-2.44425503	-0.74056697
H8	-1.77879111	-2.59282411	0.30758264
H9	-3.02252663	-2.02958125	-0.83395824
C10	-1.81632210	-3.73228003	-1.56653100
H11	-1.15312037	-4.42156860	-1.03518986
H12	-2.78107970	-4.22918857	-1.70540554
C13	-1.93466163	-2.29200994	-3.45245399
H14	-3.00481274	-2.52025120	-3.46385520
H15	-1.61208504	-2.15132456	-4.48770814
C16	0.17530971	-3.04150085	-2.67895733
H17	0.56749650	-2.68703662	-3.63678080
H18	0.75598986	-3.92015868	-2.38406622
N19	-1.22378768	-3.42965594	-2.87107851
N20	-1.08504553	-1.37786658	-1.30134413
C21	-0.97013686	-0.22640962	-0.34217459
H22	-2.55932078	1.18168452	-0.59293638
C23	-0.44155700	-0.36059673	0.88276042
H24	-0.37155470	0.56085305	1.45110792
C25	0.09688459	-1.51477718	1.65509775
O26	0.09575693	-2.69429196	1.35277990
O27	0.59394258	-1.06456887	2.80393011
C28	1.17026826	-2.04769803	3.67248694
H29	1.53888138	-1.49464506	4.53482990
H30	0.41201983	-2.77393950	3.97303928
H31	1.98795281	-2.56329262	3.16502385
C32	-1.48268887	1.11723898	-0.80644582
H33	-1.35295933	1.20693360	-1.88115363
C34	-0.75704621	2.34109532	-0.19137542
H35	-0.98627677	2.42183974	0.88027003
C36	-2.35148327	5.87405519	-2.12387057
C37	-2.67573654	5.59987698	-0.79884880
C38	-2.16410223	4.46209133	-0.17314324
C39	-1.31827207	3.59165094	-0.85875331
C40	-0.99270791	3.87781712	-2.18975986
C41	-1.50722698	5.00631769	-2.81827553
H42	-2.74569937	6.76085027	-2.61221655
H43	-3.32321506	6.27534313	-0.24600528

H44	-2.41167800	4.25769363	0.86658149
H45	-0.32040572	3.20870523	-2.72405879
H46	-1.24090529	5.21697423	-3.85072224
C47	0.72761007	2.21076794	-0.33391080
C48	1.41615462	1.58007120	-1.38630093
O49	0.97707637	1.01102708	-2.42160802
C50	1.64047509	2.59486025	0.70365633
O51	1.39713387	3.06622111	1.81734054
C52	3.03115528	2.25221131	0.20094413
C53	2.89217880	1.63955735	-1.04596445
C54	3.99123893	1.20156946	-1.75447658
C55	5.26103841	1.39555557	-1.18277188
C56	5.39833461	2.00704226	0.05932025
C57	4.26939130	2.44491139	0.77393505
H58	3.87276438	0.72819057	-2.72587989
H59	6.14855096	1.06650529	-1.71718171
H60	6.39075232	2.14820584	0.47940459
H61	4.36216515	2.92288699	1.74570111

TS-cycl-6
E(M06-2X/6-31G(d)(THF)) = -1455.455847228
E(M06-2X/6-311+G(d,p)) = -1455.833458147
G(M06-2X/6-311+G(d,p)(THF)) = -1455.39019

H1	2.34035191	-4.25910043	-0.41467210
C2	3.10932432	-2.34094962	-0.02621189
C3	1.86228727	-1.94386899	-0.52775733
H4	1.09011967	-2.69393614	-0.41586678
C5	1.35356132	-0.70091357	-1.03080518
O6	1.94535184	0.32613803	-1.37507004
O7	0.00284918	-0.78206664	-1.18619878
C8	-0.61640603	0.36662356	-1.75183640
H9	-1.68092831	0.13526582	-1.80273453
H10	-0.45044424	1.24682057	-1.12549730
H11	-0.22478156	0.56926882	-2.75237174
C12	3.29971319	-3.87645509	-0.06769943
H13	4.01042351	-4.11674817	-0.86727469
C14	3.65072986	-4.71138121	1.20555699
C15	2.96320538	-4.10851404	2.37634632
C16	2.40299190	-4.76825034	3.54923594
O17	2.32269562	-5.96208166	3.79169909
C18	2.86464114	-2.75307757	2.52797532
O19	3.27383171	-1.85309523	1.69142258
C20	1.92064912	-3.65226071	4.45783982
C21	2.19464764	-2.43265379	3.82780968
C22	1.85694324	-1.23115183	4.41440520
C23	1.23090917	-1.27373097	5.67278313
C24	0.96089469	-2.48519623	6.29990499
C25	1.30511445	-3.70364237	5.68823873
H26	2.06443297	-0.28661764	3.91983045
H27	0.95311650	-0.34493491	6.16342530
H28	0.47699245	-2.48874945	7.27254078
H29	1.09531023	-4.65787039	6.16415722
N30	4.34601208	-1.53721611	-0.46320858
C31	4.37656130	-0.11487535	0.09807544
H32	4.70113493	-0.21808972	1.13056929
H33	3.37376709	0.29492969	0.06755710
C34	4.36929138	-1.48660985	-1.98078802
H35	3.53874762	-0.84669071	-2.27291640
H36	4.18946452	-2.50036310	-2.34303072
C37	5.64864346	-2.15144236	-0.00657255
H38	5.55148688	-2.35142370	1.05924569
H39	5.77704180	-3.09740347	-0.53347277
C40	6.79699980	-1.16249715	-0.33353587
H41	7.10976576	-0.61723144	0.56201165
H42	7.66214869	-1.72358429	-0.69863080
C43	5.73423677	-0.92525277	-2.43854634
H44	6.40665120	-1.72582735	-2.76317916
H45	5.57998764	-0.25451270	-3.28880023
C46	5.37391516	0.69583892	-0.74551793
H47	4.85875893	1.23561037	-1.54643782
H48	5.86796817	1.43354682	-0.10561709
N49	6.37999641	-0.19095319	-1.34790895
C50	7.90641871	-5.40506627	1.68277825
C51	7.22103129	-5.89551654	0.57125336
C52	5.85328369	-5.67596537	0.44442995
C53	5.14479007	-4.95522705	1.41297519
C54	5.83638078	-4.49067879	2.53143507
C55	7.20755187	-4.71047944	2.66479538
H56	8.97401049	-5.57700923	1.78677070
H57	7.75245196	-6.45849116	-0.19138373
H58	5.32161597	-6.07722794	-0.41776613
H59	5.29735919	-3.94964031	3.30435375
H60	7.72737966	-4.33850316	3.54374166
H61	3.21490551	-5.70175642	1.01660783

Int2-6
E(M06-2X/6-31G(d)(THF)) = -1455.468069957
E(M06-2X/6-311+G(d,p)) = -1455.843170370
G(M06-2X/6-311+G(d,p)(THF)) = -1455.40255

H1	2.26344640	-4.42391145	-0.55566765
C2	2.82305707	-2.45492697	-0.10826445
C3	1.78176688	-2.03866395	-1.03480760
H4	1.11950889	-1.24419524	-0.71747134
C5	1.62404656	-2.61368593	-2.30264485
O6	2.31895918	-3.48294370	-2.85705256
O7	0.53551866	-2.08779840	-2.97499349
C8	0.31284646	-2.63390914	-4.26323508
H9	-0.56038198	-2.11576725	-4.66430136
H10	1.17402903	-2.47530845	-4.91897601
H11	0.11619268	-3.70881122	-4.21303280
C12	3.18084439	-3.96917715	-0.18460560
H13	3.90696496	-4.12552155	-0.98311131
C14	3.60878153	-4.75431145	1.09647964
C15	3.08198479	-4.04740743	2.29463919
C16	2.94540009	-4.54822269	3.68132959
O17	3.28267689	-5.63027008	4.11606890
C18	2.54649828	-2.80962293	2.25272832
O19	2.42173726	-2.00042207	1.20347801
C20	2.29766796	-3.42951946	4.46780474
C21	2.05182849	-2.37244927	3.58293517
C22	1.45728637	-1.20215723	4.00727212
C23	1.11234342	-1.10844012	5.36715794
C24	1.35850954	-2.15628625	6.24734844
C25	1.96037356	-3.34535831	5.79974343
H26	1.25634716	-0.38569179	3.31992060
H27	0.64218217	-0.20121365	5.73584922
H28	1.07976744	-2.05491707	7.29214889
H29	2.15456698	-4.17356866	6.47561334
N30	4.16286926	-1.56851552	-0.31737704
C31	3.89779565	-0.13061422	0.04826827

H32	3.80468850	-0.08937738	1.13368781
H33	2.94080430	0.13786060	-0.40390396
C34	4.56030786	-1.57817113	-1.77386779
H35	3.78748998	-1.01356997	-2.29579153
H36	4.51976466	-2.60289861	-2.14381316
C37	5.33334455	-2.03858797	0.49114330
H38	4.99382858	-2.21658994	1.51390630
H39	5.65515857	-2.98891829	0.06312309
C40	6.45861297	-0.97572015	0.41687140
H41	6.48176782	-0.36239607	1.32313830
H42	7.42815255	-1.47418211	0.32734328
C43	5.95718551	-0.93007712	-1.90380521
H44	6.74104680	-1.68932288	-1.98867384
H45	5.99274812	-0.31590629	-2.80841898
C46	5.07265592	0.73675465	-0.46508868
H47	4.80270677	1.26003551	-1.38748672
H48	5.32432014	1.49285400	0.28462967
N49	6.25111318	-0.09327094	-0.73405494
C50	7.80521629	-5.84149748	1.16268677
C51	6.98496345	-6.20209847	0.09490771
C52	5.63958897	-5.84825110	0.09908958
C53	5.08890330	-5.12194768	1.16109015
C54	5.91539141	-4.78202572	2.23198906
C55	7.26401541	-5.13509470	2.23232302
H56	8.85562528	-6.11814840	1.16371576
H57	7.39186714	-6.76639093	-0.73968214
H58	5.00328930	-6.14680329	-0.73259479
H59	5.50976381	-4.23065425	3.07545605
H60	7.89032793	-4.85912475	3.07629078
H61	3.08417133	-5.71799564	1.02682602

TS-elim-6-*Z*
E(M06-2X/6-31G(d)(THF)) = -1455.464480359
E(M06-2X/6-311+G(d,p)) = -1455.839514295
G(M06-2X/6-311+G(d,p)(THF)) = -1455.40049

H1	4.27656146	-1.87814390	-1.13587979
C2	2.70952326	-2.66280980	0.03328098
C3	1.87287595	-2.15829560	-1.01699909
H4	2.24817691	-1.31016860	-1.57322755
C5	0.68503942	-2.76255182	-1.49070285
O6	0.04435434	-3.72027208	-1.04761451
O7	0.23810575	-2.11794689	-2.62649128
C8	-0.94874364	-2.65307285	-3.18586949
H9	-1.16560236	-2.04875761	-4.06712551
H10	-0.81468477	-3.69795916	-3.47447282
H11	-1.78251645	-2.59658124	-2.48074791
C12	4.17031871	-2.12926407	-0.08047456
H13	4.25872518	-1.18630921	0.46667212
C14	5.37826789	-3.05139528	0.28454269
C15	4.94864485	-4.46481161	0.11264333
C16	5.77864015	-5.67105881	-0.09321666
O17	6.99053065	-5.74005897	-0.12241580
C18	3.65459628	-4.84410280	0.07062445
O19	2.57413578	-4.08298766	0.19954936
C20	4.80710089	-6.82023189	-0.26272634
C21	3.51135146	-6.29936096	-0.16993307
C22	2.39459143	-7.09140816	-0.33291436
C23	2.60920417	-8.45868278	-0.57903651
C24	3.89431603	-8.98299533	-0.66033766
C25	5.02242098	-8.15775115	-0.50658877
H26	1.39563046	-6.66765009	-0.29254976
H27	1.75511127	-9.11593547	-0.71500681
H28	4.02740884	-10.04300418	-0.85516908
H29	6.03048260	-8.55441043	-0.58527274
N30	2.11363169	-2.19673363	1.57122471
C31	0.82489266	-2.92354414	1.85243950
H32	1.09174631	-3.96029045	2.06040882
H33	0.22648489	-2.91169923	0.94107447
C34	1.81002432	-0.73149923	1.56413008
H35	0.98164781	-0.59334200	0.86767373
H36	2.67454167	-0.19421589	1.16791744
C37	3.04816655	-2.48755139	2.69692805
H38	3.40577302	-3.51365518	2.58007007
H39	3.90102706	-1.81272420	2.60412219
C40	2.30004980	-2.26680112	4.03923206
H41	1.99405921	-3.22069212	4.47998705
H42	2.96169505	-1.76649538	4.75252127
C43	1.46827877	-0.29159091	3.01001882
H44	2.32179438	0.20575910	3.48203057
H45	0.63721667	0.41982371	2.99394384
C46	0.13538299	-2.25020054	3.06317591
H47	-0.67431749	-1.58864171	2.73922644
H48	-0.30109791	-3.01468432	3.71307770
N49	1.10017511	-1.45119599	3.82902267
C50	7.26713689	-2.09636521	4.06822820
C51	7.18168313	-1.14723397	3.05027161
C52	6.58032415	-1.47689595	1.83967500
C53	6.04317773	-2.75130174	1.62571029
C54	6.15389123	-3.69864707	2.64284283
C55	6.75772237	-3.37392822	3.85692134
H56	7.73792129	-1.84362865	5.01399279
H57	7.59238442	-0.15198817	3.19695065
H58	6.53350603	-0.73502434	1.04354697
H59	5.75782383	-4.69944279	2.49244131
H60	6.83159791	-4.12627200	4.63749868
H61	6.14911149	-2.83642955	-0.46861849

TS-elim-6-*E*
E(M06-2X/6-31G(d)(THF)) = -1455.467722868
E(M06-2X/6-311+G(d,p)) = -1455.843101750
G(M06-2X/6-311+G(d,p)(THF)) = -1455.40297

H1	2.57680709	-4.35807134	-0.75932672
C2	3.13081248	-2.45004203	-0.03146416
C3	1.95092949	-1.95388871	-0.67552075
H4	1.41640756	-1.14272553	-0.19829689
C5	1.50100850	-2.42835645	-1.92643152
O6	2.02781422	-3.26719755	-2.67149572
O7	0.31846258	-1.82770040	-2.30137779
C8	-0.21374109	-2.26829930	-3.54406908
H9	-1.10415694	-1.66020712	-3.72299881
H10	0.50690652	-2.12927744	-4.35673590
H11	-0.48887471	-3.32728709	-3.50872591
C12	3.44683201	-3.95540424	-0.24557917
H13	4.27992327	-4.08870017	-0.94078899
C14	3.70560053	-4.82519323	1.02477511
C15	2.94336058	-4.24032296	2.15948129
C16	2.40247955	-4.86961206	3.38380073
O17	2.40576171	-6.04343420	3.68298651
C18	2.74436314	-2.91236674	2.25444066
O19	3.12896697	-2.00559563	1.35014919

C20	1.85348130	-3.73216880	4.22072999
C21	2.07226810	-2.53568166	3.52179593
C22	1.67747131	-1.31973313	4.03980824
C23	1.04877506	-1.32467954	5.29813899
C24	0.82976000	-2.51052960	5.99151152
C25	1.23349908	-3.74380892	5.44996294
H26	1.84077774	-0.39133878	3.49880857
H27	0.72886601	-0.38350656	5.73605810
H28	0.34115754	-2.48286629	6.96191224
H29	1.06596369	-4.67840709	5.97901043
N30	4.51720311	-1.54989453	-0.50678318
C31	4.33654905	-0.11398227	-0.11767423
H32	4.45310245	-0.05789040	0.96406491
H33	3.31425600	0.16150356	-0.38402757
C34	4.63277128	-1.58763684	-2.00052454
H35	3.79013256	-1.01174461	-2.38550248
H36	4.50765413	-2.61565469	-2.34237882
C37	5.79455317	-2.02284587	0.10339575
H38	5.62280184	-2.14733220	1.17348055
H39	6.03148409	-3.00033146	-0.32232003
C40	6.90360584	-0.98099488	-0.20259379
H41	7.09638465	-0.34805166	0.66877273
H42	7.83570910	-1.49364372	-0.45578569
C43	5.99453639	-0.97474742	-2.39594953
H44	6.73805800	-1.75425370	-2.58933820
H45	5.88530730	-0.38414632	-3.31029501
C46	5.39707363	0.73416833	-0.86090870
H47	4.96557214	1.23187640	-1.73432769
H48	5.78517247	1.50916092	-0.19385152
N49	6.50260531	-0.11659944	-1.31752032
C50	7.94992978	-5.34861134	1.74750684
C51	7.33884998	-5.88906025	0.61544942
C52	5.97202589	-5.72515496	0.41713325
C53	5.19080618	-5.01034347	1.33342439
C54	5.80790612	-4.49599316	2.47313725
C55	7.17857076	-4.66034080	2.67794187
H56	9.01713143	-5.47701795	1.90685292
H57	7.92761081	-6.44598222	-0.10873435
H58	5.49795889	-6.15831427	-0.46297329
H59	5.21833823	-3.95621888	3.20946542
H60	7.64053705	-4.25020237	3.57220080
H61	3.31382238	-5.82429407	0.79677769

TS-cycl-5
E(M06-2X/6-31G(d)(THF)) = -1455.446959697
E(M06-2X/6-311+G(d,p)) = -1455.822940621
G(M06-2X/6-311+G(d,p)(THF)) = -1455.38208

N1	4.51914315	-2.91525317	-0.94333712
C2	5.30907996	-2.31106340	0.20383074
H3	5.71616347	-3.14697227	0.77362391
H4	4.61080059	-1.76418916	0.83067440
C5	4.14822743	-1.79930036	-1.90209487
H6	3.57261121	-1.08574771	-1.31532533
H7	3.49949263	-2.25964998	-2.64783289
C8	5.45641139	-3.83205201	-1.68176564
H9	5.67761668	-4.67515056	-1.02852736
H10	4.90797200	-4.19668782	-2.55422044
C11	6.75501807	-3.06300523	-2.04089086
H12	7.59076380	-3.43043600	-1.43740308
H13	7.00757370	-3.22884224	-3.09255312
C14	5.42001783	-1.16169875	-2.50672867
H15	5.52021693	-1.41819109	-3.56636333
H16	5.36528867	-0.07169727	-2.43258299
C17	6.42320621	-1.39926338	-0.36861578
H18	6.16540983	-0.34568901	-0.22445296
H19	7.36491325	-1.58746459	0.15574669
N20	6.61625517	-1.62701449	-1.80429365
H21	2.36287234	-5.54137899	-0.27491895
C22	3.23431279	-3.64146801	-0.59686370
C23	2.28196846	-3.06449263	0.30042738
H24	1.34650691	-3.61919745	0.21527250
C25	1.94687220	-1.63925666	0.61660692
O26	2.68576966	-0.67396342	0.62328607
O27	0.67073030	-1.56499149	1.02018290
C28	0.26712458	-0.31822737	1.59334604
H29	-0.77672870	-0.44881813	1.87546684
H30	0.87695810	-0.10087416	2.47415280
H31	0.37239150	0.49019507	0.86625784
C32	3.33304551	-5.06073135	-0.09583923
H33	4.07937207	-5.70618492	-0.56495728
C34	3.54336028	-4.99464212	1.46606924
H35	4.56835463	-4.64111783	1.63663349
C36	2.66266450	-3.88365859	2.01538556
C37	3.29144677	-2.97321396	3.00783024
O38	4.47405981	-2.67358775	3.10355281
C39	1.29205099	-4.14373836	2.53560917
O40	0.46642403	-4.91196591	2.07019125
C41	2.21190855	-2.47684427	3.92131757
C42	1.04035952	-3.18406828	3.66102591
C43	-0.12516130	-2.93626700	4.36962964
C44	-0.08216961	-1.95316557	5.36208201
C45	1.09548355	-1.24461861	5.62579758
C46	2.26526464	-1.50087146	4.90547668
H47	-1.03217362	-3.49536976	4.15745747
H48	-0.97410140	-1.73418923	5.94316933
H49	1.09722159	-0.48767370	6.40562780
H50	3.18747345	-0.96103780	5.10223701
C51	3.27937548	-8.75815249	3.58090152
C52	4.29674503	-7.84965208	3.86296097
C53	4.36439956	-6.64563249	3.16842854
C54	3.42297359	-6.32185812	2.18528505
C55	2.40326026	-7.23998225	1.91430236
C56	2.33478227	-8.44735353	2.60564278
H57	3.22173697	-9.70043642	4.11891617
H58	5.03810164	-8.07757574	4.62422494
H59	5.15651769	-5.93387956	3.39456941
H60	1.64247376	-7.00124544	1.17823626
H61	1.53375138	-9.14726290	2.38325439

Int2-5
E(M06-2X/6-31G(d)(THF)) = -1455.445758072
E(M06-2X/6-311+G(d,p)) = -1455.822105648
G(M06-2X/6-311+G(d,p)(THF)) = -1455.38063

N1	4.51582139	-2.91438579	-0.96556844
C2	5.30882688	-2.31530876	0.18109892
H3	5.69680291	-3.15233771	0.76168218
H4	4.61850374	-1.74909800	0.79778852
C5	4.17166761	-1.80765807	-1.94348641
H6	3.57778652	-1.08832452	-1.38336018
H7	3.53773913	-2.27865784	-2.69403650

C8	5.45415629	-3.84673471	-1.68135822
H9	5.66840798	-4.67703611	-1.00985842
H10	4.90731879	-4.22775664	-2.54733498
C11	6.76149207	-3.09586859	-2.04969246
H12	7.59510924	-3.47217476	-1.44860997
H13	7.00806877	-3.26726366	-3.10185210
C14	5.45732727	-1.17827563	-2.52814036
H15	5.56806810	-1.43205831	-3.58728142
H16	5.41362792	-0.08783791	-2.45086945
C17	6.44675781	-1.42763360	-0.38330030
H18	6.21231155	-0.36855729	-0.23798190
H19	7.38171399	-1.63702391	0.14549367
N20	6.64304739	-1.65763477	-1.81689413
H21	2.33280495	-5.52853918	-0.30136204
C22	3.20457637	-3.63376151	-0.64687514
C23	2.28686941	-3.05720644	0.34039432
H24	1.32461942	-3.55133671	0.18663727
C25	1.98189540	-1.61552493	0.64541624
O26	2.73395771	-0.66191194	0.61338810
O27	0.72033222	-1.51016923	1.08684519
C28	0.35437121	-0.24201675	1.63737691
H29	-0.68209611	-0.34733776	1.95471976
H30	0.99564348	-0.00909587	2.49166826
H31	0.44965439	0.54368095	0.88507252
C32	3.30740689	-5.05240863	-0.13037553
H33	4.04368884	-5.70579227	-0.60427431
C34	3.52464325	-4.97361977	1.42048796
H35	4.54570475	-4.60914017	1.58798837
C36	2.62769429	-3.83729273	1.91728160
C37	3.25160622	-2.94737874	2.95498654
O38	4.42973326	-2.64913595	3.06021269
C39	1.25860257	-4.13974090	2.46458777
O40	0.44710678	-4.91167771	1.99022884
C41	2.17620836	-2.51458356	3.90012669
C42	1.01540458	-3.23595610	3.63161578
C43	-0.14081151	-3.04918599	4.37578156
C44	-0.09888332	-2.11145828	5.40878823
C45	1.06900922	-1.38752554	5.68116762
C46	2.22825564	-1.58318160	4.92869876
H47	-1.03886894	-3.61962022	4.15645391
H48	-0.98286406	-1.93986783	6.01700418
H49	1.06948438	-0.66678711	6.49428391
H50	3.14241526	-1.03262491	5.13269067
C51	3.29040457	-8.66090195	3.66784119
C52	4.27894300	-7.71881058	3.94174975
C53	4.33631620	-6.53922706	3.20548202
C54	3.41180598	-6.27305888	2.18919891
C55	2.42162562	-7.22523840	1.92614066
C56	2.36397044	-8.40854020	2.65870167
H57	3.24163683	-9.58488127	4.23756032
H58	5.00637404	-7.90203614	4.72816831
H59	5.10776054	-5.80236738	3.42295285
H60	1.67835864	-7.03375510	1.15887072
H61	1.58687232	-9.13636392	2.44124028

TS-prot-PhCO$_2$H
E(M06-2X/6-31G(d)(THF)) = -1876.145368859
E(M06-2X/6-311+G(d,p)) = -1876.639781612
G(M06-2X/6-311+G(d,p)(THF)) = -1876.09423

H1	2.23652061	-5.68380035	0.44431178
C2	2.15747824	-3.08513821	0.28780188
H3	1.15056855	-3.52369274	0.19133888
C4	1.88175720	-1.61911436	0.02713576
O5	2.17381178	-0.95428189	-0.94103138
O6	1.17254803	-1.11241070	1.05147491
C7	0.79874286	0.26490670	0.93292101
H8	0.22272972	0.49121619	1.82916953
H9	1.69103211	0.89237623	0.87791511
H10	0.19240620	0.41661158	0.03763025
C11	3.20136940	-5.16366887	0.39170323
H12	3.93185152	-5.92476491	0.10801667
C13	3.49406397	-4.54970045	1.76986460
H14	4.49381631	-4.10129360	1.74826961
C15	2.48724078	-3.35165304	1.81516121
C16	3.06781030	-2.21092613	2.65300903
O17	4.05168588	-1.55306309	2.38056257
C18	1.21175408	-3.72523496	2.58169158
O19	0.35186832	-4.47902612	2.18291836
C20	2.26819126	-2.09130288	3.90230506
C21	1.20277025	-2.98709995	3.87200969
C22	0.31170983	-3.09070072	4.93402595
C23	0.52695873	-2.26663785	6.03592539
C24	1.60249940	-1.36653848	6.06873565
C25	2.48872512	-1.26559644	4.99922279
H26	-0.51637820	-3.79250137	4.89631785
H27	-0.14388335	-2.32079941	6.88878516
H28	1.74282585	-0.74168069	6.94638147
H29	3.32464232	-0.57200544	5.01186220
C30	3.45164181	-7.08713455	5.26706851
C31	4.33725255	-6.01633568	5.17422925
C32	4.33989400	-5.21500225	4.03655982
C33	3.46326144	-5.46470795	2.97528820
C34	2.57781961	-6.54187239	3.08050999
C35	2.57362374	-7.34617721	4.21756996
H36	3.44751286	-7.71795906	6.15175416
H37	5.02765092	-5.80628004	5.98665833
H38	5.03502470	-4.37951374	3.96577260
H39	1.87927929	-6.75357912	2.27630885
H40	1.88098062	-8.18082893	4.28111629
C41	2.99462893	-4.02029640	-0.61586349
H42	2.20505183	-4.92989838	-1.64464574
C43	2.30517191	-6.25171910	-3.21987392
O44	1.70724225	-5.79640098	-2.16008540
O45	3.36873608	-5.83240006	-3.67968256
N46	4.29860103	-3.45441990	-1.21570795
C47	5.27350137	-4.57048631	-1.49206468
H48	5.69744837	-4.86505141	-0.52831595
H49	4.70641661	-5.39621869	-1.92144586
C50	3.93733815	-2.86065294	-2.56769315
H51	3.62214585	-3.71208990	-3.17169039
H52	3.09575689	-2.19468040	-2.40322191
C53	5.04111915	-2.40039791	-0.43197899
H54	4.39687117	-1.52256142	-0.39800471
H55	5.18151431	-2.75503792	0.58551955
C56	5.16521371	-2.13809384	-3.15810978
H57	5.09190188	-1.05396292	-3.02204253
H58	5.22866377	-2.33515656	-4.23294356
C59	6.36412403	-4.06414828	-2.46408033
H60	7.34297632	-4.43956952	-2.14974760
H61	6.16889824	-4.43463235	-3.47551871
N62	6.39725608	-2.59948499	-2.50674381

C63	6.39215588	-2.09978082	-1.13097472
H64	7.22114082	-2.56961510	-0.59156137
H65	6.57087259	-1.02045526	-1.13686518
C66	0.25490270	-9.45778996	-5.19894013
C67	-0.27925143	-8.91862815	-4.02924237
C68	0.38153388	-7.88402279	-3.37385633
C69	1.58099240	-7.38843303	-3.88827283
C70	2.10391854	-7.91417466	-5.07074461
C71	1.44598332	-8.95323501	-5.72136906
H72	-0.25984725	-10.26869914	-5.70735716
H73	-1.21304721	-9.30463591	-3.62958630
H74	-0.02055816	-7.45260217	-2.46354655
H75	3.03021104	-7.50157917	-5.45887151
H76	1.85829422	-9.37028629	-6.63693855

van der Waals complex of Int3-5 with $PhCO2^-$
E(M06-2X/6-31G(d)(THF)) = -1876.185345028
E(M06-2X/6-311+G(d,p)) = -1876.670926275
G(M06-2X/6-311+G(d,p)(THF)) = -1876.13016

H1	1.91131405	-5.64701426	-0.32312927
C2	2.04857143	-3.03812235	-0.00391537
H3	1.02038310	-3.35706288	-0.24204894
C4	1.94164582	-1.52531551	-0.04921295
O5	2.40548611	-0.76667936	-0.86664429
O6	1.17320021	-1.12336930	0.96978931
C7	0.93747193	0.29074071	1.05464264
H8	0.30246364	0.42883828	1.92812353
H9	1.88574474	0.81840765	1.17495034
H10	0.43379892	0.63984058	0.15131275
C11	2.91794416	-5.22526979	-0.22615875
H12	3.59918389	-5.98009079	-0.61859655
C13	3.18434148	-4.85607105	1.23324109
H14	4.22061734	-4.51193440	1.33272812
C15	2.27642118	-3.58607284	1.43376064
C16	2.90637987	-2.65334081	2.47296730
O17	3.90255655	-1.98182882	2.30515438
C18	0.94496238	-3.98393429	2.09454063
O19	0.02577172	-4.52607788	1.52564134
C20	2.12618581	-2.77344904	3.73037959
C21	0.99910847	-3.56707786	3.51922875
C22	0.11299144	-3.85512171	4.55078891
C23	0.39618099	-3.32421942	5.80723458
C24	1.52780590	-2.52224133	6.01895553
C25	2.40921392	-2.23336044	4.98035216
H26	-0.76183845	-4.47433559	4.37525752
H27	-0.26883497	-3.53100768	6.64109974
H28	1.71565868	-2.12302575	7.01172422
H29	3.28918287	-1.61464971	5.13188186
C30	2.58825353	-7.79853543	4.33255431
C31	3.50969267	-6.76639868	4.49619040
C32	3.70608753	-5.84594336	3.47251380
C33	2.98488439	-5.93461473	2.27667337
C34	2.06651662	-6.97507774	2.11979369
C35	1.87292009	-7.90179416	3.14273728
H36	2.43213229	-8.52166007	5.12827829
H37	4.07694436	-6.68004878	5.41876787
H38	4.42892285	-5.04119053	3.60242304
H39	1.49483954	-7.06943375	1.20025273
H40	1.15718488	-8.70788678	3.00785357
C41	2.87130712	-3.89858395	-1.00592000
H42	2.30097645	-4.02659249	-1.93112855
C43	2.47959161	-5.32178155	-4.06076407
O44	1.67736343	-4.36006858	-4.13462438
O45	3.65261441	-5.37359113	-4.51449876
N46	4.22895787	-3.38047696	-1.51724398
C47	5.12554619	-4.53941547	-1.91028147
H48	5.48735306	-4.98492760	-0.97960227
H49	4.52587328	-5.25230838	-2.47909066
C50	3.98159238	-2.57472410	-2.79395619
H51	3.66208799	-3.29428274	-3.55166772
H52	3.17241756	-1.87955260	-2.58413172
C53	5.00881408	-2.52211871	-0.55149652
H54	4.45636101	-1.59225503	-0.43341637
H55	5.05001799	-3.02401670	0.41238685
C56	5.29265363	-1.86532790	-3.18187584
H57	5.29595386	-0.82247321	-2.84870332
H58	5.39314588	-1.87292001	-4.27092378
C59	6.27417300	-3.98991157	-2.78282490
H60	7.20373574	-4.50967024	-2.53175584
H61	6.04708747	-4.17589503	-3.83678274
N62	6.44329775	-2.54769490	-2.57777199
C63	6.42344799	-2.28417447	-1.13966305
H64	7.15984495	-2.93563013	-0.65857469
H65	6.72067262	-1.24870671	-0.94992257
C66	1.27416422	-8.57030951	-1.46094635
C67	0.38430204	-7.53361419	-1.74577044
C68	0.76289789	-6.51059732	-2.61341789
C69	2.02747810	-6.51268393	-3.20588717
C70	2.90181822	-7.56753687	-2.93592660
C71	2.53252576	-8.58902512	-2.06242448
H72	0.98506788	-9.36523972	-0.77764810
H73	-0.60063941	-7.52149183	-1.28540816
H74	0.09454513	-5.68408433	-2.83792786
H75	3.87564865	-7.56241842	-3.41825912
H76	3.22290466	-9.40135657	-1.84921152

TS-deprot-$PhCO_2^-$
E(M06-2X/6-31G(d)(THF)) = -1876.162203049
E(M06-2X/6-311+G(d,p)) = -1876.65293908
G(M06-2X/6-311+G(d,p)(THF)) = -1876.11111

C1	2.67021859	-2.79140338	-0.04817756
H2	1.71869103	-3.15477204	-0.91020090
C3	2.44148536	-1.36023041	-0.20597691
O4	2.77694988	-0.69088585	-1.17518037
O5	1.74342086	-0.84478558	0.81619354
C6	1.10157300	0.40475897	0.56806298
H7	0.57309086	0.64909449	1.48947024
H8	1.83756646	1.17642852	0.33099661
H9	0.39552230	0.28494402	-0.25522922
C10	3.42392565	-4.66201928	1.31691268
H11	4.34120920	-4.23824277	1.75129935
C12	2.39536955	-3.49121569	1.30527208
C13	2.52836026	-2.63099076	2.57158612
O14	3.56886502	-2.18458893	3.01529846
C15	0.94215249	-3.99506349	1.38497110
O16	0.46481790	-4.86179324	0.68963211
C17	1.19304935	-2.52753277	3.21390173
C18	0.26867915	-3.29843493	2.51365161
C19	-1.05877918	-3.38025722	2.91757740
C20	-1.43425716	-2.65767746	4.04606463

C21	-0.50261355	-1.88327812	4.75447876
C22	0.82685264	-1.81106143	4.34887205
H23	-1.76658298	-3.98577705	2.35962693
H24	-2.46454982	-2.69078551	4.38932305
H25	-0.82979857	-1.33357560	5.63270876
H26	1.55819456	-1.21660914	4.88921376
C27	2.35008339	-7.96627065	3.87834232
C28	2.96103800	-6.82782158	4.39854669
C29	3.31120765	-5.78027044	3.55225178
C30	3.05205982	-5.84803833	2.17887120
C31	2.43368729	-6.99111094	1.66867019
C32	2.08952376	-8.04419571	2.51275591
H33	2.07702759	-8.78802278	4.53450556
H34	3.16783841	-6.75637821	5.46286205
H35	3.79484268	-4.89350519	3.95951549
H36	2.20080088	-7.05753000	0.61019823
H37	1.60784927	-8.92638748	2.10049707
C38	3.77678257	-3.50949742	-0.78330806
H39	3.58902498	-3.53710485	-1.86050504
C40	-0.24040868	-3.02683715	-1.46942525
O41	0.89219014	-3.52772926	-1.84077884
O42	-0.37475109	-2.12162912	-0.64051173
C43	3.66656978	-4.91436092	-0.17278193
H44	4.49262813	-5.59894728	-0.37160513
H45	2.76351640	-5.33957645	-0.62608346
N46	5.22609016	-2.86934850	-0.72892138
C47	6.31179414	-3.91057404	-0.66894732
H48	6.26851369	-4.36815353	0.32295156
H49	6.08313268	-4.66898243	-1.42043777
C50	5.43714254	-2.09147709	-2.01289716
H51	5.49648882	-2.83613271	-2.81203012
H52	4.54827873	-1.47583854	-2.15597244
C53	5.41967183	-1.90101487	0.41518032
H54	4.79054853	-1.03995259	0.19079598
H55	5.05944909	-2.35367235	1.33838873
C56	7.67953400	-3.23083175	-0.94112782
H57	8.42566052	-3.61542899	-0.23979365
H58	8.02679559	-3.45538726	-1.95418043
C59	6.73726248	-1.26383437	-1.88225158
H60	7.29513124	-1.29989475	-2.82247250
H61	6.50744940	-0.21508039	-1.67157660
C62	6.91136261	-1.50029608	0.47909923
H63	6.98974673	-0.43386950	0.70909676
H64	7.43505612	-2.04883258	1.26842946
N65	7.57756245	-1.77981696	-0.79607081
C66	-3.75476637	-4.78500013	-3.21257627
C67	-2.49682755	-5.28821337	-3.54048732
C68	-1.35292444	-4.71958452	-2.98666026
C69	-1.46268962	-3.64693398	-2.10233240
C70	-2.72250220	-3.14522312	-1.77655201
C71	-3.86679758	-3.71145873	-2.32959107
H72	-4.64795664	-5.23046334	-3.64317064
H73	-2.40980931	-6.12696297	-4.22612840
H74	-0.36480062	-5.10145755	-3.22217976
H75	-2.77846259	-2.31219677	-1.08224285
H76	-4.84729599	-3.31953282	-2.07232557

Int4-5

E(M06-2X/6-31G(d)(THF)) = --1455.51140624

E(M06-2X/6-311+G(d,p)) = -1455.885592099

G(M06-2X/6-311+G(d,p)(THF)) = -1455.44627

H1	2.40210957	-5.51995372	-0.12285289
C2	2.61628719	-2.77982044	0.50892977
C3	1.71632485	-1.74003412	0.20776801
O4	1.46639447	-1.23615861	-0.89442796
O5	1.07969424	-1.28048842	1.34320991
C6	0.10356037	-0.27577092	1.12729015
H7	-0.30554180	-0.03743494	2.11064861
H8	0.55097121	0.61660091	0.68008101
H9	-0.69117286	-0.63437291	0.46726107
C10	3.34194629	-4.99594438	0.07833940
H11	4.15355014	-5.61860023	-0.31603718
C12	3.49759690	-4.74362376	1.58603761
H13	4.52204225	-4.40677417	1.77600463
C14	2.60617382	-3.48171784	1.84226280
C15	3.18032592	-2.69953359	3.02908744
O16	4.28525416	-2.19476350	3.07367206
C17	1.19810825	-3.87030897	2.32940832
O18	0.36106679	-4.46152627	1.68400775
C19	2.19371680	-2.72570364	4.14174921
C20	1.05208358	-3.41877648	3.74428840
C21	-0.00969344	-3.61672067	4.61998410
C22	0.10591797	-3.09826703	5.90725138
C23	1.25561128	-2.40040945	6.30649940
C24	2.31620815	-2.20590518	5.42644474
H25	-0.89404941	-4.16040511	4.30044496
H26	-0.70388519	-3.23506267	6.61873698
H27	1.31464637	-2.01020765	7.31890615
H28	3.21406811	-1.67044659	5.72222933
C29	2.83535438	-7.98305872	4.36738397
C30	3.81475055	-7.02239500	4.60625827
C31	4.02015855	-5.99795930	3.68705616
C32	3.25375734	-5.90902391	2.51964994
C33	2.26857565	-6.87501915	2.29480517
C34	2.06454358	-7.90495138	3.21007194
H35	2.67263391	-8.78714892	5.07973062
H36	4.42091828	-7.07155659	5.50673462
H37	4.78762543	-5.24856861	3.87516827
H38	1.63935952	-6.81677810	1.41153900
H39	1.29449823	-8.64729648	3.01901804
C40	3.18981773	-3.60227849	-0.54348938
H41	2.62782071	-3.59493693	-1.48515021
N42	6.79786058	-2.07200985	-2.07927174
C43	5.68448466	-1.13681958	-2.27961755
H44	5.48845048	-1.06404881	-3.35404082
H45	5.98327007	-0.14455182	-1.92921008
C46	6.93522926	-2.31624640	-0.64023619
H47	7.04145357	-1.34983106	-0.13796769
H48	7.84935794	-2.88869244	-0.45668350
C49	6.46274411	-3.33609488	-2.73919309
H50	6.50805017	-3.19984103	-3.82352660
H51	7.21928947	-4.07777114	-2.46467929
C52	5.04775926	-3.81658683	-2.32063112
H53	4.29710606	-3.63057072	-3.09430220
H54	5.03112667	-4.87968015	-2.07272399
C55	5.70755679	-3.08847580	-0.09085518
H56	5.94286454	-4.14186476	0.08699042
H57	5.30062966	-2.64473097	0.82332099
C58	4.42392934	-1.61180951	-1.51918008
H59	4.24766502	-1.06321890	-0.59195183
H60	3.50712122	-1.55754113	-2.10858931
N61	4.61818414	-3.04693868	-1.11401326

TS-elim-5
E(M06-2X/6-31G(d)(THF)) = -1455.509805005
E(M06-2X/6-311+G(d,p)) = -1455.885407161
G(M06-2X/6-311+G(d,p)(THF)) = -1455.44566

H1	2.44073242	-5.59925444	-0.15631502
C2	2.55536289	-2.83098620	0.45483938
C3	1.70301028	-1.73183560	0.16244242
O4	1.49094788	-1.20713404	-0.93040408
O5	1.08370992	-1.26977136	1.29693167
C6	0.17538499	-0.19604145	1.10089144
H7	-0.22824859	0.03944796	2.08648377
H8	0.68760807	0.67551387	0.68421782
H9	-0.63087753	-0.48597924	0.42202753
C10	3.34615665	-5.01771101	0.04975117
H11	4.19502084	-5.58612675	-0.34733773
C12	3.49325530	-4.74817969	1.55738437
H13	4.51119232	-4.38786773	1.73701856
C14	2.58055873	-3.49633513	1.80619368
C15	3.15784042	-2.67708873	2.96630423
O16	4.24869030	-2.14242139	2.97939679
C17	1.18552278	-3.89444917	2.32062975
O18	0.34379547	-4.49747796	1.69288573
C19	2.19435621	-2.70916952	4.09841982
C20	1.05894564	-3.42911188	3.73247045
C21	0.01817887	-3.63579624	4.63131773
C22	0.14948769	-3.09856581	5.90940725
C23	1.29276888	-2.37287187	6.27664436
C24	2.33193651	-2.16922126	5.37331025
H25	-0.86159250	-4.20062892	4.33662726
H26	-0.64301349	-3.24206781	6.63886867
H27	1.36378285	-1.96816880	7.28265743
H28	3.22476010	-1.61236705	5.64395379
C29	2.90529244	-7.97492683	4.36864415
C30	3.88204434	-7.00620972	4.58472053
C31	4.06301344	-5.98491864	3.65691242
C32	3.27489326	-5.90804708	2.50332046
C33	2.29277615	-6.88218371	2.30111991
C34	2.11269950	-7.90855397	3.22527736
H35	2.76183613	-8.77665189	5.08778100
H36	4.50539799	-7.04684342	5.47385932
H37	4.82881838	-5.22947279	3.82687879
H38	1.64839690	-6.83328480	1.42813605
H39	1.34505817	-8.65774888	3.05184649
C40	3.12660374	-3.63860161	-0.55788738
H41	2.67770143	-3.59802465	-1.55347795
N42	6.90857425	-2.01304243	-2.03033893
C43	5.79678009	-1.06952953	-2.20087358
H44	5.63336357	-0.92587878	-3.27376827
H45	6.08453496	-0.10275121	-1.77683503
C46	7.03338067	-2.31854005	-0.60131284
H47	7.16035806	-1.37512150	-0.06123690
H48	7.93479191	-2.91774171	-0.44062270
C49	6.58601353	-3.24832902	-2.75039894
H50	6.61737550	-3.05284240	-3.82643339
H51	7.36061978	-3.98775948	-2.52323656
C52	5.18286217	-3.77734998	-2.33400236
H53	4.43298924	-3.62035944	-3.11584911
H54	5.20457887	-4.84261304	-2.08878440
C55	5.77888643	-3.08173771	-0.09187308
H56	6.00435137	-4.13656557	0.09530761
H57	5.35515320	-2.63670874	0.81480956
C58	4.51400861	-1.60502634	-1.51394366
H59	4.27501325	-1.07612283	-0.58781938
H60	3.63008851	-1.55637768	-2.15375109
N61	4.72900704	-3.03011779	-1.13792497

TS-cycl-7
E(M06-2X/6-31G(d)(THF)) = -1455.436288932
E(M06-2X/6-311+G(d,p)) = -1455.812677492
G(M06-2X/6-311+G(d,p)(THF)) = -1455.36817

C1	-1.50772180	-4.68983846	-3.74579099
C2	-1.44550765	-3.23084309	-5.84804540
C3	-1.00990119	-3.54809667	-4.42849825
O4	-0.98113893	-3.69199298	-6.86769681
H5	-1.20755021	-2.66934667	-3.80932100
O6	-2.46331177	-2.36451007	-5.84336475
C7	-2.97553934	-2.01126040	-7.13230321
H8	-3.78682468	-1.30791251	-6.94708398
H9	-2.19295427	-1.54692897	-7.73695806
H10	-3.34816982	-2.89925274	-7.64970439
C11	0.06213412	-4.65307911	-1.59092554
H12	-0.07091268	-4.24646887	-0.57744051
C13	1.36195240	-8.75445255	-1.02213561
C14	1.98536486	-7.96457500	-1.98289831
C15	1.60761867	-6.63144527	-2.15100967
C16	0.59257807	-6.06790039	-1.37456620
C17	-0.00523261	-6.86712724	-0.39160818
C18	0.36877271	-8.19541303	-0.21752200
H19	1.65769154	-9.79100130	-0.88718791
H20	2.77835626	-8.38009434	-2.59936750
H21	2.11650815	-6.01680804	-2.88951902
H22	-0.77779068	-6.43578436	0.24331408
H23	-0.10887533	-8.79528407	0.55256839
C24	1.08997303	-3.78287438	-2.26070753
C25	3.25095785	-2.72543824	-2.38528458
C26	2.64871954	-2.68832606	-3.64134974
C27	3.27979477	-2.10859343	-4.72284623
C28	4.55765287	-1.56398102	-4.51141679
C29	5.15897474	-1.60117836	-3.25654471
C30	4.50024330	-2.18758353	-2.16304830
H31	2.80452372	-2.07852295	-5.69882734
H32	5.08644480	-1.10444353	-5.34201813
H33	6.14759738	-1.17104193	-3.12314205
H34	4.95362844	-2.22120704	-1.17602225
C35	1.30322674	-3.34464105	-3.54502642
O36	0.58877302	-3.36590630	-4.64379067
C37	2.28275584	-3.41742218	-1.46267111
O38	2.46054003	-3.63310904	-0.27608370
C39	-1.35866527	-4.62966650	-2.24192938
H40	-1.78913939	-3.66225859	-1.95168284
H41	-1.97402270	-5.37232734	-1.72067256
N42	-1.53869298	-6.01651271	-4.46814957
C43	-2.69269358	-6.05346714	-5.45508745
H44	-2.45071740	-5.34654405	-6.24588145
H45	-3.56286482	-5.69361296	-4.90535797
C46	-1.78473577	-7.16711533	-3.52337154
H47	-2.79358742	-7.02867571	-3.12449346
H48	-1.06866778	-7.09474320	-2.70934277
C49	-0.25769474	-6.32660331	-5.22104267

H50	0.02279880	-5.43338241	-5.77719995
H51	0.49146337	-6.51341292	-4.44906355
C52	-0.48626079	-7.54811955	-6.14129279
H53	-0.61961172	-7.23008784	-7.18027303
H54	0.38443301	-8.21013774	-6.10565689
C55	-1.61872546	-8.50287736	-4.28617808
H56	-0.65385073	-8.95847931	-4.04177760
H57	-2.40385380	-9.20237491	-3.98341220
C58	-2.86190614	-7.48434600	-6.02267878
H59	-3.73416197	-7.97946242	-5.58401432
H60	-3.01438975	-7.44128256	-7.10522184
N61	-1.67983239	-8.29823452	-5.73368112

Int2-7
E(M06-2X/6-31G(d)(THF)) = -1455.437002658
E(M06-2X/6-311+G(d,p)) = -1455.813767706
G(M06-2X/6-311+G(d,p)(THF)) = -1455.36946

N1	-1.55389902	-6.08512270	-4.36247726
C2	-2.77564632	-6.34574578	-5.21759473
H3	-2.98361851	-5.46257816	-5.81098096
H4	-3.59709961	-6.49234065	-4.51341341
C5	-1.51442315	-7.23722254	-3.38934076
H6	-2.33255496	-7.08242297	-2.68318614
H7	-0.56595615	-7.17439536	-2.85869112
C8	-0.32413396	-6.18723557	-5.24505284
H9	-0.46760735	-5.46477407	-6.05102224
H10	0.51602825	-5.86203846	-4.63150689
C11	-0.16542418	-7.63768231	-5.75324951
H12	0.12093948	-7.63689289	-6.80931518
H13	0.61787688	-8.16000701	-5.19331325
C14	-1.65211075	-8.57460480	-4.15793671
H15	-0.92921837	-9.29591013	-3.76483990
H16	-2.65193743	-9.00106395	-4.02754971
C17	-2.51564898	-7.57330173	-6.12135889
H18	-3.42152432	-8.18412138	-6.18904247
H19	-2.24993100	-7.25648304	-7.13502791
N20	-1.41735390	-8.38541238	-5.59014316
C21	-1.68206850	-4.75577094	-3.64092775
C22	-2.18330969	-3.31851300	-5.70584965
C23	-1.41440883	-3.56353681	-4.41458653
O24	-1.87516042	-3.71075305	-6.81010657
H25	-1.60822391	-2.71206376	-3.75544062
O26	-3.29166987	-2.60906118	-5.47087701
C27	-4.10023493	-2.33307659	-6.61897677
H28	-4.94142815	-1.74432146	-6.25505381
H29	-3.52733515	-1.77037455	-7.35940544
H30	-4.45105240	-3.26429225	-7.07071044
C31	0.34685149	-4.52217298	-1.86513627
H32	0.37491145	-4.07478667	-0.85997794
C33	2.14829252	-8.44390305	-1.38158335
C34	2.55929655	-7.62736769	-2.43004118
C35	2.01954559	-6.34935279	-2.58040335
C36	1.04998203	-5.86809627	-1.69807302
C37	0.67176798	-6.68932717	-0.62774108
C38	1.20695447	-7.96310211	-0.47105277
H39	2.56919302	-9.43826934	-1.26223071
H40	3.31198607	-7.97768861	-3.13162700
H41	2.36689624	-5.71644170	-3.39305572
H42	-0.05790138	-6.32012309	0.09103456
H43	0.89547764	-8.58117808	0.36687123
C44	1.12454927	-3.59706262	-2.75445850
C45	3.08071987	-2.33564739	-3.38219723
C46	2.18748952	-2.36325923	-4.45219786
C47	2.47806313	-1.73182466	-5.64457299
C48	3.71281619	-1.06830383	-5.74093856
C49	4.60441900	-1.04232124	-4.67233691
C50	4.29100831	-1.68221319	-3.46208107
H51	1.77846863	-1.75016516	-6.47505604
H52	3.97660936	-0.56465840	-6.66693906
H53	5.55131863	-0.52035377	-4.77690896
H54	4.97290342	-1.66788471	-2.61618301
C55	0.97690363	-3.14179791	-4.03595494
O56	0.01789453	-3.24057961	-4.93422461
C57	2.43687570	-3.10631424	-2.26156229
O58	2.91005538	-3.28786647	-1.15523861
C59	-1.17680430	-4.65345203	-2.21337346
H60	-1.62818629	-3.72739309	-1.83436453
H61	-1.58273940	-5.43988312	-1.56631757

TS-prot-PhCO$_2$H
E(M06-2X/6-31G(d)(THF)) = -1876.126432708
E(M06-2X/6-311+G(d,p)) = -1876.62072815
G(M06-2X/6-311+G(d,p)(THF)) = -1876.07518

C1	5.22875129	0.02199075	-13.28343062
C2	5.49886470	0.06334396	-11.78038786
O3	6.07057940	-0.07247345	-14.14681383
H4	4.83737995	0.83795180	-11.37028568
O5	3.92138221	0.07810279	-13.51293098
C6	3.51930939	-0.00946723	-14.88433490
H7	2.43299085	0.05833351	-14.87627046
H8	3.95657939	0.80886398	-15.46015723
H9	3.84050607	-0.96256667	-15.31143186
C10	6.19296047	-0.45513422	-8.62986942
C11	7.06590896	0.59148992	-9.24673206
C12	8.81078423	2.25651948	-9.24824142
C13	8.42738668	1.98480709	-10.56194271
C14	9.02216003	2.62467471	-11.63039531
C15	10.03598312	3.55382150	-11.34283587
C16	10.41855463	3.82406684	-10.03227244
C17	9.79792782	3.17160916	-8.95481057
H18	8.71883639	2.41669954	-12.65185316
H19	10.52967045	4.07215877	-12.15994794
H20	11.20486489	4.54853050	-9.84234170
H21	10.08162213	3.37569199	-7.92600147
C22	7.33981043	0.96412862	-10.52258414
O23	6.84811390	0.59984680	-11.70677001
C24	7.95737423	1.39726411	-8.35792532
O25	7.98303463	1.36091945	-7.14504377
C26	5.01504168	-0.93112305	-9.52669753
H27	4.26452462	-0.13202401	-9.47792426
H28	4.53193695	-1.78310815	-9.03402319
C29	5.12306948	-1.18914380	-11.03328179
H30	3.66080002	-1.32570075	-11.26267713
C31	8.36855558	-3.80845536	-6.94644914
C32	7.06373402	-3.51923578	-6.54779223
C33	6.39929146	-2.42104651	-7.08090119
C34	7.01340887	-1.59526112	-8.03009968
C35	8.32797295	-1.87967135	-8.40074559
C36	9.00025643	-2.98018163	-7.86796240
H37	8.89117401	-4.66445822	-6.52954263

H38	6.56599263	-4.14779929	-5.81468195
H39	5.38317489	-2.19782491	-6.76025755
H40	8.84139866	-1.23153676	-9.10638356
H41	10.02426009	-3.18161399	-8.17049384
H42	5.71553647	0.02157629	-7.76107925
N43	5.82905094	-2.46878780	-11.50970884
C44	7.28772180	-2.29819722	-11.86704527
H45	7.36745688	-1.44871833	-12.54443095
H46	7.80246259	-2.05919663	-10.93319041
C47	5.12255160	-2.99880868	-12.74652688
H48	4.05531351	-2.98814168	-12.52737855
H49	5.35509073	-2.30794064	-13.55694423
C50	5.73016336	-3.55734754	-10.46183173
H51	6.12691711	-3.15726574	-9.53362128
H52	4.66393400	-3.76526700	-10.35366986
C53	5.64049819	-4.42095194	-13.06914705
H54	4.89250439	-5.16904281	-12.78847974
H55	5.82502986	-4.51625276	-14.14357076
C56	7.80998333	-3.60119433	-12.52804077
H57	7.95272226	-3.45166134	-13.60303267
H58	8.77922053	-3.87297862	-12.09905022
C59	6.54663224	-4.78919071	-10.91409519
H60	5.97245752	-5.70107660	-10.72492131
N61	6.87544412	-4.71091711	-12.33741944
H62	7.48133068	-4.85697873	-10.34773097
O63	2.48675325	-1.33247019	-11.15931524
C64	2.02299509	-2.46128380	-10.72669940
O65	2.59679718	-3.55050468	-10.83000251
C66	-1.75960258	-2.26667711	-8.71798392
C67	-1.12723632	-3.49299622	-8.91033521
C68	0.09310912	-3.54709562	-9.57235153
C69	0.68406865	-2.37753215	-10.04757952
C70	0.04881809	-1.15082242	-9.85592385
C71	-1.17117473	-1.09657055	-9.19107123
H72	-2.71114132	-2.22293957	-8.19695931
H73	-1.58535029	-4.40484256	-8.54043699
H74	0.60791319	-4.48927291	-9.72827439
H75	0.52567236	-0.24982861	-10.22532924
H76	-1.66380469	-0.14109662	-9.03916559

van der Waals complex of Int3-7 with $PhCO_2^-$
E(M06-2X/6-31G(d)(THF)) = -1876.152755941
E(M06-2X/6-311+G(d,p)) = -1876.643489035

C1	4.94108786	0.61264969	-12.95940396
C2	5.56833195	0.56299683	-11.56451897
O3	5.55323759	0.68869091	-13.99447965
H4	5.19169471	1.45849995	-11.04942926
O5	3.62078740	0.57294478	-12.84003368
C6	2.86952628	0.54166990	-14.06332319
H7	1.82544131	0.48784623	-13.76196163
H8	3.06592616	1.44402035	-14.64592531
H9	3.14764255	-0.34012673	-14.64463900
C10	6.56410525	0.07651726	-8.51704665
C11	7.55283303	0.83178605	-9.35538571
C12	9.60483086	2.04115710	-9.76169856
C13	8.98024154	1.76298407	-10.97862878
C14	9.53612333	2.15325523	-12.18021727
C15	10.76614940	2.83070988	-12.12915387
C16	11.39104456	3.10624282	-10.91622109
C17	10.80609749	2.71441123	-9.70103349
H18	9.04292448	1.94483017	-13.12492551
H19	11.23765282	3.14723564	-13.05516413
H20	12.34093657	3.63267295	-10.91058993
H21	11.27847963	2.93029620	-8.74666192
C22	7.71446581	1.03893694	-10.68386055
O23	6.96383215	0.70921515	-11.74779727
C24	8.71907150	1.49528399	-8.67892862
O25	8.89372149	1.57330573	-7.48287431
C26	5.20821298	-0.24286893	-9.19481374
H27	4.59115925	0.66030039	-9.12710826
H28	4.67487376	-0.98464394	-8.59709711
C29	5.08996123	-0.61320321	-10.68777216
H30	4.01088020	-0.71439925	-10.86722330
C31	8.21523955	-3.37748367	-6.45593724
C32	6.96061609	-2.86654225	-6.12572525
C33	6.46107456	-1.75636551	-6.79707988
C34	7.19333991	-1.13767893	-7.81787250
C35	8.45893272	-1.64247934	-8.11912920
C36	8.96430624	-2.75730252	-7.44956997
H37	8.60853139	-4.24402170	-5.93249360
H38	6.37261378	-3.32910168	-5.33796769
H39	5.48740139	-1.35757424	-6.51711489
H40	9.07033291	-1.16402203	-8.88063521
H41	9.95177805	-3.13243807	-7.70386419
H42	6.30899855	0.75140317	-7.68729002
N43	5.60134042	-1.99550134	-11.10456208
C44	7.09452413	-2.13429837	-11.24947992
H45	7.37931480	-1.56995315	-12.13829934
H46	7.56316840	-1.68660049	-10.37559868
C47	5.00414566	-2.38066526	-12.44921773
H48	3.92081970	-2.40307455	-12.31083471
H49	5.31188056	-1.61654051	-13.16266792
C50	5.12885538	-3.00489681	-10.06482040
H51	5.74317972	-2.82833895	-9.17966419
H52	4.08204981	-2.76889784	-9.86812917
C53	5.56190129	-3.76303814	-12.86795341
H54	4.72141816	-4.38481219	-13.18695979
H55	6.26797760	-3.66691814	-13.69890994
C56	7.42709225	-3.64287893	-11.38473623
H57	8.20283222	-3.77269804	-12.14513946
H58	7.81539571	-4.03209728	-10.43752672
C59	5.31748359	-4.43156265	-10.61718174
H60	4.35528840	-4.82144036	-10.95677890
N61	6.24337102	-4.41989875	-11.75154968
H62	5.71227464	-5.07916212	-9.82817015
O63	2.33964671	-1.92819209	-10.81720660
C64	1.83384772	-3.03734286	-11.14529102
O65	2.35100224	-3.91002581	-11.88554574
C66	-2.04476131	-3.89837397	-9.43917507
C67	-1.42580411	-4.82997535	-10.27202702
C68	-0.17853632	-4.55085061	-10.82603606
C69	0.46056407	-3.34174031	-10.55339331
C70	-0.16390770	-2.41240570	-9.72019769
C71	-1.41074563	-2.68695495	-9.16461455
H72	-3.01736769	-4.11544319	-9.00536775
H73	-1.91775429	-5.77496254	-10.48749148
H74	0.32334496	-5.26168289	-11.47556086
H75	0.35143215	-1.47783781	-9.52166158
H76	-1.89008921	-1.95774397	-8.51618029

TS-deprot-PhCO$_2^-$
E(M06-2X/6-31G(d)(THF)) = -1876.117100516
E(M06-2X/6-311+G(d,p)) = -1876.606548541
G(M06-2X/6-311+G(d,p)(THF)) = -1876.06928

C1	5.09671915	0.24795352	-13.25760659
C2	5.37207492	0.36827117	-11.80982301
O3	5.90809199	0.25355703	-14.15767698
H4	5.04428845	1.73218451	-11.65033265
O5	3.75913785	0.19205932	-13.48404837
C6	3.38218045	0.16846745	-14.86140925
H7	2.29274424	0.14709523	-14.87296535
H8	3.75313761	1.05831722	-15.37389166
H9	3.78600950	-0.71915148	-15.35722583
C10	5.85608395	0.21815712	-8.59212612
C11	6.83145959	1.09808754	-9.30478893
C12	8.60559366	2.70317219	-9.53634344
C13	8.33184362	2.13394521	-10.78185237
C14	8.98515712	2.54484357	-11.92426301
C15	9.96265646	3.54307505	-11.78018716
C16	10.24409535	4.10459463	-10.53811934
C17	9.55204606	3.69229555	-9.38763706
H18	8.73813203	2.12106830	-12.89310182
H19	10.50161978	3.89221956	-12.65664770
H20	10.99915611	4.88185092	-10.46129492
H21	9.74267326	4.14244870	-8.41735033
C22	7.23227112	1.15443732	-10.59804711
O23	6.79517525	0.46844777	-11.65917914
C24	7.66182009	2.07894713	-8.54664837
O25	7.57969884	2.31744516	-7.35793919
C26	4.61195994	-0.17761824	-9.42327276
H27	4.00103363	0.72589367	-9.48800088
H28	4.01446373	-0.88527063	-8.84197784
C29	4.67702460	-0.61775039	-10.90091811
H30	3.63557041	-0.65384492	-11.23873530
C31	7.66006322	-3.12834451	-6.48201785
C32	6.34098653	-2.74716964	-6.23506737
C33	5.78906996	-1.66597867	-6.91419537
C34	6.53031528	-0.95079057	-7.86458758
C35	7.85804540	-1.32471172	-8.07911303
C36	8.41780579	-2.40764301	-7.39900662
H37	8.09621021	-3.96873474	-5.94945294
H38	5.74628472	-3.28569189	-5.50203535
H39	4.76499087	-1.36410067	-6.69748818
H40	8.46779975	-0.75997766	-8.78052166
H41	9.45410080	-2.67862870	-7.58292299
H42	5.44114761	0.84020480	-7.78514074
N43	5.12017980	-2.12992897	-11.10782391
C44	6.62011946	-2.29327092	-11.17335866
H45	6.94182998	-1.83074328	-12.10808334
H46	7.05158391	-1.73348540	-10.34559327
C47	4.56520672	-2.67437507	-12.40394211
H48	3.47709654	-2.69957660	-12.29488267
H49	4.82513081	-1.98493490	-13.20339811
C50	4.58389434	-3.00846496	-10.00189704
H51	5.12385933	-2.73675865	-9.09438324
H52	3.52462513	-2.76749147	-9.87701681
C53	5.18117430	-4.07468494	-12.66464054
H54	4.40418823	-4.75761547	-13.02049084
H55	5.95308330	-4.01530349	-13.43773916
C56	6.95695304	-3.80080180	-11.10512380
H57	7.77294182	-4.02488289	-11.79799739
H58	7.28668235	-4.07624030	-10.09779723
C59	4.80826231	-4.49582008	-10.36752207
H60	3.87501147	-4.96740187	-10.69039190
N61	5.78583565	-4.61511314	-11.44770534
H62	5.16796419	-5.03642393	-9.48711403
O63	6.06995669	4.47640062	-10.37299884
C64	5.24869286	3.61900271	-10.66671933
O65	5.24229058	2.93504969	-11.77811732
C66	2.10356893	2.61928434	-7.90691492
C67	1.87837381	2.51584638	-9.27964969
C68	2.89554421	2.83674928	-10.17635708
C69	4.13059035	3.28945928	-9.70427936
C70	4.34008788	3.42584693	-8.33099865
C71	3.33542880	3.07205587	-7.43335788
H72	1.31731503	2.35398634	-7.20511808
H73	0.91382840	2.17763004	-9.64905540
H74	2.74743437	2.73386057	-11.24842421
H75	5.30174406	3.79107210	-7.98080571
H76	3.51069550	3.15488449	-6.36403751

Int4-7
E(M06-2X/6-31G(d)(THF)) = -1455.466345613
E(M06-2X/6-311+G(d,p)) = -1455.837597603
G(M06-2X/6-311+G(d,p)(THF)) = -1455.39868

N1	-1.38963742	-6.09444305	-4.30479794
C2	-2.29859788	-6.39651612	-5.46648648
H3	-2.24026200	-5.54814976	-6.14816718
H4	-3.31436494	-6.43700780	-5.06340694
C5	-1.65513060	-7.11744014	-3.24351971
H6	-2.63853280	-6.90037438	-2.81655428
H7	-0.89759102	-6.98731972	-2.46770547
C8	0.02545825	-6.23379038	-4.78266397
H9	0.11245083	-5.58628508	-5.65940318
H10	0.68860902	-5.84876034	-4.00790647
C11	0.29473817	-7.72569792	-5.10294047
H12	0.84651384	-7.81219835	-6.04331212
H13	0.90184844	-8.18717611	-4.31783822
C14	-1.58612189	-8.52972531	-3.88518903
H15	-1.00753720	-9.20077139	-3.24395353
H16	-2.58855748	-8.95408632	-3.99552794
C17	-1.85701365	-7.73196709	-6.10920392
H18	-2.73320763	-8.34674350	-6.33427032
H19	-1.32476055	-7.55489696	-7.04876364
N20	-0.96392001	-8.46889326	-5.20931403
C21	-2.36749798	-3.06056940	-5.65415353
C22	-1.36849476	-3.56898411	-4.79696369
O23	-3.53145324	-3.48423720	-5.73133594
O24	-1.94744350	-2.02043726	-6.43646285
C25	-2.93691913	-1.50431394	-7.31503120
H26	-2.45341509	-0.69645609	-7.86624893
H27	-3.28969575	-2.27333276	-8.00803137
H28	-3.79634257	-1.11851355	-6.76056905
C29	0.18956007	-4.39190909	-1.94382828
H30	0.52235544	-5.44082315	-1.88999998
C31	1.09920608	-3.65088778	-2.87543687
C32	3.14941896	-2.64643304	-3.61239263
C33	2.15780195	-2.39620074	-4.56030283
C34	2.41542496	-1.64909012	-5.69271529
C35	3.71576388	-1.14287812	-5.84888320

C36	4.70676246	-1.39201869	-4.90241131
C37	4.43098456	-2.16008267	-3.76037937
H38	1.63878250	-1.45696237	-6.42683687
H39	3.95332868	-0.54431743	-6.72390705
H40	5.70308017	-0.98548759	-5.05092327
H41	5.19271698	-2.36482392	-3.01310061
C42	0.90176581	-3.05240655	-4.09571446
O43	-0.09938540	-2.97842187	-4.93529827
C44	2.52515604	-3.48519809	-2.52988506
O45	3.10613228	-3.96183414	-1.57000742
C46	-1.29227469	-4.35015446	-2.35358364
H47	-1.66932441	-3.34473045	-2.13528097
H48	-1.84519377	-5.02265325	-1.68781131
C49	0.26755296	-2.85601427	2.10037942
C50	0.29698382	-4.22898868	1.87353851
C51	0.29954876	-4.72099503	0.56974504
C52	0.27477611	-3.85147553	-0.51886380
C53	0.24360780	-2.47547145	-0.28247504
C54	0.24157090	-1.97943986	1.01696659
H55	0.26852588	-2.46977864	3.11573672
H56	0.32326189	-4.91877757	2.71267532
H57	0.33284743	-5.79500037	0.39579362
H58	0.23036198	-1.79036622	-1.12800131
H59	0.22225751	-0.90610068	1.18513114
C60	-1.71665207	-4.57262774	-3.80921020
H61	-2.81054987	-4.61144084	-3.82040615

TS-elim-7
E(M06-2X/6-31G(d)(THF)) = -1455.465764802
E(M06-2X/6-311+G(d,p)) = -1455.838104285
G(M06-2X/6-311+G(d,p)(THF)) = -1455.39833

N1	-1.40161639	-6.02327324	-4.25670253
C2	-2.30822146	-6.31778907	-5.40712967
H3	-2.23430459	-5.48068055	-6.10237417
H4	-3.32878062	-6.33635883	-5.01343326
C5	-1.65248973	-7.03728396	-3.19763884
H6	-2.64440707	-6.84259159	-2.77747869
H7	-0.90630823	-6.89069221	-2.41304753
C8	0.00323890	-6.13966114	-4.73754771
H9	0.09112616	-5.46861737	-5.59739847
H10	0.67096865	-5.77469946	-3.95590866
C11	0.28587821	-7.62265009	-5.10130645
H12	0.79708245	-7.68741659	-6.06632475
H13	0.93290879	-8.08777804	-4.35078127
C14	-1.54756036	-8.45692528	-3.82624497
H15	-0.92697874	-9.10205825	-3.19726240
H16	-2.53603065	-8.91955743	-3.90696786
C17	-1.89492437	-7.66877790	-6.04388663
H18	-2.77696000	-8.29173549	-6.21947195
H19	-1.40221013	-7.51072087	-7.00830599
N20	-0.96337523	-8.38754092	-5.16803078
C21	-2.38863625	-2.95906741	-5.67959898
C22	-1.41616925	-3.39824285	-4.73276129
O23	-3.53203484	-3.41077092	-5.79057867
O24	-1.94912888	-1.94956430	-6.47957138
C25	-2.90650214	-1.47705861	-7.41946386
H26	-2.40782112	-0.68479530	-7.97904282
H27	-3.21979753	-2.27734915	-8.09510532
H28	-3.79120987	-1.08231143	-6.91350424
C29	0.14170289	-4.24920212	-1.90257902
H30	0.46823878	-5.29962637	-1.87429096
C31	1.04807486	-3.49879816	-2.82868816
C32	3.10190726	-2.49253147	-3.56236033
C33	2.10302538	-2.21703365	-4.49553858
C34	2.35612637	-1.45257425	-5.61728944
C35	3.65947764	-0.95453826	-5.77707222
C36	4.65750812	-1.22874634	-4.84513133
C37	4.38634630	-2.01511270	-3.71437395
H38	1.57477191	-1.24274752	-6.34167975
H39	3.89385156	-0.34315253	-6.64407174
H40	5.65602938	-0.82864338	-4.99665495
H41	5.15403648	-2.24111118	-2.97940022
C42	0.84776056	-2.87613892	-4.03255742
O43	-0.16508728	-2.77098937	-4.85901855
C44	2.48141604	-3.34872837	-2.49154275
O45	3.06536971	-3.84952339	-1.54726332
C46	-1.34425298	-4.19751234	-2.29676320
H47	-1.72802929	-3.20779413	-2.02075686
H48	-1.88700431	-4.90711123	-1.66297316
C49	0.28600147	-2.79650244	2.16999620
C50	0.30333218	-4.16480733	1.91440806
C51	0.28364439	-4.62970936	0.60085242
C52	0.24775808	-3.73753232	-0.46896519
C53	0.22842297	-2.36642841	-0.20392201
C54	0.24899122	-1.89733219	1.10543440
H55	0.30492730	-2.43129499	3.19302272
H56	0.33790796	-4.87209970	2.73858566
H57	0.30787795	-5.70017444	0.40404232
H58	0.20631839	-1.66362256	-1.03480733
H59	0.23924731	-0.82747310	1.29612039
C60	-1.77363875	-4.35616356	-3.74939939
H61	-2.85887710	-4.47227059	-3.79497706

PPh_3-catalysed cycloaddition

PPh_3
E(M06-2X/6-31G(d)(THF)) = -1035.964778708
E(M06-2X/6-311+G(d,p)) = -1036.163658638
G(M06-2X/6-311+G(d,p)(THF)) = -1035.93736

C1	-0.95684179	-0.29116356	1.79830988
C2	-1.88012257	-1.25368089	2.21868237
C3	-0.40002179	0.57374335	2.74907576
C4	-2.24173730	-1.34452946	3.56188473
C5	-0.77053067	0.49090951	4.08827187
C6	-1.69184031	-0.47140061	4.49770587
C7	-1.12268920	-1.57269804	-0.74680171
C8	-2.37602657	-1.61784788	-1.36562469
C9	-0.34283496	-2.73558199	-0.70989518
C10	-2.84044802	-2.80405356	-1.93117909
C11	-0.81213950	-3.92313400	-1.26407524
C12	-2.06271737	-3.95845065	-1.87835155
C13	-1.58302619	1.23568446	-0.52666998
C14	-2.70425101	1.66009411	0.19304026
C15	-1.31570339	1.82006831	-1.77108806
C16	-3.54247193	2.64596912	-0.32476351
C17	-2.15983537	2.79588276	-2.29331854
C18	-3.27493556	3.21239905	-1.56865033
P19	-0.41420504	-0.06838223	0.05062908
H20	-0.43867145	1.50753104	-2.33502854

H21	-1.94277912	3.23701196	-3.26212550
H22	-3.93074649	3.97976957	-1.97057937
H23	-4.40945743	2.96933048	0.24478617
H24	-2.92623839	1.21929753	1.16122916
H25	0.33002399	1.31854349	2.43699630
H26	-0.33403569	1.17185130	4.81383035
H27	-1.97642727	-0.54356361	5.54386240
H28	-2.95848097	-2.09813897	3.87689882
H29	-2.32095519	-1.93517655	1.49580309
H30	0.64007927	-2.70978414	-0.24272717
H31	-0.19847932	-4.81879957	-1.22371187
H32	-2.42797103	-4.88206484	-2.31883914
H33	-3.81509445	-2.82659816	-2.41122583
H34	-2.99403702	-0.72467567	-1.40534152

TS-PPh$_3$
E(M06-2X/6-31G(d)(THF)) = -1380.340150276
E(M06-2X/6-311+G(d,p)) = -1380.638937454
G(M06-2X/6-311+G(d,p)(THF)) = -1380.32326

P1	-0.30156201	-2.31872808	-0.25659943
C2	-2.02426094	-0.18623297	-1.35047378
C3	-1.54208465	-0.37057748	-0.13067720
H4	-2.79363361	0.56838557	-1.50188125
H5	-1.68852563	-0.74544323	-2.21769531
C6	-1.47771876	0.14471096	1.13638384
H7	-0.94196147	1.06903932	1.32525177
C8	-1.84707818	-0.68904199	2.24523200
O9	-2.33255951	-1.81426170	2.16375129
O10	-1.55989113	-0.12399417	3.44611076
C11	-1.77562854	-0.97242779	4.56725026
H12	-1.49716904	-0.38773245	5.44468723
H13	-2.82315973	-1.27740407	4.63227861
H14	-1.15087284	-1.86698782	4.49158814
C15	1.89923368	-2.44726045	3.78454348
C16	1.93463043	-1.30721067	2.98712936
C17	1.27759138	-1.29613789	1.75787103
C18	0.59027832	-2.43433425	1.31731934
C19	0.54307482	-3.57100140	2.13116806
C20	1.20266468	-3.57767956	3.35554273
H21	2.40973869	-2.45539533	4.74367427
H22	2.46711042	-0.42137320	3.32100844
H23	1.30460017	-0.40224808	1.13925378
H24	-0.01504182	-4.44625918	1.81038214
H25	1.16962605	-4.46608128	3.98006000
C26	-2.70328304	-6.22524297	-0.75033679
C27	-1.34947446	-6.21243435	-1.08669078
C28	-0.61166187	-5.04108288	-0.95549656
C29	-1.22492017	-3.87512232	-0.47702175
C30	-2.58283486	-3.89008233	-0.14498982
C31	-3.31701162	-5.06683617	-0.28290055
H32	-3.27874486	-7.14090224	-0.85564226
H33	-0.86871620	-7.11602713	-1.45074268
H34	0.44267210	-5.03186848	-1.22020466
H35	-3.05391099	-2.98723100	0.23012187
H36	-4.37209695	-5.07444727	-0.02488184
C37	2.84821754	-2.47386122	-3.64865801
C38	3.27106662	-2.27016546	-2.33848239
C39	2.34604130	-2.23589315	-1.29687100
C40	0.98291393	-2.40515023	-1.55840084
C41	0.56408952	-2.60632181	-2.88221611
C42	1.49063469	-2.64338605	-3.91788848
H43	3.57272881	-2.50595195	-4.45728456
H44	4.32812413	-2.14636330	-2.11984887
H45	2.69290803	-2.09369983	-0.27779602
H46	-0.49053712	-2.76079904	-3.10188405
H47	1.15276178	-2.80895326	-4.93694086

PPh$_3$-allene adduct
E(M06-2X/6-31G(d)(THF)) = -1380.365569941
E(M06-2X/6-311+G(d,p)) = -1380.663126974
G(M06-2X/6-311+G(d,p)(THF)) = -1380.34542

P1	-0.13675927	-2.38353196	-0.03028893
C2	-0.77509024	-0.01564718	-1.15675955
C3	-1.01119005	-0.78433315	-0.05802881
H4	-1.25172566	0.95874574	-1.23042826
H5	-0.12626663	-0.30937798	-1.96954007
C6	-1.79123515	-0.43308500	1.08229677
H7	-2.18738158	0.57462935	1.13361547
C8	-1.98204665	-1.29643012	2.17016768
O9	-1.59988178	-2.47868657	2.25605201
O10	-2.67067546	-0.72516785	3.21106240
C11	-2.87119050	-1.57623124	4.32806582
H12	-3.42122503	-0.98531105	5.06239146
H13	-3.44886252	-2.46336188	4.05273060
H14	-1.91721471	-1.90536632	4.74938557
C15	2.82430476	-2.49100157	3.46605783
C16	2.50803969	-1.28882770	2.83932724
C17	1.58699250	-1.26679321	1.79432860
C18	0.98882101	-2.45965885	1.38186558
C19	1.29265948	-3.66461101	2.01596232
C20	2.21481140	-3.67626861	3.05666265
H21	3.54164140	-2.50472573	4.28171068
H22	2.97285427	-0.36315978	3.16566540
H23	1.33145210	-0.32824974	1.30755924
H24	0.79288137	-4.58279837	1.72003463
H25	2.45051834	-4.61124126	3.55653741
C26	-2.84935523	-6.02692567	-0.67909355
C27	-1.46734916	-6.16253889	-0.78717213
C28	-0.64399215	-5.05932213	-0.58017238
C29	-1.20849131	-3.82804729	-0.23228449
C30	-2.59395894	-3.69131674	-0.12290841
C31	-3.41058617	-4.79238153	-0.35713345
H32	-3.49200183	-6.88558457	-0.85212140
H33	-1.02927103	-7.12204682	-1.04559113
H34	0.43247944	-5.15850687	-0.70276876
H35	-3.02000662	-2.72839966	0.14367362
H36	-4.48872064	-4.68695243	-0.28118394
C37	2.51155549	-2.62138589	-3.80479343
C38	3.09763153	-2.34096617	-2.57440580
C39	2.31319802	-2.26923520	-1.42461081
C40	0.93507859	-2.48346623	-1.50395406
C41	0.35043623	-2.77286969	-2.74494094
C42	1.13599171	-2.83621737	-3.88957717
H43	3.12575295	-2.67732270	-4.69908254
H44	4.16920952	-2.17831914	-2.50420645
H45	2.77981885	-2.05085972	-0.46883720
H46	-0.72084034	-2.94522902	-2.81542872
H47	0.67496709	-3.05665979	-4.84793377

TS-add-γ
E(M06-2X/6-31G(d)(THF)) = -2146.230921681
E(M06-2X/6-311+G(d,p)) = -2146.72239973
G(M06-2X/6-311+G(d,p)(THF)) = -2146.20804

P1	-0.21281787	-2.48154515	-0.47229481
C2	-1.42385237	-1.12055986	-0.35310246
C3	-1.69186745	1.26975519	-2.36106652
C4	-1.50723562	-0.32295400	0.79067109
H5	-2.16896458	0.53545989	0.76205134
C6	-0.94865881	-0.67652489	2.06776903
O7	-0.50084008	-1.77648606	2.38537270
O8	-0.97381958	0.35676401	2.94186042
C9	-0.40656845	0.08066858	4.21634484
H10	-0.48454767	1.00717962	4.78553671
H11	-0.95059861	-0.72284594	4.72031061
H12	0.64057606	-0.21602412	4.11385692
C13	3.78275432	-2.10241228	1.77258279
C14	3.17703576	-0.96669711	1.24184849
C15	1.96013883	-1.06535292	0.57369601
C16	1.34793586	-2.31225892	0.43814184
C17	1.94828262	-3.45271013	0.97653751
C18	3.16683820	-3.34398423	1.63894769
H19	4.73204804	-2.01977759	2.29423948
H20	3.64983876	0.00521498	1.34861755
H21	1.50139095	-0.17686932	0.15762585
H22	1.46595048	-4.42241192	0.90027109
H23	3.63014349	-4.23219700	2.05859259
C24	-2.33080531	-6.34290786	0.82955073
C25	-1.25757600	-6.41366667	-0.05658588
C26	-0.61371542	-5.24863489	-0.46155479
C27	-1.04314924	-4.01025103	0.03019240
C28	-2.11877838	-3.93992175	0.91917669
C29	-2.76037533	-5.10958296	1.31433936
H30	-2.83650852	-7.25275566	1.14050045
H31	-0.92480184	-7.37424489	-0.43857488
H32	0.21321004	-5.30403577	-1.16556088
H33	-2.44266153	-2.97668832	1.30053743
H34	-3.59837659	-5.05523012	2.00284042
C35	1.02368230	-3.03832607	-4.87449668
C36	1.85711462	-2.35377196	-3.99486611
C37	1.47964838	-2.17398934	-2.66727984
C38	0.26493471	-2.69725356	-2.21174615
C39	-0.57072324	-3.38861368	-3.09993531
C40	-0.19367461	-3.54876025	-4.42864807
H41	1.32030622	-3.17206459	-5.91128464
H42	2.80408429	-1.94925023	-4.34073820
H43	2.12946193	-1.62391596	-1.99394736
H44	-1.51192238	-3.80704892	-2.75272082
H45	-0.84882057	-4.07912996	-5.11325752
H46	-2.74140937	1.53715582	-2.22906490
C47	-2.21776374	-0.94519223	-1.47614586
H48	-3.18542537	-0.47340600	-1.33724729
H49	-2.07017706	-1.49751255	-2.39371411
C50	-0.90118658	1.84199021	-1.38940791
C51	-0.43869148	3.09753060	0.59817268
C52	0.76534539	2.56011411	0.14776316
C53	1.95728489	2.80238858	0.80771606
C54	1.91391451	3.60977036	1.94949796
C55	0.70655266	4.14706357	2.40185082
C56	-0.49314301	3.89508409	1.72731701
H57	2.88957342	2.38519434	0.43553359
H58	2.83004216	3.82756914	2.49173508
H59	0.70301709	4.77200435	3.29058128
H60	-1.43806726	4.30527483	2.07192984
C61	0.52139250	1.72949106	-1.08013445
O62	1.39948979	1.06738804	-1.62896186
C63	-1.53501247	2.64402609	-0.32212482
O64	-2.72298246	2.90156956	-0.19646680
C65	-1.03661977	-0.05614223	-6.37363321
C66	-2.32308675	0.13600735	-5.87124036
C67	-2.49631414	0.55115868	-4.55799429
C68	-1.39481809	0.77761165	-3.71422189
C69	-0.10658403	0.57491509	-4.22886426
C70	0.06257077	0.16698811	-5.54810375
H71	-0.89324217	-0.37319448	-7.40304263
H72	-3.18927027	-0.02878002	-6.50627287
H73	-3.49996481	0.71211208	-4.16899063
H74	0.75312149	0.73158337	-3.58869012
H75	1.06799765	0.01895795	-5.93375369

TS-add-α
E(M06-2X/6-31G(d)(THF)) = -2146.234897836
E(M06-2X/6-311+G(d,p)) = -2146.723104335
G(M06-2X/6-311+G(d,p)(THF)) = -2146.20812

C1	0.19508765	-2.60373744	-1.296267621
H2	0.59659274	-3.50902414	0.587838548
C3	0.90992871	-2.78184932	-0.149411693
H4	1.74658780	-2.14075485	0.113210012
C5	0.33935606	-5.41814635	-2.290412743
H6	-0.53447852	-5.97758230	-1.953545813
C7	0.25340274	-4.98033844	-6.551722687
C8	1.46360561	-4.83071292	-5.879567792
C9	1.52962868	-4.95686277	-4.491240020
C10	0.37093568	-5.23468253	-3.753762582
C11	-0.84520631	-5.39135479	-4.443548939
C12	-0.90582306	-5.26355504	-5.824110401
H13	0.21064006	-4.88534687	-7.633746639
H14	2.37211086	-4.61769215	-6.437923406
H15	2.47276311	-4.82166484	-3.973892444
H16	-1.74909602	-5.58665372	-3.868979434
H17	-1.85643890	-5.38569209	-6.336597820
C18	1.36931060	-5.43933577	-1.367901067
C19	2.37082008	-5.90536323	0.761523678
C20	3.29523976	-5.18518331	0.011581682
C21	4.54207626	-4.86396998	0.519578991
C22	4.84161002	-5.28455086	1.819336832
C23	3.91274350	-6.00834335	2.572919250
C24	2.65779829	-6.33195716	2.047319006
H25	5.25448799	-4.30751873	-0.083657339
H26	5.81079694	-5.05115470	2.252283382
H27	4.17464426	-6.32502778	3.578865011
H28	1.92775428	-6.89906646	2.618398380
C29	2.72542553	-4.87210601	-1.348013680
O30	3.37187690	-4.29114323	-2.211842070
C31	1.13230488	-6.09640796	-0.064784621
O32	0.14656186	-6.72281999	0.291269836
C33	-0.87013099	-3.39154524	-1.824829486
H34	-1.27050058	-3.14214513	-2.798008180
C35	-1.82096809	-4.15912706	-1.048290160
O36	-2.81230830	-4.70480574	-1.520132919

O37	-1.50817014	-4.26709873	0.262620869
C38	-2.34985677	-5.13783477	1.014087175
H39	-1.93763731	-5.15011839	2.022935760
H40	-3.37734700	-4.76516041	1.022269232
H41	-2.32608971	-6.14171758	0.587794700
P42	0.78148314	-1.21318327	-2.312062310
C43	-1.84097650	-0.37620113	-6.003522839
C44	-1.20864460	-1.61090349	-5.867801142
C45	-0.42358533	-1.87758345	-4.751664710
C46	-0.26582229	-0.89891676	-3.759685978
C47	-0.90761853	0.33736941	-3.895188397
C48	-1.69183869	0.59381146	-5.016969200
H49	-2.45488076	-0.17279499	-6.876576596
H50	-1.32601275	-2.37883176	-6.626243945
H51	0.05183947	-2.85052788	-4.655468116
H52	-0.80483310	1.10131117	-3.131154817
H53	-2.18867850	1.55464405	-5.114841953
C54	0.50275516	2.64763478	0.158438854
C55	1.39364552	2.56883315	-0.911616893
C56	1.50313934	1.38857510	-1.639085286
C57	0.72083108	0.28108480	-1.289233648
C58	-0.17510467	0.36141712	-0.219247757
C59	-0.28072710	1.54824750	0.501667822
H60	0.42107405	3.56986885	0.727000129
H61	2.00533136	3.42553222	-1.179203483
H62	2.19639105	1.32838526	-2.474649573
H63	-0.77747936	-0.50042978	0.052845571
H64	-0.97413513	1.60999534	1.335285765
C65	5.17215904	-1.76125118	-3.558309799
C66	4.84855213	-1.35574783	-2.265237317
C67	3.52005222	-1.16852154	-1.906240523
C68	2.50430250	-1.38693163	-2.846421438
C69	2.83230366	-1.77619147	-4.146932964
C70	4.16591839	-1.96517548	-4.496952439
H71	6.21183280	-1.91143375	-3.836070813
H72	5.63247362	-1.18220924	-1.533701144
H73	3.28043392	-0.83564168	-0.899936000
H74	2.06063058	-1.93409197	-4.892741617
H75	4.41609538	-2.27024175	-5.509108253

Int1
E(M06-2X/6-31G(d)(THF)) = -2146.272918734
E(M06-2X/6-311+G(d,p)) = -2146.758244542
G(M06-2X/6-311+G(d,p)(THF)) = -2146.24459

P1	-0.11820414	-2.22286701	-0.36509484
C2	-1.25306779	-0.79478410	-0.51850580
C3	-1.98691220	1.11045407	-2.09975628
C4	-1.68664658	-0.12352767	0.55955400
H5	-2.27670158	0.78097888	0.42985407
C6	-1.42156216	-0.51806097	1.96760064
O7	-1.18489424	-1.65235773	2.33171565
O8	-1.49586903	0.52492322	2.78745911
C9	-1.22145254	0.24652794	4.16599054
H10	-1.29517679	1.20323012	4.68028108
H11	-1.95184252	-0.46361906	4.56007089
H12	-0.21821659	-0.17306804	4.26810709
C13	3.21842971	-1.48677245	2.70197260
C14	2.89389778	-0.50178463	1.77300245
C15	1.88593229	-0.72049306	0.83698538
C16	1.20235151	-1.94106977	0.84414074
C17	1.51493444	-2.92832298	1.78298281
C18	2.52997957	-2.69889241	2.70497950
H19	4.00468501	-1.30888885	3.43039705
H20	3.42074186	0.44792368	1.77314497
H21	1.64630115	0.04244263	0.09778232
H22	0.95407096	-3.85725167	1.81868529
H23	2.77248871	-3.46365646	3.43709826
C24	-2.44056161	-6.07616592	0.55052440
C25	-1.11637814	-6.13984134	0.11790984
C26	-0.41912403	-4.96897299	-0.15803259
C27	-1.04611759	-3.72838645	0.01893813
C28	-2.37150909	-3.66603737	0.45146796
C29	-3.06723333	-4.84318109	0.71173653
H30	-2.98632777	-6.99224065	0.75832800
H31	-0.62824541	-7.10103907	-0.01305042
H32	0.60661381	-5.02150679	-0.51717726
H33	-2.85604777	-2.70455050	0.58952435
H34	-4.10007304	-4.79409067	1.04353026
C35	1.69663990	-2.99164305	-4.51578572
C36	2.39629066	-2.22175728	-3.59119244
C37	1.86478275	-1.98932743	-2.32611562
C38	0.62727781	-2.54071409	-1.98571079
C39	-0.07977551	-3.31193051	-2.91870178
C40	0.45650886	-3.53374536	-4.18135775
H41	2.11617178	-3.16809771	-5.50232047
H42	3.35785225	-1.79139524	-3.85392718
H43	2.40290133	-1.36243903	-1.62472610
H44	-1.04705352	-3.73721726	-2.66355527
H45	-0.09430311	-4.12919137	-4.90341219
H46	-2.96363736	1.30425140	-1.63474552
C47	-1.61655482	-0.38679957	-1.92714955
H48	-2.45666502	-1.01021650	-2.26728733
H49	-0.77489714	-0.60055935	-2.58622191
C50	-0.98922102	2.00742600	-1.43474415
C51	-0.06414236	3.71105933	-0.07551254
C52	0.97923614	2.88281447	-0.49247774
C53	2.28548282	3.13012821	-0.12622974
C54	2.53768538	4.24967271	0.68586880
C55	1.49818333	5.07502719	1.10178544
C56	0.17006121	4.80967199	0.72259716
H57	3.08894996	2.48068467	-0.46726505
H58	3.55618699	4.47810127	0.98959923
H59	1.71795242	5.93665949	1.72674785
H60	-0.64980827	5.44617884	1.04505554
C61	0.39423040	1.77213871	-1.33840722
O62	1.08108339	0.81316182	-1.77305629
C63	-1.35735737	3.14062983	-0.63624927
O64	-2.48331655	3.57008110	-0.36674505
C65	-2.36737887	1.83809026	-6.35054721
C66	-3.49192154	1.89125057	-5.53324738
C67	-3.37382541	1.65880175	-4.16189687
C68	-2.13394971	1.37685148	-3.59184407
C69	-1.00557137	1.33076379	-4.42005710
C70	-1.12181196	1.55651984	-5.78668605
H71	-2.45655970	2.02006470	-7.41797295
H72	-4.46536673	2.11796756	-5.96028068
H73	-4.25465686	1.70969478	-3.52508418
H74	-0.03375245	1.12297880	-3.97457988
H75	-0.23637555	1.52004618	-6.41606231

Int1-α
E(M06-2X/6-31G(d)(THF)) = -2146.267710102
E(M06-2X/6-311+G(d,p)) = -2146.752048016
G(M06-2X/6-311+G(d,p)(THF)) = -2146.23931

C1	0.75906800	-2.62267944	-1.46855823
H2	1.45501486	-3.30141489	0.40383465
C3	1.51525237	-2.53977711	-0.36451506
H4	2.20635027	-1.71555016	-0.19455438
C5	0.45129635	-5.12345174	-2.12481981
H6	-0.39725670	-5.82042709	-2.09014147
C7	1.60862367	-5.01080824	-6.29506431
C8	2.54562666	-4.69578074	-5.31393543
C9	2.20488299	-4.75637754	-3.96130558
C10	0.91056647	-5.10755139	-3.57222308
C11	-0.02410775	-5.42387094	-4.56709163
C12	0.31997569	-5.38772652	-5.91478057
H13	1.88098754	-4.97590186	-7.34666948
H14	3.55549560	-4.40791291	-5.59614758
H15	2.94578873	-4.54863294	-3.19523537
H16	-1.03342005	-5.70847909	-4.27426481
H17	-0.41654721	-5.65479717	-6.66855887
C18	1.38322019	-5.61840128	-1.06146905
C19	1.90270167	-6.81782306	0.92594044
C20	3.04449567	-6.12586157	0.53247181
C21	4.21769107	-6.20662965	1.25358270
C22	4.22499145	-7.01673446	2.40086846
C23	3.08242706	-7.71015775	2.79294115
C24	1.89401065	-7.61635926	2.05033927
H25	5.10101011	-5.65920086	0.93505404
H26	5.13272512	-7.10570036	2.99225175
H27	3.11394966	-8.33109361	3.68442198
H28	0.99495559	-8.15194011	2.34356196
C29	2.73580967	-5.32919752	-0.72247149
O30	3.60324821	-4.58342082	-1.22436097
C31	0.81796621	-6.49955152	-0.07474132
O32	-0.34078185	-6.92775967	-0.00073365
C33	-0.22762454	-3.72960618	-1.77839965
H34	-0.83479427	-3.45510985	-2.64535815
C35	-1.25916655	-4.00098544	-0.69036446
O36	-2.40512357	-4.28975712	-0.94893196
O37	-0.77147358	-3.95209918	0.55008233
C38	-1.64655385	-4.45804091	1.56707935
H39	-1.10819839	-4.33223171	2.50577388
H40	-2.58222329	-3.89515044	1.57831781
H41	-1.84086947	-5.51387975	1.37021573
P42	0.97441417	-1.26201445	-2.64758492
C43	-2.39757478	-1.36660205	-5.74741581
C44	-1.26179544	-2.15567378	-5.91917082
C45	-0.22690513	-2.10311129	-4.99178497
C46	-0.33941484	-1.25347850	-3.88387502
C47	-1.47935533	-0.46181136	-3.70839844
C48	-2.50655183	-0.52166392	-4.64515501
H49	-3.20232973	-1.41067197	-6.47582929
H50	-1.17593321	-2.81927511	-6.77426953
H51	0.65767097	-2.72129528	-5.13053477
H52	-1.56635202	0.20174318	-2.85241814
H53	-3.39181345	0.09312947	-4.51326474
C54	0.63788793	2.72637135	-0.39049687
C55	1.49953596	2.61688306	-1.47913737
C56	1.62862470	1.40341487	-2.14872232
C57	0.89177910	0.29418018	-1.72200348
C58	0.03181791	0.40241604	-0.62162113
C59	-0.09500379	1.62059234	0.03765361
H60	0.54159777	3.67456666	0.13064902
H61	2.07866779	3.47522129	-1.80661049
H62	2.30995824	1.32252981	-2.99092798
H63	-0.52868820	-0.46194118	-0.27508528
H64	-0.76210136	1.70287475	0.89056586
C65	5.01207486	-1.27962681	-4.81575970
C66	4.86899925	-1.97486376	-3.61784817
C67	3.64138292	-1.99998385	-2.95989993
C68	2.55507876	-1.32132696	-3.51817951
C69	2.69247589	-0.62650801	-4.72840203
C70	3.92394846	-0.60738949	-5.37260644
H71	5.97163773	-1.26807979	-5.32528757
H72	5.70818139	-2.51812417	-3.19351230
H73	3.53223643	-2.60170871	-2.06014484
H74	1.84279612	-0.11234939	-5.17157090
H75	4.03155146	-0.07355020	-6.31229667

TS-cycl-5
E(M06-2X/6-31G(d)(THF)) = -2146.257348693
E(M06-2X/6-311+G(d,p)) = -2146.744112463
G(M06-2X/6-311+G(d,p)(THF)) = -2146.22519

P1	4.85495086	-2.51641201	-0.74098543
H2	2.44272598	-5.23488019	-0.21130152
C3	3.42883375	-3.33342731	-0.15444085
C4	2.37937319	-2.73686767	0.57167422
H5	1.36595935	-3.09356655	0.39508021
C6	2.48135808	-1.34738843	1.09099235
O7	3.46319058	-0.64178745	0.98465738
O8	1.38265973	-0.98740510	1.76011955
C9	1.48063181	0.24671489	2.47771827
H10	0.52927184	0.36292225	2.99523176
H11	2.30591293	0.19167534	3.19206014
H12	1.65202211	1.07639191	1.78837364
C13	3.40699156	-4.81046676	0.09638084
H14	4.20243550	-5.37982117	-0.39075095
C15	3.50136188	-4.90645425	1.65968931
H16	4.46076506	-4.45146149	1.94591403
C17	2.43911672	-4.02419872	2.25576006
C18	2.69623106	-3.17444276	3.42297215
O19	3.75787356	-2.67097536	3.77705312
C20	1.02508519	-4.42629451	2.31321214
O21	0.42196728	-5.14219180	1.52368359
C22	1.38767775	-2.97876640	4.13378505
C23	0.40327754	-3.71832009	3.48180133
C24	-0.90884170	-3.72246058	3.92475143
C25	-1.21128033	-2.96580951	5.06067786
C26	-0.22462801	-2.21950074	5.71193733
C27	1.09557618	-2.21409576	5.25080750
H28	-1.66700248	-4.30282296	3.40634048
H29	-2.22733569	-2.95402114	5.44560799
H30	-0.49104998	-1.64001134	6.59167333
H31	1.87301360	-1.64060848	5.74862104
C32	3.56606149	-8.92630492	3.25182955
C33	4.41102834	-7.94321409	3.76175803
C34	4.37462532	-6.65526674	3.23674809
C35	3.49743684	-6.32342819	2.19911296
C36	2.64883215	-7.31563453	1.69823297

C37	2.68594802	-8.60621396	2.22087839
H38	3.59272177	-9.93440913	3.65605548
H39	5.10093074	-8.17936219	4.56786581
H40	5.03314713	-5.88669950	3.63730109
H41	1.94225411	-7.07483550	0.91001556
H42	2.01959778	-9.36543928	1.81993020
C43	3.57895028	1.01929141	-3.40915283
C44	4.72401944	1.13341177	-2.62440449
C45	5.11847615	0.08236095	-1.80168103
C46	4.36559388	-1.09301232	-1.76601611
C47	3.20601590	-1.20029240	-2.54094375
C48	2.81824670	-0.14651187	-3.36336460
H49	3.27392787	1.84283940	-4.04873503
H50	5.30981532	2.04798337	-2.64392222
H51	5.99673196	0.19237910	-1.17339003
H52	2.60334625	-2.10325664	-2.48821360
H53	1.91724196	-0.23654318	-3.96348727
C54	6.85319363	-5.66910571	-3.45838742
C55	7.19667108	-5.58266646	-2.11045800
C56	6.61738096	-4.60703394	-1.30565240
C57	5.68120331	-3.71609961	-1.84432252
C58	5.33712471	-3.80970828	-3.19423679
C59	5.92588666	-4.78229872	-3.99876626
H60	7.30974163	-6.42820347	-4.08738800
H61	7.91743818	-6.27411994	-1.68362490
H62	6.89422421	-4.54049263	-0.25616513
H63	4.61638552	-3.12059418	-3.62450308
H64	5.65883352	-4.84546424	-5.04977212
C65	8.36978827	-1.29060482	1.98305949
C66	8.54379994	-1.34338168	0.59939347
C67	7.47957495	-1.70182859	-0.22013107
C68	6.22818756	-1.99125366	0.34293105
C69	6.05727463	-1.94039721	1.72489285
C70	7.13201476	-1.59279957	2.54195059
H71	9.20474180	-1.01823168	2.62303129
H72	9.51100202	-1.11521388	0.16038171
H73	7.62605066	-1.76517022	-1.29658572
H74	5.09759625	-2.17164711	2.17664379
H75	6.99113915	-1.56086910	3.61860800

TS-cycl-5-α

E(M06-2X/6-31G(d)(THF)) = -2146.248569457
E(M06-2X/6-311+G(d,p)) = -2146.733695531
G(M06-2X/6-311+G(d,p)(THF)) = -2146.21824

P1	4.97263152	-2.74821326	-0.81888440
H2	2.56714233	-5.49354386	-0.23742869
C3	3.56515835	-3.61364044	-0.25081549
C4	2.45950404	-2.95448945	0.28357131
H5	1.48314763	-3.41179173	0.13643818
C6	3.51073876	-5.07218507	0.12789249
C7	3.47055033	-5.05253788	1.70352677
H8	4.42884177	-4.62467490	2.01715477
C9	2.41746994	-4.09770769	2.18490484
C10	2.68416564	-3.19437133	3.29615713
O11	3.76864571	-2.81221191	3.73308412
C12	0.98848643	-4.38265758	2.17466357
O13	0.37306432	-5.12538661	1.41309142
C14	1.35170139	-2.78185318	3.86433326
C15	0.34794831	-3.49086553	3.20667413
C16	-0.98562512	-3.32244387	3.53423563
C17	-1.29403068	-2.41817139	4.55783743
C18	-0.28785428	-1.70835354	5.21679800
C19	1.05818396	-1.88224738	4.87334537
H20	-1.75923871	-3.88285036	3.01628834
H21	-2.32998122	-2.26620833	4.84873379
H22	-0.55673487	-1.01489239	6.00922270
H23	1.85121036	-1.33825851	5.37955836
C24	3.31603742	-8.97526128	3.50823925
C25	4.21770964	-8.01531096	3.96302852
C26	4.25347572	-6.75866264	3.36695478
C27	3.38940965	-6.43645633	2.31580683
C28	2.48637667	-7.40532508	1.86653261
C29	2.45307550	-8.66523934	2.45997780
H30	3.28617893	-9.95909875	3.96862996
H31	4.89534734	-8.24558662	4.78125750
H32	4.95632955	-6.00665082	3.72045228
H33	1.79299457	-7.16839347	1.06500893
H34	1.74541539	-9.40776881	2.10070429
C35	3.47923584	1.27068961	-2.54074543
C36	4.66470513	1.22626116	-1.81338519
C37	5.11331473	0.02180510	-1.27677197
C38	4.37440675	-1.14965559	-1.46714833
C39	3.17435018	-1.09733862	-2.19151851
C40	2.73368523	0.10792925	-2.72672765
H41	3.13026673	2.21209136	-2.95570928
H42	5.24258669	2.13188398	-1.65349473
H43	6.03178129	0.00626200	-0.69923690
H44	2.57334817	-1.99314810	-2.31752013
H45	1.80142686	0.13888882	-3.28321498
C46	6.67835700	-5.18432806	-4.34782008
C47	7.40441576	-5.10370322	-3.16336495
C48	6.91850972	-4.35690544	-2.09308136
C49	5.70265025	-3.67803343	-2.20872204
C50	4.97460581	-3.76671349	-3.40025405
C51	5.46183291	-4.51582529	-4.46468068
H52	7.05733424	-5.77208578	-5.17907547
H53	8.35071230	-5.62851909	-3.06558606
H54	7.47977269	-4.31836222	-1.16528428
H55	4.02241924	-3.25484014	-3.50089306
H56	4.88849577	-4.58035703	-5.38444313
C57	8.33057618	-1.58827380	2.11060852
C58	8.58845021	-1.57359062	0.73843134
C59	7.59613923	-1.94617687	-0.16089833
C60	6.33705167	-2.34037351	0.31540277
C61	6.07705587	-2.33711410	1.68510793
C62	7.07681587	-1.96416091	2.58122367
H63	9.11087751	-1.29985935	2.80956508
H64	9.56378216	-1.27007204	0.36887990
H65	7.79933447	-1.92079465	-1.22912887
H66	5.10130084	-2.61181108	2.07557296
H67	6.86110802	-1.96957762	3.64586623
C68	4.60089346	-5.99987630	-0.36353243
O69	4.46347990	-6.85825412	-1.20042427
O70	5.75502923	-5.79613176	0.29996318
C71	6.76586848	-6.78104738	0.07736793
H72	7.63730745	-6.44805911	0.64101857
H73	6.41939167	-7.74886960	0.44824129
H74	6.99587537	-6.86442916	-0.98685499
H75	2.44023139	-1.87254031	0.40826635

Int2-5
E(M06-2X/6-31G(d)(THF)) = -2146.276824045
E(M06-2X/6-311+G(d,p)) = -2146.763815734
G(M06-2X/6-311+G(d,p)(THF)) = -2146.24165

P1	4.97487239	-2.43004637	-0.84768133
H2	2.72498451	-5.17731480	-0.36395261
C3	3.62981563	-3.22094943	-0.20520655
C4	2.53998164	-2.61353554	0.64698971
H5	1.56926655	-2.63581341	0.12422321
C6	2.72387639	-1.18089128	1.09147672
O7	3.65938946	-0.46491031	0.82314872
O8	1.68318313	-0.77692910	1.83883764
C9	1.81396003	0.52925313	2.40786824
H10	0.90204711	0.69753222	2.97945809
H11	2.69000084	0.56254573	3.05986757
H12	1.91971700	1.27819462	1.62026605
C13	3.59530445	-4.69928088	0.11047870
H14	4.48172320	-5.26390168	-0.19044917
C15	3.40972086	-4.71706769	1.63848655
H16	4.34104185	-4.32420511	2.06780866
C17	2.35254010	-3.58123436	1.87991967
C18	2.57919910	-2.99132514	3.26913549
O19	3.55672132	-2.36160616	3.61889445
C20	0.93325388	-4.14227565	1.96142605
O21	0.27910625	-4.55497383	1.02846580
C22	1.43411087	-3.38128629	4.13483999
C23	0.48650417	-4.06949943	3.38022745
C24	-0.67748842	-4.56387262	3.95711160
C25	-0.86039555	-4.35051937	5.32077125
C26	0.09341046	-3.65685751	6.08061651
C27	1.25557853	-3.16112002	5.49618204
H28	-1.40840126	-5.09844372	3.35744540
H29	-1.75527597	-4.72550245	5.80948553
H30	-0.08208719	-3.50791911	7.14232362
H31	2.00310890	-2.62397460	6.07296089
C32	2.50653387	-8.41041900	3.68832684
C33	3.32456476	-7.44966090	4.27766281
C34	3.61348658	-6.27119179	3.59642428
C35	3.09198728	-6.02714235	2.32138862
C36	2.26983325	-6.99853095	1.74128674
C37	1.98225403	-8.18077624	2.41904834
H38	2.28034100	-9.33367926	4.21451826
H39	3.74075715	-7.61893425	5.26701732
H40	4.25633916	-5.52266993	4.05708680
H41	1.84268914	-6.83041105	0.75752593
H42	1.34406746	-8.92551103	1.95119100
C43	3.57473823	0.94459100	-3.68851040
C44	4.75123849	1.12192095	-2.96395390
C45	5.19569152	0.13159415	-2.09205321
C46	4.46669607	-1.05053119	-1.94473948
C47	3.27469300	-1.21464827	-2.65772150
C48	2.83309959	-0.22305934	-3.52967973
H49	3.23088577	1.72054351	-4.36707120
H50	5.32248041	2.04003511	-3.07030109
H51	6.09757730	0.29500962	-1.51135239
H52	2.69345510	-2.12152242	-2.50785169
H53	1.90599883	-0.36131077	-4.07941113
C54	7.00508896	-5.69180340	-3.42004609
C55	7.41752770	-5.48033506	-2.10638559
C56	6.83906148	-4.46539826	-1.34865163
C57	5.83264336	-3.66220609	-1.89533963
C58	5.41973193	-3.88417008	-3.21109703
C59	6.00790306	-4.89118546	-3.97204767
H60	7.45921593	-6.48110189	-4.01284896
H61	8.19148097	-6.10522865	-1.66940737
H62	7.16873267	-4.30123589	-0.32567862
H63	4.63771643	-3.26764211	-3.64517011
H64	5.68419590	-5.05263964	-4.99651282
C65	8.58135049	-0.95905349	1.69250589
C66	8.70421275	-1.03989646	0.30520637
C67	7.62146061	-1.45533521	-0.46487136
C68	6.40120840	-1.77945978	0.14211367
C69	6.28947224	-1.70873098	1.53098168
C70	7.37613440	-1.29754527	2.30216981
H71	9.42734049	-0.64021061	2.29527075
H72	9.64578812	-0.78841173	-0.17565869
H73	7.73121518	-1.53846574	-1.54498455
H74	5.35012436	-1.97066365	2.01298557
H75	7.27796205	-1.24375307	3.38293366

Int2-5-α
E(M06-2X/6-31G(d)(THF)) = -2146.283931447
E(M06-2X/6-311+G(d,p)) = -2146.77004922
G(M06-2X/6-311+G(d,p)(THF)) = -2146.24941

P1	5.15251531	-2.64108000	-0.76623396
C2	4.46284436	-3.17269893	0.66509608
C3	3.48154641	-2.41649748	1.51744267
H4	2.89750193	-1.67236593	0.96142989
C5	4.68818543	-4.56037801	1.24744131
C6	3.64969892	-4.66051839	2.38397001
H7	4.14741957	-4.31192615	3.29764603
C8	2.59431086	-3.56529591	2.08076264
C9	1.82449531	-3.15921867	3.33544723
O10	2.32467066	-2.82532486	4.38769641
C11	1.49827309	-3.96418392	1.09867847
O12	1.63836734	-4.40538455	-0.02462731
C13	0.36634953	-3.24578751	3.04598206
C14	0.17906668	-3.71145595	1.74738895
C15	-1.09530511	-3.89561605	1.22229207
C16	-2.18015504	-3.59480332	2.04176720
C17	-1.99089971	-3.12366076	3.35021882
C18	-0.71185692	-2.94325949	3.87059021
H19	-1.22768385	-4.26445680	0.20921884
H20	-3.19166844	-3.72725509	1.66773632
H21	-2.85939384	-2.89965206	3.96341018
H22	-0.55029525	-2.58175026	4.88211195
C23	1.89115282	-8.50526884	3.26110035
C24	2.24679507	-7.63311024	4.28642636
C25	2.82951534	-6.40500016	3.98549449
C26	3.06521235	-6.02691354	2.66020959
C27	2.69893957	-6.90892313	1.63772340
C28	2.11924500	-8.13881955	1.93644937
H29	1.44043679	-9.46650353	3.49239001
H30	2.07554521	-7.91040043	5.32300841
H31	3.11135284	-5.72533197	4.78729855
H32	2.85857373	-6.62991280	0.59971289
H33	1.84409376	-8.81363207	1.13027923
C34	2.32866572	0.54407244	-2.57131997
C35	3.58711375	0.93103892	-2.12187551
C36	4.46426491	-0.01585456	-1.59571342

C37	4.08386485	-1.35739946	-1.51704844
C38	2.80737041	-1.73796055	-1.94944000
C39	1.93795149	-0.79152937	-2.48042472
H40	1.64718042	1.28273154	-2.98431705
H41	3.89002791	1.97290058	-2.17554580
H42	5.43971302	0.29857741	-1.23824277
H43	2.48467021	-2.77032862	-1.84639175
H44	0.95160358	-1.09564169	-2.81906320
C45	5.42917132	-6.20290714	-3.70776141
C46	6.53919673	-5.37737739	-3.55451683
C47	6.49718891	-4.30157938	-2.67047139
C48	5.33600204	-4.03911033	-1.94024549
C49	4.23211896	-4.89169094	-2.07324939
C50	4.27816516	-5.96312132	-2.95949202
H51	5.46518764	-7.04054776	-4.39903728
H52	7.44609677	-5.57286582	-4.11996935
H53	7.37878731	-3.68233679	-2.54468421
H54	3.35155082	-4.72676962	-1.45580180
H55	3.41601239	-6.61688934	-3.05889970
C56	9.34011019	-0.66783534	-0.53928772
C57	8.67850881	-0.70203933	-1.76811236
C58	7.42105369	-1.28836746	-1.86359611
C59	6.81536901	-1.84859380	-0.73088847
C60	7.47545871	-1.79563829	0.49671942
C61	8.73841940	-1.21059388	0.59217674
H62	10.32262366	-0.20948454	-0.46607016
H63	9.14301936	-0.26857225	-2.64956003
H64	6.90398510	-1.30515296	-2.82188747
H65	6.99002869	-2.20625959	1.38118603
H66	9.24662660	-1.17332599	1.55215764
H67	4.56637941	-5.34843141	0.49543828
C68	6.09832432	-4.67651555	1.79759400
O69	6.47048044	-4.37257352	2.91028192
O70	6.94546007	-5.09211670	0.83910749
C71	8.32121750	-5.15391826	1.21597924
H72	8.85076057	-5.53422403	0.34302341
H73	8.68889618	-4.15963031	1.48099561
H74	8.45260315	-5.82437463	2.06845455
H75	3.91579943	-1.90850630	2.39358866

TS-prot-5
E(M06-2X/6-31G(d)(THF)) = -3256.581739014
E(M06-2X/6-311+G(d,p)) = -3257.359908973

P1	4.83326703	-2.25903400	-1.02190206
H2	2.62410514	-5.11780910	-0.36637623
C3	3.42679785	-3.14233590	-0.37138995
C4	2.67635504	-2.72456757	0.89820521
H5	1.60976701	-2.81210895	0.64463665
C6	2.81155719	-1.30908780	1.39087308
O7	3.55787765	-0.46906461	0.94023404
O8	1.94579685	-1.08493560	2.38236973
C9	1.92888956	0.24902855	2.90575962
H10	1.13259939	0.26161581	3.64744522
H11	2.89417511	0.48086208	3.36195924
H12	1.71060810	0.95586622	2.10423858
C13	3.59436797	-4.65220045	-0.15615851
H14	4.33766180	-5.13397081	-0.79459585
C15	3.91960115	-4.78876957	1.33883574
H16	4.89992788	-4.32059803	1.51569235
C17	2.88100524	-3.82970814	1.99774133
C18	3.31441630	-3.36839683	3.39175608
O19	4.31870032	-2.73777695	3.65295545
C20	1.55102454	-4.55122417	2.27277037
O21	0.82901812	-5.03654260	1.43061826
C22	2.32058980	-3.85133267	4.38341653
C23	1.29541073	-4.53375176	3.73495246
C24	0.22196286	-5.06972387	4.43709277
C25	0.21542634	-4.90672600	5.81922654
C26	1.25262512	-4.22540815	6.47491623
C27	2.32096473	-3.68601698	5.76449107
H28	-0.57561221	-5.58847911	3.91390669
H29	-0.60497728	-5.31050157	6.40585971
H30	1.21445366	-4.11674340	7.55517913
H31	3.12714584	-3.15194676	6.25913779
C32	4.01106520	-8.69350035	3.17203560
C33	4.79681454	-7.65570645	3.66805989
C34	4.76456889	-6.40727727	3.05505368
C35	3.94804454	-6.17299740	1.94340646
C36	3.16070853	-7.21953861	1.45594748
C37	3.19555172	-8.47137752	2.06575775
H38	4.03638928	-9.67156668	3.64443051
H39	5.43846233	-7.81944704	4.52957708
H40	5.38379295	-5.59780549	3.43904241
H41	2.51032974	-7.05850441	0.60078474
H42	2.58097681	-9.27627040	1.67202101
C43	3.38172235	1.31923160	-3.54091483
C44	4.25547817	1.53530687	-2.47779080
C45	4.70632081	0.46616246	-1.70938598
C46	4.28798026	-0.83254573	-2.01184788
C47	3.40259688	-1.04451566	-3.07457865
C48	2.95022948	0.02865687	-3.83523225
H49	3.02244957	2.15859419	-4.12983776
H50	4.57957552	2.54315086	-2.23437296
H51	5.35949903	0.64767956	-0.86199846
H52	3.04328632	-2.04324132	-3.29542473
H53	2.24436321	-0.14575929	-4.64246915
C54	7.04987112	-5.37393467	-3.63694765
C55	7.39793734	-5.16847681	-2.30301902
C56	6.73148331	-4.20840477	-1.55147185
C57	5.70296894	-3.44256711	-2.12200163
C58	5.35187535	-3.66313391	-3.45484946
C59	6.02883154	-4.62181099	-4.20700270
H60	7.57463293	-6.11936578	-4.22817935
H61	8.19080011	-5.75369257	-1.84603300
H62	7.01814570	-4.05276885	-0.51455736
H63	4.55334892	-3.10812923	-3.92692692
H64	5.74744338	-4.77598996	-5.24478680
C65	8.46328567	-0.76825827	1.41448973
C66	8.46856336	-0.71361731	0.01977404
C67	7.36419611	-1.15773308	-0.69673461
C68	6.23836230	-1.64639429	-0.01888482
C69	6.23612182	-1.69864507	1.37127063
C70	7.35155875	-1.26365870	2.08652939
H71	9.32966580	-0.42465090	1.97309173
H72	9.33634363	-0.32916799	-0.50872698
H73	7.37677783	-1.12425921	-1.78446029
H74	5.37736296	-2.07356124	1.91505161
H75	7.33950939	-1.31208804	3.17182734
H76	2.34266424	-3.01174286	-1.25610013
C77	-2.56053241	-1.73542901	2.96106539
C78	-3.28776989	-2.80559061	3.51067463

C79	-3.26300823	-4.06200396	2.91489270
C80	-2.51388320	-4.30209607	1.74967543
H81	-2.56767339	-0.74948029	3.41788522
H82	-3.87610665	-2.65093067	4.41069266
H83	-3.83337715	-4.87424428	3.35751961
H84	-2.48262832	-5.28480571	1.28846888
C85	-1.81357869	-3.24016527	1.21650583
C86	-1.83379523	-1.97442720	1.81678731
C87	-0.94365263	-1.05875582	0.99973358
O88	-0.70269085	0.10907318	1.23811959
C89	-0.92830651	-3.13116807	0.03351431
C90	-0.42233344	-1.88711164	-0.10737947
C91	0.52104346	-1.52420725	-1.20911806
C92	1.04909565	-2.82340454	-1.81843466
O93	-0.67968541	-4.18863996	-0.74179917
H94	1.37948132	-2.71143317	-2.84467806
C95	0.51420022	-5.19532421	-2.43319825
C96	0.31288074	-4.04512924	-1.70767393
H97	-0.07906625	-6.07207052	-2.20812494
C98	1.53842890	-5.30259685	-3.43726338
O99	1.56868374	-6.55534500	-3.97633971
O100	2.33080485	-4.44374087	-3.81663206
C101	2.58495577	-6.76451922	-4.94741207
H102	2.50320753	-7.80970199	-5.24824217
H103	2.44016765	-6.11141609	-5.81271383
H104	3.57334382	-6.56681593	-4.52302206
C105	-1.38957983	1.12665047	-4.04067390
C106	-0.57625062	1.64212880	-3.03439053
C107	0.03819720	0.78487952	-2.12514786
C108	-0.14895173	-0.59469579	-2.21155795
C109	-0.96713474	-1.10421986	-3.22163427
C110	-1.58482268	-0.25026285	-4.13090879
H111	-1.86834336	1.79355202	-4.75264377
H112	-0.41735199	2.71443485	-2.95828236
H113	0.67680231	1.18399629	-1.34022682
H114	-1.11067206	-2.18020374	-3.30046137
H115	-2.21700882	-0.66046485	-4.91385641
H116	1.37009166	-0.95875579	-0.78516467

van der Waals complex of int3-5 with 3b
E(M06-2X/6-31G(d)(THF)) = -3256.614050605
E(M06-2X/6-311+G(d,p)) = -3257.39040483

P1	4.81363731	-2.47932311	-1.38594085
H2	2.69472670	-5.40345800	-0.56458302
C3	3.34103147	-3.38745955	-0.72204403
C4	2.45679577	-2.82932913	0.42062134
H5	1.42836336	-2.92114472	0.05321081
C6	2.60739062	-1.38489428	0.83085071
O7	3.49399911	-0.63439361	0.49383549
O8	1.61469057	-1.04934157	1.65443132
C9	1.66557869	0.28003593	2.18869051
H10	0.79005896	0.36944127	2.82678466
H11	2.59184005	0.41274039	2.75315769
H12	1.61634230	1.00712291	1.37597864
C13	3.59587692	-4.84570837	-0.28749313
H14	4.44852354	-5.32761903	-0.77068440
C15	3.73978308	-4.76550937	1.23346914
H16	4.67029303	-4.22670852	1.46226423
C17	2.58732290	-3.80229900	1.64530810
C18	2.84522828	-3.12576415	3.00047533
O19	3.82362079	-2.46090358	3.27770862
C20	1.26057288	-4.54991448	1.88218348
O21	0.69082773	-5.24065055	1.07015342
C22	1.72340865	-3.43948106	3.91191856
C23	0.80846192	-4.26974984	3.27009159
C24	-0.34250269	-4.70838236	3.91371406
C25	-0.55555412	-4.27160028	5.21782458
C26	0.36559210	-3.43253433	5.86363305
C27	1.52391011	-3.00860742	5.21946686
H28	-1.04783519	-5.35596309	3.40403738
H29	-1.45164139	-4.58259637	5.74768957
H30	0.16645538	-3.11094577	6.88214359
H31	2.24717309	-2.36103505	5.70677899
C32	3.78671945	-8.38911401	3.57111422
C33	4.42578881	-7.23816376	4.02657483
C34	4.41437006	-6.08524981	3.24837176
C35	3.76324437	-6.06176117	2.01058917
C36	3.12136743	-7.21897493	1.56368583
C37	3.13746091	-8.37585573	2.33949491
H38	3.79568395	-9.29298015	4.17387841
H39	4.93630536	-7.23888913	4.98574193
H40	4.91975280	-5.18733470	3.60119952
H41	2.59523925	-7.22140957	0.61347291
H42	2.63588255	-9.26934474	1.97836313
C43	3.31191614	1.27400794	-3.55759626
C44	4.34514282	1.37959110	-2.62763186
C45	4.81796374	0.24701814	-1.97521013
C46	4.26234365	-1.00085585	-2.27274596
C47	3.23536045	-1.11503854	-3.21601608
C48	2.75918197	0.03028299	-3.84993909
H49	2.93382790	2.16539686	-4.05050659
H50	4.77206916	2.35043295	-2.39323557
H51	5.58860458	0.34160453	-1.21618844
H52	2.81279272	-2.08821487	-3.45805740
H53	1.95001802	-0.05718091	-4.56930781
C54	6.88708594	-5.36678067	-4.33429445
C55	7.11353412	-5.46636474	-2.96109420
C56	6.48108630	-4.58939081	-2.08803633
C57	5.59958710	-3.61424681	-2.57919529
C58	5.37323293	-3.52056030	-3.95370672
C59	6.02489213	-4.39124411	-4.82530553
H60	7.38874046	-6.04554374	-5.01821511
H61	7.79071356	-6.21948918	-2.56892136
H62	6.69117437	-4.65418846	-1.02246672
H63	4.69555981	-2.77786445	-4.35607784
H64	5.84682506	-4.30564055	-5.89336722
C65	8.36413079	-1.28317092	1.28467847
C66	8.49901651	-1.36585425	-0.10133883
C67	7.41711883	-1.74903213	-0.88448950
C68	6.18213515	-2.03583920	-0.28197307
C69	6.04686534	-1.93612489	1.10225840
C70	7.14180655	-1.57163653	1.88250644
H71	9.21381347	-0.99011476	1.89517263
H72	9.44962586	-1.13600668	-0.57365060
H73	7.53374814	-1.82087828	-1.96327916
H74	5.09980676	-2.11773000	1.59188665
H75	7.02419653	-1.50517823	2.96032298
H76	2.74302354	-3.39479985	-1.64707454
C77	-3.01545118	-2.15049937	2.97996940
C78	-3.70447932	-3.29851688	3.41169829
C79	-3.63175515	-4.48550013	2.69047194

C80	-2.86008970	-4.57886710	1.51823331
H81	-3.07241725	-1.21421766	3.52854734
H82	-4.30987193	-3.25546948	4.31303424
H83	-4.18087793	-5.35676121	3.03773860
H84	-2.79168927	-5.50635878	0.95696539
C85	-2.19189846	-3.44440600	1.10738404
C86	-2.27389990	-2.24416066	1.82432860
C87	-1.46700869	-1.19970112	1.07285983
O88	-1.30019327	-0.04521067	1.42230503
C89	-1.33063018	-3.17591531	-0.07000970
C90	-0.93726197	-1.87987804	-0.12113176
C91	-0.15532985	-1.32750325	-1.27413493
C92	0.27066882	-2.50329178	-2.12059317
O93	-1.00143057	-4.14423195	-0.90888645
H94	0.91722920	-2.31131100	-2.96299508
C95	0.48100338	-4.99545005	-2.48120759
C96	-0.04268525	-3.80641626	-1.89943911
H97	0.01834180	-5.93313590	-2.20467169
C98	1.63445841	-5.02249147	-3.26506198
O99	1.99839736	-6.30988283	-3.62210547
O100	2.36155300	-4.07139365	-3.63811723
C101	3.27198524	-6.42107308	-4.22449550
H102	3.39097529	-7.47321575	-4.49197938
H103	3.35290584	-5.79608349	-5.11861691
H104	4.07013917	-6.12302032	-3.53203879
C105	-2.50037564	1.65868578	-3.35305208
C106	-1.61996357	2.03537407	-2.34133713
C107	-0.86041586	1.07158849	-1.68395054
C108	-0.96835703	-0.27637662	-2.02755255
C109	-1.85341025	-0.64610466	-3.03992307
C110	-2.61537770	0.31417104	-3.70000887
H111	-3.09324480	2.40925377	-3.86896437
H112	-1.52623767	3.08177899	-2.06253028
H113	-0.18161412	1.36141910	-0.88492160
H114	-1.93197344	-1.69622135	-3.31025439
H115	-3.30017102	0.01206818	-4.48831166
H116	0.73594530	-0.79398477	-0.89366214

TS-deprot-5
E(M06-2X/6-31G(d)(THF)) = -3256.587752927
E(M06-2X/6-311+G(d,p)) = -3257.365774349

P1	4.73123268	-2.39148316	-0.88922635
H2	2.96550296	-5.48754029	-0.04359411
C3	3.42675042	-3.40325188	-0.06640455
C4	2.70964066	-2.90305615	1.18330129
H5	1.40508463	-2.88261275	0.63515813
C6	2.80730311	-1.51008136	1.57086806
O7	3.36443228	-0.61901255	0.94178800
O8	2.11774079	-1.26055566	2.70997611
C9	2.14958348	0.08772306	3.17288143
H10	1.62796556	0.08430863	4.12858457
H11	3.18152055	0.42721281	3.28930455
H12	1.62627528	0.74219941	2.47049299
C13	3.81589932	-4.86601772	0.25456251
H14	4.70037759	-5.23357405	-0.27285113
C15	3.99099841	-4.88863894	1.77179088
H16	4.91344253	-4.34277354	2.01276126
C17	2.84360831	-3.97349589	2.28663678
C18	3.17471079	-3.43200670	3.68692440
O19	4.22432444	-2.91176080	4.00640873
C20	1.52835697	-4.73211394	2.51037588
O21	0.95405929	-5.41419097	1.69037992
C22	2.03036860	-3.72548713	4.58669619
C23	1.08197550	-4.48707273	3.90789450
C24	-0.08080052	-4.92070129	4.53339893
C25	-0.28430087	-4.53606082	5.85526397
C26	0.66714037	-3.76215726	6.53683224
C27	1.84428061	-3.35598506	5.91428910
H28	-0.79964841	-5.53020794	3.99508335
H29	-1.19255711	-4.83729200	6.36943573
H30	0.47877137	-3.47995322	7.56904101
H31	2.59328273	-2.76666811	6.43544767
C32	4.27435455	-8.68514192	3.80797572
C33	4.97897940	-7.57409977	4.26468877
C34	4.88262907	-6.36376203	3.58516922
C35	4.08195385	-6.23976645	2.44463317
C36	3.37319281	-7.35914344	1.99908124
C37	3.47275162	-8.57281490	2.67488995
H38	4.34901175	-9.63350542	4.33291136
H39	5.60653826	-7.64957553	5.14862194
H40	5.43414295	-5.49536433	3.94077474
H41	2.72597663	-7.28444769	1.13035798
H42	2.91657646	-9.43386986	2.31446733
C43	2.78905937	1.12563491	-3.13372159
C44	3.98797527	1.31286389	-2.44985466
C45	4.58001036	0.25477242	-1.76927523
C46	3.97985057	-1.00630726	-1.78987345
C47	2.77514903	-1.19744194	-2.47531868
C48	2.18275069	-0.12668795	-3.13952698
H49	2.32085938	1.95920029	-3.64955802
H50	4.45676160	2.29240034	-2.42715134
H51	5.48587786	0.42656624	-1.19697255
H52	2.27464704	-2.16196279	-2.49036890
H53	1.23718226	-0.27798531	-3.65176371
C54	6.37765032	-5.40172612	-3.97762465
C55	7.13524815	-5.08154402	-2.85415088
C56	6.66098401	-4.15157611	-1.93341952
C57	5.42498607	-3.52677389	-2.13807037
C58	4.65647211	-3.86698451	-3.25825524
C59	5.13775736	-4.79838259	-4.17343580
H60	6.75010659	-6.12577975	-4.69676773
H61	8.09739916	-5.55750032	-2.68835621
H62	7.25496009	-3.92086442	-1.05420085
H63	3.67624561	-3.42968886	-3.41891946
H64	4.53837281	-5.05090002	-5.04334757
C65	8.31583730	-0.91431545	1.57048580
C66	8.35769094	-0.88597066	0.17631442
C67	7.27258901	-1.34566387	-0.56053749
C68	6.13098520	-1.82256318	0.10039758
C69	6.08879656	-1.84257987	1.49595086
C70	7.18705792	-1.39600589	2.22613430
H71	9.16705745	-0.55828323	2.14419954
H72	9.23700445	-0.50931416	-0.33795262
H73	7.31383185	-1.33323022	-1.64792344
H74	5.21554053	-2.19770240	2.03094539
H75	7.14631177	-1.42130723	3.31115299
C76	-2.90866646	-1.90782311	4.94622271
C77	-3.69876509	-2.97957821	5.40018554
C78	-3.84473782	-4.12935167	4.63226869
C79	-3.20223422	-4.26097856	3.38825316
H80	-2.79152853	-0.99994955	5.53154345

H81	-4.20768293	-2.90643145	6.35718007
H82	-4.46803496	-4.94093915	4.99784772
H83	-3.31271490	-5.16119658	2.79001726
C84	-2.42485670	-3.20528599	2.95890430
C85	-2.28952150	-2.03955599	3.72495388
C86	-1.41136439	-1.07448071	2.95205321
O87	-1.08593702	0.04115239	3.31121024
C88	-1.61144105	-2.99835866	1.73748779
C89	-1.05070047	-1.77175553	1.70344261
C90	-0.27136702	-1.31545689	0.51364896
C91	0.27116815	-2.57732667	-0.14221462
O92	-1.49071472	-3.95408066	0.80968110
H93	0.75096043	-2.39566368	-1.10008466
C94	-0.46604532	-4.81696592	-1.05869163
C95	-0.54125951	-3.74736372	-0.19500695
H96	-1.09645912	-5.67615934	-0.86900194
C97	0.48766269	-4.91539391	-2.11811377
O98	0.37652875	-6.10103702	-2.78178515
O99	1.35263479	-4.10085776	-2.45664227
C100	1.32193961	-6.30092507	-3.82263993
H101	1.11989918	-7.29450662	-4.22438319
H102	1.20735057	-5.54688277	-4.60678382
H103	2.34421628	-6.24950266	-3.43599501
C104	-2.79019325	1.18414511	-1.97028213
C105	-2.22818898	1.68168233	-0.79502915
C106	-1.41291241	0.86996356	-0.01416842
C107	-1.14621901	-0.44973144	-0.39192275
C108	-1.71316114	-0.94093853	-1.56800061
C109	-2.53034548	-0.12798839	-2.35289660
H110	-3.42785401	1.81649923	-2.58211162
H111	-2.42736435	2.70467132	-0.48670999
H112	-0.98162981	1.25106383	0.90976378
H113	-1.51666897	-1.96515351	-1.87528316
H114	-2.96650058	-0.52591409	-3.26567501
H115	0.56636860	-0.68568270	0.83936597
H116	2.71652317	-3.44414924	-0.91314702

Int4-5

E(M06-2X/6-31G(d)(THF)) = -2146.275255248
E(M06-2X/6-311+G(d,p)) = -2146.762323312
G(M06-2X/6-311+G(d,p)(THF)) = -2146.24498

P1	4.83576918	-2.41218520	-0.98482518
H2	2.30212433	-5.10142043	-0.33303407
C3	3.18980303	-3.16897653	-0.35538372
C4	2.65887650	-2.58748818	0.89612880
C5	2.36971232	-1.23222792	0.99757159
O6	2.61230915	-0.36684445	0.14014188
O7	1.82711934	-0.88186411	2.22030241
C8	1.71071813	0.51284627	2.44177278
H9	1.26315722	0.62569466	3.43012121
H10	2.69206818	0.99830701	2.41765698
H11	1.07584267	0.98474286	1.68579575
C12	3.27440762	-4.67985625	-0.06552293
H13	4.04324868	-5.22520256	-0.61804075
C14	3.47098727	-4.74546812	1.45292633
H15	4.47640105	-4.36301490	1.68593501
C16	2.51394120	-3.63266267	1.96942258
C17	2.88903015	-3.20028100	3.39370817
O18	3.98989970	-2.85354075	3.77232285
C19	1.05732192	-4.11110774	2.12074667
O20	0.36241366	-4.59246853	1.24868162
C21	1.69164102	-3.34959715	4.26355228
C22	0.63017093	-3.87042919	3.52904913
C23	-0.60351206	-4.11775015	4.12274707
C24	-0.74130970	-3.82271775	5.47604482
C25	0.33097772	-3.30080260	6.21601059
C26	1.56443609	-3.06095350	5.61799677
H27	-1.42357235	-4.52651651	3.53933465
H28	-1.69134926	-3.99889013	5.97329874
H29	0.19030783	-3.08313370	7.27136787
H30	2.40514426	-2.66052690	6.17784963
C31	3.05552919	-8.52886876	3.48884241
C32	3.98097158	-7.58189322	3.92177438
C33	4.10975940	-6.37447339	3.24187571
C34	3.31980211	-6.08994576	2.12295041
C35	2.38954408	-7.04498173	1.70118655
C36	2.26138836	-8.25561896	2.37844360
H37	2.95326476	-9.47430926	4.01481196
H38	4.60462887	-7.78367877	4.78850964
H39	4.83003706	-5.63247382	3.58150513
H40	1.74945002	-6.84067885	0.84773150
H41	1.53453913	-8.98768747	2.03681646
C42	3.91858506	1.04756397	-3.86810605
C43	4.89720588	1.20024982	-2.88971342
C44	5.17496596	0.16106911	-2.00789318
C45	4.47194482	-1.04110597	-2.10588850
C46	3.48099277	-1.19067591	-3.08266147
C47	3.21012957	-0.14754604	-3.96228186
H48	3.70280667	1.86290622	-4.55286947
H49	5.44349844	2.13498488	-2.80473418
H50	5.92317535	0.29710666	-1.23334535
H51	2.91193586	-2.11334821	-3.16675613
H52	2.43954398	-0.26927547	-4.71725971
C53	7.00917359	-5.75354199	-3.31246854
C54	7.13864408	-5.64787849	-1.92729807
C55	6.50254577	-4.61812993	-1.24542120
C56	5.72951455	-3.67871999	-1.94517436
C57	5.60563169	-3.78777946	-3.33217616
C58	6.24472066	-4.82424564	-4.01092825
H59	7.50769312	-6.55871191	-3.84474111
H60	7.73682287	-6.36824361	-1.37654680
H61	6.61211814	-4.54133004	-0.16552461
H62	5.02974616	-3.05737028	-3.89210324
H63	6.14729282	-4.89821417	-5.09045698
C64	7.99432547	-1.01195182	2.05254651
C65	8.35100653	-1.29487087	0.73247510
C66	7.38744694	-1.72488937	-0.16933760
C67	6.05006420	-1.85973636	0.24087174
C68	5.69206915	-1.55433745	1.55503080
C69	6.67259258	-1.14680493	2.45944621
H70	8.75307010	-0.68915351	2.76056662
H71	9.38267792	-1.18955600	0.40956169
H72	7.67394834	-1.96020100	-1.19136541
H73	4.66850535	-1.66170177	1.88595670
H74	6.38716640	-0.94125898	3.48665735
H75	2.57773682	-2.96485156	-1.24596280

Int2-6
E(M06-2X/6-31G(d)(THF)) = -2146.244006512
E(M06-2X/6-311+G(d,p)) = -2146.73087007
G(M06-2X/6-311+G(d,p)(THF)) = -2146.20970

H1	1.85039688	-4.74061018	0.01026314
C2	2.46574011	-2.72876413	-0.02949450
C3	1.43938628	-2.53668490	-1.05275256
H4	0.64533884	-3.26083107	-1.17649412
C5	1.43973643	-1.31873816	-1.68754163
O6	2.38313648	-0.47875729	-1.53489234
O7	0.43764865	-1.03839117	-2.55393590
C8	0.56583562	0.19092916	-3.26123342
H9	-0.32496407	0.26719632	-3.88603414
H10	0.61662325	1.03606982	-2.57075386
H11	1.46450030	0.19125761	-3.88447492
C12	2.81236961	-4.22800020	0.12819311
H13	3.42361942	-4.53115413	-0.72977517
C14	3.44207726	-4.75887138	1.44337644
H15	4.52745565	-4.62713370	1.42261703
C16	2.88262540	-3.96316982	2.57133442
C17	2.99516618	-4.20105460	4.02595194
O18	3.54636847	-5.11928860	4.59835811
C19	2.24142419	-2.78492765	2.39369449
O20	1.98273778	-2.14482135	1.25935262
C21	2.29474861	-3.03434810	4.69516475
C22	1.83662026	-2.17772587	3.68826357
C23	1.16212374	-1.00982713	3.98242714
C24	0.94926860	-0.71342076	5.34032188
C25	1.40341730	-1.56365350	6.34268705
C26	2.09107869	-2.74873341	6.02619147
H27	0.80840637	-0.34874160	3.19562956
H28	0.41933626	0.19565009	5.61048123
H29	1.22485207	-1.30720744	7.38294469
H30	2.45377585	-3.41965596	6.80016433
C31	2.70330790	-9.01576368	1.60082955
C32	3.92066944	-8.52550961	1.13519937
C33	4.15656648	-7.15242004	1.10921375
C34	3.18412586	-6.25231100	1.54521662
C35	1.96525421	-6.75284487	2.00845544
C36	1.72589984	-8.12322009	2.03869704
H37	2.51783326	-10.08570877	1.62674658
H38	4.69082517	-9.21282633	0.79556124
H39	5.11109279	-6.76859846	0.75302245
H40	1.20061927	-6.06044600	2.35461928
H41	0.77480752	-8.49627249	2.40903322
P42	3.95681323	-1.56824670	-0.43048545
C43	5.92807280	-1.73323574	-4.60503593
C44	6.56626638	-1.11046288	-3.53532393
C45	5.95265539	-1.06859939	-2.28679133
C46	4.67867396	-1.61825997	-2.11082108
C47	4.03924364	-2.24200052	-3.18437272
C48	4.67059428	-2.30392951	-4.42393314
H49	6.41274525	-1.77880213	-5.57625154
H50	7.54922993	-0.66706084	-3.66587284
H51	6.47363711	-0.61269691	-1.44772326
H52	3.05815763	-2.68732203	-3.04790193
H53	4.17222741	-2.80002011	-5.25197561
C54	4.18144113	2.66244420	1.43340073
C55	3.78028070	1.59122120	2.22404971
C56	3.70541757	0.30853552	1.68578556
C57	4.02919848	0.08754305	0.34495376
C58	4.39429454	1.17495056	-0.45638818
C59	4.48358752	2.44985087	0.09012931
H60	4.24412705	3.66122621	1.85606216
H61	3.51840548	1.74431589	3.26724736
H62	3.36842182	-0.50115474	2.32013805
H63	4.57766956	1.03193668	-1.51617774
H64	4.77334884	3.28314781	-0.54366367
C65	7.32233404	-4.02715783	1.70560042
C66	6.67163202	-3.01213214	2.40007335
C67	5.70320791	-2.23581610	1.76661345
C68	5.35224122	-2.46708864	0.43190262
C69	6.02844295	-3.48671912	-0.25850377
C70	7.00398105	-4.25567880	0.36849042
H71	8.07581839	-4.63285766	2.20103322
H72	6.91737376	-2.81798682	3.44018683
H73	5.22693912	-1.44625559	2.33511731
H74	5.79408709	-3.69227651	-1.29899019
H75	7.51219915	-5.03727663	-0.18985538

Int2-7
E(M06-2X/6-31G(d)(THF)) = -2146.267470506
E(M06-2X/6-311+G(d,p)) = -2146.753478952
G(M06-2X/6-311+G(d,p)(THF)) = -2146.22742

P1	-1.46219930	-6.42645830	-4.60782774
C2	-1.19967753	-4.97110447	-3.75489499
C3	-1.83722544	-3.57461542	-5.76030161
C4	-1.14186754	-3.64657179	-4.41023601
O5	-1.36646343	-3.99675353	-6.79049504
H6	-1.58034590	-2.89173756	-3.74299691
O7	-3.04789147	-3.01819752	-5.67249357
C8	-3.79050241	-3.00701509	-6.89807605
H9	-4.78021842	-2.63057765	-6.64215360
H10	-3.30457344	-2.35053620	-7.62376769
H11	-3.85396779	-4.01844372	-7.30636212
C12	0.50966768	-4.67091478	-1.81579406
H13	0.48547539	-4.45922545	-0.73788715
C14	2.95633302	-8.21746203	-2.29203204
C15	2.84007440	-7.30774387	-3.34115496
C16	2.07474206	-6.15435003	-3.18280641
C17	1.39829210	-5.89702883	-1.98820241
C18	1.52246230	-6.81700431	-0.94392736
C19	2.29783871	-7.96441000	-1.08905954
H20	3.56231370	-9.11228537	-2.40667795
H21	3.34260014	-7.49225364	-4.28740373
H22	2.00335428	-5.44218767	-3.99842194
H23	1.00639450	-6.62724230	-0.00472511
H24	2.39167616	-8.66148664	-0.26014901
C25	1.14737199	-3.48593583	-2.47621875
C26	2.91093773	-1.87054761	-2.79075482
C27	2.12394310	-1.92699588	-3.94128045
C28	2.39830761	-1.13427426	-5.03730827
C29	3.50660941	-0.27461523	-4.95243426
C30	4.29101017	-0.21815031	-3.80432813
C31	3.99390704	-1.02462355	-2.69316928
H32	1.78282732	-1.17679903	-5.93091303
H33	3.75573078	0.35836849	-5.79968126
H34	5.14148583	0.45663046	-3.76904475
H35	4.59406273	-0.99117888	-1.78801930
C36	1.04303464	-2.93160699	-3.71458901

O37	0.19773483	-3.11121517	-4.72835955
C38	2.32459303	-2.85506430	-1.81526954
O39	2.73797790	-3.09358838	-0.69740539
C40	-0.94840338	-4.94362041	-2.26209764
H41	-1.56544677	-4.14103396	-1.82836945
H42	-1.27233336	-5.87329661	-1.78137637
C43	1.75821531	-7.99802174	-7.53806494
C44	1.69466295	-6.64454819	-7.21436521
C45	0.72374608	-6.18116142	-6.32965082
C46	-0.19219336	-7.07221826	-5.76669831
C47	-0.12959773	-8.43042977	-6.10002731
C48	0.84462428	-8.89142780	-6.98108627
H49	2.52045924	-8.35841968	-8.22375455
H50	2.40388886	-5.94594111	-7.64953013
H51	0.67183389	-5.12476664	-6.07750076
H52	-0.83792605	-9.13295066	-5.66655640
H53	0.89149022	-9.94808626	-7.23016992
C54	-2.06610193	-9.79491863	-1.50457423
C55	-3.16834027	-9.09997740	-1.99732761
C56	-2.99495860	-8.09833337	-2.94839110
C57	-1.71438117	-7.78420426	-3.41406372
C58	-0.61037432	-8.48147122	-2.91346031
C59	-0.78910154	-9.48243758	-1.96286712
H60	-2.20369714	-10.57974004	-0.76560702
H61	-4.16779747	-9.34077732	-1.64611356
H62	-3.86335786	-7.57093085	-3.33064599
H63	0.39088984	-8.24852753	-3.26330772
H64	0.07862444	-10.01318080	-1.58148268
C65	-5.47744715	-6.44881859	-6.91250832
C66	-4.36037017	-7.03882867	-7.50076675
C67	-3.12832161	-7.01330813	-6.85060141
C68	-3.00673253	-6.38988059	-5.60605127
C69	-4.12408701	-5.76941094	-5.03538987
C70	-5.35791880	-5.81027412	-5.67964402
H71	-6.43855204	-6.48040754	-7.41873195
H72	-4.44693612	-7.52331946	-8.46944527
H73	-2.26388183	-7.47301268	-7.31983724
H74	-4.01581031	-5.25125960	-4.08511045
H75	-6.22232154	-5.33689173	-5.22175886

TS-prot-7
E(M06-2X/6-31G(d)(THF)) = -3256.556052178
E(M06-2X/6-311+G(d,p)) = -3257.332129510

C1	-1.12050357	-1.37673516	0.61418177
C2	-0.68829322	-1.92814978	1.83330763
C3	-1.22055197	-3.12324462	2.30093158
C4	-2.21181248	-3.81339334	1.58036957
H5	-0.69947019	-0.45443596	0.22303661
H6	0.08274174	-1.42476411	2.40903059
H7	-0.85930033	-3.53777930	3.23767572
H8	-2.62270323	-4.75054788	1.94480189
C9	-2.63928722	-3.25501127	0.39390802
C10	-2.09357672	-2.05404969	-0.08478736
C11	-2.72753187	-1.75682948	-1.42698843
O12	-2.41935192	-0.85904267	-2.18577279
C13	-3.61896397	-3.70113386	-0.62539797
C14	-3.71172832	-2.83804017	-1.65630969
C15	-4.55813276	-3.15518295	-2.84448583
C16	-4.57164255	-4.69097814	-2.96157630
O17	-4.22875191	-4.89306252	-0.54865335
H18	-5.25819184	-5.04036445	-3.72922660
C19	-5.31347552	-6.65994675	-1.53163142
C20	-4.75954989	-5.41919107	-1.74609896
H21	-5.24714209	-7.09315297	-0.54169569
C22	-6.02012063	-7.40139448	-2.53729584
O23	-6.50982249	-8.57341601	-2.03623929
O24	-6.20032944	-7.11180594	-3.71795152
C25	-7.24523011	-9.36329739	-2.96437502
H26	-7.55243201	-10.25754310	-2.42066556
H27	-8.12258243	-8.82262320	-3.32863614
H28	-6.62434191	-9.63654686	-3.82300112
H29	-4.10428426	-2.72084451	-3.73940819
C30	-8.59383100	-1.60774904	-2.63860269
C31	-7.80364231	-1.36434980	-3.75741182
C32	-6.49715076	-1.84843675	-3.80722768
C33	-5.96407779	-2.57897290	-2.74604560
C34	-6.76602987	-2.81599596	-1.62522846
C35	-8.06890843	-2.33543559	-1.57058880
H36	-9.61275882	-1.23298501	-2.59601412
H37	-8.20251774	-0.79542913	-4.59317233
H38	-5.87759494	-1.65356833	-4.68083649
H39	-6.36809358	-3.38595372	-0.78799900
H40	-8.67906846	-2.52988537	-0.69257205
P41	-1.60935074	-6.75827514	-4.95878809
C42	-1.86685791	-5.40633158	-3.78804539
C43	-2.02270529	-3.71076997	-5.66975737
C44	-1.55085103	-3.98224144	-4.24114824
O45	-1.29866497	-3.66303275	-6.63398379
H46	-2.10264523	-3.31704781	-3.56585285
O47	-3.34350332	-3.56580078	-5.71560831
C48	-3.89972155	-3.37584365	-7.02315123
H49	-4.97918146	-3.35729549	-6.88109783
H50	-3.54417182	-2.43285892	-7.44463877
H51	-3.61002164	-4.20472532	-7.67214831
C52	-0.14813088	-5.44643530	-1.67829328
H53	-0.32304101	-5.52903539	-0.59642939
C54	2.80052693	-8.46815128	-2.62709956
C55	2.65808809	-7.35576810	-3.45348915
C56	1.73526224	-6.36242101	-3.13215270
C57	0.91556746	-6.48214671	-2.01026652
C58	1.07382546	-7.59703598	-1.18245721
C59	2.01327006	-8.57924179	-1.48128067
H60	3.53008149	-9.23715988	-2.86579067
H61	3.26835904	-7.25291567	-4.34712304
H62	1.65271077	-5.48080324	-3.76076276
H63	0.45142112	-7.69426332	-0.29519251
H64	2.13171047	-9.43326731	-0.81945096
C65	0.35072422	-4.05758901	-1.89960330
C66	1.60901170	-2.05077429	-1.48819689
C67	1.10439341	-2.02422425	-2.79252506
C68	1.32722866	-0.94754074	-3.62604803
C69	2.08858388	0.11712639	-3.11579990
C70	2.59384864	0.08992179	-1.81954850
C71	2.35291653	-1.00929154	-0.97915160
H72	0.92746966	-0.92409118	-4.63544906
H73	2.28493583	0.97997298	-3.74602264
H74	3.17908373	0.92900885	-1.45490104
H75	2.73668804	-1.04556213	0.03692127
C76	0.34843734	-3.28653896	-3.00653283
O77	-0.18814108	-3.53015687	-4.22216491

C78	1.17148942	-3.34952554	-0.86787638
O79	1.44830191	-3.76432377	0.23688370
C80	-1.53077917	-5.70572429	-2.31443399
H81	-2.21125149	-5.06462585	-1.75469845
H82	-1.83332922	-6.73091096	-2.07775075
C83	2.25016574	-7.38312575	-7.41199260
C84	1.93463326	-6.11152909	-6.94105948
C85	0.79377078	-5.91070311	-6.16694308
C86	-0.03452385	-6.99143285	-5.85538573
C87	0.27194187	-8.26621857	-6.35182808
C88	1.41450580	-8.45989613	-7.12045321
H89	3.14267062	-7.53554035	-8.01279330
H90	2.57583157	-5.26648474	-7.17538667
H91	0.55358766	-4.91213320	-5.81772421
H92	-0.38231259	-9.10915386	-6.14577156
H93	1.64829144	-9.45235403	-7.49512660
C94	-2.28744894	-10.49989552	-2.36218735
C95	-3.37934317	-9.79682592	-2.86456842
C96	-3.18269523	-8.70059649	-3.69928380
C97	-1.88288478	-8.29314694	-4.01937811
C98	-0.78634465	-8.98956329	-3.49883622
C99	-0.99375119	-10.09644426	-2.68208928
H100	-2.44548736	-11.35752659	-1.71404521
H101	-4.39066816	-10.08733103	-2.59625815
H102	-4.04924637	-8.17391564	-4.08530235
H103	0.22887345	-8.66853329	-3.71514342
H104	-0.13544301	-10.63044833	-2.28465906
C105	-4.81036625	-6.79985012	-8.27986446
C106	-3.46422401	-6.90387432	-8.62463115
C107	-2.47955696	-6.84982192	-7.64082447
C108	-2.84431690	-6.68241663	-6.29939398
C109	-4.19501788	-6.54017447	-5.96356814
C110	-5.17442053	-6.61418284	-6.94791145
H111	-5.57349313	-6.85745345	-9.05129901
H112	-3.17468635	-7.03205944	-9.66373237
H113	-1.43609654	-6.93352924	-7.92562806
H114	-4.50586398	-6.38657574	-4.93838968
H115	-6.21776112	-6.52815591	-6.65860447
H116	-3.21584126	-5.20469144	-3.55887498

Van der Waals complex of Int3-7 with 3b
E(M06-2X/6-31G(d)(THF)) = -3256.582616420
E(M06-2X/6-311+G(d,p)) = -3257.357288897

C1	-1.41199391	-1.03196772	-0.50483169
C2	-0.70276649	-1.57440074	0.58194615
C3	-1.05004580	-2.81118629	1.11399653
C4	-2.11251417	-3.56017197	0.57679046
H5	-1.14362976	-0.06921238	-0.92953377
H6	0.12991439	-1.02340623	1.00976604
H7	-0.48093895	-3.21305514	1.94750725
H8	-2.38364304	-4.52897586	0.98952355
C9	-2.79943467	-3.01632584	-0.48980147
C10	-2.46106036	-1.76176577	-1.01673347
C11	-3.41591018	-1.46719408	-2.16185268
O12	-3.39608537	-0.47522550	-2.86620775
C13	-3.91523041	-3.53314055	-1.32068247
C14	-4.30816211	-2.64060851	-2.25717990
C15	-5.44221764	-2.94302689	-3.18688168
C16	-5.54224089	-4.44698827	-3.25677945
O17	-4.35593798	-4.78136529	-1.19000153
H18	-6.09992959	-4.90026762	-4.06653978
C19	-5.22465836	-6.71022616	-2.18022374
C20	-5.11122176	-5.29217502	-2.28692360
H21	-4.68330063	-7.21332433	-1.38904643
C22	-6.12003395	-7.44086032	-2.97653731
O23	-6.08945068	-8.80198759	-2.68271477
O24	-6.86501062	-7.03975785	-3.88566101
C25	-6.93263796	-9.59718703	-3.49744003
H26	-6.79976769	-10.62687300	-3.15802736
H27	-7.98143911	-9.30485113	-3.39357713
H28	-6.65725946	-9.51420276	-4.55390522
H29	-5.19685634	-2.53130842	-4.17477629
C30	-9.01882466	-0.88929056	-1.85138996
C31	-7.92218476	-0.17205496	-2.33043566
C32	-6.78559571	-0.84512996	-2.76448039
C33	-6.72474527	-2.24229736	-2.72374318
C34	-7.82414004	-2.95306948	-2.24714076
C35	-8.96623803	-2.27926648	-1.81371348
H36	-9.90793152	-0.36534140	-1.51072967
H37	-7.95430937	0.91395679	-2.36404704
H38	-5.92663501	-0.28868540	-3.13553748
H39	-7.78104606	-4.03892169	-2.21847956
H40	-9.81665771	-2.84665099	-1.44410349
P41	-1.44266840	-6.92415813	-4.74708563
C42	-1.70574786	-5.57403569	-3.48268358
C43	-1.95072963	-3.78314449	-5.27937360
C44	-1.40559159	-4.11238572	-3.88928554
O45	-1.27536368	-3.73850185	-6.27860189
H46	-1.94164349	-3.49823144	-3.15691849
O47	-3.25821869	-3.59656060	-5.22636384
C48	-3.91427109	-3.38376463	-6.48551239
H49	-4.98113287	-3.42302289	-6.26972618
H50	-3.63146874	-2.40812383	-6.88705648
H51	-3.63305276	-4.17322531	-7.18510444
C52	0.04034173	-5.64862963	-1.41509042
H53	-0.10043414	-5.75770334	-0.33054164
C54	3.00219648	-8.61370531	-2.50985999
C55	2.82556649	-7.48452038	-3.30630848
C56	1.89221680	-6.51584051	-2.94346965
C57	1.09688519	-6.67628136	-1.80816288
C58	1.28533275	-7.80978756	-1.01325067
C59	2.23581146	-8.76773553	-1.35449570
H60	3.74212099	-9.36272895	-2.77862915
H61	3.41986727	-7.34730996	-4.20609326
H62	1.79025903	-5.62053545	-3.54947600
H63	0.68347394	-7.93958713	-0.11567216
H64	2.38046870	-9.63582794	-0.71649244
C65	0.56564546	-4.26650203	-1.62559475
C66	2.00651699	-2.36500901	-1.30019039
C67	1.41305132	-2.28769541	-2.56428867
C68	1.65584748	-1.22508401	-3.40966176
C69	2.52924463	-0.22368982	-2.95197561
C70	3.12203811	-0.29995172	-1.69547730
C71	2.86088314	-1.38596006	-0.84260253
H72	1.19031192	-1.16260775	-4.38878873
H73	2.74535833	0.62661318	-3.59265310
H74	3.79406552	0.48959898	-1.37165046
H75	3.31552730	-1.45896500	0.14191329
C76	0.55611507	-3.48874475	-2.72381143
O77	-0.04860744	-3.70597273	-3.91550429
C78	1.51193074	-3.62992028	-0.65211885

O79	1.83652338	-4.07746824	0.42600751
C80	-1.35726720	-5.91232868	-2.01150533
H81	-2.06403732	-5.32129487	-1.42638319
H82	-1.62418066	-6.95685128	-1.82042612
C83	2.31867265	-7.30519596	-7.35828003
C84	1.99138379	-6.05983011	-6.82805519
C85	0.88137791	-5.91581315	-5.99977622
C86	0.10103854	-7.03336084	-5.68981412
C87	0.41773279	-8.28356648	-6.24200395
C88	1.52763773	-8.41578046	-7.06886258
H89	3.18528712	-7.41035543	-8.00531290
H90	2.59622027	-5.18893756	-7.06378858
H91	0.62706989	-4.93526757	-5.61547709
H92	-0.20124222	-9.15239477	-6.03596975
H93	1.76937335	-9.38709293	-7.49051812
C94	-1.95746988	-10.75846996	-2.26587794
C95	-3.08022449	-10.06872323	-2.71615189
C96	-2.93304860	-8.93811544	-3.51323040
C97	-1.65119955	-8.48340134	-3.84424202
C98	-0.52164589	-9.16521186	-3.37399054
C99	-0.68139806	-10.30733142	-2.59722525
H100	-2.07622047	-11.64540812	-1.64924944
H101	-4.08226397	-10.38661223	-2.44617222
H102	-3.82572312	-8.41878087	-3.83874835
H103	0.47967178	-8.81212003	-3.60339243
H104	0.19953581	-10.83369092	-2.24121198
C105	-4.85950551	-6.75986820	-7.82239662
C106	-3.53815944	-6.85552330	-8.25522802
C107	-2.49736307	-6.85853594	-7.33103538
C108	-2.78309180	-6.75665000	-5.96343264
C109	-4.11076190	-6.61556908	-5.53313497
C110	-5.14493686	-6.63516435	-6.46424281
H111	-5.66929422	-6.77501036	-8.54684549
H112	-3.31203927	-6.93259283	-9.31481774
H113	-1.47265321	-6.93626210	-7.67998270
H114	-4.36847749	-6.50110447	-4.48274513
H115	-6.16400567	-6.56226671	-6.09675539
H116	-2.81225897	-5.58990293	-3.49091188

Product-3+2-α

E(M06-2X/6-31G(d)(THF)) = -1110.332937647
E(M06-2X/6-311+G(d,p)) = -1110.62149129
G(M06-2X/6-311+G(d,p)(THF)) = -1110.35918

C1	3.23921797	-3.60314697	-0.50260912
C2	2.62357625	-2.66819745	0.50116480
H3	1.65007276	-2.28014877	0.18544761
C4	3.77912956	-4.67653313	0.07888124
C5	3.63679646	-4.64154877	1.58863474
H6	4.56467499	-4.23589186	2.01868501
C7	2.52162185	-3.53662153	1.76886804
C8	2.71788490	-2.82517048	3.10806034
O9	3.62653413	-2.06884992	3.37258434
C10	1.13121653	-4.17168742	1.90924484
O11	0.46371203	-4.62794599	1.00911080
C12	1.63334731	-3.26252762	4.02819359
C13	0.72453322	-4.06321437	3.33796149
C14	-0.38582587	-4.60997779	3.97156246
C15	-0.55394469	-4.33459956	5.32593941
C16	0.35828591	-3.52505151	6.02031164
C17	1.46440702	-2.97458251	5.37892846
H18	-1.08771734	-5.22993842	3.42136780
H19	-1.40567742	-4.74851811	5.85847239
H20	0.19275968	-3.32656634	7.07552096
H21	2.17552817	-2.34366876	5.90456617
C22	2.60519804	-8.23351959	3.73616129
C23	3.38957799	-7.25162071	4.33612277
C24	3.74366044	-6.11135647	3.62025083
C25	3.31485219	-5.93521663	2.30320986
C26	2.52995566	-6.92527062	1.70788394
C27	2.17935355	-8.06873803	2.41956160
H28	2.32814762	-9.12515573	4.29175140
H29	3.72893377	-7.37378461	5.36101750
H30	4.36185180	-5.34640938	4.08777379
H31	2.17836815	-6.79168138	0.68682567
H32	1.56900809	-8.83182956	1.94463230
C33	4.54997737	-5.69043217	-0.68013340
O34	4.51341463	-5.82743175	-1.88378493
O35	5.31465884	-6.43659397	0.12766666
C36	6.08144689	-7.45467240	-0.52136267
H37	6.62054391	-7.97036268	0.27216144
H38	5.42075810	-8.14559725	-1.04975292
H39	6.77865609	-7.00931255	-1.23478252
H40	3.28544647	-1.80924551	0.68049163
H41	3.29437959	-3.39292004	-1.56618015
